# A Novel Prognostic Ferroptosis-Related Long Noncoding RNA Signature in Clear Cell Renal Cell Carcinoma

**DOI:** 10.1155/2022/6304824

**Published:** 2022-02-22

**Authors:** Zhixun Bai, Yongchao Zhao, Xiaomin Yang, Linglu Wang, Xianhua Yin, Yue Chen, Jing Lu

**Affiliations:** ^1^Department of Nephrology, The Second Affiliated Hospital of Zunyi Medical University, Zunyi, China; ^2^Department of Cardiology, Affiliated Hospital of Zunyi Medical University, Zunyi, China; ^3^Department of Nephrology, The First People's Hospital of Xiangtan City, Xiangtan, China; ^4^Department of Obstetrics and Gynecology, The Second Affiliated Hospital of Zunyi Medical University, Zunyi, China; ^5^Department of Clinical, Zunyi Medical and Pharmaceutical College, Zunyi, China

## Abstract

Clear cell renal cell carcinoma (ccRCC) is the most common primary malignancy of renal cancer in adults. Ferroptosis is critically associated with the prognosis of ccRCC. However, knowledge of long noncoding RNA- (lncRNA-) related ferroptosis that affects the prognosis of ccRCC is still insufficient. Using the LASSO regression, we created a risk model based on differentially expressed ferroptosis-related lncRNAs (FRLRS) in ccRCC. The analysis of Kaplan–Meier for survival, area under the curve (AUC) for diagnosis, nomogram for predicting overall survival, and gene expression for immune checkpoints were performed based on the screened independent prognostic factors. Nine lncRNAs were found to be associated with ccRCC prognosis. Furthermore, the prognostic AUC of the FRLRS signature was 0.78, demonstrating its usefulness in predicting ccRCC prognosis. The lncRNA risk model outperformed the standard clinical variables in predicting ccRCC prognosis. Finally, The Cancer Genome Atlas revealed that T cell functions, such as cytolytic activity, human leukocyte antigen activity, inflammation regulation, and type II interferon response coordination, are significantly different between two different risk levels of ccRCC. Immune checkpoints were also expressed differently in programmed cell death 1 receptor, inducible T cell costimulator, cytotoxic T-lymphocyte antigen-4, and leukocyte-associated immunoglobulin-like receptor 1. The nine FRLRS signature models may affect the prognosis of ccRCC.

## 1. Introduction

By far the most common type, renal cell carcinoma (RCC) is thought to originate in the renal epithelium in the kidney and affects over 400,000 individuals worldwide annually [1, 2]. Previous studies have reported that ccRCC is characterized as a highly metabolic disease, and fetal tumors are likely to be fundamental to the development of renal cancer-related deaths [3].

Currently, localized ccRCC can be treated with partial or radical nephrectomy [4], ablation [5], or active surveillance [6]. Approximately 30% of ccRCC who recur with the localized disease eventually develop metastases following curative nephrectomy [7–9], which be associated with higher mortality and requires systemic therapy. Mammalian targets of the rapamycin pathway have been developed. However, the response to treatment varies, and most ccRCC patients eventually make progression [10]. Treatment seems to have had a positive effect on the prognosis of patients based on the expression of clinical diagnostic markers. Therefore, identifying biomarkers is essential for effective and rapid intervention and treatment of ccRCC patients [11, 12].

Recently, a novel form of cell death, termed ferroptosis, was first proposed by Dixon in 2012 [13]. Ferroptosis is a form of unique iron-reliant and reactive oxygen autophagy-dependent on cell death with characteristics of cytological changes, such as diminished or decreased mitochondrial cristae and mitochondrial membrane condensed [14–17]. Mounting evidence indicates that ferroptosis is involved in diverse physiological and pathological conditions in human disease [18]. Hepatocellular carcinoma [19], gastric cancer [20, 21], and other cancers [22, 23] have been proven to be ferroptosis-related. Therefore, these include targeting ferroptosis has been suggested in cancer therapeutic [17]. Biologically, kidney disease is a metabolic disorder and is associated with the iron metabolism [24]. Recent studies demonstrated that ferroptosis is associated with ccRCC [25, 26]. Long noncoding RNAs (lncRNAs) have a critical predictive value in various cancers' occurrence, progression, and prognosis [27–29]. Wu conducted a novel ferroptosis-associated genes model based on the clinical significance in predicting pancreatic cancer [30]. An increasing number of genes related to ferroptosis have been found. However, the association between ferroptosis-related lncRNAs (FRLRS), and their prognostic value in ccRCC is yet to be understood.

Here, we provide new insights to assess the prognostic value of FRLRS in ccRCC. Using bioinformatics analysis, we established independent prognostic multiple FRLRS signatures and estimated lncRNAs in the immunotherapy response by inhibitory concentration.

## 2. Materials and Methods

### 2.1. Data Collection

**Figure 1:**
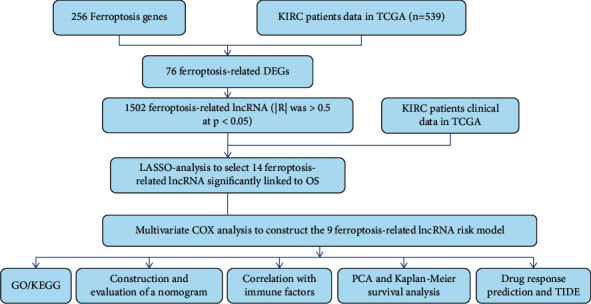
The research process flowchart.

RNA sequences of ccRCC patients' data were downloaded from TCGA-KIRC (72 patients were normal, 539 patients had tumors). Taking the corresponding genes related to ferroptosis in FerrDb [31] provides the most comprehensive database of iron bacteria and related disease markers by a web-based alliance. Our study identified 259 ferroptosis-related genes (Figure 1, Supplementary Table S1). If the correlation |*R*| was >0.5 at *p* < 0.05, the association between FRLRS and ccRCC was considered significant. The clinical information data collected from patients with ccRCC included gender, age, stage, grade, tumor-node-metastasis (TNM), survival status, and follow-up time. The additional biological function of the differentially expressed FRLRS was analyzed based on GO and KEGG data using R software via the package “cluster profile,” “ggplot2,” and “enrichplot.”

### 2.2. Construction of the FRLRS Prognostic Model

A machine learning method (LASSO) was used to identify hub genes more efficiently. Univariate Cox analysis combined with multivariate Cox regression analysis identified significant increments associated with FRLRS. LASSO was utilized to construct the FRLRS signature. The equation of risk score = (*β*1 × FRLRS − 1) + (*β*2 × FRLRS − 2) +⋯+ (*βn* × FRLRS − *n*). We established the hybrid nomogram using the selected FRLRS prognostic signature and independent factors in TCGA-KIRC. Based on the median expression levels of FRLRS, ccRCC patients were divided into different risk groups (high-risk and low-risk groups). According to the clinical variables and FRLRS in the hybrid nomogram, the ROC analysis was performed to estimate the accuracy of 1-year, 3-year, and 5-year over survival (OS).

### 2.3. Molecular Mechanism and Immune Infiltration Enrichment Analyses

Six enrichment analyses algorithms, including the CIBERSORT [32, 33], ESTIMATE, MCPconuter [34], ssGSEA [35], TIMER [36], and xCell algorithms, were compared to evaluate enrichment scores and cellular components in the two risk levels groups according to screen lncRNA signature. Enrichment analyses algorithms were applied to definite enrichment scores representing the gene set absolute enrichment in each sample with the “GSVA” package. Potential immune checkpoints were retrieved from the published literature [37]. Moreover, the TIDE model, trained from treatment-naive tumor data, can predict the likelihood of the immunotherapeutic response [38–40].

### 2.4. Statistical Analysis

LASSO, Cox analysis, and heatmaps were used to assess the correlation between FRLRS and clinicopathological characteristics. The therapeutic response was estimated by the TIDE model and the half-max inhibitory concentration (IC 50) obtained from the GDSC website. Kaplan–Meier survival curves analysis and principal component analysis (PCA) evaluated patients with ccRCC based on the FRLRS signature. Our analyses were performed in the R statistical (4.0.2).

## 3. Results

**Figure 2:**
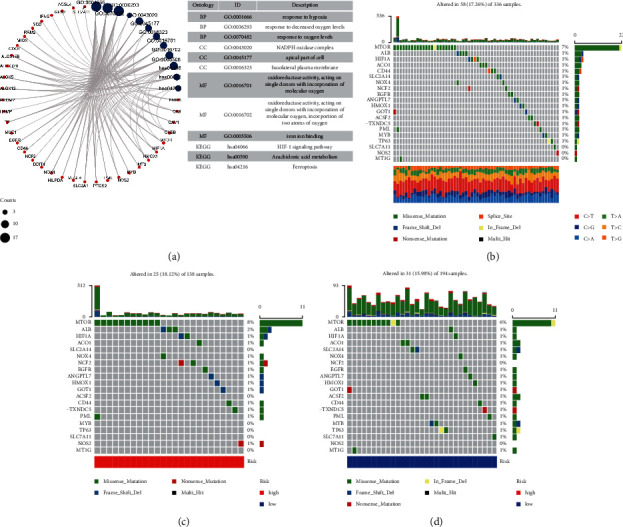
76 ferroptosis-related DEGs in TCGA-KIRC. (a) GO and KEGG enrichment analysis. (b) The mutation information in the entire set. (c) The mutation information in the high-risk group. (d) The mutation information in the low-risk group.

According to TCGA and FerrDb data, 76 ferroptosis-related DEGs were identified (42 upregulated and 34 downregulated genes) (Supplementary Table S2). BP is involved in cell production in response to chemical stress, hypoxia, cofactor metabolism, and oxidative stress. MF mainly regulates iron ion binding, coenzyme binding, and oxidoreductase activity. It acts on a single donor by combining molecular oxygen and nicotinamide adenine dinucleotide phosphate oxidase. CC was mainly elevated in the apical part of the cell, apical plasma membrane, and basolateral plasma membrane. KEGG analysis shows overexpressed genes were mainly involved in HIF-1, microRNA in cancer, Kaposi sarcoma-associated herpesvirus infection, arachidonic acid metabolism, proteoglycan in cancer, human giant cell virus infection, ferroptosis, biosynthesis of amino acids, fluid shear stress and arteriosclerosis, autophagy-animal, and serotonergic synapse (Figure 2(a) and Supplementary Table S3). The waterfall plot displays mutation information of the 76 ferroptosis-related DEGs in TCGA-KIRC entire set (Figure 2(b)) and in two risk groups (Figures 2(c) and 2(d)).

### 3.1. Identification of Prognostic FRLRS Signature in ccRCC

**Figure 3:**
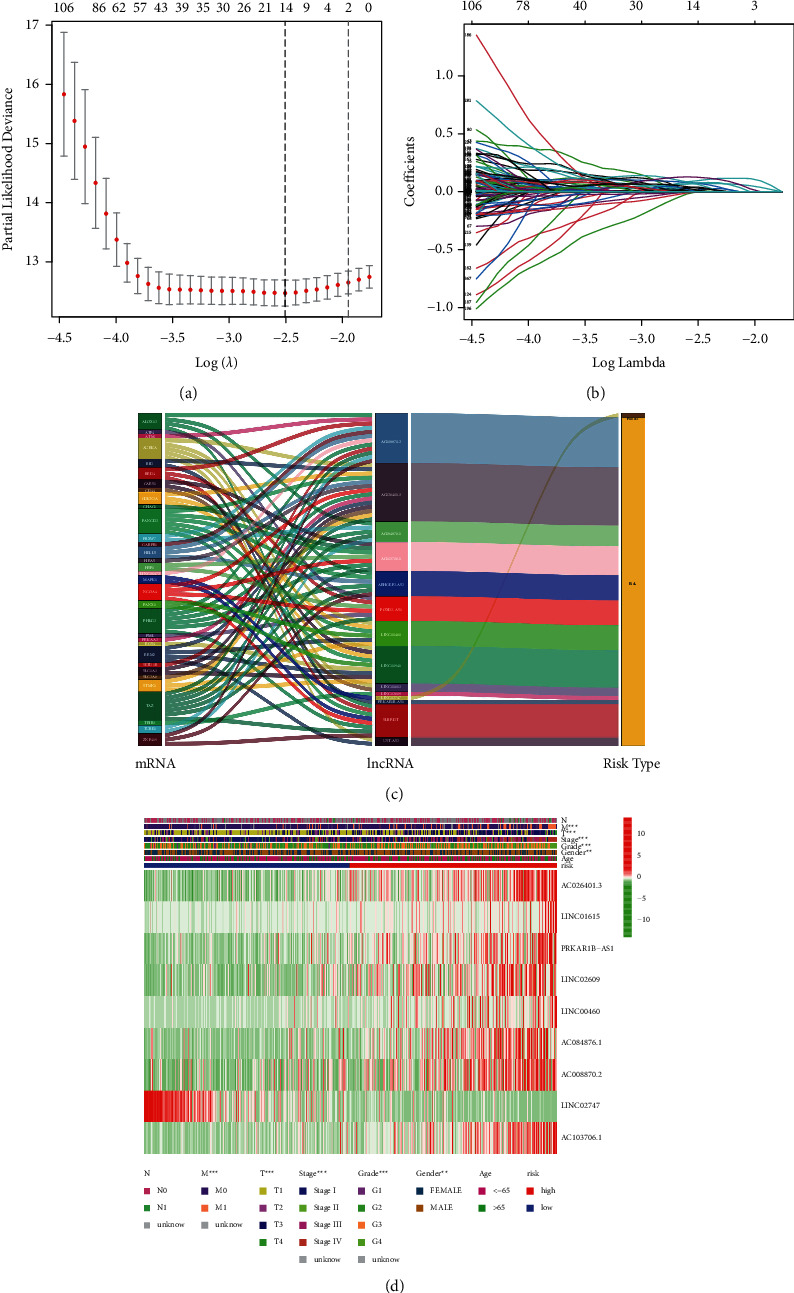
(a) The tuning parameters of LASSO. (b) LASSO coefficient profile of FRLRS. (c) Sankey diagram for ferroptosis-related genes and FRLRS. (d) The heatmap for 9 FRLRS with prognostic feature and clinicopathological variables.

**Figure 4:**
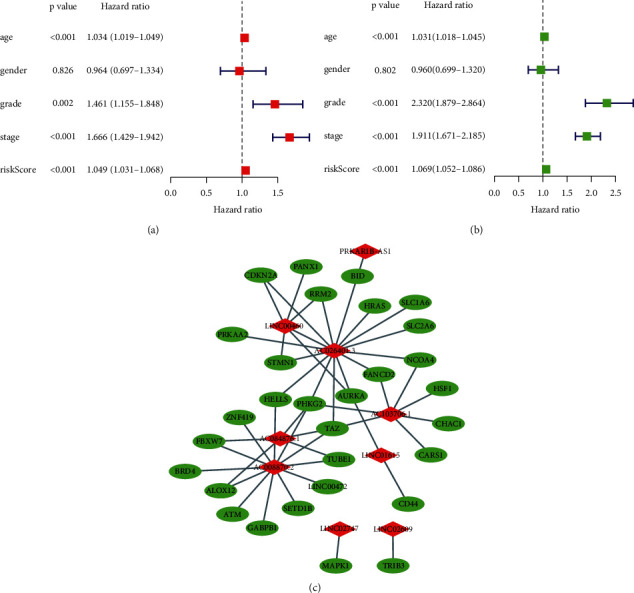
(a) Univariate Cox of FRLRS. (b) Multivariate Cox of FRLRS. (c) The correlations between FRLRS and mRNA.

We obtained 1502 FRLRS in the TCGA-KIRC cohort (Supplementary Table S4). Combined with multivariate Cox regression analysis, univariate Cox analysis identified 106 significant increments associated with FRLRS. Subsequently, 106 lncRNAs were included in the TCGA cohort for LASSO. Overall, 9 differently expressed lncRNAs (AC026401.3, LINC01615, PRKAR1B-AS1, LINC02609, LINC00460, AC084876.1, AC008870.2, LINC02747, and AC103706.1) were uncovered to be independent prognosis predictors in TCGA-KIRC (Figures 3(a) and 3(b), Supplementary Table S5). Sankey relational diagram for ferroptosis-related DEGs and FRLRS is shown in Figure 3(c). The correlation analysis was also examined with a heatmap between the FRLRS feature and clinical variables (Figure 3(d)). Cox analyses revealed that the lncRNA signature (hazard ratio (HR): 1.069, 95% confidence interval (CI): 1.052–1.086), grade (HR: 2.32, CI: 1.879–2.864), age (HR: 1.031, CI: 1.018–1.045), and stage (HR: 1.911, CI: 1.671–2.185) were independent prognostic factors for OS (Figures 4(a) and 4(b)).  Figure 4(c) shows the association between lncRNAs and mRNAs. The correlations heatmap of FRLRS and DEGs are shown in Supplementary Figure S1.

### 3.2. FRLRS Set Analyses and Construction Hybrid Nomogram

**Figure 5:**
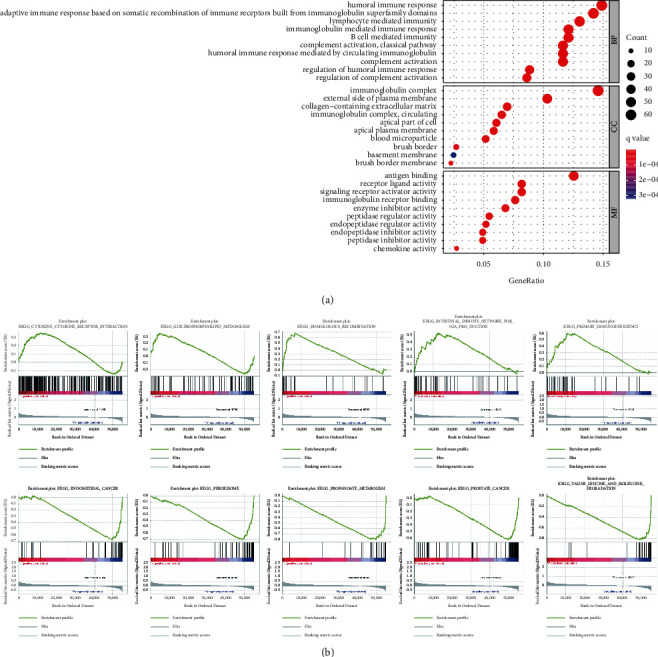
(a) GO analysis for FRLRS based on the TCGA-KIRC dataset. (b) GSEA analysis for FRLRS based on TCGA-KIRC.

**Figure 6:**
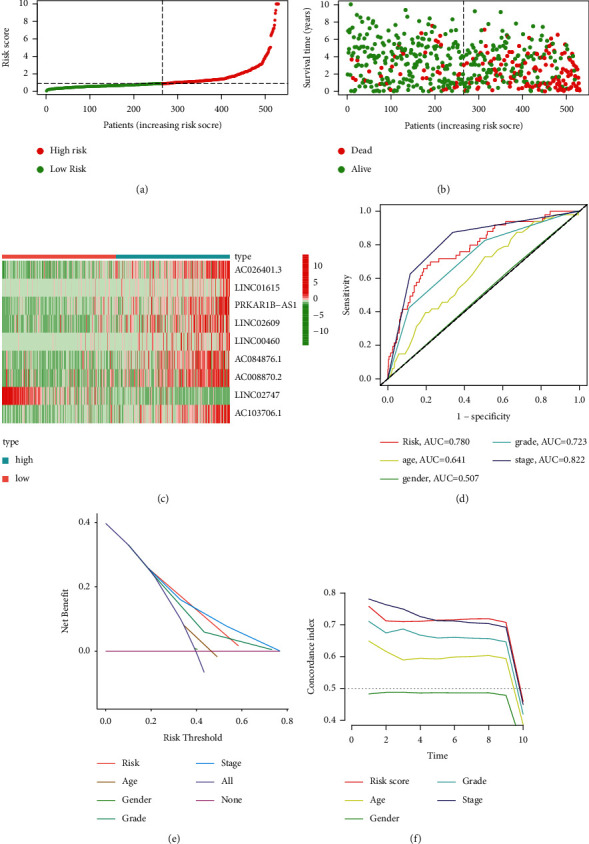
(a) The distinct distribution pattern between two different groups of ccRCC. (b) The survival status and time of ccRCC patients. (c) The relative expression standards of each ccRCC patient. (d) The AUC of the FRLRS signature and traditional clinical variables. (e) The DCA of the risk score and other clinical variables. (f) The c-index of the risk score and other clinical variables.

**Figure 7:**
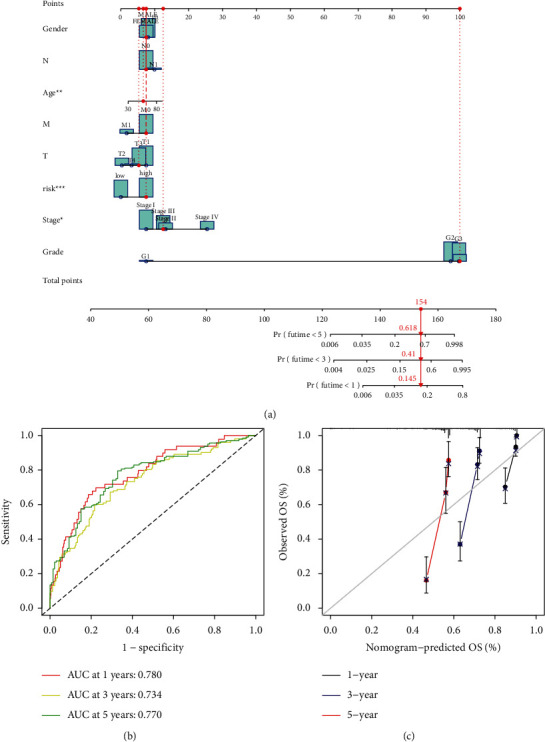
(a) A nomogram using the selected FRLRS prognostic signature and independent factors in TCGA-KIRC. (b) The OS of AUC predictive for 1-year, 3-year, and 5-year. (c) The calibration plot of the nomogram.

GO analysis and GSEA for the biological function of these FRLRS are shown in Figure 5. The novel FRLRS prognostic signature regulated could be found in tumor and immune-related pathways, including cytokine-receptor interaction, glycerophospholipid metabolism, homologous recombination, IgA production, primary immunodeficiency, endometrial cancer, peroxisome, propanoate metabolism, prostate cancer, valine leucine, and isoleucine degradation (Supplementary Table S6). As shown in Figure 6(a), we found a distinct distribution pattern between two different groups of ccRCC patients in regard to prognosis.  Figure 6(b) shows the survival status and time of ccRCC patients in two different risk levels of ccRCC patients.  Figure 6(c) shows the relative expression of 9 FRLRS for each ccRCC patient. Moreover, the prognosis AUC of FRLRS signature was 0.78, outperforming traditional clinical characteristics in predicting ccRCC patients (Figure 6(d)). The DCA of the risk level and other clinicopathological features shows that the prognostic risk model of 9 FRLRS for ccRCC was comparatively dependable (Figure 6(e)). The concordance index (C-index) shows that the risk model performs better than other traditional clinical factors (Figure 6(f)). We established the hybrid nomogram using 9 FRLRS prognostic signatures and independent factors in TCGA-KIRC (Figure 7(a)). The OS of AUC is predictive for 1-year (AUC = 0.78), 3-year (AUC = 0.734), and 5-year (AUC = 0.77) (Figure 7(b)). The calibration plot of the nomogram is shown in Figure 7(c).

### 3.3. Principal Component Analysis (PCA) and Survival Analysis

**Figure 8:**
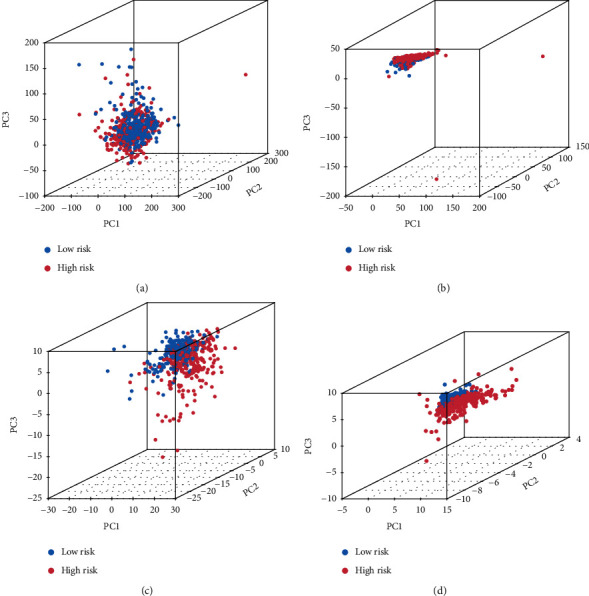
PCA analysis. (a) The entire gene expression in ccRCC patients. (b) 76 ferroptosis genes in different risk groups of ccRCC. (c) 1502 FRLRS in different risk groups of ccRCC patients. (d) 9 FRLRS risk models in different risk groups of ccRCC.

**Figure 9:**
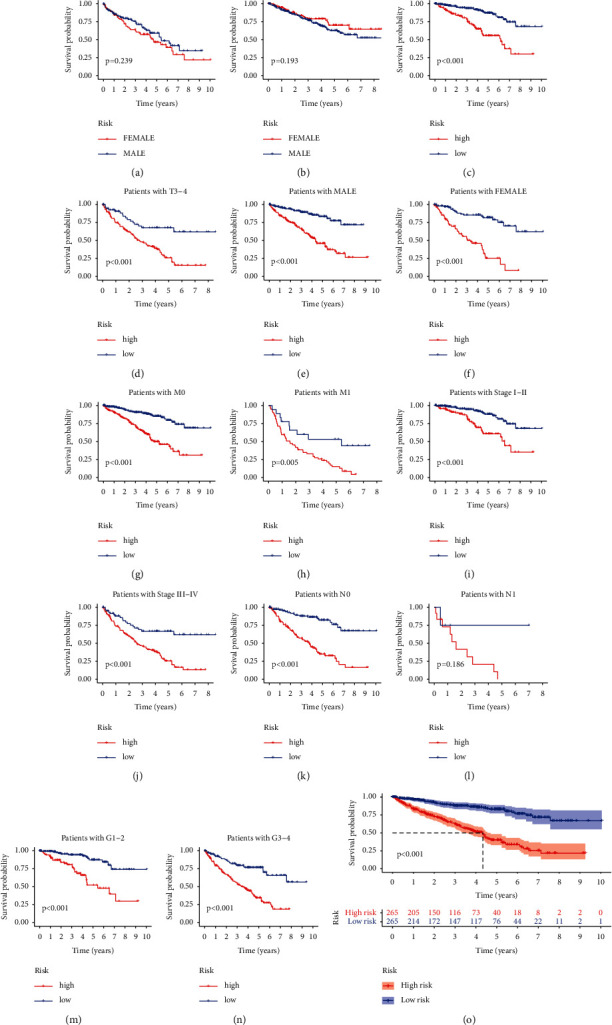
K-M curves of different gender (male/female), ages (age ≥ 65/<65), tumor grade (I-II/III-IV), or TNM stage (T1-2/3-4; M0/1; N0/1) between the different risk groups of ccRCC patients ((a)–(n)). (o) K-M survival curves of ccRCC in the entire set.

The PCA schematic diagram shows two different risk levels of ccRCC patients in entire gene expression (Figure 8(a)), 76 ferroptosis genes (Figure 8(b)), 1502 ferroptosis-related lncRNAs (Figure 8(c)), and 9 lncRNA risk models (Figure 8(d)). In the TCGA-KIRC dataset, the K-M curve showed that OS of low-risk ccRCC group patients stratified by TNM stage, gender, and tumor grade was significantly better (*p* < 0.001, Figures 9(a)–9(o)).

### 3.4. FRLRS Set Enrichment Analyses and Immunity Gene Expression

**Figure 10:**
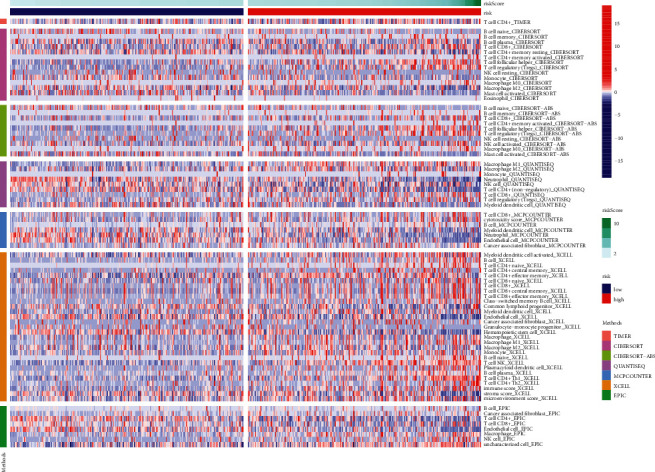
The heatmap of immune responses in two different risk groups of ccRCC patients.

**Figure 11:**
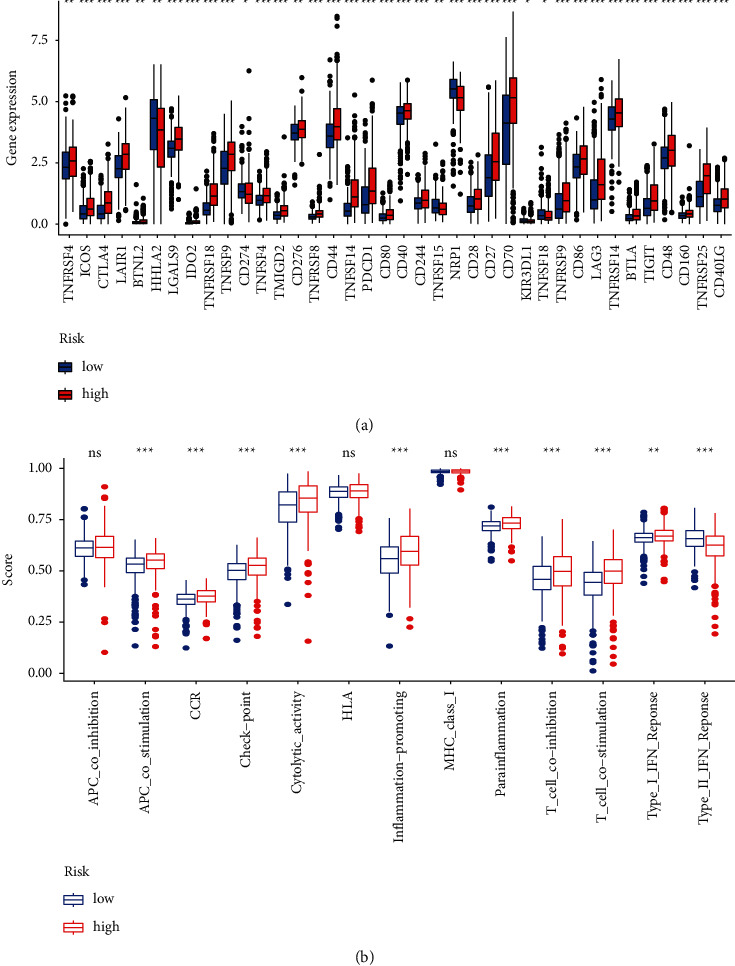
(a) The expression of immune checkpoints between two different risk groups of ccRCC patients. (b) The association between immune cell subpopulations and related functions of ccRCC patients.

Figure 10 shows an immune response heatmap based on different enrichment analyses algorithms, the TIMER, MCPcounter, GSEA, and XCELL algorithms. Because of the importance of immunotherapy based on checkpoint inhibitors, we studied the difference of immune checkpoint genes expression in two different risk groups of ccRCC. Our study indicated significant difference expression genes, such as ICOS, CTLA4, LAIR1, LGALS9, IDO2, TNFRSF18, TNFSF9, TNFSF4, TMIGD2, TNFRSF8, CD44, TNFSF14, PDCD1, CD80, CD40, CD244, NRP1, CD28, CD27, CD70, TNFRSF9, CD86, LAG3, TNFRSF14, BTLA, and TIGIT (Figure 11(a)). Using TCGA-KIRC, data revealed that T cell functions, including APC costimulation and CCR, checkpoint, cytolytic activities, promoting inflammation, parainflammation, and IFN response, were different between both risk groups of ccRCC patients (Figure 11(b)).

### 3.5. Evaluation of the Therapeutic Response with IC50 and TIDE Algorithm

**Figure 12:**
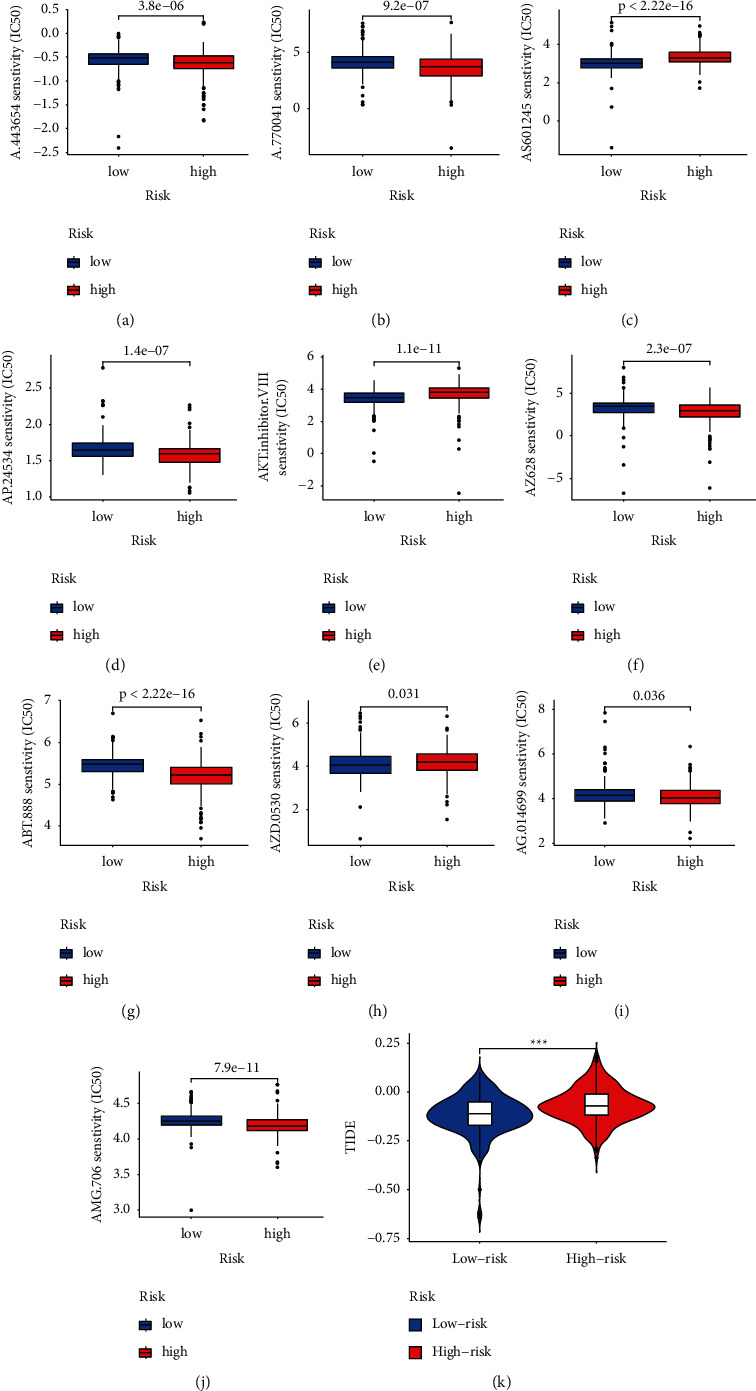
Estimation of the tumor immunotherapy response (IC50) using the FRLRS model in KIRC: (a) drugSenstivity.A.443654; (b) drugSenstivity.A.770041; (c) drugSenstivity.ABT.888; (d) drugSenstivity.AG.014699; (e) drugSenstivity.AKT.inhibitor.VIII; (f) drugSenstivity.AMG.706; (g) drugSenstivity.AP.24534; (h) drugSenstivity.AS601245; (i) drugSenstivity.AZ628; (j) drugSenstivity.AZD.0530; (k) TIDE in two risk groups of ccRCC patients.

Our study used the prophetic algorithm to assess potential drug targeting with the 9 FRLRS model for treating ccRCC. The low-risk group was more sensitive in 10 compounds with significant differences (A.443654, A.770041, ABT.888, AG.014699, AKT.inhibitor.VIII, AMG.706, AP.24534, AS601245, AZ628, and AZD.0530) by the estimated IC50. Figures 12(a)–12(j) show 10 different compounds that may be used to analyze ccRCC patients further. According to the prediction of the TIDE algorithm, the low-risk ccRCC group patient has better immunotherapy response (Figure 12(k)).

## 4. Discussion

We first identified FRLRS signatures using a combined analysis of TCGA-KIRC and FerrDb datasets in this study. This novel approach may lead to new immunotherapeutic targets for tumor treatment. During the development of immunotherapy, we explored whether FRLRS are correlated with immune cells and immunotherapy response in ccRCC prognosis. These findings led to the identification of potential biomarkers or immunotherapy targets in ferroptosis signaling pathways. According to TCGA and FerrDb data, 76 ferroptosis-related DEGs were identified. We obtained 1502 FRLRS in TCGA-KIRC. Combined with LASSO Cox analysis, 106 FRLRS were identified. KEGG analysis revealed that the genes mainly involved in Kaposi sarcoma-associated herpesvirus infection, miRNAs in cancer, arachidonic acid metabolism, proteoglycans in cancer, human cytomegalovirus infection, ferroptosis, biosynthesis of amino acids, fluid shear stress and atherosclerosis, autophagy-animal, and serotonergic synapse. Furthermore, the in-depth analysis revealed 9 differentially expressed lncRNAs (AC026401.3, LINC01615, PRKAR1B-AS1, LINC02609, LINC00460, AC084876.1, AC008870.2, LINC02747, and AC103706.1). PCA shows that the model using FRLRS can distinguish well between ccRCC patients with two different risk levels. The K-M curve shows that ccRCC patients in the low-risk group have a better survival prognosis. These FRLRS may be independent prognostic biomarkers for ccRCC patients.

Among these lncRNAs, LINC01615 was identified as a metastasis-related lncRNA in HCC [41]. LINC00460 was also found to be critical for multiple tumorigeneses. A recent study found that LINC00460 could promote epithelial-mesenchymal transition in HNSCC by facilitating peroxiredoxin-1 [42]. A related study suggested that LINC00460 is a potential biomarker related to outcomes in malignant tumors [43], which provides critical insights into the targeting of FRLRS in predicting ccRCC. Recently, high LINC02747 expression was significantly associated with advanced tumor TNM stage, histological grade, and poor outcome, thus promoting the proliferation of ccRCC according to inhibit miR-608 [44]. Collectively, these findings provide an essential basis for this study regarding the association between lncRNAs and ccRCC. Nevertheless, our findings may provide a research direction on the role of FRLRS in ccRCC prognosis for cancer treatment.

The ssGSEA algorithm revealed that T cell functions, such as APC costimulation and CCR checkpoint, and cytolytic activities, promoting inflammation and parainflammation, were significantly different in different risk groups of ccRCC. Combination ferroptosis with ICIs can synergistically promote antitumor activity, even in ICI resistance [45]. Because of the importance of immunotherapy based on checkpoint inhibitors, our data revealed a substantial difference expression with immune checkpoint-related genes in both groups of ccRCC patients, highlighting the potential significance of FRLRS in regulating ICIs. The total number of somatic encoding mutation (TMB) is a potent biomarker for predicting the response to immunotherapy. As in other studies [40], TMB exceeded the high-risk ccRCC group. Furthermore, several studies have validated that TIDE algorithms can serve as a predictive model for immunotherapy [38–40]. In this study, ccRCC patients in the low-risk group have better immunotherapy response. Ten candidate compounds for KIRC differentiation were identified in our study.

Based on the characteristics of these 9 lncRNAs, we suggest that it could assist in developing diagnostic and prognostic kits for ccRCC and offer potential comprehensive targets for the future intervention of ccRCC. We agree that in future, more prominent translational and clinical studies confirm this signature observed in our study setting. The findings of our study provide new insight into the underlying mechanisms by FRLRS that can predict the prognosis of ccRCC. Nevertheless, limitations still exist, and future studies are warranted for further investigation. We only use samples from the TCGA cohort to build the model. Further validation using different cohorts is needed to verify the signature profiles in this setting. Second, we use the retrospective data of the public database to construct and verify FRLRS. Information from prospective data to evaluate its clinical efficacy for ccRCC is limited, and its molecular mechanism has not been determined—more basic experiments need further to validate the interaction between lncRNA and ferroptosis genes. Thus, considering that clinical samples do not verify the results of this study, there is no guarantee that reliability can be directly tested.

## 5. Conclusion

In summary, we elucidated that specific FRLRS in the prognostic prediction of ccRCC. Furthermore, our current findings may provide more valuable insights for future ccRCC research by much more large clinical trials.

## Data Availability

The datasets used to support the findings of this study are publicly available in the TIDE database, FerrDb, and TCGA database (https://portal.gdc.cancer.gov/).

## Supplementary Materials

Supplementary Materials Table S1: 259 ferroptosis-related genes in FerrDb. Table S2: 76 ferroptosis-related genes in TCGA-KIRC. Table S3: GO and KEGG analysis in DEGs. Table S4: 1502 FRLRS in TCGA-KIRC cohort. Table S5: univariate and multivariate Cox analyses. Table S6: FRLRS set enrichment analyses and immunity gene expression. Figure S1. The heatmap of the correlations between ferroptosis-related lncRNA and genes.
