# Supplementary material for: A Novel Prognostic Ferroptosis-Related Long Noncoding RNA Signature in Clear Cell Renal Cell Carcinoma
Source: J Oncol. 2022 Feb 22;2022:6304824. doi: 10.1155/2022/6304824 (PMC8888116; doi:10.1155/2022/6304824)
Supplement: Supplementary Materials — Table S1: 259 ferroptosis-related genes in FerrDb. Table S2: 76 ferroptosis-related genes in TCGA-KIRC. Table S3: GO and KEGG analysis in DEGs. Table S4: 1502 FRLRS in TCGA-KIRC cohort. Table S5: univariate and multivariate Cox analyses. Table S6: FRLRS set enrichment analyses and immunity gene expression. Figure S1. The heatmap of the correlations between ferroptosis-related lncRNA and genes. [file 6304824.f1.zip › 6304824.f1/ID 6304824 Supplementary Table S1-6.pdf]

Table S1 259 ferroptosis-related genes in FerrDb

Driver

Symbol	Name	HGNC_ID
RPL8	Ribosomal protein L8	HGNC:10368
IREB2	Iron response element binding protein 2	HGNC:6115
ATP5MC3	ATP synthase membrane subunit c locus 3	HGNC:843
CS	Citrate synthase	HGNC:2422
EMC2	ER membrane protein complex subunit 2	HGNC:28963
ACSF2	Acyl-CoA synthetase family member 2	HGNC:26101
NOX1	Nicotinamide adenine dinucleotide phosphate (NADPH) oxidase (NOX) 1	HGNC:7889
CYBB	Cytochrome b-245 beta chain	HGNC:2578
NOX3	Nicotinamide adenine dinucleotide phosphate (NADPH) oxidase (NOX) 3	HGNC:7890
NOX4	Nicotinamide adenine dinucleotide phosphate (NADPH) oxidase (NOX) 4	HGNC:7891
NOX5	Nicotinamide adenine dinucleotide phosphate (NADPH) oxidase (NOX) 5	HGNC:14874
DUOX1	Dual oxidase 1	HGNC:3062
DUOX2	Dual oxidase 2	HGNC:13273
G6PD	Glucose-6-phosphate dehydrogenase	HGNC:4057
PGD	Phosphoglycerate dehydrogenase	HGNC:8891
VDAC2	Valtage-dependent anion channels 2	HGNC:12672
PIK3CA	Phosphatidylinositol-4,5-bisphosphate 3-kinase catalytic subunit alpha	HGNC:8975
FLT3	Fms related tyrosine kinase 3	HGNC:3765
SCP2	Sterol carrier protein 2	HGNC:10606
TP53	Tumor protein p53	HGNC:11998
ACSL4	Acyl-CoA synthetase long chain family member 4	HGNC:3571
LPCAT3	Lysophosphatidylcholine acyltransferase 3	HGNC:30244
NRAS	NRAS proto-oncogene, GTPase	HGNC:7989
KRAS	KRAS proto-oncogene, GTPase	HGNC:6407
HRAS	HRas proto-oncogene, GTPase	HGNC:5173
TF	Transferrin	HGNC:11740
TFRC	Transferrin receptor	HGNC:11763
TFR2	Transferrin receptor 2	HGNC:11762
SLC38A1	Solute carrier family 38 member 1	HGNC:13447
SLC1A5	Solute carrier family 1 member 5	HGNC:10943
GLS2	Glutaminase 2	HGNC:29570
GOT1	Glutamic-oxaloacetic transaminase 1	HGNC:4432
CARS1	Cysteinyl-tRNA synthetase 1	HGNC:1493
TP53	Tumor protein p53	HGNC:11998
ALOX5	Arachidonate 5-lipoxygenase	HGNC:435

KEAP1	Kelch like ECH associated protein 1	HGNC:23177
HMOX1	Heme oxygenase 1	HGNC:5013
TP53	Tumor protein p53	HGNC:11998
TP53	Tumor protein p53	HGNC:11998
GLS2	Glutaminase 2	HGNC:29570
ATG5	Autophagy related 5	HGNC:589
ATG7	Autophagy related 7	HGNC:16935
NCOA4	Nuclear receptor coactivator 4	HGNC:7671
TF	Transferrin	HGNC:11740
ALOX5	Arachidonate 5-lipoxygenase	HGNC:435
ALOX12	Arachidonate 12-lipoxygenase, 12S type	HGNC:429
ALOX12B	Arachidonate 12-lipoxygenase, 12R type	HGNC:430
ALOX15	Arachidonate 15-lipoxygenase	HGNC:433
ALOX15B	Arachidonate 15-lipoxygenase type B	HGNC:434
ALOXE3	Arachidonate lipoxygenase 3	HGNC:13743
PHKG2	Phosphorylase kinase catalytic subunit gamma 2	HGNC:8931
TFRC	Transferrin receptor	HGNC:11763
ACO1	Aconitase 1	HGNC:117
IREB2	iron responsive element binding protein 2	HGNC:6115
SLC38A1	Solute carrier family 38 member 1	HGNC:13447
GLS2	Glutaminase 2	HGNC:29570
G6PDX	_NA_	_NA_
ULK1	Unc-51 like autophagy activating kinase 1	HGNC:12558
ATG3	Autophagy related 3	HGNC:20962
ATG4D	Autophagy related 4D cysteine peptidase	HGNC:20789
ATG5	Autophagy related 5	HGNC:589
BECN1	Beclin 1	HGNC:1034
MAP1LC3A	Microtubule associated protein 1 light chain 3 alpha	HGNC:6838
GABARAPL	GABA type A receptor associated protein like 2	HGNC:13291
GABARAPL	GABA type A receptor associated protein like 1	HGNC:4068
ATG16L1	Autophagy related 16 like 1	HGNC:21498
WIPI1	WD repeat domain, phosphoinositide interacting 1	HGNC:25471
WIPI2	WD repeat domain, phosphoinositide interacting 2	HGNC:32225
SNX4	Sorting nexin 4	HGNC:11175
ATG13	Autophagy related 13	HGNC:29091
ULK2	Unc-51 like autophagy activating kinase 2	HGNC:13480
NCOA4	Nuclear receptor coactivator 4	HGNC:7671
ACSL4	Acyl-CoA synthetetase long chain family member 4	HGNC:3571

TP53	Tumor protein p53	HGNC:11998
SAT1	Spermidine/spermine N1-acetyltransferase 1	HGNC:10540
ALOX15	Arachidonate 15-lipoxygenase	HGNC:433
ACSL4	Acyl-CoA synthetase long chain family member 4	HGNC:3571
LPCAT3	Lysophosphatidylcholine acyltransferase 3	HGNC:30244
ALOX15	Arachidonate 15-lipoxygenase	HGNC:433
ACSL4	Acyl-CoA synthetase long chain family member 4	HGNC:3571
KEAP1	Kelch like ECH associated protein 1	HGNC:23177
EGFR	Epidermal growth factor receptor	HGNC:3236
NOX4	NADPH oxidase 4	HGNC:7891
MAPK3	Mitogen-activated protein kinase 3	HGNC:6877
MAPK1	Mitogen-activated protein kinase 1	HGNC:6871
BID	BH3 interacting domain death agonist	HGNC:1050
ACSL4	Acyl-CoA synthetase long chain family member 4	HGNC:3571
ZEB1	Zinc finger E-box binding homeobox 1	HGNC:11642
KEAP1	Kelch like ECH associated protein 1	HGNC:23177
DPP4	Dipeptidyl peptidase 4	HGNC:3009
ALOX15	Arachidonate 15-lipoxygenase	HGNC:433
ALOX12	Arachidonate 12-lipoxygenase, 12S type	HGNC:429
CDKN2A	Cyclin dependent kinase inhibitor 2A	HGNC:1787
PEBP1	Phosphatidylethanolamine binding protein 1	HGNC:8630
SOCS1	Suppressor of cytokine signaling 1	HGNC:19383
CDO1	Cysteine dioxygenase type 1	HGNC:1795
MYB	MYB proto-oncogene, transcription factor	HGNC:7545
HMOX1	Heme oxygenase 1	HGNC:5013
MAPK8	Mitogen-activated protein kinase 8	HGNC:6881
MAPK9	Mitogen-activated protein kinase 9	HGNC:6886
MAPK1	Mitogen-activated protein kinase 1	HGNC:6871
MAPK3	Mitogen-activated protein kinase 3	HGNC:6877
SLC1A5	Solute carrier family 1 member 5	HGNC:10943
CHAC1	ChaC glutathione specific gamma-glutamylcyclotransferase 1	HGNC:28680
MAPK14	Mitogen-activated protein kinase 14	HGNC:6876
LINC00472	Long intergenic non-protein coding RNA 472	HGNC:21380
NOX4	NADPH oxidase 4	HGNC:7891
GOT1	Glutamic-oxaloacetic transaminase 1	HGNC:4432
BECN1	Beclin 1	HGNC:1034
PRKAA2	Protein kinase AMP-activated catalytic subunit alpha 2	HGNC:9377
PRKAA1	Protein kinase AMP-activated catalytic subunit alpha 1	HGNC:9376

ELAVL1	ELAV like RNA binding protein 1	HGNC:3312
BAP1	BRCA1 associated protein 1	HGNC:950
TP53	Tumor protein p53	HGNC:11998
ABCC1	ATP binding cassette subfamily C member 1	HGNC:51
ACSL4	Acyl-CoA synthetase long chain family member 4	HGNC:3571
MIR6852	microRNA 6852	HGNC:49993
ACVR1B	Activin A receptor type 1B	HGNC:172
TGFBR1	Transforming growth factor beta receptor 1	HGNC:11772
BAP1	BRCA1 associated protein 1	HGNC:950
EPAS1	Endothelial PAS domain protein 1	HGNC:3374
HILPDA	Hypoxia inducible lipid droplet associated	HGNC:28859
HIF1A	Hypoxia inducible factor 1 subunit alpha	HGNC:4910
ALOX12	Arachidonate 12-lipoxygenase, 12S type	HGNC:429
ACSL4	Acyl-CoA synthetase long chain family member 4	HGNC:3571
HMOX1	Heme oxygenase 1	HGNC:5013
IFNG	Interferon gamma	HGNC:5438
ANO6	Anoctamin 6	HGNC:25240
LPIN1	Lipin 1	HGNC:13345
HMGB1	High mobility group box 1	HGNC:4983
TNFAIP3	TNF alpha induced protein 3	HGNC:11896
TLR4	Toll like receptor 4	HGNC:11850
NOX4	NADPH oxidase 4	HGNC:7891
ATF3	Activating transcription factor 3	HGNC:785
ATM	ATM serine/threonine kinase	HGNC:795
YY1AP1	YY1 associated protein 1	HGNC:30935
EGLN2	Egl-9 family hypoxia inducible factor 2	HGNC:14660
MIOX	Myo-inositol oxygenase	HGNC:14522
TAZ	Tafazzin	HGNC:11577
MTDH	Metadherin	HGNC:29608
IDH1	Isocitrate dehydrogenase (NADP(+)) 1	HGNC:5382
SIRT1	Sirtuin 1	HGNC:14929
TAZ	Tafazzin	HGNC:11577
BECN1	Beclin 1	HGNC:1034
FBXW7	F-box and WD repeat domain containing 7	HGNC:16712
PANX1	Pannexin 1	HGNC:8599
DNAJB6	DnaJ heat shock protein family (Hsp40) member B6	HGNC:14888
BACH1	BTB domain and CNC homolog 1	HGNC:935
ACSL4	Acyl-CoA synthetase long chain family member 4	HGNC:3571

LONP1	Lon peptidase 1, mitochondrial	HGNC:9479

Suppressor

Symbol	Name	HGNC_ID
SLC7A11	Solute carrier family 7 member 11	HGNC:11059
GPX4	Glutathione peroxidase 4	HGNC:4556
AKR1C1	Aldo-keto reductase family 1 member C1	HGNC:384
AKR1C2	Aldo-keto reductase family 1 member C2	HGNC:385
AKR1C3	Aldo-keto reductase family 1 member C3	HGNC:386
GPX4	Glutathione peroxidase 4	HGNC:4556
RB1	RB transcriptional corepressor 1	HGNC:9884
HSPB1	Heat shock protein family B (small) member 1	HGNC:5246
HSF1	Heat shock transcription factor 1	HGNC:5224
SLC7A11	Solute carrier family 7 member 11	HGNC:11059
GPX4	Glutathione peroxidase 4	HGNC:4556
GCLC	Glutamate-cysteine ligase catalytic subunit	HGNC:4311
SLC7A11	Solute carrier family 7 member 11	HGNC:11059
NFE2L2	Nuclear factor, erythroid 2 like 2	HGNC:7782
SQSTM1	Sequestosome 1	HGNC:11280
NQO1	NAD(P)H quinone dehydrogenase 1	HGNC:2874
HMOX1	Heme oxygenase 1	HGNC:5013
FTH1	Ferritin heavy chain 1	HGNC:3976
MUC1	Mucin 1, cell surface associated	HGNC:7508
SLC3A2	Solute carrier family 3 member 2	HGNC:11026
MT1G	Metallothionein 1G	HGNC:7399
NFE2L2	Nuclear factor, erythroid 2 like 2	HGNC:7782
SLC40A1	Solute carrier family 40 member 1	HGNC:10909
SLC7A11	Solute carrier family 7 member 11	HGNC:11059
GPX4	Glutathione peroxidase 4	HGNC:4556
SLC7A11	Solute carrier family 7 member 11	HGNC:11059
CISD1	CDGSH iron sulfur domain 1	HGNC:30880
SLC7A11	Solute carrier family 7 member 11	HGNC:11059
FANCD2	FA complementation group D2	HGNC:3585
GPX4	Glutathione peroxidase 4	HGNC:4556
NFE2L2	Nuclear factor, erythroid 2 like 2	HGNC:7782
FTMT	Ferritin mitochondrial	HGNC:17345
HSPA5	Heat shock protein family A (Hsp70) member 5	HGNC:5238
ATF4	Activating transcription factor 4	HGNC:786
SLC7A11	Solute carrier family 7 member 11	HGNC:11059

GPX4	Glutathione peroxidase 4	HGNC:4556
GPX4	Glutathione peroxidase 4	HGNC:4556
HMOX1	Heme oxygenase 1	HGNC:5013
ATF4	Activating transcription factor 4	HGNC:786
NFE2L2	Nuclear factor, erythroid 2 like 2	HGNC:7782
TP53	Tumor protein p53	HGNC:11998
SLC7A11	Solute carrier family 7 member 11	HGNC:11059
HELLS	Helicase, lymphoid specific	HGNC:4861
SCD	Stearoyl-CoA desaturase	HGNC:10571
FADS2	Fatty acid desaturase 2	HGNC:3575
SRC	SRC proto-oncogene, non-receptor tyrosine kinase	HGNC:11283
STAT3	Signal transducer and activator of transcription 3	HGNC:11364
NFE2L2	Nuclear factor, erythroid 2 like 2	HGNC:7782
PML	Promyelocytic leukemia	HGNC:9113
MTOR	Mechanistic target of rapamycin kinase	HGNC:3942
NFS1	NFS1 cysteine desulfurase	HGNC:15910
TP63	Tumor protein p63	HGNC:15979
SLC7A11	Solute carrier family 7 member 11	HGNC:11059
TP53	Tumor protein p53	HGNC:11998
CDKN1A	Cyclin dependent kinase inhibitor 1A	HGNC:1784
MIR137	microRNA 137	HGNC:31523
SLC40A1	Solute carrier family 40 member 1	HGNC:10909
GPX4	Glutathione peroxidase 4	HGNC:4556
GPX4	Glutathione peroxidase 4	HGNC:4556
ENPP2	Ectonucleotide pyrophosphatase/phosphodiesterase 2	HGNC:3357
VDAC2	Voltage dependent anion channel 2	HGNC:12672
FH	Fumarate hydratase	HGNC:3700
CISD2	CDGSH iron sulfur domain 2	HGNC:24212
SLC40A1	Solute carrier family 40 member 1	HGNC:10909
MIR9-1	microRNA 9-1	HGNC:31641
MIR9-2	microRNA 9-2	HGNC:31642
MIR9-3	microRNA 9-3	HGNC:31646
CBS	Cystathionine beta-synthase	HGNC:1550
NFE2L2	Nuclear factor, erythroid 2 like 2	HGNC:7782
SQSTM1	Sequestosome 1	HGNC:11280
GPX4	Glutathione peroxidase 4	HGNC:4556
ISCU	Iron-sulfur cluster assembly enzyme	HGNC:29882
FTH1	Ferritin heavy chain 1	HGNC:3976

ACSL3	Acyl-CoA synthetase long chain family member 3	HGNC:3570
OTUB1	OTU deubiquitinase, ubiquitin aldehyde binding 1	HGNC:23077
CD44	CD44 molecule (Indian blood group)	HGNC:1681
LINC00336	Long intergenic non-protein coding RNA 336	HGNC:33813
STAT3	Signal transducer and activator of transcription 3	HGNC:11364
BRD4	Bromodomain containing 4	HGNC:13575
PRDX6	Peroxiredoxin 6	HGNC:16753
MIR17	microRNA 17	HGNC:31547
SCD	Stearoyl-CoA desaturase	HGNC:10571
SESN2	Sestrin 2	HGNC:20746
NF2	Neurofibromin 2	HGNC:7773
ARNTL	Aryl hydrocarbon receptor nuclear translocator like	HGNC:701
HIF1A	Hypoxia inducible factor 1 subunit alpha	HGNC:4910
JUN	Jun proto-oncogene, AP-1 transcription factor subunit	HGNC:6204
CA9	Carbonic anhydrase 9	HGNC:1383
HSPA5	Heat shock protein family A (Hsp70) member 5	HGNC:5238
TMBIM4	Transmembrane BAX inhibitor motif containing 4	HGNC:24257
HSPA5	Heat shock protein family A (Hsp70) member 5	HGNC:5238
PLIN2	Perilipin 2	HGNC:248
MIR212	microRNA 212	HGNC:31589
Fer1HCH	Ferritin 1 Heavy Chain Homolog	_NA_
AIFM2	Apoptosis inducing factor mitochondria associated 2	HGNC:21411
AIFM2	Apoptosis inducing factor mitochondria associated 2	HGNC:21411
LAMP2	Lysosomal associated membrane protein 2	HGNC:6501
ZFP36	ZFP36 ring finger protein	HGNC:12862
GPX4	Glutathione peroxidase 4	HGNC:4556
PROM2	Prominin 2	HGNC:20685
CHMP5	Charged multivesicular body protein 5	HGNC:26942
CHMP6	Charged multivesicular body protein 6	HGNC:25675
AKR1C1	Aldo-keto reductase family 1 member C1	HGNC:384
AKR1C2	Aldo-keto reductase family 1 member C2	HGNC:385
AKR1C3	Aldo-keto reductase family 1 member C3	HGNC:386
CBS	Cystathionine beta-synthase	HGNC:1550
NFE2L2	Nuclear factor, erythroid 2 like 2	HGNC:7782
CAV1	Caveolin 1	HGNC:1527
GCH1	GTP cyclohydrolase 1	HGNC:4193

Marker

Symbol	Name	HGNC_ID

PTGS2	Prostaglandin-endoperoxide synthase 2	HGNC:9605
DUSP1	Dual specificity phosphatase 1	HGNC:3064
NOS2	Nitric oxide synthase 2	HGNC:7873
NCF2	Neutrophil cytosolic factor 2	HGNC:7661
MT3	Metallothionein 3	HGNC:7408
UBC	Ubiquitin C	HGNC:12468
ALB	Albumin	HGNC:399
TXNRD1	Thioredoxin reductase 1	HGNC:12437
SRXN1	Sulfiredoxin 1	HGNC:16132
GPX2	Glutathione peroxidase 2	HGNC:4554
BNIP3	BCL2 interacting protein 3	HGNC:1084
OXSR1	Oxidative stress responsive kinase 1	HGNC:8508
SELENOS	Selenoprotein S	HGNC:30396
ANGPTL7	Angiopoietin like 7	HGNC:24078
CHAC1	ChaC glutathione specific gamma-glutamylcyclotransferase 1	HGNC:28680
SLC7A11	Solute carrier family 7 member 11	HGNC:11059
DDIT4	DNA damage inducible transcript 4	HGNC:24944
LOC28456	_NA_	_NA_
ASNS	Asparagine synthetase (glutamine-hydrolyzing)	HGNC:753
TSC22D3	TSC22 domain family member 3	HGNC:3051
DDIT3	DNA damage inducible transcript 3	HGNC:2726
JDP2	Jun dimerization protein 2	HGNC:17546
SESN2	Sestrin 2	HGNC:20746
SLC1A4	Solute carrier family 1 member 4	HGNC:10942
PCK2	Phosphoenolpyruvate carboxykinase 2, mitochondrial	HGNC:8725
TXNIP	Thioredoxin interacting protein	HGNC:16952
VLDLR	Very low density lipoprotein receptor	HGNC:12698
GPT2	Glutamic--pyruvic transaminase 2	HGNC:18062
PSAT1	Phosphoserine aminotransferase 1	HGNC:19129
LURAP1L	Leucine rich adaptor protein 1 like	HGNC:31452
SLC7A5	Solute carrier family 7 member 5	HGNC:11063
HERPUD1	Homocysteine inducible ER protein with ubiquitin like domain 1	HGNC:13744
XBP1	X-box binding protein 1	HGNC:12801
ATF3	Activating transcription factor 3	HGNC:785
SLC3A2	Solute carrier family 3 member 2	HGNC:11026
CBS	Cystathionine beta-synthase	HGNC:1550
ATF4	Activating transcription factor 4	HGNC:786
ZNF419	Zinc finger protein 419	HGNC:20648

KLHL24	Kelch like family member 24	HGNC:25947
TRIB3	Tribbles pseudokinase 3	HGNC:16228
ZFP69B	ZFP69 zinc finger protein B	HGNC:28053
ATP6V1G2	ATPase H+ transporting V1 subunit G2	HGNC:862
VEGFA	Vascular endothelial growth factor A	HGNC:12680
GDF15	Growth differentiation factor 15	HGNC:30142
TUBE1	Tubulin epsilon 1	HGNC:20775
ARRDC3	Arrestin domain containing 3	HGNC:29263
CEBPG	CCAAT enhancer binding protein gamma	HGNC:1837
SNORA16/	Small nucleolar RNA, H/ACA box 16A	HGNC:32605
RGS4	Regulator of G protein signaling 4	HGNC:10000
BLOC1S5-1	BLOC1S5-TXNDC5 readthrough (NMD candidate)	HGNC:42001
LOC39070!	_NA_	_NA_
EIF2S1	Eukaryotic translation initiation factor 2 subunit 1	HGNC:3265
KIM-1	Kidney injury molecule-1	_NA_
IL6	Interleukin 6	HGNC:6018
CXCL2	C-X-C motif chemokine ligand 2	HGNC:4603
RELA	RELA proto-oncogene, NF-kB subunit	HGNC:9955
HSD17B11	Hydroxysteroid 17-beta dehydrogenase 11	HGNC:22960
AGPAT3	1-acylglycerol-3-phosphate O-acyltransferase 3	HGNC:326
SETD1B	SET domain containing 1B, histone lysine methyltransferase	HGNC:29187
HMOX1	Heme oxygenase 1	HGNC:5013
TF	Transferrin	HGNC:11740
FTL	Ferritin light chain	HGNC:3999
RPL8	Ribosomal protein L8	HGNC:10368
ATP5MC3	ATP synthase membrane subunit c locus 3	HGNC:843
TFRC	Transferrin receptor	HGNC:11763
MAFG	MAF bZIP transcription factor G	HGNC:6781
IL33	Interleukin 33	HGNC:16028
FTH1	Ferritin heavy chain 1	HGNC:3976
SLC40A1	Solute carrier family 40 member 1	HGNC:10909
TF	Transferrin	HGNC:11740
TFRC	Transferrin receptor	HGNC:11763
FTH1	Ferritin heavy chain 1	HGNC:3976
GPX4	Glutathione peroxidase 4	HGNC:4556
HAMP	Hepcidin antimicrobial peptide	HGNC:15598
HSPB1	Heat shock protein family B (small) member 1	HGNC:5246
NFE2L2	Nuclear factor, erythroid 2 like 2	HGNC:7782

STEAP3	STEAP3 metalloreductase	HGNC:24592
DRD5	Dopamine receptor D5	HGNC:3026
GPX4	Glutathione peroxidase 4	HGNC:4556
DRD4	Dopamine receptor D4	HGNC:3025
MAP3K5	Mitogen-activated protein kinase kinase kinase 5	HGNC:6857
MAPK14	Mitogen-activated protein kinase 14	HGNC:6876
SLC2A1	Solute carrier family 2 member 1	HGNC:11005
SLC2A3	Solute carrier family 2 member 3	HGNC:11007
SLC2A6	Solute carrier family 2 member 6	HGNC:11011
SLC2A8	Solute carrier family 2 member 8	HGNC:13812
SLC2A12	Solute carrier family 2 member 12	HGNC:18067
GLUT13	_NA_	_NA_
SLC2A14	Solute carrier family 2 member 14	HGNC:18301
EIF2AK4	Eukaryotic translation initiation factor 2 alpha kinase 4	HGNC:19687
EIF2S1	Eukaryotic translation initiation factor 2 subunit alpha	HGNC:3265
ATF4	Activating transcription factor 4	HGNC:786
ALOX5	Arachidonate 5-lipoxygenase	HGNC:435
ALOX12	Arachidonate 12-lipoxygenase, 12S type	HGNC:429
ALOX15	Arachidonate 15-lipoxygenase	HGNC:433
ALOX5	Arachidonate 5-lipoxygenase	HGNC:435
ACSF2	Acyl-CoA synthetase family member 2	HGNC:26101
IREB2	Iron responsive element binding protein 2	HGNC:6115
GPX4	Glutathione peroxidase 4	HGNC:4556
HMGB1	High mobility group box 1	HGNC:4983
HMOX1	Heme oxygenase 1	HGNC:5013
NFE2L2	Nuclear factor, erythroid 2 like 2	HGNC:7782
ELAVL1	ELAV like RNA binding protein 1	HGNC:3312
SLC3A2	Solute carrier family 3 member 2	HGNC:11026
SLC7A11	Solute carrier family 7 member 11	HGNC:11059
TFAP2C	Transcription factor AP-2 gamma	HGNC:11744
SP1	Sp1 transcription factor	HGNC:11205
HBA1	Hemoglobin subunit alpha 1	HGNC:4823
NNMT	Nicotinamide N-methyltransferase	HGNC:7861
PLIN4	Perilipin 4	HGNC:29393
HIC1	HIC ZBTB transcriptional repressor 1	HGNC:4909
STMN1	Stathmin 1	HGNC:6510
RRM2	Ribonucleotide reductase regulatory subunit M2	HGNC:10452
CAPG	Capping actin protein, gelsolin like	HGNC:1474

HNF4A	Hepatocyte nuclear factor 4 alpha	HGNC:5024
NGB	Neuroglobin	HGNC:14077
YWHAE	Tyrosine 3-monooxygenase/tryptophan 5-monooxygenase activation pro	HGNC:12851
GABPB1	GA binding protein transcription factor subunit beta 1	HGNC:4074
AURKA	Aurora kinase A	HGNC:11393
MIR4715	microRNA 4715	HGNC:41666
RIPK1	Receptor interacting serine/threonine kinase 1	HGNC:10019
PRDX1	Peroxiredoxin 1	HGNC:9352
MIR30B	microRNA 30b	HGNC:31625

Table S2 76 Ferroptosis-related genes in TCGA-KIRC

gene	conMean	treatMean	logFC	pValue	fdr
SLC7A11	0.114650648	0.388249872	1.759740939	1.52E-25	6.86E-25
AKR1C1	8.402919547	3.821344945	-1.136810178	1.97E-08	3.31E-08
AKR1C2	3.012281661	1.251670804	-1.267001499	0.000733587	0.000969684
HMOX1	24.87096883	128.4014111	2.368126444	1.97E-32	1.89E-31
MUC1	62.08031639	20.48334161	-1.599684817	2.05E-31	1.74E-30
MT1G	532.7081502	24.31696708	-4.453310148	1.42E-28	7.77E-28
SCD	11.16170549	90.01779866	3.0116528	3.02E-35	3.86E-34
PML	4.127491986	9.57467159	1.21395759	3.33E-36	5.90E-35
MTOR	9.448338153	4.229550758	-1.159556164	8.30E-37	1.88E-35
TP63	0.25876445	0.115187583	-1.167654226	1.94E-06	2.92E-06
ENPP2	23.147985	70.05449092	1.597590925	4.69E-11	9.23E-11
CBS	0.055601049	0.026033986	-1.094715851	3.51E-15	8.16E-15
CD44	7.405462972	19.1090518	1.367594356	2.19E-18	6.08E-18
HIF1A	77.61493319	33.72896895	-1.202346037	6.60E-24	2.37E-23
CA9	2.243136175	136.0519951	5.922497097	5.35E-38	1.76E-36
PLIN2	26.37278014	208.070087	2.979948003	2.63E-31	2.01E-30
PROM2	28.94209932	2.420898455	-3.5795551	1.99E-39	1.52E-37
CAV1	14.6949154	67.82712973	2.206545389	5.37E-35	6.50E-34
GCH1	8.123429958	3.89354356	-1.061005239	4.83E-20	1.48E-19
PTGS2	5.727144401	2.5058454	-1.19251857	2.04E-14	4.65E-14
NOS2	0.59413861	1.204800056	1.019922293	0.002423956	0.002997365
NCF2	2.062473179	6.994414958	1.761828031	8.44E-28	4.13E-27
MT3	0.906485847	8.786108145	3.276867857	1.05E-05	1.54E-05
ALB	54.80645898	21.52170558	-1.348553511	5.03E-16	1.23E-15
GPX2	1.981453477	0.833502518	-1.249300632	4.76E-28	2.43E-27
BNIP3	18.27417681	54.27520146	1.570486757	5.11E-30	3.36E-29
ANGPTL7	0.444157062	0.212461096	-1.063871234	2.38E-13	5.32E-13
CHAC1	4.404742008	1.083240905	-2.023703397	8.91E-28	4.27E-27
DDIT4	32.32598861	145.0282952	2.165568002	2.24E-31	1.84E-30
TSC22D3	35.17360778	78.12438941	1.151279689	2.71E-13	5.99E-13
SLC1A4	1.440338522	5.588999879	1.956182216	2.03E-38	9.33E-37
PCK2	50.91182556	10.95643502	-2.216222344	1.11E-09	2.09E-09
GPT2	7.45080825	3.420571386	-1.123159596	2.93E-18	8.02E-18
PSAT1	44.95856672	7.969221281	-2.496085381	4.15E-24	1.54E-23
LURAP1L	3.888376556	8.004429092	1.041630569	1.49E-16	3.90E-16
ATF3	46.77667497	20.46374967	-1.192718793	1.17E-09	2.17E-09
TRIB3	2.151184735	18.04057482	3.06804198	9.85E-36	1.51E-34
ATP6V1G2	0.972420825	0.464731309	-1.065183948	1.96E-25	8.68E-25
VEGFA	10.79031619	125.7155178	3.542353694	2.71E-37	7.66E-36
GDF15	79.55718229	34.44815336	-1.207565533	1.94E-16	4.90E-16
BLOC1S5-TXNDC5	0.729780425	0.198290467	-1.879847132	2.11E-15	4.96E-15
CXCL2	2.548180188	7.809354085	1.61573593	8.04E-08	1.32E-07
TF	0.247983323	4.357376627	4.135144811	1.88E-06	2.84E-06
HAMP	0.042262231	0.565979605	3.74330925	2.84E-29	1.68E-28
DRD4	0.160799212	0.651045903	2.01749893	1.40E-23	4.87E-23
SLC2A1	16.14210251	63.5368224	1.976764438	6.96E-30	4.33E-29
SLC2A3	5.570609986	22.7955221	2.032843235	1.51E-26	7.10E-26
SLC2A12	3.539748913	0.332891282	-3.410524036	1.12E-39	1.29E-37
SLC2A14	0.028615941	0.151614152	2.405513481	1.76E-19	5.19E-19
ALOX5	2.980667235	12.6033022	2.08009456	3.05E-25	1.30E-24
ALOX12	0.42439849	1.025930398	1.273441429	2.06E-24	7.90E-24
ACSF2	25.56426811	2.721778388	-3.231507213	4.86E-30	3.29E-29
TFAP2C	1.775898371	0.379051297	-2.228084016	2.65E-24	9.97E-24
NNMT	18.31632946	268.4626517	3.87351907	9.25E-36	1.51E-34
RRM2	0.665280767	2.89982108	2.123928657	2.81E-32	2.49E-31
CYBB	5.948485569	21.40530795	1.847374268	1.71E-20	5.30E-20

NOX4	13.94177603	5.867594815	-1.248573201	0.003417254	0.004136676
ACSL4	37.34497875	13.09191682	-1.512237936	2.99E-38	1.15E-36
TFR2	0.048947392	0.754157325	3.945561616	5.32E-29	2.98E-28
GOT1	84.95069431	40.84269312	-1.05654774	4.68E-28	2.43E-27
ALOX12B	0.090868373	0.354813187	1.965209486	1.93E-16	4.90E-16
ALOX15B	0.078100228	1.511289479	4.274309451	2.52E-34	2.90E-33
ACO1	25.11522347	12.09053945	-1.054683497	1.86E-24	7.38E-24
GABARAPL1	84.36010986	28.69902076	-1.555559462	3.00E-37	7.66E-36
EGFR	10.23830246	28.39874681	1.471850735	8.44E-24	2.99E-23
BID	2.75022775	5.892018811	1.099210941	1.10E-35	1.58E-34
CDKN2A	0.063563294	1.927830869	4.922640777	7.21E-42	1.66E-39
PEBP1	663.8088472	285.8428593	-1.215545607	9.00E-33	9.00E-32
CDO1	1.754360218	0.56376283	-1.637784737	9.38E-34	9.81E-33
MYB	0.071952959	0.189660884	1.398296246	1.26E-24	5.18E-24
LINC00472	4.818639603	1.329253511	-1.858009626	2.14E-35	2.89E-34
HILPDA	4.372160292	112.6098692	4.686843168	6.58E-39	3.79E-37
IFNG	0.033548735	0.770130505	4.520772651	6.90E-30	4.33E-29
TNFAIP3	6.156998153	15.3819319	1.320937669	3.51E-25	1.47E-24
MIOX	203.7872566	43.78182319	-2.2186599	2.88E-06	4.31E-06
TAZ	2.512156556	5.38509528	1.100045496	2.19E-28	1.17E-27

Table S3 GO and KEGG analysis in DEGs

ONTOLOGY	ID	Description	GeneRatio	pvalue	qvalue	Count
BP	GO:0062197	cellular response to	17/74	2.58E-14	3.43E-11	17
BP	GO:0001666	response to hypoxia	17/74	3.90E-14	3.43E-11	17
BP	GO:0036293	response to decreas	17/74	6.39E-14	3.74E-11	17
BP	GO:0070482	response to oxygen	17/74	1.77E-13	7.80E-11	17
BP	GO:0072593	reactive oxygen spe	15/74	3.05E-13	1.07E-10	15
BP	GO:0051186	cofactor metabolic p	17/74	1.45E-12	4.26E-10	17
BP	GO:0034599	cellular response to	14/74	1.19E-11	2.98E-09	14
BP	GO:0006979	response to oxidativ	16/74	1.92E-11	4.22E-09	16
BP	GO:0097193	intrinsic apoptotic si	13/74	1.01E-10	1.97E-08	13
BP	GO:0010038	response to metal io	14/74	1.32E-10	2.31E-08	14
BP	GO:0006801	superoxide metaboli	8/74	4.52E-10	7.22E-08	8
BP	GO:0046916	cellular transition m	9/74	4.93E-10	7.22E-08	9
BP	GO:0046394	carboxylic acid biosy	13/74	1.26E-09	1.63E-07	13
BP	GO:0016053	organic acid biosynt	13/74	1.30E-09	1.63E-07	13
BP	GO:0071453	cellular response to	11/74	1.98E-09	2.32E-07	11
BP	GO:0055076	transition metal ion	9/74	2.69E-09	2.95E-07	9
BP	GO:0006631	fatty acid metabolic	13/74	3.14E-09	3.13E-07	13
BP	GO:0010039	response to iron ion	6/74	3.21E-09	3.13E-07	6
BP	GO:0019372	lipoxygenase pathw	5/74	3.60E-09	3.33E-07	5
BP	GO:2000377	regulation of reactiv	10/74	4.93E-09	4.33E-07	10
BP	GO:0006879	cellular iron ion hom	7/74	5.33E-09	4.46E-07	7
BP	GO:0031667	response to nutrient	14/74	8.31E-09	6.41E-07	14
BP	GO:0071496	cellular response to	12/74	8.46E-09	6.41E-07	12
BP	GO:0071456	cellular response to	10/74	8.75E-09	6.41E-07	10
BP	GO:0036294	cellular response to	10/74	1.37E-08	9.66E-07	10
BP	GO:1903409	reactive oxygen spe	8/74	2.77E-08	1.87E-06	8
BP	GO:0055072	iron ion homeostasi	7/74	3.16E-08	2.06E-06	7
BP	GO:0097237	cellular response to	10/74	4.68E-08	2.94E-06	10
BP	GO:0048871	multicellular organis	13/74	5.29E-08	3.20E-06	13
BP	GO:0071248	cellular response to	9/74	5.53E-08	3.24E-06	9
BP	GO:2001233	regulation of apopto	12/74	6.19E-08	3.51E-06	12
BP	GO:0006636	unsaturated fatty ac	6/74	7.01E-08	3.85E-06	6
BP	GO:0001101	response to acid ch	11/74	1.02E-07	5.44E-06	11
BP	GO:0042759	long-chain fatty aci	5/74	1.12E-07	5.82E-06	5
BP	GO:0070997	neuron death	11/74	1.18E-07	5.93E-06	11
BP	GO:0035690	cellular response to	12/74	1.24E-07	6.06E-06	12
BP	GO:0048732	gland development	12/74	1.28E-07	6.06E-06	12
BP	GO:0071241	cellular response to	9/74	1.74E-07	7.90E-06	9
BP	GO:0046686	response to cadmiu	6/74	1.79E-07	7.90E-06	6
BP	GO:0008630	intrinsic apoptotic si	7/74	1.80E-07	7.90E-06	7
BP	GO:0036003	positive regulation o	5/74	1.86E-07	7.96E-06	5
BP	GO:0033559	unsaturated fatty ac	7/74	2.64E-07	1.11E-05	7
BP	GO:0042554	superoxide anion ge	5/74	2.92E-07	1.19E-05	5
BP	GO:1901568	fatty acid derivative	8/74	3.16E-07	1.25E-05	8
BP	GO:0051188	cofactor biosyntheti	9/74	3.20E-07	1.25E-05	9
BP	GO:0071276	cellular response to	5/74	3.37E-07	1.29E-05	5
BP	GO:0048660	regulation of smoot	8/74	3.46E-07	1.30E-05	8
BP	GO:0010634	positive regulation o	8/74	3.79E-07	1.36E-05	8
BP	GO:0048659	smooth muscle cell	8/74	3.79E-07	1.36E-05	8
BP	GO:0043618	regulation of transc	7/74	5.07E-07	1.78E-05	7
BP	GO:0016999	antibiotic metabolic	7/74	5.36E-07	1.85E-05	7
BP	GO:0006809	nitric oxide biosynth	6/74	5.99E-07	2.02E-05	6
BP	GO:0043620	regulation of DNA-t	7/74	7.04E-07	2.33E-05	7
BP	GO:0046209	nitric oxide metaboli	6/74	8.70E-07	2.83E-05	6
BP	GO:0042771	intrinsic apoptotic si	5/74	9.21E-07	2.94E-05	5

BP	GO:0062012	regulation of small r	11/74	9.44E-07	2.96E-05	11
BP	GO:2001057	reactive nitrogen sp	6/74	1.08E-06	3.30E-05	6
BP	GO:0043434	response to peptide	11/74	1.11E-06	3.30E-05	11
BP	GO:0031668	cellular response to	9/74	1.11E-06	3.30E-05	9
BP	GO:0033135	regulation of peptid	7/74	1.29E-06	3.79E-05	7
BP	GO:1901605	alpha-amino acid m	8/74	1.44E-06	4.15E-05	8
BP	GO:0071229	cellular response to	8/74	1.73E-06	4.89E-05	8
BP	GO:0015980	energy derivation by	9/74	1.89E-06	5.29E-05	9
BP	GO:0043281	regulation of cystein	8/74	2.13E-06	5.85E-05	8
BP	GO:0048661	positive regulation c	6/74	2.97E-06	8.03E-05	6
BP	GO:0031960	response to corticos	7/74	3.59E-06	9.57E-05	7
BP	GO:0033138	positive regulation c	6/74	3.72E-06	9.77E-05	6
BP	GO:0006633	fatty acid biosynthe	7/74	3.90E-06	0.000100224	7
BP	GO:0042136	neurotransmitter bio	6/74	3.93E-06	0.000100224	6
BP	GO:0051341	regulation of oxidor	6/74	4.15E-06	0.000104335	6
BP	GO:0031669	cellular response to	8/74	4.39E-06	0.000108676	8
BP	GO:0001676	long-chain fatty aci	6/74	4.63E-06	0.000110932	6
BP	GO:0033002	muscle cell prolifera	8/74	4.67E-06	0.000110932	8
BP	GO:2000116	regulation of cystein	8/74	4.67E-06	0.000110932	8
BP	GO:0032768	regulation of mono	5/74	5.85E-06	0.000136944	5
BP	GO:0003018	vascular process in c	7/74	5.98E-06	0.000136944	7
BP	GO:0006690	icosanoid metabolic	6/74	6.00E-06	0.000136944	6
BP	GO:0043457	regulation of cellula	4/74	6.60E-06	0.000148675	4
BP	GO:0071281	cellular response to	3/74	7.03E-06	0.000156455	3
BP	GO:0006732	coenzyme metaboli	8/74	7.51E-06	0.000165078	8
BP	GO:0050730	regulation of peptid	8/74	7.73E-06	0.000167769	8
BP	GO:0043619	regulation of transc	3/74	9.64E-06	0.000205784	3
BP	GO:0000041	transition metal ion	6/74	9.74E-06	0.000205784	6
BP	GO:0044282	small molecule cata	10/74	9.83E-06	0.000205784	10
BP	GO:1901617	organic hydroxy cor	8/74	1.02E-05	0.000211369	8
BP	GO:0042594	response to starvati	7/74	1.06E-05	0.000216656	7
BP	GO:0052547	regulation of peptid	10/74	1.13E-05	0.000226585	10
BP	GO:1901654	response to ketone	7/74	1.13E-05	0.000226585	7
BP	GO:0045333	cellular respiration	7/74	1.17E-05	0.000231696	7
BP	GO:0006826	iron ion transport	5/74	1.27E-05	0.000245915	5
BP	GO:0006534	cysteine metabolic p	3/74	1.28E-05	0.000245915	3
BP	GO:0016054	organic acid catabo	8/74	1.30E-05	0.000245915	8
BP	GO:0046395	carboxylic acid catal	8/74	1.30E-05	0.000245915	8
BP	GO:0072332	intrinsic apoptotic si	5/74	1.44E-05	0.000268824	5
BP	GO:0006066	alcohol metabolic p	9/74	1.52E-05	0.000277258	9
BP	GO:0043536	positive regulation c	5/74	1.53E-05	0.000277258	5
BP	GO:0071260	cellular response to	5/74	1.53E-05	0.000277258	5
BP	GO:0098754	detoxification	6/74	1.72E-05	0.000304221	6
BP	GO:0008652	cellular amino acid b	5/74	1.73E-05	0.000304221	5
BP	GO:0050679	positive regulation c	7/74	1.73E-05	0.000304221	7
BP	GO:0010632	regulation of epithe	8/74	1.96E-05	0.000340405	8
BP	GO:0048545	response to steroid	9/74	2.00E-05	0.000344104	9
BP	GO:0000096	sulfur amino acid m	4/74	2.06E-05	0.000348288	4
BP	GO:0009069	serine family amino	4/74	2.06E-05	0.000348288	4
BP	GO:0035296	regulation of tube d	6/74	2.19E-05	0.000363882	6
BP	GO:0097746	regulation of blood	6/74	2.19E-05	0.000363882	6
BP	GO:0035150	regulation of tube s	6/74	2.28E-05	0.000374983	6
BP	GO:0090559	regulation of memb	5/74	2.31E-05	0.000376605	5
BP	GO:0018105	peptidyl-serine pho	8/74	2.38E-05	0.000383089	8
BP	GO:0015711	organic anion transp	10/74	2.46E-05	0.000390788	10
BP	GO:0051384	response to glucocc	6/74	2.47E-05	0.000390788	6
BP	GO:0007584	response to nutrient	7/74	2.57E-05	0.000402673	7
BP	GO:0002791	regulation of peptid	10/74	2.68E-05	0.0004172	10

BP	GO:0097191	extrinsic apoptotic s	7/74	2.96E-05	0.000457108	7
BP	GO:0042133	neurotransmitter m	6/74	3.21E-05	0.000490352	6
BP	GO:0045981	positive regulation (	4/74	3.27E-05	0.000490352	4
BP	GO:1900544	positive regulation (	4/74	3.27E-05	0.000490352	4
BP	GO:1901214	regulation of neuro	8/74	3.29E-05	0.000490352	8
BP	GO:0007589	body fluid secretion	5/74	3.38E-05	0.000499201	5
BP	GO:0001894	tissue homeostasis	7/74	3.41E-05	0.000499964	7
BP	GO:2001236	regulation of extrins	6/74	3.46E-05	0.000502136	6
BP	GO:2001234	negative regulation	7/74	3.51E-05	0.000505613	7
BP	GO:0000302	response to reactive	7/74	3.71E-05	0.000526543	7
BP	GO:0043467	regulation of genera	6/74	3.71E-05	0.000526543	6
BP	GO:0007595	lactation	4/74	3.88E-05	0.000536633	4
BP	GO:0043651	linoleic acid metabo	3/74	3.91E-05	0.000536633	3
BP	GO:0007568	aging	8/74	3.94E-05	0.000536633	8
BP	GO:0010660	regulation of muscle	5/74	3.94E-05	0.000536633	5
BP	GO:0048010	vascular endothelial	5/74	3.94E-05	0.000536633	5
BP	GO:0018209	peptidyl-serine mod	8/74	4.02E-05	0.000544149	8
BP	GO:0030857	negative regulation	4/74	4.21E-05	0.000560525	4
BP	GO:2001238	positive regulation (	4/74	4.21E-05	0.000560525	4
BP	GO:0072330	monocarboxylic acid	7/74	4.25E-05	0.000561195	7
BP	GO:2001243	negative regulation	5/74	4.35E-05	0.000570454	5
BP	GO:0051402	neuron apoptotic p	7/74	4.48E-05	0.000577845	7
BP	GO:0046677	response to antibiot	8/74	4.49E-05	0.000577845	8
BP	GO:0052548	regulation of endop	9/74	4.50E-05	0.000577845	9
BP	GO:1901570	fatty acid derivative	5/74	4.57E-05	0.000579797	5
BP	GO:0010506	regulation of autopl	8/74	4.58E-05	0.000579797	8
BP	GO:0010657	muscle cell apoptot	5/74	4.79E-05	0.000601607	5
BP	GO:0001933	negative regulation	9/74	4.84E-05	0.000602408	9
BP	GO:2001242	regulation of intrins	6/74	4.90E-05	0.000602408	6
BP	GO:0019369	arachidonic acid me	4/74	4.93E-05	0.000602408	4
BP	GO:0050999	regulation of nitric-	4/74	4.93E-05	0.000602408	4
BP	GO:0098869	cellular oxidant det	5/74	5.27E-05	0.000634306	5
BP	GO:2000379	positive regulation (	5/74	5.27E-05	0.000634306	5
BP	GO:0042180	cellular ketone meta	7/74	5.38E-05	0.000642852	7
BP	GO:0034614	cellular response to	6/74	5.42E-05	0.000644082	6
BP	GO:0030949	positive regulation (	3/74	5.53E-05	0.000652869	3
BP	GO:0060249	anatomical structure	9/74	5.78E-05	0.000675559	9
BP	GO:0045926	negative regulation	7/74	5.80E-05	0.000675559	7
BP	GO:1901606	alpha-amino acid ca	5/74	6.33E-05	0.000732243	5
BP	GO:0034755	iron ion transmemb	3/74	6.49E-05	0.000745872	3
BP	GO:1904659	glucose transmemb	5/74	6.92E-05	0.000787734	5
BP	GO:0006520	cellular amino acid	8/74	6.95E-05	0.000787734	8
BP	GO:0010631	epithelial cell migra	8/74	7.52E-05	0.000847656	8
BP	GO:2001235	positive regulation (	6/74	7.71E-05	0.000863207	6
BP	GO:0001505	regulation of neuro	8/74	7.98E-05	0.000877711	8
BP	GO:0090132	epithelium migratio	8/74	7.98E-05	0.000877711	8
BP	GO:0046883	regulation of hormo	7/74	7.99E-05	0.000877711	7
BP	GO:0032722	positive regulation (	4/74	8.20E-05	0.000887418	4
BP	GO:0008645	hexose transmembr	5/74	8.23E-05	0.000887418	5
BP	GO:1990748	cellular detoxificatio	5/74	8.23E-05	0.000887418	5
BP	GO:0001667	ameboidal-type cell	9/74	8.55E-05	0.000916697	9
BP	GO:1901522	positive regulation (	3/74	8.72E-05	0.000928994	3
BP	GO:0097755	positive regulation (	4/74	8.77E-05	0.000929214	4
BP	GO:0015749	monosaccharide tra	5/74	8.95E-05	0.000938213	5
BP	GO:0090130	tissue migration	8/74	8.97E-05	0.000938213	8
BP	GO:0018108	peptidyl-tyrosine ph	8/74	9.32E-05	0.000964248	8
BP	GO:1900542	regulation of purine	5/74	9.33E-05	0.000964248	5
BP	GO:0043491	protein kinase B sig	7/74	9.42E-05	0.000964248	7

BP	GO:0042326	negative regulation	9/74	9.43E-05	0.000964248	9
BP	GO:0022900	electron transport c	6/74	9.53E-05	0.000967956	6
BP	GO:0007569	cell aging	5/74	9.72E-05	0.000976153	5
BP	GO:0034219	carbohydrate transn	5/74	9.72E-05	0.000976153	5
BP	GO:0018212	peptidyl-tyrosine m	8/74	9.86E-05	0.000985166	8
BP	GO:0061436	establishment of ski	3/74	1.00E-04	0.00099308	3
BP	GO:0050708	regulation of protei	9/74	0.000100645	0.000993889	9
BP	GO:0006140	regulation of nuclec	5/74	0.000101204	0.000993889	5
BP	GO:0006790	sulfur compound m	8/74	0.00011037	0.001077884	8
BP	GO:0044272	sulfur compound bi	6/74	0.000113407	0.00109537	6
BP	GO:0050731	positive regulation c	6/74	0.000113407	0.00109537	6
BP	GO:0022612	gland morphogenes	5/74	0.000114059	0.001095646	5
BP	GO:0009165	nucleotide biosynth	7/74	0.000115585	0.001104269	7
BP	GO:2000378	negative regulation	4/74	0.00012068	0.001146722	4
BP	GO:0050678	regulation of epithe	8/74	0.00012324	0.001164751	8
BP	GO:1901293	nucleoside phospha	7/74	0.000126284	0.001187134	7
BP	GO:0060571	morphogenesis of a	3/74	0.000129122	0.001207356	3
BP	GO:0045765	regulation of angiog	8/74	0.000134893	0.001245856	8
BP	GO:0019748	secondary metaboli	4/74	0.000136074	0.001245856	4
BP	GO:0045428	regulation of nitric c	4/74	0.000136074	0.001245856	4
BP	GO:1905710	positive regulation c	4/74	0.000136074	0.001245856	4
BP	GO:0009063	cellular amino acid c	5/74	0.000138227	0.001259008	5
BP	GO:0033561	regulation of water	3/74	0.000145549	0.001312492	3
BP	GO:0006914	autophagy	9/74	0.000146339	0.001312492	9
BP	GO:0061919	process utilizing aut	9/74	0.000146339	0.001312492	9
BP	GO:0010595	positive regulation c	5/74	0.000154501	0.001378658	5
BP	GO:0009410	response to xenobic	7/74	0.000156594	0.001390282	7
BP	GO:0030100	regulation of endoc	6/74	0.000162169	0.001432543	6
BP	GO:0090276	regulation of peptid	6/74	0.000175483	0.001542405	6
BP	GO:1990776	response to angiote	3/74	0.000182351	0.001594792	3
BP	GO:0051881	regulation of mitoch	4/74	0.000190787	0.001660317	4
BP	GO:0001889	liver development	5/74	0.000198164	0.001716019	5
BP	GO:0031100	animal organ regen	4/74	0.000201245	0.001725698	4
BP	GO:1903524	positive regulation c	4/74	0.000201245	0.001725698	4
BP	GO:0007263	nitric oxide mediate	3/74	0.000202813	0.001730698	3
BP	GO:0019216	regulation of lipid m	8/74	0.000214694	0.001823234	8
BP	GO:0009895	negative regulation	7/74	0.000217163	0.001826554	7
BP	GO:0046879	hormone secretion	7/74	0.000217163	0.001826554	7
BP	GO:0061008	hepaticobiliary syste	5/74	0.000219519	0.001837577	5
BP	GO:0071549	cellular response to	3/74	0.000224707	0.001863264	3
BP	GO:0097421	liver regeneration	3/74	0.000224707	0.001863264	3
BP	GO:0010876	lipid localization	8/74	0.000233051	0.001923372	8
BP	GO:0062013	positive regulation c	5/74	0.000242584	0.001992697	5
BP	GO:0032770	positive regulation c	3/74	0.000248076	0.002018946	3
BP	GO:1902230	negative regulation	3/74	0.000248076	0.002018946	3
BP	GO:0009914	hormone transport	7/74	0.000258781	0.002088145	7
BP	GO:0030879	mammary gland de	5/74	0.000258955	0.002088145	5
BP	GO:1901342	regulation of vascul	8/74	0.0002609	0.00209422	8
BP	GO:0045907	positive regulation c	3/74	0.000272962	0.002181083	3
BP	GO:0009108	coenzyme biosynthe	5/74	0.000285071	0.002267529	5
BP	GO:0008643	carbohydrate transp	5/74	0.00030356	0.002403726	5
BP	GO:0006163	purine nucleotide m	8/74	0.000305372	0.002407226	8
BP	GO:0009267	cellular response to	5/74	0.00031314	0.002449934	5
BP	GO:0032642	regulation of chemc	4/74	0.000314817	0.002449934	4
BP	GO:0050673	epithelial cell prolife	8/74	0.000314971	0.002449934	8
BP	GO:0006970	response to osmotic	4/74	0.000329772	0.002542562	4
BP	GO:2000106	regulation of leukoc	4/74	0.000329772	0.002542562	4
BP	GO:0046942	carboxylic acid trans	7/74	0.0003361	0.002557701	7

BP	GO:0071214	cellular response to	7/74	0.0003361	0.002557701	7
BP	GO:0104004	cellular response to	7/74	0.0003361	0.002557701	7
BP	GO:0043154	negative regulation	4/74	0.000345229	0.002615842	4
BP	GO:0015849	organic acid transpo	7/74	0.000348518	0.002629434	7
BP	GO:0072525	pyridine-containing	3/74	0.000357129	0.002671467	3
BP	GO:1905332	positive regulation (	3/74	0.000357129	0.002671467	3
BP	GO:0043405	regulation of MAP k	7/74	0.000374459	0.002777975	7
BP	GO:0001776	leukocyte homeosta	4/74	0.000377688	0.002777975	4
BP	GO:0008625	extrinsic apoptotic s	4/74	0.000377688	0.002777975	4
BP	GO:0034637	cellular carbohydrat	4/74	0.000377688	0.002777975	4
BP	GO:0030856	regulation of epithe	5/74	0.000386768	0.00279334	5
BP	GO:0043535	regulation of blood	5/74	0.000386768	0.00279334	5
BP	GO:0030947	regulation of vascul	3/74	0.000388489	0.00279334	3
BP	GO:0033572	transferrin transport	3/74	0.000388489	0.00279334	3
BP	GO:0071634	regulation of transfo	3/74	0.000388489	0.00279334	3
BP	GO:0009314	response to radiatio	8/74	0.000389311	0.00279334	8
BP	GO:0043271	negative regulation	5/74	0.000398269	0.002845993	5
BP	GO:0051896	regulation of protei	6/74	0.000413286	0.002941349	6
BP	GO:0038083	peptidyl-tyrosine au	3/74	0.000421567	0.00297619	3
BP	GO:0090050	positive regulation (	3/74	0.000421567	0.00297619	3
BP	GO:0032602	chemokine producti	4/74	0.00043039	0.003022464	4
BP	GO:0048872	homeostasis of num	6/74	0.000431561	0.003022464	6
BP	GO:0010939	regulation of necrot	3/74	0.000456403	0.003158693	3
BP	GO:0050892	intestinal absorptior	3/74	0.000456403	0.003158693	3
BP	GO:0071604	transforming growtl	3/74	0.000456403	0.003158693	3
BP	GO:0030072	peptide hormone se	6/74	0.000470003	0.003240061	6
BP	GO:0015718	monocarboxylic aci	5/74	0.000472796	0.003246583	5
BP	GO:2000117	negative regulation	4/74	0.000488143	0.003308018	4
BP	GO:0001819	positive regulation (	8/74	0.000491204	0.003308018	8
BP	GO:0072521	purine-containing c	8/74	0.000491204	0.003308018	8
BP	GO:0010661	positive regulation (	3/74	0.000493033	0.003308018	3
BP	GO:0072524	pyridine-containing	3/74	0.000493033	0.003308018	3
BP	GO:1902229	regulation of intrins	3/74	0.000493033	0.003308018	3
BP	GO:1901655	cellular response to	4/74	0.000508564	0.003399245	4
BP	GO:0035051	cardiocyte differenti	5/74	0.000528019	0.003512464	5
BP	GO:1901216	positive regulation (	4/74	0.000529585	0.003512464	4
BP	GO:0071548	response to dexame	3/74	0.000531497	0.003512464	3
BP	GO:0045861	negative regulation	7/74	0.000537252	0.003537202	7
BP	GO:0050873	brown fat cell differe	3/74	0.00057183	0.003722357	3
BP	GO:0019217	regulation of fatty a	4/74	0.000573471	0.003722357	4
BP	GO:0097327	response to antineo	4/74	0.000573471	0.003722357	4
BP	GO:0031331	positive regulation (	7/74	0.000573845	0.003722357	7
BP	GO:0016241	regulation of macro	5/74	0.000587963	0.003799913	5
BP	GO:0015908	fatty acid transport	4/74	0.000596357	0.003840044	4
BP	GO:0046165	alcohol biosynthetic	5/74	0.000652881	0.004188671	5
BP	GO:0045429	positive regulation (	3/74	0.000658254	0.004207786	3
BP	GO:0001936	regulation of endotl	5/74	0.000669918	0.004251427	5
BP	GO:0051897	positive regulation (	5/74	0.000669918	0.004251427	5
BP	GO:0010749	regulation of nitric c	2/74	0.000683228	0.004274174	2
BP	GO:0030647	aminoglycoside anti	2/74	0.000683228	0.004274174	2
BP	GO:0033483	gas homeostasis	2/74	0.000683228	0.004274174	2
BP	GO:2000271	positive regulation (	2/74	0.000683228	0.004274174	2
BP	GO:1903426	regulation of reactiv	4/74	0.000694412	0.004328733	4
BP	GO:0014002	astrocyte developm	3/74	0.000704416	0.004360173	3
BP	GO:1904407	positive regulation (	3/74	0.000704416	0.004360173	3
BP	GO:0048771	tissue remodeling	5/74	0.000723033	0.004459704	5
BP	GO:0032868	response to insulin	6/74	0.000731676	0.004497238	6
BP	GO:0010565	regulation of cellula	5/74	0.000741419	0.004509817	5

BP	GO:0043534	blood vessel endoth	5/74	0.000741419	0.004509817	5
BP	GO:0071466	cellular response to	5/74	0.000741419	0.004509817	5
BP	GO:0034766	negative regulation	4/74	0.00074749	0.004531064	4
BP	GO:1902743	regulation of lamelli	3/74	0.000752592	0.004546313	3
BP	GO:0006869	lipid transport	7/74	0.000763867	0.004598622	7
BP	GO:0071887	leukocyte apoptotic	4/74	0.000775077	0.004638261	4
BP	GO:0042176	regulation of protei	7/74	0.000775728	0.004638261	7
BP	GO:1904036	negative regulation	3/74	0.000802816	0.004783953	3
BP	GO:2000045	regulation of G1/S t	5/74	0.000818484	0.004860519	5
BP	GO:0019852	L-ascorbic acid met	2/74	0.000832914	0.004860519	2
BP	GO:0046886	positive regulation c	2/74	0.000832914	0.004860519	2
BP	GO:0048548	regulation of pinocy	2/74	0.000832914	0.004860519	2
BP	GO:0072584	caveolin-mediated e	2/74	0.000832914	0.004860519	2
BP	GO:2000392	regulation of lamelli	2/74	0.000832914	0.004860519	2
BP	GO:0000082	G1/S transition of m	6/74	0.00083502	0.004860519	6
BP	GO:0006953	acute-phase respon	3/74	0.000855123	0.004944791	3
BP	GO:1903580	positive regulation c	3/74	0.000855123	0.004944791	3
BP	GO:1905475	regulation of protei	5/74	0.000880096	0.005070408	5
BP	GO:0044262	cellular carbohydrat	6/74	0.000882615	0.005070408	6
BP	GO:0014706	striated muscle tissu	7/74	0.000889189	0.005091535	7
BP	GO:0008542	visual learning	3/74	0.000909547	0.005191193	3
BP	GO:0010959	regulation of metal	7/74	0.00094365	0.005368408	7
BP	GO:0048546	digestive tract morp	3/74	0.00096612	0.005451422	3
BP	GO:0005996	monosaccharide me	6/74	0.000966569	0.005451422	6
BP	GO:0001935	endothelial cell prol	5/74	0.000967546	0.005451422	5
BP	GO:1904019	epithelial cell apopt	4/74	0.000988703	0.005544555	4
BP	GO:0043112	receptor metabolic	5/74	0.000990384	0.005544555	5
BP	GO:0006563	L-serine metabolic p	2/74	0.000996934	0.0055459	2
BP	GO:0045080	positive regulation c	2/74	0.000996934	0.0055459	2
BP	GO:0009150	purine ribonucleotic	7/74	0.001015397	0.005630074	7
BP	GO:0042063	gliogenesis	6/74	0.001019817	0.005630074	6
BP	GO:0001938	positive regulation c	4/74	0.001022274	0.005630074	4
BP	GO:0042398	cellular modified am	3/74	0.001024876	0.005630074	3
BP	GO:0021987	cerebral cortex deve	4/74	0.001056639	0.005768508	4
BP	GO:0043200	response to amino a	4/74	0.001056639	0.005768508	4
BP	GO:0048146	positive regulation c	3/74	0.001085846	0.005909609	3
BP	GO:0016236	macroautophagy	6/74	0.001113552	0.006041691	6
BP	GO:0050890	cognition	6/74	0.001133069	0.006125343	6
BP	GO:0046890	regulation of lipid b	5/74	0.001135939	0.006125343	5
BP	GO:0060537	muscle tissue develo	7/74	0.001155338	0.006185123	7
BP	GO:0034341	response to interfer	5/74	0.001161661	0.006185123	5
BP	GO:0010822	positive regulation c	4/74	0.001164598	0.006185123	4
BP	GO:0044843	cell cycle G1/S phas	6/74	0.001172889	0.006185123	6
BP	GO:0010870	positive regulation c	2/74	0.001175173	0.006185123	2
BP	GO:0010940	positive regulation c	2/74	0.001175173	0.006185123	2
BP	GO:0050713	negative regulation	2/74	0.001175173	0.006185123	2
BP	GO:0090594	inflammatory respo	2/74	0.001175173	0.006185123	2
BP	GO:0007632	visual behavior	3/74	0.001214559	0.006354364	3
BP	GO:0010043	response to zinc ior	3/74	0.001214559	0.006354364	3
BP	GO:1902806	regulation of cell cy	5/74	0.001241421	0.006475629	5
BP	GO:0009259	ribonucleotide meta	7/74	0.001256765	0.006536274	7
BP	GO:0051353	positive regulation c	3/74	0.001282363	0.006630172	3
BP	GO:0097345	mitochondrial outer	3/74	0.001282363	0.006630172	3
BP	GO:0001558	regulation of cell gr	7/74	0.001292085	0.006660847	7
BP	GO:0045766	positive regulation c	5/74	0.001296801	0.006665614	5
BP	GO:0010508	positive regulation c	4/74	0.001320277	0.006766497	4
BP	GO:0044273	sulfur compound ca	3/74	0.001352506	0.006848885	3
BP	GO:0046456	icosanoid biosynthe	3/74	0.001352506	0.006848885	3

BP	GO:1903578	regulation of ATP m	4/74	0.00136136	0.006848885	4
BP	GO:0009071	serine family amino	2/74	0.001367521	0.006848885	2
BP	GO:0034356	NAD biosynthesis vi	2/74	0.001367521	0.006848885	2
BP	GO:0050667	homocysteine meta	2/74	0.001367521	0.006848885	2
BP	GO:0051712	positive regulation c	2/74	0.001367521	0.006848885	2
BP	GO:0061051	positive regulation c	2/74	0.001367521	0.006848885	2
BP	GO:1905477	positive regulation c	4/74	0.001403328	0.007008247	4
BP	GO:0007623	circadian rhythm	5/74	0.001413021	0.007016785	5
BP	GO:0016051	carbohydrate biosyr	5/74	0.001413021	0.007016785	5
BP	GO:0019693	ribose phosphate m	7/74	0.001441231	0.00713671	7
BP	GO:0009896	positive regulation c	7/74	0.001460784	0.007213214	7
BP	GO:0009612	response to mechar	5/74	0.001473932	0.007257752	5
BP	GO:0019751	polyol metabolic pr	4/74	0.001489961	0.007316187	4
BP	GO:0042743	hydrogen peroxide	3/74	0.00149993	0.007324219	3
BP	GO:1903428	positive regulation c	3/74	0.00149993	0.007324219	3
BP	GO:0051047	positive regulation c	7/74	0.001520692	0.007405029	7
BP	GO:0006898	receptor-mediated	6/74	0.001531452	0.007411394	6
BP	GO:0009416	response to light sti	6/74	0.001531452	0.007411394	6
BP	GO:0045471	response to ethanol	4/74	0.001534647	0.007411394	4
BP	GO:0000098	sulfur amino acid ca	2/74	0.001573866	0.007453422	2
BP	GO:0010273	detoxification of co	2/74	0.001573866	0.007453422	2
BP	GO:0032352	positive regulation c	2/74	0.001573866	0.007453422	2
BP	GO:0045073	regulation of chemo	2/74	0.001573866	0.007453422	2
BP	GO:1990169	stress response to c	2/74	0.001573866	0.007453422	2
BP	GO:0019229	regulation of vasoc	3/74	0.001577269	0.007453422	3
BP	GO:0071385	cellular response to	3/74	0.001577269	0.007453422	3
BP	GO:1902041	regulation of extrins	3/74	0.001577269	0.007453422	3
BP	GO:0048608	reproductive structu	7/74	0.001582505	0.007458116	7
BP	GO:2001020	regulation of respor	5/74	0.00160154	0.007527642	5
BP	GO:0046887	positive regulation c	4/74	0.001626806	0.007626008	4
BP	GO:0061458	reproductive system	7/74	0.001646266	0.007696707	7
BP	GO:0034763	negative regulation	4/74	0.001674299	0.007807008	4
BP	GO:0071375	cellular response to	6/74	0.001712317	0.007963155	6
BP	GO:0010001	glial cell differentiat	5/74	0.001737104	0.008025161	5
BP	GO:0002294	CD4-positive, alpha	3/74	0.001739346	0.008025161	3
BP	GO:1902110	positive regulation c	3/74	0.001739346	0.008025161	3
BP	GO:0051222	positive regulation c	7/74	0.001779797	0.008086748	7
BP	GO:0006103	2-oxoglutarate meta	2/74	0.001794096	0.008086748	2
BP	GO:0032225	regulation of synapt	2/74	0.001794096	0.008086748	2
BP	GO:0042033	chemokine biosynth	2/74	0.001794096	0.008086748	2
BP	GO:0042448	progesterone metal	2/74	0.001794096	0.008086748	2
BP	GO:0050711	negative regulation	2/74	0.001794096	0.008086748	2
BP	GO:0050755	chemokine metabol	2/74	0.001794096	0.008086748	2
BP	GO:0090335	regulation of brown	2/74	0.001794096	0.008086748	2
BP	GO:1902166	negative regulation	2/74	0.001794096	0.008086748	2
BP	GO:0002287	alpha-beta T cell ac	3/74	0.001824139	0.008159399	3
BP	GO:0002293	alpha-beta T cell di	3/74	0.001824139	0.008159399	3
BP	GO:0071384	cellular response to	3/74	0.001824139	0.008159399	3
BP	GO:0055007	cardiac muscle cell c	4/74	0.001873937	0.008360873	4
BP	GO:0002260	lymphocyte homeos	3/74	0.001911472	0.008379466	3
BP	GO:0031640	killing of cells of oth	3/74	0.001911472	0.008379466	3
BP	GO:0045453	bone resorption	3/74	0.001911472	0.008379466	3
BP	GO:0051205	protein insertion int	3/74	0.001911472	0.008379466	3
BP	GO:0070059	intrinsic apoptotic si	3/74	0.001911472	0.008379466	3
BP	GO:0070265	necrotic cell death	3/74	0.001911472	0.008379466	3
BP	GO:1902686	mitochondrial outer	3/74	0.001911472	0.008379466	3
BP	GO:0042770	signal transduction	4/74	0.001926312	0.008423518	4
BP	GO:0032496	response to lipopoly	6/74	0.001968238	0.008571659	6

BP	GO:0006766	vitamin metabolic p	4/74	0.001979694	0.008571659	4
BP	GO:0007006	mitochondrial mem	4/74	0.001979694	0.008571659	4
BP	GO:0046683	response to organo	4/74	0.001979694	0.008571659	4
BP	GO:0000097	sulfur amino acid bi	2/74	0.002028101	0.008632418	2
BP	GO:0006750	glutathione biosyntl	2/74	0.002028101	0.008632418	2
BP	GO:0009070	serine family amino	2/74	0.002028101	0.008632418	2
BP	GO:0051709	regulation of killing	2/74	0.002028101	0.008632418	2
BP	GO:0061687	detoxification of inc	2/74	0.002028101	0.008632418	2
BP	GO:0072673	lamellipodium morp	2/74	0.002028101	0.008632418	2
BP	GO:0097501	stress response to n	2/74	0.002028101	0.008632418	2
BP	GO:0032147	activation of protei	6/74	0.002059682	0.008745661	6
BP	GO:0071902	positive regulation c	6/74	0.002090867	0.008805725	6
BP	GO:0035794	positive regulation c	3/74	0.002093864	0.008805725	3
BP	GO:1901607	alpha-amino acid b	3/74	0.002093864	0.008805725	3
BP	GO:1903672	positive regulation c	3/74	0.002093864	0.008805725	3
BP	GO:0010594	regulation of endotl	5/74	0.002153347	0.009034267	5
BP	GO:1904951	positive regulation c	7/74	0.002177391	0.00911339	7
BP	GO:0051187	cofactor catabolic p	3/74	0.002188977	0.009119839	3
BP	GO:0009743	response to carboh	5/74	0.002194495	0.009119839	5
BP	GO:1904018	positive regulation c	5/74	0.002194495	0.009119839	5
BP	GO:0007586	digestion	4/74	0.002262044	0.009347122	4
BP	GO:0060749	mammary gland alv	2/74	0.002275772	0.009347122	2
BP	GO:0061377	mammary gland lob	2/74	0.002275772	0.009347122	2
BP	GO:0070233	negative regulation	2/74	0.002275772	0.009347122	2
BP	GO:1902165	regulation of intrins	2/74	0.002275772	0.009347122	2
BP	GO:1902108	regulation of mitocl	3/74	0.002286734	0.009348459	3
BP	GO:1905207	regulation of cardio	3/74	0.002286734	0.009348459	3
BP	GO:0048738	cardiac muscle tissu	5/74	0.002321389	0.009446197	5
BP	GO:0097305	response to alcohol	5/74	0.002321389	0.009446197	5
BP	GO:0042108	positive regulation c	3/74	0.002387161	0.009649827	3
BP	GO:0048662	negative regulation	3/74	0.002387161	0.009649827	3
BP	GO:0002237	response to molecu	6/74	0.002387899	0.009649827	6
BP	GO:0006469	negative regulation	5/74	0.002408905	0.009712387	5
BP	GO:0002292	T cell differentiation	3/74	0.002490284	0.009926659	3
BP	GO:0016239	positive regulation c	3/74	0.002490284	0.009926659	3
BP	GO:0019915	lipid storage	3/74	0.002490284	0.009926659	3
BP	GO:0050891	multicellular organis	3/74	0.002490284	0.009926659	3
BP	GO:0071230	cellular response to	3/74	0.002490284	0.009926659	3
BP	GO:0007050	cell cycle arrest	5/74	0.002498801	0.009938075	5
BP	GO:0019184	nonribosomal pepti	2/74	0.002537	0.009954864	2
BP	GO:0032095	regulation of respor	2/74	0.002537	0.009954864	2
BP	GO:0034138	toll-like receptor 3 s	2/74	0.002537	0.009954864	2
BP	GO:0060252	positive regulation c	2/74	0.002537	0.009954864	2
BP	GO:0071243	cellular response to	2/74	0.002537	0.009954864	2
BP	GO:1902644	tertiary alcohol met	2/74	0.002537	0.009954864	2
BP	GO:0051607	defense response to	5/74	0.002544654	0.009962658	5
BP	GO:0007612	learning	4/74	0.002636139	0.010283065	4
BP	GO:0050663	cytokine secretion	5/74	0.002638191	0.010283065	5
BP	GO:0051260	protein homooligor	6/74	0.002677731	0.010414091	6
BP	GO:0042542	response to hydrog	4/74	0.002702367	0.010463603	4
BP	GO:0045834	positive regulation c	4/74	0.002702367	0.010463603	4
BP	GO:0002262	myeloid cell homeo	4/74	0.002769728	0.010675227	4
BP	GO:0071236	cellular response to	4/74	0.002769728	0.010675227	4
BP	GO:0006071	glycerol metabolic p	2/74	0.002811676	0.010675227	2
BP	GO:0016137	glycoside metabolic	2/74	0.002811676	0.010675227	2
BP	GO:0034393	positive regulation c	2/74	0.002811676	0.010675227	2
BP	GO:0042535	positive regulation c	2/74	0.002811676	0.010675227	2
BP	GO:0071636	positive regulation c	2/74	0.002811676	0.010675227	2

BP	GO:1903204	negative regulation	2/74	0.002811676	0.010675227	2
BP	GO:1903599	positive regulation c	2/74	0.002811676	0.010675227	2
BP	GO:0061041	regulation of wounc	4/74	0.002838232	0.010752831	4
BP	GO:0051155	positive regulation c	3/74	0.002930206	0.011077404	3
BP	GO:0045862	positive regulation c	6/74	0.002952385	0.011137299	6
BP	GO:0006090	pyruvate metabolic	4/74	0.00297871	0.011212547	4
BP	GO:0016049	cell growth	7/74	0.00303541	0.011401563	7
BP	GO:0021537	telencephalon deve	5/74	0.00309028	0.011471437	5
BP	GO:0071560	cellular response to	5/74	0.00309028	0.011471437	5
BP	GO:0006907	pinocytosis	2/74	0.003099692	0.011471437	2
BP	GO:0035162	embryonic hemopo	2/74	0.003099692	0.011471437	2
BP	GO:0051900	regulation of mitocl	2/74	0.003099692	0.011471437	2
BP	GO:0055093	response to hypero:	2/74	0.003099692	0.011471437	2
BP	GO:2000269	regulation of fibrobl	2/74	0.003099692	0.011471437	2
BP	GO:0010951	negative regulation	5/74	0.003143755	0.011598266	5
BP	GO:0009064	glutamine family am	3/74	0.003166952	0.011598266	3
BP	GO:0030104	water homeostasis	3/74	0.003166952	0.011598266	3
BP	GO:0031670	cellular response to	3/74	0.003166952	0.011598266	3
BP	GO:0043367	CD4-positive, alpha	3/74	0.003166952	0.011598266	3
BP	GO:0046173	polyol biosynthetic		3/74	0.0032896	0.01199745	3
BP	GO:0150076	neuroinflammatory	3/74	0.0032896	0.01199745	3
BP	GO:0072503	cellular divalent inoı	7/74	0.003360083	0.012125269	7
BP	GO:0002052	positive regulation c	2/74	0.003400942	0.012125269	2
BP	GO:0006925	inflammatory cell ap	2/74	0.003400942	0.012125269	2
BP	GO:0010869	regulation of recept	2/74	0.003400942	0.012125269	2
BP	GO:0051000	positive regulation c	2/74	0.003400942	0.012125269	2
BP	GO:0060547	negative regulation	2/74	0.003400942	0.012125269	2
BP	GO:0090312	positive regulation c	2/74	0.003400942	0.012125269	2
BP	GO:1903589	positive regulation c	2/74	0.003400942	0.012125269	2
BP	GO:0000422	autophagy of mitoc	3/74	0.003415128	0.012125269	3
BP	GO:0042310	vasoconstriction	3/74	0.003415128	0.012125269	3
BP	GO:0045454	cell redox homeosta	3/74	0.003415128	0.012125269	3
BP	GO:0046902	regulation of mitocl	3/74	0.003415128	0.012125269	3
BP	GO:0061726	mitochondrion disa:	3/74	0.003415128	0.012125269	3
BP	GO:0071559	response to transfoı	5/74	0.003421214	0.012125269	5
BP	GO:0007611	learning or memory	5/74	0.003478755	0.012304398	5
BP	GO:0033673	negative regulation	5/74	0.003536991	0.012433527	5
BP	GO:0006110	regulation of glycol	3/74	0.003543555	0.012433527	3
BP	GO:0031397	negative regulation	3/74	0.003543555	0.012433527	3
BP	GO:0061418	regulation of transcı	3/74	0.003543555	0.012433527	3
BP	GO:0043406	positive regulation c	5/74	0.003595926	0.012592151	5
BP	GO:0008306	associative learning	3/74	0.003674904	0.012781091	3
BP	GO:0019400	alditol metabolic pro	2/74	0.003715318	0.012781091	2
BP	GO:0030810	positive regulation c	2/74	0.003715318	0.012781091	2
BP	GO:0045821	positive regulation c	2/74	0.003715318	0.012781091	2
BP	GO:0051882	mitochondrial depo	2/74	0.003715318	0.012781091	2
BP	GO:0071294	cellular response to	2/74	0.003715318	0.012781091	2
BP	GO:0072337	modified amino acio	2/74	0.003715318	0.012781091	2
BP	GO:1900373	positive regulation c	2/74	0.003715318	0.012781091	2
BP	GO:1902254	negative regulation	2/74	0.003715318	0.012781091	2
BP	GO:0010466	negative regulation	5/74	0.003838751	0.013179923	5
BP	GO:0009110	vitamin biosynthetic	2/74	0.004042715	0.0138262	2
BP	GO:0044346	fibroblast apoptotic	2/74	0.004042715	0.0138262	2
BP	GO:0048708	astrocyte differentia	3/74	0.004086682	0.013922397	3
BP	GO:2001021	negative regulation	3/74	0.004086682	0.013922397	3
BP	GO:0072331	signal transduction	5/74	0.004158577	0.014139925	5
BP	GO:1901653	cellular response to	6/74	0.004213402	0.014272072	6
BP	GO:0006094	gluconeogenesis	3/74	0.004229917	0.014272072	3

BP	GO:0090049	regulation of cell mi	3/74	0.004229917	0.014272072	3
BP	GO:0097581	lamellipodium orga	3/74	0.004229917	0.014272072	3
BP	GO:0048145	regulation of fibrob	3/74	0.004376173	0.014648099	3
BP	GO:0019430	removal of superoxi	2/74	0.004383027	0.014648099	2
BP	GO:0032104	regulation of respor	2/74	0.004383027	0.014648099	2
BP	GO:0032107	regulation of respor	2/74	0.004383027	0.014648099	2
BP	GO:0061050	regulation of cell gr	2/74	0.004383027	0.014648099	2
BP	GO:0010507	negative regulation	3/74	0.004525467	0.01500999	3
BP	GO:0046889	positive regulation c	3/74	0.004525467	0.01500999	3
BP	GO:0048144	fibroblast proliferati	3/74	0.004525467	0.01500999	3
BP	GO:0097756	negative regulation	3/74	0.004525467	0.01500999	3
BP	GO:0021543	pallium developmer	4/74	0.004557667	0.015059959	4
BP	GO:0030307	positive regulation c	4/74	0.004557667	0.015059959	4
BP	GO:0043542	endothelial cell mig	5/74	0.004637804	0.015296007	5
BP	GO:0019319	hexose biosynthetic	3/74	0.004677818	0.015399087	3
BP	GO:0032800	receptor biosynthet	2/74	0.00473615	0.015475193	2
BP	GO:1900739	regulation of protei	2/74	0.00473615	0.015475193	2
BP	GO:1900740	positive regulation c	2/74	0.00473615	0.015475193	2
BP	GO:1903203	regulation of oxidat	2/74	0.00473615	0.015475193	2
BP	GO:0070542	response to fatty ac	3/74	0.004833245	0.015763146	3
BP	GO:0001659	temperature homec	4/74	0.004949943	0.01611385	4
BP	GO:0043470	regulation of carbol	3/74	0.004991764	0.016219955	3
BP	GO:2001252	positive regulation c	4/74	0.005051444	0.016352206	4
BP	GO:0036475	neuron death in res	2/74	0.00510198	0.016352206	2
BP	GO:0046885	regulation of hormc	2/74	0.00510198	0.016352206	2
BP	GO:0048143	astrocyte activation	2/74	0.00510198	0.016352206	2
BP	GO:0071280	cellular response to	2/74	0.00510198	0.016352206	2
BP	GO:0071450	cellular response to	2/74	0.00510198	0.016352206	2
BP	GO:0071451	cellular response to	2/74	0.00510198	0.016352206	2
BP	GO:0006767	water-soluble vitam	3/74	0.005153393	0.016352206	3
BP	GO:0009060	aerobic respiration	3/74	0.005153393	0.016352206	3
BP	GO:0009791	post-embryonic dev	3/74	0.005153393	0.016352206	3
BP	GO:1900407	regulation of cellula	3/74	0.005153393	0.016352206	3
BP	GO:1903321	negative regulation	3/74	0.005153393	0.016352206	3
BP	GO:1904035	regulation of epithe	3/74	0.005153393	0.016352206	3
BP	GO:0022407	regulation of cell-ce	6/74	0.005189375	0.016436711	6
BP	GO:1903532	positive regulation c	6/74	0.005251544	0.016566497	6
BP	GO:0050796	regulation of insulin	4/74	0.005258623	0.016566497	4
BP	GO:0090316	positive regulation c	4/74	0.005258623	0.016566497	4
BP	GO:0055013	cardiac muscle cell c	3/74	0.005318149	0.016724054	3
BP	GO:0001959	regulation of cytoki	4/74	0.005364318	0.016839119	4
BP	GO:0034976	response to endopla	5/74	0.005468604	0.01694885	5
BP	GO:0051348	negative regulation	5/74	0.005468604	0.01694885	5
BP	GO:0009065	glutamine family am	2/74	0.005480413	0.01694885	2
BP	GO:0033137	negative regulation	2/74	0.005480413	0.01694885	2
BP	GO:0036296	response to increase	2/74	0.005480413	0.01694885	2
BP	GO:1902745	positive regulation c	2/74	0.005480413	0.01694885	2
BP	GO:0031058	positive regulation c	3/74	0.005486048	0.01694885	3
BP	GO:0032651	regulation of interle	3/74	0.005486048	0.01694885	3
BP	GO:0046849	bone remodeling	3/74	0.005486048	0.01694885	3
BP	GO:0010821	regulation of mitoch	4/74	0.005579964	0.017178615	4
BP	GO:1903034	regulation of respor	4/74	0.005579964	0.017178615	4
BP	GO:0046364	monosaccharide bic	3/74	0.005657107	0.017294955	3
BP	GO:0051899	membrane depolari	3/74	0.005657107	0.017294955	3
BP	GO:0060333	interferon-gamma-	3/74	0.005657107	0.017294955	3
BP	GO:1904063	negative regulation	3/74	0.005657107	0.017294955	3
BP	GO:0071346	cellular response to	4/74	0.005689931	0.017335008	4
BP	GO:1901796	regulation of signal	4/74	0.005689931	0.017335008	4

BP	GO:0002793	positive regulation c	5/74	0.005712356	0.01737322	5
BP	GO:0035710	CD4-positive, alpha	3/74	0.005831342	0.017612985	3
BP	GO:0000303	response to supero›	2/74	0.005871346	0.017612985	2
BP	GO:0009435	NAD biosynthetic pı	2/74	0.005871346	0.017612985	2
BP	GO:0010575	positive regulation c	2/74	0.005871346	0.017612985	2
BP	GO:0033032	regulation of myelo	2/74	0.005871346	0.017612985	2
BP	GO:0044550	secondary metaboli	2/74	0.005871346	0.017612985	2
BP	GO:0070229	negative regulation	2/74	0.005871346	0.017612985	2
BP	GO:2000727	positive regulation c	2/74	0.005871346	0.017612985	2
BP	GO:0008217	regulation of blood	4/74	0.005914195	0.017711297	4
BP	GO:0033273	response to vitamin	3/74	0.006008768	0.01790302	3
BP	GO:0035249	synaptic transmissio	3/74	0.006008768	0.01790302	3
BP	GO:0036473	cell death in respon	3/74	0.006008768	0.01790302	3
BP	GO:0003015	heart process	5/74	0.006049192	0.017962572	5
BP	GO:0090287	regulation of cellula	5/74	0.006049192	0.017962572	5
BP	GO:0030099	myeloid cell differer	6/74	0.006110523	0.01811409	6
BP	GO:0051146	striated muscle cell	5/74	0.006135544	0.018157644	5
BP	GO:0000305	response to oxygen	2/74	0.006274678	0.018414396	2
BP	GO:0001782	B cell homeostasis	2/74	0.006274678	0.018414396	2
BP	GO:0001844	protein insertion int	2/74	0.006274678	0.018414396	2
BP	GO:0043372	positive regulation c	2/74	0.006274678	0.018414396	2
BP	GO:0090200	positive regulation c	2/74	0.006274678	0.018414396	2
BP	GO:0048511	rhythmic process	5/74	0.006310853	0.018489692	5
BP	GO:0055006	cardiac cell develop	3/74	0.006373254	0.018641448	3
BP	GO:0090277	positive regulation c	3/74	0.006560344	0.019156801	3
BP	GO:0001963	synaptic transmissio	2/74	0.006690306	0.019375375	2
BP	GO:0006691	leukotriene metabol	2/74	0.006690306	0.019375375	2
BP	GO:0044319	wound healing, spre	2/74	0.006690306	0.019375375	2
BP	GO:0090505	epiboly involved in	2/74	0.006690306	0.019375375	2
BP	GO:1902253	regulation of intrins	2/74	0.006690306	0.019375375	2
BP	GO:0120162	positive regulation c	3/74	0.006750684	0.01948603	3
BP	GO:1902882	regulation of respor	3/74	0.006750684	0.01948603	3
BP	GO:0070372	regulation of ERK1 a	5/74	0.006764525	0.019493972	5
BP	GO:0060759	regulation of respor	4/74	0.006870176	0.019766034	4
BP	GO:0002042	cell migration involv	3/74	0.006944288	0.019914075	3
BP	GO:0045807	positive regulation c	3/74	0.006944288	0.019914075	3
BP	GO:0019359	nicotinamide nuclec	2/74	0.007118128	0.020092313	2
BP	GO:0019363	pyridine nucleotide	2/74	0.007118128	0.020092313	2
BP	GO:0046685	response to arsenic-	2/74	0.007118128	0.020092313	2
BP	GO:0046949	fatty-acyl-CoA bios	2/74	0.007118128	0.020092313	2
BP	GO:0051968	positive regulation c	2/74	0.007118128	0.020092313	2
BP	GO:0090504	epiboly	2/74	0.007118128	0.020092313	2
BP	GO:1901661	quinone metabolic	2/74	0.007118128	0.020092313	2
BP	GO:0006164	purine nucleotide bi	4/74	0.007124224	0.020092313	4
BP	GO:0007565	female pregnancy	4/74	0.007124224	0.020092313	4
BP	GO:0043648	dicarboxylic acid me	3/74	0.00714117	0.020092313	3
BP	GO:0070301	cellular response to	3/74	0.00714117	0.020092313	3
BP	GO:0046034	ATP metabolic proc	5/74	0.007143599	0.020092313	5
BP	GO:0007219	Notch signaling pat	4/74	0.007253549	0.020368971	4
BP	GO:0022600	digestive system prc	3/74	0.007341344	0.020582631	3
BP	GO:0042391	regulation of memb	6/74	0.007463957	0.020695092	6
BP	GO:0032611	interleukin-1 beta p	3/74	0.007544821	0.020695092	3
BP	GO:0046632	alpha-beta T cell dif	3/74	0.007544821	0.020695092	3
BP	GO:0048259	regulation of recept	3/74	0.007544821	0.020695092	3
BP	GO:1903008	organelle disassemt	3/74	0.007544821	0.020695092	3
BP	GO:0016242	negative regulation	2/74	0.007558046	0.020695092	2
BP	GO:0019362	pyridine nucleotide	2/74	0.007558046	0.020695092	2
BP	GO:0032691	negative regulation	2/74	0.007558046	0.020695092	2

BP	GO:0033028	myeloid cell apopto	2/74	0.007558046	0.020695092	2
BP	GO:0042755	eating behavior	2/74	0.007558046	0.020695092	2
BP	GO:0045736	negative regulation	2/74	0.007558046	0.020695092	2
BP	GO:0046496	nicotinamide nuclec	2/74	0.007558046	0.020695092	2
BP	GO:0070232	regulation of T cell a	2/74	0.007558046	0.020695092	2
BP	GO:1901797	negative regulation	2/74	0.007558046	0.020695092	2
BP	GO:1902692	regulation of neurol	2/74	0.007558046	0.020695092	2
BP	GO:0000079	regulation of cyclin-	3/74	0.007751616	0.021192106	3
BP	GO:0010952	positive regulation c	4/74	0.007786368	0.021254061	4
BP	GO:0016052	carbohydrate catab	4/74	0.00792349	0.021431756	4
BP	GO:0031099	regeneration	4/74	0.00792349	0.021431756	4
BP	GO:0018958	phenol-containing c	3/74	0.007961739	0.021431756	3
BP	GO:0032652	regulation of interle	3/74	0.007961739	0.021431756	3
BP	GO:1905269	positive regulation c	3/74	0.007961739	0.021431756	3
BP	GO:0006536	glutamate metaboli	2/74	0.008009958	0.021431756	2
BP	GO:0010574	regulation of vascul	2/74	0.008009958	0.021431756	2
BP	GO:0010922	positive regulation c	2/74	0.008009958	0.021431756	2
BP	GO:0032148	activation of protein	2/74	0.008009958	0.021431756	2
BP	GO:0042533	tumor necrosis facto	2/74	0.008009958	0.021431756	2
BP	GO:0042534	regulation of tumor	2/74	0.008009958	0.021431756	2
BP	GO:0043276	anoikis	2/74	0.008009958	0.021431756	2
BP	GO:1904030	negative regulation	2/74	0.008009958	0.021431756	2
BP	GO:0006109	regulation of carbol	4/74	0.008062194	0.021506053	4
BP	GO:0007179	transforming growtl	4/74	0.008062194	0.021506053	4
BP	GO:0051235	maintenance of loca	5/74	0.00826245	0.022006845	5
BP	GO:0006637	acyl-CoA metabolic	3/74	0.008392023	0.022217309	3
BP	GO:0006641	triglyceride metabol	3/74	0.008392023	0.022217309	3
BP	GO:0008593	regulation of Notch	3/74	0.008392023	0.022217309	3
BP	GO:0035383	thioester metabolic	3/74	0.008392023	0.022217309	3
BP	GO:0051354	negative regulation	2/74	0.008473766	0.022303117	2
BP	GO:0060251	regulation of glial ce	2/74	0.008473766	0.022303117	2
BP	GO:1901030	positive regulation c	2/74	0.008473766	0.022303117	2
BP	GO:0070371	ERK1 and ERK2 casc	5/74	0.008478238	0.022303117	5
BP	GO:0009746	response to hexose	4/74	0.008487872	0.022303117	4
BP	GO:0002286	T cell activation invc	3/74	0.008612206	0.022562373	3
BP	GO:1904029	regulation of cyclin-	3/74	0.008612206	0.022562373	3
BP	GO:0002573	myeloid leukocyte c	4/74	0.008779701	0.022898798	4
BP	GO:0006006	glucose metabolic p	4/74	0.008779701	0.022898798	4
BP	GO:0031396	regulation of protei	4/74	0.008779701	0.022898798	4
BP	GO:0030330	DNA damage respo	3/74	0.008835766	0.022966499	3
BP	GO:0071222	cellular response to	4/74	0.00892805	0.022966499	4
BP	GO:0072522	purine-containing c	4/74	0.00892805	0.022966499	4
BP	GO:0006882	cellular zinc ion hon	2/74	0.008949371	0.022966499	2
BP	GO:0010573	vascular endothelial	2/74	0.008949371	0.022966499	2
BP	GO:0030224	monocyte differenti	2/74	0.008949371	0.022966499	2
BP	GO:0034390	smooth muscle cell	2/74	0.008949371	0.022966499	2
BP	GO:0034391	regulation of smoot	2/74	0.008949371	0.022966499	2
BP	GO:0090322	regulation of superc	2/74	0.008949371	0.022966499	2
BP	GO:1903131	mononuclear cell di	2/74	0.008949371	0.022966499	2
BP	GO:2000516	positive regulation c	2/74	0.008949371	0.022966499	2
BP	GO:0002526	acute inflammatory	3/74	0.009062712	0.02322346	3
BP	GO:0009615	response to virus	5/74	0.009149073	0.023410637	5
BP	GO:0030073	insulin secretion	4/74	0.009229652	0.023548268	4
BP	GO:0034284	response to monosa	4/74	0.009229652	0.023548268	4
BP	GO:1903829	positive regulation c	5/74	0.009264347	0.023602531	5
BP	GO:0051149	positive regulation c	3/74	0.009293055	0.023641408	3
BP	GO:1901215	negative regulation	4/74	0.009382919	0.02383431	4
BP	GO:0042307	positive regulation c	2/74	0.009436674	0.02383431	2

BP	GO:0045730	respiratory burst	2/74	0.009436674	0.02383431	2
BP	GO:0051193	regulation of cofact	2/74	0.009436674	0.02383431	2
BP	GO:0071542	dopaminergic neuro	2/74	0.009436674	0.02383431	2
BP	GO:0043523	regulation of neuro	4/74	0.009694421	0.024415144	4
BP	GO:0050707	regulation of cytoki	4/74	0.009694421	0.024415144	4
BP	GO:0031623	receptor internalizat	3/74	0.009763977	0.02455514	3
BP	GO:0003298	physiological muscl	2/74	0.009935578	0.024738952	2
BP	GO:0003301	physiological cardia	2/74	0.009935578	0.024738952	2
BP	GO:0010831	positive regulation c	2/74	0.009935578	0.024738952	2
BP	GO:0032094	response to food	2/74	0.009935578	0.024738952	2
BP	GO:0032350	regulation of hormo	2/74	0.009935578	0.024738952	2
BP	GO:0055069	zinc ion homeostasi	2/74	0.009935578	0.024738952	2
BP	GO:0061049	cell growth involvec	2/74	0.009935578	0.024738952	2
BP	GO:0042303	molting cycle	3/74	0.010004574	0.024790251	3
BP	GO:0042633	hair cycle	3/74	0.010004574	0.024790251	3
BP	GO:0071219	cellular response to	4/74	0.01001259	0.024790251	4
BP	GO:1903531	negative regulation	4/74	0.01001259	0.024790251	4
BP	GO:0008202	steroid metabolic pr	5/74	0.010099548	0.024970384	5
BP	GO:0006096	glycolytic process	3/74	0.010248609	0.025303336	3
BP	GO:0045732	positive regulation c	4/74	0.01033748	0.025486958	4
BP	GO:0008207	C21-steroid hormor	2/74	0.010445986	0.025500307	2
BP	GO:0032692	negative regulation	2/74	0.010445986	0.025500307	2
BP	GO:0042417	dopamine metaboli	2/74	0.010445986	0.025500307	2
BP	GO:0051180	vitamin transport	2/74	0.010445986	0.025500307	2
BP	GO:1902042	negative regulation	2/74	0.010445986	0.025500307	2
BP	GO:1904591	positive regulation c	2/74	0.010445986	0.025500307	2
BP	GO:2000648	positive regulation c	2/74	0.010445986	0.025500307	2
BP	GO:0006757	ATP generation fror	3/74	0.01049609	0.025500307	3
BP	GO:0042035	regulation of cytoki	3/74	0.01049609	0.025500307	3
BP	GO:0042752	regulation of circadi	3/74	0.01049609	0.025500307	3
BP	GO:0070374	positive regulation c	4/74	0.010502462	0.025500307	4
BP	GO:0032869	cellular response to	4/74	0.010669144	0.025869286	4
BP	GO:0032612	interleukin-1 produ	3/74	0.010747026	0.025986438	3
BP	GO:0032963	collagen metabolic	3/74	0.010747026	0.025986438	3
BP	GO:0044242	cellular lipid catabol	4/74	0.010837533	0.026169288	4
BP	GO:0045124	regulation of bone r	2/74	0.010967801	0.026375157	2
BP	GO:0071470	cellular response to	2/74	0.010967801	0.026375157	2
BP	GO:1900371	regulation of purine	2/74	0.010967801	0.026375157	2
BP	GO:0021782	glial cell developme	3/74	0.011001427	0.026381157	3
BP	GO:1903039	positive regulation c	4/74	0.011007634	0.026381157	4
BP	GO:0003012	muscle system proc	6/74	0.011015318	0.026381157	6
BP	GO:0002699	positive regulation c	4/74	0.011179455	0.026737829	4
BP	GO:0051153	regulation of striate	3/74	0.0112593	0.026892207	3
BP	GO:0019432	triglyceride biosynth	2/74	0.011500928	0.027247197	2
BP	GO:0030808	regulation of nuclec	2/74	0.011500928	0.027247197	2
BP	GO:0035337	fatty-acyl-CoA meta	2/74	0.011500928	0.027247197	2
BP	GO:0045616	regulation of keratir	2/74	0.011500928	0.027247197	2
BP	GO:0140353	lipid export from ce	2/74	0.011500928	0.027247197	2
BP	GO:1905209	positive regulation c	2/74	0.011500928	0.027247197	2
BP	GO:0044706	multi-multicellular c	4/74	0.011705294	0.027694045	4
BP	GO:0045444	fat cell differentiatio	4/74	0.011884054	0.028079189	4
BP	GO:0010613	positive regulation c	2/74	0.01204527	0.028345807	2
BP	GO:0046688	response to copper	2/74	0.01204527	0.028345807	2
BP	GO:1903146	regulation of autopl	2/74	0.01204527	0.028345807	2
BP	GO:0048638	regulation of develc	5/74	0.012200469	0.028672648	5
BP	GO:0034765	regulation of ion tra	6/74	0.012235619	0.028716863	6
BP	GO:0006692	prostanoid metabol	2/74	0.012600735	0.029108131	2
BP	GO:0006693	prostaglandin metal	2/74	0.012600735	0.029108131	2

BP	GO:0008631	intrinsic apoptotic si	2/74	0.012600735	0.029108131	2
BP	GO:0014742	positive regulation c	2/74	0.012600735	0.029108131	2
BP	GO:0032459	regulation of protei	2/74	0.012600735	0.029108131	2
BP	GO:0034198	cellular response to	2/74	0.012600735	0.029108131	2
BP	GO:0045746	negative regulation	2/74	0.012600735	0.029108131	2
BP	GO:0046460	neutral lipid biosynt	2/74	0.012600735	0.029108131	2
BP	GO:0046463	acylglycerol biosynt	2/74	0.012600735	0.029108131	2
BP	GO:0072348	sulfur compound tra	2/74	0.012600735	0.029108131	2
BP	GO:0034101	erythrocyte homeos	3/74	0.012601032	0.029108131	3
BP	GO:0046031	ADP metabolic proc	3/74	0.012601032	0.029108131	3
BP	GO:0042089	cytokine biosyntheti	3/74	0.012879903	0.029713273	3
BP	GO:0032388	positive regulation c	4/74	0.012993572	0.029936214	4
BP	GO:0008637	apoptotic mitochon	3/74	0.013162298	0.030099606	3
BP	GO:0042107	cytokine metabolic		3/74	0.013162298	0.030099606	3
BP	GO:0032965	regulation of collag	2/74	0.013167226	0.030099606	2
BP	GO:0046189	phenol-containing c	2/74	0.013167226	0.030099606	2
BP	GO:0090311	regulation of protei	2/74	0.013167226	0.030099606	2
BP	GO:1901028	regulation of mitoch	2/74	0.013167226	0.030099606	2
BP	GO:1903320	regulation of protei	4/74	0.013377631	0.030540866	4
BP	GO:0031929	TOR signaling	3/74	0.013448224	0.03062249	3
BP	GO:2000134	negative regulation	3/74	0.013448224	0.03062249	3
BP	GO:0032309	icosanoid secretion	2/74	0.013744651	0.031216601	2
BP	GO:0051204	protein insertion int	2/74	0.013744651	0.031216601	2
BP	GO:0042445	hormone metabolic	4/74	0.013768876	0.031231269	4
BP	GO:0048588	developmental cell	4/74	0.013967206	0.031640307	4
BP	GO:0046777	protein autophosph	4/74	0.01416735	0.032033206	4
BP	GO:0050900	leukocyte migration	6/74	0.014177091	0.032033206	6
BP	GO:0002576	platelet degranulati	3/74	0.014327245	0.032055671	3
BP	GO:0001504	neurotransmitter up	2/74	0.014332916	0.032055671	2
BP	GO:0045776	negative regulation	2/74	0.014332916	0.032055671	2
BP	GO:0050706	regulation of interle	2/74	0.014332916	0.032055671	2
BP	GO:0051646	mitochondrion loca	2/74	0.014332916	0.032055671	2
BP	GO:0060443	mammary gland mo	2/74	0.014332916	0.032055671	2
BP	GO:1903587	regulation of blood	2/74	0.014332916	0.032055671	2
BP	GO:1990928	response to amino a	2/74	0.014332916	0.032055671	2
BP	GO:0071216	cellular response to	4/74	0.014369312	0.032096237	4
BP	GO:0006639	acylglycerol metabo	3/74	0.014627352	0.032631149	3
BP	GO:0051048	negative regulation	4/74	0.014778712	0.032927022	4
BP	GO:0006638	neutral lipid metabo	3/74	0.014931018	0.032934454	3
BP	GO:0043500	muscle adaptation	3/74	0.014931018	0.032934454	3
BP	GO:0031952	regulation of protei	2/74	0.014931929	0.032934454	2
BP	GO:0043370	regulation of CD4-p	2/74	0.014931929	0.032934454	2
BP	GO:0046638	positive regulation c	2/74	0.014931929	0.032934454	2
BP	GO:0070231	T cell apoptotic pro	2/74	0.014931929	0.032934454	2
BP	GO:0090151	establishment of pro	2/74	0.014931929	0.032934454	2
BP	GO:2000107	negative regulation	2/74	0.014931929	0.032934454	2
BP	GO:1902807	negative regulation	3/74	0.015238248	0.033567964	3
BP	GO:0030850	prostate gland deve	2/74	0.015541598	0.033870614	2
BP	GO:0045806	negative regulation	2/74	0.015541598	0.033870614	2
BP	GO:0046850	regulation of bone r	2/74	0.015541598	0.033870614	2
BP	GO:0055023	positive regulation c	2/74	0.015541598	0.033870614	2
BP	GO:0090199	regulation of release	2/74	0.015541598	0.033870614	2
BP	GO:0006165	nucleoside diphospl	3/74	0.015549046	0.033870614	3
BP	GO:0042177	negative regulation	3/74	0.015549046	0.033870614	3
BP	GO:0043280	positive regulation c	3/74	0.015549046	0.033870614	3
BP	GO:0045598	regulation of fat cell	3/74	0.015549046	0.033870614	3
BP	GO:0032386	regulation of intrace	5/74	0.015715024	0.0341898	5
BP	GO:0071333	cellular response to	3/74	0.015863416	0.034469983	3

BP	GO:0019318	hexose metabolic pı	4/74	0.016051091	0.034689189	4
BP	GO:0031334	positive regulation (	4/74	0.016051091	0.034689189	4
BP	GO:0003254	regulation of memb	2/74	0.016161831	0.034689189	2
BP	GO:0010712	regulation of collag	2/74	0.016161831	0.034689189	2
BP	GO:0035094	response to nicotine	2/74	0.016161831	0.034689189	2
BP	GO:0071715	icosanoid transport	2/74	0.016161831	0.034689189	2
BP	GO:1901571	fatty acid derivative	2/74	0.016161831	0.034689189	2
BP	GO:0009135	purine nucleoside d	3/74	0.016181364	0.034689189	3
BP	GO:0009179	purine ribonucleosi	3/74	0.016181364	0.034689189	3
BP	GO:0046939	nucleotide phosphc	3/74	0.016181364	0.034689189	3
BP	GO:0048565	digestive tract devel	3/74	0.016181364	0.034689189	3
BP	GO:0071331	cellular response to	3/74	0.016502893	0.035335382	3
BP	GO:1903362	regulation of cellula	4/74	0.016712377	0.03574039	4
BP	GO:0014009	glial cell proliferatio	2/74	0.016792537	0.035813396	2
BP	GO:2000725	regulation of cardia	2/74	0.016792537	0.035813396	2
BP	GO:0009185	ribonucleoside diph	3/74	0.016828007	0.035813396	3
BP	GO:0071326	cellular response to	3/74	0.016828007	0.035813396	3
BP	GO:0071383	cellular response to	4/74	0.016936556	0.036000826	4
BP	GO:0034605	cellular response to	3/74	0.017156709	0.036380807	3
BP	GO:1903670	regulation of sprout	3/74	0.017156709	0.036380807	3
BP	GO:0033157	regulation of intrace	4/74	0.017390567	0.03665847	4
BP	GO:0006733	oxidoreduction coeı	2/74	0.017433627	0.03665847	2
BP	GO:0032007	negative regulation	2/74	0.017433627	0.03665847	2
BP	GO:0048260	positive regulation (	2/74	0.017433627	0.03665847	2
BP	GO:0051496	positive regulation (	2/74	0.017433627	0.03665847	2
BP	GO:0097366	response to bronch	2/74	0.017433627	0.03665847	2
BP	GO:2001258	negative regulation	2/74	0.017433627	0.03665847	2
BP	GO:0046631	alpha-beta T cell ac	3/74	0.017489003	0.036730975	3
BP	GO:0030900	forebrain developm	5/74	0.017613431	0.03694816	5
BP	GO:0031529	ruffle organization	2/74	0.018085009	0.037654558	2
BP	GO:0035176	social behavior	2/74	0.018085009	0.037654558	2
BP	GO:0060421	positive regulation (	2/74	0.018085009	0.037654558	2
BP	GO:2000179	positive regulation (	2/74	0.018085009	0.037654558	2
BP	GO:0031330	negative regulation	4/74	0.018085783	0.037654558	4
BP	GO:0033865	nucleoside bisphos	3/74	0.018164378	0.037654558	3
BP	GO:0033875	ribonucleoside bispl	3/74	0.018164378	0.037654558	3
BP	GO:0034032	purine nucleoside b	3/74	0.018164378	0.037654558	3
BP	GO:0060048	cardiac muscle cont	3/74	0.018164378	0.037654558	3
BP	GO:1903364	positive regulation (	3/74	0.018164378	0.037654558	3
BP	GO:0051924	regulation of calciur	4/74	0.018321325	0.037929061	4
BP	GO:0042692	muscle cell different	5/74	0.01833995	0.037929061	5
BP	GO:0009411	response to UV	3/74	0.018507465	0.038185653	3
BP	GO:0050709	negative regulation	3/74	0.018507465	0.038185653	3
BP	GO:0022409	positive regulation (	4/74	0.018558777	0.038246632	4
BP	GO:0002043	blood vessel endoth	2/74	0.018746596	0.038408557	2
BP	GO:0032964	collagen biosynthet	2/74	0.018746596	0.038408557	2
BP	GO:0050702	interleukin-1 beta s	2/74	0.018746596	0.038408557	2
BP	GO:0050704	regulation of interle	2/74	0.018746596	0.038408557	2
BP	GO:0070228	regulation of lymph	2/74	0.018746596	0.038408557	2
BP	GO:0006606	protein import into	3/74	0.019204451	0.03916404	3
BP	GO:0031056	regulation of histon	3/74	0.019204451	0.03916404	3
BP	GO:0106106	cold-induced therm	3/74	0.019204451	0.03916404	3
BP	GO:0120161	regulation of cold-i	3/74	0.019204451	0.03916404	3
BP	GO:0006584	catecholamine meta	2/74	0.019418297	0.039326408	2
BP	GO:0009712	catechol-containing	2/74	0.019418297	0.039326408	2
BP	GO:0035384	thioester biosynthet	2/74	0.019418297	0.039326408	2
BP	GO:0046164	alcohol catabolic pr	2/74	0.019418297	0.039326408	2
BP	GO:0051703	intraspecies interact	2/74	0.019418297	0.039326408	2

BP	GO:0071616	acyl-CoA biosynthe	2/74	0.019418297	0.039326408	2
BP	GO:0090257	regulation of muscl	4/74	0.019527761	0.039502587	4
BP	GO:0071322	cellular response to	3/74	0.019558353	0.039518995	3
BP	GO:0071901	negative regulation	3/74	0.019915865	0.04019517	3
BP	GO:0002011	morphogenesis of a	2/74	0.020100025	0.040335306	2
BP	GO:0002090	regulation of recept	2/74	0.020100025	0.040335306	2
BP	GO:0042220	response to cocaine	2/74	0.020100025	0.040335306	2
BP	GO:0042304	regulation of fatty a	2/74	0.020100025	0.040335306	2
BP	GO:0042306	regulation of protei	2/74	0.020100025	0.040335306	2
BP	GO:0015850	organic hydroxy cor	4/74	0.020274746	0.040597732	4
BP	GO:0055123	digestive system de	3/74	0.020276987	0.040597732	3
BP	GO:0010212	response to ionizing	3/74	0.020641721	0.041234061	3
BP	GO:0043524	negative regulation	3/74	0.020641721	0.041234061	3
BP	GO:0006749	glutathione metabo	2/74	0.020791692	0.04129899	2
BP	GO:0006977	DNA damage respo	2/74	0.020791692	0.04129899	2
BP	GO:0010332	response to gamma	2/74	0.020791692	0.04129899	2
BP	GO:1903078	positive regulation c	2/74	0.020791692	0.04129899	2
BP	GO:1903202	negative regulation	2/74	0.020791692	0.04129899	2
BP	GO:0002792	negative regulation	3/74	0.021010069	0.041685655	3
BP	GO:0014074	response to purine-	3/74	0.021382032	0.042280497	3
BP	GO:2000058	regulation of ubiqui	3/74	0.021382032	0.042280497	3
BP	GO:2001056	positive regulation c	3/74	0.021382032	0.042280497	3
BP	GO:0072431	signal transduction	2/74	0.021493211	0.042357401	2
BP	GO:1900408	negative regulation	2/74	0.021493211	0.042357401	2
BP	GO:1902400	intracellular signal t	2/74	0.021493211	0.042357401	2
BP	GO:0050714	positive regulation c	4/74	0.021821229	0.042955682	4
BP	GO:0045785	positive regulation c	5/74	0.021854925	0.04297389	5
BP	GO:0055088	lipid homeostasis	3/74	0.022136804	0.043274017	3
BP	GO:0017001	antibiotic catabolic		2/74	0.022204494	0.043274017	2
BP	GO:0035306	positive regulation c	2/74	0.022204494	0.043274017	2
BP	GO:0042093	T-helper cell differe	2/74	0.022204494	0.043274017	2
BP	GO:0055081	anion homeostasis	2/74	0.022204494	0.043274017	2
BP	GO:0061900	glial cell activation	2/74	0.022204494	0.043274017	2
BP	GO:1903749	positive regulation c	2/74	0.022204494	0.043274017	2
BP	GO:1904589	regulation of protei	2/74	0.022204494	0.043274017	2
BP	GO:0045927	positive regulation c	4/74	0.022352389	0.043514005	4
BP	GO:0001678	cellular glucose hon	3/74	0.022519615	0.043742665	3
BP	GO:0001890	placenta developme	3/74	0.022519615	0.043742665	3
BP	GO:0001836	release of cytochror	2/74	0.022925457	0.044189188	2
BP	GO:0010656	negative regulation	2/74	0.022925457	0.044189188	2
BP	GO:0070527	platelet aggregatior	2/74	0.022925457	0.044189188	2
BP	GO:0072413	signal transduction	2/74	0.022925457	0.044189188	2
BP	GO:1902402	signal transduction	2/74	0.022925457	0.044189188	2
BP	GO:1902403	signal transduction	2/74	0.022925457	0.044189188	2
BP	GO:1902883	negative regulation	2/74	0.022925457	0.044189188	2
BP	GO:0009132	nucleoside diphospl	3/74	0.023296087	0.044805326	3
BP	GO:1990845	adaptive thermoger	3/74	0.023296087	0.044805326	3
BP	GO:0007517	muscle organ devel	5/74	0.023332875	0.044827037	5
BP	GO:0032507	maintenance of pro	2/74	0.023656012	0.045398231	2
BP	GO:0046486	glycerolipid metabo	5/74	0.024205912	0.046402886	5
BP	GO:0007405	neuroblast prolifera	2/74	0.024396076	0.046463416	2
BP	GO:0032233	positive regulation c	2/74	0.024396076	0.046463416	2
BP	GO:0045604	regulation of epider	2/74	0.024396076	0.046463416	2
BP	GO:0050701	interleukin-1 secreti	2/74	0.024396076	0.046463416	2
BP	GO:2000514	regulation of CD4-p	2/74	0.024396076	0.046463416	2
BP	GO:2001244	positive regulation c	2/74	0.024396076	0.046463416	2
BP	GO:0030522	intracellular recepto	4/74	0.024556066	0.04671751	4
BP	GO:0052126	movement in host e	3/74	0.024892429	0.047306238	3

BP	GO:0010830	regulation of myotu	2/74	0.025145562	0.047581541	2
BP	GO:0046635	positive regulation c	2/74	0.025145562	0.047581541	2
BP	GO:0046824	positive regulation c	2/74	0.025145562	0.047581541	2
BP	GO:0055025	positive regulation c	2/74	0.025145562	0.047581541	2
BP	GO:0043588	skin development	5/74	0.025326657	0.047872685	5
BP	GO:0060047	heart contraction	4/74	0.02570567	0.048445339	4
BP	GO:0007519	skeletal muscle tissu	3/74	0.025712291	0.048445339	3
BP	GO:0032680	regulation of tumor	3/74	0.025712291	0.048445339	3
BP	GO:0031571	mitotic G1 DNA dar	2/74	0.025904388	0.048547109	2
BP	GO:0044819	mitotic G1/S transiti	2/74	0.025904388	0.048547109	2
BP	GO:0046622	positive regulation c	2/74	0.025904388	0.048547109	2
BP	GO:0046637	regulation of alpha-	2/74	0.025904388	0.048547109	2
BP	GO:1904377	positive regulation c	2/74	0.025904388	0.048547109	2
BP	GO:0044783	G1 DNA damage ch	2/74	0.02667247	0.049827199	2
BP	GO:0045670	regulation of osteoc	2/74	0.02667247	0.049827199	2
BP	GO:0060135	maternal process in	2/74	0.02667247	0.049827199	2
BP	GO:0032640	tumor necrosis facto	3/74	0.026969174	0.050168222	3
BP	GO:0051170	import into nucleus	3/74	0.026969174	0.050168222	3
BP	GO:1902600	proton transmembr	3/74	0.026969174	0.050168222	3
BP	GO:1903555	regulation of tumor	3/74	0.026969174	0.050168222	3
BP	GO:0006940	regulation of smoot	2/74	0.027449724	0.050954303	2
BP	GO:0014823	response to activity	2/74	0.027449724	0.050954303	2
BP	GO:0043254	regulation of protei	5/74	0.027667448	0.051304284	5
BP	GO:0007162	negative regulation	4/74	0.027794772	0.051486074	4
BP	GO:0051926	negative regulation	2/74	0.028236067	0.052248456	2
BP	GO:0051090	regulation of DNA-l	5/74	0.028395771	0.052488724	5
BP	GO:0015807	L-amino acid transp	2/74	0.029031418	0.053607328	2
BP	GO:0001906	cell killing	3/74	0.029136112	0.053631643	3
BP	GO:0050806	positive regulation c	3/74	0.029136112	0.053631643	3
BP	GO:0071706	tumor necrosis facto	3/74	0.029136112	0.053631643	3
BP	GO:0050804	modulation of chem	5/74	0.029385768	0.05403461	5
BP	GO:0060538	skeletal muscle orga	3/74	0.029580301	0.054335482	3
BP	GO:0099177	regulation of trans-:	5/74	0.029636656	0.054382173	5
BP	GO:0031343	positive regulation c	2/74	0.029835695	0.05440665	2
BP	GO:0033866	nucleoside bisphosp	2/74	0.029835695	0.05440665	2
BP	GO:0034030	ribonucleoside bispl	2/74	0.029835695	0.05440665	2
BP	GO:0034033	purine nucleoside b	2/74	0.029835695	0.05440665	2
BP	GO:0035924	cellular response to	2/74	0.029835695	0.05440665	2
BP	GO:1903317	regulation of protei	2/74	0.029835695	0.05440665	2
BP	GO:0001818	negative regulation	4/74	0.029982952	0.054618521	4
BP	GO:0043433	negative regulation	3/74	0.030028085	0.054644112	3
BP	GO:1903522	regulation of blood	4/74	0.030303679	0.055088602	4
BP	GO:0015909	long-chain fatty aci	2/74	0.030648816	0.055601024	2
BP	GO:0070227	lymphocyte apopto	2/74	0.030648816	0.055601024	2
BP	GO:0009152	purine ribonucleotic	3/74	0.030934424	0.056003564	3
BP	GO:0030324	lung development	3/74	0.030934424	0.056003564	3
BP	GO:0055002	striated muscle cell	3/74	0.031392973	0.056682562	3
BP	GO:1901990	regulation of mitoti	5/74	0.031431044	0.056682562	5
BP	GO:0034121	regulation of toll-lik	2/74	0.031470702	0.056682562	2
BP	GO:0051966	regulation of synapt	2/74	0.031470702	0.056682562	2
BP	GO:0072091	regulation of stem c	2/74	0.031470702	0.056682562	2
BP	GO:0042440	pigment metabolic	2/74	0.032301271	0.058059544	2
BP	GO:0071479	cellular response to	2/74	0.032301271	0.058059544	2
BP	GO:1903037	regulation of leukoc	4/74	0.032605939	0.058547302	4
BP	GO:0009408	response to heat	3/74	0.032790103	0.058757951	3
BP	GO:0030323	respiratory tube dev	3/74	0.032790103	0.058757951	3
BP	GO:0002532	production of mole	2/74	0.033140444	0.059204687	2
BP	GO:0061180	mammary gland epi	2/74	0.033140444	0.059204687	2

BP	GO:1903747	regulation of establi	2/74	0.033140444	0.059204687	2
BP	GO:0006941	striated muscle cont	3/74	0.033262962	0.059303027	3
BP	GO:0048469	cell maturation	3/74	0.033262962	0.059303027	3
BP	GO:0010950	positive regulation c	3/74	0.033739388	0.060091482	3
BP	GO:0033555	multicellular organis	2/74	0.033988142	0.060290188	2
BP	GO:0043627	response to estroge	2/74	0.033988142	0.060290188	2
BP	GO:0072401	signal transduction	2/74	0.033988142	0.060290188	2
BP	GO:0072422	signal transduction	2/74	0.033988142	0.060290188	2
BP	GO:0008361	regulation of cell siz	3/74	0.034219378	0.060517168	3
BP	GO:0030177	positive regulation c	3/74	0.034219378	0.060517168	3
BP	GO:0051099	positive regulation c	3/74	0.034219378	0.060517168	3
BP	GO:1905330	regulation of morph	3/74	0.034702928	0.061130327	3
BP	GO:2001257	regulation of cation	3/74	0.034702928	0.061130327	3
BP	GO:0001895	retina homeostasis	2/74	0.034844286	0.061130327	2
BP	GO:0014015	positive regulation c	2/74	0.034844286	0.061130327	2
BP	GO:0050710	negative regulation	2/74	0.034844286	0.061130327	2
BP	GO:0072395	signal transduction	2/74	0.034844286	0.061130327	2
BP	GO:1900182	positive regulation c	2/74	0.034844286	0.061130327	2
BP	GO:1901616	organic hydroxy cor	2/74	0.034844286	0.061130327	2
BP	GO:0002285	lymphocyte activatio	3/74	0.035190033	0.06160886	3
BP	GO:0051147	regulation of muscle	3/74	0.035190033	0.06160886	3
BP	GO:0006874	cellular calcium ion	5/74	0.035222191	0.06160886	5
BP	GO:0034612	response to tumor r	4/74	0.035360078	0.061788563	4
BP	GO:0061138	morphogenesis of a	3/74	0.03568069	0.062150801	3
BP	GO:0002479	antigen processing .	2/74	0.035708798	0.062150801	2
BP	GO:0010611	regulation of cardia	2/74	0.035708798	0.062150801	2
BP	GO:1903201	regulation of oxidat	2/74	0.035708798	0.062150801	2
BP	GO:0002040	sprouting angiogen	3/74	0.036174893	0.062899757	3
BP	GO:0002697	regulation of immur	5/74	0.036355438	0.063151218	5
BP	GO:0051346	negative regulation	5/74	0.03664225	0.063513928	5
BP	GO:0043409	negative regulation	3/74	0.036672638	0.063513928	3
BP	GO:0048639	positive regulation c	3/74	0.036672638	0.063513928	3
BP	GO:0042110	T cell activation	5/74	0.036930465	0.06389751	5
BP	GO:0009260	ribonucleotide biosy	3/74	0.03717392	0.06412938	3
BP	GO:1902275	regulation of chrom	3/74	0.03717392	0.06412938	3
BP	GO:1903708	positive regulation c	3/74	0.03717392	0.06412938	3
BP	GO:0030308	negative regulation	3/74	0.037678734	0.064173193	3
BP	GO:0055001	muscle cell develop	3/74	0.037678734	0.064173193	3
BP	GO:0043010	camera-type eye de	4/74	0.037878022	0.064173193	4
BP	GO:0010827	regulation of glucos	2/74	0.038351765	0.064173193	2
BP	GO:0014743	regulation of muscle	2/74	0.038351765	0.064173193	2
BP	GO:0032413	negative regulation	2/74	0.038351765	0.064173193	2
BP	GO:0055021	regulation of cardia	2/74	0.038351765	0.064173193	2
BP	GO:0072347	response to anesthe	2/74	0.038351765	0.064173193	2
BP	GO:0090398	cellular senescence	2/74	0.038351765	0.064173193	2
BP	GO:0006575	cellular modified an	3/74	0.038698937	0.064173193	3
BP	GO:0001660	fever generation	1/74	0.038945473	0.064173193	1
BP	GO:0006751	glutathione cataboli	1/74	0.038945473	0.064173193	1
BP	GO:0009396	folic acid-containing	1/74	0.038945473	0.064173193	1
BP	GO:0010918	positive regulation c	1/74	0.038945473	0.064173193	1
BP	GO:0010944	negative regulation	1/74	0.038945473	0.064173193	1
BP	GO:0014041	regulation of neuro	1/74	0.038945473	0.064173193	1
BP	GO:0030388	fructose 1,6-bispho:	1/74	0.038945473	0.064173193	1
BP	GO:0031284	positive regulation c	1/74	0.038945473	0.064173193	1
BP	GO:0032025	response to cobalt i	1/74	0.038945473	0.064173193	1
BP	GO:0032328	alanine transport	1/74	0.038945473	0.064173193	1
BP	GO:0032464	positive regulation c	1/74	0.038945473	0.064173193	1
BP	GO:0033210	leptin-mediated sig	1/74	0.038945473	0.064173193	1

BP	GO:0034135	regulation of toll-lik	1/74	0.038945473	0.064173193	1
BP	GO:0035672	oligopeptide transm	1/74	0.038945473	0.064173193	1
BP	GO:0035871	protein K11-linked (	1/74	0.038945473	0.064173193	1
BP	GO:0043653	mitochondrial fragm	1/74	0.038945473	0.064173193	1
BP	GO:0045348	positive regulation (	1/74	0.038945473	0.064173193	1
BP	GO:0045792	negative regulation	1/74	0.038945473	0.064173193	1
BP	GO:0045945	positive regulation (	1/74	0.038945473	0.064173193	1
BP	GO:0048021	regulation of melan	1/74	0.038945473	0.064173193	1
BP	GO:0048102	autophagic cell dea	1/74	0.038945473	0.064173193	1
BP	GO:0048149	behavioral response	1/74	0.038945473	0.064173193	1
BP	GO:0051901	positive regulation (	1/74	0.038945473	0.064173193	1
BP	GO:0060513	prostatic bud forma	1/74	0.038945473	0.064173193	1
BP	GO:0070099	regulation of chemo	1/74	0.038945473	0.064173193	1
BP	GO:0071233	cellular response to	1/74	0.038945473	0.064173193	1
BP	GO:0090037	positive regulation (	1/74	0.038945473	0.064173193	1
BP	GO:0090336	positive regulation (	1/74	0.038945473	0.064173193	1
BP	GO:0106049	regulation of cellula	1/74	0.038945473	0.064173193	1
BP	GO:1900222	negative regulation	1/74	0.038945473	0.064173193	1
BP	GO:1901725	regulation of histon	1/74	0.038945473	0.064173193	1
BP	GO:1901838	positive regulation (	1/74	0.038945473	0.064173193	1
BP	GO:1903800	positive regulation (	1/74	0.038945473	0.064173193	1
BP	GO:1990253	cellular response to	1/74	0.038945473	0.064173193	1
BP	GO:2000109	regulation of macro	1/74	0.038945473	0.064173193	1
BP	GO:2000253	positive regulation (	1/74	0.038945473	0.064173193	1
BP	GO:2001054	negative regulation	1/74	0.038945473	0.064173193	1
BP	GO:0055074	calcium ion homeos	5/74	0.038987368	0.064173193	5
BP	GO:0060562	epithelial tube morp	4/74	0.038988097	0.064173193	4
BP	GO:0001570	vasculogenesis	2/74	0.039248975	0.064301553	2
BP	GO:0003151	outflow tract morph	2/74	0.039248975	0.064301553	2
BP	GO:0030193	regulation of blood	2/74	0.039248975	0.064301553	2
BP	GO:0042590	antigen processing	2/74	0.039248975	0.064301553	2
BP	GO:0045913	positive regulation (	2/74	0.039248975	0.064301553	2
BP	GO:0032984	protein-containing	4/74	0.039362254	0.064427094	4
BP	GO:0050769	positive regulation (	5/74	0.039890073	0.065230278	5
BP	GO:1900046	regulation of hemos	2/74	0.040154168	0.065583976	2
BP	GO:0051054	positive regulation (	3/74	0.040255601	0.065583976	3
BP	GO:0071478	cellular response to	3/74	0.040255601	0.065583976	3
BP	GO:0098657	import into cell	3/74	0.040255601	0.065583976	3
BP	GO:0017038	protein import	3/74	0.040781496	0.066317833	3
BP	GO:0046390	ribose phosphate bi	3/74	0.040781496	0.066317833	3
BP	GO:0032436	positive regulation (	2/74	0.041067269	0.066475079	2
BP	GO:0033238	regulation of cellula	2/74	0.041067269	0.066475079	2
BP	GO:0034109	homotypic cell-cell	2/74	0.041067269	0.066475079	2
BP	GO:0110110	positive regulation (	2/74	0.041067269	0.066475079	2
BP	GO:1902930	regulation of alcoho	2/74	0.041067269	0.066475079	2
BP	GO:0051224	negative regulation	3/74	0.041310885	0.0668079	3
BP	GO:0070838	divalent metal ion tr	5/74	0.041422929	0.066927527	5
BP	GO:1901987	regulation of cell cy	5/74	0.041733763	0.067289014	5
BP	GO:0003333	amino acid transme	2/74	0.041988202	0.067289014	2
BP	GO:0071158	positive regulation (	2/74	0.041988202	0.067289014	2
BP	GO:1905954	positive regulation (	2/74	0.041988202	0.067289014	2
BP	GO:0002887	negative regulation	1/74	0.042756729	0.067289014	1
BP	GO:0005984	disaccharide metabo	1/74	0.042756729	0.067289014	1
BP	GO:0006857	oligopeptide transp	1/74	0.042756729	0.067289014	1
BP	GO:0007195	adenylate cyclase-ir	1/74	0.042756729	0.067289014	1
BP	GO:0016264	gap junction assemb	1/74	0.042756729	0.067289014	1
BP	GO:0019371	cyclooxygenase pat	1/74	0.042756729	0.067289014	1
BP	GO:0031652	positive regulation (	1/74	0.042756729	0.067289014	1

BP	GO:0031915	positive regulation c	1/74	0.042756729	0.067289014	1
BP	GO:0033629	negative regulation	1/74	0.042756729	0.067289014	1
BP	GO:0034776	response to histami	1/74	0.042756729	0.067289014	1
BP	GO:0036462	TRAIL-activated apc	1/74	0.042756729	0.067289014	1
BP	GO:0040015	negative regulation	1/74	0.042756729	0.067289014	1
BP	GO:0043129	surfactant homeosta	1/74	0.042756729	0.067289014	1
BP	GO:0051561	positive regulation c	1/74	0.042756729	0.067289014	1
BP	GO:0051574	positive regulation c	1/74	0.042756729	0.067289014	1
BP	GO:0051583	dopamine uptake ir	1/74	0.042756729	0.067289014	1
BP	GO:0051934	catecholamine upta	1/74	0.042756729	0.067289014	1
BP	GO:0060525	prostate glandular a	1/74	0.042756729	0.067289014	1
BP	GO:0060947	cardiac vascular smc	1/74	0.042756729	0.067289014	1
BP	GO:0070254	mucus secretion	1/74	0.042756729	0.067289014	1
BP	GO:0070673	response to interleu	1/74	0.042756729	0.067289014	1
BP	GO:0072537	fibroblast activation	1/74	0.042756729	0.067289014	1
BP	GO:0099149	regulation of postsy	1/74	0.042756729	0.067289014	1
BP	GO:1990440	positive regulation c	1/74	0.042756729	0.067289014	1
BP	GO:2000767	positive regulation c	1/74	0.042756729	0.067289014	1
BP	GO:0042509	regulation of tyrosir	2/74	0.042916894	0.067364964	2
BP	GO:0045582	positive regulation c	2/74	0.042916894	0.067364964	2
BP	GO:0001763	morphogenesis of a	3/74	0.042919953	0.067364964	3
BP	GO:0016042	lipid catabolic proce	4/74	0.043217546	0.067771541	4
BP	GO:0009749	response to glucose	3/74	0.043463256	0.068035467	3
BP	GO:1904950	negative regulation	3/74	0.043463256	0.068035467	3
BP	GO:0072511	divalent inorganic c	5/74	0.04362868	0.068233654	5
BP	GO:0050818	regulation of coagu	2/74	0.043853271	0.068402337	2
BP	GO:0051492	regulation of stress	2/74	0.043853271	0.068402337	2
BP	GO:0060420	regulation of heart g	2/74	0.043853271	0.068402337	2
BP	GO:0060541	respiratory system c	3/74	0.044010022	0.06858598	3
BP	GO:0050866	negative regulation	3/74	0.044560244	0.069381946	3
BP	GO:0034308	primary alcohol met	2/74	0.044797258	0.069597687	2
BP	GO:0045682	regulation of epider	2/74	0.044797258	0.069597687	2
BP	GO:0007159	leukocyte cell-cell a	4/74	0.044817576	0.069597687	4
BP	GO:0045930	negative regulation	4/74	0.045222754	0.06987859	4
BP	GO:0001942	hair follicle develop	2/74	0.045748782	0.06987859	2
BP	GO:0006919	activation of cystein	2/74	0.045748782	0.06987859	2
BP	GO:0007260	tyrosine phosphoryl	2/74	0.045748782	0.06987859	2
BP	GO:0032760	positive regulation c	2/74	0.045748782	0.06987859	2
BP	GO:0042446	hormone biosynthe	2/74	0.045748782	0.06987859	2
BP	GO:0050870	positive regulation c	3/74	0.046231579	0.06987859	3
BP	GO:0051701	interaction with hos	3/74	0.046231579	0.06987859	3
BP	GO:0002551	mast cell chemotaxi	1/74	0.046553074	0.06987859	1
BP	GO:0006020	inositol metabolic p	1/74	0.046553074	0.06987859	1
BP	GO:0006527	arginine catabolic p	1/74	0.046553074	0.06987859	1
BP	GO:0010649	regulation of cell co	1/74	0.046553074	0.06987859	1
BP	GO:0010960	magnesium ion hon	1/74	0.046553074	0.06987859	1
BP	GO:0014889	muscle atrophy	1/74	0.046553074	0.06987859	1
BP	GO:0031282	regulation of guany	1/74	0.046553074	0.06987859	1
BP	GO:0031392	regulation of prosta	1/74	0.046553074	0.06987859	1
BP	GO:0032429	regulation of phosp	1/74	0.046553074	0.06987859	1
BP	GO:0042368	vitamin D biosynthe	1/74	0.046553074	0.06987859	1
BP	GO:0042416	dopamine biosynthe	1/74	0.046553074	0.06987859	1
BP	GO:0043117	positive regulation c	1/74	0.046553074	0.06987859	1
BP	GO:0051481	negative regulation	1/74	0.046553074	0.06987859	1
BP	GO:0051549	positive regulation c	1/74	0.046553074	0.06987859	1
BP	GO:0051657	maintenance of org	1/74	0.046553074	0.06987859	1
BP	GO:0060742	epithelial cell differe	1/74	0.046553074	0.06987859	1
BP	GO:0070243	regulation of thymo	1/74	0.046553074	0.06987859	1

BP	GO:0070486	leukocyte aggregati	1/74	0.046553074	0.06987859	1
BP	GO:0071481	cellular response to	1/74	0.046553074	0.06987859	1
BP	GO:0071679	commissural neuror	1/74	0.046553074	0.06987859	1
BP	GO:0071888	macrophage apopto	1/74	0.046553074	0.06987859	1
BP	GO:0072578	neurotransmitter-ga	1/74	0.046553074	0.06987859	1
BP	GO:0090331	negative regulation	1/74	0.046553074	0.06987859	1
BP	GO:0097284	hepatocyte apoptot	1/74	0.046553074	0.06987859	1
BP	GO:0097531	mast cell migration	1/74	0.046553074	0.06987859	1
BP	GO:0150065	regulation of deace	1/74	0.046553074	0.06987859	1
BP	GO:1900376	regulation of secon	1/74	0.046553074	0.06987859	1
BP	GO:1903909	regulation of recept	1/74	0.046553074	0.06987859	1
BP	GO:1904181	positive regulation c	1/74	0.046553074	0.06987859	1
BP	GO:2000121	regulation of remov	1/74	0.046553074	0.06987859	1
BP	GO:2001053	regulation of meser	1/74	0.046553074	0.06987859	1
BP	GO:0001892	embryonic placenta	2/74	0.04670777	0.06987859	2
BP	GO:0006112	energy reserve meta	2/74	0.04670777	0.06987859	2
BP	GO:0045844	positive regulation c	2/74	0.04670777	0.06987859	2
BP	GO:0048636	positive regulation c	2/74	0.04670777	0.06987859	2
BP	GO:0033044	regulation of chrom	4/74	0.046864134	0.069993386	4
BP	GO:1904062	regulation of cation	4/74	0.046864134	0.069993386	4
BP	GO:0022404	molting cycle proce	2/74	0.047674151	0.070603319	2
BP	GO:0022405	hair cycle process	2/74	0.047674151	0.070603319	2
BP	GO:0032088	negative regulation	2/74	0.047674151	0.070603319	2
BP	GO:0034103	regulation of tissue	2/74	0.047674151	0.070603319	2
BP	GO:0061097	regulation of protei	2/74	0.047674151	0.070603319	2
BP	GO:0070098	chemokine-mediate	2/74	0.047674151	0.070603319	2
BP	GO:0098773	skin epidermis deve	2/74	0.047674151	0.070603319	2
BP	GO:1901863	positive regulation c	2/74	0.047674151	0.070603319	2
BP	GO:1903557	positive regulation c	2/74	0.047674151	0.070603319	2
BP	GO:2000177	regulation of neural	2/74	0.047674151	0.070603319	2
BP	GO:0032410	negative regulation	2/74	0.048647852	0.071704107	2
BP	GO:0097306	cellular response to	2/74	0.048647852	0.071704107	2
BP	GO:0002064	epithelial cell develo	3/74	0.04908562	0.071704107	3
BP	GO:0045185	maintenance of pro	2/74	0.049628802	0.071704107	2
BP	GO:0007178	transmembrane rec	4/74	0.049816057	0.071704107	4
CC	GO:0043020	NADPH oxidase cor	3/73	2.15E-05	0.002665265	3
CC	GO:0045177	apical part of cell	8/73	8.57E-05	0.0048015	8
CC	GO:0016323	basolateral plasma r	6/73	0.00015275	0.0048015	6
CC	GO:0016324	apical plasma meml	7/73	0.000174055	0.0048015	7
CC	GO:0005901	caveola	4/73	0.000221099	0.0048015	4
CC	GO:0005811	lipid droplet	4/73	0.000231937	0.0048015	4
CC	GO:0005791	rough endoplasmic	4/73	0.00027914	0.00495316	4
CC	GO:0005741	mitochondrial outer	5/73	0.000518423	0.008049199	5
CC	GO:0044853	plasma membrane r	4/73	0.000717395	0.008960573	4
CC	GO:1990204	oxidoreductase com	4/73	0.000848605	0.008960573	4
CC	GO:0097449	astrocyte projection	2/73	0.000871189	0.008960573	2
CC	GO:0031968	organelle outer mer	5/73	0.000897187	0.008960573	5
CC	GO:0019867	outer membrane	5/73	0.000937823	0.008960573	5
CC	GO:0045121	membrane raft	6/73	0.001099278	0.009252141	6
CC	GO:0098857	membrane microdo	6/73	0.001117314	0.009252141	6
CC	GO:0098589	membrane region	6/73	0.001351971	0.010495563	6
CC	GO:0097038	perinuclear endopla	2/73	0.002977017	0.021751577	2
CC	GO:0031528	microvillus membra	2/73	0.003252755	0.022445914	2
CC	GO:0097386	glial cell projection	2/73	0.003539988	0.022473982	2
CC	GO:0005902	microvillus	3/73	0.003618692	0.022473982	3
CC	GO:0030139	endocytic vesicle	5/73	0.005314197	0.031432345	5
CC	GO:0009925	basal plasma memb	2/73	0.007025701	0.034806645	2
CC	GO:0042470	melanosome	3/73	0.007151806	0.034806645	3

CC	GO:0048770	pigment granule	3/73	0.007151806	0.034806645	3
CC	GO:0031253	cell projection mem	5/73	0.007194747	0.034806645	5
CC	GO:0031983	vesicle lumen	5/73	0.007285798	0.034806645	5
CC	GO:0016363	nuclear matrix	3/73	0.007721457	0.035521714	3
CC	GO:0034399	nuclear periphery	3/73	0.012712294	0.056392885	3
CC	GO:0042383	sarcolemma	3/73	0.014051315	0.060183489	3
CC	GO:0014704	intercalated disc	2/73	0.014768486	0.061146714	2
CC	GO:0045178	basal part of cell	2/73	0.01533485	0.061443543	2
CC	GO:0072562	blood microparticle	3/73	0.017270202	0.067035652	3
CC	GO:0031901	early endosome me	3/73	0.017896074	0.067360024	3
CC	GO:0030667	secretory granule m	4/73	0.024662544	0.090098459	4
CC	GO:0031093	platelet alpha granu	2/73	0.02560404	0.090865465	2
CC	GO:0005938	cell cortex	4/73	0.027403665	0.091657902	4
CC	GO:0044291	cell-cell contact zor	2/73	0.028506587	0.091657902	2
CC	GO:0005635	nuclear envelope	5/73	0.028740695	0.091657902	5
CC	GO:0032592	integral component	2/73	0.030005126	0.091657902	2
CC	GO:0070821	tertiary granule mer	2/73	0.030005126	0.091657902	2
CC	GO:0098573	intrinsic component	2/73	0.030765979	0.091657902	2
CC	GO:0034774	secretory granule lu	4/73	0.031230662	0.091657902	4
CC	GO:0000792	heterochromatin	2/73	0.032310525	0.091657902	2
CC	GO:0060205	cytoplasmic vesicle	4/73	0.032468647	0.091657902	4
CC	GO:0043025	neuronal cell body	5/73	0.036995078	0.100238497	5
CC	GO:0016234	inclusion body	2/73	0.037122223	0.100238497	2
CC	GO:0120111	neuron projection c	2/73	0.038783779	0.102496885	2
CC	GO:0031932	TORC2 complex	1/73	0.039990622	0.103484503	1
CC	GO:0005769	early endosome	4/73	0.040856275	0.103566926	4
CC	GO:0098858	actin-based cell pro	3/73	0.042010602	0.10436318	3
CC	GO:0031091	platelet alpha granu	2/73	0.044816461	0.107051465	2
CC	GO:0035579	specific granule mer	2/73	0.044816461	0.107051465	2
CC	GO:0044233	mitochondria-assoc	1/73	0.047090251	0.11036047	1
MF	GO:0016701	oxidoreductase acti	7/72	1.51E-11	3.46E-09	7
MF	GO:0016702	oxidoreductase acti	6/72	1.01E-09	1.16E-07	6
MF	GO:0005506	iron ion binding	9/72	1.08E-08	8.20E-07	9
MF	GO:0016651	oxidoreductase acti	7/72	2.58E-07	1.48E-05	7
MF	GO:0051213	dioxygenase activity	6/72	1.86E-06	8.51E-05	6
MF	GO:0008199	ferric iron binding	3/72	1.04E-05	0.000396309	3
MF	GO:0016175	superoxide-generat	3/72	1.38E-05	0.000429135	3
MF	GO:0015144	carbohydrate transr	4/72	1.50E-05	0.000429135	4
MF	GO:0020037	heme binding	6/72	1.82E-05	0.000462655	6
MF	GO:0050662	coenzyme binding	8/72	2.34E-05	0.000533462	8
MF	GO:0046906	tetrapyrrole binding	6/72	2.73E-05	0.00056736	6
MF	GO:0050664	oxidoreductase acti	3/72	4.22E-05	0.000802433	3
MF	GO:0016705	oxidoreductase acti	6/72	4.58E-05	0.000805606	6
MF	GO:0030170	pyridoxal phosphate	4/72	6.83E-05	0.000941303	4
MF	GO:0070279	vitamin B6 binding	4/72	6.83E-05	0.000941303	4
MF	GO:0005355	glucose transmemb	3/72	7.01E-05	0.000941303	3
MF	GO:0015149	hexose transmembr	3/72	7.01E-05	0.000941303	3
MF	GO:0008483	transaminase activit	3/72	8.15E-05	0.00103415	3
MF	GO:0015145	monosaccharide tra	3/72	9.41E-05	0.001131105	3
MF	GO:0016769	transferase activity,	3/72	0.000107882	0.001224546	3
MF	GO:0016829	lyase activity	6/72	0.000112579	0.001224546	6
MF	GO:0051119	sugar transmembrai	3/72	0.000122934	0.001276401	3
MF	GO:0031406	carboxylic acid bind	6/72	0.000133827	0.001329086	6
MF	GO:0004033	aldo-keto reductase	3/72	0.000157	0.001494257	3
MF	GO:0031625	ubiquitin protein lig	7/72	0.000175055	0.001599449	7
MF	GO:0043177	organic acid binding	6/72	0.000185831	0.001632601	6
MF	GO:0044389	ubiquitin-like protei	7/72	0.00025292	0.002139715	7
MF	GO:0016209	antioxidant activity	4/72	0.000415866	0.003298343	4

MF	GO:0019825	oxygen binding	3/72	0.000418753	0.003298343	3
MF	GO:0016709	oxidoreductase acti	3/72	0.000531327	0.00404554	3
MF	GO:0009055	electron transfer act	4/72	0.001199682	0.00882974	4
MF	GO:0004032	alditol:NADP+ 1-ox	2/72	0.001236977	0.00882974	2
MF	GO:0016836	hydro-lyase activity	3/72	0.001533829	0.010616934	3
MF	GO:0072349	modified amino aci	2/72	0.001656419	0.011128264	2
MF	GO:0003996	acyl-CoA ligase acti	2/72	0.001888076	0.012322183	2
MF	GO:0033293	monocarboxylic aci	3/72	0.002252459	0.014291921	3
MF	GO:0019842	vitamin binding	4/72	0.002416911	0.014784774	4
MF	GO:0002039	p53 binding	3/72	0.002459587	0.014784774	3
MF	GO:0008106	alcohol dehydroger	2/72	0.003260991	0.019099461	2
MF	GO:0016835	carbon-oxygen lyas	3/72	0.003535993	0.020192383	3
MF	GO:0050660	flavin adenine dinu	3/72	0.004093343	0.022805017	3
MF	GO:0008198	ferrous iron binding	2/72	0.004252248	0.023126261	2
MF	GO:0016405	CoA-ligase activity	2/72	0.004980971	0.025858151	2
MF	GO:0016628	oxidoreductase acti	2/72	0.004980971	0.025858151	2
MF	GO:0090482	vitamin transmemb	2/72	0.00536536	0.027234693	2
MF	GO:0005126	cytokine receptor bi	5/72	0.006169	0.030633247	5
MF	GO:0016878	acid-thiol ligase act	2/72	0.006597301	0.03206303	2
MF	GO:0015175	neutral amino acid t	2/72	0.007033839	0.033472435	2
MF	GO:0004497	monooxygenase act	3/72	0.007663012	0.035722311	3
MF	GO:0016229	steroid dehydrogen	2/72	0.008906551	0.039123919	2
MF	GO:0016831	carboxy-lyase activi	2/72	0.008906551	0.039123919	2
MF	GO:1901682	sulfur compound tr	2/72	0.008906551	0.039123919	2
MF	GO:0008514	organic anion trans	4/72	0.010577549	0.045587448	4
MF	GO:0016877	ligase activity, formi	2/72	0.011524213	0.048747644	2
MF	GO:0005125	cytokine activity	4/72	0.012378228	0.051408141	4
MF	GO:0016616	oxidoreductase acti	3/72	0.012628942	0.051512791	3
MF	GO:0016614	oxidoreductase acti	3/72	0.015342922	0.061485025	3
MF	GO:0015485	cholesterol binding	2/72	0.016971835	0.066840077	2
MF	GO:0016830	carbon-carbon lyas	2/72	0.018305005	0.070868618	2
MF	GO:0004601	peroxidase activity	2/72	0.018987719	0.071101552	2
MF	GO:0015459	potassium channel	2/72	0.018987719	0.071101552	2
MF	GO:0015179	L-amino acid transn	2/72	0.020384926	0.075102358	2
MF	GO:0016684	oxidoreductase acti	2/72	0.021823874	0.076692803	2
MF	GO:0032934	sterol binding	2/72	0.021823874	0.076692803	2
MF	GO:0043621	protein self-associa	2/72	0.021823874	0.076692803	2
MF	GO:0016627	oxidoreductase acti	2/72	0.023303812	0.080652747	2
MF	GO:0005507	copper ion binding	2/72	0.024058921	0.082023344	2
MF	GO:0016655	oxidoreductase acti	2/72	0.024824	0.083387122	2
MF	GO:0004860	protein kinase inhib	2/72	0.027178152	0.089971913	2
MF	GO:0019210	kinase inhibitor acti	2/72	0.030451136	0.099366866	2
MF	GO:0048365	Rac GTPase binding	2/72	0.032143518	0.103412059	2
MF	GO:0001228	DNA-binding transc	5/72	0.033135211	0.104551343	5
MF	GO:0001216	DNA-binding transc	5/72	0.033413067	0.104551343	5
MF	GO:0015171	amino acid transme	2/72	0.03475	0.107265292	2
MF	GO:0019887	protein kinase regul	3/72	0.037029375	0.112688321	3
MF	GO:0038024	cargo receptor activ	2/72	0.038348508	0.112688321	2
MF	GO:0004952	dopamine neurotra	1/72	0.039960213	0.112688321	1
MF	GO:0005351	carbohydrate:proto	1/72	0.039960213	0.112688321	1
MF	GO:0016176	superoxide-generat	1/72	0.039960213	0.112688321	1
MF	GO:0016803	ether hydrolase acti	1/72	0.039960213	0.112688321	1
MF	GO:0030957	Tat protein binding	1/72	0.039960213	0.112688321	1
MF	GO:0005536	glucose binding	1/72	0.043868551	0.115178168	1
MF	GO:0008429	phosphatidylethano	1/72	0.043868551	0.115178168	1
MF	GO:0015643	toxic substance bin	1/72	0.043868551	0.115178168	1
MF	GO:0016801	hydrolase activity, a	1/72	0.043868551	0.115178168	1
MF	GO:0042910	xenobiotic transme	1/72	0.043868551	0.115178168	1

MF	GO:0140104	molecular carrier ac	1/72	0.043868551	0.115178168	1
MF	GO:0048018	receptor ligand acti	5/72	0.046420443	0.115639763	5
MF	GO:0043178	alcohol binding	2/72	0.046934161	0.115639763	2
MF	GO:0004861	cyclin-dependent p	1/72	0.047761198	0.115639763	1
MF	GO:0061578	Lys63-specific deub	1/72	0.047761198	0.115639763	1
MF	GO:0008509	anion transmembra	4/72	0.048024529	0.115639763	4
MF	GO:0030546	signaling receptor a	5/72	0.048145259	0.115639763	5
	ID	Description	GeneRatio	pvalue	qvalue	Count
KEGG	hsa04066	HIF-1 signaling path	10/58	3.88E-09	5.68E-07	10
KEGG	hsa00590	Arachidonic acid me	6/58	4.22E-06	0.000308874	6
KEGG	hsa04216	Ferroptosis	5/58	9.69E-06	0.000472604	5
KEGG	hsa01230	Biosynthesis of amir	5/58	0.000185417	0.006144058	5
KEGG	hsa05140	Leishmaniasis	5/58	0.000209959	0.006144058	5
KEGG	hsa01210	2-Oxocarboxylic aci	3/58	0.00031068	0.006645828	3
KEGG	hsa05206	MicroRNAs in cance	9/58	0.000317948	0.006645828	9
KEGG	hsa05167	Kaposi sarcoma-ass	7/58	0.000418882	0.006918154	7
KEGG	hsa00270	Cysteine and methic	4/58	0.000425541	0.006918154	4
KEGG	hsa00220	Arginine biosynthes	3/58	0.000486281	0.007115065	3
KEGG	hsa04726	Serotonergic synaps	5/58	0.001326515	0.017147847	5
KEGG	hsa04920	Adipocytokine signa	4/58	0.001444142	0.017147847	4
KEGG	hsa05230	Central carbon meta	4/58	0.001523568	0.017147847	4
KEGG	hsa03320	PPAR signaling path	4/58	0.002065164	0.020144411	4
KEGG	hsa05212	Pancreatic cancer	4/58	0.002065164	0.020144411	4
KEGG	hsa04140	Autophagy - anima	5/58	0.002865604	0.024849615	5
KEGG	hsa05418	Fluid shear stress ar	5/58	0.003051034	0.024849615	5
KEGG	hsa05219	Bladder cancer	3/58	0.003057039	0.024849615	3
KEGG	hsa05205	Proteoglycans in cai	6/58	0.003295251	0.025376174	6
KEGG	hsa05235	PD-L1 expression ai	4/58	0.003668983	0.026841507	4
KEGG	hsa05323	Rheumatoid arthritis	4/58	0.004295673	0.029681869	4
KEGG	hsa04657	IL-17 signaling path	4/58	0.004462957	0.029681869	4
KEGG	hsa05163	Human cytomegalo	6/58	0.005195922	0.033054147	6
KEGG	hsa04931	Insulin resistance	4/58	0.007284674	0.044410949	4
KEGG	hsa00480	Glutathione metabc	3/58	0.007740369	0.045301528	3
KEGG	hsa04978	Mineral absorption	3/58	0.008916847	0.049082137	3
KEGG	hsa01200	Carbon metabolism	4/58	0.009057243	0.049082137	4

Table S4 1502 FRLRS in the TCGA-KIRC cohort

ferrGene	lncRNA	cor	pvalue	Regulation
FANCD2	AC022211.1	0.530919434	1.58E-40	postive
HELLS	AC022211.1	0.586423045	4.22E-51	postive
TUBE1	AC022211.1	0.40697442	6.43E-23	postive
ALOX12	AC022211.1	0.59287198	1.82E-52	postive
GABPB1	AC022211.1	0.403895849	1.44E-22	postive
DUOX1	AC022211.1	0.431507926	7.45E-26	postive
ATM	AC022211.1	0.501894755	9.82E-36	postive
FBXW7	AC022211.1	0.433563216	4.12E-26	postive
GPX4	AL049840.4	-0.41368485	1.07E-23	negative
BRD4	AL049840.4	0.404703821	1.17E-22	postive
ZNF419	AL049840.4	0.547004397	2.17E-43	postive
VEGFA	AL049840.4	0.4913045	4.26E-34	postive
TUBE1	AL049840.4	0.489617886	7.67E-34	postive
SETD1B	AL049840.4	0.519712463	1.27E-38	postive
ALOX12	AL049840.4	0.478455968	3.47E-32	postive
GABPB1	AL049840.4	0.550646302	4.64E-44	postive
ZEB1	AL049840.4	0.587834163	2.13E-51	postive
MAPK8	AL049840.4	0.523129304	3.40E-39	postive
ATM	AL049840.4	0.520506211	9.38E-39	postive
YY1AP1	AL049840.4	0.469250204	7.26E-31	postive
FBXW7	AL049840.4	0.499332128	2.48E-35	postive
AURKA	AL021807.1	0.400311954	3.67E-22	postive
FANCD2	AC002116.2	0.480192137	1.94E-32	postive
HELLS	AC002116.2	0.434047869	3.59E-26	postive
TFAP2C	AC002116.2	0.458916106	1.98E-29	postive
HBA1	AC002116.2	0.450906945	2.37E-28	postive
HELLS	AC018695.4	0.455291724	6.13E-29	postive
ZNF419	AC018695.4	0.509596034	5.82E-37	postive
VEGFA	AC018695.4	0.400406989	3.58E-22	postive
TUBE1	AC018695.4	0.576242539	5.22E-49	postive
SETD1B	AC018695.4	0.487151209	1.80E-33	postive
ALOX12	AC018695.4	0.588177976	1.81E-51	postive
GABPB1	AC018695.4	0.524119467	2.31E-39	postive
MAPK8	AC018695.4	0.462858012	5.68E-30	postive
LINC00472	AC018695.4	0.535628172	2.38E-41	postive
ATM	AC018695.4	0.624901986	1.01E-59	postive
FBXW7	AC018695.4	0.492888988	2.44E-34	postive
FANCD2	AP000487.1	0.421125951	1.39E-24	postive
HELLS	AP000487.1	0.565368004	7.46E-47	postive
ZNF419	AP000487.1	0.465384344	2.53E-30	postive
TUBE1	AP000487.1	0.516645112	4.11E-38	postive
ALOX12	AP000487.1	0.623405312	2.30E-59	postive
GABPB1	AP000487.1	0.552711417	1.92E-44	postive
MAPK8	AP000487.1	0.41191613	1.72E-23	postive
LINC00472	AP000487.1	0.628281074	1.55E-60	postive
ATM	AP000487.1	0.628041751	1.77E-60	postive
FBXW7	AP000487.1	0.49508592	1.13E-34	postive
KLHL24	AL139147.1	0.415426629	6.67E-24	postive
IREB2	AL139147.1	0.48456876	4.38E-33	postive
GABPB1	AL139147.1	0.458277178	2.41E-29	postive
PIK3CA	AL139147.1	0.481305268	1.33E-32	postive
ZEB1	AL139147.1	0.405163123	1.04E-22	postive
MAPK8	AL139147.1	0.478841829	3.05E-32	postive
LINC00472	AL139147.1	0.6350716	3.31E-62	postive
PRKAA2	AL139147.1	0.424734534	5.10E-25	postive
ATM	AL139147.1	0.521582128	6.19E-39	postive

KLHL24	AL359715.3	0.450128348	3.01E-28 postive
LINC00472	AL359715.3	0.64764169	2.08E-65 postive
ZNF419	AC008763.1	0.423820978	6.58E-25 postive
SLC2A12	AL353152.1	0.684767108	8.11E-76 postive
PLIN4	AL353152.1	0.416053532	5.62E-24 postive
HELLS	AC008569.2	0.514120432	1.07E-37 postive
ZNF419	AC008569.2	0.481355274	1.31E-32 postive
TUBE1	AC008569.2	0.508726418	8.04E-37 postive
ALOX12	AC008569.2	0.615400073	1.76E-57 postive
GABPB1	AC008569.2	0.51929023	1.50E-38 postive
LINC00472	AC008569.2	0.559805425	8.81E-46 postive
ATM	AC008569.2	0.601397525	2.57E-54 postive
FBXW7	AC008569.2	0.520051702	1.12E-38 postive
PHKG2	AC009403.1	0.417740422	3.55E-24 postive
PHKG2	PARD3-AS1	0.494315465	1.48E-34 postive
TAZ	PARD3-AS1	0.557475583	2.44E-45 postive
SLC2A1	BX640514.2	0.408171701	4.68E-23 postive
PROM2	AC090578.1	0.69065974	1.29E-77 postive
WIPI1	RNF157-AS1	0.442837038	2.71E-27 postive
MTOR	LINC02175	0.410228618	2.71E-23 postive
ZNF419	LINC02175	0.409348467	3.42E-23 postive
TUBE1	LINC02175	0.457701534	2.89E-29 postive
SETD1B	LINC02175	0.43325056	4.51E-26 postive
ALOX12	LINC02175	0.602862289	1.22E-54 postive
IREB2	LINC02175	0.516172267	4.92E-38 postive
SP1	LINC02175	0.42916529	1.46E-25 postive
GABPB1	LINC02175	0.466340165	1.86E-30 postive
PIK3CA	LINC02175	0.408536934	4.25E-23 postive
MAPK8	LINC02175	0.489585981	7.76E-34 postive
LINC00472	LINC02175	0.500230385	1.79E-35 postive
ATM	LINC02175	0.678135089	7.60E-74 postive
FBXW7	LINC02175	0.410583287	2.46E-23 postive
ZNF419	AC008655.2	0.437732329	1.23E-26 postive
TUBE1	AC008655.2	0.441454088	4.09E-27 postive
ALOX12	AC008655.2	0.582650442	2.56E-50 postive
RGS4	LINC02611	0.408044334	4.84E-23 postive
FH	AC118345.1	0.460314319	1.27E-29 postive
ISCU	AC118345.1	0.558824932	1.35E-45 postive
DDIT3	AC118345.1	0.480189828	1.94E-32 postive
GPT2	AC118345.1	0.533321923	6.05E-41 postive
HERPUD1	AC118345.1	0.481400143	1.29E-32 postive
KLHL24	AC118345.1	0.426013899	3.56E-25 postive
SLC2A12	AC118345.1	0.524207	2.23E-39 postive
GOT1	AC118345.1	0.426245033	3.33E-25 postive
ATG4D	AC118345.1	0.522880752	3.74E-39 postive
MAP1LC3A	AC118345.1	0.401589281	2.64E-22 postive
GABARAPL1	AC118345.1	0.778978591	6.25E-111 postive
WIPI2	AC118345.1	0.556948928	3.07E-45 postive
BAP1	AC118345.1	0.401247869	2.88E-22 postive
LPIN1	AC118345.1	0.568978929	1.47E-47 postive
KLHL24	AC095055.1	0.441045952	4.62E-27 postive
IREB2	AC095055.1	0.465686792	2.30E-30 postive
GABPB1	AC095055.1	0.453824838	9.66E-29 postive
PIK3CA	AC095055.1	0.443653555	2.13E-27 postive
KRAS	AC095055.1	0.41997511	1.92E-24 postive
MAPK8	AC095055.1	0.498958531	2.83E-35 postive
LINC00472	AC095055.1	0.588426633	1.60E-51 postive
PRKAA2	AC095055.1	0.407744125	5.25E-23 postive

PRKAA1	AC095055.1	0.519544541	1.36E-38	postive
SLC1A4	AC026369.2	0.449186798	4.01E-28	postive
ZEB1	AC026369.2	0.482233649	9.70E-33	postive
EPAS1	AC026369.2	0.444631262	1.59E-27	postive
HELLS	AC009704.2	0.54710045	2.08E-43	postive
TUBE1	AC009704.2	0.481200073	1.38E-32	postive
ALOX12	AC009704.2	0.478684071	3.22E-32	postive
GABPB1	AC009704.2	0.508276261	9.50E-37	postive
LINC00472	AC009704.2	0.486688965	2.11E-33	postive
ATM	AC009704.2	0.715969009	7.51E-86	postive
FBXW7	AC009704.2	0.473662673	1.71E-31	postive
ZNF419	OGFR-AS1	0.428259623	1.88E-25	postive
ALOX12	OGFR-AS1	0.477706064	4.46E-32	postive
PHKG2	OGFR-AS1	0.525916362	1.15E-39	postive
ULK1	OGFR-AS1	0.401312514	2.83E-22	postive
TAZ	OGFR-AS1	0.565051926	8.60E-47	postive
ZNF419	AC015871.3	0.5050121	3.16E-36	postive
TUBE1	AC015871.3	0.561872514	3.54E-46	postive
ALOX12	AC015871.3	0.579744588	1.01E-49	postive
GABPB1	AC015871.3	0.512981644	1.64E-37	postive
LINC00472	AC015871.3	0.528056005	4.93E-40	postive
LPIN1	AC015871.3	0.413889854	1.01E-23	postive
ATM	AC015871.3	0.468323105	9.80E-31	postive
BRD4	AC010809.2	0.441472397	4.07E-27	postive
ZNF419	AC010809.2	0.598979193	8.71E-54	postive
TUBE1	AC010809.2	0.500770584	1.47E-35	postive
SETD1B	AC010809.2	0.411491084	1.93E-23	postive
ALOX12	AC010809.2	0.463110775	5.24E-30	postive
GABPB1	AC010809.2	0.433018936	4.83E-26	postive
PHKG2	AC010809.2	0.511414413	2.96E-37	postive
TAZ	AC010809.2	0.630920325	3.51E-61	postive
FBXW7	AC010809.2	0.413664048	1.08E-23	postive
TUBE1	AP002840.2	0.435377481	2.44E-26	postive
ALOX12	AP002840.2	0.527653296	5.78E-40	postive
MTOR	AC007637.1	0.514164862	1.05E-37	postive
ISCU	AC007637.1	0.416902341	4.46E-24	postive
ACSL3	AC007637.1	0.488186933	1.26E-33	postive
ZNF419	AC007637.1	0.401525688	2.68E-22	postive
KLHL24	AC007637.1	0.686787159	1.98E-76	postive
MAP3K5	AC007637.1	0.454166489	8.69E-29	postive
EIF2AK4	AC007637.1	0.425303009	4.34E-25	postive
IREB2	AC007637.1	0.570787503	6.44E-48	postive
GABPB1	AC007637.1	0.410744644	2.36E-23	postive
PIK3CA	AC007637.1	0.543921215	7.90E-43	postive
KRAS	AC007637.1	0.43118582	8.18E-26	postive
SLC38A1	AC007637.1	0.404439993	1.25E-22	postive
ATG7	AC007637.1	0.406505761	7.28E-23	postive
GABARAPL1	AC007637.1	0.51577438	5.73E-38	postive
MAPK8	AC007637.1	0.408342505	4.48E-23	postive
LINC00472	AC007637.1	0.703311736	1.27E-81	postive
PRKAA2	AC007637.1	0.406033469	8.24E-23	postive
PRKAA1	AC007637.1	0.446642249	8.66E-28	postive
LPIN1	AC007637.1	0.651009748	2.71E-66	postive
ATM	AC007637.1	0.559467953	1.02E-45	postive
TFAP2C	AC008429.1	0.491506437	3.97E-34	postive
HBA1	AC008429.1	0.446002238	1.05E-27	postive
ALOX12	AC005006.1	0.416113094	5.53E-24	postive
ACSL4	CASC9	0.486554319	2.22E-33	postive

GABPB1	AC005746.2	0.413628381	1.09E-23	postive
NOX1	AC092354.1	0.486521769	2.24E-33	postive
BECN1	AC092354.1	-0.431248962	8.03E-26	negative
ALOX12	AC022916.1	0.426712365	2.92E-25	postive
BRD4	AL031714.1	0.492505899	2.80E-34	postive
ZNF419	AL031714.1	0.556934333	3.09E-45	postive
VEGFA	AL031714.1	0.556317436	4.05E-45	postive
TUBE1	AL031714.1	0.544868977	5.32E-43	postive
SETD1B	AL031714.1	0.535183155	2.85E-41	postive
DRD4	AL031714.1	0.458628771	2.16E-29	postive
ALOX12	AL031714.1	0.644932376	1.05E-64	postive
PHKG2	AL031714.1	0.477810884	4.31E-32	postive
TAZ	AL031714.1	0.609234652	4.54E-56	postive
HELLS	TTN-AS1	0.58883438	1.31E-51	postive
ZNF419	TTN-AS1	0.445337763	1.28E-27	postive
TUBE1	TTN-AS1	0.587072955	3.08E-51	postive
ALOX12	TTN-AS1	0.540366887	3.44E-42	postive
GABPB1	TTN-AS1	0.634706215	4.08E-62	postive
ATG7	TTN-AS1	0.432533525	5.55E-26	postive
MAPK8	TTN-AS1	0.464920259	2.94E-30	postive
LINC00472	TTN-AS1	0.589668002	8.76E-52	postive
ATM	TTN-AS1	0.791717326	5.00E-117	postive
FBXW7	TTN-AS1	0.605639889	2.93E-55	postive
TFAP2C	AC009509.4	0.480779854	1.59E-32	postive
HBA1	AC009509.4	0.497299267	5.12E-35	postive
TFAP2C	AL023881.1	0.479924719	2.12E-32	postive
HBA1	AL023881.1	0.55416734	1.03E-44	postive
SAT1	LINC01480	0.437518844	1.31E-26	postive
ZNF419	AC110285.2	0.500910085	1.40E-35	postive
VEGFA	AC110285.2	0.513294947	1.46E-37	postive
TUBE1	AC110285.2	0.502084351	9.17E-36	postive
ALOX12	AC110285.2	0.496574	6.64E-35	postive
PHKG2	AC110285.2	0.454043375	9.03E-29	postive
TAZ	AC110285.2	0.600855353	3.38E-54	postive
HELLS	AC105036.3	0.559258435	1.12E-45	postive
TUBE1	AC105036.3	0.424112938	6.07E-25	postive
ALOX12	AC105036.3	0.437446908	1.33E-26	postive
GABPB1	AC105036.3	0.526745601	8.27E-40	postive
LINC00472	AC105036.3	0.654218123	3.81E-67	postive
ATM	AC105036.3	0.645118353	9.38E-65	postive
FBXW7	AC105036.3	0.427096515	2.62E-25	postive
ZNF419	AC138956.2	0.479964114	2.09E-32	postive
TUBE1	AC138956.2	0.529412945	2.88E-40	postive
SETD1B	AC138956.2	0.419823652	2.00E-24	postive
ALOX12	AC138956.2	0.606569203	1.81E-55	postive
LINC00472	AC138956.2	0.482029721	1.04E-32	postive
ATM	AC138956.2	0.57262894	2.77E-48	postive
HSF1	AC016065.1	0.438107647	1.10E-26	postive
OTUB1	AC016065.1	0.406879724	6.59E-23	postive
PHKG2	AC016065.1	0.48171478	1.16E-32	postive
MAPK1	AC016065.1	-0.423488358	7.22E-25	negative
BRD4	AL031600.1	0.407042143	6.32E-23	postive
ZNF419	AL031600.1	0.509997307	5.02E-37	postive
TUBE1	AL031600.1	0.481807526	1.12E-32	postive
SETD1B	AL031600.1	0.495831061	8.64E-35	postive
ALOX12	AL031600.1	0.633707566	7.22E-62	postive
PHKG2	AL031600.1	0.436594164	1.71E-26	postive
TAZ	AL031600.1	0.470714208	4.50E-31	postive

FH	LINC00323	0.513925221	1.15E-37	postive
ISCU	LINC00323	0.586825817	3.47E-51	postive
ATP5MC3	LINC00323	0.427675209	2.22E-25	postive
SLC2A8	LINC00323	0.419551793	2.15E-24	postive
SLC2A12	LINC00323	0.467224841	1.40E-30	postive
GOT1	LINC00323	0.539224876	5.50E-42	postive
ATG4D	LINC00323	0.500144704	1.85E-35	postive
GABARAPL2	LINC00323	0.61759524	5.42E-58	postive
GABARAPL1	LINC00323	0.532503397	8.40E-41	postive
WIPI2	LINC00323	0.489136998	9.07E-34	postive
LPIN1	LINC00323	0.455779675	5.27E-29	postive
HELLS	AL121672.1	0.459471999	1.66E-29	postive
KLHL24	AL121672.1	0.533032351	6.79E-41	postive
TUBE1	AL121672.1	0.406295386	7.69E-23	postive
IREB2	AL121672.1	0.43436474	3.27E-26	postive
GABPB1	AL121672.1	0.567629785	2.70E-47	postive
PIK3CA	AL121672.1	0.463979498	3.97E-30	postive
ATG7	AL121672.1	0.440273862	5.80E-27	postive
MAPK8	AL121672.1	0.41715075	4.17E-24	postive
LINC00472	AL121672.1	0.869644608	9.48E-167	postive
TGFBR1	AL121672.1	0.407295586	5.91E-23	postive
ATM	AL121672.1	0.66663587	1.51E-70	postive
FBXW7	AL121672.1	0.442475558	3.02E-27	postive
MUC1	LINC01896	0.531952731	1.05E-40	postive
SLC2A1	LINC01896	0.402377162	2.15E-22	postive
NCOA4	AC017104.1	-0.439352246	7.62E-27	negative
PHKG2	AC017104.1	0.531094714	1.48E-40	postive
TAZ	AC017104.1	0.628197777	1.62E-60	postive
HELLS	AL442067.1	0.546843739	2.32E-43	postive
ALOX12	AL442067.1	0.466248798	1.92E-30	postive
GABPB1	AL442067.1	0.4888982	9.85E-34	postive
LINC00472	AL442067.1	0.560366992	6.88E-46	postive
ATM	AL442067.1	0.609893985	3.22E-56	postive
FBXW7	AL442067.1	0.443594449	2.16E-27	postive
OXSR1	AC004943.2	0.449684646	3.44E-28	postive
IREB2	AC004943.2	0.524519166	1.98E-39	postive
SP1	AC004943.2	0.445416188	1.25E-27	postive
PIK3CA	AC004943.2	0.475892648	8.17E-32	postive
MAPK1	AC004943.2	0.446533489	8.95E-28	postive
MAPK8	AC004943.2	0.447134452	7.47E-28	postive
LINC00472	AC004943.2	0.401345184	2.81E-22	postive
PRKAA2	AC004943.2	0.45913308	1.85E-29	postive
ATM	AC004943.2	0.461513671	8.70E-30	postive
SIRT1	AC004943.2	0.404475503	1.24E-22	postive
TFAP2C	LINC01715	0.44497899	1.43E-27	postive
HBA1	LINC01715	0.444240723	1.78E-27	postive
ALOX12	AC005962.1	0.471755504	3.20E-31	postive
TAZ	AC005962.1	0.438255274	1.05E-26	postive
TFAP2C	AC138230.1	0.548099972	1.37E-43	postive
HBA1	AC138230.1	0.492705106	2.61E-34	postive
DUOX1	AC138230.1	0.410680281	2.40E-23	postive
ISCU	LINC00239	0.400428053	3.56E-22	postive
ATG4D	LINC00239	0.424019289	6.23E-25	postive
TUBE1	AC010883.1	0.4715138	3.47E-31	postive
DRD4	AC010883.1	0.428390846	1.82E-25	postive
ALOX12	AC010883.1	0.514192876	1.04E-37	postive
PHKG2	AC010883.1	0.636101323	1.83E-62	postive
TAZ	AC010883.1	0.763633378	4.19E-104	postive

IL33	AC104260.1	0.453147294	1.19E-28	postive
GABPB1	AC104260.1	0.516385157	4.54E-38	postive
ZEB1	AC104260.1	0.400137115	3.84E-22	postive
LINC00472	AC104260.1	0.519620644	1.32E-38	postive
FBXW7	AC104260.1	0.401279133	2.86E-22	postive
PTGS2	CFAP58-DT	0.477769587	4.37E-32	postive
SLC2A3	CFAP58-DT	0.418770597	2.67E-24	postive
ZNF419	AP001107.8	0.423449313	7.30E-25	postive
VEGFA	AP001107.8	0.542394644	1.49E-42	postive
TUBE1	AP001107.8	0.414797165	7.91E-24	postive
SETD1B	AP001107.8	0.426738886	2.90E-25	postive
ALOX12	AP001107.8	0.479661517	2.31E-32	postive
TAZ	AP001107.8	0.495779436	8.80E-35	postive
ZNF419	AL359091.3	0.409948971	2.92E-23	postive
TUBE1	AL359091.3	0.419759579	2.03E-24	postive
ALOX12	AL359091.3	0.450263678	2.89E-28	postive
TFAP2C	AL359091.3	0.414344049	8.95E-24	postive
VEGFA	AC021683.1	0.413569466	1.10E-23	postive
HIC1	AC021683.1	0.414784516	7.94E-24	postive
ZEB1	AC021683.1	0.515823388	5.62E-38	postive
EPAS1	AC021683.1	0.491085429	4.60E-34	postive
FANCD2	AC007728.2	0.428287368	1.87E-25	postive
PML	AC007728.2	0.487537516	1.58E-33	postive
GCH1	AC007728.2	0.549177931	8.67E-44	postive
NCF2	AC007728.2	0.414223688	9.24E-24	postive
CYBB	AC007728.2	0.459753684	1.52E-29	postive
IFNG	AC007728.2	0.669095824	3.07E-71	postive
TNFAIP3	AC007728.2	0.523402997	3.06E-39	postive
HELLS	AC005838.2	0.587696905	2.28E-51	postive
TUBE1	AC005838.2	0.414374805	8.87E-24	postive
ALOX12	AC005838.2	0.502088044	9.16E-36	postive
GABPB1	AC005838.2	0.505250937	2.89E-36	postive
DUOX1	AC005838.2	0.402688459	1.98E-22	postive
ATG7	AC005838.2	0.409288093	3.48E-23	postive
LINC00472	AC005838.2	0.511239046	3.16E-37	postive
ATM	AC005838.2	0.655045779	2.28E-67	postive
FBXW7	AC005838.2	0.468245896	1.01E-30	postive
TFAP2C	AC096733.2	0.596343209	3.26E-53	postive
HBA1	AC096733.2	0.49054052	5.56E-34	postive
LPIN1	AC096733.2	0.459046135	1.90E-29	postive
ACO1	AC092295.2	0.473948728	1.56E-31	postive
BAP1	AC092295.2	0.469680428	6.31E-31	postive
FANCD2	AC002456.1	0.433534621	4.16E-26	postive
JDP2	AC002456.1	0.412796582	1.36E-23	postive
AURKA	AC002456.1	0.412461877	1.49E-23	postive
PHKG2	AC093458.1	0.462556628	6.25E-30	postive
TAZ	AC093458.1	0.416199582	5.40E-24	postive
TRIB3	AL117332.1	0.402689866	1.98E-22	postive
CARS1	AL117332.1	0.436236611	1.90E-26	postive
SIRT1	AL117332.1	-0.41737132	3.92E-24	negative
PHKG2	AL138960.1	0.410181195	2.74E-23	postive
TAZ	AL138960.1	0.48122616	1.37E-32	postive
MUC1	AC023669.2	0.443967369	1.94E-27	postive
SLC7A5	AC023669.2	0.468975981	7.93E-31	postive
SLC2A1	AC023669.2	0.480491666	1.75E-32	postive
GPX4	AC007406.4	-0.408062577	4.82E-23	negative
RB1	AC007406.4	0.569741568	1.04E-47	postive
KLHL24	AC007406.4	0.4660334	2.05E-30	postive

TUBE1	AC007406.4	0.411474695	1.94E-23	postive
MAP3K5	AC007406.4	0.46931664	7.10E-31	postive
IREB2	AC007406.4	0.589435521	9.80E-52	postive
HMGB1	AC007406.4	0.446429352	9.24E-28	postive
SP1	AC007406.4	0.604801432	4.51E-55	postive
GABPB1	AC007406.4	0.528202039	4.66E-40	postive
PIK3CA	AC007406.4	0.525549887	1.32E-39	postive
KRAS	AC007406.4	0.581580733	4.26E-50	postive
ZEB1	AC007406.4	0.479420298	2.51E-32	postive
MAPK8	AC007406.4	0.565510751	7.00E-47	postive
LINC00472	AC007406.4	0.454985275	6.74E-29	postive
PRKAA2	AC007406.4	0.487672533	1.51E-33	postive
PRKAA1	AC007406.4	0.582943389	2.23E-50	postive
ATM	AC007406.4	0.532062762	1.00E-40	postive
SIRT1	AC007406.4	0.516910579	3.72E-38	postive
HELLS	TPM1-AS	0.449787492	3.34E-28	postive
TUBE1	TPM1-AS	0.503610608	5.27E-36	postive
ALOX12	TPM1-AS	0.514435896	9.50E-38	postive
GABPB1	TPM1-AS	0.507322506	1.35E-36	postive
LINC00472	TPM1-AS	0.60297131	1.15E-54	postive
ATM	TPM1-AS	0.691348495	7.93E-78	postive
FBXW7	TPM1-AS	0.455941959	5.01E-29	postive
ALOX12	AC092718.4	0.448059293	5.64E-28	postive
TFAP2C	AC092718.4	0.405381663	9.79E-23	postive
HELLS	AL138689.1	0.428531595	1.74E-25	postive
ZNF419	AL138689.1	0.416091876	5.56E-24	postive
KLHL24	AL138689.1	0.44969686	3.43E-28	postive
TUBE1	AL138689.1	0.433809716	3.84E-26	postive
GABPB1	AL138689.1	0.574365002	1.25E-48	postive
PIK3CA	AL138689.1	0.414664659	8.20E-24	postive
MAPK8	AL138689.1	0.447793196	6.12E-28	postive
LINC00472	AL138689.1	0.805158537	6.08E-124	postive
ATM	AL138689.1	0.564150372	1.29E-46	postive
FBXW7	AL138689.1	0.471282186	3.74E-31	postive
TUBE1	EIF3J-DT	0.450069546	3.06E-28	postive
ALOX12	EIF3J-DT	0.499137459	2.65E-35	postive
MAPK8	EIF3J-DT	0.407864351	5.08E-23	postive
ZNF419	AL021878.2	0.541996804	1.76E-42	postive
VEGFA	AL021878.2	0.403633178	1.55E-22	postive
TUBE1	AL021878.2	0.534337527	4.02E-41	postive
SETD1B	AL021878.2	0.506201232	2.04E-36	postive
ALOX12	AL021878.2	0.530250164	2.07E-40	postive
LINC00472	AL021878.2	0.44872645	4.61E-28	postive
ATM	AL021878.2	0.4725097	2.50E-31	postive
HELLS	AC011466.1	0.467865773	1.14E-30	postive
ZNF419	AC011466.1	0.478684931	3.21E-32	postive
TUBE1	AC011466.1	0.577191015	3.35E-49	postive
ALOX12	AC011466.1	0.577839308	2.48E-49	postive
GABPB1	AC011466.1	0.52999885	2.28E-40	postive
ATM	AC011466.1	0.646045699	5.40E-65	postive
FBXW7	AC011466.1	0.537712782	1.02E-41	postive
HELLS	PAX8-AS1	0.485504206	3.18E-33	postive
TUBE1	PAX8-AS1	0.408595782	4.18E-23	postive
GABPB1	PAX8-AS1	0.535606124	2.40E-41	postive
ATG7	PAX8-AS1	0.40942932	3.35E-23	postive
LINC00472	PAX8-AS1	0.729544595	1.19E-90	postive
ATM	PAX8-AS1	0.629564159	7.54E-61	postive
FBXW7	PAX8-AS1	0.422064427	1.07E-24	postive

OXSR1	AC009486.1	0.418503839	2.88E-24	postive
VEGFA	AC009486.1	0.53218305	9.55E-41	postive
SETD1B	AC009486.1	0.400532598	3.47E-22	postive
FTL	AC009486.1	-0.402073994	2.32E-22	negative
ZEB1	AC009486.1	0.581407718	4.62E-50	postive
MAPK8	AC009486.1	0.546389007	2.81E-43	postive
EPAS1	AC009486.1	0.499139498	2.65E-35	postive
ATM	AC009486.1	0.524182701	2.26E-39	postive
YY1AP1	AC009486.1	0.403136354	1.76E-22	postive
SIRT1	AC009486.1	0.43102802	8.55E-26	postive
FBXW7	AC009486.1	0.41125221	2.06E-23	postive
HSF1	SNHG17	0.452562132	1.43E-28	postive
ATF4	SNHG17	0.464756664	3.10E-30	postive
SCP2	SNHG17	-0.433071921	4.75E-26	negative
CARS1	SNHG17	0.405332488	9.91E-23	postive
NCOA4	SNHG17	-0.509008116	7.24E-37	negative
PHKG2	SNHG17	0.571215653	5.29E-48	postive
MAPK1	SNHG17	-0.423020977	8.23E-25	negative
BID	SNHG17	0.407403636	5.74E-23	postive
TAZ	SNHG17	0.60593511	2.52E-55	postive
OXSR1	AC018647.2	0.436486324	1.76E-26	postive
IREB2	AC018647.2	0.441156655	4.47E-27	postive
MAPK8	AC018647.2	0.471487715	3.50E-31	postive
LINC00472	AL591926.2	0.485961834	2.72E-33	postive
HBA1	AC142472.1	0.406975538	6.43E-23	postive
HRAS	AC142472.1	0.648675757	1.11E-65	postive
PHKG2	AC142472.1	0.470128818	5.45E-31	postive
EGLN2	AC142472.1	0.705869656	1.85E-82	postive
TAZ	AC142472.1	0.524940498	1.68E-39	postive
VEGFA	AL596442.2	0.410676069	2.40E-23	postive
HELLS	AC087222.1	0.420198772	1.80E-24	postive
ZNF419	AC087222.1	0.473225587	1.97E-31	postive
TUBE1	AC087222.1	0.512679131	1.84E-37	postive
SETD1B	AC087222.1	0.420652588	1.59E-24	postive
ALOX12	AC087222.1	0.642299955	4.97E-64	postive
GABPB1	AC087222.1	0.468420547	9.50E-31	postive
MAPK8	AC087222.1	0.414471521	8.64E-24	postive
LINC00472	AC087222.1	0.503639482	5.21E-36	postive
ATM	AC087222.1	0.572435889	3.03E-48	postive
FBXW7	AC087222.1	0.466906378	1.55E-30	postive
HSPB1	CPNE8-AS1	0.409318646	3.45E-23	postive
HRAS	CPNE8-AS1	0.444855589	1.48E-27	postive
PHKG2	CPNE8-AS1	0.409075627	3.68E-23	postive
TAZ	CPNE8-AS1	0.416106328	5.54E-24	postive
ZNF419	LINC00921	0.48356395	6.17E-33	postive
VEGFA	LINC00921	0.512209957	2.20E-37	postive
TUBE1	LINC00921	0.488212764	1.25E-33	postive
SETD1B	LINC00921	0.457096094	3.49E-29	postive
ALOX12	LINC00921	0.638429718	4.78E-63	postive
TAZ	LINC00921	0.517465426	3.01E-38	postive
HMGB1	AC006213.4	0.425142994	4.54E-25	postive
GABPB1	AC006213.4	0.464077681	3.85E-30	postive
KRAS	AC006213.4	0.412818218	1.35E-23	postive
MAPK8	AC006213.4	0.425433427	4.19E-25	postive
LINC00472	AC006213.4	0.407361623	5.80E-23	postive
PRKAA1	AC006213.4	0.438928487	8.63E-27	postive
ATM	AC006213.4	0.427635252	2.25E-25	postive
HELLS	AC009120.3	0.438993005	8.47E-27	postive

ZNF419	AC009120.3	0.523287929	3.20E-39	postive
TUBE1	AC009120.3	0.571215606	5.29E-48	postive
SETD1B	AC009120.3	0.48599119	2.69E-33	postive
ALOX12	AC009120.3	0.674515655	8.62E-73	postive
GABPB1	AC009120.3	0.453866676	9.53E-29	postive
LINC00472	AC009120.3	0.504643878	3.61E-36	postive
ATM	AC009120.3	0.574083633	1.42E-48	postive
FBXW7	AC009120.3	0.482279746	9.55E-33	postive
JDP2	AC109479.1	0.441862028	3.63E-27	postive
HELLS	C2orf49-DT	0.420995625	1.45E-24	postive
ZNF419	C2orf49-DT	0.539002152	6.03E-42	postive
VEGFA	C2orf49-DT	0.505305888	2.83E-36	postive
TUBE1	C2orf49-DT	0.659884755	1.12E-68	postive
SETD1B	C2orf49-DT	0.465856613	2.17E-30	postive
DRD4	C2orf49-DT	0.420962954	1.46E-24	postive
ALOX12	C2orf49-DT	0.61304714	6.13E-57	postive
GABPB1	C2orf49-DT	0.45637603	4.38E-29	postive
PHKG2	C2orf49-DT	0.463262715	4.99E-30	postive
ATM	C2orf49-DT	0.453843454	9.60E-29	postive
TAZ	C2orf49-DT	0.62891994	1.08E-60	postive
FBXW7	C2orf49-DT	0.410196155	2.73E-23	postive
TFAP2C	AC008946.1	0.551498364	3.23E-44	postive
HBA1	AC008946.1	0.479019921	2.87E-32	postive
HELLS	AL513190.1	0.530361168	1.98E-40	postive
ZNF419	AL513190.1	0.494500804	1.38E-34	postive
TUBE1	AL513190.1	0.546534995	2.64E-43	postive
ALOX12	AL513190.1	0.631246395	2.92E-61	postive
GABPB1	AL513190.1	0.580988589	5.64E-50	postive
MAPK8	AL513190.1	0.417780184	3.51E-24	postive
LINC00472	AL513190.1	0.658238707	3.14E-68	postive
ATM	AL513190.1	0.698814276	3.57E-80	postive
FBXW7	AL513190.1	0.55973018	9.11E-46	postive
HELLS	AC012676.3	0.496206354	7.56E-35	postive
ZNF419	AC012676.3	0.439683259	6.91E-27	postive
TUBE1	AC012676.3	0.532655448	7.90E-41	postive
ALOX12	AC012676.3	0.533670227	5.26E-41	postive
GABPB1	AC012676.3	0.531427994	1.29E-40	postive
LINC00472	AC012676.3	0.55893825	1.29E-45	postive
ATM	AC012676.3	0.617263577	6.47E-58	postive
FBXW7	AC012676.3	0.535056525	3.00E-41	postive
HSPB1	LINC01569	0.406659818	6.99E-23	postive
ALOX12	LINC01569	0.413863306	1.02E-23	postive
PHKG2	LINC01569	0.514503363	9.26E-38	postive
TAZ	LINC01569	0.592568814	2.12E-52	postive
HSPB1	AL133353.1	0.412547432	1.45E-23	postive
HELLS	AC008750.4	0.493282263	2.13E-34	postive
ZNF419	AC008750.4	0.509737547	5.52E-37	postive
TUBE1	AC008750.4	0.595410251	5.19E-53	postive
SETD1B	AC008750.4	0.418060018	3.25E-24	postive
ALOX12	AC008750.4	0.70454626	5.03E-82	postive
GABPB1	AC008750.4	0.507130189	1.45E-36	postive
MAPK8	AC008750.4	0.42005916	1.87E-24	postive
LINC00472	AC008750.4	0.604956529	4.17E-55	postive
ATM	AC008750.4	0.708769626	2.03E-83	postive
FBXW7	AC008750.4	0.459612226	1.59E-29	postive
HELLS	AP001381.1	0.469845217	5.98E-31	postive
TFAP2C	AP001381.1	0.490459416	5.72E-34	postive
HBA1	AP001381.1	0.500891773	1.41E-35	postive

DUOX1	AP001381.1	0.440444874	5.52E-27	postive
MTOR	AC073569.2	0.490123366	6.43E-34	postive
IREB2	AC073569.2	0.424325033	5.72E-25	postive
PIK3CA	AC073569.2	0.410178498	2.74E-23	postive
ATM	AC073569.2	0.421800356	1.16E-24	postive
FANCD2	LINC00941	0.549314483	8.18E-44	postive
RGS4	LINC00941	0.462484564	6.39E-30	postive
STMN1	LINC00941	0.473633115	1.73E-31	postive
RRM2	LINC00941	0.616931095	7.74E-58	postive
AURKA	LINC00941	0.569580036	1.12E-47	postive
SLC1A5	LINC00941	0.501161422	1.28E-35	postive
CARS1	LINC00941	0.433872147	3.77E-26	postive
CDKN2A	LINC00941	0.449756317	3.37E-28	postive
PANX1	LINC00941	0.519945455	1.16E-38	postive
ISCU	GATA2-AS1	0.543342497	1.00E-42	postive
ACSL3	GATA2-AS1	0.404902576	1.11E-22	postive
KLHL24	GATA2-AS1	0.456493875	4.22E-29	postive
ATG4D	GATA2-AS1	0.476843494	5.95E-32	postive
MAP1LC3A	GATA2-AS1	0.426274868	3.30E-25	postive
GABARAPL2	GATA2-AS1	0.42898205	1.53E-25	postive
GABARAPL1	GATA2-AS1	0.632647621	1.32E-61	postive
BAP1	GATA2-AS1	0.445606522	1.18E-27	postive
LPIN1	GATA2-AS1	0.512178208	2.22E-37	postive
ALOX12	AC138150.2	0.418353834	3.00E-24	postive
PHKG2	AC138150.2	0.410497354	2.52E-23	postive
TAZ	AC138150.2	0.477402821	4.94E-32	postive
HELLS	DGCR11	0.432089234	6.31E-26	postive
LINC00472	DGCR11	0.426505786	3.10E-25	postive
ATM	DGCR11	0.436218636	1.91E-26	postive
EIF2S1	KTN1-AS1	0.443130244	2.49E-27	postive
TUBE1	AC114757.1	0.400216781	3.76E-22	postive
GABPB1	AC114757.1	0.513956443	1.14E-37	postive
ZEB1	AC114757.1	0.436691167	1.66E-26	postive
MAPK8	AC114757.1	0.413277263	1.19E-23	postive
LINC00472	AC114757.1	0.441994109	3.49E-27	postive
ATM	AC114757.1	0.492204823	3.11E-34	postive
FBXW7	AC114757.1	0.400497019	3.50E-22	postive
HELLS	RRN3P2	0.507366894	1.33E-36	postive
TUBE1	RRN3P2	0.530927145	1.58E-40	postive
ALOX12	RRN3P2	0.537245413	1.24E-41	postive
GABPB1	RRN3P2	0.540410033	3.38E-42	postive
ATM	RRN3P2	0.602617496	1.38E-54	postive
TAZ	RRN3P2	0.408333906	4.49E-23	postive
FBXW7	RRN3P2	0.568489266	1.83E-47	postive
HELLS	AP001160.4	0.524091234	2.34E-39	postive
ZNF419	AP001160.4	0.448756297	4.57E-28	postive
TUBE1	AP001160.4	0.449631446	3.50E-28	postive
ALOX12	AP001160.4	0.494065464	1.61E-34	postive
TFAP2C	AP001160.4	0.41162874	1.86E-23	postive
GABPB1	AP001160.4	0.446566727	8.86E-28	postive
FBXW7	AP001160.4	0.448321856	5.21E-28	postive
HELLS	AC092279.1	0.522032941	5.20E-39	postive
ZNF419	AC092279.1	0.424590967	5.31E-25	postive
KLHL24	AC092279.1	0.471441071	3.55E-31	postive
TUBE1	AC092279.1	0.460043618	1.39E-29	postive
ALOX12	AC092279.1	0.450058665	3.07E-28	postive
IREB2	AC092279.1	0.470510434	4.81E-31	postive
GABPB1	AC092279.1	0.567945915	2.34E-47	postive

PIK3CA	AC092279.1	0.437094432	1.48E-26	postive
ATG7	AC092279.1	0.44417433	1.82E-27	postive
MAPK8	AC092279.1	0.443093733	2.51E-27	postive
LINC00472	AC092279.1	0.772920891	3.59E-108	postive
LPIN1	AC092279.1	0.426978056	2.71E-25	postive
ATM	AC092279.1	0.650486378	3.73E-66	postive
FBXW7	AC092279.1	0.451116027	2.22E-28	postive
LINC00472	AL355472.2	0.493089952	2.28E-34	postive
PCK2	TMEM246-A	0.482472881	8.95E-33	postive
MIOX	TMEM246-A	0.555880136	4.89E-45	postive
TUBE1	AL591895.1	0.410248074	2.69E-23	postive
ALOX12	AL591895.1	0.429675486	1.26E-25	postive
PHKG2	AL591895.1	0.435077141	2.66E-26	postive
TAZ	AL591895.1	0.516356202	4.59E-38	postive
FANCD2	AC107081.1	0.435514187	2.34E-26	postive
HELLS	AC107081.1	0.543830944	8.20E-43	postive
ZNF419	AC107081.1	0.478818163	3.07E-32	postive
TUBE1	AC107081.1	0.489353384	8.41E-34	postive
ALOX12	AC107081.1	0.529882348	2.39E-40	postive
GABPB1	AC107081.1	0.441926756	3.56E-27	postive
PHKG2	AC107081.1	0.414921829	7.65E-24	postive
TAZ	AC107081.1	0.480087851	2.01E-32	postive
FBXW7	AC107081.1	0.492153918	3.16E-34	postive
ZNF419	AC087500.2	0.400156398	3.82E-22	postive
VEGFA	AC087500.2	0.401808297	2.49E-22	postive
TUBE1	AC087500.2	0.418349493	3.00E-24	postive
DRD4	AC087500.2	0.423357391	7.49E-25	postive
ALOX12	AC087500.2	0.564633479	1.04E-46	postive
PHKG2	AC087500.2	0.408393386	4.42E-23	postive
TAZ	AC087500.2	0.529341215	2.96E-40	postive
SLC3A2	LINC00957	0.404836407	1.13E-22	postive
ISCU	LINC00957	0.480726377	1.62E-32	postive
DDIT3	LINC00957	0.442305947	3.18E-27	postive
ATG4D	LINC00957	0.430173118	1.09E-25	postive
GABARAPL1	LINC00957	0.452415299	1.49E-28	postive
ALOX12	AL358781.2	0.469746778	6.17E-31	postive
TAZ	AL358781.2	0.514382328	9.70E-38	postive
TUBE1	CCNT2-AS1	0.432844373	5.08E-26	postive
ALOX12	CCNT2-AS1	0.458345554	2.36E-29	postive
GABPB1	CCNT2-AS1	0.432964277	4.90E-26	postive
MAPK8	CCNT2-AS1	0.45307932	1.22E-28	postive
ATM	CCNT2-AS1	0.513547829	1.33E-37	postive
HSPB1	AC087239.1	0.416991049	4.35E-24	postive
HSF1	AC087239.1	0.410279174	2.67E-23	postive
DRD4	AC087239.1	0.453020915	1.24E-28	postive
HRAS	AC087239.1	0.504721249	3.51E-36	postive
NCOA4	AC087239.1	-0.445758814	1.13E-27	negative
PHKG2	AC087239.1	0.566141399	5.28E-47	postive
MAP1LC3A	AC087239.1	0.411237973	2.07E-23	postive
EGLN2	AC087239.1	0.467687735	1.20E-30	postive
TAZ	AC087239.1	0.673502075	1.69E-72	postive
TFAP2C	EDIL3-DT	0.429529241	1.31E-25	postive
HBA1	EDIL3-DT	0.448416691	5.06E-28	postive
LINC00472	NHS-AS1	0.444083207	1.87E-27	postive
ZNF419	TBX2-AS1	0.430144061	1.10E-25	postive
HRAS	TBX2-AS1	0.523177767	3.34E-39	postive
MAP1LC3A	TBX2-AS1	0.401441244	2.74E-22	postive
EGLN2	TBX2-AS1	0.602587551	1.40E-54	postive

TAZ	TBX2-AS1	0.500896128	1.41E-35	postive
ASNS	Z95115.1	0.457828862	2.78E-29	postive
HELLS	AC011676.1	0.479960491	2.09E-32	postive
GABPB1	AC011676.1	0.470158674	5.40E-31	postive
LINC00472	AC011676.1	0.576513574	4.60E-49	postive
ATM	AC011676.1	0.618114157	4.10E-58	postive
FBXW7	AC011676.1	0.41034583	2.62E-23	postive
RB1	AC009318.3	0.408603437	4.18E-23	postive
HELLS	AC009318.3	0.46462735	3.23E-30	postive
GABPB1	AC009318.3	0.439060527	8.30E-27	postive
ACSL4	AC009318.3	0.404980496	1.09E-22	postive
KRAS	AC009318.3	0.448728036	4.61E-28	postive
LINC00472	AC009318.3	0.44921905	3.97E-28	postive
TGFBR1	AC009318.3	0.424996041	4.74E-25	postive
ATM	AC009318.3	0.489611608	7.69E-34	postive
FBXW7	AC009318.3	0.443349225	2.33E-27	postive
BACH1	AC009318.3	0.471675619	3.29E-31	postive
ZNF419	AL117336.1	0.427099432	2.62E-25	postive
TUBE1	AL117336.1	0.471256033	3.77E-31	postive
GABPB1	AL117336.1	0.502514105	7.85E-36	postive
LINC00472	AL117336.1	0.526210421	1.02E-39	postive
ATM	AL117336.1	0.436514115	1.75E-26	postive
FBXW7	AL117336.1	0.476539998	6.58E-32	postive
RB1	AC005332.6	0.514986655	7.72E-38	postive
HSPB1	AC005332.6	-0.412313936	1.55E-23	negative
IREB2	AC005332.6	0.467960639	1.10E-30	postive
SP1	AC005332.6	0.487186664	1.78E-33	postive
NRAS	AC005332.6	0.453008311	1.24E-28	postive
ULK2	AC005332.6	0.465125397	2.75E-30	postive
MAPK1	AC005332.6	0.450344625	2.81E-28	postive
ZEB1	AC005332.6	0.442128885	3.35E-27	postive
MAPK8	AC005332.6	0.512406683	2.04E-37	postive
PRKAA2	AC005332.6	0.430036609	1.14E-25	postive
EPAS1	AC005332.6	0.405100781	1.05E-22	postive
SIRT1	AC005332.6	0.546831573	2.33E-43	postive
PEBP1	AC093010.2	0.400310858	3.67E-22	postive
EPAS1	AC097639.1	0.495599977	9.38E-35	postive
HELLS	AL031666.1	0.430848935	9.01E-26	postive
GABPB1	AL031666.1	0.431425792	7.63E-26	postive
ATG7	AL031666.1	0.416846508	4.53E-24	postive
LINC00472	AL031666.1	0.52607589	1.08E-39	postive
ATM	AL031666.1	0.564703525	1.00E-46	postive
EGLN2	AP005432.2	0.409445817	3.34E-23	postive
TUBE1	AC109347.1	0.443702524	2.09E-27	postive
PHKG2	AL121782.1	0.480091855	2.00E-32	postive
TAZ	AL121782.1	0.509568654	5.88E-37	postive
HELLS	AC091887.1	0.538900253	6.28E-42	postive
ZNF419	AC091887.1	0.523002187	3.57E-39	postive
TUBE1	AC091887.1	0.538756253	6.67E-42	postive
ALOX12	AC091887.1	0.597946659	1.46E-53	postive
GABPB1	AC091887.1	0.524766743	1.80E-39	postive
MAPK8	AC091887.1	0.41202054	1.68E-23	postive
LINC00472	AC091887.1	0.481746919	1.14E-32	postive
ATM	AC091887.1	0.63626147	1.67E-62	postive
FBXW7	AC091887.1	0.58185633	3.74E-50	postive
ACSL3	AC008124.1	0.416897408	4.47E-24	postive
ZNF419	AC008124.1	0.498319543	3.56E-35	postive
KLHL24	AC008124.1	0.566303622	4.90E-47	postive

TUBE1	AC008124.1	0.549958444	6.22E-44	postive
ALOX12	AC008124.1	0.439101167	8.20E-27	postive
IREB2	AC008124.1	0.494664494	1.31E-34	postive
GABPB1	AC008124.1	0.61714047	6.92E-58	postive
EMC2	AC008124.1	0.40212375	2.29E-22	postive
PIK3CA	AC008124.1	0.438530312	9.70E-27	postive
KRAS	AC008124.1	0.481808464	1.12E-32	postive
MAPK8	AC008124.1	0.499274337	2.53E-35	postive
LINC00472	AC008124.1	0.759038565	3.67E-102	postive
PRKAA1	AC008124.1	0.451204358	2.16E-28	postive
LPIN1	AC008124.1	0.48779747	1.44E-33	postive
ATM	AC008124.1	0.52318425	3.33E-39	postive
VEGFA	AP001625.2	0.449690314	3.44E-28	postive
TUBE1	AP001625.2	0.428344824	1.84E-25	postive
ALOX12	AP001625.2	0.52615225	1.04E-39	postive
MAPK8	AP001625.2	0.459478346	1.66E-29	postive
ATM	AP001625.2	0.503302436	5.89E-36	postive
ALOX12	AC011346.1	0.409457568	3.33E-23	postive
ATM	AC011346.1	0.516834505	3.83E-38	postive
IREB2	MKLN1-AS	0.407778589	5.20E-23	postive
ACSL4	LINC01929	0.419545815	2.16E-24	postive
HRAS	AL732292.2	0.487212436	1.77E-33	postive
BRD4	AL021707.3	0.47307151	2.08E-31	postive
ZNF419	AL021707.3	0.504416885	3.92E-36	postive
VEGFA	AL021707.3	0.483962232	5.39E-33	postive
TUBE1	AL021707.3	0.524699859	1.84E-39	postive
SETD1B	AL021707.3	0.56211827	3.17E-46	postive
ALOX12	AL021707.3	0.626691976	3.75E-60	postive
ATM	AL021707.3	0.423909532	6.42E-25	postive
YY1AP1	AL021707.3	0.446895941	8.02E-28	postive
TAZ	AL021707.3	0.560989886	5.23E-46	postive
HELLS	LINC00476	0.436447692	1.78E-26	postive
ZNF419	LINC00476	0.404341838	1.29E-22	postive
TUBE1	LINC00476	0.441024477	4.65E-27	postive
ALOX12	LINC00476	0.522516766	4.31E-39	postive
GABPB1	LINC00476	0.462164844	7.08E-30	postive
ATM	LINC00476	0.481146655	1.40E-32	postive
FBXW7	LINC00476	0.413643069	1.08E-23	postive
TUBE1	LINC02532	0.609199366	4.63E-56	postive
ALOX12	LINC02532	0.503882064	4.77E-36	postive
PRKAA2	LINC02532	0.432279471	5.97E-26	postive
ATM	LINC02532	0.625771993	6.25E-60	postive
ALOX12	AL031848.1	0.500117005	1.87E-35	postive
TFAP2C	AC027237.2	0.570461707	7.47E-48	postive
HBA1	AC027237.2	0.499914451	2.01E-35	postive
DUOX1	AC027237.2	0.423250986	7.72E-25	postive
NCOA4	HOTAIRM1	-0.430374166	1.03E-25	negative
PHKG2	HOTAIRM1	0.646588707	3.90E-65	postive
SAT1	HOTAIRM1	0.40829032	4.54E-23	postive
MAPK1	HOTAIRM1	-0.434411822	3.23E-26	negative
TAZ	HOTAIRM1	0.642159793	5.40E-64	postive
PTGS2	AC002480.1	0.440886052	4.84E-27	postive
IL6	AC002480.1	0.702491162	2.35E-81	postive
NCF2	AP002954.1	0.406798114	6.74E-23	postive
FTL	AP002954.1	0.469433208	6.84E-31	postive
HELLS	MIR181A2HG	0.52705001	7.33E-40	postive
ZNF419	MIR181A2HG	0.4701894	5.34E-31	postive
GABPB1	MIR181A2HG	0.4871756	1.79E-33	postive

ATM	MIR181A2HC	0.412579102	1.44E-23	postive
ZNF419	AL035530.2	0.441902191	3.58E-27	postive
KLHL24	AL035530.2	0.535153222	2.89E-41	postive
TUBE1	AL035530.2	0.547675858	1.64E-43	postive
ALOX12	AL035530.2	0.408270345	4.56E-23	postive
GABPB1	AL035530.2	0.50379541	4.92E-36	postive
PIK3CA	AL035530.2	0.421710124	1.19E-24	postive
MAPK8	AL035530.2	0.407575859	5.48E-23	postive
LINC00472	AL035530.2	0.797385065	7.03E-120	postive
ATM	AL035530.2	0.522813746	3.84E-39	postive
FBXW7	AL035530.2	0.435290952	2.50E-26	postive
MTOR	GNG12-AS1	0.430804265	9.12E-26	postive
KLHL24	GNG12-AS1	0.581783273	3.87E-50	postive
TUBE1	GNG12-AS1	0.419323077	2.29E-24	postive
MAP3K5	GNG12-AS1	0.419778097	2.02E-24	postive
ALOX12	GNG12-AS1	0.41160517	1.87E-23	postive
IREB2	GNG12-AS1	0.608324369	7.30E-56	postive
GABPB1	GNG12-AS1	0.422529608	9.44E-25	postive
PIK3CA	GNG12-AS1	0.525570502	1.31E-39	postive
KRAS	GNG12-AS1	0.421119127	1.40E-24	postive
MAPK8	GNG12-AS1	0.529594117	2.68E-40	postive
LINC00472	GNG12-AS1	0.661219419	4.82E-69	postive
PRKAA2	GNG12-AS1	0.521736051	5.84E-39	postive
PRKAA1	GNG12-AS1	0.465791943	2.22E-30	postive
ATM	GNG12-AS1	0.645358571	8.13E-65	postive
KLHL24	AC010210.1	0.594988177	6.40E-53	postive
GABPB1	AC010210.1	0.461349044	9.17E-30	postive
PIK3CA	AC010210.1	0.437007857	1.52E-26	postive
LINC00472	AC010210.1	0.911523857	2.24E-209	postive
LPIN1	AC010210.1	0.47393114	1.56E-31	postive
ATM	AC010210.1	0.462908697	5.59E-30	postive
VEGFA	AP001189.1	0.438721377	9.17E-27	postive
SETD1B	AP001189.1	0.406295955	7.69E-23	postive
HIC1	AP001189.1	0.446963452	7.86E-28	postive
ZEB1	AP001189.1	0.571571118	4.50E-48	postive
EPAS1	AP001189.1	0.524872296	1.72E-39	postive
FANCD2	AC009950.1	0.449918017	3.21E-28	postive
HELLS	AC009950.1	0.475114196	1.06E-31	postive
ALOX12	AC009950.1	0.57144993	4.76E-48	postive
HBA1	AC009950.1	0.472491071	2.51E-31	postive
GABPB1	AC009950.1	0.414458606	8.67E-24	postive
DUOX1	AC009950.1	0.400658226	3.35E-22	postive
ATM	AC009950.1	0.482432206	9.07E-33	postive
FBXW7	AC009950.1	0.459289445	1.76E-29	postive
HELLS	AL022157.1	0.567178756	3.31E-47	postive
ZNF419	AL022157.1	0.406262526	7.76E-23	postive
TUBE1	AL022157.1	0.4946305	1.32E-34	postive
ALOX12	AL022157.1	0.536294621	1.82E-41	postive
GABPB1	AL022157.1	0.488773181	1.03E-33	postive
LINC00472	AL022157.1	0.597472398	1.86E-53	postive
ATM	AL022157.1	0.5930257	1.69E-52	postive
HELLS	AC007216.3	0.409765317	3.06E-23	postive
TFAP2C	AC007216.3	0.51916771	1.57E-38	postive
HBA1	AC007216.3	0.469839326	5.99E-31	postive
DUOX1	AC007216.3	0.419777856	2.02E-24	postive
LINC00472	AC007216.3	0.442108611	3.37E-27	postive
ZNF419	GUSBP11	0.403501744	1.60E-22	postive
VEGFA	GUSBP11	0.433429388	4.29E-26	postive

TUBE1	GUSBP11	0.563351299	1.84E-46	postive
SETD1B	GUSBP11	0.419894295	1.96E-24	postive
ALOX12	GUSBP11	0.554908582	7.45E-45	postive
PHKG2	GUSBP11	0.521372441	6.72E-39	postive
TAZ	GUSBP11	0.663323379	1.27E-69	postive
ALOX12	DNAJC27-AS	0.40657668	7.14E-23	postive
TFAP2C	DNAJC27-AS	0.475601617	9.00E-32	postive
HBA1	DNAJC27-AS	0.567529058	2.82E-47	postive
DUOX1	DNAJC27-AS	0.44119446	4.42E-27	postive
ALOX12B	DNAJC27-AS	0.404466565	1.24E-22	postive
AKR1C1	AL353751.1	0.470571339	4.72E-31	postive
AKR1C2	AL353751.1	0.54938629	7.93E-44	postive
NQO1	AL353751.1	0.436073733	1.99E-26	postive
ATP6V1G2	AC006449.5	0.403699193	1.52E-22	postive
WIPI1	AC006449.5	0.489508913	7.97E-34	postive
TFAP2C	AC130469.1	0.564684319	1.01E-46	postive
HBA1	AC130469.1	0.512140561	2.25E-37	postive
DUOX1	AC130469.1	0.408088523	4.79E-23	postive
HELLS	AL442125.1	0.565041507	8.64E-47	postive
BRD4	AL442125.1	0.401590104	2.64E-22	postive
ZNF419	AL442125.1	0.468880314	8.18E-31	postive
TUBE1	AL442125.1	0.49478215	1.25E-34	postive
ALOX12	AL442125.1	0.49408346	1.60E-34	postive
GABPB1	AL442125.1	0.582273638	3.07E-50	postive
LINC00472	AL442125.1	0.651995651	1.49E-66	postive
ATM	AL442125.1	0.584807742	9.16E-51	postive
FBXW7	AL442125.1	0.499666675	2.19E-35	postive
SLC3A2	AC116407.1	0.443315278	2.35E-27	postive
ATG4D	AC116407.1	0.446227794	9.82E-28	postive
HELLS	AC008759.2	0.486135142	2.56E-33	postive
ZNF419	AC008759.2	0.424866779	4.91E-25	postive
TUBE1	AC008759.2	0.464671678	3.18E-30	postive
ALOX12	AC008759.2	0.55451169	8.84E-45	postive
GABPB1	AC008759.2	0.445689428	1.15E-27	postive
ATM	AC008759.2	0.462368929	6.63E-30	postive
FBXW7	AC008759.2	0.45450566	7.82E-29	postive
LPIN1	LINC01106	0.404604207	1.20E-22	postive
VEGFA	AP000355.1	0.531521775	1.24E-40	postive
TUBE1	AP000355.1	0.464415384	3.45E-30	postive
ALOX12	AP000355.1	0.573083342	2.25E-48	postive
ATM	AP000355.1	0.491490391	3.99E-34	postive
HELLS	AC099811.4	0.510022034	4.97E-37	postive
KLHL24	AC099811.4	0.433438915	4.28E-26	postive
ALOX12	AC099811.4	0.400328975	3.65E-22	postive
GABPB1	AC099811.4	0.520334096	1.00E-38	postive
LINC00472	AC099811.4	0.793718503	5.04E-118	postive
ATM	AC099811.4	0.573010765	2.32E-48	postive
FBXW7	AC099811.4	0.425736873	3.85E-25	postive
ZNF419	AL513320.1	0.416487259	4.99E-24	postive
TUBE1	AL513320.1	0.419270953	2.33E-24	postive
DRD4	AL513320.1	0.405970268	8.38E-23	postive
ALOX12	AL513320.1	0.537025641	1.35E-41	postive
NCOA4	AL513320.1	-0.420289173	1.76E-24	negative
PHKG2	AL513320.1	0.598740116	9.82E-54	postive
TAZ	AL513320.1	0.7425274	1.60E-95	postive
RGS4	H19	0.640915418	1.12E-63	postive
KLHL24	AC004837.2	0.593047978	1.67E-52	postive
IREB2	AC004837.2	0.423988788	6.28E-25	postive

GABPB1	AC004837.2	0.478661496	3.24E-32	postive
PIK3CA	AC004837.2	0.480737213	1.61E-32	postive
KRAS	AC004837.2	0.432456323	5.68E-26	postive
LINC00472	AC004837.2	0.918341033	2.58E-218	postive
LPIN1	AC004837.2	0.4580155	2.62E-29	postive
ATM	AC004837.2	0.451436813	2.01E-28	postive
ISCU	AC013472.1	0.440804602	4.96E-27	postive
SLC2A8	AC013472.1	0.508222981	9.69E-37	postive
ATG4D	AC013472.1	0.516039334	5.18E-38	postive
GABARAPL1	AC013472.1	0.445465629	1.23E-27	postive
LPIN1	AC013472.1	0.488658841	1.07E-33	postive
TFAP2C	AL137002.1	0.480374752	1.82E-32	postive
HELLS	AC044781.1	0.524423545	2.05E-39	postive
GABPB1	AC044781.1	0.513308426	1.45E-37	postive
ATG7	AC044781.1	0.464998201	2.86E-30	postive
LINC00472	AC044781.1	0.597278002	2.05E-53	postive
ATM	AC044781.1	0.693803565	1.37E-78	postive
FBXW7	AC044781.1	0.442297106	3.19E-27	postive
TFAP2C	AC114939.1	0.573463055	1.89E-48	postive
HBA1	AC114939.1	0.522225703	4.83E-39	postive
DUOX1	AC114939.1	0.423890288	6.46E-25	postive
HELLS	AP005899.1	0.568834652	1.56E-47	postive
ZNF419	AP005899.1	0.499074287	2.72E-35	postive
TUBE1	AP005899.1	0.548190119	1.32E-43	postive
ALOX12	AP005899.1	0.670110579	1.58E-71	postive
GABPB1	AP005899.1	0.567847442	2.45E-47	postive
MAPK8	AP005899.1	0.445792601	1.12E-27	postive
LINC00472	AP005899.1	0.617592796	5.42E-58	postive
ATM	AP005899.1	0.689353223	3.27E-77	postive
FBXW7	AP005899.1	0.534510802	3.75E-41	postive
HELLS	AC007292.2	0.492437556	2.86E-34	postive
ZNF419	AC007292.2	0.420416364	1.70E-24	postive
TUBE1	AC007292.2	0.552017824	2.58E-44	postive
ALOX12	AC007292.2	0.611707668	1.24E-56	postive
GABPB1	AC007292.2	0.466941349	1.53E-30	postive
LINC00472	AC007292.2	0.436708055	1.65E-26	postive
ATM	AC007292.2	0.668637294	4.13E-71	postive
FBXW7	AC007292.2	0.472619888	2.41E-31	postive
ZNF419	OCIAD1-AS1	0.471168901	3.88E-31	postive
KLHL24	OCIAD1-AS1	0.446438758	9.21E-28	postive
TUBE1	OCIAD1-AS1	0.499126632	2.66E-35	postive
GABPB1	OCIAD1-AS1	0.494710784	1.29E-34	postive
LINC00472	OCIAD1-AS1	0.654896202	2.51E-67	postive
LPIN1	OCIAD1-AS1	0.411481515	1.94E-23	postive
ATM	OCIAD1-AS1	0.401434552	2.74E-22	postive
FBXW7	OCIAD1-AS1	0.420451145	1.68E-24	postive
HELLS	AC034229.4	0.566530875	4.43E-47	postive
ZNF419	AC034229.4	0.404703838	1.17E-22	postive
TUBE1	AC034229.4	0.529821994	2.45E-40	postive
ALOX12	AC034229.4	0.430541486	9.83E-26	postive
GABPB1	AC034229.4	0.529072371	3.30E-40	postive
LINC00472	AC034229.4	0.578589908	1.74E-49	postive
ATM	AC034229.4	0.62437512	1.35E-59	postive
FBXW7	AC034229.4	0.458867824	2.01E-29	postive
TF	LINC01914	0.440033937	6.23E-27	postive
ALOX12	LINC00910	0.482407437	9.15E-33	postive
DUOX1	LINC00910	0.400051345	3.92E-22	postive
HELLS	Z68871.1	0.456176663	4.66E-29	postive

MTOR	Z68871.1	0.566089536	5.40E-47	postive
ACSL3	Z68871.1	0.408119386	4.75E-23	postive
ZNF419	Z68871.1	0.41850939	2.87E-24	postive
KLHL24	Z68871.1	0.632032446	1.87E-61	postive
TUBE1	Z68871.1	0.430161113	1.10E-25	postive
MAP3K5	Z68871.1	0.50826443	9.54E-37	postive
EIF2AK4	Z68871.1	0.446852865	8.13E-28	postive
IREB2	Z68871.1	0.628188862	1.63E-60	postive
GABPB1	Z68871.1	0.577182206	3.37E-49	postive
PIK3CA	Z68871.1	0.630495628	4.46E-61	postive
KRAS	Z68871.1	0.405045095	1.07E-22	postive
ATG7	Z68871.1	0.551080553	3.86E-44	postive
MAPK8	Z68871.1	0.533104121	6.60E-41	postive
LINC00472	Z68871.1	0.687526397	1.18E-76	postive
PRKAA2	Z68871.1	0.499744345	2.13E-35	postive
PRKAA1	Z68871.1	0.54648603	2.70E-43	postive
TGFBR1	Z68871.1	0.463415795	4.75E-30	postive
LPIN1	Z68871.1	0.438458975	9.91E-27	postive
TLR4	Z68871.1	0.464817444	3.04E-30	postive
ATM	Z68871.1	0.800048872	2.99E-121	postive
FBXW7	Z68871.1	0.606893372	1.53E-55	postive
BACH1	Z68871.1	0.452089949	1.65E-28	postive
VEGFA	AC044849.1	0.459344734	1.73E-29	postive
ALOX12	AC044849.1	0.45077132	2.47E-28	postive
TAZ	AC044849.1	0.576425891	4.79E-49	postive
PHKG2	AC068722.2	0.479893458	2.14E-32	postive
TAZ	AC068722.2	0.552398545	2.19E-44	postive
HELLS	AL021707.8	0.404980282	1.09E-22	postive
BRD4	AL021707.8	0.433867313	3.78E-26	postive
ZNF419	AL021707.8	0.50834442	9.26E-37	postive
TUBE1	AL021707.8	0.510184384	4.68E-37	postive
ALOX12	AL021707.8	0.553286497	1.50E-44	postive
GABPB1	AL021707.8	0.419013747	2.50E-24	postive
PHKG2	AL021707.8	0.437589197	1.28E-26	postive
ATM	AL021707.8	0.47439075	1.34E-31	postive
TAZ	AL021707.8	0.554929169	7.38E-45	postive
FBXW7	AL021707.8	0.494599613	1.34E-34	postive
HELLS	AC073389.3	0.422905182	8.50E-25	postive
ZNF419	AC073389.3	0.427792699	2.15E-25	postive
TUBE1	AC073389.3	0.481959862	1.06E-32	postive
DRD4	AC073389.3	0.402928755	1.86E-22	postive
ALOX12	AC073389.3	0.558907544	1.31E-45	postive
PHKG2	AC073389.3	0.545762201	3.66E-43	postive
TAZ	AC073389.3	0.569335776	1.25E-47	postive
HELLS	AL662844.3	0.482445052	9.03E-33	postive
ZNF419	AL662844.3	0.482425959	9.09E-33	postive
TUBE1	AL662844.3	0.640101716	1.80E-63	postive
ALOX12	AL662844.3	0.738265667	6.83E-94	postive
GABPB1	AL662844.3	0.478361574	3.58E-32	postive
ATM	AL662844.3	0.629402831	8.25E-61	postive
FBXW7	AL662844.3	0.435481988	2.36E-26	postive
ZNF419	LINC01011	0.404861889	1.12E-22	postive
VEGFA	LINC01011	0.520619403	8.98E-39	postive
TUBE1	LINC01011	0.481084557	1.43E-32	postive
ALOX12	LINC01011	0.56247626	2.71E-46	postive
TAZ	LINC01011	0.500718571	1.50E-35	postive
FANCD2	AL022316.1	0.435475425	2.37E-26	postive
ALOX12	AL022316.1	0.419628388	2.11E-24	postive

TFAP2C	AL022316.1	0.409292108	3.48E-23	postive
HELLS	AC074138.1	0.530753932	1.69E-40	postive
ZNF419	AC074138.1	0.553358255	1.45E-44	postive
KLHL24	AC074138.1	0.448957543	4.30E-28	postive
TUBE1	AC074138.1	0.56325558	1.92E-46	postive
ALOX12	AC074138.1	0.601022763	3.10E-54	postive
IREB2	AC074138.1	0.419321	2.30E-24	postive
GABPB1	AC074138.1	0.606688281	1.71E-55	postive
MAPK8	AC074138.1	0.449395475	3.76E-28	postive
LINC00472	AC074138.1	0.761934486	2.22E-103	postive
ATM	AC074138.1	0.712419119	1.22E-84	postive
FBXW7	AC074138.1	0.524866576	1.73E-39	postive
PEBP1	AC022034.1	-0.444295751	1.75E-27	negative
HELLS	AC004466.2	0.543120871	1.10E-42	postive
ZNF419	AC004466.2	0.438796213	8.97E-27	postive
TUBE1	AC004466.2	0.482581592	8.62E-33	postive
ALOX12	AC004466.2	0.523510961	2.93E-39	postive
GABPB1	AC004466.2	0.541764736	1.93E-42	postive
LINC00472	AC004466.2	0.514249049	1.02E-37	postive
ATM	AC004466.2	0.698044412	6.28E-80	postive
FBXW7	AC004466.2	0.523799436	2.62E-39	postive
LINC00472	AL512306.3	0.430032246	1.14E-25	postive
ATM	AL512306.3	0.465771917	2.23E-30	postive
HELLS	AC087286.2	0.512944863	1.67E-37	postive
IREB2	AC087286.2	0.463222882	5.05E-30	postive
GABPB1	AC087286.2	0.595704701	4.48E-53	postive
PIK3CA	AC087286.2	0.474829185	1.16E-31	postive
ATG7	AC087286.2	0.486726989	2.09E-33	postive
ZEB1	AC087286.2	0.430461202	1.01E-25	postive
MAPK8	AC087286.2	0.477225168	5.24E-32	postive
LINC00472	AC087286.2	0.666941417	1.24E-70	postive
PRKAA2	AC087286.2	0.403571917	1.57E-22	postive
PRKAA1	AC087286.2	0.403051265	1.80E-22	postive
ATM	AC087286.2	0.765684804	5.49E-105	postive
FBXW7	AC087286.2	0.531175134	1.43E-40	postive
BACH1	AC087286.2	0.436600799	1.71E-26	postive
GPX4	GIHCG	0.505796052	2.37E-36	postive
HSPB1	GIHCG	0.481247903	1.36E-32	postive
HSF1	GIHCG	0.409890135	2.96E-23	postive
STAT3	GIHCG	-0.40590303	8.53E-23	negative
OTUB1	GIHCG	0.454737952	7.28E-29	postive
RPL8	GIHCG	0.436064612	2.00E-26	postive
PHKG2	GIHCG	0.500093459	1.88E-35	postive
BECN1	GIHCG	-0.410325788	2.64E-23	negative
ZEB1	GIHCG	-0.438102376	1.10E-26	negative
EPAS1	GIHCG	-0.477439876	4.88E-32	negative
ZNF419	AL035252.3	0.430598755	9.67E-26	postive
PHKG2	AL035252.3	0.436008261	2.03E-26	postive
TAZ	AL035252.3	0.467589744	1.24E-30	postive
ISCU	CA3-AS1	0.625964763	5.61E-60	postive
ATG4D	CA3-AS1	0.50717699	1.43E-36	postive
MAP1LC3A	CA3-AS1	0.424056756	6.16E-25	postive
GABARAPL2	CA3-AS1	0.496165883	7.67E-35	postive
GABARAPL1	CA3-AS1	0.464354512	3.52E-30	postive
TFAP2C	AC098487.1	0.427869989	2.10E-25	postive
HBA1	AC098487.1	0.495640783	9.25E-35	postive
ZNF419	AL355385.1	0.555012178	7.12E-45	postive
ALOX12	AL355385.1	0.406431774	7.42E-23	postive

PHKG2	AL355385.1	0.535230769	2.80E-41	postive
EGLN2	AL355385.1	0.422391047	9.81E-25	postive
TAZ	AL355385.1	0.574731223	1.05E-48	postive
ATG4D	AC139768.1	0.454233397	8.51E-29	postive
MAP1LC3A	AC139768.1	0.416746658	4.65E-24	postive
ALOX12	FBXL19-AS1	0.424733045	5.10E-25	postive
ZNF419	AC073957.3	0.487857192	1.41E-33	postive
VEGFA	AC073957.3	0.415490407	6.55E-24	postive
TUBE1	AC073957.3	0.543709429	8.62E-43	postive
SETD1B	AC073957.3	0.538172244	8.46E-42	postive
ALOX12	AC073957.3	0.680069777	2.05E-74	postive
ATM	AC073957.3	0.511983004	2.39E-37	postive
TAZ	AC073957.3	0.404765741	1.15E-22	postive
FH	LINC01532	0.418575151	2.82E-24	postive
ISCU	LINC01532	0.452954039	1.26E-28	postive
ATP5MC3	LINC01532	0.406391362	7.50E-23	postive
SLC2A8	LINC01532	0.408571819	4.21E-23	postive
GOT1	LINC01532	0.452279458	1.56E-28	postive
ATG4D	LINC01532	0.49936988	2.44E-35	postive
GABARAPL2	LINC01532	0.614227918	3.28E-57	postive
SRXN1	LINC00958	0.48986282	7.05E-34	postive
PRKAA2	AC092354.2	0.402205969	2.25E-22	postive
PRKAA1	AC092354.2	0.428251737	1.89E-25	postive
GPX4	AC133552.5	0.553376065	1.44E-44	postive
HSPB1	AC133552.5	0.462430778	6.50E-30	postive
STAT3	AC133552.5	-0.415707396	6.18E-24	negative
OTUB1	AC133552.5	0.495472964	9.81E-35	postive
RPL8	AC133552.5	0.466443885	1.80E-30	postive
SLC2A8	AC133552.5	0.406300302	7.68E-23	postive
HBA1	AC133552.5	0.401360121	2.80E-22	postive
NRAS	AC133552.5	-0.421837016	1.14E-24	negative
HRAS	AC133552.5	0.610979957	1.82E-56	postive
MAP1LC3A	AC133552.5	0.601508953	2.42E-54	postive
ANO6	AC133552.5	-0.432909013	4.98E-26	negative
EGLN2	AC133552.5	0.578172391	2.12E-49	postive
HELLS	AC007684.2	0.504949498	3.23E-36	postive
KLHL24	AC007684.2	0.471762852	3.19E-31	postive
GABPB1	AC007684.2	0.539294124	5.35E-42	postive
PIK3CA	AC007684.2	0.405197991	1.03E-22	postive
LINC00472	AC007684.2	0.817217227	1.27E-130	postive
ATM	AC007684.2	0.568203711	2.08E-47	postive
FBXW7	AC007684.2	0.439326978	7.67E-27	postive
MUC1	AC004870.2	0.403812464	1.48E-22	postive
SLC7A5	AC004870.2	0.520052776	1.12E-38	postive
HELLS	AL031705.1	0.444136018	1.84E-27	postive
BRD4	AL031705.1	0.406570353	7.16E-23	postive
ZNF419	AL031705.1	0.499171638	2.62E-35	postive
VEGFA	AL031705.1	0.42827714	1.87E-25	postive
TUBE1	AL031705.1	0.584689598	9.69E-51	postive
SETD1B	AL031705.1	0.47726352	5.17E-32	postive
DRD4	AL031705.1	0.409003917	3.75E-23	postive
ALOX12	AL031705.1	0.710180938	6.88E-84	postive
PHKG2	AL031705.1	0.444222307	1.79E-27	postive
ATM	AL031705.1	0.446425095	9.25E-28	postive
TAZ	AL031705.1	0.549055982	9.12E-44	postive
TUBE1	AL356019.2	0.424669439	5.19E-25	postive
ALOX12	AL356019.2	0.554125629	1.04E-44	postive
SP1	AL356019.2	0.469832433	6.00E-31	postive

MAPK8	AL356019.2	0.425130651	4.56E-25	postive
ATM	AL356019.2	0.445992069	1.05E-27	postive
BRD4	ARHGAP27P1	0.421619102	1.22E-24	postive
ZNF419	ARHGAP27P1	0.593604572	1.27E-52	postive
VEGFA	ARHGAP27P1	0.509682535	5.64E-37	postive
TUBE1	ARHGAP27P1	0.613617854	4.53E-57	postive
SETD1B	ARHGAP27P1	0.475893967	8.16E-32	postive
DRD4	ARHGAP27P1	0.411664176	1.84E-23	postive
ALOX12	ARHGAP27P1	0.659359241	1.56E-68	postive
GABPB1	ARHGAP27P1	0.421880846	1.13E-24	postive
PHKG2	ARHGAP27P1	0.469491429	6.71E-31	postive
TAZ	ARHGAP27P1	0.690398569	1.56E-77	postive
FBXW7	ARHGAP27P1	0.493031461	2.32E-34	postive
LINC00472	AP002518.2	0.550326244	5.32E-44	postive
ATM	AP002518.2	0.51482259	8.21E-38	postive
HELLS	AL031775.2	0.431370229	7.76E-26	postive
ZNF419	AL031775.2	0.515111984	7.36E-38	postive
VEGFA	AL031775.2	0.4001427	3.83E-22	postive
TUBE1	AL031775.2	0.685405894	5.20E-76	postive
ALOX12	AL031775.2	0.624061036	1.61E-59	postive
GABPB1	AL031775.2	0.541317636	2.32E-42	postive
LINC00472	AL031775.2	0.550304498	5.37E-44	postive
ATM	AL031775.2	0.60390675	7.14E-55	postive
TAZ	AL031775.2	0.414037504	9.72E-24	postive
FBXW7	AL031775.2	0.505553035	2.59E-36	postive
TUBE1	TMEM254-A	0.403422516	1.64E-22	postive
ALOX12	TMEM254-A	0.40800908	4.89E-23	postive
TUBE1	AC092140.2	0.457292944	3.29E-29	postive
GABPB1	AC092140.2	0.406289291	7.71E-23	postive
LINC00472	AC092140.2	0.462749066	5.88E-30	postive
ATM	AC092140.2	0.430465588	1.00E-25	postive
ZNF419	AC024361.1	0.435942142	2.07E-26	postive
ALOX12	AC024361.1	0.479990719	2.07E-32	postive
LINC00472	AC024361.1	0.514648427	8.77E-38	postive
ZNF419	LINC00667	0.446884331	8.05E-28	postive
VEGFA	LINC00667	0.409671178	3.14E-23	postive
TUBE1	LINC00667	0.5215829	6.19E-39	postive
SETD1B	LINC00667	0.416412163	5.10E-24	postive
ALOX12	LINC00667	0.488639937	1.08E-33	postive
GABPB1	LINC00667	0.5283322	4.42E-40	postive
MAPK8	LINC00667	0.521156455	7.30E-39	postive
ATM	LINC00667	0.543275072	1.03E-42	postive
YY1AP1	LINC00667	0.453321215	1.13E-28	postive
FANCD2	AL008582.1	0.413635701	1.08E-23	postive
HELLS	AL008582.1	0.444798281	1.51E-27	postive
ZNF419	AL008582.1	0.459225652	1.79E-29	postive
TUBE1	AL008582.1	0.42822723	1.90E-25	postive
ALOX12	AL008582.1	0.570878014	6.18E-48	postive
PHKG2	AL008582.1	0.481344459	1.31E-32	postive
TAZ	AL008582.1	0.551473266	3.26E-44	postive
FANCD2	THUMPD3-A	0.507023882	1.51E-36	postive
HELLS	THUMPD3-A	0.539690249	4.54E-42	postive
ZNF419	THUMPD3-A	0.685119052	6.35E-76	postive
TUBE1	THUMPD3-A	0.608748719	5.85E-56	postive
ALOX12	THUMPD3-A	0.528304603	4.47E-40	postive
GABPB1	THUMPD3-A	0.616062068	1.23E-57	postive
LINC00472	THUMPD3-A	0.596754826	2.66E-53	postive
LPIN1	THUMPD3-A	0.503174049	6.17E-36	postive

ATM	THUMPD3-A	0.526968936	7.57E-40	postive
TAZ	THUMPD3-A	0.452719394	1.36E-28	postive
FBXW7	THUMPD3-A	0.515268239	6.94E-38	postive
HELLS	AC011939.2	0.477175429	5.33E-32	postive
KLHL24	AC011939.2	0.471368843	3.63E-31	postive
IREB2	AC011939.2	0.425012042	4.71E-25	postive
GABPB1	AC011939.2	0.548388668	1.21E-43	postive
PIK3CA	AC011939.2	0.44420008	1.81E-27	postive
ATG7	AC011939.2	0.436656513	1.68E-26	postive
MAPK8	AC011939.2	0.408727832	4.04E-23	postive
LINC00472	AC011939.2	0.861581261	2.99E-160	postive
ATM	AC011939.2	0.671623797	5.85E-72	postive
FBXW7	AC011939.2	0.433078467	4.74E-26	postive
TFAP2C	C6orf99	0.538001219	9.08E-42	postive
HBA1	C6orf99	0.50951642	6.00E-37	postive
BRD4	SNHG20	0.40541149	9.71E-23	postive
ZNF419	SNHG20	0.546252248	2.98E-43	postive
VEGFA	SNHG20	0.582489888	2.77E-50	postive
TUBE1	SNHG20	0.579916143	9.35E-50	postive
SETD1B	SNHG20	0.437370746	1.36E-26	postive
DRD4	SNHG20	0.426920478	2.75E-25	postive
ALOX12	SNHG20	0.707209984	6.70E-83	postive
GABPB1	SNHG20	0.404800933	1.14E-22	postive
ATM	SNHG20	0.42411532	6.06E-25	postive
YY1AP1	SNHG20	0.436316233	1.85E-26	postive
TAZ	SNHG20	0.613274099	5.43E-57	postive
FBXW7	SNHG20	0.430134238	1.10E-25	postive
ZNF419	AFDN-DT	0.402751072	1.95E-22	postive
VEGFA	AFDN-DT	0.48900648	9.49E-34	postive
TUBE1	AFDN-DT	0.439030218	8.37E-27	postive
ALOX12	AFDN-DT	0.546301895	2.92E-43	postive
HELLS	HLA-F-AS1	0.461338519	9.20E-30	postive
ZNF419	HLA-F-AS1	0.493702225	1.84E-34	postive
TUBE1	HLA-F-AS1	0.580546614	6.95E-50	postive
ALOX12	HLA-F-AS1	0.61609461	1.21E-57	postive
GABPB1	HLA-F-AS1	0.452959945	1.26E-28	postive
ATM	HLA-F-AS1	0.553261875	1.52E-44	postive
FBXW7	HLA-F-AS1	0.423532894	7.13E-25	postive
HELLS	AL590133.1	0.441612233	3.90E-27	postive
TUBE1	AL590133.1	0.449818244	3.31E-28	postive
ALOX12	AL590133.1	0.66344313	1.17E-69	postive
ATM	AL590133.1	0.573334392	2.00E-48	postive
PHKG2	AC025171.5	0.444629597	1.59E-27	postive
TAZ	AC025171.5	0.516888997	3.75E-38	postive
ZNF419	AL136304.1	0.502513068	7.85E-36	postive
VEGFA	AL136304.1	0.403063084	1.80E-22	postive
TUBE1	AL136304.1	0.533284523	6.14E-41	postive
ALOX12	AL136304.1	0.55104224	3.92E-44	postive
PHKG2	AL136304.1	0.493276098	2.13E-34	postive
TAZ	AL136304.1	0.624242025	1.45E-59	postive
HELLS	AC002128.2	0.522910987	3.70E-39	postive
ALOX12	AC002128.2	0.498012803	3.97E-35	postive
TFAP2C	AC002128.2	0.435994275	2.04E-26	postive
GABPB1	AC002128.2	0.41370843	1.06E-23	postive
DUOX1	AC002128.2	0.450627156	2.58E-28	postive
LINC00472	AC002128.2	0.418234017	3.10E-24	postive
ATM	AC002128.2	0.482197673	9.82E-33	postive
FBXW7	AC002128.2	0.436857627	1.58E-26	postive

HELLS	AC232271.1	0.408000456	4.90E-23	postive
BRD4	AC232271.1	0.436538376	1.74E-26	postive
ZNF419	AC232271.1	0.616281667	1.10E-57	postive
VEGFA	AC232271.1	0.407181643	6.09E-23	postive
TUBE1	AC232271.1	0.571258257	5.19E-48	postive
SETD1B	AC232271.1	0.481650287	1.18E-32	postive
DRD4	AC232271.1	0.412561635	1.45E-23	postive
ALOX12	AC232271.1	0.688877608	4.57E-77	postive
PHKG2	AC232271.1	0.527572329	5.97E-40	postive
TAZ	AC232271.1	0.652288695	1.24E-66	postive
ZNF419	AC062037.2	0.463134972	5.20E-30	postive
TUBE1	AC062037.2	0.492324466	2.98E-34	postive
SETD1B	AC062037.2	0.410376862	2.60E-23	postive
ALOX12	AC062037.2	0.444224663	1.79E-27	postive
LINC00472	AC062037.2	0.412645053	1.42E-23	postive
ATM	AC062037.2	0.426831444	2.82E-25	postive
FBXW7	AC062037.2	0.406187843	7.92E-23	postive
TFAP2C	LNCTAM34A	0.43692523	1.55E-26	postive
ALOX12B	LNCTAM34A	0.483081702	7.27E-33	postive
PEBP1	AL138826.1	0.446016429	1.05E-27	postive
HELLS	LINC00894	0.513659306	1.27E-37	postive
ZNF419	LINC00894	0.536648883	1.57E-41	postive
TUBE1	LINC00894	0.607490957	1.13E-55	postive
ALOX12	LINC00894	0.588086597	1.89E-51	postive
GABPB1	LINC00894	0.548476928	1.17E-43	postive
LINC00472	LINC00894	0.668825034	3.66E-71	postive
ATM	LINC00894	0.600629598	3.79E-54	postive
TAZ	LINC00894	0.420424508	1.69E-24	postive
FBXW7	LINC00894	0.480404331	1.80E-32	postive
HELLS	AC021739.2	0.413816298	1.03E-23	postive
ZNF419	AC021739.2	0.493106012	2.26E-34	postive
TUBE1	AC021739.2	0.435994146	2.04E-26	postive
ALOX12	AC021739.2	0.495582516	9.44E-35	postive
GABPB1	AC021739.2	0.533911214	4.77E-41	postive
LINC00472	AC021739.2	0.424900318	4.86E-25	postive
ATM	AC021739.2	0.585741623	5.85E-51	postive
FBXW7	AC021739.2	0.52566992	1.26E-39	postive
HELLS	AC011726.2	0.466729545	1.64E-30	postive
TUBE1	AC011726.2	0.512876761	1.71E-37	postive
ALOX12	AC011726.2	0.581856919	3.74E-50	postive
GABPB1	AC011726.2	0.440653224	5.19E-27	postive
LINC00472	AC011726.2	0.429978892	1.15E-25	postive
ATM	AC011726.2	0.738327066	6.48E-94	postive
FBXW7	AC011726.2	0.431734281	6.99E-26	postive
MTOR	PPIC-AS1	0.429308312	1.40E-25	postive
ACSL3	PPIC-AS1	0.440618613	5.24E-27	postive
LPIN1	PPIC-AS1	0.440797127	4.97E-27	postive
ZNF419	AC006064.3	0.473001296	2.13E-31	postive
VEGFA	AC006064.3	0.478872262	3.02E-32	postive
TUBE1	AC006064.3	0.459618583	1.58E-29	postive
SETD1B	AC006064.3	0.447692436	6.31E-28	postive
ALOX12	AC006064.3	0.685966379	3.52E-76	postive
TAZ	AC006064.3	0.544164139	7.14E-43	postive
SETD1B	AP001062.1	0.410141511	2.77E-23	postive
ALOX12	AP001062.1	0.444273434	1.77E-27	postive
STAT3	AC135507.1	-0.408928827	3.83E-23	negative
ISCU	AC135507.1	0.591254653	4.03E-52	postive
TMBIM4	AC135507.1	0.438432184	9.98E-27	postive

DDIT3	AC135507.1	0.463663844	4.39E-30	postive
ATG4D	AC135507.1	0.494093747	1.60E-34	postive
MAP1LC3A	AC135507.1	0.470101023	5.50E-31	postive
GABARAPL2	AC135507.1	0.472889371	2.21E-31	postive
GABARAPL1	AC135507.1	0.447745426	6.21E-28	postive
LPIN1	AC135507.1	0.423243847	7.73E-25	postive
TFAP2C	AC108134.2	0.534949857	3.14E-41	postive
HBA1	AC108134.2	0.416750166	4.65E-24	postive
DUOX1	AC108134.2	0.412178001	1.61E-23	postive
ZNF419	AC254562.3	0.415967275	5.76E-24	postive
VEGFA	AC254562.3	0.50825563	9.57E-37	postive
TUBE1	AC254562.3	0.462380041	6.61E-30	postive
SETD1B	AC254562.3	0.410602759	2.45E-23	postive
DRD4	AC254562.3	0.420724503	1.56E-24	postive
ALOX12	AC254562.3	0.562844144	2.30E-46	postive
TAZ	AC254562.3	0.641179007	9.61E-64	postive
HELLS	AC036108.3	0.421199674	1.37E-24	postive
ZNF419	AC036108.3	0.499385702	2.43E-35	postive
KLHL24	AC036108.3	0.403015863	1.82E-22	postive
TUBE1	AC036108.3	0.600989853	3.15E-54	postive
ALOX12	AC036108.3	0.500317562	1.74E-35	postive
IREB2	AC036108.3	0.408060794	4.82E-23	postive
GABPB1	AC036108.3	0.612660723	7.52E-57	postive
MAPK8	AC036108.3	0.484593846	4.34E-33	postive
LINC00472	AC036108.3	0.673610414	1.57E-72	postive
ATM	AC036108.3	0.670935382	9.20E-72	postive
FBXW7	AC036108.3	0.526693288	8.44E-40	postive
ZNF419	AL121772.3	0.423751753	6.71E-25	postive
LINC00472	AL121772.3	0.533555353	5.50E-41	postive
HRAS	AC064836.2	0.558816232	1.36E-45	postive
EGLN2	AC064836.2	0.472289115	2.69E-31	postive
CYBB	AC243960.3	0.440372455	5.64E-27	postive
ZNF419	PTOV1-AS1	0.494793886	1.25E-34	postive
VEGFA	PTOV1-AS1	0.44150651	4.03E-27	postive
TUBE1	PTOV1-AS1	0.465911651	2.14E-30	postive
SETD1B	PTOV1-AS1	0.483977982	5.36E-33	postive
ALOX12	PTOV1-AS1	0.507748937	1.15E-36	postive
PHKG2	PTOV1-AS1	0.535734833	2.28E-41	postive
ULK1	PTOV1-AS1	0.419007907	2.50E-24	postive
TAZ	PTOV1-AS1	0.653713879	5.19E-67	postive
HELLS	AC066613.1	0.544275238	6.81E-43	postive
ZNF419	AC066613.1	0.503431799	5.62E-36	postive
TUBE1	AC066613.1	0.630858824	3.64E-61	postive
SETD1B	AC066613.1	0.41406649	9.64E-24	postive
ALOX12	AC066613.1	0.687268399	1.42E-76	postive
GABPB1	AC066613.1	0.548153472	1.34E-43	postive
MAPK8	AC066613.1	0.406508712	7.27E-23	postive
LINC00472	AC066613.1	0.500957319	1.38E-35	postive
ATM	AC066613.1	0.74897134	4.73E-98	postive
FBXW7	AC066613.1	0.542561665	1.39E-42	postive
ZNF419	AL451050.2	0.484104704	5.13E-33	postive
ALOX12	AL451050.2	0.453690366	1.01E-28	postive
NCOA4	AL451050.2	-0.413091296	1.26E-23	negative
PHKG2	AL451050.2	0.629282463	8.83E-61	postive
ULK1	AL451050.2	0.440524424	5.39E-27	postive
TAZ	AL451050.2	0.717291215	2.63E-86	postive
TUBE1	AC010175.1	0.437206055	1.43E-26	postive
ALOX12	AC010175.1	0.417611674	3.67E-24	postive

FBXW7	AC010175.1	0.420028269	1.89E-24	postive
HELLS	AC004884.2	0.440116015	6.08E-27	postive
KLHL24	AC004884.2	0.495077765	1.13E-34	postive
IREB2	AC004884.2	0.423419432	7.36E-25	postive
GABPB1	AC004884.2	0.526091991	1.07E-39	postive
PIK3CA	AC004884.2	0.453131577	1.20E-28	postive
ATG7	AC004884.2	0.405694215	9.01E-23	postive
LINC00472	AC004884.2	0.889919836	3.24E-185	postive
ATM	AC004884.2	0.62188922	5.29E-59	postive
ZNF419	AC084018.2	0.520613172	9.00E-39	postive
TUBE1	AC084018.2	0.465748078	2.25E-30	postive
SETD1B	AC084018.2	0.57659655	4.42E-49	postive
ALOX12	AC084018.2	0.632138492	1.76E-61	postive
ATM	AC084018.2	0.406872887	6.61E-23	postive
TAZ	AC084018.2	0.412005209	1.68E-23	postive
HELLS	AC006017.1	0.444203706	1.80E-27	postive
TUBE1	AC006017.1	0.61658999	9.29E-58	postive
ALOX12	AC006017.1	0.541210698	2.43E-42	postive
GABPB1	AC006017.1	0.408432583	4.37E-23	postive
LINC00472	AC006017.1	0.407351621	5.82E-23	postive
ATM	AC006017.1	0.533720842	5.15E-41	postive
FBXW7	AC006017.1	0.411231766	2.07E-23	postive
TFAP2C	APP-DT	0.438328727	1.03E-26	postive
HBA1	APP-DT	0.404316375	1.29E-22	postive
TUBE1	HNF1A-AS1	0.490171301	6.33E-34	postive
ALOX12	HNF1A-AS1	0.615152298	2.00E-57	postive
HELLS	GSN-AS1	0.557907696	2.02E-45	postive
ZNF419	GSN-AS1	0.409054425	3.70E-23	postive
TUBE1	GSN-AS1	0.444850933	1.49E-27	postive
ALOX12	GSN-AS1	0.482254253	9.64E-33	postive
IREB2	GSN-AS1	0.407101772	6.22E-23	postive
GABPB1	GSN-AS1	0.577217409	3.31E-49	postive
ATG7	GSN-AS1	0.453869987	9.53E-29	postive
MAPK8	GSN-AS1	0.425660564	3.93E-25	postive
LINC00472	GSN-AS1	0.688948871	4.35E-77	postive
ATM	GSN-AS1	0.698016958	6.41E-80	postive
FBXW7	GSN-AS1	0.509980844	5.05E-37	postive
NCF2	AL161785.1	0.415965	5.76E-24	postive
CYBB	AL161785.1	0.520885643	8.10E-39	postive
TLR4	AL161785.1	0.418631577	2.78E-24	postive
PHKG2	AC116407.2	0.490824174	5.04E-34	postive
TAZ	AC116407.2	0.571209786	5.31E-48	postive
BRD4	HM13-IT1	0.4419647	3.52E-27	postive
ZNF419	HM13-IT1	0.445884429	1.09E-27	postive
TUBE1	HM13-IT1	0.487612325	1.54E-33	postive
SETD1B	HM13-IT1	0.46115796	9.74E-30	postive
ALOX12	HM13-IT1	0.576818682	3.99E-49	postive
PHKG2	HM13-IT1	0.49408179	1.61E-34	postive
TAZ	HM13-IT1	0.565429665	7.26E-47	postive
FBXW7	HM13-IT1	0.444439797	1.68E-27	postive
BRD4	AC012615.6	0.449769195	3.35E-28	postive
ZNF419	AC012615.6	0.549111733	8.91E-44	postive
VEGFA	AC012615.6	0.546332018	2.88E-43	postive
TUBE1	AC012615.6	0.62740213	2.53E-60	postive
SETD1B	AC012615.6	0.583386459	1.81E-50	postive
DRD4	AC012615.6	0.428973309	1.54E-25	postive
ALOX12	AC012615.6	0.757251799	2.04E-101	postive
PHKG2	AC012615.6	0.445241989	1.32E-27	postive

ATM	AC012615.6	0.447931567	5.87E-28	postive
TAZ	AC012615.6	0.627882998	1.93E-60	postive
FBXW7	AC012615.6	0.411419985	1.97E-23	postive
HELLS	NUTM2B-AS	0.573003314	2.33E-48	postive
TUBE1	NUTM2B-AS	0.487796215	1.44E-33	postive
ALOX12	NUTM2B-AS	0.469504127	6.68E-31	postive
IREB2	NUTM2B-AS	0.400587759	3.42E-22	postive
GABPB1	NUTM2B-AS	0.596477407	3.05E-53	postive
MAPK8	NUTM2B-AS	0.48767602	1.50E-33	postive
LINC00472	NUTM2B-AS	0.698640146	4.06E-80	postive
ATM	NUTM2B-AS	0.730052947	7.76E-91	postive
FBXW7	NUTM2B-AS	0.503575139	5.33E-36	postive
ZNF419	AL121987.2	0.434181252	3.45E-26	postive
VEGFA	AL121987.2	0.55980215	8.82E-46	postive
TUBE1	AL121987.2	0.530081596	2.21E-40	postive
SETD1B	AL121987.2	0.512412	2.03E-37	postive
ALOX12	AL121987.2	0.572575567	2.84E-48	postive
ATM	AL121987.2	0.452998992	1.25E-28	postive
TAZ	AL121987.2	0.460875266	1.07E-29	postive
ZNF419	AC009812.1	0.428949223	1.55E-25	postive
TUBE1	AC009812.1	0.42181538	1.15E-24	postive
GABPB1	AC009812.1	0.461371973	9.10E-30	postive
TAZ	AC009812.1	0.431059672	8.48E-26	postive
PHKG2	AL096865.1	0.481791675	1.13E-32	postive
TAZ	AL096865.1	0.595984017	3.90E-53	postive
HELLS	AC060766.6	0.44557302	1.20E-27	postive
TUBE1	AC060766.6	0.414959429	7.57E-24	postive
GABPB1	AC060766.6	0.485033308	3.73E-33	postive
ATM	AC060766.6	0.554748567	7.98E-45	postive
FBXW7	AC060766.6	0.507253839	1.39E-36	postive
JDP2	AC027601.2	0.403517926	1.60E-22	postive
VEGFA	AC027601.2	0.452277441	1.56E-28	postive
MAPK3	AC027601.2	0.450905245	2.37E-28	postive
ZEB1	AC027601.2	0.58770484	2.27E-51	postive
EPAS1	AC027601.2	0.693093131	2.28E-78	postive
GCLC	DPH6-DT	0.412342368	1.54E-23	postive
PRKAA2	DPH6-DT	0.451864215	1.77E-28	postive
ALOX12	AC090844.2	0.528233537	4.60E-40	postive
LPIN1	AC011450.1	0.43489919	2.80E-26	postive
ALOX12	FAM111A-D	0.457154956	3.43E-29	postive
IREB2	FAM111A-D	0.431913181	6.64E-26	postive
SP1	FAM111A-D	0.445377502	1.27E-27	postive
ATM	FAM111A-D	0.489435897	8.17E-34	postive
TUBE1	AC003956.1	0.412400781	1.51E-23	postive
SETD1B	AC003956.1	0.428231547	1.90E-25	postive
ALOX12	AC003956.1	0.494834377	1.23E-34	postive
VEGFA	LINC00989	0.410076064	2.82E-23	postive
GABPB1	LINC00989	0.47094019	4.18E-31	postive
ZEB1	LINC00989	0.661915878	3.10E-69	postive
MAPK8	LINC00989	0.44461131	1.60E-27	postive
EPAS1	LINC00989	0.543601826	9.02E-43	postive
FBXW7	LINC00989	0.405568401	9.32E-23	postive
HELLS	AC007390.1	0.602644895	1.36E-54	postive
ZNF419	AC007390.1	0.490526371	5.59E-34	postive
TUBE1	AC007390.1	0.585910666	5.39E-51	postive
ALOX12	AC007390.1	0.63379436	6.88E-62	postive
GABPB1	AC007390.1	0.56577429	6.22E-47	postive
MAPK8	AC007390.1	0.415142216	7.20E-24	postive

LINC00472	AC007390.1	0.54052135	3.23E-42	postive
ATM	AC007390.1	0.646074808	5.31E-65	postive
FBXW7	AC007390.1	0.527466179	6.22E-40	postive
LINC00472	UBE2D3-AS1	0.580461046	7.23E-50	postive
BRD4	AC108134.3	0.408628446	4.15E-23	postive
ZNF419	AC108134.3	0.476714018	6.21E-32	postive
VEGFA	AC108134.3	0.4680578	1.07E-30	postive
TUBE1	AC108134.3	0.451053818	2.27E-28	postive
SETD1B	AC108134.3	0.407164331	6.12E-23	postive
ALOX12	AC108134.3	0.519558636	1.35E-38	postive
TAZ	AC108134.3	0.620933181	8.90E-59	postive
ISCU	AC092142.1	0.410609805	2.45E-23	postive
TMBIM4	AC092142.1	0.424036953	6.20E-25	postive
BRD4	LINC00115	0.454531817	7.76E-29	postive
ZNF419	LINC00115	0.5038523	4.82E-36	postive
VEGFA	LINC00115	0.456652901	4.01E-29	postive
TUBE1	LINC00115	0.508066431	1.03E-36	postive
DRD4	LINC00115	0.406229665	7.83E-23	postive
ALOX12	LINC00115	0.635599492	2.45E-62	postive
NCOA4	LINC00115	-0.407823637	5.14E-23	negative
PHKG2	LINC00115	0.512840724	1.73E-37	postive
TAZ	LINC00115	0.702577129	2.20E-81	postive
BRD4	AC107375.1	0.468840708	8.29E-31	postive
ZNF419	AC107375.1	0.578579706	1.75E-49	postive
VEGFA	AC107375.1	0.451941612	1.73E-28	postive
TUBE1	AC107375.1	0.523736712	2.68E-39	postive
SETD1B	AC107375.1	0.481704823	1.16E-32	postive
DRD4	AC107375.1	0.422671465	9.07E-25	postive
ALOX12	AC107375.1	0.669593349	2.22E-71	postive
GABPB1	AC107375.1	0.444604435	1.60E-27	postive
TAZ	AC107375.1	0.52696003	7.60E-40	postive
FH	INSYN1-AS1	0.493331554	2.09E-34	postive
ISCU	INSYN1-AS1	0.553081339	1.64E-44	postive
ATP5MC3	INSYN1-AS1	0.40544388	9.63E-23	postive
GOT1	INSYN1-AS1	0.471800345	3.15E-31	postive
ATG4D	INSYN1-AS1	0.497781595	4.31E-35	postive
GABARAPL2	INSYN1-AS1	0.561138437	4.90E-46	postive
GABARAPL1	INSYN1-AS1	0.512105849	2.28E-37	postive
TUBE1	AC008537.2	0.490908674	4.89E-34	postive
SETD1B	AC008537.2	0.461457431	8.86E-30	postive
ALOX12	AC008537.2	0.621748146	5.71E-59	postive
IREB2	AC008537.2	0.45899813	1.93E-29	postive
GABPB1	AC008537.2	0.49283679	2.49E-34	postive
MAPK8	AC008537.2	0.482085502	1.02E-32	postive
LINC00472	AC008537.2	0.478210029	3.77E-32	postive
ATM	AC008537.2	0.615175084	1.98E-57	postive
FBXW7	AC008537.2	0.424190352	5.94E-25	postive
ALOX12	HRAT92	0.409333085	3.44E-23	postive
VEGFA	AC078864.1	0.519744535	1.26E-38	postive
TUBE1	AC078864.1	0.531880651	1.08E-40	postive
ALOX12	AC078864.1	0.54002703	3.96E-42	postive
TAZ	AC078864.1	0.466837641	1.59E-30	postive
ZNF419	AC009404.1	0.479746885	2.25E-32	postive
TUBE1	AC009404.1	0.44759604	6.49E-28	postive
ALOX12	AC009404.1	0.545543629	4.01E-43	postive
TAZ	AC009404.1	0.477306795	5.10E-32	postive
HELLS	AC012531.1	0.40283234	1.91E-22	postive
ZNF419	AC012531.1	0.444729424	1.54E-27	postive

TUBE1	AC012531.1	0.522096989	5.07E-39	postive
SETD1B	AC012531.1	0.508318217	9.35E-37	postive
ALOX12	AC012531.1	0.639624342	2.38E-63	postive
IREB2	AC012531.1	0.413093118	1.25E-23	postive
SP1	AC012531.1	0.482339143	9.36E-33	postive
GABPB1	AC012531.1	0.475313811	9.90E-32	postive
MAPK8	AC012531.1	0.45186226	1.77E-28	postive
LINC00472	AC012531.1	0.480373657	1.82E-32	postive
ATM	AC012531.1	0.710667031	4.73E-84	postive
FBXW7	AC012531.1	0.448412509	5.07E-28	postive
FANCD2	AL445645.1	0.495450391	9.89E-35	postive
HELLS	AL445645.1	0.457975504	2.65E-29	postive
HELLS	AC007216.4	0.445731369	1.14E-27	postive
ZNF419	AC007216.4	0.509185857	6.78E-37	postive
KLHL24	AC007216.4	0.47687439	5.89E-32	postive
TUBE1	AC007216.4	0.54999516	6.12E-44	postive
ALOX12	AC007216.4	0.545481419	4.11E-43	postive
GABPB1	AC007216.4	0.56890935	1.51E-47	postive
MAPK8	AC007216.4	0.427109696	2.61E-25	postive
LINC00472	AC007216.4	0.773685449	1.63E-108	postive
ATM	AC007216.4	0.666106325	2.13E-70	postive
FBXW7	AC007216.4	0.505734615	2.42E-36	postive
MUC1	MIR31HG	0.417437985	3.85E-24	postive
CD44	MIR31HG	0.554142095	1.04E-44	postive
SOCS1	MIR31HG	0.492972897	2.37E-34	postive
TFAP2C	PCAT1	0.516543202	4.28E-38	postive
HBA1	PCAT1	0.517023826	3.56E-38	postive
DUOX1	PCAT1	0.449853339	3.27E-28	postive
BRD4	AC011462.4	0.424763536	5.05E-25	postive
ZNF419	AC011462.4	0.553546968	1.34E-44	postive
VEGFA	AC011462.4	0.427906913	2.08E-25	postive
TUBE1	AC011462.4	0.559308514	1.10E-45	postive
DRD4	AC011462.4	0.475005055	1.10E-31	postive
ALOX12	AC011462.4	0.65094594	2.82E-66	postive
PHKG2	AC011462.4	0.579479178	1.15E-49	postive
TAZ	AC011462.4	0.677744704	9.89E-74	postive
ZNF419	AL135960.1	0.469386099	6.94E-31	postive
VEGFA	AL135960.1	0.412382223	1.52E-23	postive
TUBE1	AL135960.1	0.517474471	3.00E-38	postive
ALOX12	AL135960.1	0.519210772	1.54E-38	postive
GABPB1	AL135960.1	0.506602144	1.76E-36	postive
ZEB1	AL135960.1	0.540222652	3.65E-42	postive
MAPK8	AL135960.1	0.482514007	8.82E-33	postive
ATM	AL135960.1	0.500082609	1.89E-35	postive
FBXW7	AL135960.1	0.467686578	1.20E-30	postive
FANCD2	AL683813.1	0.427489728	2.34E-25	postive
HELLS	AL683813.1	0.49819954	3.72E-35	postive
BRD4	AL683813.1	0.405390175	9.76E-23	postive
ZNF419	AL683813.1	0.618415205	3.48E-58	postive
TUBE1	AL683813.1	0.581401408	4.64E-50	postive
ALOX12	AL683813.1	0.617996378	4.37E-58	postive
GABPB1	AL683813.1	0.499953011	1.98E-35	postive
LINC00472	AL683813.1	0.551697473	2.96E-44	postive
LPIN1	AL683813.1	0.408459457	4.34E-23	postive
ATM	AL683813.1	0.494121734	1.58E-34	postive
FBXW7	AL683813.1	0.487907073	1.39E-33	postive
HELLS	Z99289.1	0.46847777	9.32E-31	postive
TUBE1	Z99289.1	0.423883156	6.47E-25	postive

GABPB1	Z99289.1	0.558360794	1.66E-45	postive
ATG7	Z99289.1	0.407314887	5.88E-23	postive
MAPK8	Z99289.1	0.407506812	5.59E-23	postive
LINC00472	Z99289.1	0.715684628	9.40E-86	postive
ATM	Z99289.1	0.670444056	1.27E-71	postive
FBXW7	Z99289.1	0.48598629	2.69E-33	postive
FANCD2	AC087645.2	0.407186103	6.08E-23	postive
HSPB1	AL121899.1	0.514837163	8.16E-38	postive
HBA1	AL121899.1	0.497548073	4.69E-35	postive
HRAS	AL121899.1	0.52310783	3.43E-39	postive
EGLN2	AL121899.1	0.550739134	4.46E-44	postive
FANCD2	AC023043.3	0.445020927	1.41E-27	postive
TFAP2C	AC023043.3	0.493203669	2.19E-34	postive
HBA1	AC023043.3	0.409639148	3.17E-23	postive
FANCD2	AC004923.4	0.419381669	2.26E-24	postive
TFAP2C	AC004923.4	0.555872958	4.91E-45	postive
HBA1	AC004923.4	0.501628205	1.08E-35	postive
DUOX1	AC004923.4	0.414319718	9.01E-24	postive
WIPI2	AL031123.2	0.406772344	6.78E-23	postive
KLHL24	AC079380.1	0.455791324	5.25E-29	postive
GABPB1	AC079380.1	0.461430894	8.93E-30	postive
MAPK8	AC079380.1	0.440788256	4.98E-27	postive
LINC00472	AC079380.1	0.505358552	2.78E-36	postive
PRKAA1	AC079380.1	0.458274915	2.42E-29	postive
ATM	AC079380.1	0.534077112	4.46E-41	postive
FBXW7	AC079380.1	0.50273706	7.24E-36	postive
HELLS	AP001793.1	0.515567644	6.19E-38	postive
ZNF419	AP001793.1	0.55367043	1.27E-44	postive
TUBE1	AP001793.1	0.616744081	8.56E-58	postive
SETD1B	AP001793.1	0.446948478	7.90E-28	postive
ALOX12	AP001793.1	0.613093076	5.98E-57	postive
GABPB1	AP001793.1	0.59654893	2.94E-53	postive
MAPK8	AP001793.1	0.453874222	9.51E-29	postive
LINC00472	AP001793.1	0.477353686	5.02E-32	postive
ATM	AP001793.1	0.707913815	3.92E-83	postive
FBXW7	AP001793.1	0.580611266	6.74E-50	postive
ZNF419	MUC20-OT1	0.472882305	2.21E-31	postive
KLHL24	MUC20-OT1	0.41894872	2.54E-24	postive
TUBE1	MUC20-OT1	0.517616639	2.84E-38	postive
ALOX12	MUC20-OT1	0.520641282	8.91E-39	postive
LINC00472	MUC20-OT1	0.607217905	1.30E-55	postive
ATM	MUC20-OT1	0.441993395	3.49E-27	postive
NCF2	LINC02285	0.540167971	3.73E-42	postive
CYBB	LINC02285	0.517574111	2.89E-38	postive
TSC22D3	AC012065.2	0.400337557	3.64E-22	postive
HRAS	AC012065.2	0.458401667	2.32E-29	postive
EGLN2	AC012065.2	0.523817315	2.60E-39	postive
VEGFA	AL132780.2	0.468986745	7.90E-31	postive
ALOX12	AL132780.2	0.500399262	1.69E-35	postive
ZNF419	LGR4-AS1	0.419409444	2.24E-24	postive
ALOX12	LGR4-AS1	0.430329749	1.04E-25	postive
TFAP2C	LGR4-AS1	0.457496776	3.08E-29	postive
DUOX1	LGR4-AS1	0.413743395	1.05E-23	postive
ALOX12	AC136475.5	0.443862386	2.00E-27	postive
HBA1	AC136475.5	0.437308508	1.39E-26	postive
MUC1	AC241644.2	0.423389295	7.43E-25	postive
HELLS	GAS5-AS1	0.409800755	3.04E-23	postive
TUBE1	GAS5-AS1	0.46015216	1.34E-29	postive

GABPB1	GAS5-AS1	0.475783666	8.47E-32	postive
LINC00472	GAS5-AS1	0.593629966	1.25E-52	postive
ATM	GAS5-AS1	0.527997294	5.05E-40	postive
ZNF419	AC015802.4	0.454225242	8.53E-29	postive
TUBE1	AC015802.4	0.466220515	1.93E-30	postive
ALOX12	AC015802.4	0.576948742	3.75E-49	postive
PHKG2	AC015802.4	0.604639099	4.90E-55	postive
TAZ	AC015802.4	0.662084324	2.79E-69	postive
TFAP2C	AL139260.1	0.409124933	3.63E-23	postive
ZNF419	AL122010.1	0.429422259	1.35E-25	postive
TUBE1	AL122010.1	0.422439112	9.68E-25	postive
ALOX12	AL122010.1	0.478593352	3.31E-32	postive
GABPB1	AL122010.1	0.450108834	3.02E-28	postive
TUBE1	AC016727.1	0.529468437	2.82E-40	postive
ALOX12	AC016727.1	0.540674477	3.03E-42	postive
MAPK8	AC016727.1	0.447966526	5.80E-28	postive
ATM	AC016727.1	0.529651369	2.62E-40	postive
AGPAT3	AC008991.1	0.410468372	2.54E-23	postive
ELAVL1	AC008991.1	0.446573009	8.85E-28	postive
TUBE1	ZBTB44-DT	0.418583677	2.81E-24	postive
ALOX12	ZBTB44-DT	0.482467862	8.96E-33	postive
PML	USP30-AS1	0.422320619	1.00E-24	postive
GCH1	USP30-AS1	0.483415007	6.49E-33	postive
IFNG	USP30-AS1	0.631116933	3.14E-61	postive
VDAC2	SNHG29	0.586885303	3.37E-51	postive
CHMP5	SNHG29	0.473576461	1.76E-31	postive
RPL8	SNHG29	0.451137397	2.21E-28	postive
YWHAE	SNHG29	0.464173253	3.73E-30	postive
FANCD2	AC022400.4	0.450251883	2.90E-28	postive
HELLS	AC022400.4	0.564860962	9.36E-47	postive
ZNF419	AC022400.4	0.485031237	3.74E-33	postive
TUBE1	AC022400.4	0.589666585	8.76E-52	postive
ALOX12	AC022400.4	0.720851729	1.52E-87	postive
GABPB1	AC022400.4	0.462420715	6.52E-30	postive
ATM	AC022400.4	0.577000658	3.66E-49	postive
FBXW7	AC022400.4	0.529767405	2.50E-40	postive
NCF2	AC090559.1	0.534311322	4.06E-41	postive
MAPK14	AC090559.1	0.433363102	4.37E-26	postive
CYBB	AC090559.1	0.695033749	5.62E-79	postive
BECN1	AC090559.1	0.41371887	1.06E-23	postive
TNFAIP3	AC090559.1	0.433700334	3.96E-26	postive
TLR4	AC090559.1	0.518153056	2.31E-38	postive
SLC3A2	LINC02608	0.440596407	5.28E-27	postive
FH	LINC02608	0.483563917	6.17E-33	postive
ISCU	LINC02608	0.581330113	4.80E-50	postive
ALB	LINC02608	0.431705397	7.04E-26	postive
GPX2	LINC02608	0.423722376	6.77E-25	postive
GABARAPL1	LINC02608	0.627506427	2.38E-60	postive
CDO1	LINC02608	0.420297638	1.75E-24	postive
LPIN1	LINC02608	0.530663103	1.75E-40	postive
KLHL24	AC093726.1	0.443283896	2.37E-27	postive
LINC00472	AC093726.1	0.405968642	8.39E-23	postive
HELLS	AC025766.1	0.510178383	4.69E-37	postive
ZNF419	AC025766.1	0.49105754	4.64E-34	postive
TUBE1	AC025766.1	0.619129388	2.37E-58	postive
ALOX12	AC025766.1	0.646072563	5.31E-65	postive
GABPB1	AC025766.1	0.522720699	3.98E-39	postive
ATM	AC025766.1	0.682177023	4.84E-75	postive

FBXW7	AC025766.1	0.494905147	1.20E-34	postive
PHKG2	AC020765.2	0.588517332	1.53E-51	postive
TAZ	AC020765.2	0.6541432	3.99E-67	postive
KLHL24	AC092894.1	0.543457864	9.58E-43	postive
LINC00472	AC092894.1	0.578493149	1.82E-49	postive
ZNF419	AC245060.5	0.507775152	1.14E-36	postive
TUBE1	AC245060.5	0.572145897	3.46E-48	postive
ALOX12	AC245060.5	0.535738651	2.28E-41	postive
GABPB1	AC245060.5	0.419377682	2.26E-24	postive
LINC00472	AC245060.5	0.490248103	6.16E-34	postive
ATM	AC245060.5	0.472916875	2.19E-31	postive
FBXW7	AC245060.5	0.405435069	9.65E-23	postive
HELLS	AC130324.1	0.406311163	7.66E-23	postive
ALOX12	AC130324.1	0.412059275	1.66E-23	postive
TFAP2C	AC130324.1	0.521894101	5.49E-39	postive
HBA1	AC130324.1	0.507212332	1.41E-36	postive
DUOX1	AC130324.1	0.429926023	1.17E-25	postive
GPX4	AC027644.3	0.46066134	1.14E-29	postive
HSPB1	AC027644.3	0.555154136	6.70E-45	postive
STAT3	AC027644.3	-0.430465889	1.00E-25	negative
MAPK14	AC027644.3	-0.423842481	6.54E-25	negative
HBA1	AC027644.3	0.45197809	1.71E-28	postive
NRAS	AC027644.3	-0.401443512	2.74E-22	negative
HRAS	AC027644.3	0.670364088	1.34E-71	postive
NCOA4	AC027644.3	-0.424259957	5.82E-25	negative
PHKG2	AC027644.3	0.466680885	1.67E-30	postive
BECN1	AC027644.3	-0.458737336	2.09E-29	negative
MAP1LC3A	AC027644.3	0.488135045	1.28E-33	postive
ANO6	AC027644.3	-0.443871456	1.99E-27	negative
EGLN2	AC027644.3	0.617984211	4.39E-58	postive
TAZ	AC027644.3	0.404003982	1.40E-22	postive
HELLS	AC007566.1	0.438129723	1.09E-26	postive
ZNF419	AC007566.1	0.531543048	1.23E-40	postive
VEGFA	AC007566.1	0.503019315	6.53E-36	postive
TUBE1	AC007566.1	0.712210962	1.43E-84	postive
SETD1B	AC007566.1	0.418130616	3.19E-24	postive
ALOX12	AC007566.1	0.730916663	3.75E-91	postive
GABPB1	AC007566.1	0.481695409	1.16E-32	postive
ATM	AC007566.1	0.675934928	3.34E-73	postive
TAZ	AC007566.1	0.493266152	2.14E-34	postive
FBXW7	AC007566.1	0.514421143	9.55E-38	postive
TFR2	AC007785.1	0.452859249	1.30E-28	postive
IDH1	AC007785.1	0.506632429	1.74E-36	postive
GPX4	TP53TG1	0.580922774	5.81E-50	postive
HSPB1	TP53TG1	0.511521775	2.84E-37	postive
STAT3	TP53TG1	-0.481698254	1.16E-32	negative
OTUB1	TP53TG1	0.406154247	7.99E-23	postive
OXSR1	TP53TG1	-0.478310573	3.64E-32	negative
RPL8	TP53TG1	0.564718968	9.98E-47	postive
ATP5MC3	TP53TG1	0.44280474	2.74E-27	postive
IREB2	TP53TG1	-0.43696467	1.53E-26	negative
SP1	TP53TG1	-0.405109476	1.05E-22	negative
HRAS	TP53TG1	0.42655677	3.05E-25	postive
BECN1	TP53TG1	-0.434334078	3.30E-26	negative
GABARAPL2	TP53TG1	0.445924222	1.08E-27	postive
ZEB1	TP53TG1	-0.404511027	1.23E-22	negative
MAPK8	TP53TG1	-0.400826077	3.21E-22	negative
SIRT1	TP53TG1	-0.408682437	4.09E-23	negative

TUBE1	AC026367.3	0.47021762	5.29E-31	postive
ATM	AC093281.2	0.42986833	1.19E-25	postive
SLC3A2	AC124798.1	0.407281428	5.93E-23	postive
ATG4D	AC124798.1	0.525924713	1.14E-39	postive
ZNF419	STK24-AS1	0.504756972	3.47E-36	postive
TUBE1	STK24-AS1	0.46440407	3.47E-30	postive
ALOX12	STK24-AS1	0.494941686	1.18E-34	postive
GABPB1	STK24-AS1	0.441918816	3.56E-27	postive
PHKG2	STK24-AS1	0.462986316	5.45E-30	postive
TAZ	STK24-AS1	0.551819755	2.81E-44	postive
HRAS	AL844908.2	0.636579208	1.39E-62	postive
EGLN2	AL844908.2	0.648443669	1.28E-65	postive
VEGFA	SMAD9-IT1	0.524411143	2.06E-39	postive
TUBE1	SMAD9-IT1	0.418493947	2.88E-24	postive
SETD1B	SMAD9-IT1	0.44815253	5.49E-28	postive
ALOX12	SMAD9-IT1	0.534640216	3.55E-41	postive
PHKG2	AL590705.1	0.46961753	6.44E-31	postive
HELLS	AC024270.3	0.445360021	1.27E-27	postive
ZNF419	AC024270.3	0.407671778	5.35E-23	postive
KLHL24	AC024270.3	0.483656719	5.98E-33	postive
TUBE1	AC024270.3	0.456137985	4.71E-29	postive
ALOX12	AC024270.3	0.46488811	2.97E-30	postive
IREB2	AC024270.3	0.46751643	1.27E-30	postive
GABPB1	AC024270.3	0.575950993	5.97E-49	postive
PIK3CA	AC024270.3	0.441722301	3.78E-27	postive
ATG7	AC024270.3	0.420280192	1.76E-24	postive
MAPK8	AC024270.3	0.443189507	2.44E-27	postive
LINC00472	AC024270.3	0.764622236	1.58E-104	postive
ATM	AC024270.3	0.720706527	1.71E-87	postive
FBXW7	AC024270.3	0.471048887	4.04E-31	postive
HELLS	AC012409.4	0.519725313	1.27E-38	postive
TUBE1	AC012409.4	0.428933234	1.56E-25	postive
ALOX12	AC012409.4	0.430646243	9.54E-26	postive
GABPB1	AC012409.4	0.503859522	4.81E-36	postive
ATG7	AC012409.4	0.403682765	1.53E-22	postive
LINC00472	AC012409.4	0.526189815	1.03E-39	postive
ATM	AC012409.4	0.706547124	1.11E-82	postive
FBXW7	AC012409.4	0.451624684	1.90E-28	postive
HELLS	WDR5B-DT	0.431339481	7.82E-26	postive
ZNF419	WDR5B-DT	0.405527914	9.42E-23	postive
TUBE1	WDR5B-DT	0.517351935	3.14E-38	postive
ALOX12	WDR5B-DT	0.490191947	6.28E-34	postive
GABPB1	WDR5B-DT	0.540182053	3.71E-42	postive
MAPK8	WDR5B-DT	0.447594694	6.50E-28	postive
LINC00472	WDR5B-DT	0.625878664	5.89E-60	postive
ATM	WDR5B-DT	0.621098871	8.14E-59	postive
HSPB1	AC147067.1	0.509137397	6.90E-37	postive
GCLC	AC147067.1	-0.403759205	1.50E-22	negative
SLC2A6	AC147067.1	0.577885886	2.42E-49	postive
IREB2	AC147067.1	-0.468901463	8.13E-31	negative
SCP2	AC147067.1	-0.411306838	2.03E-23	negative
LPCAT3	AC147067.1	-0.40434874	1.28E-22	negative
HRAS	AC147067.1	0.58976015	8.37E-52	postive
NCOA4	AC147067.1	-0.465044649	2.82E-30	negative
MAPK1	AC147067.1	-0.478379131	3.56E-32	negative
BID	AC147067.1	0.400951351	3.11E-22	postive
PRKAA2	AC147067.1	-0.459246468	1.78E-29	negative
EGLN2	AC147067.1	0.511350456	3.03E-37	postive

ALOX12	AC008750.1	0.451103884	2.23E-28	postive
IFNG	AC008750.1	0.41836047	2.99E-24	postive
TAZ	AC008750.1	0.415570033	6.41E-24	postive
HELLS	DUBR	0.402639985	2.01E-22	postive
IREB2	DUBR	0.444449805	1.68E-27	postive
GABPB1	DUBR	0.500787515	1.47E-35	postive
PIK3CA	DUBR	0.49619268	7.60E-35	postive
KRAS	DUBR	0.420046406	1.88E-24	postive
ZEB1	DUBR	0.437730986	1.23E-26	postive
MAPK8	DUBR	0.563200906	1.96E-46	postive
PRKAA1	DUBR	0.416997679	4.34E-24	postive
ATM	DUBR	0.50731812	1.35E-36	postive
SIRT1	DUBR	0.408489491	4.30E-23	postive
ATM	AC015977.1	0.475923722	8.08E-32	postive
HELLS	AC019186.1	0.446762298	8.36E-28	postive
TUBE1	AC019186.1	0.447857159	6.00E-28	postive
ALOX12	AC019186.1	0.421192984	1.37E-24	postive
GABPB1	AC019186.1	0.427646048	2.24E-25	postive
LINC00472	AC019186.1	0.413073545	1.26E-23	postive
ATM	AC019186.1	0.407763338	5.22E-23	postive
TAZ	AC019186.1	0.43286154	5.05E-26	postive
FBXW7	AC019186.1	0.421641474	1.21E-24	postive
HELLS	FMR1-IT1	0.457949262	2.68E-29	postive
ZNF419	FMR1-IT1	0.558197298	1.78E-45	postive
KLHL24	FMR1-IT1	0.40700319	6.38E-23	postive
TUBE1	FMR1-IT1	0.586987966	3.21E-51	postive
SETD1B	FMR1-IT1	0.463027799	5.38E-30	postive
ALOX12	FMR1-IT1	0.599917245	5.43E-54	postive
GABPB1	FMR1-IT1	0.530299857	2.03E-40	postive
MAPK8	FMR1-IT1	0.405621368	9.19E-23	postive
LINC00472	FMR1-IT1	0.656380266	1.00E-67	postive
ATM	FMR1-IT1	0.65104231	2.66E-66	postive
FBXW7	FMR1-IT1	0.501423809	1.16E-35	postive
FANCD2	AC006160.1	0.419796307	2.01E-24	postive
HELLS	AC006160.1	0.420849768	1.51E-24	postive
ALOX12	AC006160.1	0.42677681	2.87E-25	postive
TFAP2C	AC006160.1	0.534548753	3.69E-41	postive
HBA1	AC006160.1	0.474980827	1.11E-31	postive
DUOX1	AC006160.1	0.432596262	5.45E-26	postive
HELLS	Z98200.1	0.458541825	2.22E-29	postive
TUBE1	Z98200.1	0.511277683	3.11E-37	postive
ALOX12	Z98200.1	0.499136885	2.66E-35	postive
GABPB1	Z98200.1	0.445594684	1.19E-27	postive
LINC00472	Z98200.1	0.540760069	2.93E-42	postive
ATM	Z98200.1	0.523217937	3.28E-39	postive
FBXW7	Z98200.1	0.441147617	4.48E-27	postive
HSPB1	FOXC2-AS1	0.458306338	2.39E-29	postive
HBA1	FOXC2-AS1	0.563185958	1.98E-46	postive
HRAS	FOXC2-AS1	0.649890492	5.35E-66	postive
EGLN2	FOXC2-AS1	0.786861043	1.18E-114	postive
RPL8	LINC02482	0.40658857	7.12E-23	postive
MAPK14	LINC02482	-0.40841152	4.39E-23	negative
BECN1	LINC02482	-0.401674616	2.58E-22	negative
ZNF419	AL365330.1	0.539792468	4.36E-42	postive
VEGFA	AL365330.1	0.573238823	2.09E-48	postive
TUBE1	AL365330.1	0.560260818	7.21E-46	postive
SETD1B	AL365330.1	0.552615114	2.00E-44	postive
DRD4	AL365330.1	0.41792436	3.37E-24	postive

ALOX12	AL365330.1	0.656904943	7.22E-68	postive
PHKG2	AL365330.1	0.428293024	1.87E-25	postive
TAZ	AL365330.1	0.659459455	1.46E-68	postive
FANCD2	AC008083.3	0.528376731	4.34E-40	postive
HELLS	AC008083.3	0.405494697	9.50E-23	postive
TFAP2C	AC008083.3	0.428985536	1.53E-25	postive
HBA1	AC008083.3	0.407694213	5.32E-23	postive
IFNG	AC008083.3	0.416380534	5.14E-24	postive
FBXW7	AC008083.3	0.410816529	2.31E-23	postive
HELLS	AL139807.1	0.552784219	1.86E-44	postive
TUBE1	AL139807.1	0.408094633	4.78E-23	postive
ALOX12	AL139807.1	0.442471327	3.02E-27	postive
GABPB1	AL139807.1	0.527962998	5.12E-40	postive
ATG7	AL139807.1	0.442352475	3.13E-27	postive
LINC00472	AL139807.1	0.610663509	2.15E-56	postive
ATM	AL139807.1	0.715059361	1.54E-85	postive
FBXW7	AL139807.1	0.459151808	1.83E-29	postive
GCLC	LINC01507	0.450559556	2.64E-28	postive
IREB2	LINC01507	0.492136211	3.18E-34	postive
NCOA4	LINC01507	0.494839938	1.23E-34	postive
SNX4	LINC01507	0.484028363	5.27E-33	postive
MAPK1	LINC01507	0.557472845	2.45E-45	postive
MAPK8	LINC01507	0.551720711	2.93E-44	postive
PRKAA2	LINC01507	0.587445096	2.57E-51	postive
ATM	LINC01507	0.532136112	9.73E-41	postive
SIRT1	LINC01507	0.478722275	3.17E-32	postive
GCH1	AC090152.1	0.401266507	2.87E-22	postive
IFNG	AC090152.1	0.49724438	5.23E-35	postive
FBXW7	AL355075.4	0.495129162	1.11E-34	postive
ZNF419	FLNB-AS1	0.481985501	1.06E-32	postive
KLHL24	FLNB-AS1	0.414346167	8.94E-24	postive
ALOX12	FLNB-AS1	0.433087294	4.73E-26	postive
GABPB1	FLNB-AS1	0.46132478	9.24E-30	postive
LINC00472	FLNB-AS1	0.636808416	1.22E-62	postive
ATM	FLNB-AS1	0.474133064	1.46E-31	postive
HSPB1	UBL7-AS1	-0.43179008	6.87E-26	negative
KLHL24	UBL7-AS1	0.430805125	9.12E-26	postive
EIF2AK4	UBL7-AS1	0.46813148	1.04E-30	postive
IREB2	UBL7-AS1	0.551350548	3.44E-44	postive
PIK3CA	UBL7-AS1	0.461396172	9.03E-30	postive
ATG5	UBL7-AS1	0.427999551	2.03E-25	postive
ATG7	UBL7-AS1	0.409996164	2.88E-23	postive
MAPK8	UBL7-AS1	0.426382477	3.21E-25	postive
PRKAA1	UBL7-AS1	0.455514707	5.72E-29	postive
TLR4	UBL7-AS1	0.404805331	1.14E-22	postive
ATM	UBL7-AS1	0.471268698	3.75E-31	postive
KLHL24	AC024940.5	0.616639884	9.05E-58	postive
PIK3CA	AC024940.5	0.403962814	1.42E-22	postive
KRAS	AC024940.5	0.417569347	3.72E-24	postive
LINC00472	AC024940.5	0.838958341	5.17E-144	postive
LPIN1	AC024940.5	0.42705666	2.65E-25	postive
FANCD2	AC137932.3	0.450511803	2.67E-28	postive
HELLS	AC137932.3	0.506751483	1.67E-36	postive
ZNF419	AC137932.3	0.476317041	7.09E-32	postive
TUBE1	AC137932.3	0.4452865	1.30E-27	postive
ALOX12	AC137932.3	0.597372806	1.95E-53	postive
FBXW7	AC137932.3	0.424563914	5.35E-25	postive
ALOX12	PRANCR	0.410109318	2.80E-23	postive

PHKG2	PRANCR	0.585281916	7.30E-51	postive
TAZ	PRANCR	0.653995355	4.37E-67	postive
HBA1	AC012360.2	0.546255017	2.97E-43	postive
NCOA4	AC108673.2	-0.405629424	9.17E-23	negative
CHMP6	AP001160.3	0.455822659	5.20E-29	postive
PEBP1	AP001160.3	0.443865177	2.00E-27	postive
PLIN2	AC002401.3	0.411530092	1.91E-23	postive
HNF4A	AC002401.3	0.403089332	1.78E-22	postive
PEBP1	AC002401.3	0.43881789	8.91E-27	postive
ALB	F11-AS1	0.429212274	1.44E-25	postive
CDO1	F11-AS1	0.418710538	2.72E-24	postive
HELLS	AL158163.2	0.499876525	2.04E-35	postive
ZNF419	AL158163.2	0.438984252	8.49E-27	postive
KLHL24	AL158163.2	0.449875851	3.25E-28	postive
TUBE1	AL158163.2	0.545697383	3.76E-43	postive
ALOX12	AL158163.2	0.465692981	2.29E-30	postive
IREB2	AL158163.2	0.416419187	5.09E-24	postive
GABPB1	AL158163.2	0.625341235	7.93E-60	postive
PIK3CA	AL158163.2	0.405136579	1.04E-22	postive
MAPK8	AL158163.2	0.48046296	1.77E-32	postive
LINC00472	AL158163.2	0.798218658	2.63E-120	postive
ATM	AL158163.2	0.698028632	6.35E-80	postive
FBXW7	AL158163.2	0.493639702	1.88E-34	postive
HELLS	AC096586.2	0.42844136	1.79E-25	postive
ZNF419	AC096586.2	0.443451717	2.26E-27	postive
KLHL24	AC096586.2	0.559788716	8.87E-46	postive
TUBE1	AC096586.2	0.463222752	5.05E-30	postive
IREB2	AC096586.2	0.456487123	4.23E-29	postive
GABPB1	AC096586.2	0.574924816	9.61E-49	postive
PIK3CA	AC096586.2	0.463515318	4.60E-30	postive
KRAS	AC096586.2	0.431774275	6.91E-26	postive
MAPK8	AC096586.2	0.458873427	2.00E-29	postive
LINC00472	AC096586.2	0.898810374	1.71E-194	postive
PRKAA1	AC096586.2	0.411337739	2.01E-23	postive
ATM	AC096586.2	0.585848707	5.56E-51	postive
FBXW7	AC096586.2	0.443738516	2.07E-27	postive
HELLS	AL353804.2	0.511256139	3.14E-37	postive
KLHL24	AL353804.2	0.419957987	1.93E-24	postive
TUBE1	AL353804.2	0.462977419	5.46E-30	postive
ALOX12	AL353804.2	0.417785337	3.50E-24	postive
GABPB1	AL353804.2	0.576125223	5.51E-49	postive
PIK3CA	AL353804.2	0.400262907	3.72E-22	postive
ATG7	AL353804.2	0.410767799	2.34E-23	postive
MAPK8	AL353804.2	0.412954339	1.30E-23	postive
LINC00472	AL353804.2	0.807642647	2.80E-125	postive
ATM	AL353804.2	0.698570013	4.27E-80	postive
FBXW7	AL353804.2	0.451655757	1.88E-28	postive
ZNF419	ZNF674-AS1	0.47317849	2.01E-31	postive
EIF2S1	ZNF674-AS1	0.424844201	4.94E-25	postive
GABPB1	ZNF674-AS1	0.438303791	1.04E-26	postive
HELLS	AC053527.2	0.530852176	1.63E-40	postive
ZNF419	AC053527.2	0.430333782	1.04E-25	postive
TUBE1	AC053527.2	0.565067855	8.54E-47	postive
ALOX12	AC053527.2	0.579174571	1.33E-49	postive
GABPB1	AC053527.2	0.582320199	3.00E-50	postive
MAPK8	AC053527.2	0.465990723	2.08E-30	postive
LINC00472	AC053527.2	0.610174283	2.78E-56	postive
ATM	AC053527.2	0.768845048	2.31E-106	postive

FBXW7	AC053527.2	0.510835299	3.67E-37	postive
BRD4	AC012645.4	0.436764739	1.63E-26	postive
ZNF419	AC012645.4	0.486273258	2.44E-33	postive
TUBE1	AC012645.4	0.445997357	1.05E-27	postive
DRD4	AC012645.4	0.448572492	4.83E-28	postive
ALOX12	AC012645.4	0.539767275	4.40E-42	postive
PHKG2	AC012645.4	0.557722782	2.19E-45	postive
TAZ	AC012645.4	0.669166777	2.93E-71	postive
MTOR	AF131215.5	0.410404819	2.58E-23	postive
KLHL24	AF131215.5	0.456398811	4.34E-29	postive
IREB2	AF131215.5	0.432321132	5.90E-26	postive
GABPB1	AF131215.5	0.454096459	8.88E-29	postive
PIK3CA	AF131215.5	0.41817205	3.15E-24	postive
ATG7	AF131215.5	0.401280607	2.86E-22	postive
LINC00472	AF131215.5	0.477682276	4.50E-32	postive
LPIN1	AF131215.5	0.405997288	8.32E-23	postive
ATM	AF131215.5	0.513474458	1.37E-37	postive
FBXW7	AF131215.5	0.412517161	1.47E-23	postive
ALOX12	AP001178.1	0.42356629	7.07E-25	postive
TFAP2C	AP001178.1	0.52020224	1.05E-38	postive
HBA1	AP001178.1	0.48542159	3.27E-33	postive
DUOX1	AP001178.1	0.402362113	2.16E-22	postive
ZNF419	AC006001.2	0.407642743	5.39E-23	postive
ALOX12	AC006001.2	0.518513004	2.02E-38	postive
TFAP2C	AC022146.2	0.487589147	1.55E-33	postive
HBA1	AC022146.2	0.452093465	1.65E-28	postive
LINC00472	AC022146.2	0.520783666	8.43E-39	postive
HELLS	NADK2-AS1	0.479550593	2.40E-32	postive
KLHL24	NADK2-AS1	0.487123226	1.82E-33	postive
TUBE1	NADK2-AS1	0.466516567	1.76E-30	postive
ALOX12	NADK2-AS1	0.437621055	1.27E-26	postive
IREB2	NADK2-AS1	0.472489123	2.52E-31	postive
GABPB1	NADK2-AS1	0.553798414	1.20E-44	postive
PIK3CA	NADK2-AS1	0.455572251	5.62E-29	postive
MAPK8	NADK2-AS1	0.468523605	9.19E-31	postive
LINC00472	NADK2-AS1	0.881729718	2.38E-177	postive
PRKAA1	NADK2-AS1	0.400108347	3.87E-22	postive
ATM	NADK2-AS1	0.691301391	8.20E-78	postive
FBXW7	NADK2-AS1	0.443109759	2.50E-27	postive
IREB2	LINC02256	0.422381129	9.84E-25	postive
ATM	LINC02256	0.42932901	1.39E-25	postive
HELLS	AC098851.1	0.521716112	5.88E-39	postive
TUBE1	AC098851.1	0.547445724	1.80E-43	postive
ALOX12	AC098851.1	0.581664758	4.09E-50	postive
GABPB1	AC098851.1	0.514008989	1.12E-37	postive
LINC00472	AC098851.1	0.575256351	8.24E-49	postive
ATM	AC098851.1	0.735030381	1.13E-92	postive
FBXW7	AC098851.1	0.489654058	7.58E-34	postive
HELLS	AL353804.1	0.43518926	2.57E-26	postive
KLHL24	AL353804.1	0.524403639	2.07E-39	postive
TUBE1	AL353804.1	0.426702267	2.93E-25	postive
IREB2	AL353804.1	0.500046468	1.91E-35	postive
GABPB1	AL353804.1	0.58023427	8.05E-50	postive
PIK3CA	AL353804.1	0.511829825	2.53E-37	postive
KRAS	AL353804.1	0.430725576	9.33E-26	postive
MAPK8	AL353804.1	0.496331314	7.23E-35	postive
LINC00472	AL353804.1	0.880539192	2.96E-176	postive
PRKAA1	AL353804.1	0.431114206	8.35E-26	postive

ATM	AL353804.1	0.691792888	5.77E-78	postive
FBXW7	AL353804.1	0.455128009	6.45E-29	postive
ISCU	OLMALINC	0.53352416	5.57E-41	postive
DDIT3	OLMALINC	0.513589688	1.31E-37	postive
ATG4D	OLMALINC	0.563866609	1.46E-46	postive
GABARAPL1	OLMALINC	0.576181065	5.37E-49	postive
LPIN1	OLMALINC	0.417155528	4.16E-24	postive
ISCU	AC129507.4	0.421355787	1.31E-24	postive
CHAC1	AC129507.4	0.469383157	6.95E-31	postive
ATG4D	AC129507.4	0.4730226	2.11E-31	postive
MAP1LC3A	AC129507.4	0.515472656	6.42E-38	postive
GPX4	AC005288.1	-0.573641245	1.74E-48	negative
RB1	AC005288.1	0.578792933	1.59E-49	postive
HSPB1	AC005288.1	-0.470528534	4.78E-31	negative
GCLC	AC005288.1	0.409292299	3.48E-23	postive
NFE2L2	AC005288.1	0.508279196	9.49E-37	postive
STAT3	AC005288.1	0.593986763	1.05E-52	postive
MTOR	AC005288.1	0.496498779	6.82E-35	postive
ACSL3	AC005288.1	0.425321198	4.32E-25	postive
HIF1A	AC005288.1	0.440231701	5.88E-27	postive
OXSR1	AC005288.1	0.551270631	3.56E-44	postive
ZFP69B	AC005288.1	0.498172672	3.75E-35	postive
EIF2S1	AC005288.1	0.436562579	1.73E-26	postive
RPL8	AC005288.1	-0.486395149	2.34E-33	negative
MAP3K5	AC005288.1	0.42976546	1.23E-25	postive
MAPK14	AC005288.1	0.64224143	5.15E-64	postive
EIF2AK4	AC005288.1	0.553832288	1.19E-44	postive
IREB2	AC005288.1	0.633880339	6.55E-62	postive
SP1	AC005288.1	0.483009874	7.45E-33	postive
RIPK1	AC005288.1	0.470350582	5.07E-31	postive
PIK3CA	AC005288.1	0.579485667	1.14E-49	postive
NRAS	AC005288.1	0.544560186	6.05E-43	postive
KRAS	AC005288.1	0.503635437	5.22E-36	postive
HRAS	AC005288.1	-0.543938951	7.84E-43	negative
NCOA4	AC005288.1	0.47282596	2.25E-31	postive
PHKG2	AC005288.1	-0.453701273	1.00E-28	negative
BECN1	AC005288.1	0.708563629	2.38E-83	postive
MAP1LC3A	AC005288.1	-0.434595461	3.06E-26	negative
ULK2	AC005288.1	0.474552704	1.27E-31	postive
MAPK1	AC005288.1	0.458970075	1.94E-29	postive
ZEB1	AC005288.1	0.49982104	2.08E-35	postive
MAPK8	AC005288.1	0.512407323	2.04E-37	postive
PRKAA1	AC005288.1	0.513122488	1.56E-37	postive
ANO6	AC005288.1	0.432707721	5.28E-26	postive
TLR4	AC005288.1	0.492864822	2.46E-34	postive
MTDH	AC005288.1	0.529403467	2.89E-40	postive
SIRT1	AC005288.1	0.571416071	4.83E-48	postive
BACH1	AC005288.1	0.465858925	2.17E-30	postive
ZNF419	AC016737.1	0.460380114	1.25E-29	postive
VEGFA	AC016737.1	0.409902012	2.95E-23	postive
TUBE1	AC016737.1	0.497800114	4.29E-35	postive
DRD4	AC016737.1	0.401170248	2.94E-22	postive
ALOX12	AC016737.1	0.527896697	5.25E-40	postive
PHKG2	AC016737.1	0.610011294	3.03E-56	postive
TAZ	AC016737.1	0.693830442	1.34E-78	postive
HELLS	AL034550.1	0.428308848	1.86E-25	postive
BRD4	AL034550.1	0.422520146	9.46E-25	postive
ZNF419	AL034550.1	0.495455206	9.88E-35	postive

TUBE1	AL034550.1	0.518650278	1.91E-38	postive
SETD1B	AL034550.1	0.505867075	2.31E-36	postive
ALOX12	AL034550.1	0.558905869	1.31E-45	postive
GABPB1	AL034550.1	0.515560924	6.21E-38	postive
ZEB1	AL034550.1	0.458451112	2.29E-29	postive
MAPK8	AL034550.1	0.432693001	5.30E-26	postive
LINC00472	AL034550.1	0.461805871	7.93E-30	postive
ATM	AL034550.1	0.581598594	4.22E-50	postive
FBXW7	AL034550.1	0.496701962	6.34E-35	postive
TAZ	AC087741.2	0.424792268	5.01E-25	postive
HIC1	AC100803.3	0.511462076	2.90E-37	postive
ZEB1	AC100803.3	0.481940171	1.07E-32	postive
EPAS1	AC100803.3	0.520876162	8.13E-39	postive
TUBE1	AC006272.1	0.452403027	1.50E-28	postive
ALOX12	AC006272.1	0.512585773	1.91E-37	postive
ATM	AC006272.1	0.465371994	2.54E-30	postive
TAZ	AC006272.1	0.442070406	3.41E-27	postive
FBXW7	AC006272.1	0.440452686	5.50E-27	postive
HELLS	AC025171.2	0.40139831	2.77E-22	postive
ZNF419	AC025171.2	0.432951873	4.92E-26	postive
TUBE1	AC025171.2	0.593858441	1.12E-52	postive
SETD1B	AC025171.2	0.461094004	9.94E-30	postive
ALOX12	AC025171.2	0.59692925	2.43E-53	postive
GABPB1	AC025171.2	0.426998568	2.69E-25	postive
ATM	AC025171.2	0.582417244	2.86E-50	postive
FBXW7	AC025171.2	0.440988517	4.70E-27	postive
BRD4	RUSC1-AS1	0.408896084	3.86E-23	postive
ZNF419	RUSC1-AS1	0.618227774	3.85E-58	postive
VEGFA	RUSC1-AS1	0.519614488	1.32E-38	postive
TUBE1	RUSC1-AS1	0.660696486	6.71E-69	postive
SETD1B	RUSC1-AS1	0.527459516	6.24E-40	postive
DRD4	RUSC1-AS1	0.435686192	2.23E-26	postive
ALOX12	RUSC1-AS1	0.7608391	6.44E-103	postive
GABPB1	RUSC1-AS1	0.409332052	3.44E-23	postive
PHKG2	RUSC1-AS1	0.482899147	7.74E-33	postive
ATM	RUSC1-AS1	0.457426491	3.15E-29	postive
YY1AP1	RUSC1-AS1	0.443499247	2.23E-27	postive
TAZ	RUSC1-AS1	0.684773635	8.07E-76	postive
FBXW7	RUSC1-AS1	0.40233954	2.17E-22	postive
HBA1	HSD11B1-AS	0.553963195	1.12E-44	postive
HRAS	HSD11B1-AS	0.573025303	2.31E-48	postive
MAP1LC3A	HSD11B1-AS	0.407017795	6.36E-23	postive
EGLN2	HSD11B1-AS	0.737746528	1.07E-93	postive
HELLS	CEP250-AS1	0.452981637	1.25E-28	postive
ALOX12	CEP250-AS1	0.444801292	1.51E-27	postive
ATM	CEP250-AS1	0.503311428	5.87E-36	postive
FBXW7	CEP250-AS1	0.521477947	6.45E-39	postive
ZNF419	AL662844.4	0.483526201	6.25E-33	postive
VEGFA	AL662844.4	0.559137724	1.18E-45	postive
TUBE1	AL662844.4	0.571938858	3.80E-48	postive
ALOX12	AL662844.4	0.64635715	4.48E-65	postive
YY1AP1	AL662844.4	0.449310494	3.86E-28	postive
TAZ	AL662844.4	0.48169678	1.16E-32	postive
ASNS	LINC02577	0.409356436	3.42E-23	postive
FANCD2	SAP30L-AS1	0.436099594	1.98E-26	postive
HELLS	SAP30L-AS1	0.473461843	1.83E-31	postive
ALOX12	SAP30L-AS1	0.451187743	2.17E-28	postive
TFAP2C	SAP30L-AS1	0.503063398	6.43E-36	postive

HBA1	SAP30L-AS1	0.450273263	2.88E-28	postive
DUOX1	SAP30L-AS1	0.451120806	2.22E-28	postive
TUBE1	AC113361.1	0.428603321	1.71E-25	postive
TFAP2C	HCG14	0.552180907	2.41E-44	postive
HBA1	HCG14	0.499719377	2.15E-35	postive
TFAP2C	CATIP-AS2	0.423855932	6.52E-25	postive
DUOX1	CATIP-AS2	0.610130378	2.84E-56	postive
HELLS	AL157392.2	0.453018402	1.24E-28	postive
TUBE1	AL157392.2	0.472891818	2.20E-31	postive
ALOX12	AL157392.2	0.497449155	4.86E-35	postive
GABPB1	AL157392.2	0.471922226	3.03E-31	postive
LINC00472	AL157392.2	0.623081453	2.75E-59	postive
ATM	AL157392.2	0.65859688	2.51E-68	postive
TUBE1	AC051619.4	0.433429635	4.29E-26	postive
ALOX12	AC051619.4	0.420771559	1.54E-24	postive
ZNF419	AL445222.1	0.422618998	9.21E-25	postive
LINC00472	AL445222.1	0.670251352	1.44E-71	postive
ATM	AC104819.3	0.492994541	2.35E-34	postive
KLHL24	AC025271.4	0.551128195	3.78E-44	postive
LINC00472	AC025271.4	0.807288217	4.36E-125	postive
LPIN1	AC025271.4	0.478159204	3.83E-32	postive
HELLS	LINC01359	0.547916836	1.48E-43	postive
ZNF419	LINC01359	0.419280696	2.32E-24	postive
TUBE1	LINC01359	0.512267164	2.15E-37	postive
ALOX12	LINC01359	0.539008453	6.01E-42	postive
GABPB1	LINC01359	0.57249964	2.94E-48	postive
MAPK8	LINC01359	0.457613301	2.97E-29	postive
LINC00472	LINC01359	0.728436322	3.01E-90	postive
ATM	LINC01359	0.718812732	7.83E-87	postive
FBXW7	LINC01359	0.507329065	1.35E-36	postive
HELLS	AP000766.1	0.405285748	1.00E-22	postive
KLHL24	AP000766.1	0.566953759	3.66E-47	postive
TUBE1	AP000766.1	0.505087605	3.07E-36	postive
MAP3K5	AP000766.1	0.415772632	6.07E-24	postive
IREB2	AP000766.1	0.576854365	3.92E-49	postive
GABPB1	AP000766.1	0.59499948	6.37E-53	postive
EMC2	AP000766.1	0.434984947	2.73E-26	postive
PIK3CA	AP000766.1	0.562250035	2.99E-46	postive
KRAS	AP000766.1	0.542081041	1.70E-42	postive
ZEB1	AP000766.1	0.408613841	4.16E-23	postive
MAPK8	AP000766.1	0.603805469	7.52E-55	postive
LINC00472	AP000766.1	0.78718976	8.20E-115	postive
PRKAA2	AP000766.1	0.521410854	6.62E-39	postive
PRKAA1	AP000766.1	0.587460421	2.56E-51	postive
ATM	AP000766.1	0.670753407	1.04E-71	postive
SIRT1	AP000766.1	0.405462022	9.58E-23	postive
FBXW7	AP000766.1	0.417521264	3.76E-24	postive
HELLS	RAB33B-AS1	0.521002893	7.75E-39	postive
ZNF419	RAB33B-AS1	0.481072568	1.44E-32	postive
KLHL24	RAB33B-AS1	0.502793857	7.09E-36	postive
TUBE1	RAB33B-AS1	0.576817258	3.99E-49	postive
ALOX12	RAB33B-AS1	0.535645125	2.37E-41	postive
IREB2	RAB33B-AS1	0.475428808	9.53E-32	postive
GABPB1	RAB33B-AS1	0.61911025	2.39E-58	postive
PIK3CA	RAB33B-AS1	0.42245116	9.65E-25	postive
MAPK8	RAB33B-AS1	0.521166586	7.27E-39	postive
LINC00472	RAB33B-AS1	0.755622088	9.60E-101	postive
PRKAA2	RAB33B-AS1	0.426075698	3.50E-25	postive

ATM	RAB33B-AS1	0.723190611	2.28E-88	postive
FBXW7	RAB33B-AS1	0.497028682	5.64E-35	postive
HELLS	EZR-AS1	0.497198038	5.31E-35	postive
TUBE1	EZR-AS1	0.404648759	1.19E-22	postive
GABPB1	EZR-AS1	0.462599783	6.16E-30	postive
LINC00472	EZR-AS1	0.523100612	3.44E-39	postive
ATM	EZR-AS1	0.66948119	2.38E-71	postive
FBXW7	EZR-AS1	0.413960259	9.93E-24	postive
HELLS	AC110792.3	0.522018011	5.23E-39	postive
ZNF419	AC110792.3	0.411286006	2.04E-23	postive
TUBE1	AC110792.3	0.53082036	1.65E-40	postive
GABPB1	AC110792.3	0.627996346	1.81E-60	postive
MAPK8	AC110792.3	0.446560842	8.88E-28	postive
LINC00472	AC110792.3	0.669520091	2.32E-71	postive
ATM	AC110792.3	0.689427226	3.10E-77	postive
FBXW7	AC110792.3	0.544805172	5.46E-43	postive
TUBE1	MROCKI	0.639965032	1.95E-63	postive
ALOX12	MROCKI	0.480998661	1.47E-32	postive
TAZ	MROCKI	0.490572209	5.50E-34	postive
ZNF419	AP003419.3	0.412321096	1.54E-23	postive
ALOX12	AP003419.3	0.4527084	1.36E-28	postive
HBA1	AP003419.3	0.482852679	7.86E-33	postive
HRAS	AP003419.3	0.411736352	1.81E-23	postive
PHKG2	AP003419.3	0.450578208	2.62E-28	postive
EGLN2	AP003419.3	0.45459056	7.62E-29	postive
TAZ	AP003419.3	0.521300762	6.90E-39	postive
HELLS	AL513477.2	0.414955414	7.58E-24	postive
ALOX12	AL513477.2	0.464831494	3.02E-30	postive
TFAP2C	AL513477.2	0.437228982	1.42E-26	postive
ZNF419	AC005498.3	0.474548274	1.28E-31	postive
ULK1	DNAH10OS	0.40300462	1.82E-22	postive
ACVR1B	DNAH10OS	0.422518376	9.47E-25	postive
HBA1	AP001094.1	0.509394664	6.27E-37	postive
HRAS	AP001094.1	0.514428665	9.53E-38	postive
EGLN2	AP001094.1	0.51808417	2.38E-38	postive
HELLS	AC007663.3	0.441109433	4.53E-27	postive
ZNF419	AC007663.3	0.474574933	1.26E-31	postive
ALOX12	AC007663.3	0.416643348	4.79E-24	postive
GABPB1	AC007663.3	0.425051746	4.66E-25	postive
ATM	AC007663.3	0.407205537	6.05E-23	postive
TAZ	AC007663.3	0.465290029	2.61E-30	postive
TFAP2C	LINC02453	0.593484269	1.35E-52	postive
HBA1	LINC02453	0.516928845	3.69E-38	postive
HELLS	AL451064.1	0.559219692	1.14E-45	postive
TUBE1	AL451064.1	0.470930863	4.19E-31	postive
ALOX12	AL451064.1	0.474665708	1.23E-31	postive
GABPB1	AL451064.1	0.504707154	3.53E-36	postive
LINC00472	AL451064.1	0.608529157	6.56E-56	postive
ATM	AL451064.1	0.630446297	4.59E-61	postive
FBXW7	AL451064.1	0.432369114	5.82E-26	postive
TUBE1	UGDH-AS1	0.422731486	8.92E-25	postive
ALOX12	UGDH-AS1	0.498255298	3.64E-35	postive
IREB2	UGDH-AS1	0.582198544	3.18E-50	postive
PIK3CA	UGDH-AS1	0.407582721	5.47E-23	postive
ULK2	UGDH-AS1	0.425664753	3.92E-25	postive
MAPK8	UGDH-AS1	0.586042251	5.06E-51	postive
LINC00472	UGDH-AS1	0.4056179	9.20E-23	postive
PRKAA2	UGDH-AS1	0.485298661	3.41E-33	postive

PRKAA1	UGDH-AS1	0.405344367	9.88E-23	postive
ATM	UGDH-AS1	0.624364825	1.36E-59	postive
SIRT1	UGDH-AS1	0.47005931	5.57E-31	postive
GDF15	AC015845.2	0.450792337	2.45E-28	postive
FANCD2	AC007938.3	0.446024172	1.04E-27	postive
HELLS	AC007938.3	0.516094097	5.07E-38	postive
ZNF419	AC007938.3	0.478655219	3.25E-32	postive
ALOX12	AC007938.3	0.407731979	5.26E-23	postive
GABPB1	AC007938.3	0.413300845	1.19E-23	postive
LINC00472	AC007938.3	0.414492223	8.59E-24	postive
FBXW7	AC007938.3	0.411548612	1.90E-23	postive
ZNF419	AC008969.1	0.676939535	1.70E-73	postive
VEGFA	AC008969.1	0.520232981	1.04E-38	postive
TUBE1	AC008969.1	0.608125424	8.10E-56	postive
SETD1B	AC008969.1	0.504291894	4.11E-36	postive
ALOX12	AC008969.1	0.665787225	2.61E-70	postive
GABPB1	AC008969.1	0.519793671	1.23E-38	postive
MAPK8	AC008969.1	0.417596689	3.69E-24	postive
LINC00472	AC008969.1	0.40278164	1.93E-22	postive
ATM	AC008969.1	0.494723466	1.28E-34	postive
TAZ	AC008969.1	0.413454132	1.14E-23	postive
FBXW7	AC008969.1	0.413370216	1.16E-23	postive
FANCD2	AC024267.3	0.415922302	5.83E-24	postive
HELLS	AC024267.3	0.414954496	7.58E-24	postive
ALOX12	AC024267.3	0.544533268	6.12E-43	postive
TFAP2C	AC024267.3	0.447085261	7.58E-28	postive
HBA1	AC024267.3	0.412202347	1.60E-23	postive
DUOX1	AC024267.3	0.449678291	3.45E-28	postive
HELLS	RHOQ-AS1	0.508033358	1.04E-36	postive
ZNF419	RHOQ-AS1	0.400136162	3.84E-22	postive
KLHL24	RHOQ-AS1	0.403862104	1.46E-22	postive
TUBE1	RHOQ-AS1	0.480925498	1.51E-32	postive
ALOX12	RHOQ-AS1	0.469295165	7.15E-31	postive
GABPB1	RHOQ-AS1	0.572570889	2.85E-48	postive
MAPK8	RHOQ-AS1	0.402295902	2.19E-22	postive
LINC00472	RHOQ-AS1	0.806606157	1.02E-124	postive
ATM	RHOQ-AS1	0.679044038	4.11E-74	postive
FBXW7	RHOQ-AS1	0.495351077	1.02E-34	postive
HELLS	ALG13-AS1	0.562912713	2.23E-46	postive
ZNF419	ALG13-AS1	0.431482548	7.51E-26	postive
TUBE1	ALG13-AS1	0.523453619	3.00E-39	postive
ALOX12	ALG13-AS1	0.545901862	3.45E-43	postive
GABPB1	ALG13-AS1	0.586520122	4.02E-51	postive
MAPK8	ALG13-AS1	0.435978835	2.05E-26	postive
LINC00472	ALG13-AS1	0.702062713	3.23E-81	postive
ATM	ALG13-AS1	0.744438571	2.89E-96	postive
FBXW7	ALG13-AS1	0.513277009	1.47E-37	postive
KLHL24	AL031716.1	0.505725106	2.43E-36	postive
TUBE1	AL031716.1	0.420891761	1.49E-24	postive
GABPB1	AL031716.1	0.525415439	1.39E-39	postive
PIK3CA	AL031716.1	0.407734059	5.26E-23	postive
LINC00472	AL031716.1	0.906165307	7.62E-203	postive
ATM	AL031716.1	0.56365693	1.60E-46	postive
HELLS	AC009318.4	0.401719032	2.55E-22	postive
TUBE1	AC009318.4	0.408989169	3.77E-23	postive
ALOX12	AC009318.4	0.447365385	6.96E-28	postive
PHKG2	AC009318.4	0.410588672	2.46E-23	postive
TAZ	AC009318.4	0.436907331	1.56E-26	postive

KLHL24	TEX41	0.472790072	2.28E-31	postive
LINC00472	TEX41	0.71019105	6.82E-84	postive
ATM	TEX41	0.438645187	9.38E-27	postive
FANCD2	AC000068.1	0.431956845	6.55E-26	postive
TFAP2C	AC000068.1	0.59429295	9.03E-53	postive
HBA1	AC000068.1	0.496612359	6.55E-35	postive
DUOX1	AC000068.1	0.406594389	7.11E-23	postive
ISCU	SCAMP1-AS1	0.668344501	5.00E-71	postive
TMBIM4	SCAMP1-AS1	0.467761251	1.18E-30	postive
CHMP5	SCAMP1-AS1	0.52411075	2.32E-39	postive
DDIT3	SCAMP1-AS1	0.430857078	8.98E-26	postive
HERPUD1	SCAMP1-AS1	0.40255207	2.05E-22	postive
GOT1	SCAMP1-AS1	0.522024666	5.22E-39	postive
ATG4D	SCAMP1-AS1	0.514234716	1.03E-37	postive
GABARAPL2	SCAMP1-AS1	0.584551469	1.04E-50	postive
GABARAPL1	SCAMP1-AS1	0.471192352	3.85E-31	postive
ZNF419	LINC00685	0.438977847	8.50E-27	postive
DRD4	LINC00685	0.411360326	2.00E-23	postive
ALOX12	LINC00685	0.40910993	3.65E-23	postive
HBA1	LINC00685	0.428188688	1.92E-25	postive
PHKG2	LINC00685	0.462781677	5.82E-30	postive
EGLN2	LINC00685	0.490698521	5.26E-34	postive
TAZ	LINC00685	0.520044413	1.12E-38	postive
HBA1	AC021188.1	0.427258484	2.50E-25	postive
PHKG2	AC000068.2	0.456490135	4.22E-29	postive
EGLN2	AC000068.2	0.55468072	8.22E-45	postive
TAZ	AC000068.2	0.410354575	2.62E-23	postive
MTOR	MIR600HG	0.423430192	7.34E-25	postive
ZNF419	MIR600HG	0.478887525	3.00E-32	postive
KLHL24	MIR600HG	0.486468284	2.28E-33	postive
GABARAPL1	MIR600HG	0.480473699	1.76E-32	postive
LINC00472	MIR600HG	0.569839779	9.91E-48	postive
BAP1	MIR600HG	0.414389976	8.84E-24	postive
LPIN1	MIR600HG	0.584369427	1.13E-50	postive
ZNF419	AL158063.1	0.467604207	1.24E-30	postive
TUBE1	AL158063.1	0.473541792	1.78E-31	postive
ALOX12	AL158063.1	0.520810253	8.34E-39	postive
PHKG2	AL158063.1	0.469857177	5.95E-31	postive
TAZ	AL158063.1	0.579568659	1.10E-49	postive
PHKG2	AC026367.1	0.44284451	2.71E-27	postive
GPX4	ENTPD3-AS1	0.421301682	1.33E-24	postive
HBA1	ENTPD3-AS1	0.620140732	1.37E-58	postive
HRAS	ENTPD3-AS1	0.509838534	5.32E-37	postive
MAP1LC3A	ENTPD3-AS1	0.493154721	2.23E-34	postive
EGLN2	ENTPD3-AS1	0.641097176	1.01E-63	postive
TUBE1	HCG17	0.436052745	2.00E-26	postive
ATM	HCG17	0.407184251	6.08E-23	postive
ZNF419	AC011330.2	0.40117773	2.93E-22	postive
LINC00472	AC011330.2	0.563106159	2.05E-46	postive
LPIN1	AC011330.2	0.471661337	3.30E-31	postive
HELLS	AC009269.5	0.519407322	1.43E-38	postive
TUBE1	AC009269.5	0.450198259	2.94E-28	postive
ALOX12	AC009269.5	0.426668623	2.96E-25	postive
GABPB1	AC009269.5	0.576600922	4.41E-49	postive
ATG7	AC009269.5	0.400133493	3.84E-22	postive
LINC00472	AC009269.5	0.736181848	4.18E-93	postive
ATM	AC009269.5	0.686981141	1.73E-76	postive
FBXW7	AC009269.5	0.498349959	3.52E-35	postive

HELLS	AC004918.3	0.460411838	1.23E-29	postive
MTOR	AC004918.3	0.41101581	2.19E-23	postive
ZNF419	AC004918.3	0.454182583	8.65E-29	postive
ZFP69B	AC004918.3	0.453602733	1.03E-28	postive
TUBE1	AC004918.3	0.517412874	3.07E-38	postive
MAP3K5	AC004918.3	0.400986958	3.08E-22	postive
ALOX12	AC004918.3	0.555675296	5.35E-45	postive
IREB2	AC004918.3	0.498904208	2.89E-35	postive
SP1	AC004918.3	0.403531558	1.59E-22	postive
GABPB1	AC004918.3	0.555361819	6.12E-45	postive
PIK3CA	AC004918.3	0.4222917	1.01E-24	postive
ZEB1	AC004918.3	0.492309395	3.00E-34	postive
MAPK8	AC004918.3	0.552060076	2.54E-44	postive
LINC00472	AC004918.3	0.531956631	1.05E-40	postive
TLR4	AC004918.3	0.406536974	7.22E-23	postive
ATM	AC004918.3	0.790971651	1.17E-116	postive
FBXW7	AC004918.3	0.550033865	6.02E-44	postive
HELLS	AC009716.1	0.505184279	2.96E-36	postive
KLHL24	AC009716.1	0.457074897	3.52E-29	postive
TUBE1	AC009716.1	0.433784835	3.87E-26	postive
ALOX12	AC009716.1	0.450954778	2.34E-28	postive
IREB2	AC009716.1	0.444989239	1.42E-27	postive
GABPB1	AC009716.1	0.556666099	3.48E-45	postive
PIK3CA	AC009716.1	0.434596215	3.06E-26	postive
ATG7	AC009716.1	0.427293287	2.48E-25	postive
MAPK8	AC009716.1	0.420559144	1.63E-24	postive
LINC00472	AC009716.1	0.781137285	6.19E-112	postive
ATM	AC009716.1	0.694593925	7.73E-79	postive
FBXW7	AC009716.1	0.46053258	1.19E-29	postive
SLC2A6	AL359091.4	0.421846562	1.14E-24	postive
HRAS	AL359091.4	0.418786431	2.66E-24	postive
NCOA4	AL359091.4	-0.410510929	2.51E-23	negative
SLC2A6	RFX5-AS1	0.471659575	3.30E-31	postive
HELLS	AC005034.2	0.440219326	5.90E-27	postive
ZNF419	AC005034.2	0.411634565	1.86E-23	postive
KLHL24	AC005034.2	0.559530489	9.94E-46	postive
TUBE1	AC005034.2	0.460431682	1.23E-29	postive
IREB2	AC005034.2	0.420496122	1.66E-24	postive
GABPB1	AC005034.2	0.564383325	1.16E-46	postive
PIK3CA	AC005034.2	0.458970858	1.94E-29	postive
MAPK8	AC005034.2	0.426421586	3.17E-25	postive
LINC00472	AC005034.2	0.887933539	2.98E-183	postive
ATM	AC005034.2	0.661155855	5.02E-69	postive
FBXW7	AC005034.2	0.494123839	1.58E-34	postive
CHAC1	PIK3IP1-DT	0.534408721	3.90E-41	postive
ZNF419	PIK3IP1-DT	0.409497729	3.29E-23	postive
MAP1LC3A	PIK3IP1-DT	0.403296106	1.69E-22	postive
LINC00472	PIK3IP1-DT	0.451797476	1.80E-28	postive
LPIN1	PIK3IP1-DT	0.483233353	6.91E-33	postive
ISCU	AL133370.1	0.501600349	1.09E-35	postive
DDIT3	AL133370.1	0.470170165	5.38E-31	postive
ATG4D	AL133370.1	0.404097908	1.37E-22	postive
GABARAPL1	AL133370.1	0.583298833	1.88E-50	postive
LPIN1	AL133370.1	0.472274038	2.70E-31	postive
TUBE1	AC007546.1	0.462260745	6.86E-30	postive
ALOX12	AC007546.1	0.417857588	3.43E-24	postive
GABPB1	AC007546.1	0.408145068	4.72E-23	postive
LINC00472	AC007546.1	0.615019347	2.15E-57	postive

ZNF419	C9orf106	0.476758077	6.12E-32	postive
TUBE1	C9orf106	0.423189826	7.85E-25	postive
ALOX12	C9orf106	0.556579693	3.61E-45	postive
GABPB1	C9orf106	0.424657463	5.21E-25	postive
ATM	C9orf106	0.509696067	5.61E-37	postive
MT3	AC114803.1	0.489360077	8.39E-34	postive
HILPDA	AC114803.1	0.415719826	6.16E-24	postive
PTGS2	AC010735.2	0.498751874	3.05E-35	postive
ATF4	H1-10-AS1	0.434018324	3.62E-26	postive
BRD4	H1-10-AS1	0.485529511	3.15E-33	postive
LAMP2	H1-10-AS1	-0.403424191	1.63E-22	negative
ZNF419	H1-10-AS1	0.48456496	4.38E-33	postive
VEGFA	H1-10-AS1	0.419837903	1.99E-24	postive
DRD4	H1-10-AS1	0.412424751	1.50E-23	postive
ALOX12	H1-10-AS1	0.485478922	3.21E-33	postive
HIC1	H1-10-AS1	0.433129057	4.68E-26	postive
NCOA4	H1-10-AS1	-0.439749017	6.78E-27	negative
PHKG2	H1-10-AS1	0.486555102	2.21E-33	postive
TAZ	H1-10-AS1	0.693044475	2.36E-78	postive
LPIN1	AL391121.1	0.438978004	8.50E-27	postive
ZNF419	AC079174.2	0.475832288	8.33E-32	postive
NRAS	AC079174.2	-0.437357065	1.37E-26	negative
NCOA4	AC079174.2	-0.416654246	4.77E-24	negative
PHKG2	AC079174.2	0.636951262	1.12E-62	postive
ULK1	AC079174.2	0.410064283	2.83E-23	postive
TAZ	AC079174.2	0.611206159	1.62E-56	postive
ZNF419	NFYC-AS1	0.563743521	1.54E-46	postive
VEGFA	NFYC-AS1	0.402476994	2.09E-22	postive
TUBE1	NFYC-AS1	0.65989716	1.11E-68	postive
SETD1B	NFYC-AS1	0.513900325	1.16E-37	postive
ALOX12	NFYC-AS1	0.73688145	2.28E-93	postive
GABPB1	NFYC-AS1	0.466099885	2.01E-30	postive
LINC00472	NFYC-AS1	0.41965119	2.10E-24	postive
ATM	NFYC-AS1	0.56643428	4.62E-47	postive
TAZ	NFYC-AS1	0.486575541	2.20E-33	postive
FANCD2	AC004803.1	0.612737202	7.22E-57	postive
HELLS	AC004803.1	0.524083732	2.34E-39	postive
ANGPTL7	AC004803.1	0.542909971	1.20E-42	postive
ZNF419	AC004803.1	0.407951261	4.97E-23	postive
EIF2S1	AC004803.1	0.440646956	5.20E-27	postive
AURKA	AC004803.1	0.441811729	3.68E-27	postive
TUBE1	AL359915.1	0.440800031	4.97E-27	postive
ALOX12	AL359915.1	0.403228413	1.72E-22	postive
MAPK8	AL359915.1	0.421159535	1.38E-24	postive
PRKAA2	AL359915.1	0.441453003	4.09E-27	postive
ATM	AL359915.1	0.569388558	1.22E-47	postive
ALOX12	TRAPPC12-A	0.467730945	1.19E-30	postive
HELLS	AC011825.2	0.543082375	1.12E-42	postive
KLHL24	AC011825.2	0.40758207	5.48E-23	postive
TUBE1	AC011825.2	0.49214945	3.17E-34	postive
ALOX12	AC011825.2	0.475177017	1.04E-31	postive
IREB2	AC011825.2	0.425472204	4.14E-25	postive
GABPB1	AC011825.2	0.595269885	5.57E-53	postive
PIK3CA	AC011825.2	0.401640218	2.60E-22	postive
MAPK8	AC011825.2	0.473747614	1.66E-31	postive
LINC00472	AC011825.2	0.793136816	9.84E-118	postive
ATM	AC011825.2	0.715921937	7.79E-86	postive
FBXW7	AC011825.2	0.51019636	4.66E-37	postive

HELLS	LINC01376	0.509710927	5.58E-37	postive
ALOX12	LINC01376	0.515900636	5.46E-38	postive
TFAP2C	LINC01376	0.417657613	3.63E-24	postive
GABPB1	LINC01376	0.413263149	1.20E-23	postive
DUOX1	LINC01376	0.451903409	1.75E-28	postive
ATM	LINC01376	0.487343575	1.69E-33	postive
TFAP2C	AL596325.2	0.431985369	6.50E-26	postive
LINC00472	AL596325.2	0.616814303	8.24E-58	postive
LPIN1	AL596325.2	0.443705206	2.09E-27	postive
HELLS	AC145285.2	0.403857741	1.46E-22	postive
TUBE1	AC145285.2	0.449143821	4.06E-28	postive
ALOX12	AC145285.2	0.534370317	3.96E-41	postive
PHKG2	AC145285.2	0.486782659	2.05E-33	postive
TAZ	AC145285.2	0.535672498	2.34E-41	postive
BAP1	LINC01123	0.412292843	1.56E-23	postive
HELLS	AC053513.2	0.530597647	1.80E-40	postive
ZNF419	AC053513.2	0.479783372	2.22E-32	postive
KLHL24	AC053513.2	0.444677589	1.56E-27	postive
TUBE1	AC053513.2	0.582388647	2.90E-50	postive
ALOX12	AC053513.2	0.573453419	1.90E-48	postive
IREB2	AC053513.2	0.485167347	3.57E-33	postive
GABPB1	AC053513.2	0.607975881	8.75E-56	postive
PIK3CA	AC053513.2	0.442415056	3.08E-27	postive
MAPK8	AC053513.2	0.519410408	1.43E-38	postive
LINC00472	AC053513.2	0.703959299	7.82E-82	postive
ATM	AC053513.2	0.794603528	1.81E-118	postive
FBXW7	AC053513.2	0.553050864	1.66E-44	postive
HSPB1	AC008610.1	0.419545583	2.16E-24	postive
DRD4	AC008610.1	0.414038459	9.72E-24	postive
NRAS	AC008610.1	-0.429448383	1.34E-25	negative
NCOA4	AC008610.1	-0.481653249	1.18E-32	negative
PHKG2	AC008610.1	0.656280129	1.06E-67	postive
MAPK1	AC008610.1	-0.441669639	3.84E-27	negative
TAZ	AC008610.1	0.693907168	1.27E-78	postive
HELLS	AC078906.1	0.470398991	4.99E-31	postive
TUBE1	AC078906.1	0.505167771	2.98E-36	postive
ALOX12	AC078906.1	0.521882897	5.51E-39	postive
ATM	AC078906.1	0.492487333	2.81E-34	postive
TAZ	AC078906.1	0.480242487	1.90E-32	postive
FBXW7	AC078906.1	0.406325589	7.63E-23	postive
VEGFA	SLC25A21-A:	0.42353119	7.14E-25	postive
HELLS	AL121890.5	0.446251086	9.75E-28	postive
ZNF419	AL121890.5	0.418818205	2.64E-24	postive
TUBE1	AL121890.5	0.423595368	7.01E-25	postive
ALOX12	AL121890.5	0.464546861	3.31E-30	postive
TFAP2C	AL121890.5	0.42366387	6.88E-25	postive
HELLS	AC084824.3	0.42142751	1.28E-24	postive
ZNF419	AC084824.3	0.46507164	2.80E-30	postive
KLHL24	AC084824.3	0.4057711	8.83E-23	postive
TUBE1	AC084824.3	0.545262123	4.51E-43	postive
ALOX12	AC084824.3	0.516295789	4.70E-38	postive
GABPB1	AC084824.3	0.544368806	6.55E-43	postive
MAPK8	AC084824.3	0.404113848	1.36E-22	postive
LINC00472	AC084824.3	0.804791208	9.56E-124	postive
ATM	AC084824.3	0.610176044	2.78E-56	postive
FBXW7	AC084824.3	0.450741468	2.49E-28	postive
HELLS	AC097641.2	0.54352965	9.29E-43	postive
ZNF419	AC097641.2	0.530900991	1.59E-40	postive

TUBE1	AC097641.2	0.566746241	4.02E-47 postive
ALOX12	AC097641.2	0.583324964	1.86E-50 postive
GABPB1	AC097641.2	0.485283356	3.43E-33 postive
PHKG2	AC097641.2	0.409959374	2.91E-23 postive
LINC00472	AC097641.2	0.413711084	1.06E-23 postive
ATM	AC097641.2	0.577541984	2.85E-49 postive
TAZ	AC097641.2	0.472289468	2.69E-31 postive
FBXW7	AC097641.2	0.535072024	2.99E-41 postive
GCLC	U91328.1	0.473110188	2.05E-31 postive
IREB2	U91328.1	0.433032574	4.81E-26 postive
BID	U91328.1	-0.424786138	5.02E-25 negative
MAPK8	U91328.1	0.491373781	4.16E-34 postive
JDP2	AC083967.1	0.518155329	2.31E-38 postive
RGS4	AC083967.1	0.471248173	3.78E-31 postive
EIF2S1	AC083967.1	0.462843427	5.70E-30 postive
NOX1	AL031775.1	0.530106454	2.19E-40 postive
TFAP2C	AC100812.1	0.404733765	1.16E-22 postive
BRD4	SH3BP5-AS1	0.432438036	5.71E-26 postive
ZNF419	SH3BP5-AS1	0.584488125	1.07E-50 postive
VEGFA	SH3BP5-AS1	0.499517483	2.32E-35 postive
TUBE1	SH3BP5-AS1	0.638651326	4.20E-63 postive
SETD1B	SH3BP5-AS1	0.494723388	1.28E-34 postive
ALOX12	SH3BP5-AS1	0.66277153	1.80E-69 postive
GABPB1	SH3BP5-AS1	0.499520415	2.31E-35 postive
PHKG2	SH3BP5-AS1	0.410396485	2.59E-23 postive
ATM	SH3BP5-AS1	0.497595845	4.61E-35 postive
TAZ	SH3BP5-AS1	0.609139125	4.78E-56 postive
FBXW7	SH3BP5-AS1	0.53741228	1.15E-41 postive
HELLS	AC093423.2	0.542778444	1.27E-42 postive
TUBE1	AC093423.2	0.433560145	4.13E-26 postive
ALOX12	AC093423.2	0.434713157	2.96E-26 postive
GABPB1	AC093423.2	0.558797956	1.37E-45 postive
ATG7	AC093423.2	0.475486808	9.35E-32 postive
MAPK8	AC093423.2	0.414733543	8.05E-24 postive
LINC00472	AC093423.2	0.611111754	1.70E-56 postive
ATM	AC093423.2	0.731934629	1.58E-91 postive
FBXW7	AC093423.2	0.465335231	2.57E-30 postive
FANCD2	TMED2-DT	0.486067747	2.62E-33 postive
HELLS	TMED2-DT	0.453378808	1.11E-28 postive
ZNF419	TMED2-DT	0.507812907	1.13E-36 postive
TUBE1	TMED2-DT	0.453923612	9.37E-29 postive
DRD4	TMED2-DT	0.414807548	7.89E-24 postive
ALOX12	TMED2-DT	0.675050754	6.03E-73 postive
PHKG2	TMED2-DT	0.435751351	2.19E-26 postive
TAZ	TMED2-DT	0.502960241	6.67E-36 postive
HELLS	SNHG22	0.478605667	3.30E-32 postive
GABPB1	SNHG22	0.430535752	9.85E-26 postive
LINC00472	SNHG22	0.427346592	2.44E-25 postive
ATM	SNHG22	0.662992444	1.56E-69 postive
FBXW7	SNHG22	0.522730203	3.97E-39 postive
TUBE1	SRP14-AS1	0.509624801	5.76E-37 postive
OXSR1	AL133355.1	0.405911694	8.51E-23 postive
SP1	AL133355.1	0.504202526	4.24E-36 postive
ZEB1	AL133355.1	0.423862549	6.51E-25 postive
MAPK8	AL133355.1	0.457679595	2.91E-29 postive
EPAS1	AL133355.1	0.461048666	1.01E-29 postive
SIRT1	AL133355.1	0.44707731	7.60E-28 postive
WIPI2	LINC00865	0.407218776	6.03E-23 postive

LPIN1	LINC00865	0.512176701	2.22E-37	postive
NNMT	LINC01426	0.4284154	1.80E-25	postive
TAZ	AC010173.1	0.435825273	2.14E-26	postive
TFAP2C	FAM222A-AS	0.528320352	4.44E-40	postive
HBA1	FAM222A-AS	0.472501461	2.51E-31	postive
RB1	TRAM2-AS1	0.495969625	8.23E-35	postive
GCLC	TRAM2-AS1	0.408025078	4.87E-23	postive
CHMP5	TRAM2-AS1	0.486577484	2.20E-33	postive
HSD17B11	TRAM2-AS1	0.477240144	5.21E-32	postive
MAPK14	TRAM2-AS1	0.437457978	1.33E-26	postive
HMGB1	TRAM2-AS1	0.402379891	2.15E-22	postive
NRAS	TRAM2-AS1	0.46417894	3.72E-30	postive
ATG5	TRAM2-AS1	0.461217161	9.56E-30	postive
NCOA4	TRAM2-AS1	0.489399783	8.28E-34	postive
PHKG2	TRAM2-AS1	-0.419030899	2.49E-24	negative
SNX4	TRAM2-AS1	0.404878412	1.12E-22	postive
MAPK1	TRAM2-AS1	0.457211115	3.37E-29	postive
MAPK8	TRAM2-AS1	0.442571872	2.94E-27	postive
PRKAA2	TRAM2-AS1	0.465243043	2.65E-30	postive
SIRT1	TRAM2-AS1	0.54854898	1.13E-43	postive
HBA1	RTCA-AS1	0.50282006	7.02E-36	postive
HRAS	RTCA-AS1	0.533772727	5.04E-41	postive
EGLN2	RTCA-AS1	0.737362736	1.50E-93	postive
ISCU	NINJ2-AS1	0.487471028	1.61E-33	postive
GABARAPL1	NINJ2-AS1	0.486163753	2.53E-33	postive
WIPI2	NINJ2-AS1	0.405329992	9.92E-23	postive
LPIN1	NINJ2-AS1	0.441481183	4.06E-27	postive
NFS1	PCAT19	-0.428763894	1.63E-25	negative
SLC1A4	PCAT19	0.461897791	7.70E-30	postive
BLOC1S5-TXNDC5	PCAT19	0.442944125	2.63E-27	postive
IL33	PCAT19	0.44653406	8.95E-28	postive
HIC1	PCAT19	0.438523725	9.72E-27	postive
MAPK3	PCAT19	0.410565975	2.47E-23	postive
ZEB1	PCAT19	0.492536444	2.77E-34	postive
EPAS1	PCAT19	0.497445759	4.86E-35	postive
EGLN2	PCAT19	0.446595532	8.79E-28	postive
ZNF419	AC093797.1	0.448770073	4.55E-28	postive
VEGFA	AC093797.1	0.417447763	3.84E-24	postive
TUBE1	AC093797.1	0.527918849	5.21E-40	postive
ALOX12	AC093797.1	0.60131711	2.67E-54	postive
GABPB1	AC093797.1	0.473007828	2.12E-31	postive
MAPK8	AC093797.1	0.45166321	1.88E-28	postive
LINC00472	AC093797.1	0.53399613	4.61E-41	postive
ATM	AC093797.1	0.631338071	2.77E-61	postive
FBXW7	AC093797.1	0.426062993	3.51E-25	postive
ALOX12	AC120114.1	0.436490774	1.76E-26	postive
IREB2	AC120114.1	0.423937558	6.37E-25	postive
SP1	AC120114.1	0.526883266	7.83E-40	postive
ULK2	AC120114.1	0.439111464	8.18E-27	postive
ATM	AC120114.1	0.417363276	3.93E-24	postive
SIRT1	AC120114.1	0.401349707	2.80E-22	postive
SLC1A4	AL355803.1	0.480878506	1.54E-32	postive
VEGFA	AL355803.1	0.492822131	2.50E-34	postive
MAPK3	AL355803.1	0.457272017	3.31E-29	postive
ZEB1	AL355803.1	0.601169775	2.88E-54	postive
EPAS1	AL355803.1	0.614920834	2.27E-57	postive
TFAP2C	AC006111.2	0.589431103	9.83E-52	postive
HBA1	AC006111.2	0.488838797	1.01E-33	postive

DUOX1	AC006111.2	0.401536017	2.67E-22	postive
FANCD2	AC004816.1	0.436983063	1.53E-26	postive
ASNS	AC004816.1	0.418323395	3.02E-24	postive
JDP2	AC004816.1	0.512968446	1.65E-37	postive
PSAT1	AC004816.1	0.437730028	1.23E-26	postive
RGS4	AC004816.1	0.43305435	4.78E-26	postive
EIF2S1	AC004816.1	0.485895267	2.78E-33	postive
AURKA	AC004816.1	0.428773833	1.63E-25	postive
FANCD2	KCTD21-AS1	0.431256655	8.01E-26	postive
HBA1	KCTD21-AS1	0.427765507	2.17E-25	postive
TFAP2C	LINC01593	0.415867288	5.91E-24	postive
FH	GAS6-DT	0.400247612	3.73E-22	postive
ISCU	GAS6-DT	0.652412968	1.15E-66	postive
ACSL3	GAS6-DT	0.467345374	1.35E-30	postive
KLHL24	GAS6-DT	0.432630541	5.40E-26	postive
SLC2A8	GAS6-DT	0.420980932	1.45E-24	postive
SLC2A12	GAS6-DT	0.43864887	9.37E-27	postive
GOT1	GAS6-DT	0.440910257	4.81E-27	postive
ATG4D	GAS6-DT	0.558698677	1.43E-45	postive
MAP1LC3A	GAS6-DT	0.402778354	1.93E-22	postive
GABARAPL2	GAS6-DT	0.485639492	3.03E-33	postive
GABARAPL1	GAS6-DT	0.676807227	1.86E-73	postive
BAP1	GAS6-DT	0.477762378	4.38E-32	postive
LPIN1	GAS6-DT	0.614462678	2.89E-57	postive
HELLS	DTX2P1-UPK	0.543279616	1.03E-42	postive
ZNF419	DTX2P1-UPK	0.422155269	1.05E-24	postive
TUBE1	DTX2P1-UPK	0.547137336	2.05E-43	postive
ALOX12	DTX2P1-UPK	0.556959881	3.06E-45	postive
GABPB1	DTX2P1-UPK	0.496417499	7.02E-35	postive
LINC00472	DTX2P1-UPK	0.423701817	6.81E-25	postive
ATM	DTX2P1-UPK	0.734375004	1.98E-92	postive
FBXW7	DTX2P1-UPK	0.60728177	1.25E-55	postive
CA9	AC106897.1	0.420352024	1.73E-24	postive
TUBE1	LINC01402	0.53727692	1.22E-41	postive
SETD1B	LINC01402	0.401561663	2.65E-22	postive
ALOX12	LINC01402	0.548814779	1.01E-43	postive
ATM	LINC01402	0.544373431	6.54E-43	postive
FANCD2	AC116535.1	0.421071305	1.42E-24	postive
ALOX12	AC116535.1	0.454652215	7.48E-29	postive
TFAP2C	AC116535.1	0.457903612	2.71E-29	postive
HBA1	AC116535.1	0.42069855	1.57E-24	postive
DUOX1	AC116535.1	0.408307982	4.52E-23	postive
CHMP6	BAIAP2-DT	0.467944206	1.11E-30	postive
MAPK3	BAIAP2-DT	0.496103651	7.84E-35	postive
HELLS	PRR34	0.401798835	2.50E-22	postive
GABPB1	PRR34	0.469073912	7.68E-31	postive
ATG7	PRR34	0.460088267	1.37E-29	postive
LINC00472	PRR34	0.607114525	1.37E-55	postive
ATM	PRR34	0.56649408	4.50E-47	postive
HELLS	AC090527.3	0.408393442	4.42E-23	postive
ZNF419	AC090527.3	0.500004606	1.94E-35	postive
TUBE1	AC090527.3	0.565104478	8.40E-47	postive
ALOX12	AC090527.3	0.659854827	1.14E-68	postive
GABPB1	AC090527.3	0.424882305	4.89E-25	postive
ATM	AC090527.3	0.454718255	7.33E-29	postive
TAZ	AC090527.3	0.510758857	3.78E-37	postive
HSPA5	AC018638.7	-0.400126451	3.85E-22	negative
ZNF419	AC018638.7	0.557234347	2.71E-45	postive

TUBE1	AC018638.7	0.566471577	4.55E-47	postive
ALOX12	AC018638.7	0.467089131	1.46E-30	postive
GABPB1	AC018638.7	0.476772021	6.09E-32	postive
LINC00472	AC018638.7	0.565227993	7.95E-47	postive
ATM	AC018638.7	0.423151233	7.94E-25	postive
TAZ	AC018638.7	0.472636876	2.40E-31	postive
FBXW7	AC018638.7	0.427518914	2.32E-25	postive
NCF2	PTPRN2-AS1	0.663205188	1.37E-69	postive
FTL	PTPRN2-AS1	0.43781343	1.20E-26	postive
HAMP	PTPRN2-AS1	0.538717923	6.77E-42	postive
ALOX5	PTPRN2-AS1	0.509875002	5.25E-37	postive
CYBB	PTPRN2-AS1	0.672454133	3.38E-72	postive
ATG7	PTPRN2-AS1	0.473747571	1.66E-31	postive
IFNG	PTPRN2-AS1	0.45359829	1.04E-28	postive
GPX4	AL355001.2	0.450767438	2.47E-28	postive
OTUB1	AL355001.2	0.561447093	4.27E-46	postive
ATP5MC3	AL355001.2	0.423273393	7.67E-25	postive
ATG4D	AL355001.2	0.488423779	1.16E-33	postive
GABARAPL2	AL355001.2	0.564763408	9.78E-47	postive
TFAP2C	AP003555.2	0.522429837	4.46E-39	postive
HBA1	AP003555.2	0.494484308	1.39E-34	postive
DUOX1	AP003555.2	0.409575232	3.22E-23	postive
ALOX12	AC084117.1	0.411963627	1.70E-23	postive
PHKG2	AC084117.1	0.423115698	8.02E-25	postive
TAZ	AC084117.1	0.477475629	4.82E-32	postive
FBXW7	AC084117.1	0.44819457	5.42E-28	postive
HELLS	AP001033.2	0.480499958	1.75E-32	postive
KLHL24	AP001033.2	0.537402423	1.16E-41	postive
TUBE1	AP001033.2	0.461115703	9.87E-30	postive
IREB2	AP001033.2	0.4284432	1.79E-25	postive
GABPB1	AP001033.2	0.582365888	2.94E-50	postive
PIK3CA	AP001033.2	0.450031213	3.10E-28	postive
KRAS	AP001033.2	0.433996181	3.64E-26	postive
MAPK8	AP001033.2	0.426850055	2.81E-25	postive
LINC00472	AP001033.2	0.855191764	2.19E-155	postive
LPIN1	AP001033.2	0.425993021	3.58E-25	postive
ATM	AP001033.2	0.646796257	3.45E-65	postive
FBXW7	AP001033.2	0.459105533	1.86E-29	postive
ANGPTL7	HOTAIR	0.445856692	1.10E-27	postive
CS	REPIN1-AS1	0.516696248	4.03E-38	postive
GABARAPL1	REPIN1-AS1	0.422866565	8.59E-25	postive
WIPI2	REPIN1-AS1	0.437794231	1.20E-26	postive
HBA1	AL513165.1	0.551171365	3.71E-44	postive
HRAS	AL513165.1	0.579278783	1.26E-49	postive
EGLN2	AL513165.1	0.750721372	9.43E-99	postive
LAMP2	AC124067.4	0.46193672	7.61E-30	postive
HELLS	IRF1-AS1	0.521707371	5.90E-39	postive
ZNF419	IRF1-AS1	0.42499045	4.74E-25	postive
TUBE1	IRF1-AS1	0.579153543	1.34E-49	postive
ALOX12	IRF1-AS1	0.581175681	5.16E-50	postive
GABPB1	IRF1-AS1	0.565042266	8.63E-47	postive
LINC00472	IRF1-AS1	0.554972127	7.25E-45	postive
ATM	IRF1-AS1	0.740766768	7.61E-95	postive
FBXW7	IRF1-AS1	0.52669492	8.43E-40	postive
PHKG2	AC106820.3	0.400376232	3.61E-22	postive
HSPB1	AC023509.3	0.437433754	1.34E-26	postive
SLC2A8	AC023509.3	0.437485578	1.32E-26	postive
HBA1	AC023509.3	0.434619357	3.04E-26	postive

HRAS	AC023509.3	0.638162233	5.58E-63	postive
PHKG2	AC023509.3	0.442077579	3.40E-27	postive
MAP1LC3A	AC023509.3	0.539972689	4.05E-42	postive
EGLN2	AC023509.3	0.703743534	9.19E-82	postive
FANCD2	AC010976.1	0.416006981	5.69E-24	postive
HELLS	AC010976.1	0.612088262	1.02E-56	postive
ZNF419	AC010976.1	0.454803039	7.14E-29	postive
TUBE1	AC010976.1	0.541573762	2.09E-42	postive
ALOX12	AC010976.1	0.611133436	1.68E-56	postive
GABPB1	AC010976.1	0.551672994	2.99E-44	postive
LINC00472	AC010976.1	0.562614551	2.55E-46	postive
ATM	AC010976.1	0.619867807	1.59E-58	postive
FBXW7	AC010976.1	0.526611765	8.71E-40	postive
ISCU	AP001033.1	0.454932906	6.85E-29	postive
HELLS	AC005632.3	0.507514082	1.26E-36	postive
TUBE1	AC005632.3	0.498136074	3.80E-35	postive
ALOX12	AC005632.3	0.497145732	5.41E-35	postive
GABPB1	AC005632.3	0.490628706	5.39E-34	postive
LINC00472	AC005632.3	0.630762287	3.84E-61	postive
ATM	AC005632.3	0.619381977	2.07E-58	postive
FBXW7	AC005632.3	0.441761911	3.73E-27	postive
HELLS	AP002449.1	0.458891507	1.99E-29	postive
TUBE1	AP002449.1	0.494180263	1.55E-34	postive
GABPB1	AP002449.1	0.515996635	5.26E-38	postive
MAPK8	AP002449.1	0.42546196	4.15E-25	postive
LINC00472	AP002449.1	0.445034866	1.41E-27	postive
ATM	AP002449.1	0.51385617	1.18E-37	postive
BRD4	THBS3-AS1	0.469167347	7.45E-31	postive
ZNF419	THBS3-AS1	0.514785578	8.33E-38	postive
VEGFA	THBS3-AS1	0.465472264	2.46E-30	postive
TUBE1	THBS3-AS1	0.441646859	3.86E-27	postive
SETD1B	THBS3-AS1	0.438392599	1.01E-26	postive
DRD4	THBS3-AS1	0.41005141	2.84E-23	postive
ALOX12	THBS3-AS1	0.591175895	4.19E-52	postive
PHKG2	THBS3-AS1	0.492506691	2.80E-34	postive
ULK1	THBS3-AS1	0.434399611	3.24E-26	postive
YY1AP1	THBS3-AS1	0.417425975	3.86E-24	postive
TAZ	THBS3-AS1	0.618001578	4.35E-58	postive
ZNF419	AC100803.4	0.484810113	4.03E-33	postive
VEGFA	AC100803.4	0.455364299	5.99E-29	postive
ALOX12	AC100803.4	0.440687298	5.14E-27	postive
HIC1	AC100803.4	0.427971681	2.04E-25	postive
TAZ	AC100803.4	0.484583666	4.36E-33	postive
HBA1	AC087379.2	0.51134921	3.03E-37	postive
HRAS	AC087379.2	0.470974567	4.13E-31	postive
EGLN2	AC087379.2	0.632962645	1.10E-61	postive
FANCD2	AC008676.1	0.400160362	3.82E-22	postive
ALOX12	AC008676.1	0.411453288	1.95E-23	postive
TFAP2C	AC008676.1	0.533736785	5.12E-41	postive
HBA1	AC008676.1	0.536272283	1.84E-41	postive
DUOX1	AC008676.1	0.438800544	8.96E-27	postive
CHMP5	TMEM92-AS	-0.403498777	1.60E-22	negative
STEAP3	TMEM92-AS	0.502585398	7.65E-36	postive
FANCD2	AC026401.3	0.461439492	8.91E-30	postive
HELLS	AC026401.3	0.43360465	4.08E-26	postive
SLC2A6	AC026401.3	0.439298393	7.74E-27	postive
STMN1	AC026401.3	0.524542871	1.96E-39	postive
RRM2	AC026401.3	0.540964937	2.69E-42	postive

AURKA	AC026401.3	0.520791321	8.40E-39	postive
HRAS	AC026401.3	0.402153164	2.28E-22	postive
SLC1A5	AC026401.3	0.452657134	1.38E-28	postive
NCOA4	AC026401.3	-0.514441993	9.48E-38	negative
PHKG2	AC026401.3	0.488366201	1.18E-33	postive
BID	AC026401.3	0.452788123	1.33E-28	postive
CDKN2A	AC026401.3	0.472362311	2.62E-31	postive
PRKAA2	AC026401.3	-0.419588355	2.13E-24	negative
TAZ	AC026401.3	0.49845425	3.39E-35	postive
FANCD2	TDRKH-AS1	0.416963517	4.39E-24	postive
TFAP2C	TDRKH-AS1	0.456397899	4.35E-29	postive
HBA1	TDRKH-AS1	0.454746592	7.26E-29	postive
ALOX12	LINC02569	0.436832983	1.60E-26	postive
HELLS	AC007347.1	0.503949012	4.66E-36	postive
ALOX12	AC007347.1	0.402411632	2.13E-22	postive
GABPB1	AC007347.1	0.495767724	8.84E-35	postive
ATG7	AC007347.1	0.455791762	5.25E-29	postive
LINC00472	AC007347.1	0.577797464	2.53E-49	postive
ATM	AC007347.1	0.69762264	8.55E-80	postive
FBXW7	AC007347.1	0.435652722	2.25E-26	postive
ZNF419	MRPS9-AS1	0.540431522	3.35E-42	postive
VEGFA	MRPS9-AS1	0.40134069	2.81E-22	postive
TUBE1	MRPS9-AS1	0.599679898	6.12E-54	postive
SETD1B	MRPS9-AS1	0.434524726	3.12E-26	postive
ALOX12	MRPS9-AS1	0.56885694	1.55E-47	postive
GABPB1	MRPS9-AS1	0.458161049	2.50E-29	postive
LINC00472	MRPS9-AS1	0.484777263	4.08E-33	postive
ATM	MRPS9-AS1	0.449197776	3.99E-28	postive
TAZ	MRPS9-AS1	0.525964797	1.12E-39	postive
FBXW7	MRPS9-AS1	0.459821136	1.49E-29	postive
GPX4	LINC01637	0.436934572	1.55E-26	postive
GCLC	LINC01637	-0.407015227	6.36E-23	negative
PIK3CA	LINC01637	-0.406052766	8.20E-23	negative
PHKG2	LINC01637	0.499942012	1.99E-35	postive
PRKAA1	LINC01637	-0.427411907	2.40E-25	negative
EPAS1	LINC01637	-0.40754001	5.54E-23	negative
SIRT1	LINC01637	-0.416727688	4.68E-24	negative
TUBE1	AL353622.1	0.498260317	3.64E-35	postive
ALOX12	AL353622.1	0.524385295	2.08E-39	postive
PHKG2	AL353622.1	0.487736107	1.47E-33	postive
TAZ	AL353622.1	0.586606157	3.86E-51	postive
ZNF419	AC068620.1	0.452695849	1.37E-28	postive
TUBE1	AC068620.1	0.445728677	1.14E-27	postive
ALOX12	AC068620.1	0.458607961	2.18E-29	postive
GABPB1	AC068620.1	0.453223485	1.16E-28	postive
ATM	AC068620.1	0.552978122	1.71E-44	postive
FBXW7	AC068620.1	0.400846927	3.19E-22	postive
ZNF419	AC008875.1	0.403060623	1.80E-22	postive
PHKG2	AC008875.1	0.657964288	3.73E-68	postive
TAZ	AC008875.1	0.637807918	6.85E-63	postive
HELLS	AL133342.1	0.521667746	5.99E-39	postive
GABPB1	AL133342.1	0.502211692	8.76E-36	postive
ATG7	AL133342.1	0.469275217	7.20E-31	postive
LINC00472	AL133342.1	0.44924344	3.94E-28	postive
ATM	AL133342.1	0.687233699	1.45E-76	postive
FBXW7	AL133342.1	0.473217613	1.98E-31	postive
ZNF419	AC245884.8	0.531068559	1.49E-40	postive
TUBE1	AC245884.8	0.498874	2.92E-35	postive

DRD4	AC245884.8	0.43021492	1.08E-25	postive
ALOX12	AC245884.8	0.568645538	1.70E-47	postive
PHKG2	AC245884.8	0.565343553	7.55E-47	postive
TAZ	AC245884.8	0.666746242	1.41E-70	postive
FANCD2	PDE2A-AS2	0.543754537	8.46E-43	postive
JDP2	PDE2A-AS2	0.586284841	4.51E-51	postive
ALOX12	AC067930.3	0.403501576	1.60E-22	postive
TFAP2C	AC245100.5	0.55907038	1.22E-45	postive
HBA1	AC245100.5	0.496317182	7.27E-35	postive
DUOX1	AC245100.5	0.410136351	2.78E-23	postive
HSPA5	AC015961.2	-0.40316961	1.75E-22	negative
ZNF419	AC015961.2	0.443864035	2.00E-27	postive
NCOA4	AC015961.2	-0.418626199	2.78E-24	negative
PHKG2	AC015961.2	0.488473607	1.14E-33	postive
TAZ	AC015961.2	0.624229828	1.46E-59	postive
HELLS	ERVK13-1	0.47605192	7.75E-32	postive
ZNF419	ERVK13-1	0.555740639	5.20E-45	postive
VEGFA	ERVK13-1	0.447285838	7.13E-28	postive
TUBE1	ERVK13-1	0.672373457	3.57E-72	postive
SETD1B	ERVK13-1	0.504798951	3.41E-36	postive
ALOX12	ERVK13-1	0.693169581	2.15E-78	postive
GABPB1	ERVK13-1	0.543554433	9.20E-43	postive
MAPK8	ERVK13-1	0.425693702	3.89E-25	postive
LINC00472	ERVK13-1	0.512838241	1.73E-37	postive
ATM	ERVK13-1	0.671991533	4.59E-72	postive
TAZ	ERVK13-1	0.433190083	4.59E-26	postive
FBXW7	ERVK13-1	0.532761082	7.57E-41	postive
HELLS	ALOX12-AS1	0.428201713	1.92E-25	postive
ZNF419	ALOX12-AS1	0.485663082	3.01E-33	postive
VEGFA	ALOX12-AS1	0.426397301	3.19E-25	postive
TUBE1	ALOX12-AS1	0.584988983	8.40E-51	postive
SETD1B	ALOX12-AS1	0.482860896	7.84E-33	postive
ALOX12	ALOX12-AS1	0.891803543	4.11E-187	postive
ULK2	ALOX12-AS1	0.421158752	1.38E-24	postive
ATM	ALOX12-AS1	0.579505637	1.13E-49	postive
YY1AP1	ALOX12-AS1	0.45969259	1.55E-29	postive
ATF4	SNHG8	0.427800081	2.15E-25	postive
RPL8	SNHG8	0.480367715	1.82E-32	postive
NCOA4	AC145423.1	-0.408405046	4.40E-23	negative
PHKG2	AC145423.1	0.526209414	1.02E-39	postive
TAZ	AC145423.1	0.595344101	5.36E-53	postive
FANCD2	PRR7-AS1	0.404896459	1.11E-22	postive
HELLS	PRR7-AS1	0.528808102	3.66E-40	postive
ALOX12	PRR7-AS1	0.49080235	5.08E-34	postive
TFAP2C	PRR7-AS1	0.403376008	1.66E-22	postive
FANCD2	SPART-AS1	0.44539424	1.26E-27	postive
PEBP1	AL121992.3	0.565560235	6.85E-47	postive
MIOX	AL121992.3	0.440196974	5.94E-27	postive
VEGFA	LINC00173	0.454967259	6.78E-29	postive
TUBE1	LINC00173	0.421843917	1.14E-24	postive
ALOX12	LINC00173	0.591487216	3.60E-52	postive
TAZ	LINC00173	0.480967682	1.49E-32	postive
FANCD2	AC115618.1	0.440268254	5.81E-27	postive
ALOX12	AC026803.1	0.460788634	1.09E-29	postive
TAZ	AC026803.1	0.404982379	1.09E-22	postive
KLHL24	ATP1B3-AS1	0.458918093	1.97E-29	postive
TUBE1	ATP1B3-AS1	0.427384582	2.41E-25	postive
IREB2	ATP1B3-AS1	0.409239412	3.53E-23	postive

GABPB1	ATP1B3-AS1	0.519126151	1.59E-38	postive
PIK3CA	ATP1B3-AS1	0.441999716	3.48E-27	postive
MAPK8	ATP1B3-AS1	0.436885603	1.57E-26	postive
LINC00472	ATP1B3-AS1	0.805578342	3.63E-124	postive
ATM	ATP1B3-AS1	0.545506856	4.07E-43	postive
FBXW7	ATP1B3-AS1	0.413002483	1.29E-23	postive
ZNF419	AP003352.1	0.463760902	4.26E-30	postive
TUBE1	AP003352.1	0.438696464	9.24E-27	postive
HRAS	AP003352.1	0.424299057	5.76E-25	postive
NCOA4	AP003352.1	-0.459196502	1.81E-29	negative
PHKG2	AP003352.1	0.626786054	3.56E-60	postive
EGLN2	AP003352.1	0.457230436	3.35E-29	postive
TAZ	AP003352.1	0.702773197	1.90E-81	postive
HELLS	AL390195.2	0.444551611	1.62E-27	postive
TUBE1	AL390195.2	0.530799638	1.66E-40	postive
ALOX12	AL390195.2	0.452135844	1.63E-28	postive
IREB2	AL390195.2	0.412242677	1.58E-23	postive
GABPB1	AL390195.2	0.568619742	1.72E-47	postive
MAPK8	AL390195.2	0.517573231	2.89E-38	postive
LINC00472	AL390195.2	0.587257506	2.82E-51	postive
ATM	AL390195.2	0.702030346	3.31E-81	postive
FBXW7	AL390195.2	0.48812339	1.29E-33	postive
ALOX12	HOXA-AS2	0.407904107	5.03E-23	postive
PHKG2	HOXA-AS2	0.431145396	8.27E-26	postive
TAZ	HOXA-AS2	0.501475482	1.14E-35	postive
FANCD2	LIX1L-AS1	0.403627517	1.55E-22	postive
HELLS	LIX1L-AS1	0.475578507	9.07E-32	postive
ZNF419	LIX1L-AS1	0.475359972	9.75E-32	postive
TUBE1	LIX1L-AS1	0.483025066	7.41E-33	postive
ALOX12	LIX1L-AS1	0.610982495	1.82E-56	postive
GABPB1	LIX1L-AS1	0.422432963	9.70E-25	postive
FBXW7	LIX1L-AS1	0.439726419	6.82E-27	postive
LURAP1L	AL357033.2	0.414034287	9.73E-24	postive
KLHL24	RBM26-AS1	0.434672645	2.99E-26	postive
TUBE1	RBM26-AS1	0.491102855	4.57E-34	postive
IREB2	RBM26-AS1	0.508558138	8.56E-37	postive
GABPB1	RBM26-AS1	0.464576958	3.28E-30	postive
PIK3CA	RBM26-AS1	0.40764179	5.39E-23	postive
MAPK8	RBM26-AS1	0.509362004	6.35E-37	postive
LINC00472	RBM26-AS1	0.547751597	1.58E-43	postive
PRKAA2	RBM26-AS1	0.428647009	1.69E-25	postive
PRKAA1	RBM26-AS1	0.461311522	9.28E-30	postive
ATM	RBM26-AS1	0.604052343	6.63E-55	postive
ISCU	ITGA9-AS1	0.464561219	3.30E-30	postive
KLHL24	ITGA9-AS1	0.592505762	2.18E-52	postive
IREB2	ITGA9-AS1	0.400438909	3.55E-22	postive
PIK3CA	ITGA9-AS1	0.454879319	6.97E-29	postive
GABARAPL1	ITGA9-AS1	0.462328658	6.72E-30	postive
LINC00472	ITGA9-AS1	0.727781084	5.19E-90	postive
LPIN1	ITGA9-AS1	0.514400555	9.63E-38	postive
HBA1	AC011481.2	0.5583833	1.64E-45	postive
EGLN2	AC011481.2	0.401862781	2.45E-22	postive
HELLS	AC002128.1	0.478753313	3.14E-32	postive
ZNF419	AC002128.1	0.602727113	1.30E-54	postive
TUBE1	AC002128.1	0.603885096	7.22E-55	postive
SETD1B	AC002128.1	0.447596323	6.49E-28	postive
ALOX12	AC002128.1	0.648001737	1.67E-65	postive
GABPB1	AC002128.1	0.52271025	4.00E-39	postive

LINC00472	AC002128.1	0.544674151	5.77E-43	postive
LPIN1	AC002128.1	0.420466912	1.67E-24	postive
ATM	AC002128.1	0.602024326	1.87E-54	postive
TAZ	AC002128.1	0.409136247	3.62E-23	postive
FBXW7	AC002128.1	0.519343834	1.47E-38	postive
HELLS	AC006480.2	0.445303728	1.30E-27	postive
ZNF419	AC006480.2	0.451354577	2.07E-28	postive
KLHL24	AC006480.2	0.436891789	1.57E-26	postive
TUBE1	AC006480.2	0.498401628	3.46E-35	postive
ALOX12	AC006480.2	0.465443047	2.48E-30	postive
GABPB1	AC006480.2	0.513622249	1.29E-37	postive
LINC00472	AC006480.2	0.817199339	1.30E-130	postive
LPIN1	AC006480.2	0.427427835	2.39E-25	postive
ATM	AC006480.2	0.504587659	3.69E-36	postive
FBXW7	AC006480.2	0.41641661	5.09E-24	postive
HELLS	PITRM1-AS1	0.410212817	2.72E-23	postive
ARNTL	PITRM1-AS1	0.478676865	3.22E-32	postive
ZNF419	PITRM1-AS1	0.409106225	3.65E-23	postive
VEGFA	PITRM1-AS1	0.462401564	6.56E-30	postive
TUBE1	PITRM1-AS1	0.551722506	2.93E-44	postive
ALOX12	PITRM1-AS1	0.567636171	2.69E-47	postive
GABPB1	PITRM1-AS1	0.476617295	6.42E-32	postive
MAPK8	PITRM1-AS1	0.427029302	2.67E-25	postive
ATM	PITRM1-AS1	0.629002611	1.03E-60	postive
FBXW7	PITRM1-AS1	0.490657893	5.34E-34	postive
HELLS	AP001001.1	0.434056801	3.58E-26	postive
ZNF419	AP001001.1	0.447329565	7.04E-28	postive
KLHL24	AP001001.1	0.454781286	7.18E-29	postive
TUBE1	AP001001.1	0.558750379	1.40E-45	postive
ALOX12	AP001001.1	0.512076805	2.31E-37	postive
IREB2	AP001001.1	0.41061082	2.44E-23	postive
GABPB1	AP001001.1	0.57614329	5.46E-49	postive
PIK3CA	AP001001.1	0.407530614	5.55E-23	postive
MAPK8	AP001001.1	0.438559362	9.62E-27	postive
LINC00472	AP001001.1	0.809549088	2.56E-126	postive
ATM	AP001001.1	0.672908048	2.51E-72	postive
FBXW7	AP001001.1	0.48586279	2.81E-33	postive
RB1	AL357054.4	0.432379911	5.80E-26	postive
HMGB1	AL357054.4	0.49082202	5.04E-34	postive
GABPB1	AL357054.4	0.41672934	4.68E-24	postive
ZEB1	AL357054.4	0.428961867	1.54E-25	postive
TLR4	AL357054.4	0.449526824	3.61E-28	postive
HELLS	AC093690.1	0.581795231	3.85E-50	postive
TUBE1	AC093690.1	0.491667941	3.75E-34	postive
ALOX12	AC093690.1	0.522684552	4.04E-39	postive
GABPB1	AC093690.1	0.539597969	4.72E-42	postive
LINC00472	AC093690.1	0.618000349	4.36E-58	postive
ATM	AC093690.1	0.732394354	1.07E-91	postive
FBXW7	AC093690.1	0.50607228	2.14E-36	postive
TRIB3	SNHG15	0.405739541	8.91E-23	postive
CXCL2	SNHG15	0.406375851	7.53E-23	postive
SLC2A6	SNHG15	0.400020588	3.96E-22	postive
NCOA4	SNHG15	-0.425549763	4.05E-25	negative
PHKG2	SNHG15	0.499900529	2.02E-35	postive
TAZ	SNHG15	0.539083624	5.83E-42	postive
LAMP2	AC005332.5	-0.426036184	3.53E-25	negative
CHMP5	AC005332.5	-0.426217165	3.36E-25	negative
DRD4	AC005332.5	0.487887993	1.40E-33	postive

ALOX12	AC005332.5	0.51825832	2.22E-38	postive
NCOA4	AC005332.5	-0.49641506	7.02E-35	negative
PHKG2	AC005332.5	0.563093384	2.06E-46	postive
TAZ	AC005332.5	0.713576222	4.93E-85	postive
ALOX12	AC007406.2	0.412995326	1.29E-23	postive
PEBP1	AC007406.2	0.414687027	8.15E-24	postive
MIOX	AC007406.2	0.467998337	1.09E-30	postive
HELLS	AC110015.1	0.632769069	1.23E-61	postive
ZNF419	AC110015.1	0.428610577	1.71E-25	postive
ZFP69B	AC110015.1	0.40358289	1.57E-22	postive
TUBE1	AC110015.1	0.475201398	1.03E-31	postive
ALOX12	AC110015.1	0.524529741	1.97E-39	postive
GABPB1	AC110015.1	0.510509561	4.14E-37	postive
ATM	AC110015.1	0.692372401	3.81E-78	postive
FBXW7	AC110015.1	0.497675349	4.48E-35	postive
ALOX12	HOXB-AS1	0.53007542	2.21E-40	postive
PHKG2	HOXB-AS1	0.478642115	3.26E-32	postive
TAZ	HOXB-AS1	0.600101873	4.94E-54	postive
TFAP2C	AL645728.1	0.530664319	1.75E-40	postive
HBA1	AL645728.1	0.446905052	8.00E-28	postive
KLHL24	AC078993.1	0.454841523	7.05E-29	postive
LINC00472	AC078993.1	0.645590942	7.08E-65	postive
ANGPTL7	AP001107.5	0.499523575	2.31E-35	postive
LAMP2	AL713998.1	0.513065483	1.59E-37	postive
FANCD2	AC245014.3	0.417589804	3.69E-24	postive
HELLS	AC245014.3	0.422537884	9.42E-25	postive
TFAP2C	AC245014.3	0.528250517	4.57E-40	postive
HBA1	AC245014.3	0.479990715	2.07E-32	postive
DUOX1	AC245014.3	0.439970058	6.35E-27	postive
MAPK1	LINC01671	0.478667989	3.23E-32	postive
PRKAA2	LINC01671	0.408350041	4.47E-23	postive
HELLS	AC068790.2	0.54673765	2.43E-43	postive
TUBE1	AC068790.2	0.508853778	7.67E-37	postive
ALOX12	AC068790.2	0.494785111	1.25E-34	postive
IREB2	AC068790.2	0.425012092	4.71E-25	postive
GABPB1	AC068790.2	0.592605767	2.08E-52	postive
PIK3CA	AC068790.2	0.40376811	1.49E-22	postive
MAPK8	AC068790.2	0.466090986	2.02E-30	postive
LINC00472	AC068790.2	0.732459716	1.01E-91	postive
ATM	AC068790.2	0.780495852	1.23E-111	postive
FBXW7	AC068790.2	0.516221797	4.83E-38	postive
IREB2	LINC00623	-0.419746074	2.04E-24	negative
PHKG2	LINC00623	0.505780506	2.38E-36	postive
MAPK1	LINC00623	-0.470775888	4.41E-31	negative
BID	LINC00623	0.420918728	1.48E-24	postive
MAPK8	LINC00623	-0.435505361	2.35E-26	negative
PRKAA2	LINC00623	-0.442464705	3.03E-27	negative
FANCD2	ZNF529-AS1	0.491499354	3.98E-34	postive
HELLS	ZNF529-AS1	0.503805023	4.91E-36	postive
ZNF419	ZNF529-AS1	0.51053663	4.10E-37	postive
ALOX12	ZNF529-AS1	0.47962166	2.35E-32	postive
TFAP2C	ZNF529-AS1	0.405161582	1.04E-22	postive
HELLS	AL031282.2	0.414797452	7.91E-24	postive
ZNF419	AL031282.2	0.509408405	6.24E-37	postive
VEGFA	AL031282.2	0.409153196	3.61E-23	postive
TUBE1	AL031282.2	0.520157922	1.07E-38	postive
SETD1B	AL031282.2	0.495594238	9.40E-35	postive
ALOX12	AL031282.2	0.713996125	3.55E-85	postive

ATM	AL031282.2	0.512968782	1.65E-37	postive
YY1AP1	AL031282.2	0.426859166	2.80E-25	postive
FBXW7	AL031282.2	0.471760484	3.20E-31	postive
KLHL24	AC025917.1	0.621492275	6.57E-59	postive
TUBE1	AC025917.1	0.407303336	5.90E-23	postive
IREB2	AC025917.1	0.495946304	8.30E-35	postive
GABPB1	AC025917.1	0.52667882	8.49E-40	postive
PIK3CA	AC025917.1	0.532900608	7.16E-41	postive
KRAS	AC025917.1	0.461175602	9.69E-30	postive
MAPK8	AC025917.1	0.457610704	2.98E-29	postive
LINC00472	AC025917.1	0.94502142	7.57E-263	postive
PRKAA1	AC025917.1	0.440761703	5.02E-27	postive
LPIN1	AC025917.1	0.419823083	2.00E-24	postive
ATM	AC025917.1	0.539610709	4.70E-42	postive
HELLS	AC011247.1	0.524265639	2.18E-39	postive
TUBE1	AC011247.1	0.41261058	1.43E-23	postive
ALOX12	AC011247.1	0.409468491	3.32E-23	postive
GABPB1	AC011247.1	0.541471993	2.18E-42	postive
ATG7	AC011247.1	0.430538888	9.84E-26	postive
LINC00472	AC011247.1	0.673953727	1.25E-72	postive
ATM	AC011247.1	0.607793798	9.62E-56	postive
FBXW7	AC011247.1	0.442416266	3.07E-27	postive
HELLS	AC010998.2	0.496259906	7.42E-35	postive
ZNF419	AC010998.2	0.479855007	2.17E-32	postive
VEGFA	AC010998.2	0.402138232	2.29E-22	postive
TUBE1	AC010998.2	0.566371149	4.76E-47	postive
SETD1B	AC010998.2	0.403515215	1.60E-22	postive
ALOX12	AC010998.2	0.713834999	4.02E-85	postive
GABPB1	AC010998.2	0.426455923	3.14E-25	postive
ATM	AC010998.2	0.593374132	1.42E-52	postive
FBXW7	AC010998.2	0.45178018	1.81E-28	postive
KLHL24	AL139022.2	0.530879551	1.61E-40	postive
IREB2	AL139022.2	0.454381135	8.13E-29	postive
GABPB1	AL139022.2	0.546624902	2.55E-43	postive
PIK3CA	AL139022.2	0.479849559	2.17E-32	postive
KRAS	AL139022.2	0.434493446	3.15E-26	postive
MAPK8	AL139022.2	0.441941971	3.54E-27	postive
LINC00472	AL139022.2	0.877659065	1.18E-173	postive
PRKAA1	AL139022.2	0.431384834	7.72E-26	postive
ATM	AL139022.2	0.54357978	9.10E-43	postive
TFAP2C	AC074032.1	0.404082426	1.38E-22	postive
HBA1	AC074032.1	0.578451343	1.86E-49	postive
EGLN2	AC074032.1	0.46187972	7.75E-30	postive
ZNF419	AC048382.2	0.568534999	1.79E-47	postive
KLHL24	AC048382.2	0.424789528	5.02E-25	postive
TUBE1	AC048382.2	0.561270014	4.62E-46	postive
SETD1B	AC048382.2	0.441927705	3.56E-27	postive
ALOX12	AC048382.2	0.526849568	7.94E-40	postive
GABPB1	AC048382.2	0.473555886	1.77E-31	postive
LINC00472	AC048382.2	0.724591496	7.25E-89	postive
ATM	AC048382.2	0.479865477	2.16E-32	postive
FBXW7	AC048382.2	0.433756521	3.90E-26	postive
LAMP2	AC137630.3	-0.404837156	1.13E-22	negative
HIC1	AC137630.3	0.407215702	6.03E-23	postive
HRAS	AC137630.3	0.436156016	1.94E-26	postive
NCOA4	AC137630.3	-0.482597594	8.58E-33	negative
PHKG2	AC137630.3	0.47250336	2.50E-31	postive
TAZ	AC137630.3	0.5237912	2.63E-39	postive

ISCU	MMEL1-AS1	0.404188111	1.34E-22	postive
KLHL24	MMEL1-AS1	0.428964329	1.54E-25	postive
BRD4	AL021707.6	0.431304348	7.90E-26	postive
ZNF419	AL021707.6	0.524172065	2.27E-39	postive
TUBE1	AL021707.6	0.424984932	4.75E-25	postive
DRD4	AL021707.6	0.459719897	1.53E-29	postive
ALOX12	AL021707.6	0.433541496	4.15E-26	postive
HRAS	AL021707.6	0.489853238	7.07E-34	postive
NCOA4	AL021707.6	-0.444785678	1.51E-27	negative
PHKG2	AL021707.6	0.625940794	5.69E-60	postive
SOCS1	AL021707.6	0.43446964	3.17E-26	postive
EGLN2	AL021707.6	0.62378146	1.87E-59	postive
TAZ	AL021707.6	0.731639739	2.03E-91	postive
FANCD2	U73166.1	0.462775979	5.83E-30	postive
ZNF419	U73166.1	0.419147541	2.41E-24	postive
TFAP2C	U73166.1	0.550069593	5.93E-44	postive
HBA1	U73166.1	0.405462158	9.58E-23	postive
DUOX1	U73166.1	0.405919611	8.49E-23	postive
LPIN1	U73166.1	0.409913771	2.95E-23	postive
FH	LINC00886	0.41651254	4.96E-24	postive
ISCU	LINC00886	0.517763596	2.69E-38	postive
HERPUD1	LINC00886	0.469702838	6.26E-31	postive
KLHL24	LINC00886	0.499705201	2.16E-35	postive
SLC2A12	LINC00886	0.4960063	8.12E-35	postive
CS	LINC00886	0.422048748	1.08E-24	postive
GOT1	LINC00886	0.451328646	2.08E-28	postive
ATG4D	LINC00886	0.494318853	1.48E-34	postive
GABARAPL1	LINC00886	0.695469131	4.10E-79	postive
WIPI2	LINC00886	0.448433747	5.04E-28	postive
LPIN1	LINC00886	0.53430343	4.07E-41	postive
SLC2A8	RNF207-AS1	0.44188544	3.60E-27	postive
HBA1	RNF207-AS1	0.428376976	1.82E-25	postive
HRAS	RNF207-AS1	0.570721508	6.63E-48	postive
MAP1LC3A	RNF207-AS1	0.459736505	1.53E-29	postive
EGLN2	RNF207-AS1	0.642291955	5.00E-64	postive
KLHL24	NBAT1	0.571710098	4.22E-48	postive
IREB2	NBAT1	0.455294781	6.13E-29	postive
GABPB1	NBAT1	0.429787263	1.22E-25	postive
PIK3CA	NBAT1	0.480497278	1.75E-32	postive
LINC00472	NBAT1	0.85315318	6.96E-154	postive
ATM	NBAT1	0.425027223	4.69E-25	postive
ALOX12	SPAG5-AS1	0.421019936	1.44E-24	postive
SRC	MRPL20-AS1	0.422808407	8.73E-25	postive
OTUB1	MRPL20-AS1	0.415055623	7.38E-24	postive
PHKG2	MRPL20-AS1	0.461272022	9.39E-30	postive
LINC00472	AC011503.2	0.518010871	2.44E-38	postive
LPIN1	AC011503.2	0.454528189	7.77E-29	postive
HELLS	AC090948.2	0.517815265	2.63E-38	postive
KLHL24	AC090948.2	0.433669319	4.00E-26	postive
TUBE1	AC090948.2	0.422973822	8.34E-25	postive
ALOX12	AC090948.2	0.41581872	5.99E-24	postive
IREB2	AC090948.2	0.466548995	1.74E-30	postive
GABPB1	AC090948.2	0.574879082	9.82E-49	postive
PIK3CA	AC090948.2	0.453692759	1.01E-28	postive
ATG7	AC090948.2	0.515849756	5.56E-38	postive
MAPK8	AC090948.2	0.425867715	3.71E-25	postive
LINC00472	AC090948.2	0.762253254	1.62E-103	postive
ATM	AC090948.2	0.750220645	1.50E-98	postive

FBXW7	AC090948.2	0.487616869	1.54E-33	postive
SLC3A2	AC097359.2	0.447683347	6.32E-28	postive
NFS1	AC097359.2	0.444427526	1.69E-27	postive
FH	AC097359.2	0.491217874	4.39E-34	postive
ISCU	AC097359.2	0.530069899	2.22E-40	postive
VLDLR	AC097359.2	0.406165699	7.96E-23	postive
KLHL24	AC097359.2	0.42414214	6.02E-25	postive
ATP5MC3	AC097359.2	0.465216617	2.67E-30	postive
SLC2A12	AC097359.2	0.620150105	1.36E-58	postive
ELAVL1	AC097359.2	0.41071258	2.38E-23	postive
CS	AC097359.2	0.463549898	4.55E-30	postive
GOT1	AC097359.2	0.509175582	6.81E-37	postive
ACO1	AC097359.2	0.572265637	3.27E-48	postive
ATG4D	AC097359.2	0.409179935	3.58E-23	postive
GABARAPL1	AC097359.2	0.512573917	1.92E-37	postive
WIPI1	AC097359.2	0.422077946	1.07E-24	postive
WIPI2	AC097359.2	0.674023766	1.20E-72	postive
LPIN1	AC097359.2	0.427557379	2.30E-25	postive
SLC3A2	ATP6V0E2-A	0.505574808	2.57E-36	postive
FH	ATP6V0E2-A	0.462927072	5.55E-30	postive
ISCU	ATP6V0E2-A	0.631901942	2.02E-61	postive
DDIT3	ATP6V0E2-A	0.510823227	3.69E-37	postive
SLC2A8	ATP6V0E2-A	0.482199305	9.82E-33	postive
SLC2A12	ATP6V0E2-A	0.630541728	4.35E-61	postive
CS	ATP6V0E2-A	0.480668149	1.65E-32	postive
GOT1	ATP6V0E2-A	0.472791302	2.28E-31	postive
ATG4D	ATP6V0E2-A	0.604959918	4.16E-55	postive
MAP1LC3A	ATP6V0E2-A	0.427135025	2.59E-25	postive
GABARAPL1	ATP6V0E2-A	0.537719451	1.02E-41	postive
WIPI2	ATP6V0E2-A	0.621367123	7.03E-59	postive
LPIN1	ATP6V0E2-A	0.595749091	4.39E-53	postive
ZNF419	AL356481.1	0.50494868	3.23E-36	postive
TUBE1	AL356481.1	0.559143834	1.18E-45	postive
ALOX12	AL356481.1	0.502333774	8.38E-36	postive
GABPB1	AL356481.1	0.47273512	2.32E-31	postive
ATM	AL356481.1	0.411162761	2.11E-23	postive
HELLS	AL096701.3	0.416466318	5.02E-24	postive
ZNF419	AL096701.3	0.500621419	1.56E-35	postive
TUBE1	AL096701.3	0.535328499	2.69E-41	postive
ALOX12	AL096701.3	0.539192957	5.57E-42	postive
GABPB1	AL096701.3	0.43543933	2.39E-26	postive
PHKG2	AL096701.3	0.476479155	6.72E-32	postive
LINC00472	AL096701.3	0.416362833	5.17E-24	postive
ATM	AL096701.3	0.413924559	1.00E-23	postive
TAZ	AL096701.3	0.598807087	9.50E-54	postive
FBXW7	AL096701.3	0.474539498	1.28E-31	postive
HELLS	AC004832.5	0.572924731	2.42E-48	postive
TUBE1	AC004832.5	0.459276264	1.76E-29	postive
ALOX12	AC004832.5	0.496453828	6.93E-35	postive
GABPB1	AC004832.5	0.534876776	3.23E-41	postive
LINC00472	AC004832.5	0.59171444	3.22E-52	postive
ATM	AC004832.5	0.711650796	2.21E-84	postive
FBXW7	AC004832.5	0.488859568	9.98E-34	postive
NOX1	AC067838.1	0.498436673	3.41E-35	postive
NCOA4	AC067838.1	-0.421234646	1.35E-24	negative
PHKG2	AC067838.1	0.495872575	8.52E-35	postive
BECN1	AC067838.1	-0.470546561	4.76E-31	negative
TAZ	AC067838.1	0.466872985	1.57E-30	postive

HELLS	AC004832.4	0.548844968	9.98E-44	postive
TUBE1	AC004832.4	0.448432575	5.04E-28	postive
ALOX12	AC004832.4	0.447018135	7.73E-28	postive
GABPB1	AC004832.4	0.528810417	3.66E-40	postive
ATG7	AC004832.4	0.401012966	3.06E-22	postive
LINC00472	AC004832.4	0.605122456	3.83E-55	postive
ATM	AC004832.4	0.719961093	3.12E-87	postive
FBXW7	AC004832.4	0.470081219	5.54E-31	postive
HELLS	AC131953.1	0.436065617	2.00E-26	postive
ZNF419	AC131953.1	0.49893248	2.86E-35	postive
TUBE1	AC131953.1	0.483755466	5.78E-33	postive
ALOX12	AC131953.1	0.470536225	4.77E-31	postive
GABPB1	AC131953.1	0.556287885	4.10E-45	postive
LINC00472	AC131953.1	0.637359632	8.88E-63	postive
ATM	AC131953.1	0.604817519	4.47E-55	postive
FBXW7	AC131953.1	0.53047925	1.89E-40	postive
ENPP2	AC093278.2	0.496241763	7.47E-35	postive
SLC1A4	AC093278.2	0.555467285	5.85E-45	postive
LURAP1L	AC093278.2	0.408795802	3.97E-23	postive
IL33	AC093278.2	0.451439674	2.01E-28	postive
HMGB1	AC093278.2	0.49309891	2.27E-34	postive
CAPG	AC093278.2	-0.404938612	1.10E-22	negative
MAPK3	AC093278.2	0.46935435	7.01E-31	postive
ZEB1	AC093278.2	0.762778624	9.70E-104	postive
MAPK8	AC093278.2	0.444843559	1.49E-27	postive
EPAS1	AC093278.2	0.79358705	5.86E-118	postive
SIRT1	AC093278.2	0.478467037	3.46E-32	postive
ZNF419	RNF213-AS1	0.432326379	5.89E-26	postive
VEGFA	RNF213-AS1	0.507475468	1.28E-36	postive
TUBE1	RNF213-AS1	0.651819458	1.66E-66	postive
SETD1B	RNF213-AS1	0.409755173	3.07E-23	postive
ALOX12	RNF213-AS1	0.663859946	9.00E-70	postive
GABPB1	RNF213-AS1	0.433617817	4.06E-26	postive
ATM	RNF213-AS1	0.541767417	1.93E-42	postive
TAZ	RNF213-AS1	0.427502974	2.34E-25	postive
FBXW7	RNF213-AS1	0.427868697	2.11E-25	postive
HSPA5	AC009118.3	-0.402928635	1.86E-22	negative
ZNF419	AC009118.3	0.57202312	3.66E-48	postive
TUBE1	AC009118.3	0.605504924	3.14E-55	postive
SETD1B	AC009118.3	0.402621038	2.02E-22	postive
ALOX12	AC009118.3	0.563326132	1.86E-46	postive
GABPB1	AC009118.3	0.449446583	3.70E-28	postive
PHKG2	AC009118.3	0.518573529	1.97E-38	postive
TAZ	AC009118.3	0.647249541	2.63E-65	postive
FBXW7	AC009118.3	0.419986317	1.91E-24	postive
HELLS	AC116158.1	0.561108717	4.96E-46	postive
TUBE1	AC116158.1	0.462384966	6.60E-30	postive
ALOX12	AC116158.1	0.449096352	4.12E-28	postive
GABPB1	AC116158.1	0.557532346	2.38E-45	postive
ATG7	AC116158.1	0.491704696	3.70E-34	postive
MAPK8	AC116158.1	0.405646284	9.13E-23	postive
LINC00472	AC116158.1	0.589083676	1.16E-51	postive
ATM	AC116158.1	0.773658166	1.67E-108	postive
FBXW7	AC116158.1	0.489407867	8.25E-34	postive
MTOR	AP003486.1	0.444100769	1.86E-27	postive
RPL8	AP003486.1	-0.444574103	1.61E-27	negative
LINC00472	AP003486.1	0.507299576	1.36E-36	postive
LPIN1	AP003486.1	0.438300305	1.04E-26	postive

TUBE1	AC005208.1	0.443081829	2.52E-27	postive
ALOX12	AC005208.1	0.565812701	6.11E-47	postive
ATM	AC005208.1	0.63464584	4.23E-62	postive
HELLS	AC087854.1	0.466051601	2.04E-30	postive
ZNF419	AC087854.1	0.402348643	2.16E-22	postive
KLHL24	AC087854.1	0.438092543	1.10E-26	postive
TUBE1	AC087854.1	0.42316983	7.90E-25	postive
IREB2	AC087854.1	0.52048443	9.46E-39	postive
GABPB1	AC087854.1	0.627637459	2.22E-60	postive
PIK3CA	AC087854.1	0.500727226	1.50E-35	postive
KRAS	AC087854.1	0.427102919	2.62E-25	postive
ATG7	AC087854.1	0.416859522	4.51E-24	postive
ZEB1	AC087854.1	0.482148986	9.99E-33	postive
MAPK8	AC087854.1	0.541232901	2.41E-42	postive
LINC00472	AC087854.1	0.689544875	2.85E-77	postive
PRKAA2	AC087854.1	0.402454084	2.11E-22	postive
PRKAA1	AC087854.1	0.42939772	1.36E-25	postive
ATM	AC087854.1	0.698266542	5.34E-80	postive
FBXW7	AC087854.1	0.524057562	2.37E-39	postive
FANCD2	AC092910.3	0.439493592	7.31E-27	postive
HELLS	AC092910.3	0.422144221	1.05E-24	postive
ZNF419	AC092910.3	0.587197518	2.90E-51	postive
KLHL24	AC092910.3	0.411506568	1.92E-23	postive
TUBE1	AC092910.3	0.546122525	3.14E-43	postive
SETD1B	AC092910.3	0.433960661	3.68E-26	postive
ALOX12	AC092910.3	0.626732784	3.67E-60	postive
GABPB1	AC092910.3	0.525199784	1.52E-39	postive
LINC00472	AC092910.3	0.494211043	1.53E-34	postive
LPIN1	AC092910.3	0.431741181	6.97E-26	postive
ATM	AC092910.3	0.496471176	6.88E-35	postive
FBXW7	AC092910.3	0.404036403	1.39E-22	postive
HSPB1	AC015917.2	0.425072863	4.63E-25	postive
HBA1	AC015917.2	0.541476845	2.18E-42	postive
HRAS	AC015917.2	0.548285837	1.26E-43	postive
EGLN2	AC015917.2	0.588187755	1.80E-51	postive
FANCD2	AC133550.3	0.437666477	1.25E-26	postive
HELLS	AC133550.3	0.420784182	1.53E-24	postive
ALOX12	AC133550.3	0.530402541	1.94E-40	postive
TFAP2C	AC133550.3	0.501238529	1.25E-35	postive
HBA1	AC133550.3	0.434041105	3.59E-26	postive
DUOX1	AC133550.3	0.445426022	1.25E-27	postive
HELLS	AP005131.5	0.522087469	5.09E-39	postive
GABPB1	AP005131.5	0.501115111	1.30E-35	postive
ATG7	AP005131.5	0.448666743	4.69E-28	postive
LINC00472	AP005131.5	0.555964404	4.72E-45	postive
ATM	AP005131.5	0.685275838	5.69E-76	postive
FBXW7	AP005131.5	0.467620344	1.23E-30	postive
ZNF419	AL158196.1	0.441642539	3.87E-27	postive
TUBE1	AL158196.1	0.492030239	3.30E-34	postive
ALOX12	AL158196.1	0.535924713	2.11E-41	postive
ATM	AL158196.1	0.454596779	7.61E-29	postive
TAZ	AL158196.1	0.502193666	8.81E-36	postive
TUBE1	AC011472.4	0.47514138	1.05E-31	postive
GABPB1	AC011472.4	0.51447512	9.36E-38	postive
LINC00472	AC011472.4	0.781782496	3.09E-112	postive
ATM	AC011472.4	0.546151496	3.11E-43	postive
FBXW7	AC011472.4	0.420213594	1.79E-24	postive
HELLS	AC012358.2	0.551640659	3.04E-44	postive

TUBE1	AC012358.2	0.48061016	1.68E-32	postive
ALOX12	AC012358.2	0.485601281	3.07E-33	postive
GABPB1	AC012358.2	0.506608263	1.76E-36	postive
LINC00472	AC012358.2	0.521941513	5.39E-39	postive
ATM	AC012358.2	0.59599644	3.88E-53	postive
FBXW7	AC012358.2	0.460767676	1.10E-29	postive
ALOX12	AC020779.2	0.458854004	2.01E-29	postive
MAPK8	AC020779.2	0.433156874	4.64E-26	postive
ATM	AC020779.2	0.407043284	6.31E-23	postive
BRD4	AC037459.2	0.464710006	3.14E-30	postive
ZNF419	AC037459.2	0.54668533	2.48E-43	postive
VEGFA	AC037459.2	0.508257217	9.56E-37	postive
TUBE1	AC037459.2	0.541709964	1.98E-42	postive
SETD1B	AC037459.2	0.644446765	1.40E-64	postive
ALOX12	AC037459.2	0.630317195	4.93E-61	postive
ATM	AC037459.2	0.411494658	1.93E-23	postive
YY1AP1	AC037459.2	0.409852116	2.99E-23	postive
TAZ	AC037459.2	0.42908508	1.49E-25	postive
FBXW7	AC037459.2	0.417381646	3.91E-24	postive
SLC40A1	AC005261.3	-0.400745301	3.28E-22	negative
ZNF419	AC005261.3	0.411782222	1.79E-23	postive
MAPK14	AC005261.3	-0.415833445	5.97E-24	negative
IREB2	AC005261.3	-0.414893395	7.71E-24	negative
NOX1	AC005261.3	0.522345617	4.61E-39	postive
LPCAT3	AC005261.3	-0.471326041	3.69E-31	negative
NRAS	AC005261.3	-0.427470405	2.36E-25	negative
HRAS	AC005261.3	0.410992803	2.21E-23	postive
NCOA4	AC005261.3	-0.613010699	6.25E-57	negative
PHKG2	AC005261.3	0.580014067	8.93E-50	postive
BECN1	AC005261.3	-0.514597981	8.94E-38	negative
MAPK1	AC005261.3	-0.489861904	7.05E-34	negative
ANO6	AC005261.3	-0.441209632	4.40E-27	negative
TAZ	AC005261.3	0.564677419	1.02E-46	postive
MTDH	AC005261.3	-0.409301106	3.47E-23	negative
HELLS	AL138787.2	0.579209359	1.30E-49	postive
TUBE1	AL138787.2	0.498659689	3.15E-35	postive
ALOX12	AL138787.2	0.496930498	5.85E-35	postive
GABPB1	AL138787.2	0.555247838	6.43E-45	postive
LINC00472	AL138787.2	0.617259546	6.49E-58	postive
ATM	AL138787.2	0.683578829	1.85E-75	postive
FBXW7	AL138787.2	0.53062854	1.78E-40	postive
ZFP36	MYADM-AS1	0.423545276	7.11E-25	postive
HELLS	AP005482.2	0.48158234	1.21E-32	postive
ZNF419	AP005482.2	0.412493064	1.48E-23	postive
KLHL24	AP005482.2	0.424404907	5.59E-25	postive
TUBE1	AP005482.2	0.457072813	3.52E-29	postive
GABPB1	AP005482.2	0.65337159	6.40E-67	postive
MAPK8	AP005482.2	0.433303774	4.45E-26	postive
LINC00472	AP005482.2	0.69835427	5.01E-80	postive
ATM	AP005482.2	0.517296534	3.21E-38	postive
FBXW7	AP005482.2	0.524404267	2.07E-39	postive
HELLS	AC087500.1	0.438759819	9.07E-27	postive
ZNF419	AC087500.1	0.566782719	3.95E-47	postive
VEGFA	AC087500.1	0.449958812	3.17E-28	postive
TUBE1	AC087500.1	0.649524698	6.67E-66	postive
SETD1B	AC087500.1	0.453045408	1.23E-28	postive
ALOX12	AC087500.1	0.796755039	1.47E-119	postive
ATM	AC087500.1	0.510110032	4.81E-37	postive

YY1AP1	AC087500.1	0.436915771	1.56E-26	postive
TAZ	AC087500.1	0.511662596	2.69E-37	postive
ZNF419	AC092111.1	0.40411237	1.37E-22	postive
ALOX12	LINC01058	0.432303652	5.93E-26	postive
HELLS	ADNP-AS1	0.409616189	3.19E-23	postive
ZNF419	ADNP-AS1	0.493742729	1.81E-34	postive
KLHL24	ADNP-AS1	0.506614719	1.75E-36	postive
TUBE1	ADNP-AS1	0.511087159	3.34E-37	postive
ALOX12	ADNP-AS1	0.409967754	2.90E-23	postive
GABPB1	ADNP-AS1	0.501162952	1.28E-35	postive
MAPK8	ADNP-AS1	0.400496247	3.50E-22	postive
LINC00472	ADNP-AS1	0.769081322	1.82E-106	postive
LPIN1	ADNP-AS1	0.421821373	1.15E-24	postive
ATM	ADNP-AS1	0.524428486	2.05E-39	postive
FBXW7	ADNP-AS1	0.402334423	2.17E-22	postive
HELLS	AC004223.4	0.576913788	3.82E-49	postive
TUBE1	AC004223.4	0.493243561	2.16E-34	postive
ALOX12	AC004223.4	0.522417872	4.48E-39	postive
GABPB1	AC004223.4	0.509501457	6.03E-37	postive
LINC00472	AC004223.4	0.413333468	1.18E-23	postive
ATM	AC004223.4	0.731601787	2.10E-91	postive
FBXW7	AC004223.4	0.527855284	5.34E-40	postive
HELLS	AC099343.2	0.50798325	1.06E-36	postive
TUBE1	AC099343.2	0.430913813	8.84E-26	postive
GABPB1	AC099343.2	0.59444015	8.40E-53	postive
ATG7	AC099343.2	0.440651645	5.19E-27	postive
LINC00472	AC099343.2	0.715099995	1.49E-85	postive
LPIN1	AC099343.2	0.437709716	1.23E-26	postive
ATM	AC099343.2	0.613689004	4.36E-57	postive
FBXW7	AC099343.2	0.486665359	2.13E-33	postive
ZNF419	SNHG21	0.491357901	4.18E-34	postive
VEGFA	SNHG21	0.400449242	3.54E-22	postive
TUBE1	SNHG21	0.425526856	4.08E-25	postive
SETD1B	SNHG21	0.402314498	2.18E-22	postive
ALOX12	SNHG21	0.488810194	1.02E-33	postive
PHKG2	SNHG21	0.522736997	3.96E-39	postive
TAZ	SNHG21	0.645247591	8.69E-65	postive
TFAP2C	LINC02803	0.531754669	1.13E-40	postive
HBA1	LINC02803	0.47806392	3.96E-32	postive
KLHL24	AP001021.3	0.563240275	1.93E-46	postive
IREB2	AP001021.3	0.413671635	1.07E-23	postive
GABPB1	AP001021.3	0.505528367	2.61E-36	postive
PIK3CA	AP001021.3	0.470826425	4.34E-31	postive
KRAS	AP001021.3	0.404869554	1.12E-22	postive
LINC00472	AP001021.3	0.949043562	1.82E-271	postive
ATM	AP001021.3	0.56140439	4.35E-46	postive
HELLS	AC025178.1	0.549662951	7.05E-44	postive
ZNF419	AC025178.1	0.45989107	1.45E-29	postive
TUBE1	AC025178.1	0.573553848	1.81E-48	postive
ALOX12	AC025178.1	0.554875278	7.56E-45	postive
GABPB1	AC025178.1	0.513468432	1.37E-37	postive
LINC00472	AC025178.1	0.484843721	3.99E-33	postive
ATM	AC025178.1	0.694384008	8.99E-79	postive
FBXW7	AC025178.1	0.537991663	9.11E-42	postive
PROM2	KLHDC7B-DT	0.650639826	3.40E-66	postive
FANCD2	AC010618.3	0.44761516	6.46E-28	postive
HELLS	AC010618.3	0.414644673	8.25E-24	postive
ALOX12	AC010618.3	0.467438467	1.31E-30	postive

TFAP2C	AC010618.3	0.535536122	2.47E-41	postive
HBA1	AC010618.3	0.459989632	1.41E-29	postive
DUOX1	AC010618.3	0.439142662	8.10E-27	postive
ZNF419	AC009087.1	0.463539739	4.57E-30	postive
TUBE1	AC009087.1	0.529802853	2.47E-40	postive
ALOX12	AC009087.1	0.476090033	7.65E-32	postive
GABPB1	AC009087.1	0.429989861	1.15E-25	postive
LINC00472	AC009087.1	0.689181041	3.69E-77	postive
ATM	AC009087.1	0.444832749	1.49E-27	postive
OTUB1	AL022328.4	0.406442092	7.40E-23	postive
PHKG2	AL022328.4	0.545554909	3.99E-43	postive
ATG4D	AL022328.4	0.41863291	2.77E-24	postive
TAZ	AL022328.4	0.557571591	2.34E-45	postive
ALOX12B	LINC01816	0.454180637	8.65E-29	postive
ZNF419	LINC01535	0.434553304	3.10E-26	postive
KLHL24	LINC01535	0.414912399	7.67E-24	postive
LINC00472	LINC01535	0.618316439	3.67E-58	postive
LPIN1	LINC01535	0.509280002	6.55E-37	postive
TFAP2C	AC018450.1	0.429153245	1.46E-25	postive
ALOX12	AC007098.1	0.514987904	7.71E-38	postive
TFAP2C	AC007098.1	0.416972376	4.37E-24	postive
HBA1	AC007098.1	0.412165126	1.61E-23	postive
ZNF419	LINC01534	0.495404275	1.01E-34	postive
VEGFA	LINC01534	0.457777005	2.82E-29	postive
TUBE1	LINC01534	0.564934089	9.06E-47	postive
ALOX12	LINC01534	0.695567399	3.82E-79	postive
GABPB1	LINC01534	0.434812971	2.87E-26	postive
MAPK8	LINC01534	0.461220047	9.55E-30	postive
ATM	LINC01534	0.7225607	3.81E-88	postive
YY1AP1	LINC01534	0.41897128	2.53E-24	postive
FBXW7	LINC01534	0.414879967	7.74E-24	postive
PML	WASL-DT	-0.424145492	6.01E-25	negative
TMBIM4	WASL-DT	0.460179066	1.33E-29	postive
CHMP5	WASL-DT	0.544016756	7.59E-43	postive
ATG5	WASL-DT	0.469474133	6.75E-31	postive
GABARAPL2	WASL-DT	0.498513294	3.32E-35	postive
HELLS	AC138956.1	0.400486739	3.51E-22	postive
ZNF419	AC138956.1	0.490433522	5.77E-34	postive
TUBE1	AC138956.1	0.535303399	2.72E-41	postive
ALOX12	AC138956.1	0.577081195	3.53E-49	postive
GABPB1	AC138956.1	0.436022175	2.02E-26	postive
LINC00472	AC138956.1	0.532237855	9.34E-41	postive
ATM	AC138956.1	0.575117801	8.79E-49	postive
FBXW7	AC138956.1	0.434669551	2.99E-26	postive
HELLS	AC005253.1	0.432461627	5.67E-26	postive
BRD4	AC005253.1	0.465452369	2.48E-30	postive
ZNF419	AC005253.1	0.560843263	5.58E-46	postive
VEGFA	AC005253.1	0.523036469	3.52E-39	postive
TUBE1	AC005253.1	0.616209108	1.14E-57	postive
SETD1B	AC005253.1	0.530155198	2.15E-40	postive
ALOX12	AC005253.1	0.747318575	2.14E-97	postive
GABPB1	AC005253.1	0.428396699	1.81E-25	postive
ATM	AC005253.1	0.533938363	4.72E-41	postive
YY1AP1	AC005253.1	0.42520814	4.46E-25	postive
TAZ	AC005253.1	0.52364267	2.78E-39	postive
FBXW7	AC005253.1	0.428453637	1.78E-25	postive
CISD2	LINC02027	0.471776329	3.18E-31	postive
SCP2	LINC02027	0.459241114	1.78E-29	postive

SNX4	LINC02027	0.537124396	1.30E-41	postive
MAPK1	LINC02027	0.442294844	3.19E-27	postive
PRKAA2	LINC02027	0.47637814	6.95E-32	postive
HSPB1	AC015912.3	0.419013824	2.50E-24	postive
HSF1	AC015912.3	0.421151423	1.38E-24	postive
HRAS	AC015912.3	0.454464525	7.92E-29	postive
NCOA4	AC015912.3	-0.443850441	2.00E-27	negative
PHKG2	AC015912.3	0.554770211	7.91E-45	postive
TAZ	AC015912.3	0.607008968	1.44E-55	postive
HELLS	AC005162.2	0.507784261	1.14E-36	postive
ZNF419	AC005162.2	0.405336184	9.90E-23	postive
TUBE1	AC005162.2	0.539671253	4.58E-42	postive
ALOX12	AC005162.2	0.549609629	7.21E-44	postive
GABPB1	AC005162.2	0.524927587	1.69E-39	postive
MAPK8	AC005162.2	0.445784639	1.12E-27	postive
LINC00472	AC005162.2	0.425315542	4.33E-25	postive
ATM	AC005162.2	0.652283811	1.25E-66	postive
FBXW7	AC005162.2	0.470853132	4.30E-31	postive
ACSL3	SNRK-AS1	0.422926444	8.45E-25	postive
KLHL24	SNRK-AS1	0.554726175	8.06E-45	postive
GABARAPL1	SNRK-AS1	0.520729414	8.61E-39	postive
LINC00472	SNRK-AS1	0.711140376	3.28E-84	postive
LPIN1	SNRK-AS1	0.633783119	6.92E-62	postive
HELLS	BACH1-IT1	0.589948214	7.64E-52	postive
TUBE1	BACH1-IT1	0.464942599	2.92E-30	postive
ALOX12	BACH1-IT1	0.471289081	3.73E-31	postive
GABPB1	BACH1-IT1	0.563064539	2.09E-46	postive
ATG7	BACH1-IT1	0.449915367	3.21E-28	postive
LINC00472	BACH1-IT1	0.574418643	1.21E-48	postive
ATM	BACH1-IT1	0.726635282	1.34E-89	postive
FBXW7	BACH1-IT1	0.517613564	2.84E-38	postive
HELLS	AC000123.1	0.516948244	3.66E-38	postive
ZNF419	AC000123.1	0.426641634	2.98E-25	postive
TUBE1	AC000123.1	0.506321425	1.95E-36	postive
ALOX12	AC000123.1	0.440177231	5.97E-27	postive
GABPB1	AC000123.1	0.578728119	1.63E-49	postive
LINC00472	AC000123.1	0.627745723	2.09E-60	postive
ATM	AC000123.1	0.598425999	1.15E-53	postive
FBXW7	AC000123.1	0.516283316	4.72E-38	postive
SP1	HCG15	0.446858383	8.12E-28	postive
SIRT1	HCG15	0.413975325	9.89E-24	postive
KLHL24	AC012467.1	0.426341832	3.24E-25	postive
LINC00472	AC012467.1	0.766616755	2.17E-105	postive
ATM	AC012467.1	0.437558564	1.29E-26	postive
ZNF419	FLJ37453	0.427573687	2.29E-25	postive
ALOX12	FLJ37453	0.428312956	1.86E-25	postive
TAZ	FLJ37453	0.444283215	1.76E-27	postive
HBA1	LINC00853	0.408324812	4.50E-23	postive
ALOX12B	LINC00853	0.400402698	3.58E-22	postive
FH	AC008966.1	0.40105925	3.02E-22	postive
SP1	LINC01428	0.451526159	1.96E-28	postive
PRKAA2	LINC01428	0.443933641	1.95E-27	postive
ATM	LINC01428	0.531126643	1.46E-40	postive
HELLS	AC010201.2	0.419822743	2.00E-24	postive
ZNF419	AC010201.2	0.579261933	1.27E-49	postive
VEGFA	AC010201.2	0.458854322	2.01E-29	postive
TUBE1	AC010201.2	0.628873219	1.11E-60	postive
SETD1B	AC010201.2	0.407025545	6.34E-23	postive

ALOX12	AC010201.2	0.71273969	9.47E-85	postive
GABPB1	AC010201.2	0.499710942	2.16E-35	postive
LINC00472	AC010201.2	0.45178334	1.81E-28	postive
ATM	AC010201.2	0.587655752	2.33E-51	postive
TAZ	AC010201.2	0.490734214	5.20E-34	postive
FBXW7	AC010201.2	0.427109381	2.61E-25	postive
FANCD2	AC074117.1	0.417499417	3.79E-24	postive
BRD4	AC074117.1	0.456773029	3.87E-29	postive
ZNF419	AC074117.1	0.480204086	1.93E-32	postive
TUBE1	AC074117.1	0.513231792	1.50E-37	postive
SETD1B	AC074117.1	0.446217329	9.85E-28	postive
DRD4	AC074117.1	0.442146454	3.33E-27	postive
ALOX12	AC074117.1	0.612997603	6.29E-57	postive
PHKG2	AC074117.1	0.564717355	9.99E-47	postive
TAZ	AC074117.1	0.651082697	2.59E-66	postive
TFAP2C	PPP1R35-AS	0.491625906	3.81E-34	postive
HBA1	PPP1R35-AS	0.530273802	2.05E-40	postive
HELLS	AC013468.1	0.535779658	2.24E-41	postive
ZNF419	AC013468.1	0.444992557	1.42E-27	postive
TUBE1	AC013468.1	0.613940538	3.82E-57	postive
ALOX12	AC013468.1	0.608574556	6.41E-56	postive
GABPB1	AC013468.1	0.493636159	1.88E-34	postive
ATM	AC013468.1	0.566698832	4.11E-47	postive
FBXW7	AC013468.1	0.483132515	7.15E-33	postive
GCH1	AC243829.4	0.522738685	3.96E-39	postive
IFNG	AC243829.4	0.678040074	8.11E-74	postive
TRIB3	AC010894.1	0.426174989	3.40E-25	postive
VEGFA	AC022144.1	0.473519896	1.79E-31	postive
TUBE1	AC022144.1	0.423595297	7.01E-25	postive
DRD4	AC022144.1	0.409150997	3.61E-23	postive
ALOX12	AC022144.1	0.546813093	2.35E-43	postive
PHKG2	AC022144.1	0.413907494	1.01E-23	postive
TAZ	AC022144.1	0.609463362	4.03E-56	postive
HELLS	LINC00630	0.567612399	2.72E-47	postive
MTOR	LINC00630	0.515585964	6.15E-38	postive
ACSL3	LINC00630	0.426332818	3.25E-25	postive
ZNF419	LINC00630	0.406506642	7.28E-23	postive
KLHL24	LINC00630	0.595623368	4.67E-53	postive
TUBE1	LINC00630	0.422038857	1.08E-24	postive
MAP3K5	LINC00630	0.448975674	4.27E-28	postive
EIF2AK4	LINC00630	0.428538991	1.74E-25	postive
IREB2	LINC00630	0.61046598	2.39E-56	postive
GABPB1	LINC00630	0.598632211	1.04E-53	postive
PIK3CA	LINC00630	0.581379086	4.69E-50	postive
KRAS	LINC00630	0.418675336	2.74E-24	postive
ATG7	LINC00630	0.520253933	1.03E-38	postive
MAPK8	LINC00630	0.528516125	4.11E-40	postive
LINC00472	LINC00630	0.689548028	2.85E-77	postive
PRKAA2	LINC00630	0.46407189	3.85E-30	postive
PRKAA1	LINC00630	0.499437327	2.38E-35	postive
TGFBR1	LINC00630	0.443800266	2.03E-27	postive
LPIN1	LINC00630	0.429809568	1.21E-25	postive
TLR4	LINC00630	0.411135793	2.12E-23	postive
ATM	LINC00630	0.767534481	8.64E-106	postive
FBXW7	LINC00630	0.575453829	7.52E-49	postive
BACH1	LINC00630	0.446745026	8.40E-28	postive
ZFP36	MIR23AHG	0.563787111	1.51E-46	postive
ATF3	MIR23AHG	0.520513189	9.36E-39	postive

VEGFA	MIR23AHG	0.438784151	9.00E-27	postive
TUBE1	LINC02604	0.431280599	7.96E-26	postive
DRD4	LINC02604	0.47099769	4.10E-31	postive
ALOX12	LINC02604	0.467840263	1.15E-30	postive
NCOA4	LINC02604	-0.467838011	1.15E-30	negative
PHKG2	LINC02604	0.678531461	5.81E-74	postive
TAZ	LINC02604	0.767639622	7.78E-106	postive
ALOX12	AC010519.1	0.417593075	3.69E-24	postive
TFAP2C	AC010519.1	0.496814559	6.09E-35	postive
HBA1	AC010519.1	0.582794395	2.39E-50	postive
FANCD2	AC074029.3	0.437266375	1.41E-26	postive
HELLS	AC074029.3	0.455079196	6.55E-29	postive
ALOX12	AC074029.3	0.593565414	1.29E-52	postive
TFAP2C	AC074029.3	0.429976161	1.16E-25	postive
DUOX1	AC074029.3	0.433545267	4.15E-26	postive
PHKG2	CACNA1C-A	0.527646786	5.80E-40	postive
TAZ	CACNA1C-A	0.561642655	3.92E-46	postive
TFAP2C	LINC02585	0.578137707	2.15E-49	postive
HBA1	LINC02585	0.509940376	5.12E-37	postive
DUOX1	LINC02585	0.414476998	8.63E-24	postive
FANCD2	AC010463.3	0.473721252	1.68E-31	postive
HELLS	AC010463.3	0.459771995	1.51E-29	postive
ZNF419	AC010463.3	0.453028044	1.24E-28	postive
ALOX12	AC010463.3	0.538383864	7.76E-42	postive
TFAP2C	AC010463.3	0.434712499	2.96E-26	postive
DUOX1	AC010463.3	0.417994281	3.31E-24	postive
ALOX12	AL353801.1	0.418811051	2.64E-24	postive
ATM	AL353801.1	0.480499698	1.75E-32	postive
VEGFA	MATN1-AS1	0.42578491	3.79E-25	postive
ALOX12	MATN1-AS1	0.406701726	6.91E-23	postive
PHKG2	MATN1-AS1	0.435144549	2.61E-26	postive
TAZ	MATN1-AS1	0.609442226	4.08E-56	postive
KLHL24	AC098869.2	0.534678045	3.50E-41	postive
TUBE1	AC098869.2	0.473945433	1.56E-31	postive
ALOX12	AC098869.2	0.427526247	2.32E-25	postive
IREB2	AC098869.2	0.407909483	5.02E-23	postive
GABPB1	AC098869.2	0.492617205	2.69E-34	postive
PIK3CA	AC098869.2	0.410353209	2.62E-23	postive
LINC00472	AC098869.2	0.873328862	7.26E-170	postive
ATM	AC098869.2	0.594150786	9.69E-53	postive
FBXW7	AC098869.2	0.408945919	3.81E-23	postive
IREB2	AP000897.2	0.402391916	2.14E-22	postive
GABPB1	AP000897.2	0.438618626	9.45E-27	postive
ATG7	AP000897.2	0.433068239	4.76E-26	postive
LINC00472	AP000897.2	0.418857519	2.61E-24	postive
PRKAA2	AP000897.2	0.40960273	3.20E-23	postive
ATM	AP000897.2	0.653336361	6.54E-67	postive
FBXW7	AP000897.2	0.424372301	5.64E-25	postive
EGFR	EGFR-AS1	0.542985213	1.17E-42	postive
FANCD2	NDUFB2-AS1	0.418415969	2.95E-24	postive
TFAP2C	NDUFB2-AS1	0.570593912	7.03E-48	postive
HBA1	NDUFB2-AS1	0.497984534	4.01E-35	postive
TUBE1	VIM-AS1	0.431913358	6.64E-26	postive
GABPB1	AL357140.2	0.443001557	2.58E-27	postive
MAPK8	AL357140.2	0.453587784	1.04E-28	postive
LINC00472	AL357140.2	0.403261083	1.71E-22	postive
PRKAA2	AL357140.2	0.456387354	4.36E-29	postive
ATM	AL357140.2	0.629515983	7.74E-61	postive

FBXW7	AL357140.2	0.422710521	8.98E-25	postive
FANCD2	AC011921.1	0.431303156	7.91E-26	postive
HELLS	AC011921.1	0.473086822	2.07E-31	postive
ALOX12	AC011921.1	0.52716383	7.01E-40	postive
ZNF419	AC024361.3	0.56005929	7.88E-46	postive
VEGFA	AC024361.3	0.510410461	4.30E-37	postive
TUBE1	AC024361.3	0.590969513	4.64E-52	postive
SETD1B	AC024361.3	0.474056103	1.50E-31	postive
ALOX12	AC024361.3	0.698779182	3.66E-80	postive
ATM	AC024361.3	0.455991758	4.93E-29	postive
TAZ	AC024361.3	0.549657991	7.07E-44	postive
FBXW7	AC024361.3	0.404804619	1.14E-22	postive
HELLS	AC018410.1	0.552928163	1.75E-44	postive
TUBE1	AC018410.1	0.474204451	1.43E-31	postive
ALOX12	AC018410.1	0.501266307	1.23E-35	postive
GABPB1	AC018410.1	0.555982905	4.68E-45	postive
ATG7	AC018410.1	0.454255067	8.46E-29	postive
LINC00472	AC018410.1	0.687799443	9.76E-77	postive
ATM	AC018410.1	0.737322989	1.55E-93	postive
FBXW7	AC018410.1	0.486305483	2.41E-33	postive
HSPB1	MAFG-DT	0.49373336	1.82E-34	postive
G6PD	MAFG-DT	0.46261311	6.14E-30	postive
HELLS	CLDN10-AS1	0.501645131	1.08E-35	postive
TUBE1	CLDN10-AS1	0.435272261	2.51E-26	postive
GABPB1	CLDN10-AS1	0.516917852	3.71E-38	postive
ATG7	CLDN10-AS1	0.456811913	3.82E-29	postive
LINC00472	CLDN10-AS1	0.65224625	1.28E-66	postive
ATM	CLDN10-AS1	0.670382638	1.32E-71	postive
FBXW7	CLDN10-AS1	0.429044293	1.51E-25	postive
KLHL24	MIR99AHG	0.54336185	9.97E-43	postive
TUBE1	MIR99AHG	0.429537759	1.31E-25	postive
IREB2	MIR99AHG	0.474171	1.45E-31	postive
GABPB1	MIR99AHG	0.559475145	1.02E-45	postive
PIK3CA	MIR99AHG	0.502610414	7.58E-36	postive
KRAS	MIR99AHG	0.404281832	1.31E-22	postive
MAPK8	MIR99AHG	0.462788937	5.80E-30	postive
LINC00472	MIR99AHG	0.797556192	5.75E-120	postive
PRKAA1	MIR99AHG	0.435592262	2.29E-26	postive
LPIN1	MIR99AHG	0.430628221	9.59E-26	postive
ATM	MIR99AHG	0.568943276	1.49E-47	postive
FBXW7	MIR99AHG	0.417120884	4.20E-24	postive
FANCD2	ATP2B1-AS1	0.415121525	7.24E-24	postive
TFAP2C	ATP2B1-AS1	0.51260897	1.89E-37	postive
HBA1	ATP2B1-AS1	0.426314232	3.27E-25	postive
ZNF419	AC104564.3	0.517280778	3.23E-38	postive
VEGFA	AC104564.3	0.449366404	3.79E-28	postive
TUBE1	AC104564.3	0.543175359	1.08E-42	postive
SETD1B	AC104564.3	0.503495564	5.49E-36	postive
ALOX12	AC104564.3	0.711886366	1.84E-84	postive
PHKG2	AC104564.3	0.418937061	2.55E-24	postive
TAZ	AC104564.3	0.566377168	4.74E-47	postive
ZNF419	AC135050.3	0.425945985	3.63E-25	postive
TUBE1	AC135050.3	0.474301842	1.38E-31	postive
DRD4	AC135050.3	0.460650636	1.14E-29	postive
ALOX12	AC135050.3	0.554206276	1.01E-44	postive
PHKG2	AC135050.3	0.60074636	3.57E-54	postive
TAZ	AC135050.3	0.716911237	3.56E-86	postive
HELLS	AC089999.2	0.540877088	2.79E-42	postive

ZNF419	AC089999.2	0.44415819	1.83E-27 postive
ALOX12	AC089999.2	0.433828732	3.82E-26 postive
GABPB1	AC089999.2	0.468153517	1.04E-30 postive
LINC00472	AC089999.2	0.618265165	3.78E-58 postive
ATM	AC089999.2	0.493845489	1.74E-34 postive
FBXW7	AC089999.2	0.425797129	3.78E-25 postive
HELLS	AL157871.2	0.500711288	1.51E-35 postive
TUBE1	AL157871.2	0.438830686	8.88E-27 postive
ALOX12	AL157871.2	0.537509185	1.11E-41 postive
GABPB1	AL157871.2	0.42318204	7.87E-25 postive
ATM	AL157871.2	0.502521599	7.83E-36 postive
FBXW7	AL157871.2	0.486513687	2.25E-33 postive
SLC1A4	AC007406.3	0.455807418	5.22E-29 postive
ZEB1	AC007406.3	0.561663523	3.88E-46 postive
EPAS1	AC007406.3	0.481973804	1.06E-32 postive
HELLS	AC046158.1	0.558512769	1.55E-45 postive
TUBE1	AC046158.1	0.421462405	1.27E-24 postive
ALOX12	AC046158.1	0.452200445	1.59E-28 postive
GABPB1	AC046158.1	0.513471672	1.37E-37 postive
ATG7	AC046158.1	0.412185911	1.60E-23 postive
MAPK8	AC046158.1	0.41399885	9.82E-24 postive
LINC00472	AC046158.1	0.637818768	6.81E-63 postive
ATM	AC046158.1	0.703489335	1.11E-81 postive
FBXW7	AC046158.1	0.454042875	9.03E-29 postive
HELLS	ACBD3-AS1	0.593424593	1.39E-52 postive
ZNF419	ACBD3-AS1	0.432953039	4.92E-26 postive
TUBE1	ACBD3-AS1	0.533347548	5.98E-41 postive
ALOX12	ACBD3-AS1	0.583442941	1.76E-50 postive
GABPB1	ACBD3-AS1	0.56188061	3.53E-46 postive
LINC00472	ACBD3-AS1	0.595154364	5.89E-53 postive
ATM	ACBD3-AS1	0.708693608	2.16E-83 postive
FBXW7	ACBD3-AS1	0.523205334	3.30E-39 postive
HELLS	AC008735.4	0.42237012	9.87E-25 postive
BRD4	AC008735.4	0.459535609	1.63E-29 postive
ZNF419	AC008735.4	0.618179647	3.96E-58 postive
TUBE1	AC008735.4	0.557289988	2.65E-45 postive
SETD1B	AC008735.4	0.561491583	4.19E-46 postive
ALOX12	AC008735.4	0.611633715	1.29E-56 postive
GABPB1	AC008735.4	0.46358447	4.50E-30 postive
LINC00472	AC008735.4	0.408243354	4.60E-23 postive
ATM	AC008735.4	0.481288018	1.34E-32 postive
TAZ	AC008735.4	0.492583828	2.72E-34 postive
FBXW7	AC008735.4	0.439558852	7.17E-27 postive
TFAP2C	TOB1-AS1	0.492749561	2.57E-34 postive
HBA1	TOB1-AS1	0.442915679	2.65E-27 postive
BRD4	AL928654.2	0.470386397	5.01E-31 postive
ZNF419	AL928654.2	0.662857417	1.71E-69 postive
VEGFA	AL928654.2	0.595133597	5.96E-53 postive
TUBE1	AL928654.2	0.560235827	7.29E-46 postive
SETD1B	AL928654.2	0.530968842	1.55E-40 postive
DRD4	AL928654.2	0.415186805	7.12E-24 postive
ALOX12	AL928654.2	0.662051618	2.85E-69 postive
TAZ	AL928654.2	0.594830112	6.92E-53 postive
FBXW7	AL928654.2	0.408019828	4.88E-23 postive
PTGS2	LIMS1-AS1	0.495383788	1.01E-34 postive
IL6	LIMS1-AS1	0.448985585	4.26E-28 postive
LINC00472	LIMS1-AS1	0.592024737	2.76E-52 postive
ATM	LIMS1-AS1	0.460671353	1.14E-29 postive

CISD1	AC103760.1	0.410259847	2.69E-23	postive
TFAP2C	AL139289.1	0.56218752	3.08E-46	postive
PHKG2	AC026333.4	0.582898018	2.28E-50	postive
TAZ	AC026333.4	0.601504053	2.43E-54	postive
ZNF419	AP001458.1	0.444018973	1.91E-27	postive
TUBE1	AP001458.1	0.455996153	4.93E-29	postive
SETD1B	AP001458.1	0.405546129	9.37E-23	postive
ALOX12	AP001458.1	0.574376676	1.24E-48	postive
GABPB1	AP001458.1	0.418449244	2.92E-24	postive
ATM	AP001458.1	0.422343857	9.94E-25	postive
FBXW7	AP001458.1	0.42814604	1.95E-25	postive
TFAP2C	AC018521.5	0.497894793	4.14E-35	postive
HBA1	AC018521.5	0.489712263	7.42E-34	postive
TAZ	LINC01474	0.442285135	3.20E-27	postive
G6PD	AP000525.1	0.4302365	1.07E-25	postive
TFR2	LINC01606	0.445630956	1.17E-27	postive
IDH1	LINC01606	0.506911222	1.57E-36	postive
ISCU	AC007342.5	0.457316225	3.26E-29	postive
KLHL24	AC007342.5	0.540260227	3.60E-42	postive
GABARAPL1	AC007342.5	0.541431166	2.22E-42	postive
LINC00472	AC007342.5	0.654854094	2.57E-67	postive
LPIN1	AC007342.5	0.550693193	4.55E-44	postive
TFAP2C	AC008543.3	0.575934622	6.02E-49	postive
HBA1	AC008543.3	0.515928773	5.40E-38	postive
DUOX1	AC008543.3	0.420194892	1.80E-24	postive
HELLS	AC002553.1	0.491160127	4.48E-34	postive
ZNF419	AC002553.1	0.544096099	7.34E-43	postive
VEGFA	AC002553.1	0.422852706	8.63E-25	postive
TUBE1	AC002553.1	0.676065575	3.06E-73	postive
SETD1B	AC002553.1	0.417439368	3.85E-24	postive
ALOX12	AC002553.1	0.703688099	9.58E-82	postive
GABPB1	AC002553.1	0.503355683	5.78E-36	postive
LINC00472	AC002553.1	0.485064527	3.70E-33	postive
ATM	AC002553.1	0.595297656	5.49E-53	postive
TAZ	AC002553.1	0.504671331	3.58E-36	postive
FBXW7	AC002553.1	0.492577514	2.73E-34	postive
ASNS	AC004825.2	0.402544033	2.06E-22	postive
JDP2	AC004825.2	0.601126897	2.94E-54	postive
RGS4	AC004825.2	0.454058861	8.98E-29	postive
EIF2S1	AC004825.2	0.472868958	2.22E-31	postive
CARS1	AC004825.2	0.407362956	5.80E-23	postive
HELLS	LINC01355	0.485317569	3.39E-33	postive
ZNF419	LINC01355	0.576813294	4.00E-49	postive
TUBE1	LINC01355	0.605198388	3.68E-55	postive
ALOX12	LINC01355	0.581605935	4.21E-50	postive
GABPB1	LINC01355	0.521844619	5.60E-39	postive
PHKG2	LINC01355	0.465553581	2.40E-30	postive
ATM	LINC01355	0.527474773	6.20E-40	postive
TAZ	LINC01355	0.614099146	3.51E-57	postive
FBXW7	LINC01355	0.504364384	4.00E-36	postive
HELLS	AL355488.1	0.514096767	1.08E-37	postive
ZNF419	AL355488.1	0.554841141	7.67E-45	postive
TUBE1	AL355488.1	0.664681166	5.32E-70	postive
ALOX12	AL355488.1	0.617533893	5.60E-58	postive
GABPB1	AL355488.1	0.519614268	1.32E-38	postive
PHKG2	AL355488.1	0.480946605	1.50E-32	postive
LINC00472	AL355488.1	0.425044775	4.67E-25	postive
ATM	AL355488.1	0.55268872	1.94E-44	postive

TAZ	AL355488.1	0.596402649	3.17E-53	postive
FBXW7	AL355488.1	0.4651154	2.76E-30	postive
FANCD2	AC011479.2	0.410407852	2.58E-23	postive
ALOX12	AC011479.2	0.546170913	3.08E-43	postive
TFAP2C	AC011479.2	0.413323194	1.18E-23	postive
HSPB1	AC087623.2	0.436563991	1.73E-26	postive
HRAS	AC087623.2	0.523980385	2.44E-39	postive
PHKG2	AC087623.2	0.438949784	8.58E-27	postive
EGLN2	AC087623.2	0.479592324	2.37E-32	postive
TAZ	AC087623.2	0.414714883	8.09E-24	postive
HELLS	AC027097.1	0.548527302	1.14E-43	postive
ZNF419	AC027097.1	0.417409549	3.88E-24	postive
TUBE1	AC027097.1	0.526183589	1.03E-39	postive
ALOX12	AC027097.1	0.546850598	2.32E-43	postive
GABPB1	AC027097.1	0.621582044	6.25E-59	postive
MAPK8	AC027097.1	0.489744509	7.34E-34	postive
LINC00472	AC027097.1	0.503629909	5.23E-36	postive
ATM	AC027097.1	0.68644188	2.53E-76	postive
FBXW7	AC027097.1	0.517778193	2.67E-38	postive
ZNF419	AL161669.3	0.425831035	3.74E-25	postive
VEGFA	AL161669.3	0.427990223	2.03E-25	postive
TUBE1	AL161669.3	0.427789403	2.15E-25	postive
ALOX12	AL161669.3	0.506081294	2.13E-36	postive
PHKG2	AL161669.3	0.476191613	7.39E-32	postive
TAZ	AL161669.3	0.677536553	1.14E-73	postive
FANCD2	AL355574.1	0.485194164	3.53E-33	postive
HELLS	AL355574.1	0.526269885	9.97E-40	postive
ZNF419	AL355574.1	0.47799846	4.05E-32	postive
TUBE1	AL355574.1	0.425813374	3.76E-25	postive
SETD1B	AL355574.1	0.421753378	1.17E-24	postive
ALOX12	AL355574.1	0.569228077	1.31E-47	postive
GABPB1	AL355574.1	0.452705033	1.36E-28	postive
ATM	AL355574.1	0.442232442	3.25E-27	postive
YY1AP1	AL355574.1	0.456188883	4.64E-29	postive
HELLS	AL157834.1	0.462077441	7.28E-30	postive
GABPB1	AL157834.1	0.434196062	3.43E-26	postive
ATG7	AL157834.1	0.402591724	2.03E-22	postive
LINC00472	AL157834.1	0.438747077	9.10E-27	postive
ATM	AL157834.1	0.629975428	5.98E-61	postive
FBXW7	AL157834.1	0.427204345	2.54E-25	postive
ZNF419	AC003070.1	0.525636122	1.28E-39	postive
VEGFA	AC003070.1	0.430255186	1.07E-25	postive
TUBE1	AC003070.1	0.602009213	1.88E-54	postive
SETD1B	AC003070.1	0.528050939	4.94E-40	postive
ALOX12	AC003070.1	0.68459304	9.15E-76	postive
PHKG2	AC003070.1	0.442783298	2.76E-27	postive
ATM	AC003070.1	0.44773002	6.24E-28	postive
TAZ	AC003070.1	0.596481363	3.04E-53	postive
FBXW7	AC003070.1	0.456039194	4.86E-29	postive
FANCD2	AC091180.4	0.412294957	1.56E-23	postive
TFAP2C	AC091180.4	0.557280815	2.66E-45	postive
HBA1	AC091180.4	0.505285786	2.86E-36	postive
HSPB1	AC007448.4	0.401263949	2.87E-22	postive
TFAP2C	AC007448.4	0.456886132	3.73E-29	postive
HBA1	AC007448.4	0.495193356	1.08E-34	postive
ALOX12B	AC007448.4	0.425828153	3.75E-25	postive
PHKG2	AL121601.1	0.406750915	6.82E-23	postive
TAZ	AL121601.1	0.511409385	2.96E-37	postive

HELLS	CR936218.1	0.559201666	1.15E-45	postive
ZNF419	CR936218.1	0.485769027	2.90E-33	postive
TUBE1	CR936218.1	0.598596008	1.06E-53	postive
ALOX12	CR936218.1	0.648289548	1.41E-65	postive
GABPB1	CR936218.1	0.585165692	7.71E-51	postive
MAPK8	CR936218.1	0.435754835	2.18E-26	postive
LINC00472	CR936218.1	0.618807784	2.82E-58	postive
ATM	CR936218.1	0.739332781	2.69E-94	postive
FBXW7	CR936218.1	0.534131738	4.36E-41	postive
FANCD2	CHROMR	0.459049424	1.89E-29	postive
HELLS	CHROMR	0.598749656	9.78E-54	postive
ZNF419	CHROMR	0.546633704	2.54E-43	postive
ZFP69B	CHROMR	0.407304002	5.89E-23	postive
TUBE1	CHROMR	0.538355069	7.85E-42	postive
ALOX12	CHROMR	0.487323804	1.70E-33	postive
GABPB1	CHROMR	0.690367482	1.59E-77	postive
ZEB1	CHROMR	0.452016662	1.69E-28	postive
MAPK8	CHROMR	0.445101268	1.38E-27	postive
ATM	CHROMR	0.551170255	3.71E-44	postive
FBXW7	CHROMR	0.582412206	2.87E-50	postive
CYBB	LINC00900	0.421395705	1.29E-24	postive
BACH1	LINC00900	0.405865886	8.62E-23	postive
TFAP2C	RB1-DT	0.560546084	6.36E-46	postive
HBA1	RB1-DT	0.515311304	6.82E-38	postive
DUOX1	RB1-DT	0.405360437	9.84E-23	postive
GOT1	AC026992.2	0.404368121	1.28E-22	postive
ZNF419	AL391684.1	0.601046795	3.07E-54	postive
TUBE1	AL391684.1	0.611685687	1.26E-56	postive
ALOX12	AL391684.1	0.666350692	1.82E-70	postive
GABPB1	AL391684.1	0.421781812	1.16E-24	postive
PHKG2	AL391684.1	0.455954918	4.99E-29	postive
ATM	AL391684.1	0.432374576	5.81E-26	postive
TAZ	AL391684.1	0.577568	2.81E-49	postive
HELLS	AL138921.2	0.454670066	7.44E-29	postive
ALOX12	AL138921.2	0.486689576	2.11E-33	postive
TFAP2C	AL138921.2	0.416966399	4.38E-24	postive
HBA1	AL138921.2	0.442761823	2.77E-27	postive
ATM	AL138921.2	0.402576239	2.04E-22	postive
FBXW7	AL138921.2	0.436395967	1.81E-26	postive
CA9	PFKP-DT	0.444422604	1.69E-27	postive
VEGFA	PFKP-DT	0.500228852	1.79E-35	postive
HELLS	SLC16A12-A	0.468880101	8.18E-31	postive
TUBE1	SLC16A12-A	0.404641231	1.19E-22	postive
ALOX12	SLC16A12-A	0.413505276	1.12E-23	postive
IREB2	SLC16A12-A	0.408251461	4.59E-23	postive
GABPB1	SLC16A12-A	0.478152678	3.84E-32	postive
MAPK8	SLC16A12-A	0.434099243	3.53E-26	postive
LINC00472	SLC16A12-A	0.573846871	1.58E-48	postive
PRKAA2	SLC16A12-A	0.432909838	4.98E-26	postive
ATM	SLC16A12-A	0.725786922	2.71E-89	postive
FBXW7	SLC16A12-A	0.413488679	1.13E-23	postive
LINC00472	AC080013.3	0.460357294	1.25E-29	postive
HELLS	AP003059.1	0.423634943	6.93E-25	postive
KLHL24	AP003059.1	0.560424182	6.71E-46	postive
IREB2	AP003059.1	0.496534064	6.73E-35	postive
GABPB1	AP003059.1	0.502752588	7.20E-36	postive
PIK3CA	AP003059.1	0.502199791	8.79E-36	postive
KRAS	AP003059.1	0.407503843	5.59E-23	postive

MAPK8	AP003059.1	0.412280055	1.56E-23	postive
LINC00472	AP003059.1	0.795503378	6.37E-119	postive
PRKAA1	AP003059.1	0.417500945	3.79E-24	postive
LPIN1	AP003059.1	0.451049223	2.27E-28	postive
ATM	AP003059.1	0.631537347	2.48E-61	postive
FBXW7	AP003059.1	0.445052443	1.40E-27	postive
HELLS	NEAT1	0.425193065	4.48E-25	postive
ZNF419	NEAT1	0.475566995	9.10E-32	postive
KLHL24	NEAT1	0.47164162	3.32E-31	postive
TUBE1	NEAT1	0.526632493	8.64E-40	postive
ALOX12	NEAT1	0.514057119	1.10E-37	postive
GABPB1	NEAT1	0.546527663	2.65E-43	postive
MAPK8	NEAT1	0.401102523	2.99E-22	postive
LINC00472	NEAT1	0.791176363	9.26E-117	postive
LPIN1	NEAT1	0.437963934	1.15E-26	postive
ATM	NEAT1	0.626198505	4.93E-60	postive
FBXW7	NEAT1	0.444326852	1.74E-27	postive
ALOX12	AC009159.3	0.422565243	9.35E-25	postive
PHKG2	AC009159.3	0.469988587	5.70E-31	postive
TAZ	AC009159.3	0.584676725	9.75E-51	postive
HELLS	AC116667.1	0.450193455	2.95E-28	postive
ZNF419	AC116667.1	0.620612067	1.06E-58	postive
TUBE1	AC116667.1	0.589209026	1.09E-51	postive
ALOX12	AC116667.1	0.639309585	2.86E-63	postive
GABPB1	AC116667.1	0.589327892	1.03E-51	postive
ATM	AC116667.1	0.47360133	1.74E-31	postive
TAZ	AC116667.1	0.43512333	2.62E-26	postive
FBXW7	AC116667.1	0.530059908	2.23E-40	postive
HELLS	AP002907.1	0.462382083	6.61E-30	postive
ZNF419	AP002907.1	0.493149879	2.23E-34	postive
KLHL24	AP002907.1	0.409061998	3.70E-23	postive
TUBE1	AP002907.1	0.538336823	7.91E-42	postive
ALOX12	AP002907.1	0.49610651	7.84E-35	postive
GABPB1	AP002907.1	0.567958133	2.33E-47	postive
LINC00472	AP002907.1	0.772918689	3.60E-108	postive
ATM	AP002907.1	0.588145604	1.83E-51	postive
FBXW7	AP002907.1	0.492406582	2.90E-34	postive
HELLS	OSGEPL1-AS	0.471206028	3.83E-31	postive
ZNF419	OSGEPL1-AS	0.485967982	2.71E-33	postive
TUBE1	OSGEPL1-AS	0.599132555	8.06E-54	postive
ALOX12	OSGEPL1-AS	0.583542955	1.68E-50	postive
GABPB1	OSGEPL1-AS	0.481863718	1.10E-32	postive
ATM	OSGEPL1-AS	0.573892994	1.55E-48	postive
EGLN2	OSGEPL1-AS	0.476233324	7.29E-32	postive
TAZ	OSGEPL1-AS	0.457793122	2.81E-29	postive
FBXW7	OSGEPL1-AS	0.504953809	3.22E-36	postive
BRD4	AC005785.1	0.412405102	1.51E-23	postive
ZNF419	AC005785.1	0.467468172	1.29E-30	postive
TUBE1	AC005785.1	0.421415108	1.29E-24	postive
DRD4	AC005785.1	0.411560398	1.90E-23	postive
ALOX12	AC005785.1	0.411639314	1.86E-23	postive
SCP2	AC005785.1	-0.403550488	1.58E-22	negative
NCOA4	AC005785.1	-0.517797327	2.65E-38	negative
PHKG2	AC005785.1	0.645708796	6.60E-65	postive
BECN1	AC005785.1	-0.425505989	4.10E-25	negative
MAPK1	AC005785.1	-0.412017525	1.68E-23	negative
TAZ	AC005785.1	0.754978677	1.76E-100	postive
KLHL24	AC234772.1	0.548497751	1.16E-43	postive

PIK3CA	AC234772.1	0.420764784	1.54E-24	postive
LINC00472	AC234772.1	0.874810301	3.81E-171	postive
ZNF419	AL353796.1	0.462978687	5.46E-30	postive
TUBE1	AL353796.1	0.534954982	3.13E-41	postive
ALOX12	AL353796.1	0.446191242	9.93E-28	postive
GABPB1	AL353796.1	0.474597792	1.25E-31	postive
TFAP2C	AC010761.6	0.545362862	4.32E-43	postive
HBA1	AC010761.6	0.456158446	4.68E-29	postive
IREB2	AL033381.1	0.435900218	2.09E-26	postive
PRKAA2	AL033381.1	0.491291648	4.28E-34	postive
ATM	AL033381.1	0.694355093	9.18E-79	postive
ZNF419	AC012313.6	0.571373045	4.93E-48	postive
VEGFA	AC012313.6	0.410551886	2.48E-23	postive
TUBE1	AC012313.6	0.518159085	2.31E-38	postive
SETD1B	AC012313.6	0.439626433	7.03E-27	postive
ALOX12	AC012313.6	0.63322619	9.50E-62	postive
ATM	AC012313.6	0.411235582	2.07E-23	postive
HSPB1	AL096828.3	0.453077903	1.22E-28	postive
HRAS	AL096828.3	0.423277523	7.66E-25	postive
PHKG2	AL096828.3	0.454439688	7.99E-29	postive
PRKAA2	AL589745.1	0.471331422	3.68E-31	postive
TUBE1	AC074124.1	0.479035005	2.86E-32	postive
ALOX12	AC074124.1	0.432872848	5.03E-26	postive
ATM	AC074124.1	0.42658507	3.03E-25	postive
ENPP2	LINC00987	0.470472104	4.87E-31	postive
SLC1A4	LINC00987	0.4555745	5.62E-29	postive
ATP5MC3	LINC00987	-0.421685317	1.19E-24	negative
IL33	LINC00987	0.466624252	1.70E-30	postive
HMGB1	LINC00987	0.425284151	4.37E-25	postive
ZEB1	LINC00987	0.576848504	3.93E-49	postive
EPAS1	LINC00987	0.528726038	3.78E-40	postive
TFAP2C	AC087501.4	0.512161937	2.23E-37	postive
HBA1	AC087501.4	0.47291966	2.18E-31	postive
TUBE1	AC109460.1	0.406554835	7.18E-23	postive
SETD1B	AC109460.1	0.48210305	1.01E-32	postive
ALOX12	AC109460.1	0.593703713	1.21E-52	postive
TAZ	AC109460.1	0.427811908	2.14E-25	postive
FANCD2	LINC02481	0.437868886	1.18E-26	postive
ALOX12	LINC02481	0.408778961	3.99E-23	postive
TFAP2C	LINC02481	0.481276935	1.34E-32	postive
HBA1	LINC02481	0.448063322	5.64E-28	postive
HELLS	AL136985.2	0.480763931	1.60E-32	postive
ZNF419	AL136985.2	0.418730807	2.70E-24	postive
TUBE1	AL136985.2	0.429527905	1.31E-25	postive
ALOX12	AL136985.2	0.490263097	6.13E-34	postive
GABPB1	AL136985.2	0.492209961	3.10E-34	postive
LINC00472	AL136985.2	0.524704981	1.84E-39	postive
ATM	AL136985.2	0.612075266	1.02E-56	postive
FBXW7	AL136985.2	0.439171442	8.03E-27	postive
GABPB1	LINC01752	0.413858057	1.02E-23	postive
ATM	LINC01752	0.425642497	3.95E-25	postive
VEGFA	AP001830.1	0.538093199	8.74E-42	postive
ALOX12	AP001830.1	0.407813522	5.15E-23	postive
TAZ	AP001830.1	0.4677111	1.20E-30	postive
FANCD2	WNT5A-AS1	0.495688788	9.09E-35	postive
TFAP2C	WNT5A-AS1	0.457878053	2.74E-29	postive
GABPB1	AC120349.1	0.472376726	2.61E-31	postive
LINC00472	AC120349.1	0.695520907	3.95E-79	postive

ZNF419	AL031658.1	0.49541699	1.00E-34	postive
VEGFA	AL031658.1	0.502633995	7.51E-36	postive
TUBE1	AL031658.1	0.438422128	1.00E-26	postive
SETD1B	AL031658.1	0.429163322	1.46E-25	postive
ALOX12	AL031658.1	0.533800315	4.99E-41	postive
ZEB1	AL031658.1	0.413060143	1.27E-23	postive
PTGS2	AL391056.1	0.525909176	1.15E-39	postive
IL6	AL391056.1	0.475234578	1.02E-31	postive
SLC2A3	AL391056.1	0.405750129	8.88E-23	postive
GPX4	AC008915.2	0.499258792	2.54E-35	postive
HSPB1	AC008915.2	0.457154439	3.43E-29	postive
OTUB1	AC008915.2	0.469086156	7.65E-31	postive
HBA1	AC008915.2	0.430477242	1.00E-25	postive
KRAS	AC008915.2	-0.41607841	5.58E-24	negative
HRAS	AC008915.2	0.435577077	2.30E-26	postive
PHKG2	AC008915.2	0.433163876	4.63E-26	postive
PRKAA1	AC008915.2	-0.415070565	7.35E-24	negative
SIRT1	AC008915.2	-0.432980593	4.88E-26	negative
HIC1	RARA-AS1	0.433937487	3.70E-26	postive
HELLS	AC008906.1	0.540555295	3.18E-42	postive
TUBE1	AC008906.1	0.497159317	5.39E-35	postive
ALOX12	AC008906.1	0.440393244	5.60E-27	postive
GABPB1	AC008906.1	0.530202699	2.11E-40	postive
ATG7	AC008906.1	0.408497175	4.30E-23	postive
LINC00472	AC008906.1	0.649188166	8.18E-66	postive
ATM	AC008906.1	0.740997147	6.21E-95	postive
FBXW7	AC008906.1	0.499196699	2.60E-35	postive
ZNF419	AC009336.1	0.532813626	7.42E-41	postive
VEGFA	AC009336.1	0.485265734	3.45E-33	postive
TUBE1	AC009336.1	0.484444222	4.57E-33	postive
SETD1B	AC009336.1	0.435918782	2.08E-26	postive
ALOX12	AC009336.1	0.507651078	1.20E-36	postive
GABPB1	AC009336.1	0.471271384	3.75E-31	postive
ATM	AC009336.1	0.535000881	3.07E-41	postive
FBXW7	AC009336.1	0.485299649	3.41E-33	postive
HELLS	AC093620.1	0.402221985	2.24E-22	postive
ZNF419	AC093620.1	0.463078068	5.29E-30	postive
KLHL24	AC093620.1	0.427498141	2.34E-25	postive
GABPB1	AC093620.1	0.496373019	7.13E-35	postive
LINC00472	AC093620.1	0.648380377	1.33E-65	postive
LPIN1	AC093620.1	0.439042707	8.34E-27	postive
ATM	AC093620.1	0.504755475	3.47E-36	postive
FBXW7	AC093620.1	0.429528416	1.31E-25	postive
KLHL24	C1orf195	0.588455899	1.58E-51	postive
GABPB1	C1orf195	0.419469274	2.20E-24	postive
PIK3CA	C1orf195	0.473024181	2.11E-31	postive
KRAS	C1orf195	0.411583221	1.88E-23	postive
LINC00472	C1orf195	0.953431848	1.04E-281	postive
PML	AC145098.1	0.472513439	2.50E-31	postive
NCF2	AC145098.1	0.495828746	8.65E-35	postive
CYBB	AC145098.1	0.48865851	1.07E-33	postive
HELLS	AC025034.1	0.523252654	3.24E-39	postive
ZNF419	AC025034.1	0.489983065	6.76E-34	postive
TUBE1	AC025034.1	0.562859934	2.28E-46	postive
ALOX12	AC025034.1	0.546058049	3.23E-43	postive
GABPB1	AC025034.1	0.55179249	2.85E-44	postive
LINC00472	AC025034.1	0.631807933	2.13E-61	postive
ATM	AC025034.1	0.574834541	1.00E-48	postive

FBXW7	AC025034.1	0.511981869	2.39E-37	postive
PRKAA2	LINC01135	0.414674095	8.18E-24	postive
ATM	LINC01135	0.479894829	2.14E-32	postive
ACSF2	CEBPA-DT	0.476843339	5.95E-32	postive
HELLS	SLC2A9-AS1	0.528346958	4.40E-40	postive
TUBE1	SLC2A9-AS1	0.421214964	1.36E-24	postive
ALOX12	SLC2A9-AS1	0.423001412	8.28E-25	postive
GABPB1	SLC2A9-AS1	0.484400967	4.64E-33	postive
LINC00472	SLC2A9-AS1	0.434799635	2.88E-26	postive
ATM	SLC2A9-AS1	0.649723751	5.92E-66	postive
FBXW7	SLC2A9-AS1	0.416748601	4.65E-24	postive
FANCD2	MZF1-AS1	0.492944985	2.40E-34	postive
HELLS	MZF1-AS1	0.419154	2.40E-24	postive
ZNF419	MZF1-AS1	0.50260571	7.59E-36	postive
ALOX12	MZF1-AS1	0.548086767	1.38E-43	postive
TFAP2C	MZF1-AS1	0.45270867	1.36E-28	postive
HELLS	AC009542.1	0.489269027	8.66E-34	postive
TUBE1	AC009542.1	0.454930225	6.86E-29	postive
ALOX12	AC009542.1	0.446507439	9.02E-28	postive
GABPB1	AC009542.1	0.445154338	1.36E-27	postive
LINC00472	AC009542.1	0.463457528	4.69E-30	postive
ATM	AC009542.1	0.665165993	3.90E-70	postive
FBXW7	AC009542.1	0.429891777	1.18E-25	postive
HSF1	AC027307.2	0.443351479	2.33E-27	postive
NCOA4	AC027307.2	-0.429417963	1.36E-25	negative
TLR4	AC027307.2	-0.409926548	2.94E-23	negative
ATF4	MIR210HG	0.402991164	1.83E-22	postive
CA9	MIR210HG	0.511625632	2.73E-37	postive
VEGFA	MIR210HG	0.53253073	8.31E-41	postive
DRD4	MIR210HG	0.410221989	2.71E-23	postive
EIF2AK4	MIR210HG	-0.413287829	1.19E-23	negative
ALOX12	MIR210HG	0.445592083	1.19E-27	postive
PHKG2	MIR210HG	0.440420508	5.56E-27	postive
TAZ	MIR210HG	0.627929991	1.88E-60	postive
TUBE1	LINC00861	0.470965968	4.15E-31	postive
ALOX12	LINC00861	0.525577742	1.31E-39	postive
GABPB1	LINC00861	0.441344083	4.23E-27	postive
IFNG	LINC00861	0.432476417	5.64E-26	postive
ATM	LINC00861	0.525149511	1.55E-39	postive
FBXW7	LINC00861	0.486758728	2.06E-33	postive
HELLS	AC025430.1	0.579465599	1.16E-49	postive
ZNF419	AC025430.1	0.400083784	3.89E-22	postive
TUBE1	AC025430.1	0.43842366	1.00E-26	postive
ALOX12	AC025430.1	0.52207433	5.12E-39	postive
GABPB1	AC025430.1	0.545020609	4.99E-43	postive
ATG7	AC025430.1	0.410888464	2.27E-23	postive
LINC00472	AC025430.1	0.63412652	5.69E-62	postive
ATM	AC025430.1	0.66913363	2.99E-71	postive
FBXW7	AC025430.1	0.502372723	8.26E-36	postive
HELLS	AC004492.1	0.528294872	4.49E-40	postive
ZNF419	AC004492.1	0.412234231	1.58E-23	postive
KLHL24	AC004492.1	0.490178855	6.31E-34	postive
TUBE1	AC004492.1	0.545785643	3.62E-43	postive
MAP3K5	AC004492.1	0.410003832	2.88E-23	postive
ALOX12	AC004492.1	0.509004403	7.25E-37	postive
IREB2	AC004492.1	0.489237929	8.76E-34	postive
GABPB1	AC004492.1	0.602695671	1.33E-54	postive
PIK3CA	AC004492.1	0.472283353	2.69E-31	postive

MAPK8	AC004492.1	0.487173363	1.79E-33	postive
LINC00472	AC004492.1	0.773606781	1.76E-108	postive
PRKAA1	AC004492.1	0.422090291	1.07E-24	postive
ATM	AC004492.1	0.791494555	6.44E-117	postive
FBXW7	AC004492.1	0.528145963	4.76E-40	postive
LINC00472	ACTA2-AS1	0.498031817	3.94E-35	postive
CISD2	TAF1A-AS1	0.427567853	2.29E-25	postive
FANCD2	CDK6-AS1	0.600917599	3.27E-54	postive
HELLS	CDK6-AS1	0.410537448	2.49E-23	postive
STMN1	CDK6-AS1	0.750378578	1.29E-98	postive
RRM2	CDK6-AS1	0.583260073	1.92E-50	postive
AURKA	CDK6-AS1	0.600839779	3.40E-54	postive
CDKN2A	CDK6-AS1	0.689426732	3.10E-77	postive
HBA1	AC104667.2	0.546323995	2.89E-43	postive
HELLS	POC1B-AS1	0.532531886	8.30E-41	postive
ZNF419	POC1B-AS1	0.506301799	1.97E-36	postive
TUBE1	POC1B-AS1	0.553008071	1.69E-44	postive
ALOX12	POC1B-AS1	0.555839235	4.98E-45	postive
GABPB1	POC1B-AS1	0.591993755	2.81E-52	postive
MAPK8	POC1B-AS1	0.414851331	7.80E-24	postive
LINC00472	POC1B-AS1	0.694196709	1.03E-78	postive
ATM	POC1B-AS1	0.744532155	2.66E-96	postive
FBXW7	POC1B-AS1	0.563460145	1.75E-46	postive
HELLS	AC025171.4	0.424316809	5.73E-25	postive
ZNF419	AC025171.4	0.460626954	1.15E-29	postive
TUBE1	AC025171.4	0.492568678	2.74E-34	postive
ALOX12	AC025171.4	0.483928645	5.45E-33	postive
PHKG2	AC025171.4	0.473526432	1.79E-31	postive
TAZ	AC025171.4	0.567151242	3.35E-47	postive
HELLS	AC105389.2	0.421141236	1.39E-24	postive
MTOR	AC105389.2	0.413386476	1.16E-23	postive
KLHL24	AC105389.2	0.59855759	1.08E-53	postive
TUBE1	AC105389.2	0.419640823	2.10E-24	postive
MAP3K5	AC105389.2	0.410393404	2.59E-23	postive
IREB2	AC105389.2	0.529103746	3.26E-40	postive
GABPB1	AC105389.2	0.566094041	5.39E-47	postive
PIK3CA	AC105389.2	0.542060771	1.71E-42	postive
KRAS	AC105389.2	0.464726855	3.12E-30	postive
ATG7	AC105389.2	0.420991956	1.45E-24	postive
MAPK8	AC105389.2	0.468501779	9.25E-31	postive
LINC00472	AC105389.2	0.877185824	3.11E-173	postive
PRKAA1	AC105389.2	0.466102569	2.01E-30	postive
TGFBR1	AC105389.2	0.408620884	4.16E-23	postive
LPIN1	AC105389.2	0.469951626	5.77E-31	postive
ATM	AC105389.2	0.670767308	1.03E-71	postive
FBXW7	AC105389.2	0.474797609	1.17E-31	postive
TFAP2C	AL354872.1	0.457862794	2.75E-29	postive
HBA1	AL354872.1	0.41866204	2.75E-24	postive
FANCD2	MYG1-AS1	0.401633627	2.61E-22	postive
SCP2	MYG1-AS1	-0.414697925	8.13E-24	negative
NCOA4	MYG1-AS1	-0.438527247	9.71E-27	negative
PHKG2	MYG1-AS1	0.57176885	4.11E-48	postive
MAPK1	MYG1-AS1	-0.416923923	4.43E-24	negative
TAZ	MYG1-AS1	0.598572076	1.07E-53	postive
ALOX12	ID2-AS1	0.459696571	1.55E-29	postive
ATF4	GAS5	0.438211302	1.07E-26	postive
RPL8	GAS5	0.593902379	1.10E-52	postive
TFAP2C	BMPR1B-DT	0.551108413	3.81E-44	postive

HBA1	BMPR1B-DT	0.452102564	1.64E-28	postive
SLC2A12	AL049838.1	0.453246979	1.15E-28	postive
WIPI2	AL049838.1	0.405876489	8.59E-23	postive
LPIN1	AL049838.1	0.41531749	6.87E-24	postive
ZNF419	AL583856.2	0.410965636	2.22E-23	postive
TUBE1	AL583856.2	0.449448403	3.70E-28	postive
ALOX12	AL583856.2	0.472212774	2.76E-31	postive
TFAP2C	AL583856.2	0.419434633	2.23E-24	postive
BRD4	CTBP1-AS	0.47374715	1.66E-31	postive
ZNF419	CTBP1-AS	0.589908407	7.79E-52	postive
VEGFA	CTBP1-AS	0.490686408	5.29E-34	postive
TUBE1	CTBP1-AS	0.582733693	2.46E-50	postive
SETD1B	CTBP1-AS	0.600567217	3.91E-54	postive
ALOX12	CTBP1-AS	0.687776207	9.92E-77	postive
GABPB1	CTBP1-AS	0.452333059	1.53E-28	postive
ATM	CTBP1-AS	0.48726816	1.73E-33	postive
TAZ	CTBP1-AS	0.506757706	1.66E-36	postive
FBXW7	CTBP1-AS	0.47922596	2.68E-32	postive
ZNF419	AC012254.1	0.423278593	7.66E-25	postive
TUBE1	AC012254.1	0.491836685	3.54E-34	postive
ALOX12	AC012254.1	0.428630325	1.70E-25	postive
GABPB1	AC012254.1	0.523263141	3.23E-39	postive
MAPK8	AC012254.1	0.422764129	8.84E-25	postive
LINC00472	AC012254.1	0.627801325	2.02E-60	postive
ATM	AC012254.1	0.650499223	3.70E-66	postive
FBXW7	AC012254.1	0.541412294	2.24E-42	postive
HELLS	LINC02449	0.476706354	6.23E-32	postive
ZNF419	LINC02449	0.453024378	1.24E-28	postive
SETD1B	LINC02449	0.416356516	5.18E-24	postive
ALOX12	LINC02449	0.442541627	2.96E-27	postive
ATM	LINC02449	0.400894903	3.16E-22	postive
PCK2	PCAT7	0.40344155	1.63E-22	postive
HELLS	ZNF460-AS1	0.43289391	5.00E-26	postive
ZNF419	ZNF460-AS1	0.63526998	2.96E-62	postive
TUBE1	ZNF460-AS1	0.510339322	4.42E-37	postive
ALOX12	ZNF460-AS1	0.562756372	2.39E-46	postive
GABPB1	ZNF460-AS1	0.471668673	3.29E-31	postive
PHKG2	ZNF460-AS1	0.434439821	3.20E-26	postive
LINC00472	ZNF460-AS1	0.476023662	7.82E-32	postive
LPIN1	ZNF460-AS1	0.450177105	2.96E-28	postive
ATM	ZNF460-AS1	0.415491419	6.55E-24	postive
TAZ	ZNF460-AS1	0.499528504	2.31E-35	postive
FBXW7	ZNF460-AS1	0.444357706	1.72E-27	postive
NRAS	AC020910.5	-0.442905563	2.66E-27	negative
NCOA4	AC020910.5	-0.409566357	3.23E-23	negative
PHKG2	AC020910.5	0.508385379	9.12E-37	postive
MAPK1	AC020910.5	-0.431183855	8.18E-26	negative
TAZ	AC020910.5	0.523473799	2.97E-39	postive
FANCD2	AC079922.2	0.588498672	1.55E-51	postive
ASNS	AC079922.2	0.423648294	6.91E-25	postive
SLC2A6	AC079922.2	0.487697252	1.49E-33	postive
RRM2	AC079922.2	0.408624273	4.15E-23	postive
AURKA	AC079922.2	0.437812532	1.20E-26	postive
CARS1	AC079922.2	0.403704466	1.52E-22	postive
PHKG2	AC079922.2	0.513268901	1.47E-37	postive
EGLN2	AC079922.2	0.479218596	2.69E-32	postive
TAZ	AC079922.2	0.565780539	6.20E-47	postive
RGS4	AC016405.2	0.677983113	8.42E-74	postive

BRD4	AC005519.1	0.424235724	5.86E-25	postive
ZNF419	AC005519.1	0.623344961	2.38E-59	postive
VEGFA	AC005519.1	0.531912814	1.06E-40	postive
TUBE1	AC005519.1	0.654178713	3.90E-67	postive
SETD1B	AC005519.1	0.547529805	1.74E-43	postive
ALOX12	AC005519.1	0.789618983	5.40E-116	postive
GABPB1	AC005519.1	0.454943544	6.83E-29	postive
ATM	AC005519.1	0.557152529	2.81E-45	postive
YY1AP1	AC005519.1	0.404934245	1.10E-22	postive
TAZ	AC005519.1	0.49399992	1.65E-34	postive
FBXW7	AC005519.1	0.445347953	1.28E-27	postive
LINC00472	AC139100.1	0.478523759	3.39E-32	postive
HELLS	AL162724.1	0.545360465	4.33E-43	postive
TUBE1	AL162724.1	0.446314885	9.56E-28	postive
ALOX12	AL162724.1	0.481254767	1.35E-32	postive
GABPB1	AL162724.1	0.489790256	7.23E-34	postive
ATG7	AL162724.1	0.42388459	6.47E-25	postive
LINC00472	AL162724.1	0.486953798	1.93E-33	postive
ATM	AL162724.1	0.685461138	5.00E-76	postive
FBXW7	AL162724.1	0.442768012	2.77E-27	postive
RPL8	TIPARP-AS1	0.454362391	8.18E-29	postive
ALOX12	AL359921.2	0.501751563	1.03E-35	postive
NCOA4	AL359921.2	-0.460947291	1.04E-29	negative
PHKG2	AL359921.2	0.438553001	9.64E-27	postive
TAZ	AL359921.2	0.603504429	8.77E-55	postive
HELLS	AL161725.1	0.529176787	3.16E-40	postive
KLHL24	AL161725.1	0.423793259	6.63E-25	postive
TUBE1	AL161725.1	0.565028708	8.69E-47	postive
ALOX12	AL161725.1	0.573943855	1.51E-48	postive
IREB2	AL161725.1	0.486298294	2.42E-33	postive
GABPB1	AL161725.1	0.566275232	4.97E-47	postive
PIK3CA	AL161725.1	0.426343699	3.24E-25	postive
ATG7	AL161725.1	0.429897733	1.18E-25	postive
MAPK8	AL161725.1	0.478919688	2.97E-32	postive
LINC00472	AL161725.1	0.723741472	1.45E-88	postive
ATM	AL161725.1	0.781723389	3.29E-112	postive
FBXW7	AL161725.1	0.497867199	4.18E-35	postive
KLHL24	AC010300.1	0.510254962	4.56E-37	postive
GABPB1	AC010300.1	0.407781928	5.19E-23	postive
LINC00472	AC010300.1	0.885943178	2.54E-181	postive
LPIN1	AC010300.1	0.424724893	5.11E-25	postive
TFAP2C	AC092756.1	0.584657962	9.84E-51	postive
HBA1	AC092756.1	0.515466638	6.43E-38	postive
DUOX1	AC092756.1	0.425531652	4.07E-25	postive
PHKG2	AL162258.2	0.480723719	1.62E-32	postive
HELLS	AC007376.2	0.40895587	3.80E-23	postive
MTOR	AC007376.2	0.428809833	1.61E-25	postive
TUBE1	AC007376.2	0.425949945	3.62E-25	postive
IREB2	AC007376.2	0.448329303	5.20E-28	postive
GABPB1	AC007376.2	0.612152923	9.83E-57	postive
PIK3CA	AC007376.2	0.464837882	3.02E-30	postive
ATG7	AC007376.2	0.454660035	7.46E-29	postive
ZEB1	AC007376.2	0.557264433	2.68E-45	postive
MAPK8	AC007376.2	0.50120892	1.26E-35	postive
LINC00472	AC007376.2	0.582666222	2.54E-50	postive
TLR4	AC007376.2	0.427958608	2.05E-25	postive
ATM	AC007376.2	0.69430246	9.53E-79	postive
FBXW7	AC007376.2	0.540301988	3.53E-42	postive

HELLS	AC106795.2	0.409955853	2.91E-23	postive
ZNF419	AC106795.2	0.431260966	8.00E-26	postive
TUBE1	AC106795.2	0.466130261	1.99E-30	postive
ALOX12	AC106795.2	0.482430762	9.08E-33	postive
PHKG2	AC106795.2	0.445625055	1.18E-27	postive
TAZ	AC106795.2	0.469355734	7.01E-31	postive
HELLS	AC115989.1	0.496054824	7.98E-35	postive
ALOX12	AC115989.1	0.463838477	4.15E-30	postive
TFAP2C	AC115989.1	0.417216945	4.09E-24	postive
DUOX1	AC115989.1	0.441788274	3.71E-27	postive
LINC00472	AC115989.1	0.404661854	1.18E-22	postive
ATM	AC115989.1	0.467787127	1.17E-30	postive
HSPB1	AP001363.2	0.417148439	4.17E-24	postive
RPL8	AP001363.2	0.447655899	6.38E-28	postive
HBA1	AP001363.2	0.568028961	2.25E-47	postive
HRAS	AP001363.2	0.744617421	2.46E-96	postive
MAP1LC3A	AP001363.2	0.447451735	6.78E-28	postive
EGLN2	AP001363.2	0.75180547	3.45E-99	postive
ALOX12	GAS6-AS1	0.494909182	1.20E-34	postive
TAZ	GAS6-AS1	0.446752609	8.38E-28	postive
GPX4	AC073896.2	0.49439331	1.44E-34	postive
STAT3	AC073896.2	-0.439015663	8.41E-27	negative
ISCU	AC073896.2	0.444792977	1.51E-27	postive
OTUB1	AC073896.2	0.436526446	1.74E-26	postive
SLC2A8	AC073896.2	0.424089752	6.11E-25	postive
PHKG2	AC073896.2	0.412748114	1.38E-23	postive
ATG4D	AC073896.2	0.554962573	7.28E-45	postive
MAP1LC3A	AC073896.2	0.465022979	2.84E-30	postive
GABARAPL2	AC073896.2	0.425988116	3.58E-25	postive
SIRT1	AC073896.2	-0.412868059	1.33E-23	negative
HELLS	AL353593.2	0.475867582	8.24E-32	postive
TUBE1	AL353593.2	0.456196824	4.63E-29	postive
ALOX12	AL353593.2	0.496195738	7.59E-35	postive
GABPB1	AL353593.2	0.449635497	3.49E-28	postive
LINC00472	AL353593.2	0.458887725	1.99E-29	postive
ATM	AL353593.2	0.687330204	1.36E-76	postive
FBXW7	AL353593.2	0.430172813	1.09E-25	postive
PML	PCED1B-AS1	0.497324053	5.08E-35	postive
GCH1	PCED1B-AS1	0.448391649	5.10E-28	postive
HAMP	PCED1B-AS1	0.4458542	1.10E-27	postive
SLC2A6	PCED1B-AS1	0.489796282	7.21E-34	postive
CYBB	PCED1B-AS1	0.40126147	2.87E-22	postive
IFNG	PCED1B-AS1	0.671715816	5.51E-72	postive
ISCU	FOXP4-AS1	0.431604399	7.25E-26	postive
TFAP2C	FOXP4-AS1	0.409056661	3.70E-23	postive
GABARAPL2	FOXP4-AS1	0.414037185	9.72E-24	postive
GABARAPL1	FOXP4-AS1	0.409608003	3.20E-23	postive
HELLS	AC026124.2	0.464515108	3.34E-30	postive
KLHL24	AC026124.2	0.431636927	7.18E-26	postive
TUBE1	AC026124.2	0.412291036	1.56E-23	postive
GABPB1	AC026124.2	0.552923625	1.75E-44	postive
PIK3CA	AC026124.2	0.416718923	4.69E-24	postive
MAPK8	AC026124.2	0.452915636	1.28E-28	postive
LINC00472	AC026124.2	0.836514085	2.07E-142	postive
ATM	AC026124.2	0.66176616	3.41E-69	postive
FBXW7	AC026124.2	0.434917098	2.79E-26	postive
KLHL24	MID1IP1-AS1	0.400872115	3.17E-22	postive
CS	MID1IP1-AS1	0.467503144	1.28E-30	postive

GABARAPL1	MID1IP1-AS1	0.524624415	1.90E-39	postive
LPIN1	MID1IP1-AS1	0.423314741	7.58E-25	postive
MTOR	MAGI2-AS3	0.405543444	9.38E-23	postive
ACSL3	MAGI2-AS3	0.41731216	3.99E-24	postive
HIF1A	MAGI2-AS3	0.447953683	5.83E-28	postive
KLHL24	MAGI2-AS3	0.413421401	1.15E-23	postive
IREB2	MAGI2-AS3	0.467787727	1.17E-30	postive
GABPB1	MAGI2-AS3	0.507854602	1.11E-36	postive
PIK3CA	MAGI2-AS3	0.473535555	1.78E-31	postive
KRAS	MAGI2-AS3	0.411362888	2.00E-23	postive
ZEB1	MAGI2-AS3	0.403658749	1.54E-22	postive
MAPK8	MAGI2-AS3	0.469412278	6.88E-31	postive
LINC00472	MAGI2-AS3	0.417385304	3.91E-24	postive
PRKAA1	MAGI2-AS3	0.423765592	6.69E-25	postive
LPIN1	MAGI2-AS3	0.411794122	1.78E-23	postive
HELLS	AC130895.1	0.465133943	2.74E-30	postive
KLHL24	AC130895.1	0.477719128	4.44E-32	postive
IREB2	AC130895.1	0.424575035	5.33E-25	postive
GABPB1	AC130895.1	0.541297956	2.34E-42	postive
PIK3CA	AC130895.1	0.449889641	3.23E-28	postive
ATG7	AC130895.1	0.427369615	2.43E-25	postive
MAPK8	AC130895.1	0.401084632	3.00E-22	postive
LINC00472	AC130895.1	0.871576586	2.26E-168	postive
ATM	AC130895.1	0.647373035	2.44E-65	postive
FBXW7	AC130895.1	0.406038442	8.23E-23	postive
KLHL24	AC107398.2	0.627567877	2.30E-60	postive
IREB2	AC107398.2	0.403111302	1.77E-22	postive
GABPB1	AC107398.2	0.410200962	2.73E-23	postive
PIK3CA	AC107398.2	0.489504882	7.98E-34	postive
KRAS	AC107398.2	0.432341317	5.87E-26	postive
LINC00472	AC107398.2	0.945610097	4.56E-264	postive
LPIN1	AC107398.2	0.427936936	2.06E-25	postive
MTOR	AC012313.5	0.52996152	2.32E-40	postive
OXSR1	AC012313.5	0.412371628	1.52E-23	postive
ZNF419	AC012313.5	0.533732096	5.13E-41	postive
IREB2	AC012313.5	0.482913302	7.70E-33	postive
GABPB1	AC012313.5	0.455346409	6.03E-29	postive
MAPK8	AC012313.5	0.49599917	8.14E-35	postive
LINC00472	AC012313.5	0.416559544	4.90E-24	postive
ATM	AC012313.5	0.533457492	5.73E-41	postive
HELLS	SEC62-AS1	0.519642203	1.31E-38	postive
ZNF419	SEC62-AS1	0.410544088	2.49E-23	postive
TUBE1	SEC62-AS1	0.554852013	7.63E-45	postive
ALOX12	SEC62-AS1	0.544735515	5.62E-43	postive
IREB2	SEC62-AS1	0.400482325	3.51E-22	postive
GABPB1	SEC62-AS1	0.5452729	4.49E-43	postive
ATG7	SEC62-AS1	0.407955225	4.96E-23	postive
MAPK8	SEC62-AS1	0.441312867	4.27E-27	postive
LINC00472	SEC62-AS1	0.549491162	7.59E-44	postive
ATM	SEC62-AS1	0.770189816	5.90E-107	postive
FBXW7	SEC62-AS1	0.515099762	7.39E-38	postive
HELLS	AL133230.1	0.510738737	3.81E-37	postive
TUBE1	AL133230.1	0.456259593	4.54E-29	postive
ALOX12	AL133230.1	0.492199751	3.11E-34	postive
GABPB1	AL133230.1	0.508835688	7.72E-37	postive
LINC00472	AL133230.1	0.578869659	1.53E-49	postive
ATM	AL133230.1	0.714820734	1.86E-85	postive
FBXW7	AL133230.1	0.499233174	2.56E-35	postive

GPX4	AL121832.2	0.422960376	8.37E-25 postive
HSPB1	AL121832.2	0.52544569	1.38E-39 postive
OTUB1	AL121832.2	0.401756474	2.52E-22 postive
HRAS	AL121832.2	0.700796246	8.27E-81 postive
NCOA4	AL121832.2	-0.443889913	1.98E-27 negative
PHKG2	AL121832.2	0.558683439	1.44E-45 postive
MAP1LC3A	AL121832.2	0.473528342	1.79E-31 postive
ANO6	AL121832.2	-0.416809558	4.57E-24 negative
EGLN2	AL121832.2	0.645450959	7.70E-65 postive
TAZ	AL121832.2	0.559809193	8.80E-46 postive
CD44	AL355916.1	0.492703727	2.61E-34 postive
LAMP2	AL355916.1	0.432836275	5.09E-26 postive
HELLS	AL512652.1	0.44326151	2.39E-27 postive
ZNF419	AL512652.1	0.513541946	1.33E-37 postive
TUBE1	AL512652.1	0.538749548	6.68E-42 postive
ALOX12	AL512652.1	0.528368134	4.36E-40 postive
GABPB1	AL512652.1	0.456859267	3.76E-29 postive
PHKG2	AL512652.1	0.489290912	8.60E-34 postive
LINC00472	AL512652.1	0.405180041	1.03E-22 postive
ATM	AL512652.1	0.459193048	1.81E-29 postive
TAZ	AL512652.1	0.566918163	3.72E-47 postive
FBXW7	AL512652.1	0.42786228	2.11E-25 postive
HELLS	PLCG1-AS1	0.52945124	2.84E-40 postive
ZNF419	PLCG1-AS1	0.441674405	3.83E-27 postive
TUBE1	PLCG1-AS1	0.520766535	8.49E-39 postive
ALOX12	PLCG1-AS1	0.538766359	6.64E-42 postive
GABPB1	PLCG1-AS1	0.557665888	2.25E-45 postive
LINC00472	PLCG1-AS1	0.489207988	8.85E-34 postive
ATM	PLCG1-AS1	0.623527777	2.15E-59 postive
FBXW7	PLCG1-AS1	0.50865084	8.27E-37 postive
TUBE1	AC009159.2	0.577797108	2.53E-49 postive
ALOX12	AC009159.2	0.568217087	2.07E-47 postive
GABPB1	AC009159.2	0.424305809	5.75E-25 postive
LINC00472	AC009159.2	0.45301982	1.24E-28 postive
ATM	AC009159.2	0.612815938	6.93E-57 postive
ISCU	AC138305.1	0.586751001	3.60E-51 postive
ACSL3	AC138305.1	0.501985884	9.50E-36 postive
VLDLR	AC138305.1	0.415992655	5.72E-24 postive
KLHL24	AC138305.1	0.6381611	5.58E-63 postive
ATG4D	AC138305.1	0.430390638	1.03E-25 postive
GABARAPL1	AC138305.1	0.617035377	7.32E-58 postive
WIPI2	AC138305.1	0.40101577	3.06E-22 postive
LINC00472	AC138305.1	0.521869242	5.54E-39 postive
LPIN1	AC138305.1	0.639971342	1.95E-63 postive
FANCD2	Z84485.1	0.434654016	3.01E-26 postive
HELLS	Z84485.1	0.442674701	2.85E-27 postive
ZNF419	Z84485.1	0.441886528	3.60E-27 postive
TUBE1	Z84485.1	0.419212099	2.37E-24 postive
ALOX12	Z84485.1	0.543131428	1.10E-42 postive
PHKG2	Z84485.1	0.48418052	5.00E-33 postive
TAZ	Z84485.1	0.501423909	1.16E-35 postive
SLC3A2	AL031710.1	0.422063302	1.07E-24 postive
FH	AL031710.1	0.513797509	1.21E-37 postive
ISCU	AL031710.1	0.645123763	9.35E-65 postive
DDIT3	AL031710.1	0.553805773	1.20E-44 postive
ATP5MC3	AL031710.1	0.40764356	5.39E-23 postive
SLC2A8	AL031710.1	0.507531829	1.25E-36 postive
SLC2A12	AL031710.1	0.452107413	1.64E-28 postive

GOT1	AL031710.1	0.54198345	1.77E-42	postive
ATG4D	AL031710.1	0.650402334	3.92E-66	postive
MAP1LC3A	AL031710.1	0.64636358	4.47E-65	postive
GABARAPL2	AL031710.1	0.601281666	2.72E-54	postive
GABARAPL1	AL031710.1	0.606718979	1.68E-55	postive
WIPI2	AL031710.1	0.479319688	2.60E-32	postive
LPIN1	AL031710.1	0.448904637	4.37E-28	postive
ZNF419	AL133367.1	0.542498204	1.43E-42	postive
TUBE1	AL133367.1	0.430116765	1.11E-25	postive
SETD1B	AL133367.1	0.467163041	1.43E-30	postive
ALOX12	AL133367.1	0.582589147	2.64E-50	postive
TAZ	AL133367.1	0.433156785	4.64E-26	postive
ATF4	MAPKAPK5-/	0.438039618	1.12E-26	postive
RPL8	MAPKAPK5-/	0.535614695	2.40E-41	postive
HRAS	MAPKAPK5-/	0.50954635	5.93E-37	postive
MAP1LC3A	MAPKAPK5-/	0.436705693	1.66E-26	postive
MAPK1	MAPKAPK5-/	-0.434794768	2.89E-26	negative
EGLN2	MAPKAPK5-/	0.518266997	2.22E-38	postive
TAZ	MAPKAPK5-/	0.400533206	3.47E-22	postive
HELLS	FSIP2-AS1	0.455694148	5.41E-29	postive
ZNF419	FSIP2-AS1	0.421037598	1.43E-24	postive
TUBE1	FSIP2-AS1	0.603872894	7.26E-55	postive
ALOX12	FSIP2-AS1	0.554617718	8.45E-45	postive
GABPB1	FSIP2-AS1	0.468064829	1.07E-30	postive
ATM	FSIP2-AS1	0.609507087	3.94E-56	postive
TAZ	FSIP2-AS1	0.471140488	3.92E-31	postive
FBXW7	FSIP2-AS1	0.484685841	4.21E-33	postive
HELLS	AC026356.2	0.555768565	5.13E-45	postive
ZNF419	AC026356.2	0.420297793	1.75E-24	postive
TUBE1	AC026356.2	0.472111823	2.85E-31	postive
ALOX12	AC026356.2	0.472137166	2.82E-31	postive
GABPB1	AC026356.2	0.488996604	9.52E-34	postive
ATM	AC026356.2	0.543794754	8.32E-43	postive
FBXW7	AC026356.2	0.474183345	1.44E-31	postive
ZNF419	AL136295.6	0.467047069	1.48E-30	postive
ALOX12	AL136295.6	0.493167856	2.22E-34	postive
TFAP2C	AL136295.6	0.460799403	1.09E-29	postive
HBA1	AL136295.6	0.533053402	6.74E-41	postive
EGLN2	AL136295.6	0.427630462	2.25E-25	postive
FANCD2	AC022558.3	0.403003314	1.82E-22	postive
HELLS	AC022558.3	0.483393427	6.54E-33	postive
ZNF419	AC022558.3	0.511182336	3.22E-37	postive
TUBE1	AC022558.3	0.495779818	8.80E-35	postive
ALOX12	AC022558.3	0.554158923	1.03E-44	postive
GABPB1	AC022558.3	0.539438148	5.04E-42	postive
LINC00472	AC022558.3	0.67889836	4.54E-74	postive
LPIN1	AC022558.3	0.417462374	3.83E-24	postive
ATM	AC022558.3	0.509252798	6.61E-37	postive
FBXW7	AC022558.3	0.475445879	9.47E-32	postive
HELLS	AC009119.2	0.419207479	2.37E-24	postive
TUBE1	AC009119.2	0.457079507	3.51E-29	postive
ALOX12	AC009119.2	0.53296596	6.98E-41	postive
ATM	AC009119.2	0.60944572	4.07E-56	postive
FBXW7	AC009119.2	0.413223593	1.21E-23	postive
RB1	AF127577.4	0.443737645	2.07E-27	postive
HELLS	AF127577.4	0.438625188	9.43E-27	postive
ACSL3	AF127577.4	0.401809878	2.49E-22	postive
OTUB1	AF127577.4	-0.413494887	1.13E-23	negative

ZFP69B	AF127577.4	0.459341706	1.73E-29	postive
IREB2	AF127577.4	0.439650159	6.98E-27	postive
PIK3CA	AF127577.4	0.417625904	3.66E-24	postive
NRAS	AF127577.4	0.451017513	2.29E-28	postive
ATG5	AF127577.4	0.427068956	2.64E-25	postive
MAPK8	AF127577.4	0.419443563	2.22E-24	postive
PRKAA1	AF127577.4	0.426996345	2.70E-25	postive
ATM	AF127577.4	0.439658349	6.96E-27	postive
MTDH	AF127577.4	0.45373434	9.93E-29	postive
BACH1	AF127577.4	0.494247992	1.51E-34	postive
HELLS	AC006159.1	0.435651717	2.25E-26	postive
KLHL24	AC006159.1	0.442239354	3.24E-27	postive
GABPB1	AC006159.1	0.504641597	3.62E-36	postive
PIK3CA	AC006159.1	0.407347327	5.83E-23	postive
LINC00472	AC006159.1	0.830264899	1.98E-138	postive
ATM	AC006159.1	0.623878962	1.78E-59	postive
FBXW7	AC006159.1	0.401016999	3.06E-22	postive
FANCD2	ETV7-AS1	0.447672786	6.34E-28	postive
HELLS	ETV7-AS1	0.432211583	6.09E-26	postive
ALOX12	ETV7-AS1	0.425640199	3.95E-25	postive
ZNF419	AC084824.5	0.495773285	8.82E-35	postive
TUBE1	AC084824.5	0.522489771	4.36E-39	postive
ALOX12	AC084824.5	0.443003694	2.58E-27	postive
GABPB1	AC084824.5	0.431126834	8.32E-26	postive
NCOA4	AC084824.5	-0.409296881	3.47E-23	negative
PHKG2	AC084824.5	0.564219291	1.25E-46	postive
BECN1	AC084824.5	-0.40129162	2.85E-22	negative
LINC00472	AC084824.5	0.449370947	3.79E-28	postive
TAZ	AC084824.5	0.668822134	3.66E-71	postive
FBXW7	AC084824.5	0.422418083	9.74E-25	postive
HELLS	AL008729.1	0.47904449	2.85E-32	postive
ZNF419	AL008729.1	0.427326319	2.45E-25	postive
TUBE1	AL008729.1	0.540295858	3.54E-42	postive
ALOX12	AL008729.1	0.661162684	5.00E-69	postive
GABPB1	AL008729.1	0.412439148	1.50E-23	postive
ATM	AL008729.1	0.45924192	1.78E-29	postive
FBXW7	AL008729.1	0.419679141	2.08E-24	postive
CHAC1	AC018413.1	0.486853492	2.00E-33	postive
DDIT3	AC018413.1	0.408090234	4.79E-23	postive
MAP1LC3A	AC018413.1	0.490134962	6.41E-34	postive
KLHL24	LINC00472	0.642490877	4.45E-64	postive
IREB2	LINC00472	0.490041763	6.62E-34	postive
GABPB1	LINC00472	0.501292726	1.22E-35	postive
PIK3CA	LINC00472	0.536295849	1.82E-41	postive
KRAS	LINC00472	0.461858254	7.80E-30	postive
MAPK8	LINC00472	0.431025732	8.56E-26	postive
LINC00472	LINC00472	1	0	postive
PRKAA1	LINC00472	0.433325778	4.42E-26	postive
LPIN1	LINC00472	0.443954026	1.94E-27	postive
ATM	LINC00472	0.514102342	1.08E-37	postive
HSPB1	LINC02846	0.425072516	4.63E-25	postive
HRAS	LINC02846	0.417461963	3.83E-24	postive
PHKG2	LINC02846	0.420149688	1.83E-24	postive
TAZ	LINC02846	0.408142867	4.72E-23	postive
PHKG2	AL078604.2	0.445325295	1.29E-27	postive
HELLS	AC011815.1	0.456378989	4.37E-29	postive
ZNF419	AC011815.1	0.464028026	3.91E-30	postive
KLHL24	AC011815.1	0.567025147	3.54E-47	postive

TUBE1	AC011815.1	0.403343493	1.67E-22	postive
IREB2	AC011815.1	0.531625661	1.19E-40	postive
GABPB1	AC011815.1	0.58942931	9.83E-52	postive
PIK3CA	AC011815.1	0.483112553	7.20E-33	postive
KRAS	AC011815.1	0.426681941	2.95E-25	postive
ATG7	AC011815.1	0.400799746	3.23E-22	postive
MAPK8	AC011815.1	0.45587889	5.11E-29	postive
LINC00472	AC011815.1	0.778052293	1.67E-110	postive
PRKAA1	AC011815.1	0.435380591	2.44E-26	postive
LPIN1	AC011815.1	0.48526764	3.45E-33	postive
ATM	AC011815.1	0.626831701	3.47E-60	postive
FBXW7	AC011815.1	0.474023299	1.52E-31	postive
HELLS	MIR29B2CHG	0.503762589	4.98E-36	postive
ZNF419	MIR29B2CHG	0.412030068	1.67E-23	postive
TUBE1	MIR29B2CHG	0.471017138	4.08E-31	postive
ALOX12	MIR29B2CHG	0.496000688	8.14E-35	postive
GABPB1	MIR29B2CHG	0.570037072	9.06E-48	postive
ATG7	MIR29B2CHG	0.439386086	7.54E-27	postive
MAPK8	MIR29B2CHG	0.413585414	1.10E-23	postive
LINC00472	MIR29B2CHG	0.69144634	7.39E-78	postive
ATM	MIR29B2CHG	0.728165497	3.77E-90	postive
FBXW7	MIR29B2CHG	0.490503979	5.63E-34	postive
ZNF419	AC020915.2	0.471459856	3.53E-31	postive
ALOX12	AC020915.2	0.400533597	3.46E-22	postive
ATM	AC020915.2	0.421565812	1.23E-24	postive
HELLS	AL158834.2	0.429401429	1.36E-25	postive
ZNF419	AL158834.2	0.491339473	4.21E-34	postive
TUBE1	AL158834.2	0.616937765	7.71E-58	postive
ALOX12	AL158834.2	0.58298945	2.18E-50	postive
GABPB1	AL158834.2	0.491535456	3.93E-34	postive
PHKG2	AL158834.2	0.440295045	5.77E-27	postive
ATM	AL158834.2	0.507175075	1.43E-36	postive
TAZ	AL158834.2	0.548849892	9.96E-44	postive
FBXW7	AL158834.2	0.477665026	4.52E-32	postive
RPL8	FOXN3-AS1	0.401765855	2.52E-22	postive
HRAS	FOXN3-AS1	0.409336983	3.44E-23	postive
GPX4	SPINT1-AS1	0.491789021	3.60E-34	postive
CISD1	SPINT1-AS1	0.413988778	9.85E-24	postive
FH	SPINT1-AS1	0.448439562	5.03E-28	postive
ISCU	SPINT1-AS1	0.44008854	6.13E-27	postive
OTUB1	SPINT1-AS1	0.46112025	9.86E-30	postive
ATP5MC3	SPINT1-AS1	0.449725434	3.40E-28	postive
SLC2A8	SPINT1-AS1	0.440051494	6.20E-27	postive
GOT1	SPINT1-AS1	0.465298472	2.60E-30	postive
ATG4D	SPINT1-AS1	0.533313974	6.07E-41	postive
MAP1LC3A	SPINT1-AS1	0.444304521	1.75E-27	postive
GABARAPL2	SPINT1-AS1	0.600201574	4.70E-54	postive
HELLS	AC007991.2	0.491735748	3.66E-34	postive
TUBE1	AC007991.2	0.449140268	4.06E-28	postive
ALOX12	AC007991.2	0.459141705	1.84E-29	postive
GABPB1	AC007991.2	0.468519005	9.20E-31	postive
LINC00472	AC007991.2	0.409452137	3.33E-23	postive
ATM	AC007991.2	0.671196597	7.75E-72	postive
FBXW7	AC007991.2	0.466588748	1.72E-30	postive
ISCU	AC104958.2	0.60341821	9.17E-55	postive
DDIT3	AC104958.2	0.415386617	6.74E-24	postive
ATG4D	AC104958.2	0.432307537	5.92E-26	postive
MAP1LC3A	AC104958.2	0.40251227	2.07E-22	postive

GABARAPL1	AC104958.2	0.508229438	9.66E-37	postive
HELLS	AC010615.2	0.479753829	2.24E-32	postive
ZNF419	AC010615.2	0.400900062	3.15E-22	postive
KLHL24	AC010615.2	0.494198697	1.54E-34	postive
IREB2	AC010615.2	0.404391966	1.27E-22	postive
PIK3CA	AC010615.2	0.417758964	3.53E-24	postive
LINC00472	AC010615.2	0.600042924	5.09E-54	postive
ATM	AC010615.2	0.472200302	2.77E-31	postive
FANCD2	AC025048.2	0.401503973	2.69E-22	postive
STMN1	AC025048.2	0.533687974	5.22E-41	postive
CDKN2A	AC025048.2	0.56124469	4.67E-46	postive
HELLS	AC109587.1	0.497335113	5.06E-35	postive
ZNF419	AC109587.1	0.481422612	1.28E-32	postive
KLHL24	AC109587.1	0.456418364	4.32E-29	postive
TUBE1	AC109587.1	0.571491941	4.67E-48	postive
ALOX12	AC109587.1	0.406844039	6.66E-23	postive
GABPB1	AC109587.1	0.677437875	1.22E-73	postive
PIK3CA	AC109587.1	0.411133574	2.13E-23	postive
KRAS	AC109587.1	0.402031428	2.35E-22	postive
MAPK8	AC109587.1	0.425504685	4.10E-25	postive
LINC00472	AC109587.1	0.783532226	4.62E-113	postive
ATM	AC109587.1	0.631718708	2.24E-61	postive
FBXW7	AC109587.1	0.557082599	2.90E-45	postive
HELLS	AL080317.2	0.524589045	1.93E-39	postive
TUBE1	AL080317.2	0.60590266	2.56E-55	postive
ALOX12	AL080317.2	0.512912339	1.69E-37	postive
GABPB1	AL080317.2	0.453047636	1.23E-28	postive
LINC00472	AL080317.2	0.452664621	1.38E-28	postive
ATM	AL080317.2	0.612839237	6.84E-57	postive
FBXW7	AL080317.2	0.431323296	7.86E-26	postive
TFAP2C	LINC01976	0.453360768	1.11E-28	postive
HBA1	LINC01976	0.8063365	1.42E-124	postive
HRAS	LINC01976	0.449923728	3.20E-28	postive
EGLN2	LINC01976	0.622509528	3.77E-59	postive
CD44	LINC01615	0.494040495	1.63E-34	postive
AURKA	LINC01615	0.410877241	2.28E-23	postive
KLHL24	LINC00513	0.560736981	5.85E-46	postive
IREB2	LINC00513	0.414801362	7.90E-24	postive
GABPB1	LINC00513	0.479816499	2.20E-32	postive
PIK3CA	LINC00513	0.460241316	1.30E-29	postive
LINC00472	LINC00513	0.921631903	6.51E-223	postive
ATM	LINC00513	0.457477739	3.10E-29	postive
GABPB1	AP000240.1	0.407829828	5.13E-23	postive
NOX1	AP000240.1	0.401612124	2.62E-22	postive
BECN1	AP000240.1	-0.459111902	1.86E-29	negative
LINC00472	AP000240.1	0.422791188	8.78E-25	postive
FBXW7	AP000240.1	0.452069828	1.66E-28	postive
FANCD2	HCG25	0.466916309	1.55E-30	postive
HELLS	HCG25	0.437760457	1.22E-26	postive
ALOX12	HCG25	0.583941629	1.39E-50	postive
TFAP2C	HCG25	0.441583678	3.94E-27	postive
DUOX1	HCG25	0.429762135	1.23E-25	postive
GPX4	ZSCAN16-AS	0.452374875	1.51E-28	postive
HSPB1	ZSCAN16-AS	0.451627664	1.90E-28	postive
RPL8	ZSCAN16-AS	0.456304158	4.47E-29	postive
HBA1	ZSCAN16-AS	0.605981963	2.46E-55	postive
HRAS	ZSCAN16-AS	0.733345559	4.77E-92	postive
MAP1LC3A	ZSCAN16-AS	0.503414656	5.66E-36	postive

EGLN2	ZSCAN16-AS	0.814326074	5.63E-129	postive
ATM	LINC00649	0.411235391	2.07E-23	postive
HELLS	AC011377.1	0.53616242	1.92E-41	postive
TUBE1	AC011377.1	0.422415622	9.74E-25	postive
ALOX12	AC011377.1	0.529589937	2.69E-40	postive
ATM	AC011377.1	0.507586978	1.23E-36	postive
FBXW7	AC011377.1	0.401293773	2.85E-22	postive
HELLS	AC016747.3	0.526209215	1.02E-39	postive
ZNF419	AC016747.3	0.434570103	3.08E-26	postive
TUBE1	AC016747.3	0.527769538	5.52E-40	postive
ALOX12	AC016747.3	0.522743197	3.95E-39	postive
GABPB1	AC016747.3	0.588923759	1.26E-51	postive
MAPK8	AC016747.3	0.440813301	4.95E-27	postive
LINC00472	AC016747.3	0.754394212	3.06E-100	postive
ATM	AC016747.3	0.755982085	6.82E-101	postive
FBXW7	AC016747.3	0.531289101	1.37E-40	postive
ZNF419	MIR3936HG	0.456274525	4.52E-29	postive
VEGFA	MIR3936HG	0.431300031	7.91E-26	postive
TUBE1	MIR3936HG	0.614072009	3.56E-57	postive
ALOX12	MIR3936HG	0.657374897	5.39E-68	postive
ATM	MIR3936HG	0.507508105	1.26E-36	postive
TAZ	MIR3936HG	0.492890271	2.44E-34	postive
MAPK1	EGOT	0.413411268	1.15E-23	postive
MAPK8	EGOT	0.479394756	2.53E-32	postive
PRKAA2	EGOT	0.406545763	7.20E-23	postive
ATM	EGOT	0.425953348	3.62E-25	postive
SIRT1	EGOT	0.413782305	1.04E-23	postive
ZNF419	AC034236.2	0.52005303	1.12E-38	postive
TUBE1	AC034236.2	0.461835026	7.86E-30	postive
DRD4	AC034236.2	0.460955415	1.04E-29	postive
ALOX12	AC034236.2	0.47989334	2.14E-32	postive
NRAS	AC034236.2	-0.412302553	1.55E-23	negative
PHKG2	AC034236.2	0.659993999	1.04E-68	postive
TAZ	AC034236.2	0.715887872	8.01E-86	postive
AKR1C1	AC007036.1	0.428930441	1.56E-25	postive
ZNF419	AC048341.2	0.511889558	2.48E-37	postive
TUBE1	AC048341.2	0.416429023	5.07E-24	postive
ALOX12	AC048341.2	0.409018823	3.74E-23	postive
PHKG2	AC048341.2	0.655306548	1.94E-67	postive
SAT1	AC048341.2	0.409591415	3.21E-23	postive
SOCS1	AC048341.2	0.428582765	1.72E-25	postive
TAZ	AC048341.2	0.683241538	2.33E-75	postive
ALOX12	SLC6A12-AS	0.499985297	1.96E-35	postive
HELLS	AC032044.1	0.526101019	1.06E-39	postive
ZNF419	AC032044.1	0.419420759	2.23E-24	postive
TUBE1	AC032044.1	0.465256648	2.64E-30	postive
ALOX12	AC032044.1	0.583247133	1.93E-50	postive
GABPB1	AC032044.1	0.498514814	3.32E-35	postive
LINC00472	AC032044.1	0.480131422	1.98E-32	postive
ATM	AC032044.1	0.660331578	8.44E-69	postive
FBXW7	AC032044.1	0.492958554	2.38E-34	postive
FANCD2	AP001107.1	0.416164793	5.45E-24	postive
HELLS	AP001107.1	0.434337874	3.30E-26	postive
ZNF419	AP001107.1	0.447432528	6.82E-28	postive
TUBE1	AP001107.1	0.437233113	1.42E-26	postive
ALOX12	AP001107.1	0.637163354	9.94E-63	postive
TFAP2C	AP001107.1	0.435952115	2.06E-26	postive
HBA1	AP001107.1	0.406113299	8.07E-23	postive

DUOX1	AP001107.1	0.425153135	4.53E-25	postive
FBXW7	AP001107.1	0.403401035	1.64E-22	postive
ZNF419	LINC01786	0.41249972	1.47E-23	postive
TUBE1	LINC01786	0.42417609	5.96E-25	postive
ALOX12	LINC01786	0.634330804	5.06E-62	postive
PHKG2	LINC01786	0.409089275	3.67E-23	postive
TAZ	LINC01786	0.561302473	4.55E-46	postive
HBA1	ARHGAP29-A	0.426433662	3.16E-25	postive
EGLN2	ARHGAP29-A	0.406894728	6.57E-23	postive
ATM	AL157395.1	0.435765589	2.18E-26	postive
HELLS	AL138963.1	0.555398122	6.03E-45	postive
TUBE1	AL138963.1	0.431399783	7.69E-26	postive
ALOX12	AL138963.1	0.449462445	3.68E-28	postive
GABPB1	AL138963.1	0.550860976	4.24E-44	postive
ATG7	AL138963.1	0.437127396	1.46E-26	postive
LINC00472	AL138963.1	0.73100618	3.48E-91	postive
ATM	AL138963.1	0.708192237	3.17E-83	postive
FBXW7	AL138963.1	0.465343718	2.56E-30	postive
ALOX12	DIP2C-AS1	0.424809197	4.99E-25	postive
MAPK8	DIP2C-AS1	0.408428919	4.37E-23	postive
ATM	DIP2C-AS1	0.550042012	6.00E-44	postive
HELLS	AC068790.6	0.517670664	2.78E-38	postive
KLHL24	AC068790.6	0.426016733	3.55E-25	postive
TUBE1	AC068790.6	0.443464297	2.25E-27	postive
ALOX12	AC068790.6	0.416284956	5.28E-24	postive
IREB2	AC068790.6	0.421420951	1.28E-24	postive
GABPB1	AC068790.6	0.574987634	9.34E-49	postive
PIK3CA	AC068790.6	0.423020966	8.23E-25	postive
ATG7	AC068790.6	0.418251596	3.08E-24	postive
MAPK8	AC068790.6	0.44334521	2.33E-27	postive
LINC00472	AC068790.6	0.790179243	2.87E-116	postive
ATM	AC068790.6	0.731971766	1.54E-91	postive
FBXW7	AC068790.6	0.485810305	2.86E-33	postive
HELLS	AC026356.1	0.542348992	1.52E-42	postive
GABPB1	AC026356.1	0.516011147	5.23E-38	postive
LINC00472	AC026356.1	0.515815122	5.64E-38	postive
ATM	AC026356.1	0.596708747	2.72E-53	postive
BACH1	AC026356.1	0.427916309	2.08E-25	postive
MTOR	AC008764.2	0.445782936	1.12E-27	postive
ZNF419	AC008764.2	0.477362422	5.00E-32	postive
TUBE1	AC008764.2	0.430576288	9.74E-26	postive
SETD1B	AC008764.2	0.560327915	7.00E-46	postive
ALOX12	AC008764.2	0.488690519	1.06E-33	postive
IREB2	AC008764.2	0.449993022	3.13E-28	postive
ULK2	AC008764.2	0.404056128	1.39E-22	postive
LPIN1	AC008764.2	0.40985892	2.99E-23	postive
ATM	AC008764.2	0.455438363	5.86E-29	postive
HELLS	AC018926.2	0.522392342	4.53E-39	postive
ZNF419	AC018926.2	0.420240402	1.78E-24	postive
TUBE1	AC018926.2	0.452203432	1.59E-28	postive
ALOX12	AC018926.2	0.410159776	2.76E-23	postive
GABPB1	AC018926.2	0.511158082	3.25E-37	postive
PHKG2	AC018926.2	0.403204182	1.73E-22	postive
LINC00472	AC018926.2	0.518996404	1.68E-38	postive
ATM	AC018926.2	0.507636659	1.20E-36	postive
TAZ	AC018926.2	0.411649577	1.85E-23	postive
FBXW7	AC018926.2	0.468714861	8.63E-31	postive
ATG4D	WWTR1-AS1	0.494130511	1.58E-34	postive

TUBE1	CNNM3-DT	0.409715587	3.11E-23	postive
PEBP1	CNNM3-DT	0.457572032	3.01E-29	postive
ZEB1	AC068870.2	0.409228734	3.54E-23	postive
EPAS1	AC068870.2	0.484183929	4.99E-33	postive
HELLS	AC010999.1	0.474846288	1.16E-31	postive
ZNF419	AC010999.1	0.506307838	1.96E-36	postive
TUBE1	AC010999.1	0.573179223	2.15E-48	postive
ALOX12	AC010999.1	0.542975979	1.17E-42	postive
GABPB1	AC010999.1	0.56409853	1.32E-46	postive
LINC00472	AC010999.1	0.597205859	2.12E-53	postive
ATM	AC010999.1	0.599341209	7.26E-54	postive
FBXW7	AC010999.1	0.452133706	1.63E-28	postive
ZNF419	INTS6-AS1	0.551900591	2.72E-44	postive
VEGFA	INTS6-AS1	0.418151401	3.17E-24	postive
TUBE1	INTS6-AS1	0.559172221	1.16E-45	postive
ALOX12	INTS6-AS1	0.718947916	7.02E-87	postive
GABPB1	INTS6-AS1	0.421672324	1.20E-24	postive
ATM	INTS6-AS1	0.426505193	3.10E-25	postive
TAZ	INTS6-AS1	0.481846351	1.11E-32	postive
ALOX12	NBR2	0.56810749	2.17E-47	postive
PHKG2	NBR2	0.409909306	2.95E-23	postive
TAZ	NBR2	0.406216752	7.85E-23	postive
SRC	PICART1	0.466010592	2.07E-30	postive
PHKG2	PICART1	0.500583965	1.58E-35	postive
TAZ	PICART1	0.480893028	1.53E-32	postive
TAZ	AL158071.2	0.408366453	4.45E-23	postive
ZFP69B	AL031985.3	0.7680062	5.38E-106	postive
ATM	AC009166.1	0.405109344	1.05E-22	postive
HELLS	AC011510.1	0.512008605	2.37E-37	postive
TUBE1	AC011510.1	0.457338891	3.24E-29	postive
ALOX12	AC011510.1	0.470806433	4.37E-31	postive
GABPB1	AC011510.1	0.515783753	5.71E-38	postive
ATG7	AC011510.1	0.407052217	6.30E-23	postive
LINC00472	AC011510.1	0.504310719	4.08E-36	postive
ATM	AC011510.1	0.660836286	6.14E-69	postive
FBXW7	AC011510.1	0.503586013	5.31E-36	postive
SLC3A2	Z82185.1	0.469253519	7.25E-31	postive
FH	Z82185.1	0.515484934	6.39E-38	postive
ISCU	Z82185.1	0.656920037	7.15E-68	postive
DDIT3	Z82185.1	0.431251102	8.02E-26	postive
SLC2A8	Z82185.1	0.423101175	8.05E-25	postive
SLC2A12	Z82185.1	0.406855236	6.64E-23	postive
CS	Z82185.1	0.476116376	7.58E-32	postive
GOT1	Z82185.1	0.49031224	6.02E-34	postive
ATG4D	Z82185.1	0.634129954	5.68E-62	postive
GABARAPL2	Z82185.1	0.513415645	1.40E-37	postive
GABARAPL1	Z82185.1	0.696709824	1.67E-79	postive
LPIN1	Z82185.1	0.525589143	1.30E-39	postive
ATF4	EPB41L4A-A!	0.443161479	2.46E-27	postive
RPL8	EPB41L4A-A!	0.468336291	9.76E-31	postive
ARNTL	AC079921.2	0.408608659	4.17E-23	postive
ZNF419	AC079921.2	0.448659473	4.70E-28	postive
TUBE1	AC079921.2	0.513075098	1.59E-37	postive
SETD1B	AC079921.2	0.451179811	2.18E-28	postive
ALOX12	AC079921.2	0.554113465	1.05E-44	postive
GABPB1	AC079921.2	0.538772165	6.62E-42	postive
ZEB1	AC079921.2	0.542177108	1.63E-42	postive
MAPK8	AC079921.2	0.474133592	1.46E-31	postive

ATM	AC079921.2	0.65496	2.41E-67	postive
YY1AP1	AC079921.2	0.448002037	5.74E-28	postive
FBXW7	AC079921.2	0.527241588	6.80E-40	postive
PHKG2	AL590560.3	0.508680342	8.18E-37	postive
TAZ	AL590560.3	0.603290954	9.78E-55	postive
RGS4	AC010247.2	0.674712901	7.56E-73	postive
ACSL3	AC087071.2	0.454977966	6.76E-29	postive
KLHL24	AC087071.2	0.627495649	2.40E-60	postive
IREB2	AC087071.2	0.521468753	6.47E-39	postive
GABPB1	AC087071.2	0.468883756	8.17E-31	postive
PIK3CA	AC087071.2	0.543478006	9.50E-43	postive
KRAS	AC087071.2	0.507631201	1.21E-36	postive
MAPK8	AC087071.2	0.417760963	3.53E-24	postive
LINC00472	AC087071.2	0.849272947	4.38E-151	postive
PRKAA1	AC087071.2	0.459521711	1.63E-29	postive
TGFBR1	AC087071.2	0.426239391	3.34E-25	postive
LPIN1	AC087071.2	0.459795922	1.50E-29	postive
ATM	AC087071.2	0.47047262	4.87E-31	postive
NOX1	AC010969.2	0.495040262	1.14E-34	postive
HRAS	AC010969.2	0.563181944	1.98E-46	postive
PHKG2	AC010969.2	0.460368842	1.25E-29	postive
BECN1	AC010969.2	-0.439639804	7.00E-27	negative
EGLN2	AC010969.2	0.456954806	3.65E-29	postive
TAZ	AC010969.2	0.422600885	9.25E-25	postive
MUC1	AC023669.1	0.421334202	1.32E-24	postive
SLC7A5	AC023669.1	0.452733673	1.35E-28	postive
SLC2A1	AC023669.1	0.46929412	7.15E-31	postive
HELLS	AC009318.2	0.505845469	2.33E-36	postive
GABPB1	AC009318.2	0.440123884	6.07E-27	postive
TGFBR1	AC009318.2	0.416398117	5.12E-24	postive
ATM	AC009318.2	0.505426479	2.71E-36	postive
FBXW7	AC009318.2	0.448434817	5.04E-28	postive
BACH1	AC009318.2	0.443266569	2.39E-27	postive
ALB	LINC01780	0.403035389	1.81E-22	postive
PCK2	LINC01780	0.476891707	5.86E-32	postive
AGPAT3	CCDC183-AS	0.442065681	3.41E-27	postive
MIOX	CCDC183-AS	0.428062993	1.99E-25	postive
TFAP2C	AC009090.1	0.553051391	1.66E-44	postive
HBA1	AC009090.1	0.654201637	3.85E-67	postive
DUOX1	AC009090.1	0.40124773	2.88E-22	postive
EGLN2	AC009090.1	0.410093233	2.81E-23	postive
SLC3A2	AP000757.2	0.430621102	9.61E-26	postive
ISCU	AP000757.2	0.497774572	4.32E-35	postive
DDIT3	AP000757.2	0.49157569	3.87E-34	postive
PHKG2	AP000757.2	0.412962406	1.30E-23	postive
ATG4D	AP000757.2	0.586781229	3.55E-51	postive
GABARAPL1	AP000757.2	0.456564641	4.13E-29	postive
BRD4	LINC01278	0.469136997	7.53E-31	postive
SETD1B	LINC01278	0.553408232	1.42E-44	postive
ALOX12	LINC01278	0.476141564	7.52E-32	postive
YY1AP1	LINC01278	0.445437005	1.25E-27	postive
HELLS	AP001893.1	0.459433778	1.68E-29	postive
ZNF419	AP001893.1	0.447446438	6.79E-28	postive
TUBE1	AP001893.1	0.586992117	3.20E-51	postive
SETD1B	AP001893.1	0.408813285	3.95E-23	postive
ALOX12	AP001893.1	0.588716718	1.39E-51	postive
IREB2	AP001893.1	0.503787077	4.94E-36	postive
GABPB1	AP001893.1	0.569244475	1.30E-47	postive

PIK3CA	AP001893.1	0.42746844	2.36E-25	postive
MAPK8	AP001893.1	0.562993884	2.15E-46	postive
LINC00472	AP001893.1	0.668406336	4.80E-71	postive
ATM	AP001893.1	0.758215325	8.11E-102	postive
FBXW7	AP001893.1	0.462798839	5.78E-30	postive
ZNF419	GRPEL2-AS1	0.497772087	4.33E-35	postive
VEGFA	GRPEL2-AS1	0.449474329	3.67E-28	postive
TUBE1	GRPEL2-AS1	0.432641291	5.38E-26	postive
SETD1B	GRPEL2-AS1	0.428403761	1.81E-25	postive
ALOX12	GRPEL2-AS1	0.428938801	1.55E-25	postive
GABPB1	GRPEL2-AS1	0.433825891	3.82E-26	postive
ZEB1	GRPEL2-AS1	0.510288158	4.50E-37	postive
FBXW7	GRPEL2-AS1	0.433847546	3.80E-26	postive
HELLS	AC006504.7	0.423446837	7.31E-25	postive
KLHL24	AC006504.7	0.425372	4.26E-25	postive
LINC00472	AC006504.7	0.553252537	1.52E-44	postive
ALOX12	C1orf229	0.519187605	1.56E-38	postive
HELLS	LINC00662	0.483570747	6.16E-33	postive
HELLS	AC105339.3	0.588963365	1.23E-51	postive
ZNF419	AC105339.3	0.424381468	5.63E-25	postive
TUBE1	AC105339.3	0.510114447	4.80E-37	postive
ALOX12	AC105339.3	0.617590372	5.43E-58	postive
GABPB1	AC105339.3	0.54508825	4.85E-43	postive
LINC00472	AC105339.3	0.57394595	1.51E-48	postive
ATM	AC105339.3	0.707510291	5.33E-83	postive
FBXW7	AC105339.3	0.493615231	1.89E-34	postive
TUBE1	TNFRSF14-A	0.431382064	7.73E-26	postive
ALOX12	TNFRSF14-A	0.451175078	2.18E-28	postive
ATG4D	TNFRSF14-A	0.433464806	4.24E-26	postive
TAZ	TNFRSF14-A	0.418167959	3.15E-24	postive
SLC2A6	AC104758.1	0.422089182	1.07E-24	postive
NCOA4	AC104758.1	-0.439096563	8.21E-27	negative
PHKG2	AC104758.1	0.559530926	9.94E-46	postive
TAZ	AC104758.1	0.60008167	5.00E-54	postive
HELLS	AC093788.1	0.43966712	6.94E-27	postive
ZNF419	AC093788.1	0.453238897	1.16E-28	postive
KLHL24	AC093788.1	0.441858473	3.63E-27	postive
TUBE1	AC093788.1	0.488563702	1.11E-33	postive
GABPB1	AC093788.1	0.571660798	4.32E-48	postive
LINC00472	AC093788.1	0.832186432	1.23E-139	postive
ATM	AC093788.1	0.548051549	1.40E-43	postive
FBXW7	AC093788.1	0.479161032	2.74E-32	postive
ZNF419	AL355987.4	0.445012542	1.41E-27	postive
TAZ	AL355987.4	0.405956726	8.41E-23	postive
FH	AC103563.7	0.430824814	9.07E-26	postive
ISCU	AC103563.7	0.731182521	3.00E-91	postive
DDIT3	AC103563.7	0.509631167	5.75E-37	postive
KLHL24	AC103563.7	0.43681998	1.60E-26	postive
SLC2A12	AC103563.7	0.457832644	2.78E-29	postive
CS	AC103563.7	0.400163779	3.81E-22	postive
GOT1	AC103563.7	0.4652687	2.63E-30	postive
ATG4D	AC103563.7	0.663493284	1.14E-69	postive
MAP1LC3A	AC103563.7	0.524745861	1.81E-39	postive
GABARAPL2	AC103563.7	0.512947557	1.66E-37	postive
GABARAPL1	AC103563.7	0.741945164	2.68E-95	postive
WIPI2	AC103563.7	0.507042893	1.50E-36	postive
BAP1	AC103563.7	0.476901777	5.84E-32	postive
LPIN1	AC103563.7	0.583971924	1.37E-50	postive

SLC3A2	INSIG1-DT	0.517466894	3.01E-38	postive
ISCU	INSIG1-DT	0.507485632	1.27E-36	postive
ASNS	INSIG1-DT	0.414321074	9.00E-24	postive
DDIT3	INSIG1-DT	0.604632247	4.92E-55	postive
ATG4D	INSIG1-DT	0.425665218	3.92E-25	postive
GABARAPL2	INSIG1-DT	0.41994086	1.94E-24	postive
GABARAPL1	INSIG1-DT	0.594713715	7.33E-53	postive
LPIN1	INSIG1-DT	0.448754437	4.57E-28	postive
HELLS	LINC02100	0.405942234	8.44E-23	postive
ATM	LINC02100	0.442424451	3.07E-27	postive
HELLS	AC027796.1	0.569280246	1.28E-47	postive
TUBE1	AC027796.1	0.438104439	1.10E-26	postive
ALOX12	AC027796.1	0.594018031	1.04E-52	postive
GABPB1	AC027796.1	0.44901807	4.22E-28	postive
DUOX1	AC027796.1	0.419477897	2.20E-24	postive
LINC00472	AC027796.1	0.41460867	8.33E-24	postive
ATM	AC027796.1	0.606720848	1.68E-55	postive
FBXW7	AC027796.1	0.453900787	9.43E-29	postive
FANCD2	LINC02084	0.48971658	7.41E-34	postive
PML	LINC02084	0.401583235	2.64E-22	postive
SLC2A6	LINC02084	0.403033407	1.81E-22	postive
IFNG	LINC02084	0.637279004	9.30E-63	postive
ZNF419	AC113139.1	0.424388319	5.62E-25	postive
PHKG2	AC113139.1	0.467995107	1.09E-30	postive
MAPK3	AC113139.1	-0.408781407	3.98E-23	negative
LPIN1	AC113139.1	0.433128581	4.68E-26	postive
TAZ	AC113139.1	0.449179776	4.01E-28	postive
ZNF419	AC005840.4	0.422331664	9.97E-25	postive
PHKG2	AC005840.4	0.689125429	3.84E-77	postive
EGLN2	AC005840.4	0.46868816	8.71E-31	postive
TAZ	AC005840.4	0.625741819	6.35E-60	postive
FANCD2	AC100861.1	0.45966425	1.56E-29	postive
MTOR	AC242426.2	0.470186575	5.35E-31	postive
ZNF419	AC242426.2	0.425539354	4.06E-25	postive
KLHL24	AC242426.2	0.505820268	2.35E-36	postive
TUBE1	AC242426.2	0.43750047	1.31E-26	postive
MAP3K5	AC242426.2	0.441035107	4.63E-27	postive
ALOX12	AC242426.2	0.459712306	1.54E-29	postive
IREB2	AC242426.2	0.606999059	1.45E-55	postive
GABPB1	AC242426.2	0.595409281	5.19E-53	postive
PIK3CA	AC242426.2	0.529190695	3.15E-40	postive
ATG7	AC242426.2	0.409131149	3.63E-23	postive
MAPK8	AC242426.2	0.527524264	6.08E-40	postive
LINC00472	AC242426.2	0.651018058	2.70E-66	postive
PRKAA2	AC242426.2	0.435229426	2.54E-26	postive
PRKAA1	AC242426.2	0.457731472	2.87E-29	postive
LPIN1	AC242426.2	0.400095456	3.88E-22	postive
ATM	AC242426.2	0.666971447	1.22E-70	postive
FBXW7	AC242426.2	0.452597692	1.41E-28	postive
TFAP2C	VASH1-AS1	0.545422333	4.22E-43	postive
HBA1	VASH1-AS1	0.642403985	4.68E-64	postive
HELLS	AC008966.2	0.412647527	1.41E-23	postive
TUBE1	AC008966.2	0.424522543	5.41E-25	postive
ALOX12	AC008966.2	0.425313078	4.33E-25	postive
ATM	AC008966.2	0.401349846	2.80E-22	postive
HELLS	AC022558.1	0.543961261	7.76E-43	postive
ZNF419	AC022558.1	0.441574038	3.95E-27	postive
TUBE1	AC022558.1	0.499654989	2.20E-35	postive

ALOX12	AC022558.1	0.543430928	9.68E-43	postive
IREB2	AC022558.1	0.429863584	1.19E-25	postive
GABPB1	AC022558.1	0.579707225	1.03E-49	postive
MAPK8	AC022558.1	0.440267664	5.81E-27	postive
LINC00472	AC022558.1	0.727376694	7.27E-90	postive
LPIN1	AC022558.1	0.410190231	2.74E-23	postive
ATM	AC022558.1	0.674160183	1.09E-72	postive
FBXW7	AC022558.1	0.512475152	1.99E-37	postive
MTOR	FGD5-AS1	0.419102585	2.44E-24	postive
ISCU	FGD5-AS1	0.453178532	1.18E-28	postive
ACSL3	FGD5-AS1	0.547110374	2.08E-43	postive
HIF1A	FGD5-AS1	0.483707011	5.88E-33	postive
CA9	FGD5-AS1	-0.474219687	1.42E-31	negative
CHMP5	FGD5-AS1	0.403156435	1.75E-22	postive
OXSR1	FGD5-AS1	0.435178735	2.58E-26	postive
KLHL24	FGD5-AS1	0.553206049	1.55E-44	postive
EIF2S1	FGD5-AS1	0.410115003	2.79E-23	postive
EIF2AK4	FGD5-AS1	0.458312358	2.39E-29	postive
IREB2	FGD5-AS1	0.482764791	8.10E-33	postive
PIK3CA	FGD5-AS1	0.557502142	2.42E-45	postive
ACSL4	FGD5-AS1	0.440328378	5.71E-27	postive
KRAS	FGD5-AS1	0.495251729	1.06E-34	postive
ATG5	FGD5-AS1	0.512619909	1.88E-37	postive
ATG7	FGD5-AS1	0.428810639	1.61E-25	postive
ATG3	FGD5-AS1	0.437757342	1.22E-26	postive
GABARAPL1	FGD5-AS1	0.521829612	5.63E-39	postive
PRKAA1	FGD5-AS1	0.47163675	3.33E-31	postive
BAP1	FGD5-AS1	0.55135835	3.43E-44	postive
TGFBR1	FGD5-AS1	0.464337429	3.54E-30	postive
LPIN1	FGD5-AS1	0.572718406	2.66E-48	postive
CISD2	LINC00526	0.449491625	3.65E-28	postive
PEBP1	LINC00526	0.51574656	5.79E-38	postive
NCF2	AC004921.1	0.409483523	3.30E-23	postive
HAMP	AC004921.1	0.407434619	5.69E-23	postive
SETD1B	CTBP1-DT	0.427494961	2.34E-25	postive
SP1	CTBP1-DT	0.424153534	6.00E-25	postive
RIPK1	CTBP1-DT	0.413900317	1.01E-23	postive
BECN1	CTBP1-DT	0.431132463	8.30E-26	postive
YY1AP1	CTBP1-DT	0.445746209	1.13E-27	postive
PHKG2	BCRP3	0.42474954	5.07E-25	postive
TAZ	BCRP3	0.504345136	4.03E-36	postive
ZNF419	AF111169.3	0.521938181	5.40E-39	postive
TUBE1	AF111169.3	0.560505199	6.47E-46	postive
SETD1B	AF111169.3	0.475532992	9.20E-32	postive
ALOX12	AF111169.3	0.618950669	2.61E-58	postive
GABPB1	AF111169.3	0.538319724	7.97E-42	postive
ZEB1	AF111169.3	0.427451083	2.37E-25	postive
ATM	AF111169.3	0.613053713	6.11E-57	postive
FBXW7	AF111169.3	0.549251047	8.40E-44	postive
HELLS	N4BP2L2-IT2	0.524467822	2.02E-39	postive
ZNF419	N4BP2L2-IT2	0.438301117	1.04E-26	postive
KLHL24	N4BP2L2-IT2	0.403125186	1.77E-22	postive
TUBE1	N4BP2L2-IT2	0.535176276	2.86E-41	postive
ALOX12	N4BP2L2-IT2	0.500916217	1.40E-35	postive
GABPB1	N4BP2L2-IT2	0.615121701	2.04E-57	postive
MAPK8	N4BP2L2-IT2	0.420613924	1.61E-24	postive
LINC00472	N4BP2L2-IT2	0.786443748	1.88E-114	postive
ATM	N4BP2L2-IT2	0.731630925	2.05E-91	postive

FBXW7	N4BP2L2-IT2	0.528605894	3.97E-40	postive
TUBE1	MIR3142HG	0.412730649	1.38E-23	postive
ALOX12	MIR3142HG	0.486875374	1.98E-33	postive
ATM	MIR3142HG	0.406091685	8.12E-23	postive
HELLS	Z93930.3	0.51043868	4.26E-37	postive
ZNF419	Z93930.3	0.428235745	1.90E-25	postive
TUBE1	Z93930.3	0.505911184	2.27E-36	postive
ALOX12	Z93930.3	0.427793176	2.15E-25	postive
GABPB1	Z93930.3	0.587751016	2.22E-51	postive
LINC00472	Z93930.3	0.757118723	2.31E-101	postive
ATM	Z93930.3	0.701794906	3.94E-81	postive
FBXW7	Z93930.3	0.534958329	3.13E-41	postive
ISCU	AC024575.1	0.457703713	2.89E-29	postive
ATG4D	AC024575.1	0.56112704	4.92E-46	postive
GABARAPL2	AC024575.1	0.448315265	5.22E-28	postive
TUBE1	TMEM9B-AS	0.417701691	3.58E-24	postive
ALOX12	TMEM9B-AS	0.494351404	1.46E-34	postive
LPIN1	TMEM9B-AS	0.402426285	2.12E-22	postive
ATM	TMEM9B-AS	0.402864958	1.89E-22	postive
HELLS	U73169.1	0.537148309	1.29E-41	postive
ZNF419	U73169.1	0.409086527	3.67E-23	postive
TUBE1	U73169.1	0.509878097	5.24E-37	postive
ALOX12	U73169.1	0.499767547	2.12E-35	postive
GABPB1	U73169.1	0.575210759	8.42E-49	postive
ATG7	U73169.1	0.437074302	1.49E-26	postive
MAPK8	U73169.1	0.411442081	1.96E-23	postive
LINC00472	U73169.1	0.638157253	5.59E-63	postive
ATM	U73169.1	0.74430031	3.28E-96	postive
FBXW7	U73169.1	0.546652368	2.52E-43	postive
HELLS	AC012435.3	0.510974557	3.48E-37	postive
ZNF419	AC012435.3	0.423981328	6.29E-25	postive
KLHL24	AC012435.3	0.447970134	5.80E-28	postive
TUBE1	AC012435.3	0.503910234	4.72E-36	postive
ALOX12	AC012435.3	0.466880014	1.56E-30	postive
IREB2	AC012435.3	0.41301596	1.28E-23	postive
GABPB1	AC012435.3	0.558975491	1.27E-45	postive
MAPK8	AC012435.3	0.42996785	1.16E-25	postive
LINC00472	AC012435.3	0.819816119	3.98E-132	postive
ATM	AC012435.3	0.674902643	6.66E-73	postive
FBXW7	AC012435.3	0.475892706	8.17E-32	postive
FANCD2	AC022400.1	0.401727359	2.54E-22	postive
ALOX12	AC022400.1	0.508087489	1.02E-36	postive
TFAP2C	AC022400.1	0.424441611	5.53E-25	postive
FBXW7	DTNB-AS1	0.447993133	5.76E-28	postive
TFAP2C	AC018761.1	0.586910358	3.33E-51	postive
HBA1	AC018761.1	0.578137994	2.15E-49	postive
OTUB1	ARIH2OS	0.413024441	1.28E-23	postive
BAP1	ARIH2OS	0.406620613	7.06E-23	postive
HSF1	VPS13B-DT	0.514755778	8.42E-38	postive
NRAS	VPS13B-DT	-0.417067254	4.26E-24	negative
NCOA4	VPS13B-DT	-0.424822029	4.97E-25	negative
PHKG2	VPS13B-DT	0.548988229	9.39E-44	postive
ATG4D	VPS13B-DT	0.424414078	5.58E-25	postive
ANO6	VPS13B-DT	-0.437027072	1.51E-26	negative
TAZ	VPS13B-DT	0.557198719	2.76E-45	postive
HELLS	AC139887.1	0.502219951	8.73E-36	postive
ZNF419	AC139887.1	0.425130509	4.56E-25	postive
TUBE1	AC139887.1	0.492709913	2.60E-34	postive

ALOX12	AC139887.1	0.566323624	4.86E-47	postive
GABPB1	AC139887.1	0.52139995	6.64E-39	postive
LINC00472	AC139887.1	0.591646031	3.33E-52	postive
ATM	AC139887.1	0.706831692	8.93E-83	postive
FBXW7	AC139887.1	0.4810322	1.46E-32	postive
TFAP2C	AL109947.1	0.551491907	3.24E-44	postive
HBA1	AL109947.1	0.501500563	1.13E-35	postive
DUOX1	AL109947.1	0.41314302	1.24E-23	postive
HELLS	AL590723.1	0.544383239	6.51E-43	postive
TUBE1	AL590723.1	0.421876865	1.13E-24	postive
GABPB1	AL590723.1	0.578108174	2.18E-49	postive
PIK3CA	AL590723.1	0.401573317	2.65E-22	postive
ATG7	AL590723.1	0.463626536	4.44E-30	postive
MAPK8	AL590723.1	0.407492322	5.61E-23	postive
LINC00472	AL590723.1	0.728401028	3.10E-90	postive
ATM	AL590723.1	0.719869558	3.35E-87	postive
FBXW7	AL590723.1	0.476547376	6.57E-32	postive
HELLS	AL080317.1	0.585595126	6.28E-51	postive
TUBE1	AL080317.1	0.488365635	1.18E-33	postive
ALOX12	AL080317.1	0.426534847	3.07E-25	postive
GABPB1	AL080317.1	0.4740453	1.51E-31	postive
LINC00472	AL080317.1	0.541631262	2.04E-42	postive
ATM	AL080317.1	0.658250681	3.12E-68	postive
FBXW7	AL080317.1	0.45792819	2.69E-29	postive
HELLS	AC130650.2	0.51617773	4.91E-38	postive
ZNF419	AC130650.2	0.445569547	1.20E-27	postive
KLHL24	AC130650.2	0.474527276	1.28E-31	postive
TUBE1	AC130650.2	0.50082392	1.45E-35	postive
ALOX12	AC130650.2	0.408798471	3.96E-23	postive
GABPB1	AC130650.2	0.619390087	2.06E-58	postive
MAPK8	AC130650.2	0.403619286	1.55E-22	postive
LINC00472	AC130650.2	0.810691046	6.01E-127	postive
ATM	AC130650.2	0.62972437	6.89E-61	postive
FBXW7	AC130650.2	0.527483433	6.18E-40	postive
FANCD2	AC007773.1	0.466283201	1.90E-30	postive
HELLS	AC007773.1	0.528105963	4.84E-40	postive
PSAT1	AC007773.1	0.416328751	5.22E-24	postive
HELLS	AL021368.2	0.499833878	2.07E-35	postive
ZNF419	AL021368.2	0.580654023	6.60E-50	postive
VEGFA	AL021368.2	0.458092155	2.56E-29	postive
TUBE1	AL021368.2	0.72738932	7.19E-90	postive
SETD1B	AL021368.2	0.415693561	6.20E-24	postive
ALOX12	AL021368.2	0.702578563	2.20E-81	postive
IREB2	AL021368.2	0.44113857	4.49E-27	postive
SP1	AL021368.2	0.459436759	1.68E-29	postive
GABPB1	AL021368.2	0.631280247	2.87E-61	postive
ZEB1	AL021368.2	0.407249579	5.98E-23	postive
MAPK8	AL021368.2	0.558548971	1.53E-45	postive
LINC00472	AL021368.2	0.50040595	1.68E-35	postive
ATM	AL021368.2	0.795373549	7.40E-119	postive
FBXW7	AL021368.2	0.567736464	2.57E-47	postive
HMGB1	AC011451.1	0.467403492	1.32E-30	postive
TFR2	IGFL2-AS1	0.444496728	1.65E-27	postive
IDH1	IGFL2-AS1	0.518959048	1.70E-38	postive
ZNF419	CD27-AS1	0.407921641	5.00E-23	postive
ALOX12	CD27-AS1	0.436812295	1.60E-26	postive
PHKG2	CD27-AS1	0.553730701	1.24E-44	postive
ULK1	CD27-AS1	0.47222417	2.74E-31	postive

TAZ	CD27-AS1	0.568109876	2.17E-47	postive
HELLS	AL359095.1	0.529112663	3.25E-40	postive
TUBE1	AL359095.1	0.43727221	1.40E-26	postive
ALOX12	AL359095.1	0.423140642	7.96E-25	postive
GABPB1	AL359095.1	0.499198288	2.60E-35	postive
ATG7	AL359095.1	0.430876781	8.93E-26	postive
LINC00472	AL359095.1	0.524228433	2.22E-39	postive
ATM	AL359095.1	0.69160274	6.61E-78	postive
FBXW7	AL359095.1	0.403168897	1.75E-22	postive
ZNF419	AC072061.1	0.427911826	2.08E-25	postive
ALOX12	AC072061.1	0.401438814	2.74E-22	postive
TFAP2C	AL603832.1	0.562648058	2.51E-46	postive
HBA1	AL603832.1	0.505130528	3.02E-36	postive
PHKG2	AC107464.2	0.563771613	1.52E-46	postive
TAZ	AC107464.2	0.441923785	3.56E-27	postive
ZNF419	AL159169.2	0.577915192	2.39E-49	postive
TUBE1	AL159169.2	0.514211564	1.03E-37	postive
ALOX12	AL159169.2	0.484537507	4.43E-33	postive
GABPB1	AL159169.2	0.516650913	4.10E-38	postive
PHKG2	AL159169.2	0.403512471	1.60E-22	postive
SOCS1	AL159169.2	0.436013706	2.03E-26	postive
TAZ	AL159169.2	0.506435831	1.87E-36	postive
FBXW7	AL159169.2	0.516135979	4.99E-38	postive
ALB	TCL6	0.611456903	1.42E-56	postive
GPX2	TCL6	0.456469904	4.25E-29	postive
TFR2	TCL6	0.446816771	8.22E-28	postive
CDO1	TCL6	0.558234532	1.75E-45	postive
KLHL24	RMST	0.408723222	4.05E-23	postive
FBXW7	RMST	0.46608163	2.02E-30	postive
STMN1	AL441992.1	0.432916693	4.97E-26	postive
CDKN2A	AL441992.1	0.412548815	1.45E-23	postive
HELLS	AC007496.1	0.524753671	1.81E-39	postive
GABPB1	AC007496.1	0.519762622	1.25E-38	postive
ATG7	AC007496.1	0.476776322	6.09E-32	postive
LINC00472	AC007496.1	0.621336664	7.15E-59	postive
ATM	AC007496.1	0.71295787	7.99E-85	postive
FBXW7	AC007496.1	0.460357211	1.25E-29	postive
FANCD2	ASB16-AS1	0.433424852	4.29E-26	postive
BRD4	ASB16-AS1	0.495877999	8.50E-35	postive
ZNF419	ASB16-AS1	0.460965039	1.04E-29	postive
SETD1B	ASB16-AS1	0.462005179	7.45E-30	postive
ALOX12	ASB16-AS1	0.570670404	6.79E-48	postive
PHKG2	ASB16-AS1	0.620934001	8.90E-59	postive
TAZ	ASB16-AS1	0.649326674	7.52E-66	postive
ZNF419	AL590666.1	0.480271306	1.88E-32	postive
VEGFA	AL590666.1	0.416892879	4.47E-24	postive
TUBE1	AL590666.1	0.515867438	5.53E-38	postive
SETD1B	AL590666.1	0.425759331	3.82E-25	postive
ALOX12	AL590666.1	0.588767711	1.36E-51	postive
GABPB1	AL590666.1	0.418384428	2.97E-24	postive
ATM	AL590666.1	0.41598211	5.73E-24	postive
TAZ	AL590666.1	0.463508297	4.61E-30	postive
FBXW7	AL590666.1	0.457009826	3.59E-29	postive
KLHL24	AC027117.1	0.644945926	1.04E-64	postive
PIK3CA	AC027117.1	0.411024109	2.19E-23	postive
LINC00472	AC027117.1	0.837133242	8.17E-143	postive
LPIN1	AC027117.1	0.513648196	1.28E-37	postive
KLHL24	AC040174.2	0.57254548	2.88E-48	postive

GABARAPL1	AC040174.2	0.401282675	2.85E-22	postive
LINC00472	AC040174.2	0.802104172	2.52E-122	postive
LPIN1	AC040174.2	0.454052901	9.00E-29	postive
FANCD2	AC135178.4	0.425303463	4.34E-25	postive
ALOX12	AC135178.4	0.439555699	7.17E-27	postive
TFAP2C	AC135178.4	0.541475924	2.18E-42	postive
HBA1	AC135178.4	0.459465196	1.66E-29	postive
DUOX1	AC135178.4	0.44751099	6.66E-28	postive
CA9	BX322562.1	0.444439883	1.68E-27	postive
MAPK14	BX322562.1	-0.457138961	3.45E-29	negative
EIF2AK4	BX322562.1	-0.467436292	1.31E-30	negative
IREB2	BX322562.1	-0.436665399	1.68E-26	negative
PIK3CA	BX322562.1	-0.46174711	8.08E-30	negative
NRAS	BX322562.1	-0.406488761	7.31E-23	negative
HRAS	BX322562.1	0.510847938	3.65E-37	postive
ATG5	BX322562.1	-0.407402865	5.74E-23	negative
NCOA4	BX322562.1	-0.458874895	2.00E-29	negative
PHKG2	BX322562.1	0.435598676	2.29E-26	postive
ANO6	BX322562.1	-0.412966384	1.30E-23	negative
TLR4	BX322562.1	-0.446564885	8.87E-28	negative
TAZ	BX322562.1	0.460964401	1.04E-29	postive
MTDH	BX322562.1	-0.442893098	2.67E-27	negative
ZEB1	AP001922.6	0.465654979	2.32E-30	postive
EPAS1	AP001922.6	0.468526635	9.18E-31	postive
ISCU	AC069224.1	0.460089732	1.37E-29	postive
ZNF419	AC069224.1	0.400838277	3.20E-22	postive
KLHL24	AC069224.1	0.604786936	4.54E-55	postive
GABARAPL1	AC069224.1	0.553340766	1.46E-44	postive
LINC00472	AC069224.1	0.50109253	1.31E-35	postive
LPIN1	AC069224.1	0.482819366	7.95E-33	postive
FANCD2	ZNF232-AS1	0.417200408	4.11E-24	postive
ASNS	ZNF232-AS1	0.405397961	9.74E-23	postive
SLC2A6	ZNF232-AS1	0.416142588	5.49E-24	postive
PHKG2	ZNF232-AS1	0.429588525	1.29E-25	postive
EGLN2	ZNF232-AS1	0.407142678	6.15E-23	postive
TFAP2C	AL121821.2	0.515266134	6.94E-38	postive
LPIN1	AL121821.2	0.411293322	2.04E-23	postive
HELLS	AC131934.1	0.510944208	3.52E-37	postive
ZNF419	AC131934.1	0.426410334	3.18E-25	postive
TUBE1	AC131934.1	0.435840148	2.13E-26	postive
ALOX12	AC131934.1	0.490857782	4.98E-34	postive
GABPB1	AC131934.1	0.531057711	1.50E-40	postive
LINC00472	AC131934.1	0.700205516	1.28E-80	postive
ATM	AC131934.1	0.63017822	5.34E-61	postive
FBXW7	AC131934.1	0.453141546	1.19E-28	postive
KLHL24	MFF-DT	0.50234253	8.35E-36	postive
GABPB1	MFF-DT	0.425322361	4.32E-25	postive
PIK3CA	MFF-DT	0.404580397	1.21E-22	postive
LINC00472	MFF-DT	0.785586466	4.85E-114	postive
LPIN1	MFF-DT	0.422502375	9.51E-25	postive
ATM	MFF-DT	0.402175059	2.26E-22	postive
HELLS	AC009495.2	0.41878343	2.66E-24	postive
TUBE1	AC009495.2	0.603859215	7.31E-55	postive
ALOX12	AC009495.2	0.547835951	1.53E-43	postive
GABPB1	AC009495.2	0.42485542	4.93E-25	postive
ATM	AC009495.2	0.639028713	3.37E-63	postive
FBXW7	AC009495.2	0.45088484	2.39E-28	postive
CHAC1	ASMTL-AS1	0.408836091	3.93E-23	postive

ZNF419	ASMTL-AS1	0.403854675	1.46E-22	postive
PHKG2	ASMTL-AS1	0.593235362	1.52E-52	postive
TAZ	ASMTL-AS1	0.655721194	1.50E-67	postive
MAP1LC3A	AC004241.3	0.42303814	8.19E-25	postive
LINC00472	AC004241.3	0.565315595	7.64E-47	postive
LPIN1	AC004241.3	0.517732686	2.72E-38	postive
KLHL24	MIR222HG	0.531772083	1.13E-40	postive
KRAS	MIR222HG	0.409759262	3.07E-23	postive
LINC00472	MIR222HG	0.797961421	3.56E-120	postive
LPIN1	MIR222HG	0.486358739	2.37E-33	postive
AKR1C2	LINC00942	0.407319979	5.87E-23	postive
NQO1	LINC00942	0.425522442	4.08E-25	postive
FANCD2	AC009690.2	0.411635979	1.86E-23	postive
HELLS	AC009690.2	0.50158034	1.10E-35	postive
ZNF419	AC009690.2	0.592748867	1.94E-52	postive
TUBE1	AC009690.2	0.59809384	1.36E-53	postive
SETD1B	AC009690.2	0.404831454	1.13E-22	postive
ALOX12	AC009690.2	0.694453167	8.55E-79	postive
GABPB1	AC009690.2	0.454161573	8.70E-29	postive
PHKG2	AC009690.2	0.499245713	2.55E-35	postive
LINC00472	AC009690.2	0.41913698	2.42E-24	postive
ATM	AC009690.2	0.500451392	1.65E-35	postive
TAZ	AC009690.2	0.563414032	1.79E-46	postive
FBXW7	AC009690.2	0.525764366	1.22E-39	postive
TUBE1	AL117336.2	0.475701864	8.70E-32	postive
ALOX12	AL117336.2	0.519901401	1.18E-38	postive
TAZ	AL117336.2	0.443749592	2.07E-27	postive
VEGFA	AC091849.2	0.451927444	1.73E-28	postive
ALOX12	AC091849.2	0.40361551	1.55E-22	postive
SETD1B	CENATAC-D	0.457600292	2.99E-29	postive
ALOX12	CENATAC-D	0.452144221	1.62E-28	postive
IREB2	CENATAC-D	0.462808215	5.77E-30	postive
MAPK8	CENATAC-D	0.476352551	7.01E-32	postive
ATM	CENATAC-D	0.472024744	2.93E-31	postive
YY1AP1	CENATAC-D	0.433737861	3.92E-26	postive
HELLS	WARS2-AS1	0.520671237	8.80E-39	postive
ZNF419	WARS2-AS1	0.507946924	1.07E-36	postive
TUBE1	WARS2-AS1	0.588250108	1.74E-51	postive
ALOX12	WARS2-AS1	0.563611205	1.64E-46	postive
GABPB1	WARS2-AS1	0.590192882	6.78E-52	postive
MAPK8	WARS2-AS1	0.465073949	2.80E-30	postive
LINC00472	WARS2-AS1	0.579298056	1.25E-49	postive
ATM	WARS2-AS1	0.768511495	3.23E-106	postive
FBXW7	WARS2-AS1	0.540862889	2.80E-42	postive
TFAP2C	LINC02289	0.412968305	1.30E-23	postive
HBA1	LINC02289	0.409930281	2.93E-23	postive
FANCD2	ARHGEF2-AS	0.46204985	7.34E-30	postive
HELLS	ARHGEF2-AS	0.46861786	8.91E-31	postive
ZNF419	ARHGEF2-AS	0.412038719	1.67E-23	postive
ALOX12	ARHGEF2-AS	0.468955915	7.98E-31	postive
PHKG2	ARHGEF2-AS	0.561909856	3.48E-46	postive
TAZ	ARHGEF2-AS	0.611204937	1.62E-56	postive
ALOX12	AL121845.4	0.466600539	1.71E-30	postive
MIOX	AL121845.4	0.533865168	4.86E-41	postive
ZNF419	ZNF790-AS1	0.465224773	2.66E-30	postive
LINC00472	ZNF790-AS1	0.451964622	1.71E-28	postive
ALOX12	AC127521.1	0.603417425	9.17E-55	postive
TAZ	AC127521.1	0.435277316	2.51E-26	postive

VEGFA	C6orf223	0.453658563	1.02E-28	postive
FANCD2	SLBP-DT	0.416355001	5.18E-24	postive
BRD4	SLBP-DT	0.431640354	7.18E-26	postive
ZNF419	SLBP-DT	0.48036288	1.83E-32	postive
ALOX12	SLBP-DT	0.420382384	1.71E-24	postive
NCOA4	SLBP-DT	-0.455671011	5.45E-29	negative
PHKG2	SLBP-DT	0.722784798	3.17E-88	postive
MAPK1	SLBP-DT	-0.435362878	2.45E-26	negative
TAZ	SLBP-DT	0.764285704	2.20E-104	postive
BNIP3	LINC02275	0.416001793	5.70E-24	postive
ALOX12	LINC02275	0.455651611	5.48E-29	postive
ATM	LINC02275	0.437082189	1.48E-26	postive
HELLS	AC092301.1	0.481360386	1.30E-32	postive
BRD4	AC092301.1	0.438566256	9.60E-27	postive
ZNF419	AC092301.1	0.560651395	6.07E-46	postive
TUBE1	AC092301.1	0.584009576	1.34E-50	postive
SETD1B	AC092301.1	0.461501808	8.73E-30	postive
ALOX12	AC092301.1	0.620022194	1.46E-58	postive
GABPB1	AC092301.1	0.503404392	5.68E-36	postive
LINC00472	AC092301.1	0.524632493	1.89E-39	postive
ATM	AC092301.1	0.581979478	3.53E-50	postive
TAZ	AC092301.1	0.428986148	1.53E-25	postive
FBXW7	AC092301.1	0.492284974	3.02E-34	postive
TFAP2C	AC063943.1	0.478029049	4.00E-32	postive
HBA1	AP003032.1	0.693772413	1.40E-78	postive
HRAS	AP003032.1	0.554619547	8.44E-45	postive
EGLN2	AP003032.1	0.702395568	2.52E-81	postive
FANCD2	PRDX6-AS1	0.46054018	1.18E-29	postive
HELLS	PRDX6-AS1	0.554407229	9.25E-45	postive
ALOX12	PRDX6-AS1	0.510449747	4.24E-37	postive
TFAP2C	PRDX6-AS1	0.443760557	2.06E-27	postive
HBA1	PRDX6-AS1	0.41088478	2.27E-23	postive
GABPB1	PRDX6-AS1	0.409869231	2.98E-23	postive
DUOX1	PRDX6-AS1	0.479821109	2.19E-32	postive
ATM	PRDX6-AS1	0.427701999	2.21E-25	postive
FBXW7	PRDX6-AS1	0.425433935	4.19E-25	postive
HELLS	AC009120.2	0.404893415	1.11E-22	postive
ZNF419	AC009120.2	0.592103389	2.66E-52	postive
TUBE1	AC009120.2	0.662686174	1.90E-69	postive
SETD1B	AC009120.2	0.457632724	2.96E-29	postive
ALOX12	AC009120.2	0.622114488	4.67E-59	postive
GABPB1	AC009120.2	0.457067213	3.53E-29	postive
PHKG2	AC009120.2	0.553100885	1.62E-44	postive
LINC00472	AC009120.2	0.459203361	1.81E-29	postive
ATM	AC009120.2	0.442460187	3.03E-27	postive
TAZ	AC009120.2	0.612056563	1.03E-56	postive
FBXW7	AC009120.2	0.438835202	8.87E-27	postive
RB1	AC004112.1	0.545079308	4.87E-43	postive
MTOR	AC004112.1	0.444454624	1.67E-27	postive
OTUB1	AC004112.1	-0.427346251	2.44E-25	negative
OXSR1	AC004112.1	0.40660331	7.09E-23	postive
KLHL24	AC004112.1	0.413154929	1.23E-23	postive
TUBE1	AC004112.1	0.417642999	3.64E-24	postive
RPL8	AC004112.1	-0.417631546	3.65E-24	negative
MAP3K5	AC004112.1	0.486908061	1.96E-33	postive
MAPK14	AC004112.1	0.458655609	2.14E-29	postive
IREB2	AC004112.1	0.646385288	4.41E-65	postive
SP1	AC004112.1	0.493470078	1.99E-34	postive

GABPB1	AC004112.1	0.410832662	2.30E-23	postive
PIK3CA	AC004112.1	0.575237296	8.32E-49	postive
NRAS	AC004112.1	0.435269301	2.52E-26	postive
KRAS	AC004112.1	0.482605008	8.55E-33	postive
NCOA4	AC004112.1	0.436895174	1.57E-26	postive
MAPK1	AC004112.1	0.46656388	1.73E-30	postive
MAPK8	AC004112.1	0.576129401	5.50E-49	postive
MAPK9	AC004112.1	0.453447072	1.09E-28	postive
LINC00472	AC004112.1	0.504115391	4.38E-36	postive
PRKAA2	AC004112.1	0.637754873	7.06E-63	postive
PRKAA1	AC004112.1	0.545857202	3.52E-43	postive
TLR4	AC004112.1	0.502173628	8.88E-36	postive
ATM	AC004112.1	0.706640769	1.03E-82	postive
SIRT1	AC004112.1	0.455809048	5.22E-29	postive
HELLS	AC010531.5	0.436454865	1.78E-26	postive
ZNF419	AC010531.5	0.401175535	2.93E-22	postive
TUBE1	AC010531.5	0.456368469	4.39E-29	postive
ALOX12	AC010531.5	0.591358909	3.83E-52	postive
GABPB1	AC010531.5	0.429473435	1.33E-25	postive
MAPK8	AC010531.5	0.401065534	3.02E-22	postive
ATM	AC010531.5	0.585617018	6.21E-51	postive
FBXW7	AC010531.5	0.445096194	1.38E-27	postive
KLHL24	AL136980.1	0.503699474	5.10E-36	postive
TUBE1	AL136980.1	0.418270166	3.07E-24	postive
GABPB1	AL136980.1	0.528986854	3.41E-40	postive
PIK3CA	AL136980.1	0.420438822	1.69E-24	postive
LINC00472	AL136980.1	0.916016612	3.51E-215	postive
ATM	AL136980.1	0.542194313	1.62E-42	postive
ISCU	AC116312.1	0.484689	4.20E-33	postive
CHAC1	AC116312.1	0.400413241	3.57E-22	postive
DDIT3	AC116312.1	0.477401894	4.94E-32	postive
GPT2	AC116312.1	0.406569456	7.16E-23	postive
ATG4D	AC116312.1	0.546692587	2.48E-43	postive
MAP1LC3A	AC116312.1	0.478154174	3.84E-32	postive
GABARAPL1	AC116312.1	0.601187232	2.85E-54	postive
WIPI2	AC116312.1	0.420391019	1.71E-24	postive
LPIN1	AC116312.1	0.450491164	2.69E-28	postive
ZNF419	AC092542.1	0.404725007	1.16E-22	postive
VEGFA	AC092542.1	0.42544191	4.18E-25	postive
TUBE1	AC092542.1	0.460686665	1.13E-29	postive
ALOX12	AC092542.1	0.442753756	2.78E-27	postive
GABPB1	AC092542.1	0.49645263	6.93E-35	postive
ZEB1	AC092542.1	0.536779504	1.49E-41	postive
MAPK8	AC092542.1	0.457849426	2.76E-29	postive
ATM	AC092542.1	0.553179615	1.57E-44	postive
FBXW7	AC092542.1	0.490495296	5.65E-34	postive
BRD4	AC138028.4	0.431999447	6.47E-26	postive
ZNF419	AC138028.4	0.582856975	2.32E-50	postive
VEGFA	AC138028.4	0.540369527	3.44E-42	postive
TUBE1	AC138028.4	0.53166997	1.17E-40	postive
SETD1B	AC138028.4	0.567404988	2.99E-47	postive
ALOX12	AC138028.4	0.664632065	5.49E-70	postive
ATM	AC138028.4	0.422589243	9.28E-25	postive
YY1AP1	AC138028.4	0.50006002	1.91E-35	postive
TAZ	AC138028.4	0.503173837	6.17E-36	postive
FBXW7	AC138028.4	0.410425275	2.57E-23	postive
HELLS	AL031768.1	0.535075209	2.98E-41	postive
TUBE1	AL031768.1	0.471499693	3.48E-31	postive

ALOX12	AL031768.1	0.403515957	1.60E-22	postive
GABPB1	AL031768.1	0.554237342	9.95E-45	postive
LINC00472	AL031768.1	0.768218902	4.34E-106	postive
ATM	AL031768.1	0.663199917	1.37E-69	postive
FBXW7	AL031768.1	0.464253749	3.64E-30	postive
ZNF419	AC120053.1	0.495246449	1.06E-34	postive
TUBE1	AC120053.1	0.549657911	7.07E-44	postive
ALOX12	AC120053.1	0.499901629	2.02E-35	postive
PHKG2	AC120053.1	0.532232319	9.36E-41	postive
TAZ	AC120053.1	0.576273994	5.14E-49	postive
LINC00472	AC025165.5	0.580747241	6.32E-50	postive
ATM	AC025165.5	0.436749068	1.63E-26	postive
MTOR	KIAA1671-AS	0.533506845	5.61E-41	postive
IREB2	KIAA1671-AS	0.482084762	1.02E-32	postive
ATG7	KIAA1671-AS	0.44186651	3.62E-27	postive
MAPK8	KIAA1671-AS	0.423509896	7.18E-25	postive
LINC00472	KIAA1671-AS	0.455512746	5.72E-29	postive
PRKAA2	KIAA1671-AS	0.431940793	6.58E-26	postive
TLR4	KIAA1671-AS	0.402844816	1.90E-22	postive
ATM	KIAA1671-AS	0.597756692	1.61E-53	postive
HSPA5	AP000866.5	-0.411726912	1.81E-23	negative
TUBE1	AP000866.5	0.451377481	2.05E-28	postive
ALOX12	AP000866.5	0.448451879	5.01E-28	postive
ZNF419	AC095057.3	0.423982481	6.29E-25	postive
TUBE1	AC095057.3	0.484401207	4.64E-33	postive
NCOA4	AC095057.3	-0.402452295	2.11E-22	negative
PHKG2	AC095057.3	0.580387611	7.49E-50	postive
BECN1	AC095057.3	-0.414866246	7.76E-24	negative
TAZ	AC095057.3	0.674799333	7.13E-73	postive
ISCU	LINC01213	0.416929503	4.43E-24	postive
GABARAPL1	LINC01213	0.50298901	6.60E-36	postive
LPIN1	LINC01213	0.446296772	9.62E-28	postive
RB1	ARRDC1-AS1	-0.417250844	4.05E-24	negative
HSF1	ARRDC1-AS1	0.538003169	9.07E-42	postive
OTUB1	ARRDC1-AS1	0.446314844	9.56E-28	postive
ZNF419	ARRDC1-AS1	0.41703359	4.30E-24	postive
SLC2A8	ARRDC1-AS1	0.452789762	1.33E-28	postive
NRAS	ARRDC1-AS1	-0.472744086	2.31E-31	negative
PHKG2	ARRDC1-AS1	0.656764217	7.88E-68	postive
ATG4D	ARRDC1-AS1	0.574708738	1.06E-48	postive
MAP1LC3A	ARRDC1-AS1	0.449762958	3.36E-28	postive
MAPK1	ARRDC1-AS1	-0.437383035	1.36E-26	negative
ANO6	ARRDC1-AS1	-0.493078104	2.29E-34	negative
TAZ	ARRDC1-AS1	0.623009692	2.86E-59	postive
VEGFA	AC087477.2	0.433085662	4.73E-26	postive
TUBE1	AC087477.2	0.526359116	9.62E-40	postive
ALOX12	AC087477.2	0.479367706	2.56E-32	postive
ZNF419	AC009090.3	0.524365506	2.10E-39	postive
KLHL24	AC009090.3	0.448039328	5.68E-28	postive
TUBE1	AC009090.3	0.592216154	2.52E-52	postive
ALOX12	AC009090.3	0.507812778	1.13E-36	postive
GABPB1	AC009090.3	0.483462607	6.39E-33	postive
MAPK8	AC009090.3	0.404803509	1.14E-22	postive
LINC00472	AC009090.3	0.72265673	3.52E-88	postive
ATM	AC009090.3	0.527097538	7.20E-40	postive
FBXW7	AC009090.3	0.408026952	4.87E-23	postive
GPX4	AL049840.3	-0.411828874	1.76E-23	negative
HELLS	AL049840.3	0.404122791	1.36E-22	postive

MTOR	AL049840.3	0.491517387	3.95E-34	postive
ARNTL	AL049840.3	0.402401445	2.13E-22	postive
ZNF419	AL049840.3	0.513949169	1.14E-37	postive
ZFP69B	AL049840.3	0.453553169	1.05E-28	postive
VEGFA	AL049840.3	0.411678992	1.84E-23	postive
TUBE1	AL049840.3	0.501450318	1.15E-35	postive
SETD1B	AL049840.3	0.500375186	1.70E-35	postive
ALOX12	AL049840.3	0.528248065	4.57E-40	postive
IREB2	AL049840.3	0.473926477	1.57E-31	postive
GABPB1	AL049840.3	0.58227772	3.06E-50	postive
PIK3CA	AL049840.3	0.428244086	1.89E-25	postive
ZEB1	AL049840.3	0.601145013	2.92E-54	postive
MAPK8	AL049840.3	0.569727132	1.04E-47	postive
LINC00472	AL049840.3	0.472600529	2.43E-31	postive
TLR4	AL049840.3	0.432324173	5.90E-26	postive
ATM	AL049840.3	0.67560929	4.15E-73	postive
YY1AP1	AL049840.3	0.410661329	2.41E-23	postive
FBXW7	AL049840.3	0.564095779	1.32E-46	postive
HELLS	KANSL1L-AS	0.583614457	1.62E-50	postive
ZNF419	KANSL1L-AS	0.413048586	1.27E-23	postive
TUBE1	KANSL1L-AS	0.570898316	6.12E-48	postive
ALOX12	KANSL1L-AS	0.57575071	6.56E-49	postive
GABPB1	KANSL1L-AS	0.596457603	3.08E-53	postive
MAPK8	KANSL1L-AS	0.450433345	2.74E-28	postive
LINC00472	KANSL1L-AS	0.701776542	4.00E-81	postive
ATM	KANSL1L-AS	0.783371321	5.50E-113	postive
FBXW7	KANSL1L-AS	0.528435141	4.25E-40	postive
FANCD2	AC008764.6	0.461113642	9.88E-30	postive
ALOX12	AC008764.6	0.424589616	5.31E-25	postive
TFAP2C	AC008764.6	0.533205246	6.34E-41	postive
HBA1	AC008764.6	0.439542381	7.20E-27	postive
RPL8	SNHG5	0.484990677	3.79E-33	postive
BECN1	AC009812.4	-0.449333198	3.83E-28	negative
EPAS1	AL390955.2	0.413493951	1.13E-23	postive
HELLS	RERE-AS1	0.498695543	3.11E-35	postive
ZNF419	RERE-AS1	0.422448827	9.65E-25	postive
TUBE1	RERE-AS1	0.517407452	3.08E-38	postive
ALOX12	RERE-AS1	0.505662414	2.49E-36	postive
GABPB1	RERE-AS1	0.565067308	8.54E-47	postive
LINC00472	RERE-AS1	0.726196271	1.93E-89	postive
ATM	RERE-AS1	0.666119848	2.11E-70	postive
FBXW7	RERE-AS1	0.504596032	3.68E-36	postive
PHKG2	AC007383.1	0.479766878	2.23E-32	postive
TAZ	AC007383.1	0.502015543	9.40E-36	postive
BRD4	SEMA3F-AS1	0.448752867	4.57E-28	postive
ZNF419	SEMA3F-AS1	0.617250631	6.52E-58	postive
VEGFA	SEMA3F-AS1	0.493276425	2.13E-34	postive
TUBE1	SEMA3F-AS1	0.624132499	1.54E-59	postive
SETD1B	SEMA3F-AS1	0.511905975	2.46E-37	postive
ALOX12	SEMA3F-AS1	0.636537712	1.43E-62	postive
GABPB1	SEMA3F-AS1	0.531302829	1.36E-40	postive
ATM	SEMA3F-AS1	0.481937997	1.07E-32	postive
TAZ	SEMA3F-AS1	0.476272327	7.20E-32	postive
FBXW7	SEMA3F-AS1	0.544308095	6.72E-43	postive
FANCD2	AC010618.2	0.420857594	1.50E-24	postive
ALOX12	AC010618.2	0.43062429	9.60E-26	postive
TFAP2C	AC010618.2	0.527116421	7.14E-40	postive
HBA1	AC010618.2	0.475677272	8.77E-32	postive

DUOX1	AC010618.2	0.418954819	2.54E-24	postive
BRD4	PKD1P6-NPII	0.40745976	5.66E-23	postive
ZNF419	PKD1P6-NPII	0.413130313	1.24E-23	postive
VEGFA	PKD1P6-NPII	0.551742884	2.91E-44	postive
TUBE1	PKD1P6-NPII	0.427184293	2.56E-25	postive
SETD1B	PKD1P6-NPII	0.479755295	2.24E-32	postive
ALOX12	PKD1P6-NPII	0.536257073	1.85E-41	postive
NCOA4	PKD1P6-NPII	-0.408032246	4.86E-23	negative
PHKG2	PKD1P6-NPII	0.477581616	4.65E-32	postive
TAZ	PKD1P6-NPII	0.613914415	3.87E-57	postive
ZNF419	AC022762.2	0.478545544	3.37E-32	postive
TUBE1	AC022762.2	0.523038486	3.52E-39	postive
ALOX12	AC022762.2	0.543165453	1.08E-42	postive
PHKG2	AC022762.2	0.552906023	1.77E-44	postive
TAZ	AC022762.2	0.67687047	1.78E-73	postive
HELLS	AC069549.1	0.466816984	1.60E-30	postive
GABPB1	AC069549.1	0.436934924	1.55E-26	postive
LINC00472	AC069549.1	0.517338713	3.16E-38	postive
ATM	AC069549.1	0.567577313	2.76E-47	postive
PML	AC012645.3	0.41261598	1.43E-23	postive
ALOX12	AC012645.3	0.465278201	2.62E-30	postive
PHKG2	AC012645.3	0.454717613	7.33E-29	postive
IFNG	AC012645.3	0.454621449	7.55E-29	postive
TAZ	AC012645.3	0.518898865	1.74E-38	postive
NCOA4	AC092757.3	-0.417800653	3.49E-24	negative
PHKG2	AC092757.3	0.474046142	1.51E-31	postive
ULK1	AC092757.3	0.404276273	1.31E-22	postive
HELLS	AC005899.7	0.513812313	1.20E-37	postive
ZNF419	AC005899.7	0.406040427	8.23E-23	postive
TUBE1	AC005899.7	0.483747207	5.80E-33	postive
ALOX12	AC005899.7	0.520800452	8.38E-39	postive
GABPB1	AC005899.7	0.496606391	6.56E-35	postive
LINC00472	AC005899.7	0.471209562	3.83E-31	postive
ATM	AC005899.7	0.645843799	6.09E-65	postive
FBXW7	AC005899.7	0.483094847	7.24E-33	postive
HELLS	AC005730.3	0.412079693	1.65E-23	postive
TFAP2C	AC005730.3	0.509860963	5.28E-37	postive
HBA1	AC005730.3	0.462018564	7.41E-30	postive
DUOX1	AC005730.3	0.440764153	5.02E-27	postive
LINC00472	AC005730.3	0.414021498	9.76E-24	postive
CHMP5	LINC00997	-0.408043214	4.85E-23	negative
STEAP3	LINC00997	0.416126373	5.51E-24	postive
BID	LINC00997	0.413268877	1.20E-23	postive
HELLS	AC108463.3	0.492819437	2.50E-34	postive
ZNF419	AC108463.3	0.409569989	3.23E-23	postive
ZFP69B	AC108463.3	0.405170515	1.03E-22	postive
TUBE1	AC108463.3	0.459945532	1.43E-29	postive
ALOX12	AC108463.3	0.4897046	7.44E-34	postive
GABPB1	AC108463.3	0.540987485	2.66E-42	postive
LINC00472	AC108463.3	0.474556685	1.27E-31	postive
ATM	AC108463.3	0.678115851	7.70E-74	postive
FBXW7	AC108463.3	0.526214266	1.02E-39	postive
GPX4	AC138696.2	0.535394652	2.62E-41	postive
HSPB1	AC138696.2	0.654969662	2.39E-67	postive
IREB2	AC138696.2	-0.431732216	6.99E-26	negative
NOX1	AC138696.2	0.41724258	4.06E-24	postive
PIK3CA	AC138696.2	-0.422017731	1.09E-24	negative
NRAS	AC138696.2	-0.41248158	1.48E-23	negative

KRAS	AC138696.2	-0.405437912	9.64E-23	negative
HRAS	AC138696.2	0.57904388	1.41E-49	postive
NCOA4	AC138696.2	-0.423720994	6.77E-25	negative
PHKG2	AC138696.2	0.522348366	4.60E-39	postive
BECN1	AC138696.2	-0.44447448	1.66E-27	negative
PRKAA1	AC138696.2	-0.451442912	2.01E-28	negative
SIRT1	AC138696.2	-0.453562242	1.05E-28	negative
LONP1	AC138696.2	0.420645617	1.59E-24	postive
HELLS	AL136320.1	0.522064677	5.14E-39	postive
TUBE1	AL136320.1	0.400770574	3.26E-22	postive
GABPB1	AL136320.1	0.498491277	3.35E-35	postive
ATG7	AL136320.1	0.473061449	2.08E-31	postive
LINC00472	AL136320.1	0.552959135	1.73E-44	postive
ATM	AL136320.1	0.697964773	6.66E-80	postive
FBXW7	AL136320.1	0.424110084	6.07E-25	postive
TAZ	AC019257.1	0.435014414	2.71E-26	postive
TFAP2C	AC044840.1	0.523151526	3.37E-39	postive
HBA1	AC044840.1	0.473500898	1.80E-31	postive
DUOX1	AC044840.1	0.433801519	3.85E-26	postive
MUC1	AL139280.1	0.449437722	3.71E-28	postive
SOCS1	AL139280.1	0.473888862	1.59E-31	postive
DDIT3	LINC01055	0.439674269	6.93E-27	postive
GABARAPL1	LINC01055	0.449340985	3.82E-28	postive
ATM	MGAT3-AS1	0.552424154	2.17E-44	postive
VEGFA	AP001626.1	0.404369449	1.28E-22	postive
HELLS	AC117383.1	0.407429351	5.70E-23	postive
ATM	AC117383.1	0.433584039	4.10E-26	postive
HELLS	AC131159.1	0.517776102	2.67E-38	postive
TUBE1	AC131159.1	0.555168863	6.66E-45	postive
ALOX12	AC131159.1	0.536052082	2.01E-41	postive
GABPB1	AC131159.1	0.487237856	1.75E-33	postive
LINC00472	AC131159.1	0.477986675	4.06E-32	postive
ATM	AC131159.1	0.670403425	1.30E-71	postive
FBXW7	AC131159.1	0.44069875	5.12E-27	postive
FANCD2	AL035587.1	0.503724217	5.05E-36	postive
HELLS	AL035587.1	0.481212508	1.37E-32	postive
ZNF419	AL035587.1	0.452176836	1.61E-28	postive
ALOX12	AL035587.1	0.516681096	4.06E-38	postive
ATM	AL035587.1	0.422682649	9.05E-25	postive
FBXW7	AL035587.1	0.409863198	2.99E-23	postive
TFAP2C	AC037198.2	0.428273584	1.88E-25	postive
HBA1	AC037198.2	0.422087915	1.07E-24	postive
HELLS	AL589935.1	0.502793031	7.09E-36	postive
KLHL24	AL589935.1	0.401731462	2.54E-22	postive
TUBE1	AL589935.1	0.502507774	7.86E-36	postive
ALOX12	AL589935.1	0.438095637	1.10E-26	postive
GABPB1	AL589935.1	0.560385717	6.82E-46	postive
MAPK8	AL589935.1	0.412577321	1.44E-23	postive
LINC00472	AL589935.1	0.807343362	4.07E-125	postive
ATM	AL589935.1	0.699097388	2.90E-80	postive
FBXW7	AL589935.1	0.485637823	3.04E-33	postive
HELLS	AC139887.4	0.430643023	9.55E-26	postive
ZNF419	AC139887.4	0.418756977	2.68E-24	postive
KLHL24	AC139887.4	0.44430483	1.75E-27	postive
TUBE1	AC139887.4	0.452477465	1.46E-28	postive
ALOX12	AC139887.4	0.441957053	3.52E-27	postive
GABPB1	AC139887.4	0.545432229	4.20E-43	postive
LINC00472	AC139887.4	0.858748608	4.59E-158	postive

ATM	AC139887.4	0.613602366	4.57E-57	postive
FBXW7	AC139887.4	0.4412329	4.37E-27	postive
ALOX12	LRRC8C-DT	0.44205327	3.42E-27	postive
GABPB1	LRRC8C-DT	0.566886809	3.77E-47	postive
ZEB1	LRRC8C-DT	0.507721223	1.17E-36	postive
MAPK8	LRRC8C-DT	0.40535592	9.85E-23	postive
ATM	LRRC8C-DT	0.591723926	3.20E-52	postive
FBXW7	LRRC8C-DT	0.485849051	2.82E-33	postive
MTOR	SUCLG2-AS1	0.554296618	9.70E-45	postive
ACSL3	SUCLG2-AS1	0.46226665	6.85E-30	postive
HIF1A	SUCLG2-AS1	0.424700553	5.15E-25	postive
OXSR1	SUCLG2-AS1	0.408206401	4.64E-23	postive
KLHL24	SUCLG2-AS1	0.616388763	1.04E-57	postive
MAP3K5	SUCLG2-AS1	0.420179862	1.81E-24	postive
EIF2AK4	SUCLG2-AS1	0.405141172	1.04E-22	postive
IREB2	SUCLG2-AS1	0.540112539	3.82E-42	postive
PIK3CA	SUCLG2-AS1	0.563061427	2.09E-46	postive
ATG5	SUCLG2-AS1	0.408242628	4.60E-23	postive
ATG7	SUCLG2-AS1	0.487695055	1.49E-33	postive
LINC00472	SUCLG2-AS1	0.520119026	1.09E-38	postive
PRKAA2	SUCLG2-AS1	0.445243285	1.32E-27	postive
PRKAA1	SUCLG2-AS1	0.424462574	5.50E-25	postive
TGFBR1	SUCLG2-AS1	0.451291342	2.11E-28	postive
LPIN1	SUCLG2-AS1	0.507959971	1.07E-36	postive
ATM	SUCLG2-AS1	0.472840855	2.24E-31	postive
TRIB3	AC107021.2	0.429987092	1.15E-25	postive
SLC2A1	AC107021.2	0.402681831	1.98E-22	postive
HELLS	KLF7-IT1	0.528629838	3.93E-40	postive
TUBE1	KLF7-IT1	0.416800485	4.59E-24	postive
IREB2	KLF7-IT1	0.404950851	1.10E-22	postive
GABPB1	KLF7-IT1	0.552415822	2.18E-44	postive
ATG7	KLF7-IT1	0.469001906	7.87E-31	postive
MAPK8	KLF7-IT1	0.413459984	1.14E-23	postive
LINC00472	KLF7-IT1	0.700516194	1.02E-80	postive
ATM	KLF7-IT1	0.742050661	2.44E-95	postive
FBXW7	KLF7-IT1	0.469885495	5.90E-31	postive
TFAP2C	AC002310.1	0.45696402	3.64E-29	postive
HBA1	AC002310.1	0.499050714	2.74E-35	postive
HELLS	AC084781.1	0.460034862	1.39E-29	postive
ALOX12	AC084781.1	0.450612399	2.59E-28	postive
ATM	AC084781.1	0.438851794	8.83E-27	postive
HELLS	AC112503.2	0.444268287	1.77E-27	postive
TUBE1	AC112503.2	0.481351075	1.31E-32	postive
ALOX12	AC112503.2	0.47369615	1.69E-31	postive
GABPB1	AC112503.2	0.478966581	2.92E-32	postive
LINC00472	AC112503.2	0.465875263	2.16E-30	postive
ATM	AC112503.2	0.659733589	1.23E-68	postive
FBXW7	AC112503.2	0.402171003	2.27E-22	postive
RB1	AC003984.1	0.420218907	1.79E-24	postive
TUBE1	AC003984.1	0.429293491	1.40E-25	postive
MAPK8	AC003984.1	0.447720881	6.25E-28	postive
PRKAA2	AC003984.1	0.51290549	1.69E-37	postive
ATM	AC003984.1	0.402475098	2.09E-22	postive
SIRT1	AC003984.1	0.473235688	1.97E-31	postive
HELLS	AC019080.1	0.490657465	5.34E-34	postive
TUBE1	AC019080.1	0.503249022	6.01E-36	postive
ALOX12	AC019080.1	0.454830431	7.07E-29	postive
GABPB1	AC019080.1	0.43812575	1.09E-26	postive

MAPK8	AC019080.1	0.42384434	6.54E-25	postive
LINC00472	AC019080.1	0.456840917	3.78E-29	postive
ATM	AC019080.1	0.644988017	1.01E-64	postive
FBXW7	AC019080.1	0.411420353	1.97E-23	postive
HELLS	AC007497.1	0.422459164	9.63E-25	postive
PHKG2	AC007497.1	0.416635235	4.80E-24	postive
PML	AL022328.3	0.413289015	1.19E-23	postive
BRD4	AL022328.3	0.479581695	2.38E-32	postive
ZNF419	AL022328.3	0.538112848	8.67E-42	postive
TUBE1	AL022328.3	0.455723206	5.36E-29	postive
SETD1B	AL022328.3	0.474246625	1.41E-31	postive
ALOX12	AL022328.3	0.559765177	8.97E-46	postive
PHKG2	AL022328.3	0.535779575	2.24E-41	postive
EGLN2	AL022328.3	0.441536903	3.99E-27	postive
TAZ	AL022328.3	0.629476365	7.92E-61	postive
FBXW7	AL022328.3	0.418729285	2.70E-24	postive
TFAP2C	LINC02848	0.545612751	3.89E-43	postive
HBA1	LINC02848	0.524734041	1.82E-39	postive
HELLS	AC103591.3	0.507818099	1.13E-36	postive
TUBE1	AC103591.3	0.506958588	1.54E-36	postive
ALOX12	AC103591.3	0.422855752	8.62E-25	postive
GABPB1	AC103591.3	0.483209463	6.96E-33	postive
ATM	AC103591.3	0.57973743	1.02E-49	postive
FBXW7	AC103591.3	0.460765012	1.10E-29	postive
STEAP3	DAAM2-AS1	0.413046243	1.27E-23	postive
ZNF419	AL354836.1	0.46291301	5.58E-30	postive
DRD4	AL354836.1	0.463776144	4.24E-30	postive
ALOX12	AL354836.1	0.419058051	2.47E-24	postive
NCOA4	AL354836.1	-0.438552945	9.64E-27	negative
PHKG2	AL354836.1	0.643562186	2.36E-64	postive
EGLN2	AL354836.1	0.401967777	2.39E-22	postive
TAZ	AL354836.1	0.719084748	6.30E-87	postive
GPX4	AC242842.1	0.42199667	1.09E-24	postive
PHKG2	AC242842.1	0.499892967	2.02E-35	postive
HELLS	AP000919.1	0.465676275	2.30E-30	postive
GABPB1	AP000919.1	0.423121113	8.00E-25	postive
ATG7	AP000919.1	0.424632683	5.24E-25	postive
LINC00472	AP000919.1	0.476360839	6.99E-32	postive
ATM	AP000919.1	0.657491053	5.01E-68	postive
FBXW7	AP000919.1	0.411442388	1.96E-23	postive
HELLS	AL133406.2	0.40492093	1.10E-22	postive
ZNF419	AL133406.2	0.470222212	5.29E-31	postive
VEGFA	AL133406.2	0.440039589	6.22E-27	postive
TUBE1	AL133406.2	0.558172729	1.80E-45	postive
SETD1B	AL133406.2	0.468537164	9.15E-31	postive
ALOX12	AL133406.2	0.564089013	1.32E-46	postive
GABPB1	AL133406.2	0.425438494	4.18E-25	postive
ATM	AL133406.2	0.496561018	6.67E-35	postive
FBXW7	AL133406.2	0.507095948	1.47E-36	postive
GABPB1	AC011389.1	0.491232876	4.37E-34	postive
LINC00472	AC011389.1	0.775835189	1.73E-109	postive
ATM	AC011389.1	0.439318164	7.69E-27	postive
TFAP2C	AC007993.2	0.603135815	1.06E-54	postive
HBA1	AC007993.2	0.497378071	4.98E-35	postive
AKR1C2	ZMIZ1-AS1	0.452392476	1.50E-28	postive
NQO1	ZMIZ1-AS1	0.493500733	1.97E-34	postive
STEAP3	ZMIZ1-AS1	0.410181715	2.74E-23	postive
G6PD	ZMIZ1-AS1	0.4433327	2.34E-27	postive

FANCD2	AC016831.1	0.440012257	6.27E-27 postive
HELLS	AC016831.1	0.464076097	3.85E-30 postive
STMN1	AC016831.1	0.517758393	2.69E-38 postive
RRM2	AC016831.1	0.420988305	1.45E-24 postive
AURKA	AC016831.1	0.444494458	1.65E-27 postive
CDKN2A	AC016831.1	0.585931745	5.34E-51 postive
LINC00472	AC016831.1	0.446412284	9.29E-28 postive
HELLS	AC005021.1	0.542438942	1.46E-42 postive
TUBE1	AC005021.1	0.438140529	1.09E-26 postive
ALOX12	AC005021.1	0.480668885	1.65E-32 postive
GABPB1	AC005021.1	0.483505759	6.29E-33 postive
LINC00472	AC005021.1	0.447863291	5.99E-28 postive
ATM	AC005021.1	0.664595642	5.62E-70 postive
FBXW7	AC005021.1	0.480429811	1.79E-32 postive
ZNF419	AC009093.6	0.41263344	1.42E-23 postive
LPIN1	AC009093.6	0.434451034	3.19E-26 postive
FANCD2	AC145285.3	0.410921579	2.25E-23 postive
TFAP2C	AC145285.3	0.548184453	1.32E-43 postive
HBA1	AC145285.3	0.494062825	1.62E-34 postive
DUOX1	AC145285.3	0.424051558	6.17E-25 postive
ZNF419	AC124319.1	0.40585354	8.64E-23 postive
TUBE1	AC124319.1	0.446025707	1.04E-27 postive
ALOX12	AC124319.1	0.452138292	1.62E-28 postive
PHKG2	AC124319.1	0.407795415	5.17E-23 postive
LINC00472	AC124319.1	0.475318055	9.88E-32 postive
TAZ	AC124319.1	0.44165773	3.85E-27 postive
FANCD2	AC005911.1	0.423867022	6.50E-25 postive
TFAP2C	AC005911.1	0.54053672	3.21E-42 postive
HBA1	AC005911.1	0.506719488	1.69E-36 postive
JDP2	RASAL2-AS1	0.496567985	6.65E-35 postive
GABPB1	RASAL2-AS1	0.505920255	2.26E-36 postive
ZEB1	RASAL2-AS1	0.610843201	1.96E-56 postive
MAPK8	RASAL2-AS1	0.503910903	4.72E-36 postive
EPAS1	RASAL2-AS1	0.518093089	2.37E-38 postive
HELLS	AC006566.1	0.512600048	1.90E-37 postive
GABPB1	AC006566.1	0.502603751	7.60E-36 postive
ATG7	AC006566.1	0.425628844	3.96E-25 postive
LINC00472	AC006566.1	0.564937164	9.05E-47 postive
ATM	AC006566.1	0.68070517	1.33E-74 postive
FBXW7	AC006566.1	0.43671999	1.65E-26 postive
ATF4	SNHG1	0.469555327	6.57E-31 postive
TAZ	SNHG1	0.409939088	2.93E-23 postive
KLHL24	SREBF2-AS1	0.414070884	9.63E-24 postive
GABARAPL1	SREBF2-AS1	0.404721439	1.16E-22 postive
LINC00472	SREBF2-AS1	0.563560765	1.67E-46 postive
LPIN1	SREBF2-AS1	0.495100907	1.12E-34 postive
HELLS	AC004253.1	0.430291632	1.06E-25 postive
ZNF419	AC004253.1	0.559565016	9.79E-46 postive
VEGFA	AC004253.1	0.477553558	4.69E-32 postive
TUBE1	AC004253.1	0.670612216	1.14E-71 postive
DRD4	AC004253.1	0.422843367	8.65E-25 postive
ALOX12	AC004253.1	0.678162248	7.46E-74 postive
GABPB1	AC004253.1	0.405694372	9.01E-23 postive
PHKG2	AC004253.1	0.485801802	2.87E-33 postive
ATM	AC004253.1	0.441841026	3.65E-27 postive
TAZ	AC004253.1	0.656884371	7.31E-68 postive
FBXW7	AC004253.1	0.474717122	1.21E-31 postive
FANCD2	GCC2-AS1	0.428935847	1.55E-25 postive

TFAP2C	GCC2-AS1	0.525749001	1.22E-39	postive
LPIN1	GCC2-AS1	0.408253332	4.58E-23	postive
HELLS	AC010542.5	0.460778416	1.10E-29	postive
ZNF419	AC010542.5	0.541421529	2.23E-42	postive
TUBE1	AC010542.5	0.532311314	9.07E-41	postive
ALOX12	AC010542.5	0.510177815	4.69E-37	postive
YWHAE	AC010542.5	-0.409400437	3.38E-23	negative
GABPB1	AC010542.5	0.434784262	2.90E-26	postive
PHKG2	AC010542.5	0.547235005	1.97E-43	postive
ATM	AC010542.5	0.45602991	4.87E-29	postive
TAZ	AC010542.5	0.611194046	1.63E-56	postive
FBXW7	AC010542.5	0.476471564	6.74E-32	postive
GABPB1	LINC01465	0.40249883	2.08E-22	postive
LINC00472	LINC01465	0.457073699	3.52E-29	postive
ATM	LINC01465	0.431713551	7.03E-26	postive
HELLS	AC073046.1	0.443087882	2.52E-27	postive
MTOR	AC073046.1	0.506299632	1.97E-36	postive
ZFP69B	AC073046.1	0.514919355	7.91E-38	postive
ALOX12	AC073046.1	0.480919667	1.51E-32	postive
IREB2	AC073046.1	0.469463865	6.77E-31	postive
SP1	AC073046.1	0.446211435	9.87E-28	postive
GABPB1	AC073046.1	0.446110282	1.02E-27	postive
CYBB	AC073046.1	0.424593081	5.30E-25	postive
ATG7	AC073046.1	0.496883363	5.94E-35	postive
ULK2	AC073046.1	0.423345081	7.52E-25	postive
MAPK8	AC073046.1	0.449133377	4.07E-28	postive
TLR4	AC073046.1	0.491709205	3.70E-34	postive
ATM	AC073046.1	0.794828754	1.39E-118	postive
FBXW7	AC073046.1	0.517612578	2.85E-38	postive
BACH1	AC073046.1	0.437786163	1.21E-26	postive
LINC00472	AC107952.2	0.414663612	8.20E-24	postive
HELLS	AC093484.4	0.574896379	9.74E-49	postive
ZNF419	AC093484.4	0.522357837	4.59E-39	postive
TUBE1	AC093484.4	0.516541097	4.28E-38	postive
ALOX12	AC093484.4	0.585376717	6.97E-51	postive
GABPB1	AC093484.4	0.508826522	7.75E-37	postive
LINC00472	AC093484.4	0.508746695	7.98E-37	postive
ATM	AC093484.4	0.540573243	3.16E-42	postive
FBXW7	AC093484.4	0.463353957	4.85E-30	postive
HSPB1	AL161729.3	0.502387517	8.22E-36	postive
HBA1	AL161729.3	0.632896317	1.15E-61	postive
HRAS	AL161729.3	0.654186785	3.88E-67	postive
MAP1LC3A	AL161729.3	0.470467454	4.88E-31	postive
EGLN2	AL161729.3	0.822004407	2.06E-133	postive
FANCD2	AC006064.1	0.440013082	6.27E-27	postive
HELLS	AC006064.1	0.492317234	2.99E-34	postive
ZNF419	AC006064.1	0.507784105	1.14E-36	postive
ZFP69B	AC006064.1	0.402553964	2.05E-22	postive
TUBE1	AC006064.1	0.516003767	5.25E-38	postive
SETD1B	AC006064.1	0.482131091	1.00E-32	postive
ALOX12	AC006064.1	0.691242252	8.55E-78	postive
GABPB1	AC006064.1	0.451242969	2.14E-28	postive
ATM	AC006064.1	0.603019306	1.12E-54	postive
FBXW7	AC006064.1	0.464865448	2.99E-30	postive
HELLS	AC012442.1	0.553962624	1.12E-44	postive
TUBE1	AC012442.1	0.415606789	6.35E-24	postive
ALOX12	AC012442.1	0.484252451	4.88E-33	postive
GABPB1	AC012442.1	0.412525535	1.46E-23	postive

LINC00472	AC012442.1	0.420032184	1.89E-24	postive
ATM	AC012442.1	0.473663168	1.71E-31	postive
TFAP2C	TFPI2-DT	0.52951069	2.77E-40	postive
HBA1	TFPI2-DT	0.506206319	2.04E-36	postive
ZNF419	TSC22D1-AS	0.50539483	2.74E-36	postive
KLHL24	TSC22D1-AS	0.491706346	3.70E-34	postive
ALOX12	TSC22D1-AS	0.412336748	1.54E-23	postive
GABPB1	TSC22D1-AS	0.530861573	1.62E-40	postive
LINC00472	TSC22D1-AS	0.78572743	4.15E-114	postive
LPIN1	TSC22D1-AS	0.47637284	6.96E-32	postive
ATM	TSC22D1-AS	0.418437419	2.93E-24	postive
HELLS	AC015727.1	0.570144301	8.63E-48	postive
ZNF419	AC015727.1	0.471020696	4.07E-31	postive
TUBE1	AC015727.1	0.549421501	7.81E-44	postive
ALOX12	AC015727.1	0.622595836	3.59E-59	postive
GABPB1	AC015727.1	0.562563642	2.61E-46	postive
MAPK8	AC015727.1	0.426914301	2.76E-25	postive
LINC00472	AC015727.1	0.661283687	4.63E-69	postive
ATM	AC015727.1	0.683549158	1.88E-75	postive
FBXW7	AC015727.1	0.511596702	2.76E-37	postive
GABPB1	AC002044.1	0.408092941	4.78E-23	postive
LINC00472	AC002044.1	0.630170835	5.36E-61	postive
TUBE1	PSMB8-AS1	0.428512495	1.75E-25	postive
PHKG2	PSMB8-AS1	0.419306941	2.31E-24	postive
IFNG	PSMB8-AS1	0.479251885	2.66E-32	postive
TAZ	PSMB8-AS1	0.46577399	2.23E-30	postive
RPL8	OSER1-DT	0.531712067	1.15E-40	postive
HRAS	OSER1-DT	0.413519625	1.12E-23	postive
HSF1	DNAJC9-AS1	0.414966422	7.56E-24	postive
HELLS	DNAJC9-AS1	0.404010866	1.40E-22	postive
ZNF419	DNAJC9-AS1	0.405876121	8.59E-23	postive
PHKG2	DNAJC9-AS1	0.563795276	1.51E-46	postive
TAZ	DNAJC9-AS1	0.616524597	9.62E-58	postive
ZNF419	AC016026.1	0.452206202	1.59E-28	postive
VEGFA	AC016026.1	0.493217083	2.18E-34	postive
TUBE1	AC016026.1	0.58572129	5.91E-51	postive
SETD1B	AC016026.1	0.470361157	5.05E-31	postive
ALOX12	AC016026.1	0.634029715	6.01E-62	postive
ATM	AC016026.1	0.565949103	5.75E-47	postive
FBXW7	AC016026.1	0.437519455	1.30E-26	postive
SLC3A2	AP000757.1	0.529366752	2.93E-40	postive
FH	AP000757.1	0.445775766	1.12E-27	postive
ISCU	AP000757.1	0.666146848	2.07E-70	postive
DDIT3	AP000757.1	0.613374352	5.15E-57	postive
HERPUD1	AP000757.1	0.40235932	2.16E-22	postive
SLC2A12	AP000757.1	0.410867128	2.28E-23	postive
CS	AP000757.1	0.412970088	1.30E-23	postive
GOT1	AP000757.1	0.457111171	3.48E-29	postive
ATG4D	AP000757.1	0.632656387	1.31E-61	postive
MAP1LC3A	AP000757.1	0.46629534	1.89E-30	postive
GABARAPL2	AP000757.1	0.430901192	8.87E-26	postive
GABARAPL1	AP000757.1	0.798425711	2.06E-120	postive
WIPI2	AP000757.1	0.457475542	3.10E-29	postive
BAP1	AP000757.1	0.449101904	4.11E-28	postive
LPIN1	AP000757.1	0.577908691	2.40E-49	postive
HELLS	AL121672.2	0.458141193	2.52E-29	postive
KLHL24	AL121672.2	0.50752263	1.25E-36	postive
TUBE1	AL121672.2	0.416054004	5.62E-24	postive

IREB2	AL121672.2	0.440266696	5.82E-27	postive
GABPB1	AL121672.2	0.564907421	9.17E-47	postive
PIK3CA	AL121672.2	0.456477463	4.24E-29	postive
ATG7	AL121672.2	0.431736544	6.98E-26	postive
MAPK8	AL121672.2	0.421966452	1.10E-24	postive
LINC00472	AL121672.2	0.873399499	6.32E-170	postive
ATM	AL121672.2	0.66929759	2.69E-71	postive
FBXW7	AL121672.2	0.408512515	4.28E-23	postive
HELLS	AL133297.1	0.516016236	5.22E-38	postive
ALOX12	AL133297.1	0.526247846	1.01E-39	postive
ATM	AL133297.1	0.506966642	1.54E-36	postive
FBXW7	AL133297.1	0.407994807	4.91E-23	postive
HELLS	AC087284.1	0.557579411	2.34E-45	postive
ALOX12	AC087284.1	0.427654844	2.24E-25	postive
GABPB1	AC087284.1	0.523128159	3.40E-39	postive
ATG7	AC087284.1	0.400081662	3.89E-22	postive
LINC00472	AC087284.1	0.663774871	9.50E-70	postive
ATM	AC087284.1	0.592477583	2.21E-52	postive
FBXW7	AC087284.1	0.458777355	2.06E-29	postive
RGS4	AC105118.1	0.517832376	2.62E-38	postive
KLHL24	TRHDE-AS1	0.478476437	3.45E-32	postive
TUBE1	TRHDE-AS1	0.439271293	7.80E-27	postive
MAPK8	TRHDE-AS1	0.420174459	1.81E-24	postive
LINC00472	TRHDE-AS1	0.597975831	1.44E-53	postive
OTUB1	AC002470.1	0.468975455	7.93E-31	postive
ATG4D	AC002470.1	0.411233188	2.07E-23	postive
RGS4	AP003390.1	0.546252438	2.98E-43	postive
VLDLR	VLDLR-AS1	0.47479214	1.18E-31	postive
TFAP2C	VLDLR-AS1	0.486339633	2.38E-33	postive
LPIN1	VLDLR-AS1	0.519674115	1.29E-38	postive
HELLS	AC010680.4	0.491555277	3.90E-34	postive
ZNF419	AC010680.4	0.42452087	5.41E-25	postive
TUBE1	AC010680.4	0.556098266	4.45E-45	postive
ALOX12	AC010680.4	0.551083775	3.85E-44	postive
GABPB1	AC010680.4	0.536712755	1.53E-41	postive
MAPK8	AC010680.4	0.400245932	3.73E-22	postive
LINC00472	AC010680.4	0.578433969	1.88E-49	postive
ATM	AC010680.4	0.670122296	1.57E-71	postive
FBXW7	AC010680.4	0.485967922	2.71E-33	postive
LPIN1	PGM5-AS1	0.410111029	2.79E-23	postive
HELLS	MIR762HG	0.406950006	6.47E-23	postive
ZNF419	MIR762HG	0.434386402	3.25E-26	postive
NCOA4	MIR762HG	-0.414449893	8.69E-24	negative
PHKG2	MIR762HG	0.663652881	1.03E-69	postive
MAPK1	MIR762HG	-0.449608737	3.52E-28	negative
ANO6	MIR762HG	-0.40273895	1.95E-22	negative
TAZ	MIR762HG	0.622341864	4.13E-59	postive
TAZ	AC136475.7	0.454571167	7.67E-29	postive
BRD4	AL356481.3	0.422733146	8.92E-25	postive
ZNF419	AL356481.3	0.545425043	4.21E-43	postive
VEGFA	AL356481.3	0.508910135	7.51E-37	postive
TUBE1	AL356481.3	0.553780173	1.21E-44	postive
SETD1B	AL356481.3	0.470820681	4.35E-31	postive
ALOX12	AL356481.3	0.570005761	9.19E-48	postive
PHKG2	AL356481.3	0.422036926	1.08E-24	postive
YY1AP1	AL356481.3	0.425837809	3.74E-25	postive
TAZ	AL356481.3	0.585317458	7.17E-51	postive
FANCD2	AC100778.2	0.416741101	4.66E-24	postive

HELLS	AC100778.2	0.45461423	7.57E-29	postive
ZNF419	AC100778.2	0.522648777	4.10E-39	postive
TUBE1	AC100778.2	0.523801628	2.62E-39	postive
ALOX12	AC100778.2	0.663899019	8.78E-70	postive
GABPB1	AC100778.2	0.420747318	1.55E-24	postive
ATM	AC100778.2	0.487966416	1.36E-33	postive
FBXW7	AC100778.2	0.409697612	3.12E-23	postive
FANCD2	AC010326.3	0.417131608	4.19E-24	postive
HELLS	AC010326.3	0.455050309	6.61E-29	postive
BRD4	AC010326.3	0.45383587	9.63E-29	postive
ZNF419	AC010326.3	0.65845223	2.75E-68	postive
TUBE1	AC010326.3	0.502422994	8.11E-36	postive
ALOX12	AC010326.3	0.526278477	9.93E-40	postive
PHKG2	AC010326.3	0.566779116	3.96E-47	postive
TAZ	AC010326.3	0.567470214	2.90E-47	postive
FBXW7	AC010326.3	0.410018283	2.86E-23	postive
HELLS	AC008972.1	0.441208471	4.40E-27	postive
GABPB1	AC008972.1	0.414214211	9.27E-24	postive
ATM	AC008972.1	0.504176339	4.28E-36	postive
HELLS	AC004263.1	0.54036834	3.44E-42	postive
ZNF419	AC004263.1	0.447212555	7.29E-28	postive
TUBE1	AC004263.1	0.594728198	7.28E-53	postive
ALOX12	AC004263.1	0.614079527	3.55E-57	postive
GABPB1	AC004263.1	0.493889771	1.72E-34	postive
LINC00472	AC004263.1	0.462340484	6.69E-30	postive
ATM	AC004263.1	0.662922108	1.64E-69	postive
FBXW7	AC004263.1	0.468991458	7.89E-31	postive
PHKG2	AL359881.1	0.47095201	4.17E-31	postive
GCH1	HCP5	0.436049084	2.00E-26	postive
IFNG	HCP5	0.419236085	2.35E-24	postive
TFAP2C	AP003392.4	0.491788656	3.60E-34	postive
HBA1	AP003392.4	0.410953985	2.23E-23	postive
NCF2	CCR5AS	0.449697842	3.43E-28	postive
HRAS	PPP1R21-DT	0.432809517	5.13E-26	postive
TFAP2C	AC093001.1	0.584977725	8.44E-51	postive
HBA1	AC093001.1	0.495784552	8.79E-35	postive
ISCU	B3GAT1-DT	0.472163446	2.80E-31	postive
ATG4D	B3GAT1-DT	0.467290288	1.37E-30	postive
GABARAPL1	B3GAT1-DT	0.539273283	5.39E-42	postive
LPIN1	B3GAT1-DT	0.447007926	7.76E-28	postive
ZNF419	AC012313.1	0.464722792	3.13E-30	postive
SETD1B	AC012313.1	0.424984624	4.75E-25	postive
ALOX12	AC012313.1	0.500370591	1.70E-35	postive
BAP1	AC012313.1	0.476228705	7.30E-32	postive
EIF2S1	AF111167.2	0.408818586	3.94E-23	postive
HRAS	AC125494.2	0.404735957	1.16E-22	postive
PHKG2	AC125494.2	0.43849484	9.80E-27	postive
TAZ	AC125494.2	0.562343094	2.87E-46	postive
ZNF419	AP006623.1	0.452737163	1.35E-28	postive
TUBE1	AP006623.1	0.416781481	4.61E-24	postive
ALOX12	AP006623.1	0.684784702	8.01E-76	postive
TAZ	AP006623.1	0.411743809	1.80E-23	postive
TFAP2C	AL121944.1	0.510883473	3.60E-37	postive
HBA1	AL121944.1	0.651884917	1.59E-66	postive
EGLN2	AL121944.1	0.417127408	4.19E-24	postive
ATF4	SNHG7	0.439861489	6.56E-27	postive
RPL8	SNHG7	0.512587706	1.91E-37	postive
JDP2	NFE4	0.612499233	8.19E-57	postive

RGS4	NFE4	0.473338248	1.90E-31	postive
EIF2S1	NFE4	0.484515636	4.46E-33	postive
KLHL24	AC104779.1	0.536064656	2.00E-41	postive
IREB2	AC104779.1	0.480061921	2.02E-32	postive
GABPB1	AC104779.1	0.45115155	2.20E-28	postive
PIK3CA	AC104779.1	0.510323153	4.44E-37	postive
KRAS	AC104779.1	0.490616446	5.42E-34	postive
MAPK8	AC104779.1	0.418020389	3.28E-24	postive
LINC00472	AC104779.1	0.901722014	1.02E-197	postive
PRKAA1	AC104779.1	0.422812324	8.72E-25	postive
ATM	AC104779.1	0.493926044	1.70E-34	postive
ISCU	AC019197.1	0.632884233	1.15E-61	postive
DDIT3	AC019197.1	0.44492882	1.45E-27	postive
KLHL24	AC019197.1	0.437620978	1.27E-26	postive
SLC2A12	AC019197.1	0.49455584	1.36E-34	postive
GOT1	AC019197.1	0.423239429	7.74E-25	postive
ATG4D	AC019197.1	0.446746386	8.40E-28	postive
MAP1LC3A	AC019197.1	0.449884792	3.24E-28	postive
GABARAPL2	AC019197.1	0.431573883	7.32E-26	postive
GABARAPL1	AC019197.1	0.648266516	1.43E-65	postive
WIPI2	AC019197.1	0.427531652	2.32E-25	postive
LINC00472	AC019197.1	0.403726537	1.51E-22	postive
LPIN1	AC019197.1	0.56651631	4.46E-47	postive
ISCU	AC079848.1	0.451685693	1.87E-28	postive
CHMP5	AC079848.1	0.516507448	4.33E-38	postive
GOT1	AC079848.1	0.427803013	2.14E-25	postive
GABARAPL2	AC079848.1	0.451185058	2.18E-28	postive
ISCU	LINC02343	0.475028934	1.09E-31	postive
ACSL3	LINC02343	0.459800753	1.50E-29	postive
KLHL24	LINC02343	0.622584811	3.61E-59	postive
GABARAPL1	LINC02343	0.593778477	1.17E-52	postive
WIPI2	LINC02343	0.407811799	5.15E-23	postive
LINC00472	LINC02343	0.597224318	2.10E-53	postive
LPIN1	LINC02343	0.641492919	7.99E-64	postive
HRAS	CAMTA1-DT	0.580238412	8.03E-50	postive
PHKG2	CAMTA1-DT	0.502247084	8.64E-36	postive
EGLN2	CAMTA1-DT	0.609178069	4.68E-56	postive
TAZ	CAMTA1-DT	0.449086401	4.13E-28	postive
ISCU	AC005281.1	0.492537263	2.77E-34	postive
KLHL24	AC005281.1	0.486636436	2.15E-33	postive
ATG4D	AC005281.1	0.410759375	2.35E-23	postive
GABARAPL1	AC005281.1	0.611664962	1.27E-56	postive
WIPI2	AC005281.1	0.422417779	9.74E-25	postive
BAP1	AC005281.1	0.476970786	5.70E-32	postive
LPIN1	AC005281.1	0.55933613	1.08E-45	postive
HELLS	AC007991.4	0.431151214	8.26E-26	postive
TUBE1	AC007991.4	0.43816642	1.08E-26	postive
ALOX12	AC007991.4	0.516107708	5.05E-38	postive
GABPB1	AC007991.4	0.420140525	1.83E-24	postive
ATM	AC007991.4	0.529876574	2.40E-40	postive
FBXW7	AC007991.4	0.469401361	6.91E-31	postive
TUBE1	AL121748.1	0.428791917	1.62E-25	postive
ALOX12	AL121748.1	0.461963607	7.54E-30	postive
GABPB1	AL121748.1	0.427803424	2.14E-25	postive
ATM	AL121748.1	0.519723288	1.27E-38	postive
FBXW7	AL121748.1	0.481511433	1.24E-32	postive
HBA1	AC006273.1	0.476446176	6.79E-32	postive
MAPK8	LHFPL3-AS2	0.403197877	1.73E-22	postive

HELLS	AC112496.1	0.474270504	1.40E-31	postive
TUBE1	AC112496.1	0.482626422	8.49E-33	postive
ALOX12	AC112496.1	0.565311531	7.65E-47	postive
GABPB1	AC112496.1	0.434253293	3.38E-26	postive
LINC00472	AC112496.1	0.597230364	2.09E-53	postive
ATM	AC112496.1	0.529888144	2.39E-40	postive
FBXW7	AC112496.1	0.436424618	1.80E-26	postive
HSF1	AC074212.1	0.442312658	3.17E-27	postive
SRC	AC074212.1	0.406500665	7.29E-23	postive
CHAC1	AC074212.1	0.410195657	2.73E-23	postive
PHKG2	AC074212.1	0.650136364	4.61E-66	postive
ULK1	AC074212.1	0.415954321	5.78E-24	postive
TAZ	AC074212.1	0.582789337	2.40E-50	postive
SOCS1	AC080013.2	0.406797371	6.74E-23	postive
LINC00472	AC080013.2	0.606926026	1.51E-55	postive
HBA1	GATA3-AS1	0.593168768	1.57E-52	postive
HRAS	GATA3-AS1	0.550063034	5.95E-44	postive
EGLN2	GATA3-AS1	0.809711403	2.08E-126	postive
HELLS	AL162724.2	0.42500324	4.73E-25	postive
ZNF419	AL162724.2	0.411123182	2.13E-23	postive
TUBE1	AL162724.2	0.565252764	7.86E-47	postive
ALOX12	AL162724.2	0.579528968	1.12E-49	postive
GABPB1	AL162724.2	0.490320964	6.01E-34	postive
ATM	AL162724.2	0.626491747	4.19E-60	postive
FBXW7	AL162724.2	0.417552737	3.73E-24	postive
HELLS	AC090181.2	0.52436668	2.10E-39	postive
KLHL24	AC090181.2	0.426911877	2.76E-25	postive
TUBE1	AC090181.2	0.428369017	1.83E-25	postive
IREB2	AC090181.2	0.430753603	9.25E-26	postive
GABPB1	AC090181.2	0.580298468	7.81E-50	postive
PIK3CA	AC090181.2	0.417180745	4.13E-24	postive
MAPK8	AC090181.2	0.440435144	5.53E-27	postive
LINC00472	AC090181.2	0.823305973	3.47E-134	postive
ATM	AC090181.2	0.695675376	3.53E-79	postive
FBXW7	AC090181.2	0.470664307	4.58E-31	postive
HELLS	AC010761.3	0.579279209	1.26E-49	postive
TUBE1	AC010761.3	0.517773269	2.68E-38	postive
ALOX12	AC010761.3	0.56792184	2.37E-47	postive
GABPB1	AC010761.3	0.558132685	1.83E-45	postive
ATG7	AC010761.3	0.405272348	1.01E-22	postive
MAPK8	AC010761.3	0.459625233	1.58E-29	postive
LINC00472	AC010761.3	0.564652074	1.03E-46	postive
ATM	AC010761.3	0.778097137	1.60E-110	postive
FBXW7	AC010761.3	0.512884236	1.70E-37	postive
HELLS	AL391001.1	0.417951748	3.35E-24	postive
KLHL24	AL391001.1	0.479029461	2.86E-32	postive
TUBE1	AL391001.1	0.41293348	1.31E-23	postive
IREB2	AL391001.1	0.455584673	5.60E-29	postive
GABPB1	AL391001.1	0.526966879	7.58E-40	postive
PIK3CA	AL391001.1	0.464072676	3.85E-30	postive
KRAS	AL391001.1	0.43669596	1.66E-26	postive
MAPK8	AL391001.1	0.442165873	3.31E-27	postive
LINC00472	AL391001.1	0.754895242	1.91E-100	postive
PRKAA2	AL391001.1	0.429399437	1.36E-25	postive
PRKAA1	AL391001.1	0.403544636	1.58E-22	postive
ATM	AL391001.1	0.58574394	5.84E-51	postive
HSPB1	IDH1-AS1	0.409587625	3.21E-23	postive
HRAS	IDH1-AS1	0.425811115	3.77E-25	postive

SLC3A2	AC120498.4	0.406300246	7.68E-23	postive
FH	AC120498.4	0.413163656	1.23E-23	postive
ISCU	AC120498.4	0.488886853	9.89E-34	postive
CHAC1	AC120498.4	0.420867462	1.50E-24	postive
DDIT3	AC120498.4	0.440185689	5.96E-27	postive
SLC2A8	AC120498.4	0.556253534	4.16E-45	postive
ATG4D	AC120498.4	0.557261384	2.68E-45	postive
MAP1LC3A	AC120498.4	0.459807375	1.49E-29	postive
GABARAPL1	AC120498.4	0.493534395	1.95E-34	postive
WIPI2	AC120498.4	0.465340432	2.57E-30	postive
LPIN1	AC120498.4	0.492631653	2.68E-34	postive
TUBE1	AC008554.1	0.432336596	5.87E-26	postive
ALOX12	AC008554.1	0.422052481	1.08E-24	postive
ATM	AC008554.1	0.460164071	1.33E-29	postive
PHKG2	AC092757.2	0.422595084	9.27E-25	postive
SLC2A12	AC104237.2	0.452035479	1.68E-28	postive
ZNF419	AL390066.1	0.414042207	9.71E-24	postive
TUBE1	AL390066.1	0.437001347	1.52E-26	postive
ALOX12	AL390066.1	0.577067686	3.55E-49	postive
PHKG2	AL390066.1	0.422063949	1.07E-24	postive
TAZ	AL390066.1	0.486440167	2.30E-33	postive
GABPB1	AC137932.2	0.430631894	9.58E-26	postive
ATM	AC137932.2	0.402972038	1.84E-22	postive
FBXW7	AC137932.2	0.40525683	1.01E-22	postive
HELLS	AL049869.2	0.51339903	1.40E-37	postive
ZNF419	AL049869.2	0.4162607	5.31E-24	postive
KLHL24	AL049869.2	0.423857632	6.52E-25	postive
TUBE1	AL049869.2	0.51425162	1.02E-37	postive
ALOX12	AL049869.2	0.520830409	8.28E-39	postive
IREB2	AL049869.2	0.462461036	6.44E-30	postive
GABPB1	AL049869.2	0.577795481	2.53E-49	postive
PIK3CA	AL049869.2	0.415007432	7.47E-24	postive
MAPK8	AL049869.2	0.486333287	2.39E-33	postive
LINC00472	AL049869.2	0.74769221	1.52E-97	postive
ATM	AL049869.2	0.753773038	5.49E-100	postive
FBXW7	AL049869.2	0.4949917	1.16E-34	postive
FANCD2	AP003774.2	0.446267154	9.70E-28	postive
TFAP2C	AP003774.2	0.484527945	4.44E-33	postive
HBA1	AP003774.2	0.45194293	1.72E-28	postive
ARNTL	AL139011.1	0.434413998	3.22E-26	postive
ZNF419	AL139011.1	0.493756782	1.80E-34	postive
VEGFA	AL139011.1	0.410634957	2.43E-23	postive
TUBE1	AL139011.1	0.568110516	2.17E-47	postive
SETD1B	AL139011.1	0.528556612	4.05E-40	postive
ALOX12	AL139011.1	0.662843498	1.72E-69	postive
GABPB1	AL139011.1	0.48371477	5.86E-33	postive
MAPK8	AL139011.1	0.459147226	1.84E-29	postive
LINC00472	AL139011.1	0.487398739	1.66E-33	postive
ATM	AL139011.1	0.675978533	3.24E-73	postive
YY1AP1	AL139011.1	0.431738607	6.98E-26	postive
FBXW7	AL139011.1	0.458790191	2.06E-29	postive
NCF2	AC002091.1	0.648548432	1.20E-65	postive
CYBB	AC002091.1	0.553853138	1.17E-44	postive
ALOX15B	AC002091.1	0.413218948	1.21E-23	postive
TNFAIP3	AC002091.1	0.411553534	1.90E-23	postive
IL6	AC004264.1	0.505577804	2.57E-36	postive
SLC2A3	AC004264.1	0.423657888	6.89E-25	postive
ZNF419	AC005674.1	0.545585082	3.94E-43	postive

VEGFA	AC005674.1	0.440652358	5.19E-27	postive
TUBE1	AC005674.1	0.610480561	2.37E-56	postive
SETD1B	AC005674.1	0.452965347	1.26E-28	postive
ALOX12	AC005674.1	0.529630624	2.64E-40	postive
GABPB1	AC005674.1	0.503804954	4.91E-36	postive
ATM	AC005674.1	0.484861937	3.96E-33	postive
TAZ	AC005674.1	0.514962466	7.79E-38	postive
FBXW7	AC005674.1	0.480224055	1.92E-32	postive
PHKG2	AL162741.1	0.444642619	1.58E-27	postive
TAZ	AL162741.1	0.495666535	9.16E-35	postive
ASNS	AC034213.1	0.46732157	1.36E-30	postive
TRIB3	AC034213.1	0.495391348	1.01E-34	postive
CARS1	AC034213.1	0.430512141	9.92E-26	postive
GPX4	CH17-340M2	0.502541369	7.77E-36	postive
HSPB1	CH17-340M2	0.555499576	5.77E-45	postive
RPL8	CH17-340M2	0.413593765	1.10E-23	postive
HBA1	CH17-340M2	0.691168536	9.01E-78	postive
HRAS	CH17-340M2	0.700324058	1.17E-80	postive
MAP1LC3A	CH17-340M2	0.507691488	1.18E-36	postive
EGLN2	CH17-340M2	0.788410471	2.10E-115	postive
KLHL24	AC090579.1	0.557837468	2.09E-45	postive
IREB2	AC090579.1	0.427035299	2.67E-25	postive
GABPB1	AC090579.1	0.50545651	2.68E-36	postive
PIK3CA	AC090579.1	0.487193194	1.78E-33	postive
LINC00472	AC090579.1	0.892009227	2.54E-187	postive
TGFBR1	AC090579.1	0.423916883	6.41E-25	postive
ATM	AC090579.1	0.553371023	1.45E-44	postive
HELLS	AC009054.2	0.412702964	1.39E-23	postive
VEGFA	AC010655.2	0.417858933	3.43E-24	postive
HILPDA	AC010655.2	0.527054958	7.32E-40	postive
TAZ	AC010655.2	0.44521343	1.33E-27	postive
HELLS	AP002340.1	0.481364016	1.30E-32	postive
ZNF419	AP002340.1	0.426955611	2.73E-25	postive
TUBE1	AP002340.1	0.531352642	1.33E-40	postive
ALOX12	AP002340.1	0.534090427	4.44E-41	postive
GABPB1	AP002340.1	0.547736108	1.59E-43	postive
MAPK8	AP002340.1	0.433954385	3.68E-26	postive
LINC00472	AP002340.1	0.572233377	3.32E-48	postive
ATM	AP002340.1	0.721774919	7.21E-88	postive
FBXW7	AP002340.1	0.510278261	4.52E-37	postive
FANCD2	AC011481.1	0.421738865	1.18E-24	postive
ZNF419	AC011481.1	0.452330107	1.53E-28	postive
ALOX12	AC011481.1	0.562894987	2.25E-46	postive
TFAP2C	AC011481.1	0.439214544	7.93E-27	postive
TAZ	AC011481.1	0.410901037	2.26E-23	postive
TFAP2C	AC010531.6	0.608602271	6.32E-56	postive
HBA1	AC010531.6	0.577588547	2.79E-49	postive
ISCU	AC008736.1	0.49795693	4.05E-35	postive
DDIT3	AC008736.1	0.403225689	1.72E-22	postive
SLC2A8	AC008736.1	0.527578653	5.95E-40	postive
TFAP2C	AC008736.1	0.401555443	2.66E-22	postive
ATG4D	AC008736.1	0.486040937	2.64E-33	postive
MAP1LC3A	AC008736.1	0.497797929	4.29E-35	postive
WIPI2	AC008736.1	0.455518217	5.71E-29	postive
MAPK1	AC008736.1	-0.418771301	2.67E-24	negative
LPIN1	AC008736.1	0.448629847	4.75E-28	postive
SIRT1	AC008736.1	-0.450123775	3.01E-28	negative
HELLS	AC005261.1	0.442383394	3.10E-27	postive

ZNF419	AC005261.1	0.576898176	3.84E-49	postive
TUBE1	AC005261.1	0.465165316	2.72E-30	postive
GABPB1	AC005261.1	0.567606852	2.73E-47	postive
LINC00472	AC005261.1	0.555319103	6.24E-45	postive
TAZ	AC005261.1	0.416356209	5.18E-24	postive
FBXW7	AC005261.1	0.4720635	2.89E-31	postive
ZNF419	PPP1R26-AS	0.407648825	5.38E-23	postive
TUBE1	PPP1R26-AS	0.421634205	1.21E-24	postive
ALOX12	PPP1R26-AS	0.561244159	4.67E-46	postive
ISCU	GSEC	0.407022191	6.35E-23	postive
KLHL24	GSEC	0.44678257	8.30E-28	postive
LINC00472	GSEC	0.603579452	8.44E-55	postive
LPIN1	GSEC	0.53061083	1.79E-40	postive
ZNF419	AC253536.6	0.533351346	5.98E-41	postive
TUBE1	AC253536.6	0.46451389	3.35E-30	postive
ALOX12	AC253536.6	0.476136562	7.53E-32	postive
GABPB1	AC253536.6	0.446769296	8.34E-28	postive
LINC00472	AC253536.6	0.690580153	1.37E-77	postive
LPIN1	AC253536.6	0.462758258	5.86E-30	postive
ATM	AC253536.6	0.426685052	2.94E-25	postive
TAZ	AC253536.6	0.41021603	2.72E-23	postive
TUBE1	AC079906.1	0.426393737	3.20E-25	postive
PHKG2	AC079906.1	0.451135832	2.21E-28	postive
TAZ	AC079906.1	0.579355125	1.22E-49	postive
BRD4	AC004771.4	0.425719543	3.86E-25	postive
ZNF419	AC004771.4	0.448218719	5.38E-28	postive
VEGFA	AC004771.4	0.441242118	4.36E-27	postive
TUBE1	AC004771.4	0.506920434	1.57E-36	postive
SETD1B	AC004771.4	0.541753989	1.94E-42	postive
ALOX12	AC004771.4	0.670823532	9.90E-72	postive
GABPB1	AC004771.4	0.405069638	1.06E-22	postive
LINC00472	AC004771.4	0.432099975	6.29E-26	postive
ATM	AC004771.4	0.566930405	3.70E-47	postive
FBXW7	AC004771.4	0.402935562	1.86E-22	postive
HSF1	MHENCR	0.418242159	3.09E-24	postive
DRD4	MHENCR	0.410846902	2.30E-23	postive
NRAS	MHENCR	-0.411965791	1.70E-23	negative
HRAS	MHENCR	0.477476315	4.82E-32	postive
NCOA4	MHENCR	-0.504177456	4.28E-36	negative
PHKG2	MHENCR	0.756810218	3.11E-101	postive
BECN1	MHENCR	-0.431631904	7.19E-26	negative
MAPK1	MHENCR	-0.430687618	9.43E-26	negative
ANO6	MHENCR	-0.424137475	6.02E-25	negative
TAZ	MHENCR	0.834950694	2.12E-141	postive
TUBE1	AC092903.2	0.42761047	2.27E-25	postive
ALOX12	AC092903.2	0.421183325	1.37E-24	postive
LINC00472	AC092903.2	0.51449053	9.31E-38	postive
ATM	AC092903.2	0.470740416	4.46E-31	postive
LINC00472	MAILR	0.589808871	8.17E-52	postive
FANCD2	HMGA1P4	0.44876975	4.55E-28	postive
TFAP2C	HMGA1P4	0.570317311	7.98E-48	postive
HBA1	HMGA1P4	0.512665099	1.85E-37	postive
DUOX1	HMGA1P4	0.411855129	1.75E-23	postive
GPX4	TYMSOS	0.410682789	2.40E-23	postive
PEBP1	TYMSOS	0.408203637	4.64E-23	postive
MUC1	AC130371.2	0.488414633	1.16E-33	postive
WIPI2	AC130371.2	0.434081732	3.55E-26	postive
EGLN2	AC130371.2	0.546147061	3.11E-43	postive

ZNF419	AC005013.1	0.454377869	8.14E-29	postive
TUBE1	AC005013.1	0.532505819	8.39E-41	postive
ALOX12	AC005013.1	0.531554174	1.23E-40	postive
GABPB1	AC005013.1	0.471519945	3.46E-31	postive
LINC00472	AC005013.1	0.400741482	3.28E-22	postive
ATM	AC005013.1	0.606469003	1.91E-55	postive
FBXW7	AC005013.1	0.435241039	2.54E-26	postive
DUOX1	AC004846.1	0.474765442	1.19E-31	postive
TFAP2C	ARF4-AS1	0.457414283	3.16E-29	postive
HBA1	ARF4-AS1	0.431193146	8.16E-26	postive
FANCD2	TRG-AS1	0.433583583	4.10E-26	postive
PML	TRG-AS1	0.436782591	1.62E-26	postive
GCH1	TRG-AS1	0.483750796	5.79E-33	postive
IFNG	TRG-AS1	0.661664385	3.64E-69	postive
TNFAIP3	TRG-AS1	0.431327353	7.85E-26	postive
ENPP2	AC007998.3	0.476745337	6.15E-32	postive
SLC1A4	AC007998.3	0.435573479	2.30E-26	postive
HELLS	LINC00271	0.401639463	2.60E-22	postive
TUBE1	LINC00271	0.574979293	9.37E-49	postive
MAP3K5	LINC00271	0.402420701	2.12E-22	postive
IREB2	LINC00271	0.488965689	9.62E-34	postive
GABPB1	LINC00271	0.459854545	1.47E-29	postive
MAPK8	LINC00271	0.512086017	2.30E-37	postive
LINC00472	LINC00271	0.459438597	1.68E-29	postive
PRKAA2	LINC00271	0.543561817	9.17E-43	postive
PRKAA1	LINC00271	0.426052686	3.52E-25	postive
ATM	LINC00271	0.653542166	5.77E-67	postive
BRD4	LINC00926	0.452682073	1.37E-28	postive
ALOX12	LINC00926	0.448305249	5.24E-28	postive
PHKG2	LINC00926	0.474774203	1.18E-31	postive
TAZ	LINC00926	0.58111303	5.31E-50	postive
FBXW7	LINC00926	0.420577012	1.62E-24	postive
TRIB3	AC100872.2	0.464552295	3.30E-30	postive
IL6	AC100872.2	0.402073765	2.32E-22	postive
STEAP3	AC100872.2	0.404692955	1.17E-22	postive
HELLS	AC025423.1	0.539959254	4.07E-42	postive
TUBE1	AC025423.1	0.440447783	5.51E-27	postive
ALOX12	AC025423.1	0.433199714	4.58E-26	postive
GABPB1	AC025423.1	0.525438801	1.38E-39	postive
ATG7	AC025423.1	0.472234736	2.74E-31	postive
LINC00472	AC025423.1	0.598001231	1.42E-53	postive
ATM	AC025423.1	0.748171126	9.83E-98	postive
FBXW7	AC025423.1	0.499604272	2.24E-35	postive
NCF2	AC008972.2	0.442718612	2.81E-27	postive
CYBB	AC008972.2	0.405508379	9.47E-23	postive
ATG7	AC008972.2	0.433587887	4.10E-26	postive
ALOX15B	AC008972.2	0.426093167	3.48E-25	postive
HELLS	AC021205.3	0.467408972	1.32E-30	postive
ZNF419	AC021205.3	0.509518965	5.99E-37	postive
VEGFA	AC021205.3	0.416626991	4.81E-24	postive
TUBE1	AC021205.3	0.718272091	1.21E-86	postive
ALOX12	AC021205.3	0.587304724	2.76E-51	postive
GABPB1	AC021205.3	0.558334101	1.68E-45	postive
MAPK8	AC021205.3	0.420508274	1.65E-24	postive
LINC00472	AC021205.3	0.430959422	8.72E-26	postive
ATM	AC021205.3	0.686965415	1.75E-76	postive
FBXW7	AC021205.3	0.517554067	2.91E-38	postive
ISCU	LINC00265	0.464312126	3.57E-30	postive

CS	LINC00265	0.406044139	8.22E-23	postive
ATG4D	LINC00265	0.505741889	2.42E-36	postive
GABARAPL1	LINC00265	0.566931208	3.70E-47	postive
LPIN1	LINC00265	0.63961954	2.39E-63	postive
SLC3A2	AP003068.2	0.462148474	7.11E-30	postive
FH	AP003068.2	0.428746541	1.64E-25	postive
ISCU	AP003068.2	0.487631779	1.53E-33	postive
ATP5MC3	AP003068.2	0.410525092	2.50E-23	postive
GOT1	AP003068.2	0.488524323	1.12E-33	postive
ATG4D	AP003068.2	0.485561664	3.12E-33	postive
GABARAPL2	AP003068.2	0.462153629	7.10E-30	postive
GABARAPL1	AP003068.2	0.430121464	1.11E-25	postive
MAPK14	LINC00654	0.433691132	3.97E-26	postive
BACH1	LINC00654	0.404405613	1.26E-22	postive
HELLS	JMJD1C-AS1	0.549622658	7.17E-44	postive
ALOX12	JMJD1C-AS1	0.404147094	1.35E-22	postive
GABPB1	JMJD1C-AS1	0.473749436	1.66E-31	postive
ATM	JMJD1C-AS1	0.460274303	1.29E-29	postive
VEGFA	AC009495.1	0.435886652	2.10E-26	postive
TUBE1	AC009495.1	0.501031638	1.34E-35	postive
ALOX12	AC009495.1	0.408371266	4.44E-23	postive
DDIT3	LINC00482	0.406363756	7.56E-23	postive
ATG4D	LINC00482	0.401937804	2.41E-22	postive
ZNF419	AC068620.2	0.539242734	5.46E-42	postive
TUBE1	AC068620.2	0.472829648	2.25E-31	postive
ALOX12	AC068620.2	0.401068689	3.02E-22	postive
GABPB1	AC068620.2	0.419045978	2.48E-24	postive
PHKG2	AC068620.2	0.565692386	6.45E-47	postive
TAZ	AC068620.2	0.643229805	2.87E-64	postive
HELLS	AC010530.1	0.42593194	3.64E-25	postive
ZNF419	AC010530.1	0.452795584	1.33E-28	postive
TUBE1	AC010530.1	0.411475572	1.94E-23	postive
ALOX12	AC010530.1	0.52385346	2.56E-39	postive
GABPB1	AC010530.1	0.459257042	1.77E-29	postive
LINC00472	AC010530.1	0.660707202	6.66E-69	postive
LPIN1	AC010530.1	0.411733102	1.81E-23	postive
ATM	AC010530.1	0.464478635	3.38E-30	postive
FBXW7	AC010530.1	0.417580395	3.70E-24	postive
VEGFA	ZNF30-AS1	0.460496676	1.20E-29	postive
TUBE1	ZNF30-AS1	0.469240316	7.28E-31	postive
ALOX12	ZNF30-AS1	0.568714514	1.65E-47	postive
ATM	ZNF30-AS1	0.448559402	4.85E-28	postive
NOX1	AC083799.1	0.499340644	2.47E-35	postive
NOX1	AC055822.1	0.460473957	1.21E-29	postive
BECN1	AC055822.1	-0.468999088	7.87E-31	negative
TAZ	DLGAP1-AS1	0.403899982	1.44E-22	postive
ZNF419	AC012313.2	0.49675454	6.22E-35	postive
SETD1B	AC012313.2	0.510244096	4.58E-37	postive
VEGFA	AL645608.7	0.449817302	3.31E-28	postive
TAZ	AL645608.7	0.401223965	2.90E-22	postive
BID	PRKAR1B-AS	0.40325678	1.71E-22	postive
ZNF419	CAHM	0.428329901	1.85E-25	postive
TUBE1	CAHM	0.432635886	5.39E-26	postive
PHKG2	CAHM	0.541358341	2.29E-42	postive
TAZ	CAHM	0.675267515	5.22E-73	postive
FH	B4GALT1-AS	0.470474177	4.87E-31	postive
SLC2A8	B4GALT1-AS	0.488908097	9.82E-34	postive
MAP1LC3A	B4GALT1-AS	0.437964823	1.15E-26	postive

GABARAPL2	B4GALT1-AS	0.55742109	2.50E-45	postive
HMGB1	AP001318.2	0.423333325	7.54E-25	postive
HSPB1	PPP1R14B-A	0.585938882	5.32E-51	postive
SLC2A6	PPP1R14B-A	0.460070924	1.37E-29	postive
HRAS	PPP1R14B-A	0.423402921	7.40E-25	postive
SIRT1	PPP1R14B-A	-0.428259393	1.88E-25	negative
TFAP2C	AP000350.5	0.576979715	3.70E-49	postive
HBA1	AP000350.5	0.477235729	5.22E-32	postive
TFAP2C	AC096540.1	0.587448836	2.57E-51	postive
HBA1	AC096540.1	0.526630612	8.65E-40	postive
BRD4	AC005387.2	0.460936527	1.04E-29	postive
ZNF419	AC005387.2	0.530455323	1.90E-40	postive
VEGFA	AC005387.2	0.480798131	1.58E-32	postive
TUBE1	AC005387.2	0.546717281	2.45E-43	postive
SETD1B	AC005387.2	0.400380857	3.60E-22	postive
DRD4	AC005387.2	0.409621912	3.18E-23	postive
ALOX12	AC005387.2	0.668246863	5.33E-71	postive
PHKG2	AC005387.2	0.467561499	1.25E-30	postive
TAZ	AC005387.2	0.624889361	1.02E-59	postive
SNX4	AC063919.1	0.443183597	2.45E-27	postive
FANCD2	AC000120.1	0.401362943	2.80E-22	postive
HELLS	AC000120.1	0.455811534	5.22E-29	postive
ALOX12	AC000120.1	0.437950684	1.15E-26	postive
TFAP2C	AC000120.1	0.502654594	7.46E-36	postive
HBA1	AC000120.1	0.45819632	2.48E-29	postive
DUOX1	AC000120.1	0.456620899	4.05E-29	postive
ZNF419	AC132192.2	0.529249891	3.07E-40	postive
TUBE1	AC132192.2	0.504044315	4.50E-36	postive
ALOX12	AC132192.2	0.558867847	1.33E-45	postive
PHKG2	AC132192.2	0.577160427	3.40E-49	postive
TAZ	AC132192.2	0.661623241	3.73E-69	postive
HELLS	AGBL5-IT1	0.415258887	6.98E-24	postive
ZNF419	AGBL5-IT1	0.487857094	1.41E-33	postive
TUBE1	AGBL5-IT1	0.423715437	6.78E-25	postive
ALOX12	AGBL5-IT1	0.525249682	1.49E-39	postive
HBA1	AGBL5-IT1	0.403089664	1.78E-22	postive
TAZ	AGBL5-IT1	0.415559413	6.43E-24	postive
TAZ	LINC02640	0.413235836	1.21E-23	postive
FANCD2	AC011461.1	0.466130173	1.99E-30	postive
HELLS	AC011461.1	0.546013744	3.29E-43	postive
ZNF419	AC011461.1	0.508958144	7.38E-37	postive
TUBE1	AC011461.1	0.499429351	2.39E-35	postive
ALOX12	AC011461.1	0.58083568	6.06E-50	postive
GABPB1	AC011461.1	0.486062005	2.62E-33	postive
LINC00472	AC011461.1	0.525162734	1.54E-39	postive
ATM	AC011461.1	0.532589745	8.11E-41	postive
FBXW7	AC011461.1	0.504455525	3.87E-36	postive
RPL8	SLC12A9-AS	0.4889337	9.73E-34	postive
HBA1	SLC12A9-AS	0.407056485	6.29E-23	postive
HELLS	AC009318.1	0.570714681	6.65E-48	postive
ZNF419	AC009318.1	0.461156479	9.74E-30	postive
TUBE1	AC009318.1	0.54951886	7.50E-44	postive
ALOX12	AC009318.1	0.612730643	7.24E-57	postive
GABPB1	AC009318.1	0.532176313	9.58E-41	postive
LINC00472	AC009318.1	0.402971676	1.84E-22	postive
ATM	AC009318.1	0.608257924	7.56E-56	postive
FBXW7	AC009318.1	0.513131313	1.55E-37	postive
ISCU	AC017100.1	0.457699857	2.89E-29	postive

HERPUD1	AC017100.1	0.421895815	1.13E-24	postive
GOT1	AC017100.1	0.408850276	3.91E-23	postive
GABARAPL1	AC017100.1	0.457468184	3.11E-29	postive
BRD4	STARD7-AS1	0.44471402	1.55E-27	postive
ZNF419	STARD7-AS1	0.453144342	1.19E-28	postive
SETD1B	STARD7-AS1	0.437438581	1.34E-26	postive
ALOX12	STARD7-AS1	0.423379212	7.45E-25	postive
LPIN1	STARD7-AS1	0.40115639	2.95E-22	postive
EGLN2	STARD7-AS1	0.449379704	3.78E-28	postive
HELLS	AC005154.4	0.551731014	2.92E-44	postive
ZNF419	AC005154.4	0.433706964	3.96E-26	postive
TUBE1	AC005154.4	0.560162798	7.53E-46	postive
ALOX12	AC005154.4	0.591561755	3.47E-52	postive
GABPB1	AC005154.4	0.551766842	2.88E-44	postive
ATG7	AC005154.4	0.401493538	2.70E-22	postive
MAPK8	AC005154.4	0.405549808	9.36E-23	postive
LINC00472	AC005154.4	0.511215747	3.18E-37	postive
ATM	AC005154.4	0.769403452	1.31E-106	postive
FBXW7	AC005154.4	0.505495943	2.64E-36	postive
BRD4	AC132872.1	0.502420523	8.12E-36	postive
ZNF419	AC132872.1	0.478652626	3.25E-32	postive
VEGFA	AC132872.1	0.418687939	2.73E-24	postive
DRD4	AC132872.1	0.418327901	3.02E-24	postive
ALOX12	AC132872.1	0.440439197	5.53E-27	postive
SCP2	AC132872.1	-0.406928764	6.51E-23	negative
NCOA4	AC132872.1	-0.457839331	2.77E-29	negative
PHKG2	AC132872.1	0.518001984	2.45E-38	postive
EGLN2	AC132872.1	0.419229273	2.35E-24	postive
TAZ	AC132872.1	0.701579281	4.63E-81	postive
IL33	LINC02202	0.483834847	5.63E-33	postive
HIC1	LINC02202	0.55820633	1.78E-45	postive
ZEB1	LINC02202	0.64143248	8.28E-64	postive
EPAS1	LINC02202	0.591336871	3.87E-52	postive
ALOX12	AC026992.1	0.400815211	3.22E-22	postive
GABARAPL1	AC026992.1	0.417881593	3.41E-24	postive
HSPB1	AC011445.1	0.487831248	1.43E-33	postive
HBA1	AC011445.1	0.576595757	4.43E-49	postive
HRAS	AC011445.1	0.763378636	5.38E-104	postive
MAP1LC3A	AC011445.1	0.439843849	6.59E-27	postive
EGLN2	AC011445.1	0.845883035	1.05E-148	postive
ZNF419	AL590096.1	0.411036821	2.18E-23	postive
TUBE1	AL590096.1	0.472671104	2.37E-31	postive
ALOX12	AL590096.1	0.601945784	1.94E-54	postive
PHKG2	AL590096.1	0.420353836	1.73E-24	postive
TAZ	AL590096.1	0.572736861	2.64E-48	postive
NOX1	AL008635.1	0.443037171	2.56E-27	postive
PHKG2	AL008635.1	0.417211465	4.10E-24	postive
BECN1	AL008635.1	-0.445329614	1.29E-27	negative
HELLS	AL592430.1	0.424558241	5.35E-25	postive
ZNF419	AL592430.1	0.405338378	9.90E-23	postive
KLHL24	AL592430.1	0.413026083	1.28E-23	postive
TUBE1	AL592430.1	0.553020429	1.68E-44	postive
ALOX12	AL592430.1	0.536714	1.53E-41	postive
IREB2	AL592430.1	0.415802316	6.02E-24	postive
GABPB1	AL592430.1	0.499230133	2.57E-35	postive
MAPK8	AL592430.1	0.494966172	1.17E-34	postive
LINC00472	AL592430.1	0.711595677	2.30E-84	postive
ATM	AL592430.1	0.60538981	3.33E-55	postive

FBXW7	AL592430.1	0.406187706	7.92E-23 postive
ALOX12	SNHG28	0.421334448	1.32E-24 postive
TFAP2C	AP003170.3	0.493862877	1.73E-34 postive
HBA1	AP003170.3	0.433112516	4.70E-26 postive
DUOX1	AP003170.3	0.411838871	1.76E-23 postive
LINC00472	AP003170.3	0.470877075	4.27E-31 postive
FANCD2	TSPOAP1-AS	0.500114817	1.87E-35 postive
PML	TSPOAP1-AS	0.424941567	4.81E-25 postive
GCH1	TSPOAP1-AS	0.410717852	2.38E-23 postive
ALOX12	TSPOAP1-AS	0.544148901	7.18E-43 postive
IFNG	TSPOAP1-AS	0.537657019	1.04E-41 postive
TNFAIP3	TSPOAP1-AS	0.422823293	8.70E-25 postive
YY1AP1	TSPOAP1-AS	0.405996291	8.32E-23 postive
TUBE1	AC093627.5	0.44207645	3.40E-27 postive
ALOX12	AC093627.5	0.460028856	1.39E-29 postive
HIC1	LINC02875	0.42916741	1.46E-25 postive
TAZ	LINC02875	0.409331723	3.44E-23 postive
HIC1	AC000067.1	0.465243089	2.65E-30 postive
MTOR	LINC00571	0.416707616	4.70E-24 postive
ACSL3	LINC00571	0.51187808	2.49E-37 postive
KLHL24	LINC00571	0.746562201	4.26E-97 postive
MAP3K5	LINC00571	0.442756034	2.78E-27 postive
EIF2AK4	LINC00571	0.414495165	8.59E-24 postive
IREB2	LINC00571	0.531722334	1.15E-40 postive
EMC2	LINC00571	0.408069183	4.81E-23 postive
PIK3CA	LINC00571	0.593506146	1.33E-52 postive
KRAS	LINC00571	0.458750199	2.08E-29 postive
SLC38A1	LINC00571	0.440156757	6.01E-27 postive
GABARAPL1	LINC00571	0.411646415	1.85E-23 postive
LINC00472	LINC00571	0.750605896	1.05E-98 postive
PRKAA1	LINC00571	0.535557298	2.45E-41 postive
TGFBR1	LINC00571	0.428011686	2.02E-25 postive
LPIN1	LINC00571	0.539816989	4.31E-42 postive
ATM	LINC00571	0.458491741	2.26E-29 postive
ZNF419	AC068473.5	0.427640081	2.25E-25 postive
FANCD2	VPS9D1-AS1	0.51932901	1.48E-38 postive
HELLS	VPS9D1-AS1	0.428256127	1.89E-25 postive
BRD4	VPS9D1-AS1	0.481348693	1.31E-32 postive
ASNS	VPS9D1-AS1	0.41841615	2.95E-24 postive
ZNF419	VPS9D1-AS1	0.414607228	8.33E-24 postive
SETD1B	VPS9D1-AS1	0.448182112	5.44E-28 postive
ALOX12	VPS9D1-AS1	0.414755174	8.00E-24 postive
PHKG2	VPS9D1-AS1	0.546787349	2.38E-43 postive
TAZ	VPS9D1-AS1	0.569090828	1.39E-47 postive
TFAP2C	AC002398.1	0.441322287	4.26E-27 postive
NCOA4	AC002398.1	-0.441836274	3.65E-27 negative
PHKG2	AC002398.1	0.466503078	1.77E-30 postive
TAZ	AC002398.1	0.487441594	1.63E-33 postive
MTOR	AC018521.6	0.419607683	2.12E-24 postive
ZNF419	AC018521.6	0.415944686	5.79E-24 postive
SETD1B	AC018521.6	0.52255852	4.24E-39 postive
ALOX12	AC018521.6	0.402263009	2.21E-22 postive
ZEB1	AC018521.6	0.484424323	4.60E-33 postive
EPAS1	AC018521.6	0.418827914	2.63E-24 postive
YY1AP1	AC018521.6	0.464542979	3.31E-30 postive
ALOX12	AL022341.1	0.430458286	1.01E-25 postive
HBA1	AL022341.1	0.408964126	3.79E-23 postive
HRAS	AL022341.1	0.464085841	3.84E-30 postive

PHKG2	AL022341.1	0.423853938	6.52E-25	postive
EGLN2	AL022341.1	0.487157037	1.80E-33	postive
TAZ	AL022341.1	0.489340863	8.45E-34	postive
BRD4	AC027601.1	0.424100919	6.09E-25	postive
ZNF419	AC027601.1	0.495088706	1.12E-34	postive
VEGFA	AC027601.1	0.442055366	3.42E-27	postive
TUBE1	AC027601.1	0.483136827	7.14E-33	postive
SETD1B	AC027601.1	0.507322733	1.35E-36	postive
DRD4	AC027601.1	0.447520756	6.64E-28	postive
ALOX12	AC027601.1	0.609671691	3.62E-56	postive
PHKG2	AC027601.1	0.613982447	3.73E-57	postive
TAZ	AC027601.1	0.758372544	6.97E-102	postive
KLHL24	AC073651.1	0.568868937	1.54E-47	postive
GABPB1	AC073651.1	0.407988875	4.92E-23	postive
PIK3CA	AC073651.1	0.46345899	4.69E-30	postive
KRAS	AC073651.1	0.411186073	2.10E-23	postive
LINC00472	AC073651.1	0.946109458	4.10E-265	postive
KLHL24	PLAC4	0.570416814	7.62E-48	postive
PIK3CA	PLAC4	0.438391397	1.01E-26	postive
LINC00472	PLAC4	0.892461368	8.77E-188	postive
TUBE1	LINC01320	0.426278976	3.30E-25	postive
ALOX12	LINC01320	0.504607499	3.66E-36	postive
PRKAA2	LINC01320	0.407439962	5.69E-23	postive
ATM	LINC01320	0.633137046	1.00E-61	postive
HELLS	AC138207.5	0.409003412	3.75E-23	postive
ALOX12	AC138207.5	0.437971022	1.14E-26	postive
ATM	AC138207.5	0.441145622	4.48E-27	postive
FBXW7	AC138207.5	0.483220647	6.94E-33	postive
GPX4	AC008443.4	0.453509167	1.06E-28	postive
STAT3	AC008443.4	-0.4081289	4.74E-23	negative
RPL8	AC008443.4	0.45882624	2.03E-29	postive
MAPK14	AC008443.4	-0.406530654	7.23E-23	negative
HRAS	AC008443.4	0.565533019	6.93E-47	postive
BECN1	AC008443.4	-0.416487067	4.99E-24	negative
SLC3A2	LINC02185	0.483329301	6.69E-33	postive
ISCU	LINC02185	0.556088028	4.47E-45	postive
DDIT3	LINC02185	0.570786059	6.44E-48	postive
HERPUD1	LINC02185	0.407013907	6.36E-23	postive
TFAP2C	LINC02185	0.42624898	3.33E-25	postive
ATG4D	LINC02185	0.447897608	5.93E-28	postive
GABARAPL1	LINC02185	0.767453975	9.37E-106	postive
LPIN1	LINC02185	0.597319297	2.00E-53	postive
TFAP2C	AC010203.2	0.6127687	7.10E-57	postive
HBA1	AC010203.2	0.514906701	7.95E-38	postive
TFAP2C	AC011825.4	0.55205333	2.54E-44	postive
HBA1	AC011825.4	0.494994927	1.16E-34	postive
BRD4	AC069281.2	0.428478866	1.77E-25	postive
SETD1B	AC069281.2	0.402233327	2.23E-22	postive
ALOX12	AC069281.2	0.438724076	9.16E-27	postive
NCOA4	AC069281.2	-0.467072178	1.47E-30	negative
PHKG2	AC069281.2	0.709722911	9.79E-84	postive
ULK1	AC069281.2	0.421478382	1.26E-24	postive
MAPK1	AC069281.2	-0.448487032	4.96E-28	negative
TAZ	AC069281.2	0.750219967	1.50E-98	postive
TUBE1	AC092134.1	0.425916616	3.66E-25	postive
PHKG2	AC092134.1	0.478187599	3.80E-32	postive
TAZ	AC092134.1	0.501348552	1.20E-35	postive
ZNF419	AC018845.3	0.468958231	7.98E-31	postive

KLHL24	AC018845.3	0.409401467	3.38E-23	postive
GABPB1	AC018845.3	0.458080523	2.57E-29	postive
LINC00472	AC018845.3	0.656941243	7.06E-68	postive
LPIN1	AC018845.3	0.492909733	2.43E-34	postive
ATM	AC018845.3	0.424940068	4.81E-25	postive
RB1	OTUD6B-AS:	0.416149787	5.48E-24	postive
HSPB1	OTUD6B-AS:	-0.400066449	3.91E-22	negative
GCLC	OTUD6B-AS:	0.483405361	6.51E-33	postive
PML	OTUD6B-AS:	-0.417327831	3.97E-24	negative
ACSL3	OTUD6B-AS:	0.623673561	1.99E-59	postive
CHMP5	OTUD6B-AS:	0.518048565	2.41E-38	postive
KLHL24	OTUD6B-AS:	0.59810434	1.35E-53	postive
MAP3K5	OTUD6B-AS:	0.422929303	8.44E-25	postive
IREB2	OTUD6B-AS:	0.615996463	1.28E-57	postive
EMC2	OTUD6B-AS:	0.735998873	4.89E-93	postive
PIK3CA	OTUD6B-AS:	0.586525457	4.01E-51	postive
SCP2	OTUD6B-AS:	0.415210685	7.07E-24	postive
KRAS	OTUD6B-AS:	0.596811496	2.58E-53	postive
ATG5	OTUD6B-AS:	0.449947644	3.18E-28	postive
GABARAPL2	OTUD6B-AS:	0.417414648	3.88E-24	postive
GABARAPL1	OTUD6B-AS:	0.426167455	3.41E-25	postive
MAPK8	OTUD6B-AS:	0.496169242	7.66E-35	postive
PRKAA2	OTUD6B-AS:	0.551121498	3.79E-44	postive
PRKAA1	OTUD6B-AS:	0.659995092	1.04E-68	postive
MTDH	OTUD6B-AS:	0.488547316	1.11E-33	postive
SIRT1	OTUD6B-AS:	0.41897404	2.53E-24	postive
HELLS	AL157932.1	0.464415868	3.45E-30	postive
ALOX12	AL157932.1	0.464711266	3.14E-30	postive
LINC00472	AL157932.1	0.441273912	4.32E-27	postive
GPX4	OIP5-AS1	-0.400533719	3.46E-22	negative
RB1	OIP5-AS1	0.652882462	8.64E-67	postive
HSPB1	OIP5-AS1	-0.479635492	2.34E-32	negative
HSF1	OIP5-AS1	-0.402243321	2.22E-22	negative
GCLC	OIP5-AS1	0.460917484	1.05E-29	postive
MTOR	OIP5-AS1	0.441456823	4.09E-27	postive
ACSL3	OIP5-AS1	0.495343142	1.03E-34	postive
OTUB1	OIP5-AS1	-0.460172938	1.33E-29	negative
OXSR1	OIP5-AS1	0.480494214	1.75E-32	postive
KLHL24	OIP5-AS1	0.680480335	1.55E-74	postive
MAP3K5	OIP5-AS1	0.514595771	8.94E-38	postive
MAPK14	OIP5-AS1	0.481141946	1.40E-32	postive
EIF2AK4	OIP5-AS1	0.461799576	7.95E-30	postive
IREB2	OIP5-AS1	0.811076728	3.68E-127	postive
SP1	OIP5-AS1	0.478834551	3.06E-32	postive
GABPB1	OIP5-AS1	0.434754844	2.92E-26	postive
EMC2	OIP5-AS1	0.503905552	4.73E-36	postive
PIK3CA	OIP5-AS1	0.735559961	7.14E-93	postive
NRAS	OIP5-AS1	0.55169664	2.96E-44	postive
KRAS	OIP5-AS1	0.656129069	1.17E-67	postive
HRAS	OIP5-AS1	-0.466442859	1.80E-30	negative
SLC38A1	OIP5-AS1	0.56943195	1.19E-47	postive
ATG5	OIP5-AS1	0.410242914	2.70E-23	postive
NCOA4	OIP5-AS1	0.525284336	1.47E-39	postive
ULK2	OIP5-AS1	0.455340356	6.04E-29	postive
MAPK1	OIP5-AS1	0.491525536	3.94E-34	postive
MAPK8	OIP5-AS1	0.600370593	4.32E-54	postive
MAPK9	OIP5-AS1	0.494283973	1.49E-34	postive
LINC00472	OIP5-AS1	0.507327036	1.35E-36	postive

PRKAA2	OIP5-AS1	0.690345094	1.62E-77	postive
PRKAA1	OIP5-AS1	0.680506097	1.52E-74	postive
TGFBR1	OIP5-AS1	0.490376942	5.89E-34	postive
ANO6	OIP5-AS1	0.417152824	4.16E-24	postive
TLR4	OIP5-AS1	0.528048029	4.95E-40	postive
ATM	OIP5-AS1	0.595859324	4.15E-53	postive
MTDH	OIP5-AS1	0.514092835	1.08E-37	postive
SIRT1	OIP5-AS1	0.543321256	1.01E-42	postive
BACH1	OIP5-AS1	0.518487437	2.04E-38	postive
ISCU	AC144831.1	0.406244989	7.80E-23	postive
DDIT3	AC144831.1	0.450700296	2.52E-28	postive
ATG4D	AC144831.1	0.490279984	6.09E-34	postive
GABARAPL1	AC144831.1	0.577551911	2.83E-49	postive
WIPI2	AC144831.1	0.451986142	1.70E-28	postive
BAP1	AC144831.1	0.443693566	2.10E-27	postive
LPIN1	AC144831.1	0.543566344	9.15E-43	postive
FANCD2	AC099778.1	0.464248444	3.64E-30	postive
ZNF419	AC099778.1	0.513437334	1.38E-37	postive
ALOX12	AC099778.1	0.512812387	1.75E-37	postive
TFAP2C	AC099778.1	0.465501305	2.44E-30	postive
HBA1	AC099778.1	0.41993001	1.94E-24	postive
DUOX1	AC099778.1	0.422872885	8.58E-25	postive
SLC3A2	AC022007.1	0.408907229	3.85E-23	postive
ISCU	AC022007.1	0.52335989	3.11E-39	postive
ASNS	AC022007.1	0.43333985	4.40E-26	postive
DDIT3	AC022007.1	0.554423535	9.19E-45	postive
RIPK1	AC022007.1	-0.410928103	2.25E-23	negative
ATG4D	AC022007.1	0.510479864	4.19E-37	postive
MAP1LC3A	AC022007.1	0.428347793	1.84E-25	postive
GABARAPL1	AC022007.1	0.555636236	5.44E-45	postive
WIPI2	AC022007.1	0.464183351	3.72E-30	postive
BAP1	AC022007.1	0.559470632	1.02E-45	postive
LPIN1	AC022007.1	0.535867908	2.16E-41	postive
ANO6	DANCR	-0.407008306	6.37E-23	negative
ZNF419	AC008982.2	0.422516009	9.48E-25	postive
VEGFA	AC008982.2	0.414647246	8.24E-24	postive
TUBE1	AC008982.2	0.444874873	1.47E-27	postive
SETD1B	AC008982.2	0.435810675	2.15E-26	postive
MAFG	AC008982.2	0.427903332	2.08E-25	postive
ALOX12	AC008982.2	0.525554157	1.32E-39	postive
ATM	AC008982.2	0.504641134	3.62E-36	postive
FBXW7	AC008982.2	0.442656948	2.86E-27	postive
FANCD2	AC023157.2	0.598916833	8.99E-54	postive
HELLS	AC023157.2	0.414528465	8.51E-24	postive
RPL8	PITPNA-AS1	0.430601039	9.67E-26	postive
ALOX12	RERG-IT1	0.44975765	3.37E-28	postive
LINC00472	RERG-IT1	0.584177265	1.24E-50	postive
ATM	RERG-IT1	0.496065728	7.95E-35	postive
HMOX1	AC110995.1	0.462606459	6.15E-30	postive
SLC40A1	AC110995.1	0.631602719	2.39E-61	postive
CYBB	AC110995.1	0.49645563	6.92E-35	postive
TLR4	AC110995.1	0.463277404	4.97E-30	postive
FANCD2	AP002807.1	0.437702193	1.24E-26	postive
BRD4	AP002807.1	0.426296543	3.28E-25	postive
ZNF419	AP002807.1	0.480820285	1.57E-32	postive
TUBE1	AP002807.1	0.424683923	5.17E-25	postive
SETD1B	AP002807.1	0.415425383	6.67E-24	postive
ALOX12	AP002807.1	0.503530555	5.42E-36	postive

PHKG2	AP002807.1	0.553924455	1.14E-44	postive
TAZ	AP002807.1	0.659190811	1.73E-68	postive
HELLS	AC097505.1	0.472320299	2.66E-31	postive
TFAP2C	AC097505.1	0.439321587	7.69E-27	postive
DUOX1	AC097505.1	0.418380998	2.97E-24	postive
ATM	AC097505.1	0.417814551	3.47E-24	postive
ZNF419	AC010504.1	0.463556366	4.54E-30	postive
LINC00472	AC010504.1	0.420031569	1.89E-24	postive
HSPB1	AC109322.1	0.487715026	1.48E-33	postive
HSF1	AC109322.1	0.435728425	2.20E-26	postive
SRC	AC109322.1	0.402589119	2.03E-22	postive
NCOA4	AC109322.1	-0.410158361	2.76E-23	negative
PHKG2	AC109322.1	0.622699728	3.39E-59	postive
MAPK1	AC109322.1	-0.416022634	5.67E-24	negative
ANO6	AC109322.1	-0.423644601	6.92E-25	negative
TAZ	AC109322.1	0.544659207	5.80E-43	postive
SIRT1	AC109322.1	-0.424879796	4.89E-25	negative
ARNTL	LINC00641	0.402285474	2.20E-22	postive
ZNF419	LINC00641	0.416673648	4.75E-24	postive
VEGFA	LINC00641	0.45895902	1.95E-29	postive
TUBE1	LINC00641	0.549569148	7.34E-44	postive
SETD1B	LINC00641	0.44194541	3.54E-27	postive
ALOX12	LINC00641	0.508411879	9.03E-37	postive
IREB2	LINC00641	0.428811249	1.61E-25	postive
GABPB1	LINC00641	0.511663548	2.69E-37	postive
ZEB1	LINC00641	0.4300857	1.12E-25	postive
MAPK8	LINC00641	0.558853953	1.34E-45	postive
LINC00472	LINC00641	0.516654332	4.10E-38	postive
ATM	LINC00641	0.639198286	3.06E-63	postive
FBXW7	LINC00641	0.446866299	8.10E-28	postive
HELLS	AC008737.1	0.500838152	1.44E-35	postive
TUBE1	AC008737.1	0.501503257	1.13E-35	postive
ALOX12	AC008737.1	0.54239969	1.49E-42	postive
GABPB1	AC008737.1	0.540868835	2.80E-42	postive
MAPK8	AC008737.1	0.42580045	3.78E-25	postive
LINC00472	AC008737.1	0.610493866	2.35E-56	postive
ATM	AC008737.1	0.740130129	1.33E-94	postive
FBXW7	AC008737.1	0.482660915	8.39E-33	postive
SLC2A12	RAMP2-AS1	0.542469966	1.44E-42	postive
PLIN4	RAMP2-AS1	0.480133152	1.97E-32	postive
ZEB1	RAMP2-AS1	0.44754581	6.59E-28	postive
HSPA5	TTC28-AS1	-0.433822504	3.83E-26	negative
ZNF419	TTC28-AS1	0.586903563	3.34E-51	postive
VEGFA	TTC28-AS1	0.629134404	9.59E-61	postive
TUBE1	TTC28-AS1	0.664867016	4.72E-70	postive
SETD1B	TTC28-AS1	0.42212921	1.06E-24	postive
ALOX12	TTC28-AS1	0.646246325	4.79E-65	postive
GABPB1	TTC28-AS1	0.445026944	1.41E-27	postive
ATM	TTC28-AS1	0.52567638	1.26E-39	postive
TAZ	TTC28-AS1	0.567601808	2.73E-47	postive
FBXW7	TTC28-AS1	0.439483924	7.33E-27	postive
HELLS	DLEU2	0.603042286	1.11E-54	postive
ZNF419	DLEU2	0.407643296	5.39E-23	postive
KLHL24	DLEU2	0.403669521	1.53E-22	postive
TUBE1	DLEU2	0.521560291	6.25E-39	postive
ALOX12	DLEU2	0.471966339	2.99E-31	postive
IREB2	DLEU2	0.403216547	1.73E-22	postive
GABPB1	DLEU2	0.650002759	5.00E-66	postive

ATG7	DLEU2	0.424296158	5.76E-25	postive
MAPK8	DLEU2	0.44668114	8.56E-28	postive
LINC00472	DLEU2	0.732121829	1.35E-91	postive
ATM	DLEU2	0.773386733	2.22E-108	postive
FBXW7	DLEU2	0.564257816	1.23E-46	postive
GABARAPL1	AC117386.2	0.487594457	1.55E-33	postive
KLHL24	GPRC5D-AS1	0.453485124	1.07E-28	postive
LINC00472	GPRC5D-AS1	0.549410342	7.85E-44	postive
LPIN1	GPRC5D-AS1	0.460953095	1.04E-29	postive
OTUB1	AC006449.6	0.441349781	4.22E-27	postive
NRAS	AC006449.6	-0.407892689	5.04E-23	negative
PHKG2	AC006449.6	0.586821806	3.48E-51	postive
MAPK1	AC006449.6	-0.412671056	1.41E-23	negative
ANO6	AC006449.6	-0.455974038	4.96E-29	negative
TAZ	AC006449.6	0.516766663	3.93E-38	postive
HELLS	AC138932.4	0.506550245	1.80E-36	postive
ZNF419	AC138932.4	0.495471932	9.82E-35	postive
TUBE1	AC138932.4	0.540842007	2.83E-42	postive
ALOX12	AC138932.4	0.584652252	9.87E-51	postive
GABPB1	AC138932.4	0.486500318	2.26E-33	postive
LINC00472	AC138932.4	0.488525018	1.12E-33	postive
ATM	AC138932.4	0.602455191	1.50E-54	postive
TAZ	AC138932.4	0.406030458	8.25E-23	postive
FBXW7	AC138932.4	0.485823635	2.85E-33	postive
ATG4D	AC068338.2	0.448234154	5.35E-28	postive
HELLS	AL596202.1	0.540602107	3.12E-42	postive
ZNF419	AL596202.1	0.445336644	1.28E-27	postive
TUBE1	AL596202.1	0.69666723	1.72E-79	postive
ALOX12	AL596202.1	0.594131607	9.79E-53	postive
GABPB1	AL596202.1	0.536528362	1.65E-41	postive
MAPK8	AL596202.1	0.419311151	2.30E-24	postive
LINC00472	AL596202.1	0.439930903	6.42E-27	postive
ATM	AL596202.1	0.670743337	1.04E-71	postive
FBXW7	AL596202.1	0.48813485	1.28E-33	postive
HELLS	USP3-AS1	0.454333412	8.25E-29	postive
ZNF419	USP3-AS1	0.433933546	3.71E-26	postive
KLHL24	USP3-AS1	0.559488893	1.01E-45	postive
TUBE1	USP3-AS1	0.502079893	9.18E-36	postive
IREB2	USP3-AS1	0.421637771	1.21E-24	postive
GABPB1	USP3-AS1	0.540586137	3.14E-42	postive
PIK3CA	USP3-AS1	0.431958595	6.55E-26	postive
MAPK8	USP3-AS1	0.415266908	6.96E-24	postive
LINC00472	USP3-AS1	0.838005165	2.19E-143	postive
LPIN1	USP3-AS1	0.454924359	6.87E-29	postive
ATM	USP3-AS1	0.645291635	8.46E-65	postive
FBXW7	USP3-AS1	0.459999505	1.40E-29	postive
PHKG2	AC092535.5	0.406916698	6.53E-23	postive
PTGS2	LINC01679	0.5010956	1.31E-35	postive
IL6	LINC01679	0.633658346	7.43E-62	postive
SLC2A3	LINC01679	0.543447748	9.62E-43	postive
FANCD2	AC025682.1	0.463053003	5.33E-30	postive
HELLS	AC025682.1	0.530556376	1.83E-40	postive
ZNF419	AC025682.1	0.411622291	1.86E-23	postive
ALOX12	AC025682.1	0.501589979	1.10E-35	postive
TFAP2C	AC025682.1	0.419162831	2.40E-24	postive
HBA1	AC025682.1	0.422533717	9.43E-25	postive
GABPB1	AC025682.1	0.411616056	1.87E-23	postive
DUOX1	AC025682.1	0.461650086	8.33E-30	postive

HELLS	AL590652.1	0.431197569	8.15E-26	postive
ZNF419	AL590652.1	0.468763725	8.50E-31	postive
VEGFA	AL590652.1	0.405960674	8.40E-23	postive
TUBE1	AL590652.1	0.574186141	1.35E-48	postive
ALOX12	AL590652.1	0.599472672	6.79E-54	postive
GABPB1	AL590652.1	0.428294937	1.87E-25	postive
ATM	AL590652.1	0.55074385	4.45E-44	postive
HELLS	AC110296.1	0.541503292	2.15E-42	postive
GABPB1	AC110296.1	0.496582649	6.62E-35	postive
ATG7	AC110296.1	0.463748811	4.27E-30	postive
LINC00472	AC110296.1	0.507566492	1.23E-36	postive
ATM	AC110296.1	0.713223363	6.49E-85	postive
FBXW7	AC110296.1	0.441234204	4.37E-27	postive
HELLS	SEPSECS-AS1	0.450906716	2.37E-28	postive
TUBE1	SEPSECS-AS1	0.502622496	7.54E-36	postive
ALOX12	SEPSECS-AS1	0.455515365	5.72E-29	postive
GABPB1	SEPSECS-AS1	0.504428983	3.91E-36	postive
MAPK8	SEPSECS-AS1	0.425196119	4.48E-25	postive
LINC00472	SEPSECS-AS1	0.488000833	1.34E-33	postive
PRKAA2	SEPSECS-AS1	0.417928269	3.37E-24	postive
ATM	SEPSECS-AS1	0.687601293	1.12E-76	postive
FBXW7	SEPSECS-AS1	0.429414689	1.36E-25	postive
GABARAPL2	ZNF561-AS1	0.436015203	2.02E-26	postive
HELLS	PAXBP1-AS1	0.445511235	1.22E-27	postive
ARNTL	PAXBP1-AS1	0.414026823	9.75E-24	postive
ZNF419	PAXBP1-AS1	0.477817084	4.30E-32	postive
VEGFA	PAXBP1-AS1	0.452956548	1.26E-28	postive
TUBE1	PAXBP1-AS1	0.642245562	5.14E-64	postive
SETD1B	PAXBP1-AS1	0.42834208	1.84E-25	postive
ALOX12	PAXBP1-AS1	0.679554866	2.90E-74	postive
IREB2	PAXBP1-AS1	0.428520805	1.75E-25	postive
SP1	PAXBP1-AS1	0.446504373	9.03E-28	postive
GABPB1	PAXBP1-AS1	0.549311464	8.19E-44	postive
MAPK8	PAXBP1-AS1	0.558951183	1.28E-45	postive
LINC00472	PAXBP1-AS1	0.515300964	6.85E-38	postive
ATM	PAXBP1-AS1	0.800242465	2.37E-121	postive
YY1AP1	PAXBP1-AS1	0.426613441	3.00E-25	postive
FBXW7	PAXBP1-AS1	0.481675508	1.17E-32	postive
ZNF419	SNHG14	0.448558289	4.85E-28	postive
KLHL24	SNHG14	0.533740444	5.11E-41	postive
TUBE1	SNHG14	0.53688441	1.43E-41	postive
ALOX12	SNHG14	0.400774398	3.26E-22	postive
IREB2	SNHG14	0.563678258	1.59E-46	postive
GABPB1	SNHG14	0.566065762	5.46E-47	postive
EMC2	SNHG14	0.419066754	2.46E-24	postive
PIK3CA	SNHG14	0.484438953	4.58E-33	postive
KRAS	SNHG14	0.41731429	3.98E-24	postive
MAPK8	SNHG14	0.527571651	5.97E-40	postive
LINC00472	SNHG14	0.775804054	1.79E-109	postive
PRKAA2	SNHG14	0.433851246	3.79E-26	postive
PRKAA1	SNHG14	0.503731013	5.04E-36	postive
ATM	SNHG14	0.643569772	2.35E-64	postive
ISCU	LINC01003	0.450528271	2.66E-28	postive
CHMP5	LINC01003	0.404170608	1.34E-22	postive
ATG4D	LINC01003	0.455776678	5.27E-29	postive
GABARAPL2	LINC01003	0.490361151	5.92E-34	postive
HELLS	AL133243.3	0.628512436	1.36E-60	postive
ZNF419	AL133243.3	0.418364761	2.99E-24	postive

TUBE1	AL133243.3	0.489726266	7.39E-34	postive
ALOX12	AL133243.3	0.457276533	3.30E-29	postive
GABPB1	AL133243.3	0.610922302	1.88E-56	postive
ATG7	AL133243.3	0.440154381	6.01E-27	postive
LINC00472	AL133243.3	0.660755488	6.46E-69	postive
ATM	AL133243.3	0.72577696	2.73E-89	postive
FBXW7	AL133243.3	0.5642009	1.26E-46	postive
ATM	AC023794.1	0.402473667	2.09E-22	postive
TRIB3	LINC02609	0.419516101	2.18E-24	postive
ZNF419	AC027271.1	0.438747574	9.10E-27	postive
NOX1	AC027271.1	0.419501356	2.18E-24	postive
PHKG2	AC027271.1	0.553445519	1.40E-44	postive
BECN1	AC027271.1	-0.451424171	2.02E-28	negative
TAZ	AC027271.1	0.570516943	7.28E-48	postive
HELLS	AL031717.1	0.441752704	3.74E-27	postive
ZNF419	AL031717.1	0.522265892	4.75E-39	postive
TUBE1	AL031717.1	0.537353806	1.18E-41	postive
SETD1B	AL031717.1	0.449673346	3.45E-28	postive
ALOX12	AL031717.1	0.627663208	2.18E-60	postive
GABPB1	AL031717.1	0.463792063	4.21E-30	postive
LINC00472	AL031717.1	0.530499994	1.87E-40	postive
ATM	AL031717.1	0.585833721	5.60E-51	postive
FBXW7	AL031717.1	0.500560584	1.59E-35	postive
HELLS	APOA1-AS	0.438952474	8.57E-27	postive
ZNF419	APOA1-AS	0.405148037	1.04E-22	postive
KLHL24	APOA1-AS	0.492564553	2.74E-34	postive
TUBE1	APOA1-AS	0.451283545	2.11E-28	postive
ALOX12	APOA1-AS	0.443133358	2.48E-27	postive
IREB2	APOA1-AS	0.458877007	2.00E-29	postive
GABPB1	APOA1-AS	0.561485307	4.20E-46	postive
PIK3CA	APOA1-AS	0.46028601	1.28E-29	postive
MAPK8	APOA1-AS	0.459660864	1.56E-29	postive
LINC00472	APOA1-AS	0.881723315	2.41E-177	postive
ATM	APOA1-AS	0.627777263	2.05E-60	postive
FBXW7	APOA1-AS	0.458463894	2.28E-29	postive
ZNF419	AL161729.4	0.555646766	5.41E-45	postive
TUBE1	AL161729.4	0.4228695	8.59E-25	postive
ALOX12	AL161729.4	0.40718986	6.07E-23	postive
PHKG2	AL161729.4	0.466406768	1.82E-30	postive
ULK1	AL161729.4	0.404093082	1.37E-22	postive
TAZ	AL161729.4	0.605342932	3.41E-55	postive
BRD4	AC084018.1	0.46568923	2.29E-30	postive
ZNF419	AC084018.1	0.563569666	1.67E-46	postive
VEGFA	AC084018.1	0.46832946	9.78E-31	postive
TUBE1	AC084018.1	0.504291979	4.11E-36	postive
SETD1B	AC084018.1	0.602297676	1.62E-54	postive
ALOX12	AC084018.1	0.609124853	4.81E-56	postive
ULK1	AC084018.1	0.418310653	3.03E-24	postive
TAZ	AC084018.1	0.548927403	9.64E-44	postive
CYBB	NCK1-DT	0.455717305	5.37E-29	postive
ATG7	NCK1-DT	0.40973378	3.09E-23	postive
TLR4	NCK1-DT	0.413497441	1.12E-23	postive
ATM	NCK1-DT	0.513052219	1.60E-37	postive
PHKG2	AP001437.1	0.436673029	1.67E-26	postive
TAZ	AP001437.1	0.468797958	8.40E-31	postive
HELLS	LINC02605	0.544815914	5.44E-43	postive
ALOX12	LINC02605	0.435001171	2.72E-26	postive
GABPB1	LINC02605	0.482808769	7.98E-33	postive

LINC00472	LINC02605	0.498768832	3.03E-35	postive
ATM	LINC02605	0.70169095	4.26E-81	postive
FBXW7	LINC02605	0.459716508	1.54E-29	postive
ZNF419	LINC01252	0.409967803	2.90E-23	postive
ALOX12	LINC01252	0.40170233	2.56E-22	postive
TFAP2C	LINC01252	0.447987045	5.77E-28	postive
GOT1	ALDH1L1-AS	0.422053189	1.08E-24	postive
PEBP1	ALDH1L1-AS	0.425326982	4.32E-25	postive
SP1	AL662791.1	0.403851839	1.46E-22	postive
HELLS	AC007319.1	0.533652841	5.29E-41	postive
TUBE1	AC007319.1	0.438317774	1.03E-26	postive
ALOX12	AC007319.1	0.420445648	1.68E-24	postive
GABPB1	AC007319.1	0.543823314	8.22E-43	postive
ATG7	AC007319.1	0.471556957	3.42E-31	postive
MAPK8	AC007319.1	0.407868116	5.08E-23	postive
LINC00472	AC007319.1	0.653396605	6.31E-67	postive
ATM	AC007319.1	0.752286758	2.20E-99	postive
FBXW7	AC007319.1	0.470837823	4.32E-31	postive
KLHL24	AC024145.1	0.518420429	2.09E-38	postive
GABPB1	AC024145.1	0.429977563	1.16E-25	postive
LINC00472	AC024145.1	0.857211563	6.73E-157	postive
LPIN1	AC024145.1	0.418554871	2.84E-24	postive
ZNF419	AC011468.5	0.448824301	4.47E-28	postive
TUBE1	AC011468.5	0.499950126	1.98E-35	postive
ALOX12	AC011468.5	0.412430248	1.50E-23	postive
GABPB1	AC011468.5	0.466002952	2.07E-30	postive
HELLS	AL353801.3	0.460829943	1.08E-29	postive
ZNF419	AL353801.3	0.458726704	2.10E-29	postive
TUBE1	AL353801.3	0.532686944	7.80E-41	postive
ALOX12	AL353801.3	0.599963576	5.30E-54	postive
GABPB1	AL353801.3	0.447572184	6.54E-28	postive
ATM	AL353801.3	0.562841791	2.30E-46	postive
TAZ	AL353801.3	0.442198292	3.28E-27	postive
FBXW7	AL353801.3	0.405864727	8.62E-23	postive
IREB2	HCG11	0.408482066	4.31E-23	postive
SP1	HCG11	0.436873269	1.58E-26	postive
PRKAA2	HCG11	0.437059109	1.49E-26	postive
ATM	HCG11	0.448934432	4.33E-28	postive
SIRT1	HCG11	0.41338458	1.16E-23	postive
HELLS	AC121761.2	0.406494478	7.30E-23	postive
TUBE1	AC121761.2	0.488472025	1.14E-33	postive
GABPB1	AC121761.2	0.405496345	9.50E-23	postive
ATM	AC121761.2	0.464139035	3.77E-30	postive
TFAP2C	AC026979.2	0.420616751	1.61E-24	postive
HBA1	AC026979.2	0.599908131	5.45E-54	postive
HRAS	AC026979.2	0.471332068	3.68E-31	postive
EGLN2	AC026979.2	0.419829752	2.00E-24	postive
HELLS	AC009948.2	0.534818473	3.31E-41	postive
GABPB1	AC009948.2	0.486912659	1.96E-33	postive
ATG7	AC009948.2	0.411084378	2.15E-23	postive
LINC00472	AC009948.2	0.480638433	1.67E-32	postive
ATM	AC009948.2	0.554702512	8.14E-45	postive
FH	AC010776.2	0.411725194	1.81E-23	postive
ISCU	AC010776.2	0.513210724	1.51E-37	postive
GPT2	AC010776.2	0.4338858	3.76E-26	postive
HERPUD1	AC010776.2	0.448809976	4.49E-28	postive
KLHL24	AC010776.2	0.470910968	4.22E-31	postive
SLC2A12	AC010776.2	0.510497509	4.16E-37	postive

GOT1	AC010776.2	0.424497588	5.45E-25	postive
ATG4D	AC010776.2	0.455383181	5.96E-29	postive
GABARAPL1	AC010776.2	0.732509362	9.73E-92	postive
WIPI2	AC010776.2	0.517002216	3.59E-38	postive
LPIN1	AC010776.2	0.524115385	2.32E-39	postive
BRD4	AC011472.1	0.45756604	3.02E-29	postive
ZNF419	AC011472.1	0.548096339	1.37E-43	postive
VEGFA	AC011472.1	0.59138615	3.78E-52	postive
TUBE1	AC011472.1	0.503680365	5.13E-36	postive
SETD1B	AC011472.1	0.441174306	4.45E-27	postive
DRD4	AC011472.1	0.414072089	9.63E-24	postive
ALOX12	AC011472.1	0.589967325	7.57E-52	postive
TAZ	AC011472.1	0.553196696	1.56E-44	postive
HELLS	NUTM2A-AS	0.497426076	4.90E-35	postive
TUBE1	NUTM2A-AS	0.437142927	1.46E-26	postive
ALOX12	NUTM2A-AS	0.417078834	4.25E-24	postive
IREB2	NUTM2A-AS	0.430271778	1.06E-25	postive
GABPB1	NUTM2A-AS	0.491942302	3.41E-34	postive
MAPK8	NUTM2A-AS	0.521121277	7.40E-39	postive
LINC00472	NUTM2A-AS	0.428453073	1.78E-25	postive
ATM	NUTM2A-AS	0.596126244	3.63E-53	postive
ISCU	AL031123.1	0.574425061	1.21E-48	postive
TMBIM4	AL031123.1	0.437133692	1.46E-26	postive
CHMP5	AL031123.1	0.439970823	6.35E-27	postive
ATG4D	AL031123.1	0.414066801	9.64E-24	postive
GABARAPL2	AL031123.1	0.425910883	3.66E-25	postive
GABARAPL1	AL031123.1	0.415778116	6.06E-24	postive
MAPK1	AC027458.1	0.404771882	1.15E-22	postive
PRKAA2	AC027458.1	0.41177974	1.79E-23	postive
ZNF419	AC023908.3	0.546521669	2.66E-43	postive
TUBE1	AC023908.3	0.573880548	1.56E-48	postive
SETD1B	AC023908.3	0.483135696	7.14E-33	postive
ALOX12	AC023908.3	0.639398206	2.72E-63	postive
GABPB1	AC023908.3	0.409717919	3.10E-23	postive
PHKG2	AC023908.3	0.515459676	6.45E-38	postive
ATM	AC023908.3	0.418595215	2.80E-24	postive
TAZ	AC023908.3	0.576838127	3.95E-49	postive
HRAS	AC025754.2	0.477151759	5.37E-32	postive
ISCU	AC007996.1	0.401104224	2.99E-22	postive
KLHL24	AC007996.1	0.613177056	5.72E-57	postive
PIK3CA	AC007996.1	0.425876462	3.70E-25	postive
KRAS	AC007996.1	0.414666551	8.20E-24	postive
GABARAPL1	AC007996.1	0.488337353	1.20E-33	postive
LINC00472	AC007996.1	0.782794919	1.03E-112	postive
LPIN1	AC007996.1	0.572593474	2.82E-48	postive
CA9	SAP30-DT	0.445161508	1.35E-27	postive
BNIP3	SAP30-DT	0.404121825	1.36E-22	postive
VEGFA	SAP30-DT	0.531877489	1.08E-40	postive
HILPDA	SAP30-DT	0.44154526	3.98E-27	postive
HELLS	AC013403.2	0.507139829	1.45E-36	postive
ZNF419	AC013403.2	0.509440608	6.17E-37	postive
TUBE1	AC013403.2	0.558345028	1.67E-45	postive
ALOX12	AC013403.2	0.62718759	2.85E-60	postive
GABPB1	AC013403.2	0.491359599	4.18E-34	postive
DUOX1	AC013403.2	0.401296499	2.84E-22	postive
LINC00472	AC013403.2	0.487013925	1.89E-33	postive
ATM	AC013403.2	0.495245733	1.06E-34	postive
FBXW7	AC013403.2	0.459000865	1.92E-29	postive

ZNF419	AC124045.1	0.525176759	1.53E-39	postive
HIC1	AC124045.1	0.402895338	1.88E-22	postive
GABPB1	AC124045.1	0.586034933	5.08E-51	postive
ZEB1	AC124045.1	0.489782925	7.24E-34	postive
LINC00472	AC124045.1	0.403433503	1.63E-22	postive
FBXW7	AC124045.1	0.476254664	7.24E-32	postive
RB1	AC011477.2	0.470393786	5.00E-31	postive
HSPB1	AC011477.2	-0.40447339	1.24E-22	negative
KLHL24	AC011477.2	0.524201702	2.24E-39	postive
MAP3K5	AC011477.2	0.446129617	1.01E-27	postive
MAPK14	AC011477.2	0.421289969	1.33E-24	postive
IREB2	AC011477.2	0.598901454	9.06E-54	postive
SP1	AC011477.2	0.512243467	2.17E-37	postive
PIK3CA	AC011477.2	0.500823688	1.45E-35	postive
KRAS	AC011477.2	0.445567971	1.20E-27	postive
SLC38A1	AC011477.2	0.428747148	1.64E-25	postive
NCOA4	AC011477.2	0.42853549	1.74E-25	postive
ULK2	AC011477.2	0.473553289	1.77E-31	postive
MAPK8	AC011477.2	0.515053076	7.52E-38	postive
MAPK9	AC011477.2	0.436059409	2.00E-26	postive
PRKAA2	AC011477.2	0.53945851	5.00E-42	postive
PRKAA1	AC011477.2	0.501904992	9.79E-36	postive
ATM	AC011477.2	0.492471036	2.83E-34	postive
SIRT1	AC011477.2	0.415286567	6.93E-24	postive
TFAP2C	LINC02255	0.587465304	2.55E-51	postive
HBA1	LINC02255	0.526837943	7.97E-40	postive
HELLS	AC084824.4	0.515670021	5.96E-38	postive
TUBE1	AC084824.4	0.510814927	3.70E-37	postive
GABPB1	AC084824.4	0.510251063	4.56E-37	postive
MAPK3	AC084824.4	-0.414234257	9.22E-24	negative
LINC00472	AC084824.4	0.60687271	1.55E-55	postive
ATM	AC084824.4	0.524397897	2.07E-39	postive
FBXW7	AC084824.4	0.441826731	3.66E-27	postive
NOX1	AC060766.3	0.404253943	1.32E-22	postive
HRAS	AC060766.3	0.423989899	6.28E-25	postive
EGLN2	AC060766.3	0.443558255	2.19E-27	postive
BAP1	AC103974.1	0.414776946	7.96E-24	postive
TFAP2C	REV3L-IT1	0.573940926	1.51E-48	postive
HBA1	REV3L-IT1	0.517518864	2.95E-38	postive
DUOX1	REV3L-IT1	0.42784729	2.12E-25	postive
HELLS	STARD13-AS	0.532576334	8.16E-41	postive
TUBE1	STARD13-AS	0.409617177	3.19E-23	postive
ALOX12	STARD13-AS	0.404034452	1.39E-22	postive
GABPB1	STARD13-AS	0.508819232	7.77E-37	postive
ATG7	STARD13-AS	0.431599283	7.26E-26	postive
LINC00472	STARD13-AS	0.582937813	2.24E-50	postive
ATM	STARD13-AS	0.719734771	3.74E-87	postive
FBXW7	STARD13-AS	0.446079233	1.03E-27	postive
HELLS	DLEU1	0.436573438	1.72E-26	postive
TUBE1	DLEU1	0.415110457	7.27E-24	postive
IREB2	DLEU1	0.437677971	1.25E-26	postive
GABPB1	DLEU1	0.412546873	1.45E-23	postive
MAPK8	DLEU1	0.445263647	1.31E-27	postive
LINC00472	DLEU1	0.5683111	1.98E-47	postive
PRKAA2	DLEU1	0.455358581	6.01E-29	postive
ATM	DLEU1	0.600321896	4.42E-54	postive
OTUB1	AC026304.1	0.446473791	9.12E-28	postive
HRAS	AC026304.1	0.445242042	1.32E-27	postive

PHKG2	AC026304.1	0.478665163	3.24E-32	postive
ZNF419	AL360181.2	0.438251809	1.05E-26	postive
VEGFA	AL360181.2	0.505071747	3.09E-36	postive
TUBE1	AL360181.2	0.488950653	9.67E-34	postive
SETD1B	AL360181.2	0.510979354	3.48E-37	postive
ALOX12	AL360181.2	0.652816997	9.00E-67	postive
PHKG2	AL360181.2	0.424874856	4.90E-25	postive
TAZ	AL360181.2	0.577771825	2.56E-49	postive
DDIT3	LINC02761	0.428157701	1.94E-25	postive
MAP1LC3A	LINC02761	0.445239341	1.32E-27	postive
TUBE1	AC093726.2	0.40881241	3.95E-23	postive
ALOX12	AC093726.2	0.429167472	1.46E-25	postive
NCOA4	AC093726.2	-0.480830281	1.56E-32	negative
PHKG2	AC093726.2	0.566343086	4.82E-47	postive
TAZ	AC093726.2	0.643254485	2.83E-64	postive
ENPP2	AC073636.1	0.511381804	2.99E-37	postive
VEGFA	AC073636.1	0.422628194	9.18E-25	postive
MAPK8	AC073636.1	0.408530821	4.26E-23	postive
ATM	AC073636.1	0.441781094	3.71E-27	postive
SIRT1	AC073636.1	0.410037703	2.85E-23	postive
HBA1	AC099791.2	0.440404998	5.58E-27	postive
HRAS	AC099791.2	0.46821528	1.02E-30	postive
PHKG2	AC099791.2	0.500686558	1.52E-35	postive
EGLN2	AC099791.2	0.482252158	9.64E-33	postive
TAZ	AC099791.2	0.528157774	4.74E-40	postive
HELLS	AC025164.1	0.507537447	1.25E-36	postive
TUBE1	AC025164.1	0.493280421	2.13E-34	postive
ALOX12	AC025164.1	0.533401948	5.85E-41	postive
GABPB1	AC025164.1	0.447733664	6.23E-28	postive
ATM	AC025164.1	0.623533294	2.15E-59	postive
FBXW7	AC025164.1	0.431111819	8.35E-26	postive
HELLS	AC027607.1	0.406905192	6.55E-23	postive
ZNF419	AC027607.1	0.460745866	1.11E-29	postive
TUBE1	AC027607.1	0.504525809	3.77E-36	postive
ALOX12	AC027607.1	0.474934277	1.12E-31	postive
GABPB1	AC027607.1	0.536017482	2.04E-41	postive
LINC00472	AC027607.1	0.681232939	9.25E-75	postive
ATM	AC027607.1	0.575624429	6.95E-49	postive
FBXW7	AC027607.1	0.511999889	2.37E-37	postive
CHMP6	AC067852.2	0.504713392	3.52E-36	postive
BAP1	AC067852.2	0.422920717	8.46E-25	postive
HELLS	MANEA-DT	0.556397033	3.91E-45	postive
ZNF419	MANEA-DT	0.528159455	4.73E-40	postive
TUBE1	MANEA-DT	0.705740414	2.04E-82	postive
ALOX12	MANEA-DT	0.656650511	8.45E-68	postive
GABPB1	MANEA-DT	0.60874009	5.88E-56	postive
MAPK8	MANEA-DT	0.416559248	4.90E-24	postive
LINC00472	MANEA-DT	0.481195762	1.38E-32	postive
ATM	MANEA-DT	0.753593403	6.49E-100	postive
FBXW7	MANEA-DT	0.547173079	2.02E-43	postive
SLC40A1	AC139530.1	-0.404128349	1.36E-22	negative
OTUB1	AC139530.1	0.401241584	2.88E-22	postive
LAMP2	AC139530.1	-0.417518647	3.77E-24	negative
CHMP6	AC139530.1	0.410440912	2.56E-23	postive
ZNF419	AC139530.1	0.403243723	1.71E-22	postive
DRD4	AC139530.1	0.437928896	1.16E-26	postive
ALOX12	AC139530.1	0.457442796	3.14E-29	postive
NRAS	AC139530.1	-0.418866889	2.60E-24	negative

HRAS	AC139530.1	0.55880053	1.37E-45	postive
NCOA4	AC139530.1	-0.474347704	1.36E-31	negative
PHKG2	AC139530.1	0.618780914	2.86E-58	postive
ANO6	AC139530.1	-0.459843655	1.48E-29	negative
EGLN2	AC139530.1	0.438509947	9.76E-27	postive
TAZ	AC139530.1	0.706314968	1.32E-82	postive
MTDH	AC139530.1	-0.454504051	7.83E-29	negative
BACH1	AC139530.1	-0.424433101	5.55E-25	negative
FANCD2	AL021707.7	0.405023993	1.08E-22	postive
ALOX12	AL021707.7	0.440471117	5.47E-27	postive
TFAP2C	AL021707.7	0.531430408	1.29E-40	postive
HBA1	AL021707.7	0.477824019	4.29E-32	postive
DUOX1	AL021707.7	0.427972393	2.04E-25	postive
ZNF419	AC005479.2	0.404023697	1.40E-22	postive
ALOX12	AC005479.2	0.422599051	9.26E-25	postive
ATM	AC005479.2	0.413641057	1.08E-23	postive
ISCU	AC009570.1	0.455264051	6.18E-29	postive
ATG4D	AC009570.1	0.458443656	2.29E-29	postive
GABARAPL1	AC009570.1	0.50456621	3.72E-36	postive
LINC00472	AC009570.1	0.489309174	8.54E-34	postive
HELLS	AC073218.1	0.437580211	1.28E-26	postive
ALOX12	AC073218.1	0.438675794	9.29E-27	postive
ATM	AC073218.1	0.586411474	4.24E-51	postive
VEGFA	LMNTD2-AS	0.478803882	3.09E-32	postive
TUBE1	LMNTD2-AS	0.432748208	5.22E-26	postive
ALOX12	LMNTD2-AS	0.484198015	4.97E-33	postive
PHKG2	LMNTD2-AS	0.519538558	1.36E-38	postive
TAZ	LMNTD2-AS	0.640384417	1.53E-63	postive
HELLS	AL513550.1	0.505373649	2.77E-36	postive
ZNF419	AL513550.1	0.436205381	1.92E-26	postive
KLHL24	AL513550.1	0.415748447	6.11E-24	postive
TUBE1	AL513550.1	0.517012588	3.58E-38	postive
ALOX12	AL513550.1	0.517791321	2.66E-38	postive
IREB2	AL513550.1	0.403283145	1.70E-22	postive
GABPB1	AL513550.1	0.533194871	6.36E-41	postive
MAPK8	AL513550.1	0.406644212	7.02E-23	postive
LINC00472	AL513550.1	0.510556637	4.07E-37	postive
LPIN1	AL513550.1	0.407165377	6.11E-23	postive
ATM	AL513550.1	0.611129756	1.68E-56	postive
FBXW7	AL513550.1	0.464365148	3.51E-30	postive
HELLS	MIR155HG	0.423937517	6.37E-25	postive
PHKG2	MIR155HG	0.410649765	2.42E-23	postive
IFNG	MIR155HG	0.511864785	2.50E-37	postive
ATM	MIR155HG	0.402570713	2.04E-22	postive
TAZ	MIR155HG	0.420112624	1.85E-24	postive
FBXW7	MIR155HG	0.436400393	1.81E-26	postive
VEGFA	LINC01126	0.466814453	1.60E-30	postive
TUBE1	LINC01126	0.564853187	9.40E-47	postive
ALOX12	LINC01126	0.664867163	4.72E-70	postive
ATM	LINC01126	0.533669232	5.26E-41	postive
TAZ	LINC01126	0.434665122	3.00E-26	postive
FANCD2	NUP50-DT	0.500920776	1.40E-35	postive
ISCU	NUP50-DT	0.455990489	4.93E-29	postive
ASNS	NUP50-DT	0.449070072	4.15E-28	postive
DDIT3	NUP50-DT	0.403135433	1.76E-22	postive
PHKG2	NUP50-DT	0.463935009	4.03E-30	postive
ATG4D	NUP50-DT	0.425612651	3.98E-25	postive
MAPK3	NUP50-DT	-0.439220824	7.92E-27	negative

LPIN1	NUP50-DT	0.565111303	8.37E-47	postive
TAZ	NUP50-DT	0.410153019	2.76E-23	postive
GPX4	PRR34-AS1	0.628543298	1.34E-60	postive
HSPB1	PRR34-AS1	0.46920221	7.37E-31	postive
STAT3	PRR34-AS1	-0.468534039	9.16E-31	negative
RPL8	PRR34-AS1	0.424324476	5.72E-25	postive
MAPK14	PRR34-AS1	-0.427544969	2.31E-25	negative
SLC2A8	PRR34-AS1	0.401196527	2.92E-22	postive
NRAS	PRR34-AS1	-0.403433413	1.63E-22	negative
HRAS	PRR34-AS1	0.656310682	1.04E-67	postive
BECN1	PRR34-AS1	-0.524815449	1.76E-39	negative
MAP1LC3A	PRR34-AS1	0.510604261	4.00E-37	postive
EGLN2	PRR34-AS1	0.53717004	1.27E-41	postive
SIRT1	PRR34-AS1	-0.401861874	2.46E-22	negative
HELLS	AC016747.2	0.52613398	1.05E-39	postive
ZNF419	AC016747.2	0.493520138	1.96E-34	postive
TUBE1	AC016747.2	0.541412061	2.24E-42	postive
ALOX12	AC016747.2	0.68306592	2.63E-75	postive
GABPB1	AC016747.2	0.444156915	1.83E-27	postive
ATM	AC016747.2	0.567293899	3.14E-47	postive
FBXW7	AC016747.2	0.459904306	1.45E-29	postive
ALOX12	FIGNL2-DT	0.5065689	1.78E-36	postive
TAZ	FIGNL2-DT	0.458262594	2.43E-29	postive
HELLS	AC116651.1	0.451104604	2.23E-28	postive
GABPB1	AC116651.1	0.414125875	9.49E-24	postive
LINC00472	AC116651.1	0.447020833	7.73E-28	postive
ATM	AC116651.1	0.616164347	1.17E-57	postive
IFNG	AL158071.3	0.521537096	6.30E-39	postive
TUBE1	AL035404.2	0.40535337	9.86E-23	postive
ALOX12	AL035404.2	0.477996808	4.05E-32	postive
GABPB1	AL035404.2	0.484937134	3.86E-33	postive
LINC00472	AL035404.2	0.493146741	2.23E-34	postive
ATM	AL035404.2	0.552043295	2.56E-44	postive
FBXW7	AL035404.2	0.43830582	1.04E-26	postive
SP1	AC226118.1	0.447965409	5.81E-28	postive
ATM	AC024588.1	0.40733958	5.84E-23	postive
TFAP2C	AC002094.4	0.578846899	1.55E-49	postive
HBA1	AC002094.4	0.520548311	9.23E-39	postive
DUOX1	AC002094.4	0.420924681	1.47E-24	postive
TXNIP	USP27X-AS1	0.431187404	8.17E-26	postive
CISD1	IQCH-AS1	0.414850234	7.80E-24	postive
FH	IQCH-AS1	0.449191119	4.00E-28	postive
ISCU	IQCH-AS1	0.616712036	8.70E-58	postive
ACSL3	IQCH-AS1	0.416945753	4.41E-24	postive
KLHL24	IQCH-AS1	0.580213227	8.13E-50	postive
GOT1	IQCH-AS1	0.446602504	8.77E-28	postive
ATG4D	IQCH-AS1	0.494439067	1.42E-34	postive
GABARAPL2	IQCH-AS1	0.474597922	1.25E-31	postive
GABARAPL1	IQCH-AS1	0.594453525	8.34E-53	postive
WIPI2	IQCH-AS1	0.414206211	9.29E-24	postive
MAPK3	IQCH-AS1	-0.456205628	4.61E-29	negative
LINC00472	IQCH-AS1	0.53263669	7.96E-41	postive
LPIN1	IQCH-AS1	0.594387285	8.62E-53	postive
TAZ	ZFAND2A-D	0.481773103	1.13E-32	postive
ZNF419	AL139123.1	0.533103088	6.60E-41	postive
TUBE1	AL139123.1	0.512241352	2.17E-37	postive
ALOX12	AL139123.1	0.553411396	1.42E-44	postive
PHKG2	AL139123.1	0.606129993	2.28E-55	postive

TAZ	AL139123.1	0.728750308	2.31E-90	postive
ZNF419	AC055855.1	0.600641982	3.76E-54	postive
VEGFA	AC055855.1	0.503951595	4.65E-36	postive
TUBE1	AC055855.1	0.627270699	2.72E-60	postive
SETD1B	AC055855.1	0.48837468	1.18E-33	postive
ALOX12	AC055855.1	0.611616887	1.30E-56	postive
GABPB1	AC055855.1	0.403009803	1.82E-22	postive
PHKG2	AC055855.1	0.470652537	4.59E-31	postive
ATM	AC055855.1	0.442213646	3.27E-27	postive
TAZ	AC055855.1	0.63823939	5.33E-63	postive
FBXW7	AC055855.1	0.469444012	6.81E-31	postive
PML	TFAP2E-AS1	0.501927732	9.71E-36	postive
SLC2A6	TFAP2E-AS1	0.462230122	6.93E-30	postive
PHKG2	TFAP2E-AS1	0.430135688	1.10E-25	postive
IFNG	TFAP2E-AS1	0.587770661	2.20E-51	postive
TAZ	TFAP2E-AS1	0.429739412	1.24E-25	postive
HELLS	AC012404.2	0.472442222	2.56E-31	postive
KLHL24	AC012404.2	0.442919462	2.65E-27	postive
TUBE1	AC012404.2	0.402088609	2.31E-22	postive
IREB2	AC012404.2	0.411183759	2.10E-23	postive
GABPB1	AC012404.2	0.541896126	1.83E-42	postive
PIK3CA	AC012404.2	0.425681662	3.91E-25	postive
ATG7	AC012404.2	0.411483594	1.93E-23	postive
MAPK8	AC012404.2	0.404909028	1.11E-22	postive
LINC00472	AC012404.2	0.845259493	2.85E-148	postive
ATM	AC012404.2	0.658832053	2.17E-68	postive
FBXW7	AC012404.2	0.412449065	1.49E-23	postive
AGPAT3	LINC01014	0.494032502	1.63E-34	postive
ACO1	LINC01014	0.435998527	2.03E-26	postive
ZNF419	AC233728.1	0.52312554	3.40E-39	postive
TUBE1	AC233728.1	0.482118883	1.01E-32	postive
SETD1B	AC233728.1	0.449061918	4.16E-28	postive
ALOX12	AC233728.1	0.50037813	1.70E-35	postive
PHKG2	AC233728.1	0.521821859	5.64E-39	postive
TAZ	AC233728.1	0.639349056	2.80E-63	postive
TFAP2C	AC092171.5	0.559154584	1.17E-45	postive
HBA1	AC092171.5	0.476124036	7.56E-32	postive
HELLS	AF129075.1	0.576659023	4.30E-49	postive
ZNF419	AF129075.1	0.507597242	1.22E-36	postive
TUBE1	AF129075.1	0.610025432	3.01E-56	postive
ALOX12	AF129075.1	0.586707534	3.68E-51	postive
GABPB1	AF129075.1	0.619175692	2.31E-58	postive
MAPK8	AF129075.1	0.488545281	1.11E-33	postive
LINC00472	AF129075.1	0.685124834	6.32E-76	postive
ATM	AF129075.1	0.725707537	2.89E-89	postive
FBXW7	AF129075.1	0.545152735	4.72E-43	postive
SLC3A2	AC011523.1	0.452688699	1.37E-28	postive
FH	AC011523.1	0.451452783	2.00E-28	postive
ISCU	AC011523.1	0.578909563	1.50E-49	postive
DDIT3	AC011523.1	0.476514841	6.64E-32	postive
KLHL24	AC011523.1	0.440945889	4.76E-27	postive
SLC2A8	AC011523.1	0.509768277	5.46E-37	postive
GOT1	AC011523.1	0.42640275	3.19E-25	postive
ATG4D	AC011523.1	0.590934866	4.72E-52	postive
MAP1LC3A	AC011523.1	0.46035264	1.26E-29	postive
GABARAPL2	AC011523.1	0.444439329	1.68E-27	postive
GABARAPL1	AC011523.1	0.620379265	1.20E-58	postive
WIPI2	AC011523.1	0.496031177	8.05E-35	postive

LPIN1	AC011523.1	0.581801909	3.84E-50	postive
SP1	AL096799.1	0.418345361	3.00E-24	postive
PRKAA2	AL096799.1	0.432826073	5.10E-26	postive
ATM	AL096799.1	0.535560859	2.45E-41	postive
PHKG2	ELF3-AS1	0.560759291	5.79E-46	postive
TAZ	ELF3-AS1	0.558855676	1.34E-45	postive
HELLS	AC027097.2	0.487477025	1.61E-33	postive
TUBE1	AC027097.2	0.45215005	1.62E-28	postive
ALOX12	AC027097.2	0.579555439	1.11E-49	postive
GABPB1	AC027097.2	0.453321091	1.13E-28	postive
ATM	AC027097.2	0.576763821	4.09E-49	postive
FBXW7	AC027097.2	0.43843979	9.96E-27	postive
HELLS	AL158166.1	0.507149047	1.44E-36	postive
GABPB1	AL158166.1	0.455614338	5.55E-29	postive
ATM	AL158166.1	0.531097371	1.47E-40	postive
FBXW7	AL158166.1	0.409706452	3.11E-23	postive
ALOX12	AL590822.1	0.496174601	7.65E-35	postive
ALOX12	AL662797.1	0.407433716	5.70E-23	postive
NCOA4	AL662797.1	-0.417564143	3.72E-24	negative
PHKG2	AL662797.1	0.60176438	2.13E-54	postive
TAZ	AL662797.1	0.658086488	3.46E-68	postive
CHMP6	LBX2-AS1	0.55871882	1.42E-45	postive
PEBP1	LBX2-AS1	0.449024287	4.21E-28	postive
MIOX	LBX2-AS1	0.433541323	4.15E-26	postive
FANCD2	AL592494.2	0.477427767	4.90E-32	postive
MUC1	ERVE-1	0.401209935	2.91E-22	postive
GABARAPL1	ERVE-1	0.521956349	5.36E-39	postive
LPIN1	ERVE-1	0.515815366	5.64E-38	postive
TFAP2C	AC108134.4	0.565621926	6.66E-47	postive
HBA1	AC108134.4	0.573307768	2.03E-48	postive
DUOX1	AC108134.4	0.40352187	1.59E-22	postive
HSPB1	AL023803.1	0.440193592	5.94E-27	postive
HBA1	AL023803.1	0.619032927	2.50E-58	postive
HRAS	AL023803.1	0.718873572	7.45E-87	postive
MAP1LC3A	AL023803.1	0.442126484	3.35E-27	postive
EGLN2	AL023803.1	0.844829531	5.64E-148	postive
HELLS	AC010168.2	0.458953597	1.95E-29	postive
ZNF419	AC010168.2	0.539647185	4.63E-42	postive
TUBE1	AC010168.2	0.545230628	4.57E-43	postive
ALOX12	AC010168.2	0.594524841	8.05E-53	postive
GABPB1	AC010168.2	0.54019326	3.70E-42	postive
LINC00472	AC010168.2	0.697842934	7.28E-80	postive
ATM	AC010168.2	0.583891423	1.42E-50	postive
FBXW7	AC010168.2	0.452370465	1.51E-28	postive
ZNF419	NDUFA6-DT	0.532258563	9.26E-41	postive
VEGFA	NDUFA6-DT	0.438423967	1.00E-26	postive
TUBE1	NDUFA6-DT	0.571160572	5.43E-48	postive
SETD1B	NDUFA6-DT	0.432583546	5.47E-26	postive
ALOX12	NDUFA6-DT	0.724313515	9.10E-89	postive
GABPB1	NDUFA6-DT	0.419308287	2.30E-24	postive
ATM	NDUFA6-DT	0.538894867	6.30E-42	postive
TAZ	NDUFA6-DT	0.401305538	2.84E-22	postive
FBXW7	NDUFA6-DT	0.408563474	4.22E-23	postive
HELLS	CKMT2-AS1	0.426275007	3.30E-25	postive
ZNF419	CKMT2-AS1	0.476387493	6.93E-32	postive
TUBE1	CKMT2-AS1	0.442157013	3.32E-27	postive
ALOX12	CKMT2-AS1	0.558730841	1.41E-45	postive
GABPB1	CKMT2-AS1	0.407508802	5.58E-23	postive

MAPK8	CKMT2-AS1	0.410099487	2.80E-23	postive
ATM	CKMT2-AS1	0.407342565	5.83E-23	postive
TUBE1	AC112484.1	0.407899922	5.03E-23	postive
SETD1B	AC112484.1	0.402070591	2.33E-22	postive
ALOX12	AC112484.1	0.484755937	4.11E-33	postive
TAZ	AC112484.1	0.465076852	2.79E-30	postive
HELLS	AC129510.1	0.405598982	9.24E-23	postive
ZNF419	AC129510.1	0.490154964	6.36E-34	postive
VEGFA	AC129510.1	0.463374225	4.82E-30	postive
TUBE1	AC129510.1	0.646085812	5.27E-65	postive
SETD1B	AC129510.1	0.510967511	3.49E-37	postive
DRD4	AC129510.1	0.433556666	4.13E-26	postive
ALOX12	AC129510.1	0.683049228	2.66E-75	postive
PHKG2	AC129510.1	0.532933013	7.07E-41	postive
ATM	AC129510.1	0.416461818	5.03E-24	postive
TAZ	AC129510.1	0.653170362	7.25E-67	postive
FBXW7	AC129510.1	0.403449375	1.62E-22	postive
TMBIM4	UBR5-AS1	0.467207913	1.41E-30	postive
KLHL24	UBR5-AS1	0.420208646	1.80E-24	postive
TUBE1	UBR5-AS1	0.411439972	1.96E-23	postive
HMGB1	UBR5-AS1	0.428474285	1.77E-25	postive
GABPB1	UBR5-AS1	0.450600551	2.60E-28	postive
EMC2	UBR5-AS1	0.475278339	1.00E-31	postive
ACSL4	UBR5-AS1	0.409843923	3.00E-23	postive
KRAS	UBR5-AS1	0.452833873	1.31E-28	postive
LINC00472	UBR5-AS1	0.437634927	1.26E-26	postive
PRKAA1	UBR5-AS1	0.414874476	7.75E-24	postive
MTOR	AC124312.2	0.493797324	1.77E-34	postive
OXSR1	AC124312.2	0.44377681	2.05E-27	postive
KLHL24	AC124312.2	0.586957948	3.26E-51	postive
TUBE1	AC124312.2	0.426371784	3.22E-25	postive
MAP3K5	AC124312.2	0.438709793	9.20E-27	postive
IREB2	AC124312.2	0.687622716	1.11E-76	postive
SP1	AC124312.2	0.404612224	1.20E-22	postive
GABPB1	AC124312.2	0.497712184	4.42E-35	postive
PIK3CA	AC124312.2	0.622321384	4.17E-59	postive
KRAS	AC124312.2	0.490163623	6.34E-34	postive
SLC38A1	AC124312.2	0.405476201	9.55E-23	postive
ZEB1	AC124312.2	0.426762365	2.88E-25	postive
MAPK8	AC124312.2	0.601237282	2.78E-54	postive
LINC00472	AC124312.2	0.709707144	9.91E-84	postive
PRKAA2	AC124312.2	0.562064056	3.25E-46	postive
PRKAA1	AC124312.2	0.55814134	1.83E-45	postive
TLR4	AC124312.2	0.401222599	2.90E-22	postive
ATM	AC124312.2	0.688150316	7.63E-77	postive
FBXW7	AC124312.2	0.438911005	8.67E-27	postive
HELLS	AC105105.3	0.516257039	4.77E-38	postive
ALOX12	AC105105.3	0.515955631	5.34E-38	postive
ATM	AC105105.3	0.55102285	3.95E-44	postive
HELLS	LINC00390	0.474632597	1.24E-31	postive
TUBE1	LINC00390	0.405570622	9.31E-23	postive
ALOX12	LINC00390	0.406060385	8.19E-23	postive
GABPB1	LINC00390	0.413907209	1.01E-23	postive
LINC00472	LINC00390	0.411643388	1.85E-23	postive
ATM	LINC00390	0.651249636	2.34E-66	postive
HELLS	AC010531.3	0.437997019	1.13E-26	postive
ZNF419	AC010531.3	0.411134702	2.12E-23	postive
TUBE1	AC010531.3	0.446668275	8.60E-28	postive

ALOX12	AC010531.3	0.566898291	3.75E-47	postive
ATM	AC010531.3	0.438834993	8.87E-27	postive
FBXW7	AC010531.3	0.408933971	3.82E-23	postive
ALOX12	HLA-DQB1-/	0.434408366	3.23E-26	postive
ATM	HLA-DQB1-/	0.416513452	4.96E-24	postive
ZNF419	ZEB2-AS1	0.41405134	9.68E-24	postive
VEGFA	ZEB2-AS1	0.424852075	4.93E-25	postive
TUBE1	ZEB2-AS1	0.514523161	9.19E-38	postive
ALOX12	ZEB2-AS1	0.578488427	1.83E-49	postive
ATM	ZEB2-AS1	0.424910945	4.85E-25	postive
TAZ	ZEB2-AS1	0.576085416	5.61E-49	postive
HSPB1	AC093673.1	0.458000402	2.63E-29	postive
IREB2	AC093673.1	-0.421845212	1.14E-24	negative
SLC1A5	AC093673.1	0.474627163	1.24E-31	postive
NCOA4	AC093673.1	-0.423666228	6.87E-25	negative
PRKAA2	AC093673.1	-0.43270531	5.28E-26	negative
HELLS	AC016722.2	0.51538334	6.64E-38	postive
ZNF419	AC016722.2	0.526834663	7.98E-40	postive
TUBE1	AC016722.2	0.578574148	1.76E-49	postive
ALOX12	AC016722.2	0.670731931	1.05E-71	postive
GABPB1	AC016722.2	0.512567828	1.92E-37	postive
MAPK8	AC016722.2	0.45908839	1.87E-29	postive
LINC00472	AC016722.2	0.517782105	2.67E-38	postive
ATM	AC016722.2	0.615559842	1.61E-57	postive
FBXW7	AC016722.2	0.520955531	7.89E-39	postive
VEGFA	ATXN1-AS1	0.455465978	5.81E-29	postive
TUBE1	ATXN1-AS1	0.402230713	2.23E-22	postive
SETD1B	ATXN1-AS1	0.40916403	3.60E-23	postive
ALOX12	ATXN1-AS1	0.419929692	1.94E-24	postive
HELLS	AC017083.1	0.409010372	3.75E-23	postive
ZNF419	AC017083.1	0.509241313	6.64E-37	postive
TUBE1	AC017083.1	0.526152908	1.04E-39	postive
DRD4	AC017083.1	0.406047518	8.21E-23	postive
ALOX12	AC017083.1	0.591424209	3.71E-52	postive
PHKG2	AC017083.1	0.559724104	9.13E-46	postive
TAZ	AC017083.1	0.703138917	1.45E-81	postive
ZNF419	LINC02352	0.413149036	1.24E-23	postive
PHKG2	LINC02352	0.586807915	3.50E-51	postive
TAZ	LINC02352	0.508685659	8.16E-37	postive
BRD4	PRKCZ-AS1	0.448739407	4.59E-28	postive
ZNF419	PRKCZ-AS1	0.453619553	1.03E-28	postive
VEGFA	PRKCZ-AS1	0.538846078	6.42E-42	postive
TUBE1	PRKCZ-AS1	0.422308859	1.00E-24	postive
SETD1B	PRKCZ-AS1	0.545967783	3.36E-43	postive
ALOX12	PRKCZ-AS1	0.537681795	1.03E-41	postive
TAZ	PRKCZ-AS1	0.544656198	5.81E-43	postive
HELLS	AP000692.1	0.426013238	3.56E-25	postive
ZNF419	AP000692.1	0.589467342	9.65E-52	postive
VEGFA	AP000692.1	0.413943469	9.97E-24	postive
TUBE1	AP000692.1	0.594408531	8.53E-53	postive
SETD1B	AP000692.1	0.409475975	3.31E-23	postive
ALOX12	AP000692.1	0.614352553	3.07E-57	postive
GABPB1	AP000692.1	0.52661918	8.69E-40	postive
LINC00472	AP000692.1	0.57626211	5.17E-49	postive
ATM	AP000692.1	0.578219426	2.07E-49	postive
FBXW7	AP000692.1	0.562666365	2.49E-46	postive
ENPP2	AC074286.1	0.401623313	2.61E-22	postive
GABPB1	AC074286.1	0.420676582	1.58E-24	postive

MAPK8	AC074286.1	0.432088698	6.31E-26	postive
ATM	AC074286.1	0.568592253	1.75E-47	postive
FBXW7	AC074286.1	0.430849525	9.00E-26	postive
HELLS	AC006059.1	0.458246595	2.44E-29	postive
KLHL24	AC006059.1	0.550883084	4.20E-44	postive
IREB2	AC006059.1	0.452456458	1.47E-28	postive
GABPB1	AC006059.1	0.561000928	5.20E-46	postive
PIK3CA	AC006059.1	0.498762184	3.04E-35	postive
ATG7	AC006059.1	0.503098784	6.34E-36	postive
LINC00472	AC006059.1	0.850409352	6.76E-152	postive
TGFBR1	AC006059.1	0.446587117	8.81E-28	postive
LPIN1	AC006059.1	0.432408097	5.76E-26	postive
ATM	AC006059.1	0.641292467	8.99E-64	postive
FBXW7	AC006059.1	0.445738973	1.14E-27	postive
GOT1	AL162377.1	0.441445522	4.10E-27	postive
ATG4D	AL162377.1	0.453956454	9.27E-29	postive
ALOX12	AL603839.3	0.411323483	2.02E-23	postive
TFAP2C	AL603839.3	0.451449666	2.01E-28	postive
HBA1	AL603839.3	0.448263006	5.30E-28	postive
TFAP2C	AL109976.1	0.485982521	2.70E-33	postive
LPIN1	AL109976.1	0.467090433	1.46E-30	postive
TFAP2C	HNF4A-AS1	0.459743637	1.52E-29	postive
HBA1	HNF4A-AS1	0.417948461	3.35E-24	postive
TUBE1	AC011444.1	0.47375394	1.66E-31	postive
ALOX12	AC011444.1	0.461173848	9.69E-30	postive
TAZ	AC011444.1	0.443046634	2.55E-27	postive
IL6	AC015819.2	0.509168831	6.82E-37	postive
HELLS	AC012409.1	0.471283289	3.74E-31	postive
TUBE1	AC012409.1	0.4686568	8.80E-31	postive
ALOX12	AC012409.1	0.502966324	6.66E-36	postive
GABPB1	AC012409.1	0.473468882	1.82E-31	postive
LINC00472	AC012409.1	0.594418313	8.49E-53	postive
ATM	AC012409.1	0.660087175	9.85E-69	postive
TUBE1	MRPS30-DT	0.54078259	2.90E-42	postive
IREB2	MRPS30-DT	0.41042524	2.57E-23	postive
GABPB1	MRPS30-DT	0.499184223	2.61E-35	postive
MAPK8	MRPS30-DT	0.491250994	4.34E-34	postive
LINC00472	MRPS30-DT	0.434454779	3.19E-26	postive
PRKAA2	MRPS30-DT	0.468494798	9.27E-31	postive
PRKAA1	MRPS30-DT	0.47609825	7.63E-32	postive
ATM	MRPS30-DT	0.659473117	1.45E-68	postive
FBXW7	MRPS30-DT	0.415699868	6.19E-24	postive
HELLS	TH2LCRR	0.549300346	8.23E-44	postive
ZNF419	TH2LCRR	0.408411267	4.39E-23	postive
KLHL24	TH2LCRR	0.417113634	4.21E-24	postive
TUBE1	TH2LCRR	0.513113282	1.56E-37	postive
ALOX12	TH2LCRR	0.501411534	1.17E-35	postive
GABPB1	TH2LCRR	0.582199575	3.18E-50	postive
ATG7	TH2LCRR	0.403485666	1.61E-22	postive
MAPK8	TH2LCRR	0.415499849	6.54E-24	postive
LINC00472	TH2LCRR	0.78734929	6.87E-115	postive
ATM	TH2LCRR	0.726514003	1.49E-89	postive
FBXW7	TH2LCRR	0.496532581	6.73E-35	postive
TRIB3	AC133644.1	0.421952862	1.11E-24	postive
HELLS	AL132656.2	0.564210021	1.25E-46	postive
ZNF419	AL132656.2	0.401144169	2.96E-22	postive
TUBE1	AL132656.2	0.611461077	1.41E-56	postive
ALOX12	AL132656.2	0.488060175	1.32E-33	postive

GABPB1	AL132656.2	0.521696042	5.93E-39	postive
ATM	AL132656.2	0.510683068	3.88E-37	postive
FBXW7	AL132656.2	0.479836469	2.18E-32	postive
IFNG	AC006369.1	0.465778227	2.23E-30	postive
LINC00472	AC108058.1	0.41612665	5.51E-24	postive
NCOA4	U47924.3	-0.405246491	1.01E-22	negative
PHKG2	U47924.3	0.67668311	2.02E-73	postive
TAZ	U47924.3	0.691932457	5.23E-78	postive
GPX4	AP002360.1	0.401550169	2.66E-22	postive
OTUB1	AP002360.1	0.417000079	4.34E-24	postive
SLC2A8	AP002360.1	0.403064569	1.80E-22	postive
RIPK1	AP002360.1	-0.400753504	3.27E-22	negative
HRAS	AP002360.1	0.483297877	6.76E-33	postive
ATG4D	AP002360.1	0.442616449	2.90E-27	postive
MAP1LC3A	AP002360.1	0.46692364	1.54E-30	postive
EGLN2	AP002360.1	0.422869893	8.59E-25	postive
SLC3A2	LINC01612	0.460836862	1.08E-29	postive
FH	LINC01612	0.523365902	3.10E-39	postive
ISCU	LINC01612	0.59636475	3.23E-53	postive
DDIT3	LINC01612	0.468240853	1.01E-30	postive
GOT1	LINC01612	0.500004906	1.94E-35	postive
ATG4D	LINC01612	0.545857019	3.52E-43	postive
GABARAPL2	LINC01612	0.5571432	2.82E-45	postive
GABARAPL1	LINC01612	0.693278809	1.99E-78	postive
LPIN1	LINC01612	0.458467493	2.27E-29	postive
HIC1	SLC25A34-A[illegible]	0.427974393	2.04E-25	postive
ZNF419	AP001029.1	0.418747592	2.69E-24	postive
ALOX12	AP001029.1	0.509972735	5.06E-37	postive
PHKG2	AP001029.1	0.424727117	5.11E-25	postive
TAZ	AP001029.1	0.520707415	8.68E-39	postive
HBA1	AC135178.6	0.476089216	7.65E-32	postive
NCOA4	AC135178.6	-0.453746225	9.90E-29	negative
PHKG2	AC135178.6	0.43436943	3.27E-26	postive
EGLN2	AC135178.6	0.403408226	1.64E-22	postive
TAZ	AC135178.6	0.438387875	1.01E-26	postive
FANCD2	AC127024.4	0.417321835	3.98E-24	postive
HELLS	AC127024.4	0.597244988	2.08E-53	postive
ZNF419	AC127024.4	0.519140889	1.59E-38	postive
TUBE1	AC127024.4	0.5548416	7.67E-45	postive
ALOX12	AC127024.4	0.655951134	1.30E-67	postive
GABPB1	AC127024.4	0.548693269	1.06E-43	postive
LINC00472	AC127024.4	0.577196103	3.35E-49	postive
ATM	AC127024.4	0.625876305	5.90E-60	postive
FBXW7	AC127024.4	0.519086712	1.62E-38	postive
VEGFA	LINC01144	0.448159562	5.47E-28	postive
ALOX12	LINC01144	0.435067191	2.67E-26	postive
TAZ	LINC01144	0.46047883	1.21E-29	postive
DRD4	AC009133.1	0.416755746	4.64E-24	postive
ALOX12	AC009133.1	0.563350544	1.84E-46	postive
PHKG2	AC009133.1	0.582382127	2.91E-50	postive
TAZ	AC009133.1	0.643764153	2.10E-64	postive
MUC1	AC005082.1	0.459835143	1.48E-29	postive
ZNF419	AC020558.2	0.431362421	7.77E-26	postive
VEGFA	AC020558.2	0.44266404	2.86E-27	postive
SETD1B	AC020558.2	0.454194274	8.62E-29	postive
ALOX12	AC020558.2	0.490756097	5.16E-34	postive
TAZ	AC020558.2	0.558336462	1.68E-45	postive
ANGPTL7	ARHGAP5-A[illegible]	0.442287297	3.19E-27	postive

EIF2S1	ARHGAP5-A:	0.4226828	9.05E-25	postive
FANCD2	AC080038.1	0.525148487	1.55E-39	postive
AURKA	AC080038.1	0.401877968	2.45E-22	postive
G6PD	AC080038.1	0.408567018	4.22E-23	postive
CDKN2A	AC080038.1	0.431887392	6.69E-26	postive
FANCD2	AP000873.3	0.402540459	2.06E-22	postive
HELLS	AP000873.3	0.440416847	5.56E-27	postive
ZNF419	AP000873.3	0.429064561	1.50E-25	postive
TUBE1	AP000873.3	0.429798394	1.22E-25	postive
ALOX12	AP000873.3	0.566577831	4.34E-47	postive
TFAP2C	AP000873.3	0.442063238	3.41E-27	postive
HBA1	AP000873.3	0.421087872	1.41E-24	postive
DUOX1	AP000873.3	0.41724588	4.06E-24	postive
FBXW7	AP000873.3	0.412792949	1.36E-23	postive
TAZ	AC136475.3	0.418768936	2.67E-24	postive
LINC00472	AC119424.1	0.560512931	6.45E-46	postive
ATM	AC119424.1	0.429165459	1.46E-25	postive
MUC1	AC104072.1	0.493046353	2.31E-34	postive
SLC2A1	AC104072.1	0.421582609	1.23E-24	postive
TAZ	TOLLIP-AS1	0.422907378	8.50E-25	postive
SLC3A2	LINC01802	0.429753939	1.23E-25	postive
FH	LINC01802	0.440398118	5.59E-27	postive
ISCU	LINC01802	0.575003399	9.27E-49	postive
SLC2A8	LINC01802	0.436108955	1.97E-26	postive
CS	LINC01802	0.445701008	1.15E-27	postive
ATG4D	LINC01802	0.562395792	2.81E-46	postive
GABARAPL1	LINC01802	0.632634276	1.33E-61	postive
LPIN1	LINC01802	0.558334704	1.68E-45	postive
IREB2	AC008555.1	0.403332582	1.67E-22	postive
SP1	AC008555.1	0.511656672	2.70E-37	postive
ZEB1	AC008555.1	0.430204999	1.08E-25	postive
PRKAA2	AC008555.1	0.415792088	6.04E-24	postive
EPAS1	AC008555.1	0.465865881	2.17E-30	postive
SIRT1	AC008555.1	0.552355451	2.24E-44	postive
AKR1C2	AL033397.1	0.634019834	6.05E-62	postive
AKR1C3	AL033397.1	0.468272292	9.97E-31	postive
NQO1	AL033397.1	0.59582928	4.21E-53	postive
TXNRD1	AL033397.1	0.461848707	7.82E-30	postive
SRXN1	AL033397.1	0.415461479	6.61E-24	postive
HELLS	AL353600.1	0.497279873	5.16E-35	postive
TUBE1	AL353600.1	0.414757521	8.00E-24	postive
ALOX12	AL353600.1	0.400333444	3.65E-22	postive
GABPB1	AL353600.1	0.447182635	7.36E-28	postive
ATM	AL353600.1	0.561580891	4.03E-46	postive
FBXW7	AL353600.1	0.459117656	1.85E-29	postive
TFAP2C	AC008050.1	0.444381518	1.71E-27	postive
HBA1	AC008050.1	0.430395599	1.03E-25	postive
GPX4	AC108673.3	0.411209776	2.08E-23	postive
HSPB1	AC108673.3	0.465394025	2.52E-30	postive
HSF1	AC108673.3	0.447563592	6.56E-28	postive
LAMP2	AC108673.3	-0.418103867	3.21E-24	negative
EIF2S1	AC108673.3	-0.421890359	1.13E-24	negative
MAPK14	AC108673.3	-0.40553153	9.41E-23	negative
EIF2AK4	AC108673.3	-0.409534677	3.26E-23	negative
IREB2	AC108673.3	-0.4443217	1.74E-27	negative
NOX1	AC108673.3	0.474789959	1.18E-31	postive
PIK3CA	AC108673.3	-0.422543204	9.40E-25	negative
NRAS	AC108673.3	-0.462515712	6.33E-30	negative

HRAS	AC108673.3	0.493638826	1.88E-34	postive
NCOA4	AC108673.3	-0.564284369	1.21E-46	negative
PHKG2	AC108673.3	0.64326115	2.82E-64	postive
BECN1	AC108673.3	-0.530478107	1.89E-40	negative
MAPK1	AC108673.3	-0.496921493	5.86E-35	negative
ANO6	AC108673.3	-0.405153325	1.04E-22	negative
TAZ	AC108673.3	0.557520522	2.40E-45	postive
MTDH	AC108673.3	-0.459027372	1.91E-29	negative
SIRT1	AC108673.3	-0.418983716	2.52E-24	negative
TUBE1	AC084782.3	0.495666701	9.16E-35	postive
ATM	AC084782.3	0.44811899	5.54E-28	postive
HELLS	AC118344.1	0.416673079	4.75E-24	postive
ZNF419	AC118344.1	0.535797045	2.23E-41	postive
KLHL24	AC118344.1	0.498600332	3.22E-35	postive
TUBE1	AC118344.1	0.502994486	6.59E-36	postive
SETD1B	AC118344.1	0.443262881	2.39E-27	postive
ALOX12	AC118344.1	0.574047894	1.44E-48	postive
IREB2	AC118344.1	0.470271353	5.20E-31	postive
GABPB1	AC118344.1	0.53917797	5.61E-42	postive
PIK3CA	AC118344.1	0.436277979	1.88E-26	postive
MAPK8	AC118344.1	0.488183572	1.26E-33	postive
LINC00472	AC118344.1	0.771936643	9.88E-108	postive
LPIN1	AC118344.1	0.4140606	9.66E-24	postive
ATM	AC118344.1	0.62675824	3.61E-60	postive
FBXW7	AC118344.1	0.463174949	5.13E-30	postive
HELLS	DCUN1D2-A	0.450584308	2.62E-28	postive
ZNF419	DCUN1D2-A	0.447180886	7.36E-28	postive
VEGFA	DCUN1D2-A	0.424158057	5.99E-25	postive
TUBE1	DCUN1D2-A	0.541225671	2.41E-42	postive
SETD1B	DCUN1D2-A	0.438850032	8.83E-27	postive
ALOX12	DCUN1D2-A	0.599943358	5.36E-54	postive
GABPB1	DCUN1D2-A	0.46839739	9.57E-31	postive
MAPK8	DCUN1D2-A	0.409791426	3.04E-23	postive
ATM	DCUN1D2-A	0.641175043	9.63E-64	postive
FBXW7	DCUN1D2-A	0.526852343	7.93E-40	postive
TFAP2C	AC120498.3	0.554463701	9.03E-45	postive
ISCU	AC106791.1	0.40334112	1.67E-22	postive
KLHL24	AC106791.1	0.504188406	4.27E-36	postive
MAP3K5	AC106791.1	0.431183075	8.18E-26	postive
IREB2	AC106791.1	0.434515429	3.13E-26	postive
GABPB1	AC106791.1	0.404843011	1.13E-22	postive
PIK3CA	AC106791.1	0.429709696	1.25E-25	postive
ATG7	AC106791.1	0.409936912	2.93E-23	postive
LINC00472	AC106791.1	0.650726139	3.22E-66	postive
LPIN1	AC106791.1	0.519492053	1.39E-38	postive
ATM	AC106791.1	0.507431638	1.30E-36	postive
HELLS	AC073534.2	0.540206307	3.68E-42	postive
ALOX12	AC073534.2	0.403052942	1.80E-22	postive
GABPB1	AC073534.2	0.477859068	4.24E-32	postive
LINC00472	AC073534.2	0.559386899	1.06E-45	postive
ATM	AC073534.2	0.504803626	3.41E-36	postive
FBXW7	AC073534.2	0.445395863	1.26E-27	postive
BNIP3	LINC02188	0.424535883	5.39E-25	postive
HNF4A	LINC02188	0.45724467	3.34E-29	postive
ALOX12	AL357079.1	0.421478695	1.26E-24	postive
TAZ	AL357079.1	0.413352597	1.17E-23	postive
CYBB	LINC01150	0.475236242	1.02E-31	postive
HELLS	AP000350.6	0.457079717	3.51E-29	postive

TUBE1	AP000350.6	0.532791377	7.48E-41	postive
ALOX12	AP000350.6	0.544056265	7.46E-43	postive
IREB2	AP000350.6	0.433268831	4.49E-26	postive
GABPB1	AP000350.6	0.493502509	1.97E-34	postive
MAPK8	AP000350.6	0.451340147	2.08E-28	postive
LINC00472	AP000350.6	0.614781586	2.44E-57	postive
ATM	AP000350.6	0.707199396	6.75E-83	postive
FBXW7	AP000350.6	0.439001712	8.45E-27	postive
HELLS	AL450998.2	0.581525582	4.37E-50	postive
ZNF419	AL450998.2	0.517475989	3.00E-38	postive
TUBE1	AL450998.2	0.564790642	9.66E-47	postive
SETD1B	AL450998.2	0.406162478	7.97E-23	postive
ALOX12	AL450998.2	0.716729961	4.11E-86	postive
GABPB1	AL450998.2	0.533336688	6.01E-41	postive
LINC00472	AL450998.2	0.481996812	1.05E-32	postive
ATM	AL450998.2	0.694661856	7.36E-79	postive
FBXW7	AL450998.2	0.546980823	2.19E-43	postive
ZNF419	HCG27	0.485175664	3.56E-33	postive
VEGFA	HCG27	0.52153829	6.30E-39	postive
TUBE1	HCG27	0.672922589	2.48E-72	postive
ALOX12	HCG27	0.705572309	2.32E-82	postive
ATM	HCG27	0.462848213	5.69E-30	postive
TAZ	HCG27	0.58750266	2.50E-51	postive
HELLS	SCAANT1	0.492190575	3.12E-34	postive
TUBE1	SCAANT1	0.404998504	1.08E-22	postive
ALOX12	SCAANT1	0.416797056	4.59E-24	postive
GABPB1	SCAANT1	0.449069936	4.15E-28	postive
LINC00472	SCAANT1	0.505214377	2.93E-36	postive
ATM	SCAANT1	0.503063091	6.43E-36	postive
FBXW7	SCAANT1	0.406524548	7.24E-23	postive
TFAP2C	AC012485.2	0.477197584	5.29E-32	postive
HBA1	AC012485.2	0.436517838	1.75E-26	postive
HBA1	LINC02723	0.432934966	4.94E-26	postive
HELLS	LAMC1-AS1	0.469565427	6.55E-31	postive
ZNF419	LAMC1-AS1	0.468103353	1.05E-30	postive
VEGFA	LAMC1-AS1	0.435653879	2.25E-26	postive
TUBE1	LAMC1-AS1	0.566705708	4.09E-47	postive
SETD1B	LAMC1-AS1	0.423508949	7.18E-25	postive
ALOX12	LAMC1-AS1	0.573901953	1.54E-48	postive
GABPB1	LAMC1-AS1	0.495696045	9.07E-35	postive
MAPK8	LAMC1-AS1	0.407799989	5.17E-23	postive
ATM	LAMC1-AS1	0.616437062	1.01E-57	postive
FBXW7	LAMC1-AS1	0.523396086	3.06E-39	postive
VEGFA	AC136475.2	0.548721438	1.05E-43	postive
ALOX12	AC136475.2	0.505077045	3.08E-36	postive
TAZ	AC136475.2	0.552939175	1.74E-44	postive
VEGFA	AC064875.1	0.412256235	1.57E-23	postive
ZEB1	AC064875.1	0.536393819	1.75E-41	postive
MAPK8	AC064875.1	0.415850227	5.94E-24	postive
EPAS1	AC064875.1	0.481404125	1.29E-32	postive
ZNF419	AL355297.2	0.435614324	2.28E-26	postive
TUBE1	AL355297.2	0.533626402	5.35E-41	postive
ALOX12	AL355297.2	0.465929042	2.12E-30	postive
GABPB1	AL355297.2	0.53381627	4.96E-41	postive
LINC00472	AL355297.2	0.677542412	1.13E-73	postive
ATM	AL355297.2	0.492225168	3.09E-34	postive
HELLS	AC079760.1	0.446518658	8.99E-28	postive
TUBE1	AC079760.1	0.422500523	9.52E-25	postive

ALOX12	AC079760.1	0.440121623	6.07E-27	postive
GABPB1	AC079760.1	0.404978889	1.09E-22	postive
ATM	AC079760.1	0.664258148	6.98E-70	postive
HELLS	SCAT2	0.436175452	1.93E-26	postive
ZNF419	SCAT2	0.46580569	2.21E-30	postive
TUBE1	SCAT2	0.464448312	3.42E-30	postive
ALOX12	SCAT2	0.421126902	1.39E-24	postive
PHKG2	SCAT2	0.615387764	1.77E-57	postive
TAZ	SCAT2	0.66274474	1.83E-69	postive
RB1	TNFRSF10A-.	0.581542907	4.34E-50	postive
IREB2	TNFRSF10A-.	0.427870555	2.10E-25	postive
HMGB1	TNFRSF10A-.	0.4074624	5.65E-23	postive
SP1	TNFRSF10A-.	0.413473248	1.13E-23	postive
EMC2	TNFRSF10A-.	0.411089828	2.15E-23	postive
NRAS	TNFRSF10A-.	0.539436859	5.04E-42	postive
KRAS	TNFRSF10A-.	0.406249701	7.79E-23	postive
MAPK8	TNFRSF10A-.	0.487810313	1.44E-33	postive
PRKAA2	TNFRSF10A-.	0.497702863	4.44E-35	postive
PRKAA1	TNFRSF10A-.	0.478859291	3.03E-32	postive
ATM	TNFRSF10A-.	0.557272661	2.67E-45	postive
SIRT1	TNFRSF10A-.	0.540428139	3.36E-42	postive
BACH1	TNFRSF10A-.	0.408286188	4.54E-23	postive
HELLS	MED8-AS1	0.409938337	2.93E-23	postive
BRD4	MED8-AS1	0.415845195	5.95E-24	postive
ZNF419	MED8-AS1	0.535140177	2.90E-41	postive
TUBE1	MED8-AS1	0.485200377	3.53E-33	postive
SETD1B	MED8-AS1	0.434115059	3.52E-26	postive
ALOX12	MED8-AS1	0.490576824	5.49E-34	postive
PHKG2	MED8-AS1	0.453714172	1.00E-28	postive
ATM	MED8-AS1	0.417204033	4.11E-24	postive
TAZ	MED8-AS1	0.530792771	1.66E-40	postive
FBXW7	MED8-AS1	0.429055587	1.50E-25	postive
TFAP2C	AC126773.3	0.585704381	5.96E-51	postive
HBA1	AC126773.3	0.555579524	5.57E-45	postive
DUOX1	AC126773.3	0.402709608	1.97E-22	postive
ZNF419	LINC02062	0.485787913	2.88E-33	postive
TUBE1	LINC02062	0.454975558	6.76E-29	postive
ALOX12	LINC02062	0.494021353	1.64E-34	postive
PHKG2	LINC02062	0.512474909	1.99E-37	postive
TAZ	LINC02062	0.665104826	4.05E-70	postive
HELLS	AL049539.1	0.525957246	1.13E-39	postive
ALOX12	AL049539.1	0.438053848	1.12E-26	postive
TFAP2C	AL049539.1	0.4239679	6.32E-25	postive
GABPB1	AL049539.1	0.430046564	1.13E-25	postive
DUOX1	AL049539.1	0.426471596	3.13E-25	postive
LINC00472	AL049539.1	0.514300365	1.00E-37	postive
ATM	AL049539.1	0.437769891	1.21E-26	postive
ZNF419	AL161935.1	0.427425141	2.39E-25	postive
TUBE1	AL161935.1	0.444032591	1.90E-27	postive
ALOX12	AL161935.1	0.580258298	7.96E-50	postive
TAZ	AL161935.1	0.492713983	2.60E-34	postive
LINC00472	AL358216.1	0.415897319	5.87E-24	postive
ATM	AL358216.1	0.52178833	5.72E-39	postive
ACSL3	NCOA7-AS1	0.425949262	3.62E-25	postive
KLHL24	NCOA7-AS1	0.69856268	4.30E-80	postive
IREB2	NCOA7-AS1	0.427758674	2.17E-25	postive
PIK3CA	NCOA7-AS1	0.500190389	1.82E-35	postive
KRAS	NCOA7-AS1	0.453881028	9.49E-29	postive

GABARAPL1	NCOA7-AS1	0.467398863	1.32E-30	postive
LINC00472	NCOA7-AS1	0.901011195	6.37E-197	postive
PRKAA1	NCOA7-AS1	0.406424116	7.44E-23	postive
LPIN1	NCOA7-AS1	0.58792924	2.04E-51	postive
HELLS	AL442128.2	0.453070559	1.22E-28	postive
ZNF419	AL442128.2	0.556001084	4.64E-45	postive
TUBE1	AL442128.2	0.603438973	9.07E-55	postive
ALOX12	AL442128.2	0.564386091	1.16E-46	postive
GABPB1	AL442128.2	0.547681698	1.63E-43	postive
PHKG2	AL442128.2	0.47665932	6.33E-32	postive
LINC00472	AL442128.2	0.522071888	5.12E-39	postive
ATM	AL442128.2	0.512529174	1.95E-37	postive
TAZ	AL442128.2	0.552573559	2.04E-44	postive
FBXW7	AL442128.2	0.454946465	6.82E-29	postive
PHKG2	AC110772.2	0.430150669	1.10E-25	postive
MTOR	AC024075.3	0.457366	3.21E-29	postive
ZNF419	AC024075.3	0.512305452	2.12E-37	postive
KLHL24	AC024075.3	0.542350445	1.52E-42	postive
TUBE1	AC024075.3	0.469223132	7.32E-31	postive
SETD1B	AC024075.3	0.432594054	5.46E-26	postive
ALOX12	AC024075.3	0.457337112	3.24E-29	postive
IREB2	AC024075.3	0.522192051	4.89E-39	postive
GABPB1	AC024075.3	0.502117167	9.06E-36	postive
PIK3CA	AC024075.3	0.441968237	3.51E-27	postive
MAPK8	AC024075.3	0.462618563	6.13E-30	postive
LINC00472	AC024075.3	0.627901012	1.91E-60	postive
LPIN1	AC024075.3	0.47024513	5.25E-31	postive
ATM	AC024075.3	0.645177271	9.06E-65	postive
FBXW7	AC024075.3	0.497465024	4.83E-35	postive
FANCD2	AL683807.1	0.42605114	3.52E-25	postive
ALOX12	AL683807.1	0.430130109	1.11E-25	postive
IFNG	AL683807.1	0.405645583	9.13E-23	postive
TAZ	AL683807.1	0.455663612	5.46E-29	postive
FBXW7	AL683807.1	0.400895784	3.15E-22	postive
TFAP2C	AL589739.1	0.581116168	5.31E-50	postive
HBA1	AL589739.1	0.553360692	1.45E-44	postive
ZNF419	AL031709.1	0.573518041	1.84E-48	postive
VEGFA	AL031709.1	0.521964887	5.34E-39	postive
TUBE1	AL031709.1	0.607739638	9.89E-56	postive
SETD1B	AL031709.1	0.512944164	1.67E-37	postive
ALOX12	AL031709.1	0.742150058	2.23E-95	postive
GABPB1	AL031709.1	0.405076361	1.06E-22	postive
ATM	AL031709.1	0.504795723	3.42E-36	postive
TAZ	AL031709.1	0.454559617	7.69E-29	postive
TUBE1	LINC00240	0.414364442	8.90E-24	postive
PHKG2	AL359881.2	0.540784512	2.90E-42	postive
TAZ	AL359881.2	0.41495448	7.58E-24	postive
LINC00472	MCPH1-AS1	0.407623724	5.42E-23	postive
ATM	MCPH1-AS1	0.460627027	1.15E-29	postive
HELLS	AC022306.3	0.445330075	1.29E-27	postive
TUBE1	AC022306.3	0.426440479	3.15E-25	postive
NOX1	AC022306.3	0.462354871	6.66E-30	postive
HELLS	RNF216P1	0.472730034	2.32E-31	postive
ASNS	RNF216P1	0.437178775	1.44E-26	postive
ALOX12	AC005479.1	0.415273758	6.95E-24	postive
ATM	AC005479.1	0.531626533	1.19E-40	postive
LAMP2	LINC01705	0.500265693	1.77E-35	postive
PHKG2	AL139286.1	0.493566132	1.93E-34	postive

TAZ	AL139286.1	0.441292173	4.29E-27	postive
TFAP2C	AC022960.1	0.575814509	6.36E-49	postive
HBA1	AC022960.1	0.520839625	8.25E-39	postive
DUOX1	AC022960.1	0.421739256	1.18E-24	postive
CA9	SNHG18	0.407144033	6.15E-23	postive
SLC2A6	LINC01871	0.469048293	7.75E-31	postive
BID	LINC01871	0.41778749	3.50E-24	postive
IFNG	LINC01871	0.546531538	2.65E-43	postive
BRD4	AL031186.1	0.419375752	2.26E-24	postive
ZNF419	AL031186.1	0.544025349	7.56E-43	postive
TUBE1	AL031186.1	0.42805361	2.00E-25	postive
SETD1B	AL031186.1	0.500833695	1.44E-35	postive
ALOX12	AL031186.1	0.490027889	6.65E-34	postive
PHKG2	AL031186.1	0.525152867	1.54E-39	postive
TAZ	AL031186.1	0.638285963	5.19E-63	postive
ACSL3	AC112220.2	0.458548749	2.22E-29	postive
OXSR1	AC112220.2	0.535119291	2.93E-41	postive
KLHL24	AC112220.2	0.608131538	8.07E-56	postive
IREB2	AC112220.2	0.498377694	3.49E-35	postive
PIK3CA	AC112220.2	0.501447448	1.15E-35	postive
KRAS	AC112220.2	0.459410919	1.69E-29	postive
GABARAPL1	AC112220.2	0.527223612	6.85E-40	postive
MAPK8	AC112220.2	0.40270306	1.97E-22	postive
LINC00472	AC112220.2	0.491037986	4.68E-34	postive
PRKAA1	AC112220.2	0.460938909	1.04E-29	postive
BAP1	AC112220.2	0.442683569	2.84E-27	postive
LPIN1	AC112220.2	0.484740186	4.13E-33	postive
HELLS	AL138921.1	0.455137747	6.43E-29	postive
ZNF419	AL138921.1	0.522909312	3.70E-39	postive
TUBE1	AL138921.1	0.608976297	5.20E-56	postive
SETD1B	AL138921.1	0.407980564	4.93E-23	postive
ALOX12	AL138921.1	0.638526612	4.52E-63	postive
GABPB1	AL138921.1	0.476291032	7.15E-32	postive
LINC00472	AL138921.1	0.428837653	1.60E-25	postive
ATM	AL138921.1	0.533350768	5.98E-41	postive
TAZ	AL138921.1	0.498958563	2.83E-35	postive
FBXW7	AL138921.1	0.498299449	3.58E-35	postive
HELLS	AC090425.3	0.419196244	2.38E-24	postive
TUBE1	AC090425.3	0.416526964	4.94E-24	postive
GABPB1	AC090425.3	0.409679957	3.14E-23	postive
ATM	AC090425.3	0.463318299	4.90E-30	postive
ALOX12	SUV39H2-DT	0.407496402	5.60E-23	postive
PHKG2	SUV39H2-DT	0.407408706	5.73E-23	postive
TAZ	SUV39H2-DT	0.538553877	7.24E-42	postive
FANCD2	LINC00460	0.441582756	3.94E-27	postive
STMN1	LINC00460	0.520268565	1.03E-38	postive
RRM2	LINC00460	0.572292088	3.23E-48	postive
AURKA	LINC00460	0.542306356	1.54E-42	postive
CDKN2A	LINC00460	0.52895831	3.45E-40	postive
PANX1	LINC00460	0.42488681	4.88E-25	postive
TFAP2C	AC100847.1	0.556312835	4.05E-45	postive
HBA1	AC100847.1	0.505602445	2.54E-36	postive
ISCU	SBF2-AS1	0.566836912	3.86E-47	postive
DDIT3	SBF2-AS1	0.503911122	4.72E-36	postive
KLHL24	SBF2-AS1	0.413093739	1.25E-23	postive
ATG4D	SBF2-AS1	0.402861297	1.89E-22	postive
GABARAPL1	SBF2-AS1	0.540235087	3.63E-42	postive
ALOX12	ADORA2A-A	0.437285862	1.40E-26	postive

GPX4	AL023284.4	0.408791817	3.97E-23	postive
HRAS	AL023284.4	0.414971301	7.55E-24	postive
PEBP1	AL023284.4	0.505909061	2.27E-36	postive
MTDH	AL023284.4	-0.425855417	3.72E-25	negative
BACH1	AL023284.4	-0.425314895	4.33E-25	negative
OTUB1	URB1-AS1	0.459486938	1.65E-29	postive
CHMP6	URB1-AS1	0.420954622	1.46E-24	postive
FANCD2	AC003102.1	0.408784526	3.98E-23	postive
BRD4	AC003102.1	0.444580844	1.61E-27	postive
ZNF419	AC003102.1	0.552998101	1.70E-44	postive
TUBE1	AC003102.1	0.433386234	4.34E-26	postive
DRD4	AC003102.1	0.41266403	1.41E-23	postive
ALOX12	AC003102.1	0.541522059	2.14E-42	postive
PHKG2	AC003102.1	0.55771723	2.20E-45	postive
TAZ	AC003102.1	0.628072481	1.74E-60	postive
SLC3A2	AC021087.4	0.419662465	2.09E-24	postive
ISCU	AC021087.4	0.473890644	1.59E-31	postive
CHAC1	AC021087.4	0.420029462	1.89E-24	postive
DDIT3	AC021087.4	0.551119913	3.79E-44	postive
SLC2A12	AC021087.4	0.413610362	1.09E-23	postive
ATG4D	AC021087.4	0.503997261	4.57E-36	postive
MAP1LC3A	AC021087.4	0.546400094	2.80E-43	postive
GABARAPL1	AC021087.4	0.63821698	5.40E-63	postive
WIPI2	AC021087.4	0.502550126	7.74E-36	postive
LPIN1	AC021087.4	0.462875989	5.64E-30	postive
HELLS	AC002064.2	0.579303838	1.25E-49	postive
TUBE1	AC002064.2	0.477897455	4.18E-32	postive
ALOX12	AC002064.2	0.57398359	1.49E-48	postive
GABPB1	AC002064.2	0.462964206	5.49E-30	postive
DUOX1	AC002064.2	0.42504409	4.67E-25	postive
ATM	AC002064.2	0.611335472	1.51E-56	postive
FBXW7	AC002064.2	0.476157866	7.48E-32	postive
HELLS	AC008731.1	0.460761674	1.10E-29	postive
ZNF419	AC008731.1	0.407250838	5.98E-23	postive
ALOX12	AC008731.1	0.491048852	4.66E-34	postive
TFAP2C	AC008731.1	0.443549539	2.19E-27	postive
GABPB1	AC008731.1	0.41911325	2.43E-24	postive
DUOX1	AC008731.1	0.435170039	2.59E-26	postive
LINC00472	AC008731.1	0.565634644	6.62E-47	postive
ATM	AC008731.1	0.409469722	3.32E-23	postive
FBXW7	AC008731.1	0.404801192	1.14E-22	postive
ATM	AC026412.3	0.433350703	4.39E-26	postive
TAZ	AC026412.3	0.409835584	3.01E-23	postive
KLHL24	ZRANB2-AS1	0.415744002	6.12E-24	postive
TUBE1	ZRANB2-AS1	0.413100268	1.25E-23	postive
IREB2	ZRANB2-AS1	0.409980569	2.89E-23	postive
GABPB1	ZRANB2-AS1	0.409948984	2.92E-23	postive
MAPK8	ZRANB2-AS1	0.404536986	1.22E-22	postive
LINC00472	ZRANB2-AS1	0.606091081	2.32E-55	postive
LPIN1	ZRANB2-AS1	0.406798791	6.74E-23	postive
ATM	ZRANB2-AS1	0.593463239	1.36E-52	postive
PHKG2	LINC01341	0.450135265	3.00E-28	postive
TAZ	LINC01341	0.540414751	3.37E-42	postive
FANCD2	KLRK1-AS1	0.583831792	1.46E-50	postive
HELLS	KLRK1-AS1	0.576306887	5.06E-49	postive
HELLS	AC018809.2	0.469136133	7.53E-31	postive
ZNF419	AC018809.2	0.455939446	5.01E-29	postive
TUBE1	AC018809.2	0.487910185	1.39E-33	postive

ALOX12	AC018809.2	0.573014666	2.32E-48 postive
GABPB1	AC018809.2	0.505421217	2.72E-36 postive
MAPK8	AC018809.2	0.437948015	1.15E-26 postive
LINC00472	AC018809.2	0.494663888	1.31E-34 postive
ATM	AC018809.2	0.660893547	5.92E-69 postive
FBXW7	AC018809.2	0.445376566	1.27E-27 postive
TUBE1	AC135050.6	0.412472452	1.48E-23 postive
PHKG2	AC135050.6	0.572355694	3.14E-48 postive
ATG4D	AC135050.6	0.410341092	2.63E-23 postive
TAZ	AC135050.6	0.507474904	1.28E-36 postive
CD44	LINC01605	0.401740868	2.53E-22 postive
SLC1A5	LINC01605	0.425131932	4.56E-25 postive
CDKN2A	LINC01605	0.423260018	7.70E-25 postive
HELLS	AL031673.1	0.434050473	3.58E-26 postive
TUBE1	AL031673.1	0.512106062	2.28E-37 postive
ALOX12	AL031673.1	0.4870257	1.88E-33 postive
LINC00472	AL031673.1	0.492320272	2.98E-34 postive
ATM	AL031673.1	0.543484222	9.47E-43 postive
HELLS	AL132989.1	0.452573408	1.42E-28 postive
ZNF419	AL132989.1	0.565199446	8.05E-47 postive
VEGFA	AL132989.1	0.480491899	1.75E-32 postive
TUBE1	AL132989.1	0.654683238	2.86E-67 postive
SETD1B	AL132989.1	0.529526991	2.75E-40 postive
ALOX12	AL132989.1	0.708274524	2.97E-83 postive
GABPB1	AL132989.1	0.548099312	1.37E-43 postive
MAPK8	AL132989.1	0.478980168	2.91E-32 postive
LINC00472	AL132989.1	0.537106566	1.31E-41 postive
ATM	AL132989.1	0.682038489	5.33E-75 postive
YY1AP1	AL132989.1	0.417666349	3.62E-24 postive
FBXW7	AL132989.1	0.524749948	1.81E-39 postive
HELLS	OSMR-AS1	0.582961173	2.21E-50 postive
TUBE1	OSMR-AS1	0.461276827	9.38E-30 postive
ALOX12	OSMR-AS1	0.543848333	8.14E-43 postive
GABPB1	OSMR-AS1	0.514861746	8.09E-38 postive
LINC00472	OSMR-AS1	0.468851116	8.26E-31 postive
ATM	OSMR-AS1	0.674246255	1.03E-72 postive
FBXW7	OSMR-AS1	0.503349296	5.79E-36 postive
TFAP2C	AC119396.1	0.585762978	5.79E-51 postive
HBA1	AC119396.1	0.526129588	1.05E-39 postive
DUOX1	AC119396.1	0.409605584	3.20E-23 postive
ISCU	AP000894.4	0.554539092	8.74E-45 postive
GOT1	AP000894.4	0.446513264	9.01E-28 postive
ATG4D	AP000894.4	0.572418464	3.05E-48 postive
GABARAPL2	AP000894.4	0.522204937	4.87E-39 postive
GABARAPL1	AP000894.4	0.418082654	3.23E-24 postive
ISCU	AC027682.6	0.495322874	1.03E-34 postive
ATG4D	AC027682.6	0.47278177	2.29E-31 postive
MAP1LC3A	AC027682.6	0.487483151	1.61E-33 postive
GABARAPL2	AC027682.6	0.449181857	4.01E-28 postive
HELLS	AC004466.3	0.426214082	3.36E-25 postive
ZNF419	AC004466.3	0.466886866	1.56E-30 postive
TUBE1	AC004466.3	0.484230568	4.92E-33 postive
ALOX12	AC004466.3	0.474312842	1.38E-31 postive
GABPB1	AC004466.3	0.575420493	7.64E-49 postive
MAPK8	AC004466.3	0.401562236	2.65E-22 postive
LINC00472	AC004466.3	0.728822579	2.18E-90 postive
ATM	AC004466.3	0.608286984	7.44E-56 postive
FBXW7	AC004466.3	0.481660894	1.18E-32 postive

HBA1	AC016735.1	0.440471528	5.47E-27	postive
HELLS	MIR34AHG	0.40695598	6.46E-23	postive
ALOX12	MIR34AHG	0.500709038	1.51E-35	postive
ATM	MIR34AHG	0.605648259	2.92E-55	postive
FBXW7	MIR34AHG	0.438082177	1.11E-26	postive
HELLS	AC021851.1	0.548964915	9.48E-44	postive
ZNF419	AC021851.1	0.447599827	6.49E-28	postive
TUBE1	AC021851.1	0.635951652	2.00E-62	postive
ALOX12	AC021851.1	0.529564882	2.71E-40	postive
GABPB1	AC021851.1	0.555570371	5.59E-45	postive
ATM	AC021851.1	0.640526028	1.41E-63	postive
TAZ	AC021851.1	0.419694921	2.07E-24	postive
FBXW7	AC021851.1	0.493197973	2.19E-34	postive
HELLS	AC068790.3	0.525084348	1.59E-39	postive
TUBE1	AC068790.3	0.490488037	5.67E-34	postive
ALOX12	AC068790.3	0.461155207	9.75E-30	postive
GABPB1	AC068790.3	0.574188292	1.35E-48	postive
MAPK8	AC068790.3	0.423323031	7.57E-25	postive
LINC00472	AC068790.3	0.745417364	1.20E-96	postive
ATM	AC068790.3	0.739918162	1.61E-94	postive
FBXW7	AC068790.3	0.495358441	1.02E-34	postive
TFAP2C	LINC00551	0.532785702	7.50E-41	postive
HBA1	LINC00551	0.489356526	8.40E-34	postive
CD44	CD44-AS1	0.61587292	1.36E-57	postive
SOCS1	CD44-AS1	0.624089069	1.58E-59	postive
HRAS	CDC37L1-DT	0.598314551	1.22E-53	postive
EGLN2	CDC37L1-DT	0.59409616	9.96E-53	postive
HELLS	AC108010.1	0.468226882	1.01E-30	postive
MTOR	AC108010.1	0.428319232	1.85E-25	postive
ZNF419	AC108010.1	0.454952497	6.81E-29	postive
VEGFA	AC108010.1	0.414296263	9.06E-24	postive
TUBE1	AC108010.1	0.517358754	3.13E-38	postive
SETD1B	AC108010.1	0.48424276	4.89E-33	postive
ALOX12	AC108010.1	0.637915765	6.43E-63	postive
IREB2	AC108010.1	0.481414402	1.28E-32	postive
SP1	AC108010.1	0.44629383	9.62E-28	postive
GABPB1	AC108010.1	0.497658905	4.51E-35	postive
ZEB1	AC108010.1	0.40029981	3.68E-22	postive
MAPK8	AC108010.1	0.495530346	9.62E-35	postive
LINC00472	AC108010.1	0.530882313	1.61E-40	postive
ATM	AC108010.1	0.775369237	2.82E-109	postive
FBXW7	AC108010.1	0.517829632	2.62E-38	postive
GPX4	SNHG9	0.479320606	2.60E-32	postive
RB1	SNHG9	-0.414165127	9.39E-24	negative
HSPB1	SNHG9	0.64422756	1.59E-64	postive
OXSR1	SNHG9	-0.409839109	3.00E-23	negative
MAPK14	SNHG9	-0.437086453	1.48E-26	negative
SLC2A6	SNHG9	0.409461775	3.32E-23	postive
IREB2	SNHG9	-0.458373231	2.34E-29	negative
HBA1	SNHG9	0.454699126	7.37E-29	postive
NOX1	SNHG9	0.423379914	7.45E-25	postive
PIK3CA	SNHG9	-0.42976685	1.23E-25	negative
NRAS	SNHG9	-0.41303643	1.27E-23	negative
KRAS	SNHG9	-0.410249396	2.69E-23	negative
HRAS	SNHG9	0.7057182	2.08E-82	postive
NCOA4	SNHG9	-0.532168761	9.60E-41	negative
PHKG2	SNHG9	0.602595056	1.40E-54	postive
BECN1	SNHG9	-0.500333901	1.73E-35	negative

MAP1LC3A	SNHG9	0.41620976	5.39E-24	postive
MAPK1	SNHG9	-0.466203141	1.94E-30	negative
PRKAA1	SNHG9	-0.426044899	3.53E-25	negative
ANO6	SNHG9	-0.403379985	1.65E-22	negative
EGLN2	SNHG9	0.56852535	1.80E-47	postive
TAZ	SNHG9	0.501107519	1.31E-35	postive
SIRT1	SNHG9	-0.451707426	1.85E-28	negative
HELLS	AC007619.1	0.480166905	1.95E-32	postive
ALOX12	AC007619.1	0.441941256	3.54E-27	postive
TFAP2C	AC007619.1	0.477232224	5.23E-32	postive
HBA1	AC007619.1	0.420512154	1.65E-24	postive
DUOX1	AC007619.1	0.435709513	2.21E-26	postive
LINC00472	AC007619.1	0.455748268	5.32E-29	postive
FH	MCF2L-AS1	0.419070737	2.46E-24	postive
ISCU	MCF2L-AS1	0.589627756	8.93E-52	postive
DDIT3	MCF2L-AS1	0.428553092	1.73E-25	postive
GPT2	MCF2L-AS1	0.407107496	6.21E-23	postive
KLHL24	MCF2L-AS1	0.412609575	1.43E-23	postive
SLC2A8	MCF2L-AS1	0.419484765	2.19E-24	postive
SLC2A12	MCF2L-AS1	0.466402527	1.82E-30	postive
CS	MCF2L-AS1	0.401787418	2.50E-22	postive
ATG4D	MCF2L-AS1	0.611999605	1.07E-56	postive
MAP1LC3A	MCF2L-AS1	0.448980836	4.27E-28	postive
GABARAPL1	MCF2L-AS1	0.722564967	3.79E-88	postive
WIPI2	MCF2L-AS1	0.501028245	1.34E-35	postive
LPIN1	MCF2L-AS1	0.633221264	9.53E-62	postive
HELLS	LINC01239	0.440980274	4.71E-27	postive
ALOX12	LINC01239	0.429829606	1.21E-25	postive
ATM	LINC01239	0.485012416	3.76E-33	postive
IREB2	AC064807.1	0.404167498	1.35E-22	postive
HELLS	AC004000.1	0.459453941	1.67E-29	postive
ALOX12	AC004000.1	0.417968143	3.33E-24	postive
TFAP2C	AC004000.1	0.471694652	3.27E-31	postive
HBA1	AC004000.1	0.44664731	8.65E-28	postive
DUOX1	AC004000.1	0.411050878	2.17E-23	postive
ACSL3	AL353699.1	0.406722103	6.87E-23	postive
KLHL24	AL353699.1	0.651001412	2.73E-66	postive
PIK3CA	AL353699.1	0.457548759	3.03E-29	postive
KRAS	AL353699.1	0.406339826	7.60E-23	postive
GABARAPL1	AL353699.1	0.462634398	6.10E-30	postive
LINC00472	AL353699.1	0.798605939	1.66E-120	postive
LPIN1	AL353699.1	0.529457951	2.83E-40	postive
HELLS	AL513327.1	0.55715157	2.81E-45	postive
ZNF419	AL513327.1	0.454952127	6.81E-29	postive
TUBE1	AL513327.1	0.545924918	3.42E-43	postive
ALOX12	AL513327.1	0.610689938	2.12E-56	postive
GABPB1	AL513327.1	0.559479958	1.02E-45	postive
LINC00472	AL513327.1	0.537722325	1.02E-41	postive
ATM	AL513327.1	0.721972803	6.14E-88	postive
FBXW7	AL513327.1	0.529697097	2.57E-40	postive
HELLS	AC084357.2	0.46489867	2.96E-30	postive
ALOX12	AC084357.2	0.440418106	5.56E-27	postive
TFAP2C	AC084357.2	0.441037477	4.63E-27	postive
HSPB1	NUP153-AS1	0.484260381	4.87E-33	postive
SLC2A6	NUP153-AS1	0.496558073	6.67E-35	postive
IREB2	NUP153-AS1	-0.413592823	1.10E-23	negative
HRAS	NUP153-AS1	0.444218599	1.80E-27	postive
SLC1A5	NUP153-AS1	0.413278166	1.19E-23	postive

NCOA4	NUP153-AS1	-0.488395797	1.17E-33	negative
PHKG2	NUP153-AS1	0.500660027	1.53E-35	postive
MAPK1	NUP153-AS1	-0.421410866	1.29E-24	negative
BID	NUP153-AS1	0.476730333	6.18E-32	postive
MAPK9	NUP153-AS1	-0.402847163	1.90E-22	negative
PRKAA2	NUP153-AS1	-0.407879878	5.06E-23	negative
TAZ	NUP153-AS1	0.464814512	3.04E-30	postive
HELLS	SUGT1P4-ST	0.473989686	1.53E-31	postive
ZNF419	SUGT1P4-ST	0.575343641	7.92E-49	postive
TUBE1	SUGT1P4-ST	0.609752069	3.47E-56	postive
ALOX12	SUGT1P4-ST	0.560546147	6.36E-46	postive
GABPB1	SUGT1P4-ST	0.54455144	6.07E-43	postive
MAPK8	SUGT1P4-ST	0.411751037	1.80E-23	postive
LINC00472	SUGT1P4-ST	0.566440905	4.61E-47	postive
ATM	SUGT1P4-ST	0.648781831	1.05E-65	postive
FBXW7	SUGT1P4-ST	0.478649981	3.25E-32	postive
HELLS	AL162430.2	0.515361621	6.69E-38	postive
ALOX12	AL162430.2	0.466196767	1.95E-30	postive
TFAP2C	AL162430.2	0.42560957	3.99E-25	postive
GABPB1	AL162430.2	0.426920654	2.75E-25	postive
DUOX1	AL162430.2	0.439917469	6.45E-27	postive
LINC00472	AL162430.2	0.529092193	3.27E-40	postive
ATM	AL162430.2	0.473106156	2.05E-31	postive
FBXW7	AL162430.2	0.405661689	9.09E-23	postive
BRD4	CAPN10-DT	0.427447886	2.37E-25	postive
ZNF419	CAPN10-DT	0.574351346	1.25E-48	postive
TUBE1	CAPN10-DT	0.552642991	1.98E-44	postive
SETD1B	CAPN10-DT	0.439385149	7.54E-27	postive
ALOX12	CAPN10-DT	0.577897351	2.41E-49	postive
PHKG2	CAPN10-DT	0.579782067	9.96E-50	postive
ULK1	CAPN10-DT	0.427968663	2.05E-25	postive
TAZ	CAPN10-DT	0.672857561	2.59E-72	postive
HELLS	AC114316.1	0.503050131	6.46E-36	postive
TUBE1	AC114316.1	0.421683192	1.19E-24	postive
GABPB1	AC114316.1	0.446593079	8.79E-28	postive
LINC00472	AC114316.1	0.421923662	1.12E-24	postive
ATM	AC114316.1	0.656747207	7.96E-68	postive
ZNF419	AC005740.4	0.483272842	6.81E-33	postive
VEGFA	AC005740.4	0.516817189	3.85E-38	postive
TUBE1	AC005740.4	0.504231858	4.20E-36	postive
SETD1B	AC005740.4	0.44172467	3.78E-27	postive
ALOX12	AC005740.4	0.487766301	1.46E-33	postive
GABPB1	AC005740.4	0.491471082	4.02E-34	postive
PRDX1	AC005740.4	-0.40261118	2.02E-22	negative
ZEB1	AC005740.4	0.549632597	7.14E-44	postive
MAPK8	AC005740.4	0.402884405	1.88E-22	postive
ATM	AC005740.4	0.424639477	5.23E-25	postive
FBXW7	AC005740.4	0.461861407	7.79E-30	postive
PCK2	AC016888.1	0.432456798	5.68E-26	postive
BRD4	AC040162.3	0.419891669	1.96E-24	postive
ZNF419	AC040162.3	0.461166194	9.71E-30	postive
VEGFA	AC040162.3	0.527836269	5.38E-40	postive
TUBE1	AC040162.3	0.485757305	2.91E-33	postive
SETD1B	AC040162.3	0.455105706	6.50E-29	postive
DRD4	AC040162.3	0.471229935	3.80E-31	postive
ALOX12	AC040162.3	0.520795635	8.39E-39	postive
NCOA4	AC040162.3	-0.442806809	2.74E-27	negative
PHKG2	AC040162.3	0.645252913	8.66E-65	postive

TAZ	AC040162.3	0.775594819	2.23E-109	postive
HELLS	PRRT3-AS1	0.46537546	2.54E-30	postive
HELLS	AC100830.2	0.485059647	3.70E-33	postive
ZNF419	AC100830.2	0.453248137	1.15E-28	postive
KLHL24	AC100830.2	0.432222562	6.07E-26	postive
TUBE1	AC100830.2	0.4913868	4.14E-34	postive
ALOX12	AC100830.2	0.534079418	4.46E-41	postive
IREB2	AC100830.2	0.438079792	1.11E-26	postive
GABPB1	AC100830.2	0.57528695	8.13E-49	postive
PIK3CA	AC100830.2	0.401692918	2.57E-22	postive
MAPK8	AC100830.2	0.484544668	4.41E-33	postive
LINC00472	AC100830.2	0.772493156	5.58E-108	postive
ATM	AC100830.2	0.643325863	2.72E-64	postive
FBXW7	AC100830.2	0.480248453	1.90E-32	postive
HELLS	MACC1-AS1	0.467211531	1.40E-30	postive
GABPB1	MACC1-AS1	0.451661026	1.88E-28	postive
LINC00472	MACC1-AS1	0.40828956	4.54E-23	postive
ATM	MACC1-AS1	0.625035345	9.39E-60	postive
MTOR	AL450326.1	0.454347465	8.22E-29	postive
MAFG	AL450326.1	0.451971568	1.71E-28	postive
CS	AL450326.1	0.421261866	1.34E-24	postive
LPCAT3	AL450326.1	0.400418162	3.57E-22	postive
BECN1	AL450326.1	0.49307971	2.29E-34	postive
HELLS	AC092653.1	0.537687465	1.03E-41	postive
TUBE1	AC092653.1	0.512299099	2.12E-37	postive
ALOX12	AC092653.1	0.527867856	5.31E-40	postive
GABPB1	AC092653.1	0.560621267	6.15E-46	postive
MAPK8	AC092653.1	0.403404845	1.64E-22	postive
LINC00472	AC092653.1	0.63004822	5.74E-61	postive
ATM	AC092653.1	0.74941783	3.14E-98	postive
FBXW7	AC092653.1	0.476492434	6.69E-32	postive
ZNF419	AC010976.2	0.435534363	2.33E-26	postive
VEGFA	AC010976.2	0.433287226	4.47E-26	postive
TUBE1	AC010976.2	0.409306638	3.46E-23	postive
ALOX12	AC010976.2	0.444668567	1.57E-27	postive
TAZ	AC010976.2	0.464893667	2.96E-30	postive
ZNF419	BX649601.1	0.462481585	6.40E-30	postive
ALOX12	BX649601.1	0.505317177	2.82E-36	postive
TAZ	BX649601.1	0.471778767	3.18E-31	postive
NQO1	AP003119.2	0.420648097	1.59E-24	postive
ASNS	AP003119.2	0.411367253	2.00E-23	postive
PSAT1	AP003119.2	0.51361873	1.29E-37	postive
G6PD	AP003119.2	0.470039093	5.61E-31	postive
SLC1A5	AP003119.2	0.416990029	4.35E-24	postive
HELLS	TMEM30A-D	0.480548493	1.72E-32	postive
ZNF419	TMEM30A-D	0.516136235	4.99E-38	postive
TUBE1	TMEM30A-D	0.652126427	1.37E-66	postive
ALOX12	TMEM30A-D	0.638856183	3.73E-63	postive
IREB2	TMEM30A-D	0.443960536	1.94E-27	postive
GABPB1	TMEM30A-D	0.605447785	3.24E-55	postive
MAPK8	TMEM30A-D	0.553954546	1.12E-44	postive
LINC00472	TMEM30A-D	0.563123523	2.03E-46	postive
ATM	TMEM30A-D	0.688512376	5.91E-77	postive
FBXW7	TMEM30A-D	0.535186067	2.85E-41	postive
ISCU	UBAC2-AS1	0.426930649	2.75E-25	postive
ATP5MC3	UBAC2-AS1	0.402795682	1.93E-22	postive
SLC2A8	UBAC2-AS1	0.425676625	3.91E-25	postive
SLC2A12	UBAC2-AS1	0.411777191	1.79E-23	postive

ATG4D	UBAC2-AS1	0.424908305	4.85E-25	postive
GABARAPL2	UBAC2-AS1	0.514489066	9.31E-38	postive
WIPI2	UBAC2-AS1	0.449744672	3.38E-28	postive
HELLS	AP001469.3	0.581277241	4.92E-50	postive
ZNF419	AP001469.3	0.460191982	1.32E-29	postive
TUBE1	AP001469.3	0.459624231	1.58E-29	postive
ALOX12	AP001469.3	0.596566524	2.92E-53	postive
GABPB1	AP001469.3	0.513113507	1.56E-37	postive
LINC00472	AP001469.3	0.57982943	9.74E-50	postive
ATM	AP001469.3	0.56139139	4.38E-46	postive
HELLS	AL645939.4	0.457036197	3.56E-29	postive
TUBE1	AL645939.4	0.49750735	4.76E-35	postive
ALOX12	AL645939.4	0.455920324	5.04E-29	postive
GABPB1	AL645939.4	0.435656286	2.25E-26	postive
ATM	AL645939.4	0.507707201	1.17E-36	postive
HELLS	AL606834.2	0.46686953	1.57E-30	postive
ATM	AL606834.2	0.407140674	6.15E-23	postive
FBXW7	AL606834.2	0.41950948	2.18E-24	postive
ZNF419	ZNF32-AS2	0.555652897	5.40E-45	postive
VEGFA	ZNF32-AS2	0.57805922	2.24E-49	postive
TUBE1	ZNF32-AS2	0.61137897	1.48E-56	postive
SETD1B	ZNF32-AS2	0.549285724	8.28E-44	postive
ALOX12	ZNF32-AS2	0.667119732	1.11E-70	postive
GABPB1	ZNF32-AS2	0.400192256	3.78E-22	postive
ATM	ZNF32-AS2	0.486908623	1.96E-33	postive
YY1AP1	ZNF32-AS2	0.427987709	2.04E-25	postive
TAZ	ZNF32-AS2	0.558885973	1.32E-45	postive
FBXW7	ZNF32-AS2	0.44581214	1.11E-27	postive
HELLS	CFLAR-AS1	0.569563909	1.12E-47	postive
TUBE1	CFLAR-AS1	0.458203992	2.47E-29	postive
ALOX12	CFLAR-AS1	0.464265455	3.62E-30	postive
GABPB1	CFLAR-AS1	0.544371868	6.54E-43	postive
ATG7	CFLAR-AS1	0.453555871	1.05E-28	postive
LINC00472	CFLAR-AS1	0.586359184	4.35E-51	postive
ATM	CFLAR-AS1	0.754968779	1.78E-100	postive
FBXW7	CFLAR-AS1	0.491832875	3.54E-34	postive
ALOX12	AP003717.1	0.414876619	7.74E-24	postive
ZNF419	AP001462.1	0.429781117	1.22E-25	postive
ALOX12	AP001462.1	0.490584341	5.48E-34	postive
TFAP2C	AP001462.1	0.42213926	1.05E-24	postive
HBA1	AP001462.1	0.577849473	2.47E-49	postive
EGLN2	AP001462.1	0.486551379	2.22E-33	postive
ZNF419	LINC00893	0.572146086	3.46E-48	postive
VEGFA	LINC00893	0.431284934	7.95E-26	postive
TUBE1	LINC00893	0.575950547	5.97E-49	postive
SETD1B	LINC00893	0.470460163	4.89E-31	postive
ALOX12	LINC00893	0.632970282	1.10E-61	postive
PHKG2	LINC00893	0.529362725	2.94E-40	postive
TAZ	LINC00893	0.661456795	4.15E-69	postive
TFAP2C	AC022613.2	0.45412046	8.82E-29	postive
HBA1	AC022613.2	0.644766859	1.16E-64	postive
FANCD2	AL136531.1	0.439359744	7.60E-27	postive
HELLS	AL136531.1	0.551012389	3.97E-44	postive
TUBE1	AL136531.1	0.426079055	3.49E-25	postive
ALOX12	AL136531.1	0.589581186	9.13E-52	postive
GABPB1	AL136531.1	0.431739401	6.98E-26	postive
DUOX1	AL136531.1	0.410619221	2.44E-23	postive
LINC00472	AL136531.1	0.409349117	3.42E-23	postive

ATM	AL136531.1	0.451417876	2.03E-28	postive
FANCD2	AC020913.3	0.411136214	2.12E-23	postive
HELLS	AC020913.3	0.438119242	1.09E-26	postive
ALOX12	AC020913.3	0.422937076	8.43E-25	postive
TFAP2C	AC020913.3	0.519569415	1.35E-38	postive
HBA1	AC020913.3	0.47390457	1.58E-31	postive
DUOX1	AC020913.3	0.460394526	1.24E-29	postive
PML	KIF1C-AS1	0.434995459	2.72E-26	postive
BRD4	KIF1C-AS1	0.414099796	9.56E-24	postive
ZNF419	KIF1C-AS1	0.414024936	9.75E-24	postive
TUBE1	KIF1C-AS1	0.416208394	5.39E-24	postive
SETD1B	KIF1C-AS1	0.404272161	1.31E-22	postive
ALOX12	KIF1C-AS1	0.545332391	4.38E-43	postive
PHKG2	KIF1C-AS1	0.573406326	1.94E-48	postive
TAZ	KIF1C-AS1	0.654435639	3.33E-67	postive
FBXW7	KIF1C-AS1	0.400857506	3.19E-22	postive
HELLS	AC073130.2	0.561826549	3.61E-46	postive
TUBE1	AC073130.2	0.437611955	1.27E-26	postive
ALOX12	AC073130.2	0.435926316	2.08E-26	postive
GABPB1	AC073130.2	0.512216551	2.19E-37	postive
ATG7	AC073130.2	0.417017044	4.32E-24	postive
LINC00472	AC073130.2	0.573797557	1.62E-48	postive
ATM	AC073130.2	0.697163444	1.20E-79	postive
FBXW7	AC073130.2	0.443988826	1.92E-27	postive
FANCD2	AP001628.1	0.42028992	1.76E-24	postive
HELLS	AP001628.1	0.503039626	6.48E-36	postive
ALOX12	AP001628.1	0.515397216	6.61E-38	postive
TFAP2C	AP001628.1	0.417688286	3.60E-24	postive
DUOX1	AP001628.1	0.412717309	1.39E-23	postive
OXSR1	AC097534.1	0.427323118	2.46E-25	postive
ZNF419	AC097534.1	0.413197546	1.22E-23	postive
TUBE1	AC097534.1	0.455852406	5.15E-29	postive
ALOX12	AC097534.1	0.452875417	1.29E-28	postive
IREB2	AC097534.1	0.430565162	9.77E-26	postive
SP1	AC097534.1	0.485336986	3.37E-33	postive
GABPB1	AC097534.1	0.452625082	1.40E-28	postive
MAPK8	AC097534.1	0.439249928	7.85E-27	postive
PRKAA2	AC097534.1	0.404058189	1.38E-22	postive
ATM	AC097534.1	0.564264493	1.22E-46	postive
HELLS	GEMIN7-AS1	0.461345107	9.18E-30	postive
ZNF419	GEMIN7-AS1	0.471490325	3.49E-31	postive
TUBE1	GEMIN7-AS1	0.531926842	1.06E-40	postive
ALOX12	GEMIN7-AS1	0.692653548	3.12E-78	postive
PHKG2	GEMIN7-AS1	0.424411335	5.58E-25	postive
ATM	GEMIN7-AS1	0.437940453	1.15E-26	postive
TAZ	GEMIN7-AS1	0.515600167	6.12E-38	postive
HELLS	AC023510.2	0.451190369	2.17E-28	postive
TUBE1	AC023510.2	0.659498231	1.43E-68	postive
ALOX12	AC023510.2	0.574575599	1.13E-48	postive
GABPB1	AC023510.2	0.51795425	2.50E-38	postive
ATM	AC023510.2	0.671518963	6.27E-72	postive
FBXW7	AC023510.2	0.470895658	4.24E-31	postive
TAZ	AC008438.1	0.419946554	1.93E-24	postive
HBA1	Z93930.2	0.45104628	2.27E-28	postive
TUBE1	AC009974.1	0.511179636	3.23E-37	postive
ALOX12	AC009974.1	0.465476161	2.46E-30	postive
TAZ	AC009974.1	0.475090839	1.07E-31	postive
HELLS	AL021707.1	0.411698083	1.83E-23	postive

ZNF419	AL021707.1	0.550763758	4.42E-44	postive
VEGFA	AL021707.1	0.400588408	3.42E-22	postive
TUBE1	AL021707.1	0.587887836	2.08E-51	postive
ALOX12	AL021707.1	0.564868257	9.33E-47	postive
GABPB1	AL021707.1	0.455566691	5.63E-29	postive
PHKG2	AL021707.1	0.42433824	5.70E-25	postive
LINC00472	AL021707.1	0.417937217	3.36E-24	postive
ATM	AL021707.1	0.496890103	5.93E-35	postive
TAZ	AL021707.1	0.565581161	6.78E-47	postive
FBXW7	AL021707.1	0.511705309	2.65E-37	postive
STAT3	AC011374.2	-0.437425659	1.34E-26	negative
NOX1	AC011374.2	0.570392946	7.71E-48	postive
PHKG2	AC011374.2	0.490159161	6.35E-34	postive
BECN1	AC011374.2	-0.558527407	1.54E-45	negative
TFAP2C	AC022382.1	0.561894181	3.51E-46	postive
HBA1	AC022382.1	0.497269913	5.18E-35	postive
DUOX1	AC022382.1	0.403977368	1.41E-22	postive
FANCD2	AC018766.1	0.417042073	4.29E-24	postive
HELLS	AC018766.1	0.454480313	7.89E-29	postive
ZNF419	AC018766.1	0.421394042	1.29E-24	postive
ALOX12	AC018766.1	0.496396088	7.07E-35	postive
TFAP2C	AC018766.1	0.50078971	1.46E-35	postive
HBA1	AC018766.1	0.420488719	1.66E-24	postive
DUOX1	AC018766.1	0.429480002	1.33E-25	postive
SLC2A12	LINC01564	0.426171773	3.40E-25	postive
PHKG2	AC007220.1	0.469957633	5.76E-31	postive
TAZ	AC007220.1	0.547338268	1.89E-43	postive
HSPB1	CHKB-DT	0.460631529	1.15E-29	postive
SLC2A8	CHKB-DT	0.454321686	8.28E-29	postive
PHKG2	CHKB-DT	0.412127027	1.63E-23	postive
HELLS	AC025287.3	0.414731241	8.05E-24	postive
ZNF419	AC025287.3	0.555678319	5.34E-45	postive
TUBE1	AC025287.3	0.568355099	1.94E-47	postive
SETD1B	AC025287.3	0.534214057	4.22E-41	postive
ALOX12	AC025287.3	0.677615053	1.08E-73	postive
GABPB1	AC025287.3	0.477703065	4.47E-32	postive
LINC00472	AC025287.3	0.513973165	1.13E-37	postive
ATM	AC025287.3	0.62403393	1.63E-59	postive
FBXW7	AC025287.3	0.493626259	1.89E-34	postive
HELLS	AC087276.1	0.54789829	1.49E-43	postive
ZNF419	AC087276.1	0.437383559	1.36E-26	postive
TUBE1	AC087276.1	0.469882223	5.91E-31	postive
ALOX12	AC087276.1	0.521009939	7.72E-39	postive
GABPB1	AC087276.1	0.559466447	1.02E-45	postive
LINC00472	AC087276.1	0.678357573	6.54E-74	postive
ATM	AC087276.1	0.621382277	6.97E-59	postive
FBXW7	AC087276.1	0.532715237	7.71E-41	postive
LPIN1	LINC01762	0.410168856	2.75E-23	postive
RPL8	AC138207.2	0.534491592	3.77E-41	postive
HELLS	AL354696.2	0.42660368	3.01E-25	postive
ALOX12	AL354696.2	0.455168888	6.37E-29	postive
TFAP2C	AL354696.2	0.443389821	2.30E-27	postive
HBA1	AL354696.2	0.433636055	4.04E-26	postive
DUOX1	AL354696.2	0.418945059	2.55E-24	postive
GCLC	AGAP2-AS1	-0.426976559	2.71E-25	negative
TRIB3	AGAP2-AS1	0.462704547	5.96E-30	postive
SLC2A6	AGAP2-AS1	0.449367119	3.79E-28	postive
NCOA4	AGAP2-AS1	-0.439051509	8.32E-27	negative

PRKAA2	AGAP2-AS1	-0.422253061	1.02E-24	negative
GPX4	RAB11B-AS1	0.499128203	2.66E-35	postive
RB1	RAB11B-AS1	-0.437431086	1.34E-26	negative
HSPB1	RAB11B-AS1	0.413468259	1.13E-23	postive
STAT3	RAB11B-AS1	-0.476085621	7.66E-32	negative
OTUB1	RAB11B-AS1	0.435394249	2.43E-26	postive
MAPK14	RAB11B-AS1	-0.477909097	4.17E-32	negative
SLC2A8	RAB11B-AS1	0.466822264	1.59E-30	postive
NRAS	RAB11B-AS1	-0.498762858	3.04E-35	negative
HRAS	RAB11B-AS1	0.554005243	1.10E-44	postive
NCOA4	RAB11B-AS1	-0.46209796	7.23E-30	negative
PHKG2	RAB11B-AS1	0.479210706	2.69E-32	postive
ATG4D	RAB11B-AS1	0.425715308	3.87E-25	postive
BECN1	RAB11B-AS1	-0.506042888	2.16E-36	negative
MAP1LC3A	RAB11B-AS1	0.615330223	1.82E-57	postive
MAPK1	RAB11B-AS1	-0.519983915	1.15E-38	negative
ANO6	RAB11B-AS1	-0.417349933	3.95E-24	negative
EGLN2	RAB11B-AS1	0.459475455	1.66E-29	postive
MTDH	RAB11B-AS1	-0.46762179	1.23E-30	negative
SIRT1	RAB11B-AS1	-0.437349249	1.37E-26	negative
BRD4	AC009283.1	0.41796237	3.34E-24	postive
ZNF419	AC009283.1	0.524307446	2.15E-39	postive
TUBE1	AC009283.1	0.47599656	7.89E-32	postive
SETD1B	AC009283.1	0.418081992	3.23E-24	postive
DRD4	AC009283.1	0.407216528	6.03E-23	postive
ALOX12	AC009283.1	0.558312666	1.70E-45	postive
NCOA4	AC009283.1	-0.448969104	4.28E-28	negative
PHKG2	AC009283.1	0.657657713	4.52E-68	postive
MAPK1	AC009283.1	-0.405856261	8.64E-23	negative
TAZ	AC009283.1	0.76644582	2.57E-105	postive
BRD4	IGBP1-AS1	0.415730597	6.14E-24	postive
ZNF419	IGBP1-AS1	0.578344992	1.96E-49	postive
TUBE1	IGBP1-AS1	0.544875237	5.30E-43	postive
SETD1B	IGBP1-AS1	0.452799883	1.33E-28	postive
ALOX12	IGBP1-AS1	0.568925364	1.50E-47	postive
GABPB1	IGBP1-AS1	0.469564472	6.55E-31	postive
LINC00472	IGBP1-AS1	0.568842238	1.56E-47	postive
ATM	IGBP1-AS1	0.457469599	3.11E-29	postive
TAZ	IGBP1-AS1	0.493364249	2.07E-34	postive
FBXW7	IGBP1-AS1	0.412123005	1.63E-23	postive
TRIB3	AC016405.3	0.411108024	2.14E-23	postive
ZNF419	Z94721.1	0.402158326	2.27E-22	postive
VEGFA	Z94721.1	0.466474765	1.78E-30	postive
TUBE1	Z94721.1	0.558259202	1.74E-45	postive
ALOX12	Z94721.1	0.655353101	1.89E-67	postive
ATM	Z94721.1	0.401395581	2.77E-22	postive
HELLS	AC018926.3	0.464422194	3.45E-30	postive
KLHL24	AC018926.3	0.485815573	2.86E-33	postive
TUBE1	AC018926.3	0.478654672	3.25E-32	postive
ALOX12	AC018926.3	0.404869776	1.12E-22	postive
IREB2	AC018926.3	0.41729076	4.01E-24	postive
GABPB1	AC018926.3	0.563040416	2.11E-46	postive
PIK3CA	AC018926.3	0.416356266	5.18E-24	postive
MAPK8	AC018926.3	0.423637887	6.93E-25	postive
LINC00472	AC018926.3	0.846742437	2.66E-149	postive
ATM	AC018926.3	0.674684443	7.70E-73	postive
FBXW7	AC018926.3	0.481522981	1.23E-32	postive
CHMP6	AL451085.2	0.417558095	3.73E-24	postive

HRAS	AL451085.2	0.532766726	7.56E-41	postive
TAZ	AL451085.2	0.437383171	1.36E-26	postive
PHKG2	PPP4R3B-DT	0.463642556	4.42E-30	postive
TAZ	PPP4R3B-DT	0.480536341	1.72E-32	postive
HELLS	AC124283.2	0.483231971	6.91E-33	postive
HELLS	AC127024.5	0.576740613	4.14E-49	postive
ZNF419	AC127024.5	0.568382902	1.92E-47	postive
TUBE1	AC127024.5	0.651130752	2.52E-66	postive
ALOX12	AC127024.5	0.663175988	1.39E-69	postive
GABPB1	AC127024.5	0.609012086	5.10E-56	postive
LINC00472	AC127024.5	0.569796148	1.01E-47	postive
ATM	AC127024.5	0.66322695	1.35E-69	postive
TAZ	AC127024.5	0.426246463	3.33E-25	postive
FBXW7	AC127024.5	0.554186579	1.02E-44	postive
HELLS	AC010261.1	0.49344827	2.01E-34	postive
KLHL24	AC010261.1	0.481103772	1.42E-32	postive
TUBE1	AC010261.1	0.433967577	3.67E-26	postive
ALOX12	AC010261.1	0.4192716	2.33E-24	postive
IREB2	AC010261.1	0.434839905	2.85E-26	postive
GABPB1	AC010261.1	0.557575063	2.34E-45	postive
PIK3CA	AC010261.1	0.438812942	8.93E-27	postive
MAPK8	AC010261.1	0.430139567	1.10E-25	postive
LINC00472	AC010261.1	0.86902595	3.09E-166	postive
ATM	AC010261.1	0.658033719	3.57E-68	postive
FBXW7	AC010261.1	0.439455756	7.39E-27	postive
FANCD2	AC009961.1	0.401352365	2.80E-22	postive
HELLS	AC009961.1	0.455494584	5.76E-29	postive
TFAP2C	AC009961.1	0.489455221	8.12E-34	postive
HBA1	AC009961.1	0.457229097	3.35E-29	postive
DUOX1	AC009961.1	0.418800665	2.65E-24	postive
BRD4	AL162586.1	0.498738508	3.06E-35	postive
ZNF419	AL162586.1	0.6043704	5.63E-55	postive
VEGFA	AL162586.1	0.500551602	1.60E-35	postive
TUBE1	AL162586.1	0.563158202	2.00E-46	postive
SETD1B	AL162586.1	0.504232969	4.20E-36	postive
DRD4	AL162586.1	0.425608776	3.99E-25	postive
ALOX12	AL162586.1	0.636189974	1.74E-62	postive
GABPB1	AL162586.1	0.449492029	3.65E-28	postive
TAZ	AL162586.1	0.591324519	3.90E-52	postive
FBXW7	AL162586.1	0.439097052	8.21E-27	postive
HELLS	AP005136.3	0.555593103	5.54E-45	postive
ZNF419	AP005136.3	0.408154494	4.70E-23	postive
TUBE1	AP005136.3	0.512250684	2.16E-37	postive
ALOX12	AP005136.3	0.433226078	4.55E-26	postive
GABPB1	AP005136.3	0.59955663	6.51E-54	postive
MAPK8	AP005136.3	0.438567438	9.60E-27	postive
LINC00472	AP005136.3	0.743895548	4.71E-96	postive
ATM	AP005136.3	0.661861215	3.21E-69	postive
FBXW7	AP005136.3	0.473806343	1.63E-31	postive
ZNF419	AC006213.5	0.434558421	3.09E-26	postive
KLHL24	AC006213.5	0.497008781	5.68E-35	postive
IREB2	AC006213.5	0.493997173	1.65E-34	postive
GABPB1	AC006213.5	0.509951954	5.10E-37	postive
PIK3CA	AC006213.5	0.460794095	1.09E-29	postive
KRAS	AC006213.5	0.408401901	4.41E-23	postive
MAPK8	AC006213.5	0.499302044	2.50E-35	postive
LINC00472	AC006213.5	0.685736004	4.13E-76	postive
PRKAA1	AC006213.5	0.432142265	6.21E-26	postive

ATM	AC006213.5	0.550380035	5.20E-44	postive
ZNF419	AP000892.3	0.536005993	2.04E-41	postive
ALOX12	AP000892.3	0.478830809	3.06E-32	postive
PHKG2	AP000892.3	0.427729155	2.19E-25	postive
EGLN2	AP000892.3	0.467281432	1.37E-30	postive
TAZ	AP000892.3	0.573835257	1.59E-48	postive
HELLS	STARD4-AS1	0.445883217	1.09E-27	postive
ZNF419	STARD4-AS1	0.482491301	8.89E-33	postive
VEGFA	STARD4-AS1	0.419677034	2.08E-24	postive
TUBE1	STARD4-AS1	0.579374101	1.21E-49	postive
SETD1B	STARD4-AS1	0.413355079	1.17E-23	postive
ALOX12	STARD4-AS1	0.619561026	1.88E-58	postive
IREB2	STARD4-AS1	0.407972346	4.94E-23	postive
SP1	STARD4-AS1	0.410683873	2.40E-23	postive
GABPB1	STARD4-AS1	0.505803243	2.36E-36	postive
ZEB1	STARD4-AS1	0.422045826	1.08E-24	postive
MAPK8	STARD4-AS1	0.48977503	7.26E-34	postive
LINC00472	STARD4-AS1	0.408376365	4.44E-23	postive
ATM	STARD4-AS1	0.693471948	1.73E-78	postive
FBXW7	STARD4-AS1	0.49882283	2.97E-35	postive
CHAC1	AL049629.1	0.585807287	5.67E-51	postive
DDIT3	AL049629.1	0.475434621	9.51E-32	postive
ATG4D	AL049629.1	0.463387425	4.80E-30	postive
MAP1LC3A	AL049629.1	0.534643854	3.55E-41	postive
WIPI2	AL049629.1	0.402312364	2.18E-22	postive
FANCD2	AC005776.2	0.41423831	9.21E-24	postive
HELLS	AC005776.2	0.400247458	3.73E-22	postive
ZNF419	AC005776.2	0.401094654	3.00E-22	postive
ALOX12	AC005776.2	0.488392146	1.17E-33	postive
TFAP2C	AC005776.2	0.487356559	1.68E-33	postive
HBA1	AC005776.2	0.435334828	2.47E-26	postive
DUOX1	AC005776.2	0.41520399	7.08E-24	postive
PHKG2	AC015660.3	0.521206808	7.16E-39	postive
TAZ	AC015660.3	0.617974733	4.42E-58	postive
HELLS	AC112722.1	0.468458293	9.38E-31	postive
ZNF419	AC112722.1	0.529278307	3.04E-40	postive
TUBE1	AC112722.1	0.539186528	5.59E-42	postive
ALOX12	AC112722.1	0.559714884	9.17E-46	postive
GABPB1	AC112722.1	0.471962392	2.99E-31	postive
ATM	AC112722.1	0.557551721	2.36E-45	postive
FBXW7	AC112722.1	0.528961517	3.45E-40	postive
ALOX12	SNAI3-AS1	0.42178078	1.16E-24	postive
HBA1	SNAI3-AS1	0.580812086	6.13E-50	postive
EGLN2	SNAI3-AS1	0.473358019	1.89E-31	postive
ATM	AC023137.1	0.456946225	3.66E-29	postive
FANCD2	AC138393.2	0.43575782	2.18E-26	postive
HELLS	AC138393.2	0.615318497	1.83E-57	postive
ZNF419	AC138393.2	0.431470811	7.53E-26	postive
TUBE1	AC138393.2	0.496570319	6.64E-35	postive
ALOX12	AC138393.2	0.554591359	8.54E-45	postive
GABPB1	AC138393.2	0.570019461	9.13E-48	postive
ATG7	AC138393.2	0.417336475	3.96E-24	postive
MAPK8	AC138393.2	0.400830345	3.21E-22	postive
LINC00472	AC138393.2	0.599828976	5.68E-54	postive
ATM	AC138393.2	0.688083833	7.99E-77	postive
FBXW7	AC138393.2	0.545397729	4.26E-43	postive
HELLS	AC079921.1	0.491612325	3.82E-34	postive
ZNF419	AC079921.1	0.430661243	9.50E-26	postive

TUBE1	AC079921.1	0.54417673	7.10E-43	postive
ALOX12	AC079921.1	0.528034868	4.97E-40	postive
IREB2	AC079921.1	0.407993479	4.91E-23	postive
GABPB1	AC079921.1	0.627122952	2.95E-60	postive
ZEB1	AC079921.1	0.408994447	3.76E-23	postive
MAPK8	AC079921.1	0.488124456	1.29E-33	postive
LINC00472	AC079921.1	0.633937215	6.34E-62	postive
ATM	AC079921.1	0.72727708	7.89E-90	postive
FBXW7	AC079921.1	0.54471659	5.67E-43	postive
HELLS	AL049840.6	0.506176263	2.06E-36	postive
KLHL24	AL049840.6	0.47239473	2.60E-31	postive
TUBE1	AL049840.6	0.459125335	1.85E-29	postive
ALOX12	AL049840.6	0.409764066	3.07E-23	postive
IREB2	AL049840.6	0.458035245	2.60E-29	postive
GABPB1	AL049840.6	0.606522484	1.86E-55	postive
PIK3CA	AL049840.6	0.471376719	3.62E-31	postive
ATG7	AL049840.6	0.429874989	1.19E-25	postive
MAPK8	AL049840.6	0.472479494	2.52E-31	postive
LINC00472	AL049840.6	0.844121278	1.73E-147	postive
ATM	AL049840.6	0.721234152	1.12E-87	postive
FBXW7	AL049840.6	0.495162638	1.10E-34	postive
ZNF419	ZFHX2-AS1	0.452002519	1.69E-28	postive
ALOX12	ZFHX2-AS1	0.562299083	2.93E-46	postive
TFAP2C	ZFHX2-AS1	0.474128121	1.47E-31	postive
HBA1	ZFHX2-AS1	0.469737912	6.19E-31	postive
DUOX1	ZFHX2-AS1	0.400597002	3.41E-22	postive
HELLS	MAST4-AS1	0.539665919	4.59E-42	postive
GABPB1	MAST4-AS1	0.507875459	1.10E-36	postive
ATG7	MAST4-AS1	0.451850201	1.77E-28	postive
LINC00472	MAST4-AS1	0.511238225	3.16E-37	postive
ATM	MAST4-AS1	0.701194176	6.16E-81	postive
FBXW7	MAST4-AS1	0.443216381	2.42E-27	postive
NCF2	AC026369.3	0.406506634	7.28E-23	postive
ISCU	AC006547.1	0.503010216	6.55E-36	postive
DDIT3	AC006547.1	0.433434926	4.28E-26	postive
KLHL24	AC006547.1	0.400243445	3.73E-22	postive
SLC2A12	AC006547.1	0.41637436	5.15E-24	postive
TFAP2C	AC006547.1	0.400980437	3.09E-22	postive
ATG4D	AC006547.1	0.457560434	3.02E-29	postive
GABARAPL1	AC006547.1	0.58392086	1.40E-50	postive
WIPI2	AC006547.1	0.489698954	7.46E-34	postive
LPIN1	AC006547.1	0.677194727	1.43E-73	postive
TFAP2C	AL031289.1	0.582240453	3.12E-50	postive
HBA1	AL031289.1	0.521587567	6.18E-39	postive
HELLS	AL162385.2	0.518187206	2.28E-38	postive
TUBE1	AL162385.2	0.507681717	1.18E-36	postive
ALOX12	AL162385.2	0.425692057	3.89E-25	postive
GABPB1	AL162385.2	0.578149179	2.14E-49	postive
MAPK8	AL162385.2	0.423710955	6.79E-25	postive
LINC00472	AL162385.2	0.780308378	1.51E-111	postive
ATM	AL162385.2	0.672131555	4.19E-72	postive
FBXW7	AL162385.2	0.503649566	5.19E-36	postive
RB1	AC005034.3	0.467057635	1.48E-30	postive
ACSL3	AC005034.3	0.413023363	1.28E-23	postive
KLHL24	AC005034.3	0.449098659	4.12E-28	postive
IREB2	AC005034.3	0.466712241	1.65E-30	postive
PIK3CA	AC005034.3	0.523976712	2.44E-39	postive
KRAS	AC005034.3	0.564561236	1.07E-46	postive

SLC38A1	AC005034.3	0.439856231	6.57E-27	postive
MAPK9	AC005034.3	0.438662512	9.33E-27	postive
LINC00472	AC005034.3	0.517835037	2.61E-38	postive
PRKAA2	AC005034.3	0.429694178	1.25E-25	postive
PRKAA1	AC005034.3	0.466060086	2.04E-30	postive
TGFBR1	AC005034.3	0.459013422	1.92E-29	postive
BACH1	AC005034.3	0.466439257	1.80E-30	postive
HELLS	AC068987.2	0.49405893	1.62E-34	postive
TUBE1	AC068987.2	0.515137128	7.29E-38	postive
ALOX12	AC068987.2	0.636543208	1.42E-62	postive
GABPB1	AC068987.2	0.426410609	3.18E-25	postive
ATM	AC068987.2	0.623537555	2.14E-59	postive
FBXW7	AC068987.2	0.412291541	1.56E-23	postive
HSPB1	AL035071.1	0.434411904	3.23E-26	postive
STAT3	AL035071.1	-0.436506466	1.75E-26	negative
MAPK14	AL035071.1	-0.519159798	1.57E-38	negative
NOX1	AL035071.1	0.520159672	1.07E-38	postive
NRAS	AL035071.1	-0.452505042	1.45E-28	negative
HRAS	AL035071.1	0.407165574	6.11E-23	postive
NCOA4	AL035071.1	-0.577849912	2.47E-49	negative
PHKG2	AL035071.1	0.558872858	1.33E-45	postive
BECN1	AL035071.1	-0.596681853	2.76E-53	negative
MAPK1	AL035071.1	-0.46992277	5.83E-31	negative
ANO6	AL035071.1	-0.447744772	6.21E-28	negative
TAZ	AL035071.1	0.522206305	4.86E-39	postive
MTDH	AL035071.1	-0.463496816	4.63E-30	negative
NCOA4	AC010331.1	-0.425350121	4.29E-25	negative
PHKG2	AC010331.1	0.597486876	1.84E-53	postive
ULK1	AC010331.1	0.411934087	1.71E-23	postive
TAZ	AC010331.1	0.66788386	6.74E-71	postive
ATG4D	LMO7-AS1	0.407360085	5.81E-23	postive
GABARAPL2	LMO7-AS1	0.404519985	1.23E-22	postive
ALOX12	AC012368.1	0.405913781	8.51E-23	postive
TAZ	AC012368.1	0.56437162	1.17E-46	postive
ZNF419	AC092574.1	0.42085064	1.50E-24	postive
TUBE1	AC092574.1	0.43789667	1.17E-26	postive
GABPB1	AC092574.1	0.444606468	1.60E-27	postive
LINC00472	AC092574.1	0.610171661	2.78E-56	postive
ZNF419	AC073655.2	0.429889113	1.18E-25	postive
TUBE1	AC073655.2	0.512386696	2.05E-37	postive
ALOX12	AC073655.2	0.522770454	3.91E-39	postive
PHKG2	AC073655.2	0.479879013	2.15E-32	postive
TAZ	AC073655.2	0.630670902	4.04E-61	postive
SLC3A2	AC010501.2	0.448363957	5.14E-28	postive
FH	AC010501.2	0.448227266	5.36E-28	postive
ISCU	AC010501.2	0.676423946	2.41E-73	postive
ACSL3	AC010501.2	0.401398357	2.77E-22	postive
DDIT3	AC010501.2	0.475800992	8.42E-32	postive
KLHL24	AC010501.2	0.444621126	1.59E-27	postive
SLC2A8	AC010501.2	0.430289842	1.06E-25	postive
CS	AC010501.2	0.449706828	3.42E-28	postive
GOT1	AC010501.2	0.452270608	1.56E-28	postive
ATG4D	AC010501.2	0.601178154	2.87E-54	postive
MAP1LC3A	AC010501.2	0.440158798	6.00E-27	postive
GABARAPL2	AC010501.2	0.462356563	6.66E-30	postive
GABARAPL1	AC010501.2	0.772173473	7.75E-108	postive
WIPI2	AC010501.2	0.413411046	1.15E-23	postive
LPIN1	AC010501.2	0.609691009	3.58E-56	postive

FANCD2	AL049780.2	0.436509401	1.75E-26	postive
HELLS	AL049780.2	0.558966564	1.27E-45	postive
ZNF419	AL049780.2	0.423169452	7.90E-25	postive
TUBE1	AL049780.2	0.453891302	9.46E-29	postive
ALOX12	AL049780.2	0.548372876	1.22E-43	postive
GABPB1	AL049780.2	0.517873397	2.58E-38	postive
MAPK8	AL049780.2	0.413244178	1.20E-23	postive
LINC00472	AL049780.2	0.507857568	1.11E-36	postive
ATM	AL049780.2	0.534589934	3.63E-41	postive
FBXW7	AL049780.2	0.421901269	1.12E-24	postive
HELLS	AC010320.3	0.50655677	1.79E-36	postive
ZNF419	AC010320.3	0.544611253	5.92E-43	postive
KLHL24	AC010320.3	0.401019235	3.06E-22	postive
TUBE1	AC010320.3	0.527786092	5.49E-40	postive
ALOX12	AC010320.3	0.563858888	1.46E-46	postive
GABPB1	AC010320.3	0.548763548	1.03E-43	postive
LINC00472	AC010320.3	0.634393321	4.88E-62	postive
ATM	AC010320.3	0.6615554	3.90E-69	postive
FBXW7	AC010320.3	0.530611162	1.79E-40	postive
SETD1B	AC026471.1	0.403185097	1.74E-22	postive
ALOX12	AC026471.1	0.528516748	4.11E-40	postive
ATM	AC026471.1	0.463908126	4.06E-30	postive
TFAP2C	AC107057.1	0.431155226	8.25E-26	postive
HBA1	AC107057.1	0.543655121	8.82E-43	postive
FANCD2	AC004076.2	0.444605246	1.60E-27	postive
HELLS	AC004076.2	0.580823338	6.09E-50	postive
ZNF419	AC004076.2	0.643872955	1.97E-64	postive
TUBE1	AC004076.2	0.494369297	1.45E-34	postive
ALOX12	AC004076.2	0.571389283	4.89E-48	postive
GABPB1	AC004076.2	0.553679523	1.27E-44	postive
LINC00472	AC004076.2	0.502725386	7.27E-36	postive
ATM	AC004076.2	0.49716353	5.38E-35	postive
FBXW7	AC004076.2	0.492942802	2.40E-34	postive
ISCU	AC012555.1	0.463234717	5.03E-30	postive
ACSL3	AC012555.1	0.410060312	2.83E-23	postive
KLHL24	AC012555.1	0.50601772	2.18E-36	postive
CS	AC012555.1	0.414204725	9.29E-24	postive
GABARAPL1	AC012555.1	0.474204091	1.43E-31	postive
WIPI2	AC012555.1	0.404089643	1.37E-22	postive
LPIN1	AC012555.1	0.412578997	1.44E-23	postive
JDP2	AL049871.1	0.402035089	2.35E-22	postive
HIC1	AL049871.1	0.428795299	1.62E-25	postive
ZEB1	AL049871.1	0.58288041	2.30E-50	postive
EPAS1	AL049871.1	0.505063044	3.10E-36	postive
HELLS	AC011978.1	0.435750763	2.19E-26	postive
TFAP2C	AC011978.1	0.521739651	5.83E-39	postive
NOX1	AC011978.1	0.462056255	7.33E-30	postive
ISCU	LINC00706	0.552386874	2.21E-44	postive
TMBIM4	LINC00706	0.484277822	4.84E-33	postive
CHMP5	LINC00706	0.419091894	2.45E-24	postive
DDIT3	LINC00706	0.404653768	1.18E-22	postive
GOT1	LINC00706	0.40416007	1.35E-22	postive
MAP1LC3A	LINC00706	0.408154546	4.70E-23	postive
GABARAPL2	LINC00706	0.44086651	4.87E-27	postive
TSC22D3	LINC01704	0.407947643	4.97E-23	postive
ZEB1	LINC01704	0.504155566	4.32E-36	postive
EPAS1	LINC01704	0.547001237	2.17E-43	postive
HELLS	AC009996.1	0.536800842	1.48E-41	postive

TUBE1	AC009996.1	0.409061341	3.70E-23	postive
ALOX12	AC009996.1	0.486655145	2.14E-33	postive
GABPB1	AC009996.1	0.510094215	4.84E-37	postive
LINC00472	AC009996.1	0.658599887	2.51E-68	postive
ATM	AC009996.1	0.560195259	7.42E-46	postive
FBXW7	AC009996.1	0.472982348	2.14E-31	postive
HELLS	AC010245.2	0.434602781	3.05E-26	postive
ZNF419	AC010245.2	0.525020208	1.63E-39	postive
TUBE1	AC010245.2	0.59068792	5.32E-52	postive
ALOX12	AC010245.2	0.490306498	6.04E-34	postive
GABPB1	AC010245.2	0.488118422	1.29E-33	postive
PHKG2	AC010245.2	0.429536684	1.31E-25	postive
ATM	AC010245.2	0.451601581	1.92E-28	postive
TAZ	AC010245.2	0.555814096	5.03E-45	postive
FBXW7	AC010245.2	0.476534702	6.60E-32	postive
HELLS	AL358072.1	0.417246151	4.06E-24	postive
ZNF419	AL358072.1	0.534432507	3.87E-41	postive
TUBE1	AL358072.1	0.553923399	1.14E-44	postive
ALOX12	AL358072.1	0.46263316	6.10E-30	postive
GABPB1	AL358072.1	0.521884819	5.51E-39	postive
PHKG2	AL358072.1	0.457004454	3.60E-29	postive
LINC00472	AL358072.1	0.507147195	1.44E-36	postive
ATM	AL358072.1	0.452308234	1.54E-28	postive
TAZ	AL358072.1	0.513881812	1.17E-37	postive
FBXW7	AL358072.1	0.438423242	1.00E-26	postive
TUBE1	GK-AS1	0.555569216	5.60E-45	postive
ALOX12	GK-AS1	0.582709173	2.49E-50	postive
LINC00472	GK-AS1	0.417940289	3.36E-24	postive
ATM	GK-AS1	0.520282565	1.02E-38	postive
HELLS	RASGRP3-AS	0.403293479	1.69E-22	postive
ZNF419	RASGRP3-AS	0.413141731	1.24E-23	postive
TUBE1	RASGRP3-AS	0.505734297	2.42E-36	postive
ALOX12	RASGRP3-AS	0.43374243	3.92E-26	postive
PHKG2	RASGRP3-AS	0.507492191	1.27E-36	postive
LINC00472	RASGRP3-AS	0.490671051	5.32E-34	postive
TAZ	RASGRP3-AS	0.518971119	1.69E-38	postive
FBXW7	RASGRP3-AS	0.408675034	4.10E-23	postive
HELLS	AL157392.3	0.51833466	2.16E-38	postive
ZNF419	AL157392.3	0.544516258	6.16E-43	postive
VEGFA	AL157392.3	0.406729113	6.86E-23	postive
TUBE1	AL157392.3	0.627451992	2.46E-60	postive
SETD1B	AL157392.3	0.429015518	1.52E-25	postive
ALOX12	AL157392.3	0.666679018	1.47E-70	postive
GABPB1	AL157392.3	0.616996527	7.47E-58	postive
ZEB1	AL157392.3	0.409675499	3.14E-23	postive
MAPK8	AL157392.3	0.51262677	1.88E-37	postive
LINC00472	AL157392.3	0.623397664	2.31E-59	postive
ATM	AL157392.3	0.768781489	2.46E-106	postive
FBXW7	AL157392.3	0.579216773	1.30E-49	postive
LINC00472	AL137024.1	0.443121487	2.49E-27	postive
FBXW7	AL137024.1	0.401313928	2.83E-22	postive
GPX4	ATXN2-AS	0.426955547	2.73E-25	postive
HSF1	ATXN2-AS	0.451229829	2.15E-28	postive
PHKG2	ATXN2-AS	0.43027426	1.06E-25	postive
KLHL24	AC005753.1	0.4658808	2.16E-30	postive
GABPB1	AC005753.1	0.431411365	7.66E-26	postive
LINC00472	AC005753.1	0.793943316	3.89E-118	postive
MUC1	ZNF350-AS1	0.425246302	4.41E-25	postive

JDP2	LINC01537	0.686865013	1.88E-76	postive
EIF2S1	LINC01537	0.482572505	8.65E-33	postive
ZEB1	LINC01537	0.489087085	9.23E-34	postive
EPAS1	LINC01537	0.513159503	1.54E-37	postive
SLC2A6	AC027575.2	0.423110453	8.03E-25	postive
IREB2	AC027575.2	-0.424419795	5.57E-25	negative
NOX1	AC027575.2	0.420700225	1.57E-24	postive
PIK3CA	AC027575.2	-0.423937095	6.37E-25	negative
LPCAT3	AC027575.2	-0.412682588	1.40E-23	negative
HRAS	AC027575.2	0.421283878	1.33E-24	postive
NCOA4	AC027575.2	-0.476871486	5.90E-32	negative
PHKG2	AC027575.2	0.485818004	2.85E-33	postive
BECN1	AC027575.2	-0.451521896	1.96E-28	negative
MAPK1	AC027575.2	-0.437229214	1.42E-26	negative
ANO6	AC027575.2	-0.408251568	4.59E-23	negative
TAZ	AC027575.2	0.487313996	1.70E-33	postive
MTDH	AC027575.2	-0.454981567	6.75E-29	negative
ATM	AC096541.1	0.434771531	2.91E-26	postive
ATM	AL357033.3	0.51337245	1.42E-37	postive
ZNF419	AC025162.2	0.437442433	1.33E-26	postive
ALOX12	AC025162.2	0.455755493	5.31E-29	postive
HBA1	AC025162.2	0.55343289	1.41E-44	postive
HRAS	AC025162.2	0.442206695	3.27E-27	postive
EGLN2	AC025162.2	0.55799648	1.95E-45	postive
TAZ	AC025162.2	0.475454545	9.45E-32	postive
ALOX12	AL512306.2	0.424155648	5.99E-25	postive
TFAP2C	AL512306.2	0.474025207	1.52E-31	postive
HBA1	AL512306.2	0.434460038	3.18E-26	postive
ZNF419	JPX	0.421739311	1.18E-24	postive
TUBE1	JPX	0.426661368	2.96E-25	postive
ZNF419	AC008760.1	0.487979033	1.35E-33	postive
VEGFA	AC008760.1	0.562850863	2.29E-46	postive
TUBE1	AC008760.1	0.608382251	7.08E-56	postive
SETD1B	AC008760.1	0.454353541	8.20E-29	postive
ALOX12	AC008760.1	0.598886321	9.13E-54	postive
PHKG2	AC008760.1	0.427909957	2.08E-25	postive
TAZ	AC008760.1	0.61967539	1.76E-58	postive
HELLS	AC093157.2	0.585659767	6.09E-51	postive
ZNF419	AC093157.2	0.419014748	2.50E-24	postive
TUBE1	AC093157.2	0.521148405	7.32E-39	postive
ALOX12	AC093157.2	0.488595372	1.09E-33	postive
GABPB1	AC093157.2	0.620033555	1.45E-58	postive
ATG7	AC093157.2	0.425206215	4.46E-25	postive
MAPK8	AC093157.2	0.406124099	8.05E-23	postive
LINC00472	AC093157.2	0.669446618	2.44E-71	postive
ATM	AC093157.2	0.722841598	3.03E-88	postive
FBXW7	AC093157.2	0.543463337	9.55E-43	postive
ZNF419	AL136295.2	0.472455759	2.54E-31	postive
TUBE1	AL136295.2	0.452788103	1.33E-28	postive
ALOX12	AL136295.2	0.546709497	2.46E-43	postive
PHKG2	AL136295.2	0.445001992	1.42E-27	postive
TAZ	AL136295.2	0.590978937	4.62E-52	postive
HSF1	ZNNT1	0.424728869	5.10E-25	postive
PHKG2	ZNNT1	0.471692663	3.27E-31	postive
TAZ	ZNNT1	0.495176711	1.09E-34	postive
ENPP2	KCNJ2-AS1	0.52167601	5.97E-39	postive
SLC1A4	KCNJ2-AS1	0.46240194	6.56E-30	postive
VEGFA	KCNJ2-AS1	0.418745548	2.69E-24	postive

ZEB1	KCNJ2-AS1	0.514396082	9.65E-38	postive
EPAS1	KCNJ2-AS1	0.405341978	9.89E-23	postive
ISCU	POLR2J4	0.443758477	2.06E-27	postive
PHKG2	POLR2J4	0.433813539	3.84E-26	postive
ATG4D	POLR2J4	0.553103897	1.62E-44	postive
MAP1LC3A	POLR2J4	0.572395207	3.08E-48	postive
TAZ	POLR2J4	0.420707808	1.57E-24	postive
FANCD2	LINC02446	0.403678584	1.53E-22	postive
STMN1	LINC02446	0.599655647	6.19E-54	postive
RRM2	LINC02446	0.547962959	1.45E-43	postive
AURKA	LINC02446	0.505422101	2.72E-36	postive
CDKN2A	LINC02446	0.507301079	1.36E-36	postive
IFNG	LINC02446	0.492511197	2.79E-34	postive
BRD4	CIRBP-AS1	0.441199938	4.41E-27	postive
SETD1B	CIRBP-AS1	0.419588265	2.13E-24	postive
ALOX12	CIRBP-AS1	0.429907219	1.18E-25	postive
PHKG2	CIRBP-AS1	0.429906036	1.18E-25	postive
TAZ	CIRBP-AS1	0.488192811	1.26E-33	postive
GPX4	ILRUN-AS1	0.469272149	7.20E-31	postive
STAT3	ILRUN-AS1	-0.427652519	2.24E-25	negative
OTUB1	ILRUN-AS1	0.423285956	7.64E-25	postive
SP1	ILRUN-AS1	-0.462665612	6.04E-30	negative
NOX1	ILRUN-AS1	0.405986554	8.35E-23	postive
HRAS	ILRUN-AS1	0.415493447	6.55E-24	postive
PHKG2	ILRUN-AS1	0.423335293	7.54E-25	postive
MAPK1	ILRUN-AS1	-0.407116654	6.19E-23	negative
ANO6	ILRUN-AS1	-0.492893689	2.44E-34	negative
SIRT1	ILRUN-AS1	-0.401930719	2.41E-22	negative
BACH1	ILRUN-AS1	-0.411683942	1.83E-23	negative
GPX4	OVOL1-AS1	0.417356776	3.94E-24	postive
ZNF419	SLFNL1-AS1	0.433869339	3.78E-26	postive
TUBE1	SLFNL1-AS1	0.465465684	2.47E-30	postive
ALOX12	SLFNL1-AS1	0.588726879	1.38E-51	postive
GABPB1	SLFNL1-AS1	0.4091425	3.62E-23	postive
ATM	SLFNL1-AS1	0.476826061	5.99E-32	postive
FBXW7	SLFNL1-AS1	0.405398828	9.74E-23	postive
HELLS	AC104564.1	0.549651102	7.09E-44	postive
TUBE1	AC104564.1	0.403010991	1.82E-22	postive
ALOX12	AC104564.1	0.539795747	4.35E-42	postive
GABPB1	AC104564.1	0.474854969	1.15E-31	postive
LINC00472	AC104564.1	0.588499601	1.55E-51	postive
ATM	AC104564.1	0.618842788	2.77E-58	postive
FBXW7	AC104564.1	0.436163997	1.94E-26	postive
ZNF419	IBA57-DT	0.50370042	5.10E-36	postive
VEGFA	IBA57-DT	0.411560176	1.90E-23	postive
TUBE1	IBA57-DT	0.416883714	4.48E-24	postive
ALOX12	IBA57-DT	0.571195559	5.34E-48	postive
PHKG2	IBA57-DT	0.410312679	2.65E-23	postive
TAZ	IBA57-DT	0.537510745	1.11E-41	postive
HELLS	ACAP2-IT1	0.505606504	2.54E-36	postive
KLHL24	ACAP2-IT1	0.543198524	1.07E-42	postive
TUBE1	ACAP2-IT1	0.461418631	8.97E-30	postive
IREB2	ACAP2-IT1	0.492657047	2.65E-34	postive
GABPB1	ACAP2-IT1	0.601486439	2.45E-54	postive
PIK3CA	ACAP2-IT1	0.509585055	5.85E-37	postive
KRAS	ACAP2-IT1	0.430116268	1.11E-25	postive
ATG7	ACAP2-IT1	0.434637051	3.02E-26	postive
MAPK8	ACAP2-IT1	0.476461491	6.76E-32	postive

LINC00472	ACAP2-IT1	0.879232406	4.57E-175	postive
PRKAA1	ACAP2-IT1	0.418906643	2.57E-24	postive
TGFBR1	ACAP2-IT1	0.41782982	3.46E-24	postive
ATM	ACAP2-IT1	0.707688388	4.65E-83	postive
FBXW7	ACAP2-IT1	0.482750881	8.14E-33	postive
TUBE1	AP000254.2	0.438331537	1.03E-26	postive
NOX1	AP000254.2	0.534486776	3.78E-41	postive
NCOA4	AP000254.2	-0.419505044	2.18E-24	negative
PHKG2	AP000254.2	0.531929998	1.06E-40	postive
BECN1	AP000254.2	-0.496454734	6.92E-35	negative
TAZ	AP000254.2	0.593800958	1.15E-52	postive
ZNF419	AC092119.2	0.522248409	4.79E-39	postive
TUBE1	AC092119.2	0.541122376	2.52E-42	postive
ALOX12	AC092119.2	0.5939018	1.10E-52	postive
PHKG2	AC092119.2	0.612397655	8.64E-57	postive
TAZ	AC092119.2	0.653477507	6.00E-67	postive
TRIB3	COSMOC	0.465293089	2.61E-30	postive
BID	COSMOC	0.442656669	2.86E-27	postive
HMGB1	AC125257.1	0.436937027	1.55E-26	postive
GABPB1	AC125257.1	0.457567936	3.02E-29	postive
NOX1	AC125257.1	0.450040782	3.09E-28	postive
TFAP2C	AC051619.6	0.517942217	2.51E-38	postive
HBA1	AC051619.6	0.564324194	1.19E-46	postive
FANCD2	AC004585.1	0.400163968	3.81E-22	postive
PML	AC004585.1	0.443520444	2.21E-27	postive
GCH1	AC004585.1	0.520567139	9.16E-39	postive
SLC2A6	AC004585.1	0.404806895	1.14E-22	postive
IFNG	AC004585.1	0.719659199	3.97E-87	postive
IREB2	LINC01801	0.469927991	5.82E-31	postive
SP1	LINC01801	0.533471952	5.69E-41	postive
PHKG2	LINC01801	-0.401770937	2.51E-22	negative
MAPK1	LINC01801	0.403841304	1.47E-22	postive
ZEB1	LINC01801	0.439387243	7.54E-27	postive
PRKAA2	LINC01801	0.431429369	7.62E-26	postive
EPAS1	LINC01801	0.458142644	2.52E-29	postive
SIRT1	LINC01801	0.534930137	3.16E-41	postive
FANCD2	AC106028.3	0.523911654	2.51E-39	postive
HELLS	AC106028.3	0.456803166	3.83E-29	postive
ZNF419	AC106028.3	0.426379168	3.21E-25	postive
ALOX12	AC106028.3	0.447014551	7.74E-28	postive
TFAP2C	AC106028.3	0.412809634	1.35E-23	postive
TAZ	AC106028.3	0.459487899	1.65E-29	postive
SLC3A2	AC016866.1	0.40642231	7.44E-23	postive
ISCU	AC016866.1	0.479156826	2.74E-32	postive
DDIT3	AC016866.1	0.403980873	1.41E-22	postive
ATG4D	AC016866.1	0.46820874	1.02E-30	postive
MAP1LC3A	AC016866.1	0.40277342	1.94E-22	postive
GABARAPL1	AC016866.1	0.496862367	5.99E-35	postive
LPIN1	AC016866.1	0.42023557	1.78E-24	postive
HELLS	AC109460.2	0.47829157	3.67E-32	postive
BRD4	AC109460.2	0.436643116	1.69E-26	postive
ZNF419	AC109460.2	0.601967427	1.92E-54	postive
VEGFA	AC109460.2	0.510177048	4.69E-37	postive
TUBE1	AC109460.2	0.668934514	3.41E-71	postive
SETD1B	AC109460.2	0.56870499	1.66E-47	postive
ALOX12	AC109460.2	0.792200173	2.88E-117	postive
GABPB1	AC109460.2	0.477335996	5.05E-32	postive
ATM	AC109460.2	0.607320642	1.23E-55	postive

YY1AP1	AC109460.2	0.442451958	3.04E-27	postive
TAZ	AC109460.2	0.460789361	1.09E-29	postive
FBXW7	AC109460.2	0.491117782	4.55E-34	postive
VEGFA	TBC1D8-AS1	0.426818141	2.83E-25	postive
ALOX12	TBC1D8-AS1	0.500996102	1.36E-35	postive
ATM	TBC1D8-AS1	0.448926716	4.34E-28	postive
YY1AP1	TBC1D8-AS1	0.422536461	9.42E-25	postive
ZNF419	AC092375.2	0.516435241	4.45E-38	postive
VEGFA	AC092375.2	0.435744686	2.19E-26	postive
TUBE1	AC092375.2	0.57528735	8.13E-49	postive
SETD1B	AC092375.2	0.40929343	3.48E-23	postive
ALOX12	AC092375.2	0.565757648	6.27E-47	postive
GABPB1	AC092375.2	0.448202225	5.40E-28	postive
LINC00472	AC092375.2	0.400571535	3.43E-22	postive
ATM	AC092375.2	0.504099056	4.41E-36	postive
TAZ	AC092375.2	0.446566999	8.86E-28	postive
FBXW7	AC092375.2	0.424117958	6.06E-25	postive
ATM	FP671120.6	0.405168523	1.04E-22	postive
HELLS	AC007878.1	0.523236623	3.26E-39	postive
KLHL24	AC007878.1	0.415466004	6.60E-24	postive
TUBE1	AC007878.1	0.44038085	5.62E-27	postive
IREB2	AC007878.1	0.432309978	5.92E-26	postive
GABPB1	AC007878.1	0.573645866	1.74E-48	postive
PIK3CA	AC007878.1	0.426985462	2.70E-25	postive
ATG7	AC007878.1	0.444802132	1.51E-27	postive
MAPK8	AC007878.1	0.448475602	4.97E-28	postive
LINC00472	AC007878.1	0.789303167	7.71E-116	postive
ATM	AC007878.1	0.740790615	7.45E-95	postive
FBXW7	AC007878.1	0.479909862	2.13E-32	postive
STEAP3	LNCSRLR	0.413267051	1.20E-23	postive
CYBB	LNCSRLR	0.409287609	3.48E-23	postive
ANO6	LNCSRLR	0.430245492	1.07E-25	postive
ATM	LNCSRLR	0.402113478	2.30E-22	postive
MTDH	LNCSRLR	0.468640855	8.84E-31	postive
BACH1	LNCSRLR	0.590488826	5.87E-52	postive
MAPK9	LINCMD1	0.405956263	8.41E-23	postive
GPX4	RAP2C-AS1	-0.415128371	7.23E-24	negative
RB1	RAP2C-AS1	0.452673033	1.38E-28	postive
HSPB1	RAP2C-AS1	-0.435063271	2.67E-26	negative
GCLC	RAP2C-AS1	0.488630368	1.08E-33	postive
MTOR	RAP2C-AS1	0.541898256	1.83E-42	postive
ACSL3	RAP2C-AS1	0.467185661	1.42E-30	postive
OTUB1	RAP2C-AS1	-0.423593267	7.02E-25	negative
HIF1A	RAP2C-AS1	0.408640715	4.13E-23	postive
OXSR1	RAP2C-AS1	0.474249559	1.41E-31	postive
KLHL24	RAP2C-AS1	0.548733131	1.05E-43	postive
ZFP69B	RAP2C-AS1	0.435855913	2.12E-26	postive
EIF2S1	RAP2C-AS1	0.508200023	9.77E-37	postive
MAP3K5	RAP2C-AS1	0.505488559	2.65E-36	postive
MAPK14	RAP2C-AS1	0.418197429	3.13E-24	postive
EIF2AK4	RAP2C-AS1	0.484891572	3.92E-33	postive
IREB2	RAP2C-AS1	0.743092709	9.65E-96	postive
SP1	RAP2C-AS1	0.421464759	1.27E-24	postive
GABPB1	RAP2C-AS1	0.556055444	4.53E-45	postive
PIK3CA	RAP2C-AS1	0.6449905	1.01E-64	postive
KRAS	RAP2C-AS1	0.620837562	9.38E-59	postive
HRAS	RAP2C-AS1	-0.423687929	6.83E-25	negative
ULK2	RAP2C-AS1	0.40765618	5.37E-23	postive

MAPK1	RAP2C-AS1	0.420545633	1.64E-24	postive
ZEB1	RAP2C-AS1	0.457231582	3.35E-29	postive
MAPK8	RAP2C-AS1	0.688094732	7.93E-77	postive
MAPK9	RAP2C-AS1	0.427702168	2.21E-25	postive
LINC00472	RAP2C-AS1	0.566460793	4.57E-47	postive
PRKAA2	RAP2C-AS1	0.584707283	9.61E-51	postive
PRKAA1	RAP2C-AS1	0.602327385	1.60E-54	postive
TGFBR1	RAP2C-AS1	0.401675126	2.58E-22	postive
TLR4	RAP2C-AS1	0.474942544	1.12E-31	postive
ATM	RAP2C-AS1	0.63861005	4.30E-63	postive
SIRT1	RAP2C-AS1	0.535306432	2.72E-41	postive
TUBE1	AC105206.2	0.45554584	5.67E-29	postive
SETD1B	AC105206.2	0.448873465	4.41E-28	postive
ALOX12	AC105206.2	0.520716805	8.65E-39	postive
ULK2	AC105206.2	0.401716699	2.55E-22	postive
ATM	AC105206.2	0.570857029	6.24E-48	postive
HELLS	AC145423.2	0.444100119	1.86E-27	postive
ZNF419	AC145423.2	0.482946099	7.62E-33	postive
TUBE1	AC145423.2	0.433119722	4.69E-26	postive
ALOX12	AC145423.2	0.608478181	6.74E-56	postive
DUOX1	AC145423.2	0.419048976	2.47E-24	postive
FBXW7	AC145423.2	0.400296138	3.68E-22	postive
TAZ	MIR503HG	0.56171371	3.80E-46	postive
CD44	AL133330.1	0.574960156	9.46E-49	postive
SOCS1	AL133330.1	0.548109728	1.36E-43	postive
TUBE1	AC016737.2	0.410494019	2.52E-23	postive
ALOX12	AC016737.2	0.401675745	2.58E-22	postive
LINC00472	AC016737.2	0.692743564	2.92E-78	postive
ATM	AC016737.2	0.407589323	5.47E-23	postive
ZNF419	AC135178.2	0.470347201	5.08E-31	postive
VEGFA	AC135178.2	0.55498051	7.22E-45	postive
TUBE1	AC135178.2	0.462457367	6.45E-30	postive
SETD1B	AC135178.2	0.431556126	7.35E-26	postive
ALOX12	AC135178.2	0.471883655	3.07E-31	postive
ZEB1	AC135178.2	0.422689397	9.03E-25	postive
TAZ	AC135178.2	0.429485817	1.33E-25	postive
ZNF419	AC078883.1	0.410540182	2.49E-23	postive
KLHL24	AC078883.1	0.41646289	5.03E-24	postive
TUBE1	AC078883.1	0.436854376	1.59E-26	postive
GABPB1	AC078883.1	0.518066484	2.39E-38	postive
MAPK8	AC078883.1	0.418194898	3.13E-24	postive
LINC00472	AC078883.1	0.679099317	3.96E-74	postive
ATM	AC078883.1	0.499069284	2.72E-35	postive
FBXW7	AC078883.1	0.508527866	8.65E-37	postive
VEGFA	AC007292.1	0.480296245	1.87E-32	postive
TUBE1	AC007292.1	0.437655703	1.25E-26	postive
DRD4	AC007292.1	0.4485533	4.86E-28	postive
ALOX12	AC007292.1	0.565680753	6.49E-47	postive
PHKG2	AC007292.1	0.476145158	7.51E-32	postive
TAZ	AC007292.1	0.642347784	4.84E-64	postive
PHKG2	AL137127.1	0.555916298	4.81E-45	postive
TAZ	AL137127.1	0.507017075	1.51E-36	postive
FANCD2	APCDD1L-D	0.470259246	5.22E-31	postive
STMN1	APCDD1L-D	0.736563962	3.00E-93	postive
RRM2	APCDD1L-D	0.559172571	1.16E-45	postive
AURKA	APCDD1L-D	0.569155158	1.35E-47	postive
CDKN2A	APCDD1L-D	0.789411651	6.82E-116	postive
ISCU	AL451069.1	0.402416954	2.13E-22	postive

DDIT3	AL451069.1	0.424890359	4.88E-25	postive
GABARAPL1	AL451069.1	0.446637355	8.68E-28	postive
ALOX12	AC073316.2	0.442318956	3.16E-27	postive
HBA1	AP007216.2	0.532057738	1.00E-40	postive
HIC1	AP007216.2	0.427560696	2.30E-25	postive
HRAS	AP007216.2	0.574237271	1.32E-48	postive
EGLN2	AP007216.2	0.646640437	3.78E-65	postive
ALOX12	AC129492.1	0.403850733	1.46E-22	postive
ALOX12B	AC129492.1	0.679797018	2.46E-74	postive
HELLS	AL161725.2	0.484140564	5.07E-33	postive
ZNF419	AL161725.2	0.406810521	6.72E-23	postive
TUBE1	AL161725.2	0.557095027	2.88E-45	postive
ALOX12	AL161725.2	0.713936896	3.71E-85	postive
ATM	AL161725.2	0.576974045	3.71E-49	postive
ZNF419	AC132938.1	0.400072038	3.90E-22	postive
TUBE1	AC132938.1	0.409342688	3.43E-23	postive
DRD4	AC132938.1	0.418198974	3.13E-24	postive
ALOX12	AC132938.1	0.471371424	3.63E-31	postive
HBA1	AC132938.1	0.405537163	9.39E-23	postive
TAZ	AC132938.1	0.484623816	4.30E-33	postive
TFAP2C	SPON1-AS1	0.552205577	2.38E-44	postive
HBA1	SPON1-AS1	0.506286029	1.98E-36	postive
DUOX1	SPON1-AS1	0.40915213	3.61E-23	postive
ZNF419	AC004846.2	0.418536029	2.85E-24	postive
TUBE1	AC004846.2	0.477823823	4.29E-32	postive
ALOX12	AC004846.2	0.581041319	5.50E-50	postive
PHKG2	AC004846.2	0.470821947	4.35E-31	postive
TAZ	AC004846.2	0.584103093	1.28E-50	postive
HELLS	AC016586.1	0.513375984	1.42E-37	postive
ZNF419	AC016586.1	0.46048909	1.20E-29	postive
TUBE1	AC016586.1	0.50834365	9.26E-37	postive
ALOX12	AC016586.1	0.533788996	5.01E-41	postive
GABPB1	AC016586.1	0.582421864	2.86E-50	postive
ATG7	AC016586.1	0.428214211	1.91E-25	postive
MAPK8	AC016586.1	0.44812747	5.53E-28	postive
LINC00472	AC016586.1	0.632303639	1.61E-61	postive
ATM	AC016586.1	0.751441296	4.84E-99	postive
FBXW7	AC016586.1	0.529596199	2.68E-40	postive
TFAP2C	AD001527.1	0.566257545	5.01E-47	postive
HBA1	AD001527.1	0.512966985	1.65E-37	postive
DUOX1	AD001527.1	0.41847544	2.90E-24	postive
AKR1C1	CTD-2297D1	0.424647801	5.22E-25	postive
AKR1C2	CTD-2297D1	0.450293651	2.86E-28	postive
ENPP2	AC007743.1	0.413434995	1.14E-23	postive
VEGFA	AC007743.1	0.404925945	1.10E-22	postive
FTL	AC007743.1	-0.433321925	4.42E-26	negative
IREB2	AC007743.1	0.405707946	8.98E-23	postive
CAPG	AC007743.1	-0.431631055	7.20E-26	negative
PRDX1	AC007743.1	-0.418266985	3.07E-24	negative
ZEB1	AC007743.1	0.616960578	7.62E-58	postive
MAPK8	AC007743.1	0.520751043	8.54E-39	postive
PRKAA1	AC007743.1	0.416520276	4.95E-24	postive
EPAS1	AC007743.1	0.627546682	2.33E-60	postive
SIRT1	AC007743.1	0.480120562	1.98E-32	postive
CHAC1	AC018638.6	0.668977835	3.31E-71	postive
DDIT3	AC018638.6	0.407568554	5.50E-23	postive
PHKG2	AC018638.6	0.437347335	1.37E-26	postive
MAP1LC3A	AC018638.6	0.49595473	8.27E-35	postive

TAZ	AC018638.6	0.442160745	3.32E-27	postive
HELLS	AC010186.3	0.61795167	4.47E-58	postive
TUBE1	AC010186.3	0.423784819	6.65E-25	postive
ALOX12	AC010186.3	0.426925489	2.75E-25	postive
IREB2	AC010186.3	0.410231032	2.71E-23	postive
GABPB1	AC010186.3	0.589738212	8.46E-52	postive
ATG7	AC010186.3	0.512186812	2.21E-37	postive
MAPK8	AC010186.3	0.400074732	3.90E-22	postive
LINC00472	AC010186.3	0.64302248	3.25E-64	postive
ATM	AC010186.3	0.75007101	1.72E-98	postive
FBXW7	AC010186.3	0.532370263	8.86E-41	postive
BACH1	AC010186.3	0.431584046	7.29E-26	postive
GPX4	AC073896.4	0.449484503	3.66E-28	postive
RB1	AC073896.4	-0.436213226	1.91E-26	negative
HSPB1	AC073896.4	0.594508598	8.12E-53	postive
GCLC	AC073896.4	-0.429252245	1.42E-25	negative
SLC40A1	AC073896.4	-0.437776569	1.21E-26	negative
MTOR	AC073896.4	-0.400478362	3.51E-22	negative
LAMP2	AC073896.4	-0.414924448	7.64E-24	negative
CHMP5	AC073896.4	-0.405936916	8.46E-23	negative
EIF2S1	AC073896.4	-0.451683502	1.87E-28	negative
MAPK14	AC073896.4	-0.518755153	1.84E-38	negative
EIF2AK4	AC073896.4	-0.436299382	1.86E-26	negative
IREB2	AC073896.4	-0.534039012	4.53E-41	negative
HBA1	AC073896.4	0.416165935	5.45E-24	postive
NOX1	AC073896.4	0.442450013	3.04E-27	postive
PIK3CA	AC073896.4	-0.48415869	5.04E-33	negative
SCP2	AC073896.4	-0.415364666	6.78E-24	negative
NRAS	AC073896.4	-0.50757017	1.23E-36	negative
KRAS	AC073896.4	-0.405696678	9.01E-23	negative
HRAS	AC073896.4	0.746727162	3.66E-97	postive
ATG5	AC073896.4	-0.421756398	1.17E-24	negative
NCOA4	AC073896.4	-0.624812109	1.06E-59	negative
PHKG2	AC073896.4	0.650836272	3.01E-66	postive
BECN1	AC073896.4	-0.561821149	3.62E-46	negative
MAP1LC3A	AC073896.4	0.45484344	7.05E-29	postive
MAPK1	AC073896.4	-0.527977227	5.09E-40	negative
MAPK9	AC073896.4	-0.462350903	6.67E-30	negative
PRKAA2	AC073896.4	-0.401858073	2.46E-22	negative
PRKAA1	AC073896.4	-0.444114649	1.85E-27	negative
ANO6	AC073896.4	-0.445109687	1.37E-27	negative
TLR4	AC073896.4	-0.450807728	2.44E-28	negative
EGLN2	AC073896.4	0.653592835	5.59E-67	postive
TAZ	AC073896.4	0.608123405	8.10E-56	postive
MTDH	AC073896.4	-0.530695901	1.73E-40	negative
SIRT1	AC073896.4	-0.422381135	9.84E-25	negative
BACH1	AC073896.4	-0.417102518	4.22E-24	negative
ATF4	KMT2E-AS1	0.427362433	2.43E-25	postive
DRD4	KMT2E-AS1	0.404218309	1.33E-22	postive
HRAS	KMT2E-AS1	0.438855864	8.82E-27	postive
NCOA4	KMT2E-AS1	-0.438529646	9.70E-27	negative
PHKG2	KMT2E-AS1	0.645667149	6.77E-65	postive
TAZ	KMT2E-AS1	0.761062953	5.18E-103	postive
MTDH	KMT2E-AS1	-0.410968503	2.22E-23	negative
PHKG2	AL355802.3	0.488669148	1.07E-33	postive
TAZ	AL355802.3	0.456101928	4.77E-29	postive
FANCD2	AL356299.2	0.418157906	3.16E-24	postive
HELLS	AL356299.2	0.455819053	5.20E-29	postive

ZNF419	AL356299.2	0.424903127	4.86E-25	postive
ALOX12	AL356299.2	0.540281215	3.56E-42	postive
TFAP2C	AL356299.2	0.413294942	1.19E-23	postive
HELLS	RBMS3-AS3	0.48173262	1.15E-32	postive
GABPB1	RBMS3-AS3	0.534630781	3.57E-41	postive
ATG7	RBMS3-AS3	0.442062226	3.42E-27	postive
MAPK8	RBMS3-AS3	0.418670408	2.75E-24	postive
LINC00472	RBMS3-AS3	0.525410042	1.40E-39	postive
ATM	RBMS3-AS3	0.668088294	5.91E-71	postive
FBXW7	RBMS3-AS3	0.412490339	1.48E-23	postive
STAT3	AC022509.3	0.431867208	6.72E-26	postive
SP1	AC022509.3	0.469906336	5.86E-31	postive
ANO6	AC022509.3	0.479243353	2.66E-32	postive
HELLS	AC145207.8	0.462260474	6.87E-30	postive
ZNF419	AC145207.8	0.426715453	2.92E-25	postive
TUBE1	AC145207.8	0.43467044	2.99E-26	postive
MAFG	AC145207.8	0.50087441	1.42E-35	postive
ALOX12	AC145207.8	0.620487745	1.13E-58	postive
ATM	AC145207.8	0.447562727	6.56E-28	postive
FBXW7	AC145207.8	0.423409765	7.38E-25	postive
HELLS	AL592148.3	0.415588734	6.38E-24	postive
TUBE1	AL592148.3	0.425512854	4.10E-25	postive
GABPB1	AL592148.3	0.4770071	5.63E-32	postive
ATM	AL592148.3	0.475034892	1.09E-31	postive
FBXW7	AL592148.3	0.442470059	3.03E-27	postive
FANCD2	AC079322.1	0.400025259	3.95E-22	postive
ALOX12	AC079322.1	0.427750821	2.18E-25	postive
TFAP2C	AC079322.1	0.540255238	3.60E-42	postive
HBA1	AC079322.1	0.477506583	4.77E-32	postive
DUOX1	AC079322.1	0.430624914	9.60E-26	postive
RRM2	UST-AS2	0.454361953	8.18E-29	postive
AURKA	UST-AS2	0.473926113	1.57E-31	postive
KLHL24	AL021154.1	0.443365216	2.32E-27	postive
GABARAPL1	AL021154.1	0.464662037	3.19E-30	postive
LINC00472	AL021154.1	0.559914736	8.40E-46	postive
LPIN1	AL021154.1	0.507425158	1.30E-36	postive
ALOX12	FAM153CP	0.42511021	4.59E-25	postive
HELLS	MIRLET7A1H	0.495129766	1.11E-34	postive
KLHL24	MIRLET7A1H	0.409178727	3.58E-23	postive
TUBE1	MIRLET7A1H	0.461512567	8.70E-30	postive
IREB2	MIRLET7A1H	0.436027335	2.02E-26	postive
GABPB1	MIRLET7A1H	0.579745109	1.01E-49	postive
PIK3CA	MIRLET7A1H	0.427142176	2.59E-25	postive
ATG7	MIRLET7A1H	0.411534196	1.91E-23	postive
MAPK8	MIRLET7A1H	0.472106867	2.85E-31	postive
LINC00472	MIRLET7A1H	0.752382478	2.02E-99	postive
ATM	MIRLET7A1H	0.724652521	6.89E-89	postive
FBXW7	MIRLET7A1H	0.476444247	6.80E-32	postive
HELLS	ZNF346-IT1	0.518639331	1.92E-38	postive
ZNF419	ZNF346-IT1	0.441446562	4.10E-27	postive
TUBE1	ZNF346-IT1	0.466778401	1.62E-30	postive
ALOX12	ZNF346-IT1	0.529971577	2.31E-40	postive
GABPB1	ZNF346-IT1	0.53561064	2.40E-41	postive
MAPK8	ZNF346-IT1	0.41666453	4.76E-24	postive
LINC00472	ZNF346-IT1	0.732344524	1.12E-91	postive
ATM	ZNF346-IT1	0.585793052	5.71E-51	postive
FBXW7	ZNF346-IT1	0.468121232	1.05E-30	postive
ALOX12	AC005041.3	0.433182244	4.60E-26	postive

EGLN2	AC005041.3	0.481859333	1.10E-32	postive
TAZ	AC005041.3	0.400168663	3.81E-22	postive
TFAP2C	AC025048.4	0.58066587	6.56E-50	postive
HBA1	AC025048.4	0.512936015	1.67E-37	postive
DUOX1	AC025048.4	0.400373768	3.61E-22	postive
VEGFA	AC024337.2	0.586521878	4.02E-51	postive
TUBE1	AC024337.2	0.569619313	1.10E-47	postive
ALOX12	AC024337.2	0.468035471	1.08E-30	postive
TAZ	AC024337.2	0.591169977	4.20E-52	postive
TUBE1	AC024896.1	0.408782979	3.98E-23	postive
ALOX12	AC024896.1	0.40982188	3.02E-23	postive
HELLS	AC015813.1	0.457680281	2.91E-29	postive
ZNF419	AC015813.1	0.544606048	5.93E-43	postive
TUBE1	AC015813.1	0.536199776	1.89E-41	postive
ALOX12	AC015813.1	0.618336831	3.63E-58	postive
GABPB1	AC015813.1	0.435424389	2.40E-26	postive
ATM	AC015813.1	0.427663821	2.23E-25	postive
TAZ	AC015813.1	0.538009555	9.05E-42	postive
FBXW7	AC015813.1	0.443826086	2.02E-27	postive
HELLS	BCL2L1-AS1	0.493983682	1.66E-34	postive
KLHL24	BCL2L1-AS1	0.445872476	1.09E-27	postive
TUBE1	BCL2L1-AS1	0.408083689	4.79E-23	postive
IREB2	BCL2L1-AS1	0.41201412	1.68E-23	postive
GABPB1	BCL2L1-AS1	0.55071668	4.50E-44	postive
PIK3CA	BCL2L1-AS1	0.431738933	6.98E-26	postive
ATG7	BCL2L1-AS1	0.456335549	4.43E-29	postive
LINC00472	BCL2L1-AS1	0.815123781	1.99E-129	postive
ATM	BCL2L1-AS1	0.687327965	1.36E-76	postive
FBXW7	BCL2L1-AS1	0.431006502	8.61E-26	postive
HELLS	MALAT1	0.45017185	2.97E-28	postive
KLHL24	MALAT1	0.49358189	1.91E-34	postive
IREB2	MALAT1	0.417060256	4.27E-24	postive
GABPB1	MALAT1	0.543267123	1.04E-42	postive
PIK3CA	MALAT1	0.440674333	5.16E-27	postive
ATG7	MALAT1	0.409914999	2.94E-23	postive
MAPK8	MALAT1	0.402681744	1.98E-22	postive
LINC00472	MALAT1	0.882000234	1.34E-177	postive
ATM	MALAT1	0.654041011	4.24E-67	postive
FBXW7	MALAT1	0.438009524	1.13E-26	postive
ISCU	LINC01594	0.480807104	1.57E-32	postive
LINC00472	LINC01594	0.435859724	2.12E-26	postive
LPIN1	LINC01594	0.447281349	7.14E-28	postive
HELLS	AC012593.2	0.484547404	4.41E-33	postive
ZNF419	AC012593.2	0.410742393	2.36E-23	postive
TUBE1	AC012593.2	0.528293013	4.49E-40	postive
ALOX12	AC012593.2	0.611688102	1.26E-56	postive
GABPB1	AC012593.2	0.485419156	3.27E-33	postive
ATM	AC012593.2	0.686330875	2.73E-76	postive
FBXW7	AC012593.2	0.455870927	5.12E-29	postive
ALOX12	AL162431.1	0.465560313	2.39E-30	postive
TAZ	AL162431.1	0.506908315	1.57E-36	postive
TFAP2C	AC005332.1	0.573226097	2.11E-48	postive
HBA1	AC005332.1	0.524333491	2.13E-39	postive
DUOX1	AC005332.1	0.412860095	1.34E-23	postive
TUBE1	AC105105.1	0.535426237	2.59E-41	postive
ALOX12	AC105105.1	0.61655049	9.49E-58	postive
ATM	AC105105.1	0.54461879	5.90E-43	postive
TAZ	AC105105.1	0.453920802	9.38E-29	postive

HELLS	AC068792.1	0.434211285	3.42E-26	postive
GABPB1	AC068792.1	0.403678469	1.53E-22	postive
LINC00472	AC068792.1	0.625575939	6.96E-60	postive
FBXW7	AC068792.1	0.400490534	3.50E-22	postive
HRAS	AC026803.2	0.510640915	3.95E-37	postive
EGLN2	AC026803.2	0.564464405	1.12E-46	postive
LINC00472	AC110609.1	0.410414372	2.58E-23	postive
ATM	AC110609.1	0.453713552	1.00E-28	postive
ZNF419	LINC00653	0.503584807	5.32E-36	postive
TUBE1	LINC00653	0.490297868	6.05E-34	postive
ALOX12	LINC00653	0.545900057	3.45E-43	postive
PHKG2	LINC00653	0.583081414	2.09E-50	postive
TAZ	LINC00653	0.682541062	3.77E-75	postive
HELLS	AC019080.4	0.454779931	7.19E-29	postive
LINC00472	AC019080.4	0.429757212	1.23E-25	postive
ATM	AC019080.4	0.422582923	9.30E-25	postive
PHKG2	SNHG12	0.405209666	1.02E-22	postive
SAT1	SNHG12	0.426362152	3.22E-25	postive
TAZ	SNHG12	0.487360041	1.68E-33	postive
ACO1	ST20-AS1	0.446386295	9.36E-28	postive
WIPI1	ST20-AS1	0.477654199	4.54E-32	postive
HELLS	AC008735.1	0.421194118	1.37E-24	postive
ZNF419	AC008735.1	0.533511734	5.60E-41	postive
VEGFA	AC008735.1	0.409499782	3.29E-23	postive
TUBE1	AC008735.1	0.549411789	7.85E-44	postive
SETD1B	AC008735.1	0.500746206	1.49E-35	postive
ALOX12	AC008735.1	0.70987641	8.70E-84	postive
ATM	AC008735.1	0.444499138	1.65E-27	postive
TAZ	AC008735.1	0.408593228	4.19E-23	postive
FBXW7	AC008735.1	0.400709148	3.31E-22	postive
BRD4	AL592211.1	0.410950931	2.23E-23	postive
ZNF419	AL592211.1	0.49874898	3.05E-35	postive
TUBE1	AL592211.1	0.475544471	9.17E-32	postive
ALOX12	AL592211.1	0.487012442	1.89E-33	postive
PHKG2	AL592211.1	0.546817204	2.35E-43	postive
TAZ	AL592211.1	0.610647602	2.17E-56	postive
STMN1	AC079949.1	0.614717359	2.53E-57	postive
RRM2	AC079949.1	0.400527593	3.47E-22	postive
CDKN2A	AC079949.1	0.677750807	9.85E-74	postive
MUC1	AL121820.2	0.472757052	2.30E-31	postive
ZNF419	AC010973.2	0.429913925	1.18E-25	postive
DRD4	AC010973.2	0.416093445	5.56E-24	postive
NCOA4	AC010973.2	-0.467679835	1.21E-30	negative
PHKG2	AC010973.2	0.709521957	1.14E-83	postive
TAZ	AC010973.2	0.791337977	7.70E-117	postive
TFAP2C	LINC00471	0.540432546	3.35E-42	postive
HBA1	LINC00471	0.495108254	1.12E-34	postive
DUOX1	LINC00471	0.405356367	9.85E-23	postive
ZNF419	AL450384.2	0.454102502	8.86E-29	postive
TUBE1	AL450384.2	0.431367977	7.76E-26	postive
ALOX12	AL450384.2	0.533161761	6.45E-41	postive
TFAP2C	AL450384.2	0.425155238	4.53E-25	postive
TAZ	AL450384.2	0.414558224	8.44E-24	postive
RB1	AC107027.3	0.463142975	5.18E-30	postive
ACSL3	AC107027.3	0.424517189	5.42E-25	postive
OXSR1	AC107027.3	0.429563799	1.30E-25	postive
KLHL24	AC107027.3	0.44688696	8.05E-28	postive
MAPK14	AC107027.3	0.475593055	9.02E-32	postive

IREB2	AC107027.3	0.510634666	3.96E-37 postive
PIK3CA	AC107027.3	0.51999751	1.14E-38 postive
NRAS	AC107027.3	0.421244823	1.35E-24 postive
KRAS	AC107027.3	0.511263454	3.13E-37 postive
SLC38A1	AC107027.3	0.528534744	4.08E-40 postive
NCOA4	AC107027.3	0.442627203	2.89E-27 postive
SNX4	AC107027.3	0.506898314	1.58E-36 postive
MAPK1	AC107027.3	0.456403858	4.34E-29 postive
MAPK9	AC107027.3	0.451120189	2.22E-28 postive
PRKAA2	AC107027.3	0.492908093	2.43E-34 postive
PRKAA1	AC107027.3	0.457134608	3.45E-29 postive
TGFBR1	AC107027.3	0.435087014	2.65E-26 postive
ANO6	AC107027.3	0.484414944	4.62E-33 postive
MTDH	AC107027.3	0.442632917	2.88E-27 postive
BACH1	AC107027.3	0.445216288	1.33E-27 postive
FANCD2	AL157838.1	0.487097211	1.84E-33 postive
HELLS	AL157838.1	0.552292679	2.30E-44 postive
ZNF419	AL157838.1	0.437442285	1.33E-26 postive
TUBE1	AL157838.1	0.433925556	3.71E-26 postive
ALOX12	AL157838.1	0.539084097	5.83E-42 postive
GABPB1	AL157838.1	0.46496609	2.89E-30 postive
LINC00472	AL157838.1	0.55242873	2.17E-44 postive
ATM	AL157838.1	0.518293771	2.19E-38 postive
FBXW7	AL157838.1	0.448807517	4.50E-28 postive
ATP6V1G2	LINC01315	0.444541294	1.63E-27 postive
GCLC	PLBD1-AS1	0.46108195	9.98E-30 postive
MTOR	PLBD1-AS1	0.42632775	3.26E-25 postive
FH	PLBD1-AS1	0.436099463	1.98E-26 postive
ISCU	PLBD1-AS1	0.541341436	2.30E-42 postive
ACSL3	PLBD1-AS1	0.482567242	8.66E-33 postive
KLHL24	PLBD1-AS1	0.553495547	1.37E-44 postive
EIF2AK4	PLBD1-AS1	0.477444245	4.87E-32 postive
IREB2	PLBD1-AS1	0.450810692	2.44E-28 postive
CS	PLBD1-AS1	0.539088701	5.82E-42 postive
PIK3CA	PLBD1-AS1	0.449249307	3.93E-28 postive
GOT1	PLBD1-AS1	0.438809625	8.94E-27 postive
GABARAPL1	PLBD1-AS1	0.617282932	6.41E-58 postive
WIPI2	PLBD1-AS1	0.430727791	9.32E-26 postive
LPIN1	PLBD1-AS1	0.511544772	2.82E-37 postive
HSF1	MELTF-AS1	0.554290118	9.73E-45 postive
G6PD	MELTF-AS1	0.421608348	1.22E-24 postive
NCOA4	MELTF-AS1	-0.462326701	6.72E-30 negative
PHKG2	MELTF-AS1	0.585899033	5.43E-51 postive
ULK1	MELTF-AS1	0.480291255	1.87E-32 postive
BID	MELTF-AS1	0.461784899	7.98E-30 postive
TAZ	MELTF-AS1	0.549521673	7.49E-44 postive
ZNF419	MIR4453HG	0.569980067	9.30E-48 postive
TUBE1	MIR4453HG	0.560581518	6.26E-46 postive
SETD1B	MIR4453HG	0.428390922	1.82E-25 postive
ALOX12	MIR4453HG	0.493031484	2.32E-34 postive
GABPB1	MIR4453HG	0.595671277	4.56E-53 postive
MAPK8	MIR4453HG	0.45730335	3.28E-29 postive
LINC00472	MIR4453HG	0.419558343	2.15E-24 postive
LPIN1	MIR4453HG	0.442756036	2.78E-27 postive
ATM	MIR4453HG	0.467217647	1.40E-30 postive
FBXW7	MIR4453HG	0.418421086	2.94E-24 postive
LINC00472	AC012313.9	0.504915212	3.27E-36 postive
HELLS	GARS1-DT	0.51617299	4.92E-38 postive

ZNF419	GARS1-DT	0.523723279	2.70E-39	postive
TUBE1	GARS1-DT	0.662680536	1.91E-69	postive
ALOX12	GARS1-DT	0.647484645	2.28E-65	postive
GABPB1	GARS1-DT	0.531166066	1.43E-40	postive
LINC00472	GARS1-DT	0.480058336	2.03E-32	postive
ATM	GARS1-DT	0.657268405	5.76E-68	postive
TAZ	GARS1-DT	0.47485798	1.15E-31	postive
FBXW7	GARS1-DT	0.53209601	9.89E-41	postive
ZEB1	AF064858.2	0.442867922	2.69E-27	postive
EPAS1	AF064858.2	0.449068161	4.15E-28	postive
ZNF419	AL512791.1	0.546051014	3.24E-43	postive
VEGFA	AL512791.1	0.507614971	1.21E-36	postive
TUBE1	AL512791.1	0.602186219	1.72E-54	postive
SETD1B	AL512791.1	0.503488085	5.51E-36	postive
ALOX12	AL512791.1	0.644180649	1.64E-64	postive
GABPB1	AL512791.1	0.445106703	1.38E-27	postive
ZEB1	AL512791.1	0.401177803	2.93E-22	postive
MAPK8	AL512791.1	0.464289823	3.59E-30	postive
ATM	AL512791.1	0.550163252	5.70E-44	postive
YY1AP1	AL512791.1	0.407027587	6.34E-23	postive
FBXW7	AL512791.1	0.456888211	3.73E-29	postive
HELLS	AL137779.2	0.423026266	8.22E-25	postive
IREB2	AL137779.2	0.401471094	2.72E-22	postive
GABPB1	AL137779.2	0.435877353	2.11E-26	postive
LINC00472	AL137779.2	0.526439988	9.32E-40	postive
ATM	AL137779.2	0.43222237	6.07E-26	postive
TUBE1	ZDHHC20-IT	0.42223887	1.02E-24	postive
ALOX12	ZDHHC20-IT	0.401940046	2.41E-22	postive
GABPB1	ZDHHC20-IT	0.42896393	1.54E-25	postive
LINC00472	ZDHHC20-IT	0.734712186	1.48E-92	postive
ATM	ZDHHC20-IT	0.478671858	3.23E-32	postive
TAZ	PRKAG2-AS1	0.427649231	2.24E-25	postive
AGPAT3	AC010307.4	0.511706328	2.65E-37	postive
VEGFA	AC021016.2	0.441698314	3.81E-27	postive
ALOX12	AC021016.2	0.531827762	1.10E-40	postive
TAZ	AC021016.2	0.501717706	1.05E-35	postive
ATM	AC090912.1	0.475854762	8.27E-32	postive
FBXW7	AC090912.1	0.406780636	6.77E-23	postive
HELLS	AC010761.1	0.513317479	1.45E-37	postive
BRD4	AC010761.1	0.410181779	2.74E-23	postive
ZNF419	AC010761.1	0.428884724	1.58E-25	postive
VEGFA	AC010761.1	0.40475105	1.15E-22	postive
TUBE1	AC010761.1	0.496350328	7.19E-35	postive
ALOX12	AC010761.1	0.545318903	4.40E-43	postive
PHKG2	AC010761.1	0.431083741	8.42E-26	postive
TAZ	AC010761.1	0.567864732	2.43E-47	postive
FBXW7	AC010761.1	0.426420413	3.17E-25	postive
HELLS	AL157392.4	0.511981398	2.39E-37	postive
ZNF419	AL157392.4	0.441232578	4.37E-27	postive
TUBE1	AL157392.4	0.561349802	4.46E-46	postive
ALOX12	AL157392.4	0.552279585	2.31E-44	postive
GABPB1	AL157392.4	0.526995979	7.49E-40	postive
LINC00472	AL157392.4	0.536819197	1.47E-41	postive
ATM	AL157392.4	0.669334023	2.62E-71	postive
FBXW7	AL157392.4	0.505249868	2.89E-36	postive
ALOX12	AC079015.1	0.479864567	2.16E-32	postive
IFNG	AC079015.1	0.453666857	1.01E-28	postive
ATM	AC079015.1	0.42231512	1.00E-24	postive

VEGFA	C1RL-AS1	0.508859824	7.65E-37	postive
TUBE1	C1RL-AS1	0.557771387	2.15E-45	postive
SETD1B	C1RL-AS1	0.506809917	1.63E-36	postive
ALOX12	C1RL-AS1	0.711190232	3.15E-84	postive
SP1	C1RL-AS1	0.418197178	3.13E-24	postive
ATM	C1RL-AS1	0.406350289	7.58E-23	postive
YY1AP1	C1RL-AS1	0.402174132	2.26E-22	postive
TAZ	C1RL-AS1	0.453455869	1.08E-28	postive
TFAP2C	AC106738.1	0.486444752	2.30E-33	postive
HBA1	AC106738.1	0.57884544	1.55E-49	postive
TAZ	AC137834.2	0.479666334	2.31E-32	postive
HELLS	AC018690.1	0.50987733	5.24E-37	postive
ZNF419	AC018690.1	0.507588976	1.22E-36	postive
TUBE1	AC018690.1	0.56917739	1.34E-47	postive
ALOX12	AC018690.1	0.557503316	2.41E-45	postive
GABPB1	AC018690.1	0.514948281	7.83E-38	postive
ATM	AC018690.1	0.552942055	1.74E-44	postive
TAZ	AC018690.1	0.413816827	1.03E-23	postive
FBXW7	AC018690.1	0.52460062	1.92E-39	postive
FANCD2	AC092123.1	0.472172573	2.79E-31	postive
HELLS	AC092123.1	0.541468101	2.18E-42	postive
ZNF419	AC092123.1	0.518028205	2.43E-38	postive
TUBE1	AC092123.1	0.531128804	1.46E-40	postive
ALOX12	AC092123.1	0.725376619	3.80E-89	postive
GABPB1	AC092123.1	0.446785438	8.30E-28	postive
DUOX1	AC092123.1	0.419470327	2.20E-24	postive
ATM	AC092123.1	0.530938165	1.57E-40	postive
FBXW7	AC092123.1	0.44203196	3.45E-27	postive
HELLS	AL078459.1	0.460561296	1.18E-29	postive
TUBE1	AL078459.1	0.425544022	4.06E-25	postive
ALOX12	AL078459.1	0.414226061	9.24E-24	postive
GABPB1	AL078459.1	0.477538937	4.72E-32	postive
LINC00472	AL078459.1	0.476725139	6.19E-32	postive
ATM	AL078459.1	0.508187731	9.81E-37	postive
FBXW7	AL078459.1	0.458798634	2.05E-29	postive
HELLS	SOS1-IT1	0.500294441	1.75E-35	postive
KLHL24	SOS1-IT1	0.541883736	1.84E-42	postive
TUBE1	SOS1-IT1	0.481771498	1.14E-32	postive
IREB2	SOS1-IT1	0.51905041	1.64E-38	postive
GABPB1	SOS1-IT1	0.604592964	5.02E-55	postive
PIK3CA	SOS1-IT1	0.537820801	9.77E-42	postive
KRAS	SOS1-IT1	0.475209567	1.02E-31	postive
MAPK8	SOS1-IT1	0.521501019	6.39E-39	postive
LINC00472	SOS1-IT1	0.869505438	1.24E-166	postive
PRKAA1	SOS1-IT1	0.449056058	4.17E-28	postive
TGFBR1	SOS1-IT1	0.405907431	8.52E-23	postive
ATM	SOS1-IT1	0.69293935	2.54E-78	postive
FBXW7	SOS1-IT1	0.479922774	2.12E-32	postive
ZNF419	AC007066.2	0.687229371	1.46E-76	postive
VEGFA	AC007066.2	0.429638144	1.27E-25	postive
TUBE1	AC007066.2	0.655724409	1.50E-67	postive
SETD1B	AC007066.2	0.461468714	8.83E-30	postive
ALOX12	AC007066.2	0.600503212	4.04E-54	postive
GABPB1	AC007066.2	0.630446031	4.59E-61	postive
MAPK8	AC007066.2	0.461827282	7.88E-30	postive
LINC00472	AC007066.2	0.465094883	2.78E-30	postive
ATM	AC007066.2	0.500483622	1.64E-35	postive
TAZ	AC007066.2	0.471768778	3.19E-31	postive

FBXW7	AC007066.2	0.44050275	5.42E-27	postive
HSF1	LINC01311	0.448683086	4.67E-28	postive
ATF4	LINC01311	0.439355027	7.61E-27	postive
ZNF419	LINC01311	0.534046389	4.52E-41	postive
TUBE1	LINC01311	0.42571963	3.86E-25	postive
DRD4	LINC01311	0.43786577	1.18E-26	postive
ALOX12	LINC01311	0.469612916	6.45E-31	postive
NCOA4	LINC01311	-0.428585694	1.72E-25	negative
PHKG2	LINC01311	0.640033773	1.88E-63	postive
ULK1	LINC01311	0.429657281	1.27E-25	postive
TAZ	LINC01311	0.776305488	1.06E-109	postive
RPL8	SNHG32	0.465797276	2.22E-30	postive
FANCD2	AC079684.1	0.413329922	1.18E-23	postive
HELLS	AC079684.1	0.493998029	1.65E-34	postive
ZNF419	AC079684.1	0.487965745	1.36E-33	postive
TUBE1	AC079684.1	0.40997526	2.90E-23	postive
ALOX12	AC079684.1	0.504001752	4.57E-36	postive
GABPB1	AC079684.1	0.412137751	1.62E-23	postive
TAZ	AC079684.1	0.456652623	4.01E-29	postive
FBXW7	AC079684.1	0.417577911	3.71E-24	postive
FANCD2	AC096642.1	0.425635115	3.96E-25	postive
HELLS	AC096642.1	0.467026611	1.49E-30	postive
ALOX12	AC096642.1	0.52845109	4.22E-40	postive
TFAP2C	AC096642.1	0.488088644	1.30E-33	postive
HBA1	AC096642.1	0.439563214	7.16E-27	postive
DUOX1	AC096642.1	0.451686524	1.87E-28	postive
RRM2	AL035446.1	0.441089446	4.56E-27	postive
AURKA	AL035446.1	0.410083389	2.82E-23	postive
MAPK8	AL050309.1	0.402448119	2.11E-22	postive
ZNF419	AC005264.1	0.500672393	1.53E-35	postive
VEGFA	AC005264.1	0.589559215	9.23E-52	postive
TUBE1	AC005264.1	0.47431829	1.38E-31	postive
SETD1B	AC005264.1	0.427503686	2.33E-25	postive
ALOX12	AC005264.1	0.493476141	1.99E-34	postive
TAZ	AC005264.1	0.532736105	7.65E-41	postive
ISCU	KIF9-AS1	0.544381702	6.52E-43	postive
DDIT3	KIF9-AS1	0.477084445	5.49E-32	postive
SLC2A12	KIF9-AS1	0.495497549	9.73E-35	postive
ATG4D	KIF9-AS1	0.437986884	1.14E-26	postive
GABARAPL1	KIF9-AS1	0.60741827	1.17E-55	postive
BAP1	KIF9-AS1	0.515868067	5.53E-38	postive
LPIN1	KIF9-AS1	0.633798819	6.86E-62	postive
EIF2S1	AL121603.2	0.430624488	9.60E-26	postive
HMGB1	AL121603.2	0.517348346	3.15E-38	postive
NRAS	AL121603.2	0.439780725	6.71E-27	postive
FANCD2	AL591043.2	0.436482172	1.77E-26	postive
TFAP2C	AL591043.2	0.422845103	8.65E-25	postive
STAT3	AC125807.2	0.452244645	1.57E-28	postive
JDP2	AC125807.2	0.437149243	1.45E-26	postive
ZFP69B	AC125807.2	0.404904845	1.11E-22	postive
MAFG	AC125807.2	0.469744079	6.18E-31	postive
ZEB1	AC125807.2	0.461273482	9.39E-30	postive
EPAS1	AC125807.2	0.436041645	2.01E-26	postive
PRKAA2	AP001542.3	0.443779978	2.05E-27	postive
HELLS	AC008957.1	0.459789647	1.50E-29	postive
GABPB1	AC008957.1	0.444920088	1.45E-27	postive
CYBB	AC008957.1	0.461606421	8.45E-30	postive
ATG7	AC008957.1	0.57934636	1.22E-49	postive

LINC00472	AC008957.1	0.453935886	9.33E-29	postive
TGFBR1	AC008957.1	0.411569254	1.89E-23	postive
TLR4	AC008957.1	0.444748085	1.53E-27	postive
ATM	AC008957.1	0.655917377	1.33E-67	postive
FBXW7	AC008957.1	0.408231588	4.61E-23	postive
BACH1	AC008957.1	0.443942223	1.95E-27	postive
TFAP2C	LINC02572	0.484207872	4.95E-33	postive
HBA1	LINC02572	0.446054391	1.03E-27	postive
FANCD2	WAKMAR2	0.415390462	6.73E-24	postive
HELLS	WAKMAR2	0.486693015	2.11E-33	postive
ZNF419	WAKMAR2	0.476723172	6.19E-32	postive
TUBE1	WAKMAR2	0.515999808	5.26E-38	postive
ALOX12	WAKMAR2	0.550397927	5.16E-44	postive
GABPB1	WAKMAR2	0.567160878	3.33E-47	postive
TNFAIP3	WAKMAR2	0.447528837	6.63E-28	postive
ATM	WAKMAR2	0.55175547	2.89E-44	postive
FBXW7	WAKMAR2	0.593195795	1.55E-52	postive
GPX4	AP004609.3	0.410592774	2.46E-23	postive
HSPB1	AP004609.3	0.421751046	1.17E-24	postive
HBA1	AP004609.3	0.634412093	4.83E-62	postive
HRAS	AP004609.3	0.764301484	2.17E-104	postive
MAP1LC3A	AP004609.3	0.460200426	1.32E-29	postive
EGLN2	AP004609.3	0.830795316	9.21E-139	postive
ZNF419	ASH1L-AS1	0.403222276	1.72E-22	postive
TUBE1	ASH1L-AS1	0.420122031	1.84E-24	postive
SETD1B	ASH1L-AS1	0.416365253	5.16E-24	postive
ALOX12	ASH1L-AS1	0.520338406	1.00E-38	postive
GABPB1	ASH1L-AS1	0.44487728	1.47E-27	postive
LINC00472	ASH1L-AS1	0.41273514	1.38E-23	postive
BRD4	PDXDC2P-NI	0.412335569	1.54E-23	postive
ZNF419	PDXDC2P-NI	0.600054706	5.06E-54	postive
VEGFA	PDXDC2P-NI	0.470590924	4.69E-31	postive
TUBE1	PDXDC2P-NI	0.57095954	5.95E-48	postive
SETD1B	PDXDC2P-NI	0.474591719	1.26E-31	postive
ALOX12	PDXDC2P-NI	0.604144403	6.32E-55	postive
PHKG2	PDXDC2P-NI	0.541482938	2.17E-42	postive
TAZ	PDXDC2P-NI	0.634196949	5.46E-62	postive
FBXW7	PDXDC2P-NI	0.440069783	6.16E-27	postive
NOX1	AC069307.1	0.453790497	9.76E-29	postive
PHKG2	AC069307.1	0.41136323	2.00E-23	postive
BECN1	AC069307.1	-0.412227081	1.58E-23	negative
MAPK8	AC021087.1	0.440091021	6.13E-27	postive
ATM	AC021087.1	0.439056543	8.31E-27	postive
TUBE1	AC087752.4	0.516285199	4.72E-38	postive
GABPB1	AC087752.4	0.452206987	1.59E-28	postive
LINC00472	AC087752.4	0.415757148	6.09E-24	postive
HELLS	AC108727.1	0.407319951	5.87E-23	postive
KLHL24	AC108727.1	0.482502905	8.86E-33	postive
GABPB1	AC108727.1	0.496138836	7.75E-35	postive
LINC00472	AC108727.1	0.832265295	1.10E-139	postive
ATM	AC108727.1	0.442904165	2.66E-27	postive
PRKAA2	AC004053.1	0.431457164	7.56E-26	postive
ATM	AC004053.1	0.570277326	8.12E-48	postive
ALOX12	NOP53-AS1	0.414477013	8.63E-24	postive
ATM	NOP53-AS1	0.574480946	1.18E-48	postive
VEGFA	LINC02048	0.402234745	2.23E-22	postive
TUBE1	LINC02048	0.445988811	1.05E-27	postive
ALOX12	LINC02048	0.542609045	1.36E-42	postive

ATM	LINC02048	0.489588084	7.75E-34	postive
TAZ	LINC02048	0.406970056	6.44E-23	postive
LINC00472	AC091982.1	0.45832804	2.38E-29	postive
ATM	AC091982.1	0.510219043	4.62E-37	postive
PHKG2	AC003965.1	0.455185659	6.34E-29	postive
TFAP2C	AL512274.1	0.462853396	5.69E-30	postive
HELLS	AC005046.1	0.402798647	1.92E-22	postive
BRD4	AC005046.1	0.417585846	3.70E-24	postive
ZNF419	AC005046.1	0.4133024	1.19E-23	postive
ALOX12	AC005046.1	0.443886929	1.98E-27	postive
TAZ	AC005046.1	0.41036109	2.61E-23	postive
TFAP2C	AC010422.4	0.46889218	8.15E-31	postive
HBA1	AC010422.4	0.417003271	4.34E-24	postive
LINC00472	AC010422.4	0.55437583	9.38E-45	postive
HELLS	AKT3-IT1	0.544241149	6.91E-43	postive
TUBE1	AKT3-IT1	0.410002438	2.88E-23	postive
ALOX12	AKT3-IT1	0.428037732	2.01E-25	postive
GABPB1	AKT3-IT1	0.553631455	1.29E-44	postive
LINC00472	AKT3-IT1	0.730238042	6.64E-91	postive
ATM	AKT3-IT1	0.627090937	3.00E-60	postive
FBXW7	AKT3-IT1	0.486975189	1.92E-33	postive
HELLS	AC018752.1	0.481420703	1.28E-32	postive
MTOR	AC018752.1	0.451950135	1.72E-28	postive
KLHL24	AC018752.1	0.427823311	2.13E-25	postive
MAP3K5	AC018752.1	0.423280815	7.66E-25	postive
ALOX12	AC018752.1	0.402693905	1.98E-22	postive
IREB2	AC018752.1	0.609449821	4.06E-56	postive
GABPB1	AC018752.1	0.520251152	1.03E-38	postive
PIK3CA	AC018752.1	0.540031459	3.95E-42	postive
KRAS	AC018752.1	0.436130159	1.96E-26	postive
ATG7	AC018752.1	0.460799889	1.09E-29	postive
MAPK8	AC018752.1	0.554812833	7.76E-45	postive
LINC00472	AC018752.1	0.658484578	2.69E-68	postive
PRKAA2	AC018752.1	0.563960893	1.40E-46	postive
PRKAA1	AC018752.1	0.46292023	5.57E-30	postive
TLR4	AC018752.1	0.437502215	1.31E-26	postive
ATM	AC018752.1	0.764433054	1.90E-104	postive
FBXW7	AC018752.1	0.42515555	4.53E-25	postive
BACH1	AC018752.1	0.433081493	4.74E-26	postive
GCLC	PAXIP1-AS2	0.447551381	6.58E-28	postive
MTOR	PAXIP1-AS2	0.483291027	6.77E-33	postive
OXSR1	PAXIP1-AS2	0.40173816	2.54E-22	postive
KLHL24	PAXIP1-AS2	0.545790743	3.61E-43	postive
MAP3K5	PAXIP1-AS2	0.497386143	4.97E-35	postive
MAPK14	PAXIP1-AS2	0.42718202	2.56E-25	postive
EIF2AK4	PAXIP1-AS2	0.504386237	3.97E-36	postive
IREB2	PAXIP1-AS2	0.680038304	2.09E-74	postive
SP1	PAXIP1-AS2	0.553969164	1.12E-44	postive
PIK3CA	PAXIP1-AS2	0.60395328	6.97E-55	postive
KRAS	PAXIP1-AS2	0.484873128	3.95E-33	postive
HRAS	PAXIP1-AS2	-0.462128537	7.16E-30	negative
SLC38A1	PAXIP1-AS2	0.451904082	1.75E-28	postive
BECN1	PAXIP1-AS2	0.433040951	4.80E-26	postive
MAPK8	PAXIP1-AS2	0.496985535	5.73E-35	postive
MAPK9	PAXIP1-AS2	0.433978931	3.66E-26	postive
LINC00472	PAXIP1-AS2	0.415827044	5.98E-24	postive
PRKAA2	PAXIP1-AS2	0.470806179	4.37E-31	postive
PRKAA1	PAXIP1-AS2	0.525688009	1.25E-39	postive

ANO6	PAXIP1-AS2	0.429157794	1.46E-25	postive
TLR4	PAXIP1-AS2	0.434003193	3.63E-26	postive
ATM	PAXIP1-AS2	0.519422224	1.42E-38	postive
SIRT1	PAXIP1-AS2	0.421823399	1.15E-24	postive
HELLS	AP000223.1	0.50858422	8.47E-37	postive
TUBE1	AP000223.1	0.424825645	4.97E-25	postive
GABPB1	AP000223.1	0.504096472	4.41E-36	postive
LINC00472	AP000223.1	0.6259695	5.60E-60	postive
ATM	AP000223.1	0.626608708	3.93E-60	postive
FBXW7	AP000223.1	0.419450213	2.22E-24	postive
ISCU	AL138756.1	0.513752742	1.23E-37	postive
KLHL24	AL138756.1	0.539799492	4.35E-42	postive
GABARAPL1	AL138756.1	0.626868828	3.40E-60	postive
LINC00472	AL138756.1	0.580659018	6.59E-50	postive
LPIN1	AL138756.1	0.632890454	1.15E-61	postive
NCF2	AC002091.2	0.574475533	1.18E-48	postive
CYBB	AC002091.2	0.563781937	1.52E-46	postive
ATG7	AC002091.2	0.40066691	3.35E-22	postive
ALOX15B	AC002091.2	0.412889412	1.33E-23	postive
TLR4	AC002091.2	0.421620843	1.22E-24	postive
RB1	AC021483.2	0.424415495	5.57E-25	postive
GCLC	AC021483.2	0.401156095	2.95E-22	postive
IREB2	AC021483.2	0.590628193	5.48E-52	postive
PIK3CA	AC021483.2	0.427031891	2.67E-25	postive
NRAS	AC021483.2	0.411328113	2.02E-23	postive
KRAS	AC021483.2	0.429223832	1.43E-25	postive
NCOA4	AC021483.2	0.425221488	4.44E-25	postive
MAPK1	AC021483.2	0.525808986	1.19E-39	postive
MAPK8	AC021483.2	0.541435715	2.21E-42	postive
PRKAA2	AC021483.2	0.638442668	4.74E-63	postive
PRKAA1	AC021483.2	0.458711519	2.11E-29	postive
ATM	AC021483.2	0.631087441	3.20E-61	postive
SIRT1	AC021483.2	0.438770907	9.04E-27	postive
ISCU	AC010776.3	0.499124895	2.67E-35	postive
GPT2	AC010776.3	0.415233592	7.03E-24	postive
HERPUD1	AC010776.3	0.436036721	2.01E-26	postive
KLHL24	AC010776.3	0.571808051	4.04E-48	postive
SLC2A12	AC010776.3	0.505767999	2.39E-36	postive
ATG4D	AC010776.3	0.410485893	2.53E-23	postive
GABARAPL1	AC010776.3	0.720158787	2.66E-87	postive
WIPI2	AC010776.3	0.514120808	1.07E-37	postive
LINC00472	AC010776.3	0.518308395	2.18E-38	postive
LPIN1	AC010776.3	0.591872858	2.98E-52	postive
HELLS	AC005070.3	0.539154873	5.66E-42	postive
KLHL24	AC005070.3	0.466689373	1.66E-30	postive
TUBE1	AC005070.3	0.451399649	2.04E-28	postive
ALOX12	AC005070.3	0.405677408	9.05E-23	postive
IREB2	AC005070.3	0.439126839	8.14E-27	postive
GABPB1	AC005070.3	0.599313466	7.36E-54	postive
PIK3CA	AC005070.3	0.442332282	3.15E-27	postive
ATG7	AC005070.3	0.444280796	1.76E-27	postive
MAPK8	AC005070.3	0.445295788	1.30E-27	postive
LINC00472	AC005070.3	0.833039727	3.54E-140	postive
ATM	AC005070.3	0.715687849	9.38E-86	postive
FBXW7	AC005070.3	0.503648279	5.19E-36	postive
HELLS	MYLK-AS1	0.447919039	5.89E-28	postive
TUBE1	MYLK-AS1	0.435215091	2.56E-26	postive
ALOX12	MYLK-AS1	0.421355679	1.31E-24	postive

GABPB1	MYLK-AS1	0.457371787	3.21E-29	postive
LINC00472	MYLK-AS1	0.539525071	4.86E-42	postive
ATM	MYLK-AS1	0.496576727	6.63E-35	postive
FBXW7	MYLK-AS1	0.409779953	3.05E-23	postive
MTOR	ZKSCAN7-AS	0.435399129	2.42E-26	postive
ZNF419	ZKSCAN7-AS	0.421808022	1.15E-24	postive
KLHL24	ZKSCAN7-AS	0.541528646	2.13E-42	postive
IREB2	ZKSCAN7-AS	0.503501528	5.48E-36	postive
GABPB1	ZKSCAN7-AS	0.483171351	7.05E-33	postive
PIK3CA	ZKSCAN7-AS	0.516247077	4.78E-38	postive
ATG7	ZKSCAN7-AS	0.423648656	6.91E-25	postive
MAPK8	ZKSCAN7-AS	0.446397434	9.33E-28	postive
LINC00472	ZKSCAN7-AS	0.636918631	1.15E-62	postive
PRKAA1	ZKSCAN7-AS	0.425555275	4.05E-25	postive
LPIN1	ZKSCAN7-AS	0.495648941	9.22E-35	postive
ATM	ZKSCAN7-AS	0.516098354	5.06E-38	postive
HELLS	AC016590.2	0.42817427	1.93E-25	postive
KLHL24	AC016590.2	0.564791126	9.66E-47	postive
TUBE1	AC016590.2	0.420330495	1.74E-24	postive
IREB2	AC016590.2	0.488929445	9.75E-34	postive
GABPB1	AC016590.2	0.555953622	4.74E-45	postive
PIK3CA	AC016590.2	0.504147206	4.33E-36	postive
KRAS	AC016590.2	0.415659467	6.26E-24	postive
MAPK8	AC016590.2	0.461872212	7.77E-30	postive
LINC00472	AC016590.2	0.914731213	1.73E-213	postive
PRKAA1	AC016590.2	0.422217894	1.03E-24	postive
ATM	AC016590.2	0.679609121	2.80E-74	postive
FBXW7	AC016590.2	0.478594217	3.31E-32	postive
TUBE1	AC078922.1	0.483363421	6.61E-33	postive
ALOX12	AC078922.1	0.474577996	1.26E-31	postive
TUBE1	ADAMTS9-A	0.458278941	2.41E-29	postive
ALOX12	ADAMTS9-A	0.416983339	4.36E-24	postive
PEBP1	ADAMTS9-A	0.402938659	1.86E-22	postive
MIOX	ADAMTS9-A	0.455033169	6.64E-29	postive
HELLS	AC009061.2	0.415028062	7.43E-24	postive
ZNF419	AC009061.2	0.401178308	2.93E-22	postive
ALOX12	AC009061.2	0.404028748	1.40E-22	postive
PHKG2	AC009061.2	0.455546541	5.66E-29	postive
SLC2A12	LINC00645	0.77320953	2.66E-108	postive
WIPI2	LINC00645	0.543528758	9.30E-43	postive
ISCU	AL139275.2	0.400377716	3.61E-22	postive
ACO1	AL139275.2	0.520096867	1.10E-38	postive
TUBE1	AL133338.1	0.430198115	1.08E-25	postive
OXSR1	ACVR2B-AS1	0.40966567	3.15E-23	postive
HELLS	AC245884.1C	0.532771301	7.54E-41	postive
ZNF419	AC245884.1C	0.454928145	6.86E-29	postive
TUBE1	AC245884.1C	0.436187715	1.93E-26	postive
ALOX12	AC245884.1C	0.534948258	3.14E-41	postive
GABPB1	AC245884.1C	0.504114544	4.38E-36	postive
LINC00472	AC245884.1C	0.648686407	1.11E-65	postive
ATM	AC245884.1C	0.560221198	7.34E-46	postive
FBXW7	AC245884.1C	0.469969565	5.74E-31	postive
ISCU	EMSLR	0.450181017	2.96E-28	postive
ASNS	EMSLR	0.44720895	7.30E-28	postive
DDIT3	EMSLR	0.499618846	2.23E-35	postive
GABARAPL1	EMSLR	0.492042308	3.29E-34	postive
WIPI2	EMSLR	0.429414528	1.36E-25	postive
HELLS	WWTR1-IT1	0.502080191	9.18E-36	postive

ALOX12	WWTR1-IT1	0.430545662	9.82E-26	postive
TFAP2C	WWTR1-IT1	0.432108512	6.27E-26	postive
HBA1	WWTR1-IT1	0.404485908	1.24E-22	postive
GABPB1	WWTR1-IT1	0.429675966	1.26E-25	postive
DUOX1	WWTR1-IT1	0.444742931	1.53E-27	postive
LINC00472	WWTR1-IT1	0.522777648	3.90E-39	postive
ATM	WWTR1-IT1	0.449831429	3.29E-28	postive
FBXW7	WWTR1-IT1	0.408057852	4.83E-23	postive
ZNF419	HNRNPD-DT	0.431938608	6.59E-26	postive
HRAS	HNRNPD-DT	0.401770698	2.51E-22	postive
NCOA4	HNRNPD-DT	-0.404891399	1.11E-22	negative
PHKG2	HNRNPD-DT	0.545392849	4.27E-43	postive
TAZ	HNRNPD-DT	0.636204711	1.73E-62	postive
HELLS	AC067852.3	0.430572591	9.75E-26	postive
ZNF419	AC067852.3	0.415818147	5.99E-24	postive
VEGFA	AC067852.3	0.435651609	2.25E-26	postive
TUBE1	AC067852.3	0.592654112	2.03E-52	postive
SETD1B	AC067852.3	0.476433002	6.82E-32	postive
ALOX12	AC067852.3	0.727733111	5.40E-90	postive
GABPB1	AC067852.3	0.402537718	2.06E-22	postive
MAPK8	AC067852.3	0.480825123	1.56E-32	postive
LINC00472	AC067852.3	0.416800946	4.58E-24	postive
ATM	AC067852.3	0.682999249	2.75E-75	postive
FBXW7	AC067852.3	0.452089842	1.65E-28	postive
EIF2S1	MIR100HG	0.427085568	2.63E-25	postive
CDKN2A	MIR100HG	0.448692673	4.66E-28	postive
LINC00472	MIR100HG	0.499237523	2.56E-35	postive
LPIN1	MIR100HG	0.420912548	1.48E-24	postive
ZNF419	AC106782.5	0.407749233	5.24E-23	postive
VEGFA	AC106782.5	0.443218352	2.42E-27	postive
DRD4	AC106782.5	0.415703989	6.18E-24	postive
ALOX12	AC106782.5	0.546410239	2.79E-43	postive
NCOA4	AC106782.5	-0.421355628	1.31E-24	negative
PHKG2	AC106782.5	0.538245708	8.21E-42	postive
TAZ	AC106782.5	0.663953133	8.48E-70	postive
HELLS	AC124312.3	0.428774183	1.63E-25	postive
MTOR	AC124312.3	0.43601816	2.02E-26	postive
KLHL24	AC124312.3	0.512227571	2.18E-37	postive
MAP3K5	AC124312.3	0.416603503	4.84E-24	postive
IREB2	AC124312.3	0.455684318	5.43E-29	postive
GABPB1	AC124312.3	0.51633122	4.63E-38	postive
PIK3CA	AC124312.3	0.476567755	6.52E-32	postive
ATG7	AC124312.3	0.489551234	7.85E-34	postive
MAPK8	AC124312.3	0.402848465	1.90E-22	postive
LINC00472	AC124312.3	0.62955705	7.57E-61	postive
PRKAA2	AC124312.3	0.401782147	2.51E-22	postive
PRKAA1	AC124312.3	0.456033618	4.87E-29	postive
ATM	AC124312.3	0.696184109	2.44E-79	postive
FBXW7	AC124312.3	0.544817054	5.43E-43	postive
FANCD2	PANK2-AS1	0.447865171	5.99E-28	postive
HELLS	PANK2-AS1	0.422291939	1.01E-24	postive
ALOX12	PANK2-AS1	0.459049413	1.89E-29	postive
TAZ	AC090578.2	0.417668362	3.62E-24	postive
GPX4	AC006942.1	0.459613563	1.59E-29	postive
RB1	AC006942.1	-0.450045295	3.08E-28	negative
HSF1	AC006942.1	0.482218327	9.75E-33	postive
STAT3	AC006942.1	-0.493322373	2.10E-34	negative
ISCU	AC006942.1	0.411674244	1.84E-23	postive

OTUB1	AC006942.1	0.465127758	2.75E-30	postive
DDIT3	AC006942.1	0.474740189	1.20E-31	postive
MAPK14	AC006942.1	-0.449149282	4.05E-28	negative
NRAS	AC006942.1	-0.503556073	5.37E-36	negative
NCOA4	AC006942.1	-0.406622896	7.06E-23	negative
PHKG2	AC006942.1	0.47788001	4.21E-32	postive
ATG4D	AC006942.1	0.589961915	7.59E-52	postive
BECN1	AC006942.1	-0.410474635	2.54E-23	negative
MAP1LC3A	AC006942.1	0.537690388	1.03E-41	postive
MAPK1	AC006942.1	-0.425878306	3.69E-25	negative
ANO6	AC006942.1	-0.466357748	1.85E-30	negative
TAZ	AC006942.1	0.492060175	3.27E-34	postive
MTDH	AC006942.1	-0.441683785	3.82E-27	negative
BACH1	AC006942.1	-0.415188172	7.11E-24	negative
HELLS	INE1	0.406534932	7.22E-23	postive
BRD4	INE1	0.469761417	6.14E-31	postive
ZNF419	INE1	0.574036429	1.45E-48	postive
VEGFA	INE1	0.451168164	2.19E-28	postive
TUBE1	INE1	0.546667425	2.50E-43	postive
SETD1B	INE1	0.569762754	1.03E-47	postive
ALOX12	INE1	0.585713965	5.93E-51	postive
GABPB1	INE1	0.440898917	4.82E-27	postive
LINC00472	INE1	0.44638061	9.38E-28	postive
ATM	INE1	0.455402746	5.92E-29	postive
TAZ	INE1	0.464628642	3.22E-30	postive
FBXW7	INE1	0.437203571	1.43E-26	postive
MUC1	SEMA3B-AS1	0.433702749	3.96E-26	postive
ISCU	SEMA3B-AS1	0.479634317	2.34E-32	postive
DDIT3	SEMA3B-AS1	0.499903158	2.02E-35	postive
SLC2A8	SEMA3B-AS1	0.6024551	1.50E-54	postive
ATG4D	SEMA3B-AS1	0.445125898	1.37E-27	postive
MAP1LC3A	SEMA3B-AS1	0.606211348	2.18E-55	postive
GABARAPL1	SEMA3B-AS1	0.514467969	9.39E-38	postive
SCP2	AC007485.1	0.418294321	3.05E-24	postive
RB1	AP001486.2	0.52599353	1.11E-39	postive
OXSR1	AP001486.2	0.438985458	8.49E-27	postive
TUBE1	AP001486.2	0.517525358	2.94E-38	postive
IREB2	AP001486.2	0.541190441	2.45E-42	postive
HMGB1	AP001486.2	0.477883382	4.20E-32	postive
SP1	AP001486.2	0.422874324	8.57E-25	postive
GABPB1	AP001486.2	0.564810772	9.58E-47	postive
EMC2	AP001486.2	0.445534411	1.21E-27	postive
PIK3CA	AP001486.2	0.443109641	2.50E-27	postive
NRAS	AP001486.2	0.437297237	1.39E-26	postive
KRAS	AP001486.2	0.476018924	7.83E-32	postive
ULK2	AP001486.2	0.426040493	3.53E-25	postive
MAPK1	AP001486.2	0.426531299	3.07E-25	postive
ZEB1	AP001486.2	0.509524788	5.98E-37	postive
MAPK8	AP001486.2	0.673832713	1.36E-72	postive
PRKAA2	AP001486.2	0.611491029	1.39E-56	postive
PRKAA1	AP001486.2	0.545389938	4.28E-43	postive
ATM	AP001486.2	0.603687984	7.98E-55	postive
SIRT1	AP001486.2	0.547409788	1.83E-43	postive
BAP1	ILF3-DT	0.413874991	1.02E-23	postive
EGLN2	ILF3-DT	0.407819091	5.14E-23	postive
SLC1A4	MIR1915HG	0.419762228	2.03E-24	postive
MAPK3	MIR1915HG	0.474170543	1.45E-31	postive
ZEB1	MIR1915HG	0.420032942	1.89E-24	postive

EPAS1	MIR1915HG	0.456664043	4.00E-29	postive
HELLS	AC096992.2	0.436693068	1.66E-26	postive
ZNF419	AC096992.2	0.454594634	7.61E-29	postive
TUBE1	AC096992.2	0.595664608	4.57E-53	postive
SETD1B	AC096992.2	0.404274516	1.31E-22	postive
ALOX12	AC096992.2	0.60113187	2.94E-54	postive
SP1	AC096992.2	0.414345199	8.94E-24	postive
GABPB1	AC096992.2	0.521308442	6.88E-39	postive
MAPK8	AC096992.2	0.424127105	6.04E-25	postive
ATM	AC096992.2	0.672499941	3.28E-72	postive
FBXW7	AC096992.2	0.453529485	1.06E-28	postive
MAPK14	AL606489.1	0.427939528	2.06E-25	postive
ALOX12	AL606489.1	0.401351441	2.80E-22	postive
ATM	AL606489.1	0.494743871	1.27E-34	postive
GPX4	FLJ20021	0.543350739	1.00E-42	postive
RB1	FLJ20021	-0.407903206	5.03E-23	negative
STAT3	FLJ20021	-0.441609385	3.91E-27	negative
MTOR	FLJ20021	-0.421594123	1.22E-24	negative
OTUB1	FLJ20021	0.507913644	1.09E-36	postive
RPL8	FLJ20021	0.627123057	2.95E-60	postive
MAPK14	FLJ20021	-0.445891745	1.09E-27	negative
RIPK1	FLJ20021	-0.407374537	5.79E-23	negative
KRAS	FLJ20021	-0.402500775	2.08E-22	negative
HRAS	FLJ20021	0.559313072	1.09E-45	postive
BECN1	FLJ20021	-0.417963455	3.33E-24	negative
MAP1LC3A	FLJ20021	0.486884205	1.98E-33	postive
ANO6	FLJ20021	-0.415096946	7.29E-24	negative
FANCD2	AC016394.1	0.43960996	7.06E-27	postive
HELLS	AC016394.1	0.673643884	1.54E-72	postive
ZNF419	AC016394.1	0.431065998	8.46E-26	postive
TUBE1	AC016394.1	0.514322079	9.92E-38	postive
ALOX12	AC016394.1	0.500428327	1.67E-35	postive
GABPB1	AC016394.1	0.553133694	1.60E-44	postive
ATG7	AC016394.1	0.41000061	2.88E-23	postive
LINC00472	AC016394.1	0.55274336	1.89E-44	postive
ATM	AC016394.1	0.646862194	3.31E-65	postive
FBXW7	AC016394.1	0.469986804	5.71E-31	postive
HELLS	AL137244.1	0.415786911	6.05E-24	postive
ZNF419	AL137244.1	0.50961504	5.78E-37	postive
TUBE1	AL137244.1	0.555479453	5.82E-45	postive
ALOX12	AL137244.1	0.470872403	4.27E-31	postive
GABPB1	AL137244.1	0.51465801	8.74E-38	postive
LINC00472	AL137244.1	0.720459648	2.09E-87	postive
LPIN1	AL137244.1	0.421194273	1.37E-24	postive
ATM	AL137244.1	0.512078339	2.31E-37	postive
FBXW7	AL137244.1	0.425834084	3.74E-25	postive
HELLS	AC092755.1	0.528557077	4.05E-40	postive
TUBE1	AC092755.1	0.479357352	2.56E-32	postive
ALOX12	AC092755.1	0.414817936	7.87E-24	postive
GABPB1	AC092755.1	0.585651318	6.11E-51	postive
ATG7	AC092755.1	0.447993534	5.76E-28	postive
MAPK8	AC092755.1	0.422719559	8.95E-25	postive
LINC00472	AC092755.1	0.587306854	2.75E-51	postive
ATM	AC092755.1	0.74993439	1.95E-98	postive
FBXW7	AC092755.1	0.551858701	2.77E-44	postive
HSPA5	AC004148.1	-0.426549001	3.06E-25	negative
BRD4	AC004148.1	0.441243877	4.36E-27	postive
ZNF419	AC004148.1	0.634075973	5.85E-62	postive

VEGFA	AC004148.1	0.534069851	4.47E-41	postive
TUBE1	AC004148.1	0.646486682	4.15E-65	postive
SETD1B	AC004148.1	0.507470327	1.28E-36	postive
DRD4	AC004148.1	0.434250906	3.38E-26	postive
ALOX12	AC004148.1	0.694140426	1.07E-78	postive
PHKG2	AC004148.1	0.535265269	2.76E-41	postive
TAZ	AC004148.1	0.736400488	3.46E-93	postive
FBXW7	AC004148.1	0.427173242	2.56E-25	postive
HELLS	LRIG2-DT	0.601065046	3.04E-54	postive
TUBE1	LRIG2-DT	0.456351095	4.41E-29	postive
ALOX12	LRIG2-DT	0.53573959	2.28E-41	postive
GABPB1	LRIG2-DT	0.536988552	1.37E-41	postive
LINC00472	LRIG2-DT	0.586436287	4.19E-51	postive
ATM	LRIG2-DT	0.666794092	1.37E-70	postive
FBXW7	LRIG2-DT	0.474574247	1.26E-31	postive
ISCU	HOXB-AS3	0.609278439	4.44E-56	postive
DDIT3	HOXB-AS3	0.443509869	2.22E-27	postive
SLC2A8	HOXB-AS3	0.442436366	3.06E-27	postive
ATG4D	HOXB-AS3	0.61953191	1.91E-58	postive
MAP1LC3A	HOXB-AS3	0.584500373	1.06E-50	postive
GABARAPL2	HOXB-AS3	0.508283865	9.47E-37	postive
GABARAPL1	HOXB-AS3	0.525328882	1.44E-39	postive
BAP1	HOXB-AS3	0.421117354	1.40E-24	postive
LPIN1	HOXB-AS3	0.46500268	2.86E-30	postive
FANCD2	AL021707.2	0.41567353	6.23E-24	postive
HELLS	AL021707.2	0.424495048	5.45E-25	postive
ZNF419	AL021707.2	0.507741816	1.16E-36	postive
TUBE1	AL021707.2	0.44627656	9.67E-28	postive
ALOX12	AL021707.2	0.599420455	6.97E-54	postive
DUOX1	AL021707.2	0.406864926	6.62E-23	postive
TFAP2C	AC019080.3	0.572306926	3.21E-48	postive
HBA1	AC019080.3	0.512082361	2.30E-37	postive
DUOX1	AC019080.3	0.421406686	1.29E-24	postive
ISCU	AC090515.2	0.51543402	6.51E-38	postive
SLC2A8	AC090515.2	0.419561397	2.15E-24	postive
TFAP2C	AC090515.2	0.415091883	7.30E-24	postive
ATG4D	AC090515.2	0.534323016	4.04E-41	postive
MAP1LC3A	AC090515.2	0.433082953	4.74E-26	postive
GABARAPL1	AC090515.2	0.539996856	4.01E-42	postive
LPIN1	AC090515.2	0.533320099	6.05E-41	postive
HRAS	EXOC3-AS1	0.428159484	1.94E-25	postive
HELLS	AL357078.2	0.513685811	1.26E-37	postive
KLHL24	AL357078.2	0.414484438	8.61E-24	postive
TUBE1	AL357078.2	0.446113588	1.02E-27	postive
ALOX12	AL357078.2	0.454844699	7.04E-29	postive
GABPB1	AL357078.2	0.557314432	2.62E-45	postive
MAPK8	AL357078.2	0.418732035	2.70E-24	postive
LINC00472	AL357078.2	0.805575717	3.64E-124	postive
ATM	AL357078.2	0.653412023	6.25E-67	postive
FBXW7	AL357078.2	0.466158052	1.97E-30	postive
TUBE1	HMGN3-AS1	0.508468977	8.84E-37	postive
ATM	HMGN3-AS1	0.483256087	6.85E-33	postive
ISCU	MAP4K3-DT	0.532146061	9.69E-41	postive
TMBIM4	MAP4K3-DT	0.40993442	2.93E-23	postive
ZNF419	AL049780.1	0.483356193	6.62E-33	postive
TUBE1	AL049780.1	0.448432396	5.04E-28	postive
ALOX12	AL049780.1	0.553083918	1.64E-44	postive
TAZ	AL049780.1	0.545584123	3.94E-43	postive

FBXW7	AL049780.1	0.424226084	5.88E-25	postive
ALOX12	AC114488.1	0.451743596	1.83E-28	postive
TFAP2C	AC114488.1	0.461513942	8.70E-30	postive
HBA1	AC114488.1	0.423417652	7.37E-25	postive
PHKG2	AC104794.3	0.406642193	7.02E-23	postive
TAZ	AC104794.3	0.447642554	6.40E-28	postive
MTOR	LINC01521	0.420940953	1.47E-24	postive
MAFG	LINC01521	0.417969842	3.33E-24	postive
BECN1	LINC01521	0.429573268	1.30E-25	postive
HELLS	AP000866.6	0.509229678	6.67E-37	postive
ZNF419	AP000866.6	0.420964082	1.46E-24	postive
KLHL24	AP000866.6	0.444600273	1.60E-27	postive
TUBE1	AP000866.6	0.497006694	5.69E-35	postive
ALOX12	AP000866.6	0.404720858	1.16E-22	postive
GABPB1	AP000866.6	0.600000996	5.20E-54	postive
MAPK8	AP000866.6	0.409227605	3.54E-23	postive
LINC00472	AP000866.6	0.848697836	1.12E-150	postive
ATM	AP000866.6	0.655498769	1.73E-67	postive
FBXW7	AP000866.6	0.484695561	4.19E-33	postive
SLC2A12	AC010273.3	0.40671208	6.89E-23	postive
ACO1	AC010273.3	0.409421636	3.36E-23	postive
DDIT3	ITPR1-DT	0.404168146	1.35E-22	postive
ATG4D	ITPR1-DT	0.434343256	3.29E-26	postive
GABARAPL1	ITPR1-DT	0.422223547	1.03E-24	postive
WIPI2	ITPR1-DT	0.411159144	2.11E-23	postive
LPIN1	ITPR1-DT	0.480200711	1.93E-32	postive
TSC22D3	HS1BP3-IT1	0.431538748	7.39E-26	postive
ZNF419	AC119674.1	0.433056065	4.77E-26	postive
TUBE1	AC119674.1	0.543612999	8.98E-43	postive
ALOX12	AC119674.1	0.448096673	5.58E-28	postive
GABPB1	AC119674.1	0.536026207	2.03E-41	postive
ZEB1	AC119674.1	0.414086928	9.59E-24	postive
ATM	AC119674.1	0.513267724	1.48E-37	postive
FBXW7	AC119674.1	0.546090266	3.19E-43	postive
BRD4	AC087289.2	0.439324877	7.68E-27	postive
ZNF419	AC087289.2	0.515559909	6.21E-38	postive
VEGFA	AC087289.2	0.441448478	4.10E-27	postive
TUBE1	AC087289.2	0.507497072	1.27E-36	postive
SETD1B	AC087289.2	0.445491745	1.23E-27	postive
DRD4	AC087289.2	0.444055028	1.89E-27	postive
ALOX12	AC087289.2	0.525044011	1.61E-39	postive
NCOA4	AC087289.2	-0.454700965	7.36E-29	negative
PHKG2	AC087289.2	0.673080711	2.24E-72	postive
ULK1	AC087289.2	0.428076831	1.98E-25	postive
TAZ	AC087289.2	0.783441635	5.09E-113	postive
HELLS	AP001178.2	0.551099929	3.83E-44	postive
TUBE1	AP001178.2	0.467741091	1.18E-30	postive
ALOX12	AP001178.2	0.498741565	3.06E-35	postive
GABPB1	AP001178.2	0.520698931	8.71E-39	postive
ATG7	AP001178.2	0.415879102	5.90E-24	postive
MAPK8	AP001178.2	0.420561054	1.63E-24	postive
LINC00472	AP001178.2	0.54342853	9.69E-43	postive
ATM	AP001178.2	0.764181071	2.44E-104	postive
FBXW7	AP001178.2	0.461906085	7.68E-30	postive
HELLS	LINC01515	0.572529007	2.90E-48	postive
TUBE1	LINC01515	0.471598059	3.37E-31	postive
ALOX12	LINC01515	0.465297376	2.60E-30	postive
IREB2	LINC01515	0.427448128	2.37E-25	postive

SP1	LINC01515	0.401642241	2.60E-22	postive
GABPB1	LINC01515	0.500354994	1.71E-35	postive
ATG7	LINC01515	0.414654445	8.22E-24	postive
MAPK8	LINC01515	0.489638155	7.62E-34	postive
LINC00472	LINC01515	0.406770464	6.79E-23	postive
PRKAA2	LINC01515	0.483762804	5.77E-33	postive
ATM	LINC01515	0.794095745	3.26E-118	postive
FBXW7	LINC01515	0.469484238	6.72E-31	postive
BACH1	LINC01515	0.408479829	4.32E-23	postive
TUBE1	AC007406.1	0.409834403	3.01E-23	postive
ALOX12	AC007406.1	0.506305369	1.96E-36	postive
GDF15	AL135924.2	0.439864632	6.55E-27	postive
MIOX	AL135924.2	0.411546665	1.90E-23	postive
TUBE1	KCNIP2-AS1	0.433016217	4.83E-26	postive
ALOX12	KCNIP2-AS1	0.579954518	9.18E-50	postive
HELLS	AC008764.8	0.458991452	1.93E-29	postive
BRD4	AC008764.8	0.426755753	2.88E-25	postive
ZNF419	AC008764.8	0.558380602	1.65E-45	postive
TUBE1	AC008764.8	0.574792709	1.02E-48	postive
SETD1B	AC008764.8	0.439246503	7.86E-27	postive
DRD4	AC008764.8	0.435108068	2.64E-26	postive
ALOX12	AC008764.8	0.697650337	8.38E-80	postive
PHKG2	AC008764.8	0.561460422	4.25E-46	postive
ATM	AC008764.8	0.450171251	2.97E-28	postive
TAZ	AC008764.8	0.658454742	2.74E-68	postive
FBXW7	AC008764.8	0.404356249	1.28E-22	postive
SLC1A4	HID1-AS1	0.459407473	1.69E-29	postive
HIC1	HID1-AS1	0.419568217	2.14E-24	postive
MAPK3	HID1-AS1	0.470336299	5.09E-31	postive
ZEB1	HID1-AS1	0.714252505	2.90E-85	postive
EPAS1	HID1-AS1	0.724607201	7.15E-89	postive
ZNF419	AC093157.1	0.422149134	1.05E-24	postive
TUBE1	AC093157.1	0.554414228	9.22E-45	postive
PHKG2	AC093157.1	0.420637724	1.60E-24	postive
TAZ	AC093157.1	0.508377768	9.15E-37	postive
KLHL24	AC010333.2	0.611000142	1.80E-56	postive
IREB2	AC010333.2	0.426529242	3.08E-25	postive
GABPB1	AC010333.2	0.402332312	2.17E-22	postive
PIK3CA	AC010333.2	0.468932745	8.04E-31	postive
KRAS	AC010333.2	0.412965998	1.30E-23	postive
LINC00472	AC010333.2	0.88487127	2.70E-180	postive
LPIN1	AC010333.2	0.478342073	3.61E-32	postive
ATM	AC010333.2	0.462988937	5.44E-30	postive
BRD4	AL139287.1	0.442865429	2.69E-27	postive
ZNF419	AL139287.1	0.592212398	2.52E-52	postive
VEGFA	AL139287.1	0.627538371	2.34E-60	postive
TUBE1	AL139287.1	0.594813847	6.98E-53	postive
SETD1B	AL139287.1	0.588843224	1.31E-51	postive
ALOX12	AL139287.1	0.722877214	2.94E-88	postive
ATM	AL139287.1	0.412420316	1.50E-23	postive
YY1AP1	AL139287.1	0.48045067	1.77E-32	postive
TAZ	AL139287.1	0.597300355	2.02E-53	postive
HELLS	GHRLOS	0.531615907	1.20E-40	postive
ZNF419	GHRLOS	0.465162291	2.72E-30	postive
TUBE1	GHRLOS	0.54367995	8.73E-43	postive
ALOX12	GHRLOS	0.537990121	9.12E-42	postive
GABPB1	GHRLOS	0.525707979	1.24E-39	postive
LINC00472	GHRLOS	0.435632914	2.26E-26	postive

ATM	GHRLOS	0.614172856	3.37E-57	postive
TAZ	GHRLOS	0.400361438	3.62E-22	postive
FBXW7	GHRLOS	0.539609548	4.70E-42	postive
FANCD2	MAP3K14-AS	0.444313545	1.74E-27	postive
HELLS	MAP3K14-AS	0.439525762	7.24E-27	postive
TUBE1	MAP3K14-AS	0.562745405	2.40E-46	postive
SETD1B	MAP3K14-AS	0.402143032	2.28E-22	postive
ALOX12	MAP3K14-AS	0.64766524	2.05E-65	postive
ATM	MAP3K14-AS	0.44555128	1.20E-27	postive
TAZ	MAP3K14-AS	0.436071085	1.99E-26	postive
PLIN2	AC009053.3	0.412283514	1.56E-23	postive
MAPK1	AC009053.3	0.417196146	4.12E-24	postive
FANCD2	MYOSLID	0.444878476	1.47E-27	postive
STMN1	MYOSLID	0.7508605	8.29E-99	postive
RRM2	MYOSLID	0.55282902	1.82E-44	postive
AURKA	MYOSLID	0.568528565	1.80E-47	postive
CDKN2A	MYOSLID	0.802129069	2.45E-122	postive
CD44	LINC01588	0.419383662	2.26E-24	postive
SOCS1	LINC01588	0.44683282	8.18E-28	postive
GABPB1	AC005884.1	0.432465976	5.66E-26	postive
ZEB1	AC005884.1	0.407307559	5.89E-23	postive
LINC00472	AC005884.1	0.463486393	4.65E-30	postive
BRD4	AL022328.1	0.408921719	3.84E-23	postive
ZNF419	AL022328.1	0.566986939	3.61E-47	postive
VEGFA	AL022328.1	0.540812527	2.86E-42	postive
TUBE1	AL022328.1	0.537404747	1.16E-41	postive
SETD1B	AL022328.1	0.520260265	1.03E-38	postive
DRD4	AL022328.1	0.426655128	2.97E-25	postive
ALOX12	AL022328.1	0.71472327	2.00E-85	postive
YY1AP1	AL022328.1	0.431796052	6.86E-26	postive
TAZ	AL022328.1	0.558667094	1.45E-45	postive
ZNF419	AP000442.1	0.500791313	1.46E-35	postive
TUBE1	AP000442.1	0.5852566	7.39E-51	postive
SETD1B	AP000442.1	0.405422815	9.68E-23	postive
ALOX12	AP000442.1	0.588095512	1.88E-51	postive
PHKG2	AP000442.1	0.447540974	6.60E-28	postive
TAZ	AP000442.1	0.523796586	2.62E-39	postive
BRD4	ZNF528-AS1	0.412931423	1.31E-23	postive
ZNF419	ZNF528-AS1	0.642132542	5.49E-64	postive
VEGFA	ZNF528-AS1	0.401800335	2.50E-22	postive
TUBE1	ZNF528-AS1	0.41514574	7.20E-24	postive
ALOX12	ZNF528-AS1	0.476160162	7.47E-32	postive
PHKG2	ZNF528-AS1	0.491106889	4.56E-34	postive
ULK1	ZNF528-AS1	0.448790319	4.52E-28	postive
TAZ	ZNF528-AS1	0.560742106	5.83E-46	postive
ZNF419	Z97989.1	0.40595168	8.42E-23	postive
VEGFA	Z97989.1	0.416916917	4.44E-24	postive
TUBE1	Z97989.1	0.517695475	2.76E-38	postive
GABPB1	Z97989.1	0.451670249	1.88E-28	postive
ZEB1	Z97989.1	0.419561331	2.15E-24	postive
BRD4	LINC00342	0.466541511	1.74E-30	postive
ZNF419	LINC00342	0.577373889	3.08E-49	postive
VEGFA	LINC00342	0.538603622	7.10E-42	postive
TUBE1	LINC00342	0.588673535	1.42E-51	postive
SETD1B	LINC00342	0.470143285	5.42E-31	postive
DRD4	LINC00342	0.462336554	6.70E-30	postive
ALOX12	LINC00342	0.632750366	1.25E-61	postive
PHKG2	LINC00342	0.522183464	4.91E-39	postive

TAZ	LINC00342	0.75027587	1.42E-98	postive
FBXW7	LINC00342	0.423236179	7.75E-25	postive
ZNF419	ZNF32-AS1	0.479123074	2.77E-32	postive
TUBE1	ZNF32-AS1	0.504333671	4.05E-36	postive
ALOX12	ZNF32-AS1	0.421736812	1.18E-24	postive
PHKG2	ZNF32-AS1	0.539812108	4.32E-42	postive
TAZ	ZNF32-AS1	0.690673177	1.28E-77	postive
FBXW7	ZNF32-AS1	0.406734679	6.85E-23	postive
HELLS	AC015911.3	0.544915528	5.21E-43	postive
TUBE1	AC015911.3	0.452598995	1.41E-28	postive
ALOX12	AC015911.3	0.502453818	8.02E-36	postive
GABPB1	AC015911.3	0.511910303	2.46E-37	postive
ATG7	AC015911.3	0.421607179	1.22E-24	postive
IFNG	AC015911.3	0.441849545	3.64E-27	postive
ATM	AC015911.3	0.666720635	1.43E-70	postive
FBXW7	AC015911.3	0.523014986	3.55E-39	postive
EGLN2	AC107294.2	0.435877754	2.11E-26	postive
FANCD2	FAM66C	0.442107513	3.37E-27	postive
HELLS	FAM66C	0.470920731	4.21E-31	postive
ALOX12	FAM66C	0.429578688	1.29E-25	postive
TFAP2C	FAM66C	0.446864867	8.10E-28	postive
DUOX1	FAM66C	0.451856872	1.77E-28	postive
HELLS	ARMC2-AS1	0.452718642	1.36E-28	postive
TUBE1	ARMC2-AS1	0.491994339	3.35E-34	postive
ALOX12	ARMC2-AS1	0.516466581	4.40E-38	postive
GABPB1	ARMC2-AS1	0.443888023	1.98E-27	postive
ATM	ARMC2-AS1	0.686261931	2.86E-76	postive
FBXW7	ARMC2-AS1	0.463731434	4.30E-30	postive
HELLS	AC010536.2	0.550810554	4.33E-44	postive
TUBE1	AC010536.2	0.421598929	1.22E-24	postive
ALOX12	AC010536.2	0.478990668	2.90E-32	postive
GABPB1	AC010536.2	0.487966934	1.36E-33	postive
LINC00472	AC010536.2	0.566916809	3.72E-47	postive
ATM	AC010536.2	0.663304651	1.28E-69	postive
FBXW7	AC010536.2	0.468361521	9.68E-31	postive
TUBE1	AC026979.3	0.525352866	1.43E-39	postive
ATM	AC026979.3	0.44429548	1.75E-27	postive
TFAP2C	AC087301.1	0.577275238	3.22E-49	postive
HBA1	AC087301.1	0.537571891	1.08E-41	postive
DUOX1	AC087301.1	0.415757642	6.09E-24	postive
HELLS	Z83843.1	0.495678175	9.12E-35	postive
ZNF419	Z83843.1	0.410270985	2.68E-23	postive
KLHL24	Z83843.1	0.491339873	4.21E-34	postive
TUBE1	Z83843.1	0.476607396	6.44E-32	postive
ALOX12	Z83843.1	0.407655983	5.37E-23	postive
IREB2	Z83843.1	0.441259491	4.33E-27	postive
GABPB1	Z83843.1	0.618294648	3.72E-58	postive
PIK3CA	Z83843.1	0.459898046	1.45E-29	postive
ATG7	Z83843.1	0.412315427	1.55E-23	postive
MAPK8	Z83843.1	0.456014074	4.90E-29	postive
LINC00472	Z83843.1	0.865668978	1.71E-163	postive
ATM	Z83843.1	0.700943717	7.42E-81	postive
FBXW7	Z83843.1	0.482495456	8.88E-33	postive
HELLS	AC007622.2	0.513006808	1.63E-37	postive
ZNF419	AC007622.2	0.450716397	2.51E-28	postive
KLHL24	AC007622.2	0.469702241	6.26E-31	postive
TUBE1	AC007622.2	0.447550675	6.58E-28	postive
ALOX12	AC007622.2	0.488598716	1.09E-33	postive

IREB2	AC007622.2	0.425597429	4.00E-25	postive
GABPB1	AC007622.2	0.522964908	3.62E-39	postive
MAPK8	AC007622.2	0.406260547	7.76E-23	postive
LINC00472	AC007622.2	0.780974656	7.38E-112	postive
LPIN1	AC007622.2	0.470181264	5.36E-31	postive
ATM	AC007622.2	0.575467017	7.48E-49	postive
FBXW7	AC007622.2	0.460742005	1.11E-29	postive
PHKG2	AC131159.2	0.4292516	1.42E-25	postive
BECN1	AC131159.2	-0.472629861	2.40E-31	negative
ISCU	AC055720.2	0.663739903	9.72E-70	postive
DDIT3	AC055720.2	0.522479513	4.38E-39	postive
HERPUD1	AC055720.2	0.448695588	4.65E-28	postive
SLC2A12	AC055720.2	0.501346987	1.20E-35	postive
CS	AC055720.2	0.404594898	1.20E-22	postive
GOT1	AC055720.2	0.415258879	6.98E-24	postive
ATG4D	AC055720.2	0.51532822	6.78E-38	postive
MAP1LC3A	AC055720.2	0.454315911	8.30E-29	postive
GABARAPL1	AC055720.2	0.669668743	2.11E-71	postive
WIPI2	AC055720.2	0.549143308	8.79E-44	postive
BAP1	AC055720.2	0.448638516	4.73E-28	postive
LPIN1	AC055720.2	0.606792298	1.62E-55	postive
KLHL24	AC037198.1	0.412722719	1.39E-23	postive
GABPB1	AC037198.1	0.457019059	3.58E-29	postive
LINC00472	AC037198.1	0.782580094	1.30E-112	postive
HELLS	AP000786.1	0.440700174	5.12E-27	postive
KLHL24	AP000786.1	0.455754414	5.31E-29	postive
TUBE1	AP000786.1	0.478938796	2.95E-32	postive
ALOX12	AP000786.1	0.426215224	3.36E-25	postive
IREB2	AP000786.1	0.484942885	3.85E-33	postive
GABPB1	AP000786.1	0.559832574	8.71E-46	postive
PIK3CA	AP000786.1	0.466190168	1.95E-30	postive
MAPK8	AP000786.1	0.499746454	2.13E-35	postive
LINC00472	AP000786.1	0.783826915	3.35E-113	postive
ATM	AP000786.1	0.714684673	2.07E-85	postive
FBXW7	AP000786.1	0.462381569	6.61E-30	postive
HELLS	DHRSX-IT1	0.457202024	3.38E-29	postive
ALOX12	DHRSX-IT1	0.486127452	2.57E-33	postive
GABPB1	DHRSX-IT1	0.415446323	6.63E-24	postive
LINC00472	DHRSX-IT1	0.450402621	2.77E-28	postive
ATM	DHRSX-IT1	0.577696529	2.65E-49	postive
BRD4	AL359921.1	0.428161153	1.94E-25	postive
ZNF419	AL359921.1	0.504241348	4.18E-36	postive
VEGFA	AL359921.1	0.483601535	6.09E-33	postive
TUBE1	AL359921.1	0.556308441	4.06E-45	postive
SETD1B	AL359921.1	0.542595842	1.37E-42	postive
ALOX12	AL359921.1	0.703668343	9.72E-82	postive
ATM	AL359921.1	0.479913886	2.13E-32	postive
YY1AP1	AL359921.1	0.415860304	5.93E-24	postive
TAZ	AL359921.1	0.503316446	5.86E-36	postive
NOX1	AP001330.4	0.601133918	2.93E-54	postive
BECN1	AP001330.4	-0.401741957	2.53E-22	negative
RB1	USP46-DT	0.40738691	5.77E-23	postive
GCLC	USP46-DT	0.409005127	3.75E-23	postive
MTOR	USP46-DT	0.456947413	3.66E-29	postive
RPL8	USP46-DT	-0.40347578	1.61E-22	negative
MAPK14	USP46-DT	0.471938356	3.01E-31	postive
IREB2	USP46-DT	0.53314528	6.49E-41	postive
PIK3CA	USP46-DT	0.405321124	9.94E-23	postive

KRAS	USP46-DT	0.433265896	4.49E-26	postive
NCOA4	USP46-DT	0.41611185	5.53E-24	postive
SNX4	USP46-DT	0.436792384	1.61E-26	postive
MAPK8	USP46-DT	0.482161925	9.94E-33	postive
PRKAA2	USP46-DT	0.419889364	1.96E-24	postive
HELLS	AL139288.1	0.413687335	1.07E-23	postive
ALOX12	AL139288.1	0.41315018	1.24E-23	postive
TFAP2C	AL139288.1	0.427807499	2.14E-25	postive
DUOX1	AL139288.1	0.481809333	1.12E-32	postive
HSPB1	AC009309.1	0.405764	8.85E-23	postive
HBA1	AC009309.1	0.64473229	1.18E-64	postive
HRAS	AC009309.1	0.630292165	5.00E-61	postive
MAP1LC3A	AC009309.1	0.4840548	5.22E-33	postive
EGLN2	AC009309.1	0.780060735	1.97E-111	postive
FH	AL161668.4	0.545911734	3.44E-43	postive
ISCU	AL161668.4	0.531798917	1.11E-40	postive
ALB	AL161668.4	0.646089939	5.26E-65	postive
GPX2	AL161668.4	0.485025216	3.75E-33	postive
TFR2	AL161668.4	0.493909178	1.71E-34	postive
GOT1	AL161668.4	0.499848396	2.06E-35	postive
ATG4D	AL161668.4	0.427014786	2.68E-25	postive
GABARAPL2	AL161668.4	0.495234647	1.07E-34	postive
GABARAPL1	AL161668.4	0.476847126	5.94E-32	postive
CDO1	AL161668.4	0.608401154	7.01E-56	postive
ZNF419	AL606760.2	0.476924395	5.79E-32	postive
TUBE1	AL606760.2	0.550743727	4.45E-44	postive
ALOX12	AL606760.2	0.641646143	7.31E-64	postive
TAZ	AL606760.2	0.488240552	1.24E-33	postive
HELLS	AL031667.3	0.473196185	1.99E-31	postive
KLHL24	AL031667.3	0.506074227	2.14E-36	postive
GABPB1	AL031667.3	0.57192794	3.82E-48	postive
PIK3CA	AL031667.3	0.432896884	5.00E-26	postive
LINC00472	AL031667.3	0.812994785	3.15E-128	postive
ATM	AL031667.3	0.625054566	9.29E-60	postive
FBXW7	AL031667.3	0.488110336	1.29E-33	postive
HELLS	AC016405.1	0.502292274	8.50E-36	postive
GABPB1	AC016405.1	0.459414055	1.69E-29	postive
LINC00472	AC016405.1	0.527187948	6.95E-40	postive
ATM	AC016405.1	0.644408484	1.43E-64	postive
FBXW7	AC016405.1	0.420344395	1.73E-24	postive
TAZ	PAXIP1-DT	0.47532746	9.85E-32	postive
HELLS	AC087277.2	0.575678467	6.78E-49	postive
TUBE1	AC087277.2	0.436874853	1.58E-26	postive
ALOX12	AC087277.2	0.432370904	5.82E-26	postive
GABPB1	AC087277.2	0.538820694	6.49E-42	postive
ATG7	AC087277.2	0.451708555	1.85E-28	postive
LINC00472	AC087277.2	0.575243877	8.29E-49	postive
ATM	AC087277.2	0.720030664	2.95E-87	postive
FBXW7	AC087277.2	0.513584332	1.31E-37	postive
TRIB3	AC156455.1	0.426046441	3.52E-25	postive
TAZ	AC156455.1	0.408548092	4.24E-23	postive
ZNF419	LINC00106	0.476989885	5.67E-32	postive
TUBE1	LINC00106	0.443094936	2.51E-27	postive
SETD1B	LINC00106	0.507006006	1.52E-36	postive
ALOX12	LINC00106	0.586050695	5.04E-51	postive
PHKG2	LINC00106	0.452969149	1.26E-28	postive
TAZ	LINC00106	0.543851832	8.13E-43	postive
GCLC	DNAJC3-DT	0.407255278	5.97E-23	postive

ACSL3	DNAJC3-DT	0.467361473	1.34E-30	postive
KLHL24	DNAJC3-DT	0.538940442	6.18E-42	postive
IREB2	DNAJC3-DT	0.457631822	2.96E-29	postive
GABPB1	DNAJC3-DT	0.44049415	5.44E-27	postive
PIK3CA	DNAJC3-DT	0.400682491	3.33E-22	postive
GABARAPL1	DNAJC3-DT	0.408375618	4.44E-23	postive
MAPK8	DNAJC3-DT	0.401460633	2.73E-22	postive
LINC00472	DNAJC3-DT	0.521172354	7.26E-39	postive
LPIN1	DNAJC3-DT	0.477140743	5.39E-32	postive
HSPA5	RAD51-AS1	-0.431747362	6.96E-26	negative
ZNF419	RAD51-AS1	0.648968027	9.34E-66	postive
VEGFA	RAD51-AS1	0.498326633	3.55E-35	postive
TUBE1	RAD51-AS1	0.680930962	1.14E-74	postive
SETD1B	RAD51-AS1	0.492577533	2.73E-34	postive
ALOX12	RAD51-AS1	0.695865596	3.08E-79	postive
GABPB1	RAD51-AS1	0.444773291	1.52E-27	postive
PHKG2	RAD51-AS1	0.429820721	1.21E-25	postive
ATM	RAD51-AS1	0.429105055	1.48E-25	postive
TAZ	RAD51-AS1	0.602708917	1.32E-54	postive
FBXW7	RAD51-AS1	0.409921307	2.94E-23	postive
NCF2	MIR223HG	0.41377375	1.04E-23	postive
BRD4	AL049840.5	0.488493259	1.13E-33	postive
ZNF419	AL049840.5	0.595225312	5.69E-53	postive
VEGFA	AL049840.5	0.439576853	7.13E-27	postive
TUBE1	AL049840.5	0.455359326	6.00E-29	postive
SETD1B	AL049840.5	0.4189017	2.58E-24	postive
ALOX12	AL049840.5	0.459168886	1.82E-29	postive
GABPB1	AL049840.5	0.520548402	9.23E-39	postive
EGLN2	AL049840.5	0.517085746	3.48E-38	postive
TAZ	AL049840.5	0.523377702	3.09E-39	postive
FBXW7	AL049840.5	0.512630655	1.87E-37	postive
TFAP2C	IDH2-DT	0.661392267	4.32E-69	postive
HBA1	IDH2-DT	0.534247829	4.16E-41	postive
NCF2	AC011899.2	0.508304816	9.40E-37	postive
FTL	AC011899.2	0.427907725	2.08E-25	postive
HAMP	AC011899.2	0.432893667	5.00E-26	postive
ALOX5	AC011899.2	0.414244874	9.19E-24	postive
IFNG	AC011899.2	0.416224566	5.37E-24	postive
HELLS	AC090517.2	0.474489378	1.30E-31	postive
TUBE1	AC090517.2	0.547178493	2.02E-43	postive
ALOX12	AC090517.2	0.422300414	1.01E-24	postive
GABPB1	AC090517.2	0.556208655	4.24E-45	postive
LINC00472	AC090517.2	0.494114958	1.59E-34	postive
ATM	AC090517.2	0.543752124	8.47E-43	postive
HELLS	MAP3K5-AS1	0.465092465	2.78E-30	postive
KLHL24	MAP3K5-AS1	0.409230835	3.53E-23	postive
GABPB1	MAP3K5-AS1	0.520192141	1.06E-38	postive
LINC00472	MAP3K5-AS1	0.818356866	2.80E-131	postive
ATM	MAP3K5-AS1	0.562953605	2.19E-46	postive
FBXW7	MAP3K5-AS1	0.445389028	1.26E-27	postive
HELLS	AP001271.1	0.400605973	3.40E-22	postive
ALOX12	AP001271.1	0.500963532	1.38E-35	postive
ATM	AP001271.1	0.57221232	3.35E-48	postive
TXNRD1	AP003119.3	0.420766422	1.54E-24	postive
KLHL24	ARHGAP31-/	0.468567029	9.06E-31	postive
ALOX12	ARHGAP31-/	0.413810726	1.03E-23	postive
IREB2	ARHGAP31-/	0.453011417	1.24E-28	postive
GABPB1	ARHGAP31-/	0.41760026	3.68E-24	postive

PIK3CA	ARHGAP31-/	0.444768251	1.52E-27	postive
MAPK8	ARHGAP31-/	0.457703163	2.89E-29	postive
LINC00472	ARHGAP31-/	0.606988463	1.46E-55	postive
ATM	ARHGAP31-/	0.583133945	2.04E-50	postive
FANCD2	AC012073.1	0.500469872	1.64E-35	postive
HELLS	AC012073.1	0.568702344	1.66E-47	postive
PML	AC012073.1	0.417082901	4.24E-24	postive
BRD4	AC012073.1	0.435507545	2.35E-26	postive
SETD1B	AC012073.1	0.405994548	8.33E-23	postive
ALOX12	AC012073.1	0.44371454	2.09E-27	postive
TAZ	AC012073.1	0.486421724	2.32E-33	postive
GPX4	YTHDF3-AS1	0.52420704	2.23E-39	postive
HSPB1	YTHDF3-AS1	0.437025804	1.51E-26	postive
HSF1	YTHDF3-AS1	0.457662458	2.93E-29	postive
OTUB1	YTHDF3-AS1	0.541774826	1.92E-42	postive
CHMP6	YTHDF3-AS1	0.42774652	2.18E-25	postive
RPL8	YTHDF3-AS1	0.497600473	4.60E-35	postive
SLC2A8	YTHDF3-AS1	0.424440008	5.54E-25	postive
HRAS	YTHDF3-AS1	0.565106003	8.39E-47	postive
PHKG2	YTHDF3-AS1	0.416515681	4.96E-24	postive
KLHL24	AC016831.4	0.568854495	1.55E-47	postive
GABPB1	AC016831.4	0.420670286	1.58E-24	postive
PIK3CA	AC016831.4	0.466903251	1.55E-30	postive
KRAS	AC016831.4	0.410277451	2.67E-23	postive
LINC00472	AC016831.4	0.951266836	1.55E-276	postive
ZNF419	ZNF436-AS1	0.400621707	3.39E-22	postive
ALOX12	ZNF436-AS1	0.450823476	2.43E-28	postive
PHKG2	ZNF436-AS1	0.542833491	1.24E-42	postive
TAZ	ZNF436-AS1	0.621326215	7.19E-59	postive
HELLS	AC024060.2	0.430561119	9.78E-26	postive
ZNF419	AC024060.2	0.596793553	2.61E-53	postive
TUBE1	AC024060.2	0.567523398	2.83E-47	postive
ALOX12	AC024060.2	0.432267394	5.99E-26	postive
GABPB1	AC024060.2	0.534214115	4.22E-41	postive
PHKG2	AC024060.2	0.555864807	4.92E-45	postive
LINC00472	AC024060.2	0.427477937	2.35E-25	postive
TAZ	AC024060.2	0.639925838	2.00E-63	postive
FBXW7	AC024060.2	0.517964598	2.49E-38	postive
BRD4	AC006435.2	0.473228743	1.97E-31	postive
ZNF419	AC006435.2	0.591337875	3.87E-52	postive
TUBE1	AC006435.2	0.54885483	9.94E-44	postive
SETD1B	AC006435.2	0.520198709	1.06E-38	postive
DRD4	AC006435.2	0.404678037	1.18E-22	postive
ALOX12	AC006435.2	0.600808931	3.46E-54	postive
PHKG2	AC006435.2	0.527750277	5.56E-40	postive
TAZ	AC006435.2	0.649180218	8.22E-66	postive
FBXW7	AC006435.2	0.417893529	3.40E-24	postive
PHKG2	AC018904.1	0.473051565	2.09E-31	postive
BID	AC018904.1	0.404947377	1.10E-22	postive
TAZ	AC018904.1	0.404592197	1.20E-22	postive
HELLS	AL513008.1	0.51825607	2.23E-38	postive
TUBE1	AL513008.1	0.406147296	8.00E-23	postive
ALOX12	AL513008.1	0.429901964	1.18E-25	postive
GABPB1	AL513008.1	0.477674858	4.51E-32	postive
ATG7	AL513008.1	0.446226428	9.82E-28	postive
LINC00472	AL513008.1	0.455833871	5.18E-29	postive
ATM	AL513008.1	0.686740585	2.05E-76	postive
FBXW7	AL513008.1	0.417279467	4.02E-24	postive

TFAP2C	AP003555.1	0.580840976	6.04E-50	postive
HBA1	AP003555.1	0.534609126	3.60E-41	postive
DUOX1	AP003555.1	0.400345392	3.64E-22	postive
ZNF419	AC007686.3	0.560304946	7.07E-46	postive
TUBE1	AC007686.3	0.470167067	5.38E-31	postive
ALOX12	AC007686.3	0.603712568	7.88E-55	postive
TAZ	AC007686.3	0.432828018	5.10E-26	postive
HSPB1	AL731577.2	-0.451302793	2.10E-28	negative
STAT3	AL731577.2	0.471401147	3.60E-31	postive
MTOR	AL731577.2	0.542110775	1.67E-42	postive
OXSR1	AL731577.2	0.477906017	4.17E-32	postive
MAPK14	AL731577.2	0.482269973	9.58E-33	postive
EIF2AK4	AL731577.2	0.421411837	1.29E-24	postive
IREB2	AL731577.2	0.570755961	6.53E-48	postive
NOX1	AL731577.2	-0.472431831	2.56E-31	negative
PIK3CA	AL731577.2	0.526923714	7.71E-40	postive
NRAS	AL731577.2	0.417327417	3.97E-24	postive
NCOA4	AL731577.2	0.548364358	1.22E-43	postive
PHKG2	AL731577.2	-0.483910364	5.48E-33	negative
BECN1	AL731577.2	0.521277043	6.97E-39	postive
MAPK1	AL731577.2	0.466145692	1.98E-30	postive
ZEB1	AL731577.2	0.419887343	1.96E-24	postive
MAPK8	AL731577.2	0.460663039	1.14E-29	postive
PRKAA2	AL731577.2	0.44562889	1.18E-27	postive
PRKAA1	AL731577.2	0.404623094	1.19E-22	postive
EPAS1	AL731577.2	0.417108188	4.22E-24	postive
TLR4	AL731577.2	0.468788684	8.43E-31	postive
TAZ	AL731577.2	-0.417373998	3.92E-24	negative
SIRT1	AL731577.2	0.520139653	1.08E-38	postive
HELLS	UBE2Q1-AS1	0.430101084	1.12E-25	postive
BRD4	UBE2Q1-AS1	0.412938017	1.31E-23	postive
ZNF419	UBE2Q1-AS1	0.531709538	1.15E-40	postive
VEGFA	UBE2Q1-AS1	0.443353045	2.33E-27	postive
TUBE1	UBE2Q1-AS1	0.622998883	2.88E-59	postive
SETD1B	UBE2Q1-AS1	0.515388303	6.63E-38	postive
ALOX12	UBE2Q1-AS1	0.705244791	2.97E-82	postive
GABPB1	UBE2Q1-AS1	0.437781606	1.21E-26	postive
ATM	UBE2Q1-AS1	0.566364742	4.77E-47	postive
YY1AP1	UBE2Q1-AS1	0.458804576	2.05E-29	postive
TAZ	UBE2Q1-AS1	0.487922158	1.38E-33	postive
FBXW7	UBE2Q1-AS1	0.476336411	7.05E-32	postive
TUBE1	LIFR-AS1	0.443900076	1.97E-27	postive
MAPK8	LIFR-AS1	0.438153692	1.08E-26	postive
PRKAA2	LIFR-AS1	0.432432977	5.71E-26	postive
ATM	LIFR-AS1	0.402240546	2.23E-22	postive
ISCU	AL356740.1	0.481034209	1.46E-32	postive
ACSL3	AL356740.1	0.435424113	2.40E-26	postive
KLHL24	AL356740.1	0.424958622	4.79E-25	postive
SLC2A12	AL356740.1	0.416840292	4.54E-24	postive
ATG4D	AL356740.1	0.495212397	1.08E-34	postive
GABARAPL1	AL356740.1	0.631855811	2.07E-61	postive
WIPI2	AL356740.1	0.425392904	4.24E-25	postive
LPIN1	AL356740.1	0.657591515	4.71E-68	postive
HELLS	FLJ31104	0.410529778	2.50E-23	postive
TUBE1	FLJ31104	0.502502401	7.88E-36	postive
ALOX12	FLJ31104	0.52629438	9.87E-40	postive
TAZ	FLJ31104	0.450140312	3.00E-28	postive
HELLS	AC091185.1	0.4414693	4.07E-27	postive

ZNF419	AC091185.1	0.516850237	3.80E-38	postive
VEGFA	AC091185.1	0.464414689	3.45E-30	postive
TUBE1	AC091185.1	0.651888063	1.59E-66	postive
SETD1B	AC091185.1	0.466823495	1.59E-30	postive
ALOX12	AC091185.1	0.695705401	3.46E-79	postive
GABPB1	AC091185.1	0.50635963	1.93E-36	postive
ATM	AC091185.1	0.667758706	7.31E-71	postive
YY1AP1	AC091185.1	0.400961162	3.10E-22	postive
FBXW7	AC091185.1	0.46455804	3.30E-30	postive
ZNF419	AC005393.1	0.447638157	6.41E-28	postive
PHKG2	AC005393.1	0.587156945	2.96E-51	postive
TAZ	AC005393.1	0.657557943	4.81E-68	postive
TF	LINC01151	0.599472083	6.80E-54	postive
ALOX12	HDHD5-AS1	0.409037674	3.72E-23	postive
HBA1	HDHD5-AS1	0.432420075	5.74E-26	postive
ALOX12B	HDHD5-AS1	0.461772777	8.02E-30	postive
FANCD2	RHOA-IT1	0.423382789	7.44E-25	postive
HELLS	RHOA-IT1	0.479686028	2.30E-32	postive
ALOX12	RHOA-IT1	0.532856889	7.29E-41	postive
TFAP2C	RHOA-IT1	0.452080765	1.65E-28	postive
HBA1	RHOA-IT1	0.407617168	5.43E-23	postive
GABPB1	RHOA-IT1	0.402962265	1.84E-22	postive
DUOX1	RHOA-IT1	0.442905846	2.66E-27	postive
ATM	RHOA-IT1	0.417563533	3.72E-24	postive
FBXW7	RHOA-IT1	0.405535462	9.40E-23	postive
KLHL24	CARMN	0.486322386	2.40E-33	postive
GABPB1	CARMN	0.493072328	2.29E-34	postive
PIK3CA	CARMN	0.424157965	5.99E-25	postive
LINC00472	CARMN	0.870056181	4.30E-167	postive
ATM	CARMN	0.522401838	4.51E-39	postive
HELLS	AL512656.1	0.538589634	7.14E-42	postive
TUBE1	AL512656.1	0.498192381	3.72E-35	postive
ALOX12	AL512656.1	0.522409356	4.50E-39	postive
GABPB1	AL512656.1	0.542208937	1.61E-42	postive
MAPK8	AL512656.1	0.425673993	3.91E-25	postive
LINC00472	AL512656.1	0.676075314	3.04E-73	postive
ATM	AL512656.1	0.703944092	7.91E-82	postive
FBXW7	AL512656.1	0.494540919	1.37E-34	postive
ZNF419	AC021321.1	0.483660548	5.97E-33	postive
KLHL24	AC021321.1	0.516335673	4.63E-38	postive
GABPB1	AC021321.1	0.490537777	5.57E-34	postive
LINC00472	AC021321.1	0.782639901	1.22E-112	postive
LPIN1	AC021321.1	0.508258998	9.56E-37	postive
HELLS	AL020997.2	0.496621797	6.52E-35	postive
TUBE1	AL020997.2	0.415741457	6.12E-24	postive
GABPB1	AL020997.2	0.535465708	2.55E-41	postive
LINC00472	AL020997.2	0.705883595	1.83E-82	postive
ATM	AL020997.2	0.649260102	7.83E-66	postive
FBXW7	AL020997.2	0.474349372	1.36E-31	postive
ALOX12	SEC24B-AS1	0.402078812	2.32E-22	postive
GABPB1	SEC24B-AS1	0.427082944	2.63E-25	postive
VEGFA	AL353803.1	0.466635531	1.69E-30	postive
ALOX12	AL353803.1	0.413933292	1.00E-23	postive
TUBE1	THAP9-AS1	0.437423392	1.34E-26	postive
TAZ	THAP9-AS1	0.411322744	2.02E-23	postive
ALOX12	AL355312.2	0.456053039	4.84E-29	postive
TFAP2C	AL355312.2	0.402197586	2.25E-22	postive
HELLS	AC007216.2	0.433476552	4.23E-26	postive

ALOX12	AC007216.2	0.401197744	2.92E-22	postive
TFAP2C	AC007216.2	0.471617064	3.35E-31	postive
HBA1	AC007216.2	0.42768276	2.22E-25	postive
DUOX1	AC007216.2	0.422979106	8.33E-25	postive
LINC00472	AC007216.2	0.519647118	1.31E-38	postive
ALOX12	AP006621.3	0.491093902	4.59E-34	postive
PHKG2	AP006621.3	0.578993526	1.44E-49	postive
TAZ	AP006621.3	0.673967266	1.24E-72	postive
SLC40A1	LINC01094	0.402660046	2.00E-22	postive
NCF2	LINC01094	0.610862852	1.94E-56	postive
HAMP	LINC01094	0.499031455	2.76E-35	postive
CYBB	LINC01094	0.782684982	1.16E-112	postive
ATG7	LINC01094	0.477412112	4.92E-32	postive
TGFBR1	LINC01094	0.401713533	2.55E-22	postive
TLR4	LINC01094	0.47516362	1.04E-31	postive
MTDH	LINC01094	0.420627979	1.60E-24	postive
IDH1	LINC01094	0.465627366	2.34E-30	postive
BACH1	LINC01094	0.505565147	2.58E-36	postive
ZNF419	AC127024.6	0.493506677	1.97E-34	postive
TUBE1	AC127024.6	0.507503905	1.26E-36	postive
ALOX12	AC127024.6	0.47856539	3.35E-32	postive
GABPB1	AC127024.6	0.450573549	2.62E-28	postive
PHKG2	AC127024.6	0.454988076	6.74E-29	postive
TAZ	AC127024.6	0.569606817	1.10E-47	postive
FBXW7	AC127024.6	0.406592323	7.11E-23	postive
PML	IL10RB-DT	0.484386812	4.66E-33	postive
ALOX5	IL10RB-DT	0.447952303	5.83E-28	postive
ZNF419	AC105020.6	0.493851889	1.74E-34	postive
VEGFA	AC105020.6	0.441355551	4.21E-27	postive
TUBE1	AC105020.6	0.524610763	1.91E-39	postive
SETD1B	AC105020.6	0.416143633	5.49E-24	postive
ALOX12	AC105020.6	0.659082084	1.85E-68	postive
TAZ	AC105020.6	0.559646793	9.45E-46	postive
NCF2	LINC01857	0.532270928	9.22E-41	postive
FTL	LINC01857	0.614678778	2.58E-57	postive
CAPG	LINC01857	0.453015808	1.24E-28	postive
ALOX15B	LINC01857	0.461520813	8.68E-30	postive
ALOX12	SGMS1-AS1	0.431960749	6.55E-26	postive
IREB2	SGMS1-AS1	0.433490122	4.21E-26	postive
SP1	SGMS1-AS1	0.4464411	9.21E-28	postive
MAPK1	SGMS1-AS1	0.427781959	2.16E-25	postive
MAPK8	SGMS1-AS1	0.535638388	2.37E-41	postive
PRKAA2	SGMS1-AS1	0.5374252	1.15E-41	postive
ATM	SGMS1-AS1	0.519793464	1.23E-38	postive
SIRT1	SGMS1-AS1	0.470074567	5.55E-31	postive
JDP2	LINC01560	0.459030832	1.91E-29	postive
ZNF419	LINC01560	0.449023659	4.21E-28	postive
EIF2S1	LINC01560	0.489521601	7.93E-34	postive
GABPB1	LINC01560	0.51812159	2.34E-38	postive
HELLS	AC137932.1	0.445580176	1.19E-27	postive
ZNF419	AC137932.1	0.425971948	3.60E-25	postive
TUBE1	AC137932.1	0.517138257	3.41E-38	postive
SETD1B	AC137932.1	0.430780468	9.18E-26	postive
ALOX12	AC137932.1	0.622684653	3.42E-59	postive
GABPB1	AC137932.1	0.414225855	9.24E-24	postive
ATM	AC137932.1	0.640955897	1.10E-63	postive
FBXW7	AC137932.1	0.43677495	1.62E-26	postive
ALOX12	PPP1R12A-A	0.431019888	8.57E-26	postive

TFAP2C	PPP1R12A-A	0.415475412	6.58E-24	postive
HSPB1	AC083880.1	0.499904926	2.01E-35	postive
HRAS	AC083880.1	0.408234086	4.61E-23	postive
PHKG2	AC083880.1	0.487771475	1.46E-33	postive
TAZ	AC083880.1	0.415342635	6.82E-24	postive
LONP1	AC083880.1	0.417482301	3.81E-24	postive
HELLS	AL132780.1	0.44822067	5.37E-28	postive
ZNF419	AL132780.1	0.533835447	4.92E-41	postive
TUBE1	AL132780.1	0.494088254	1.60E-34	postive
ALOX12	AL132780.1	0.566145893	5.26E-47	postive
GABPB1	AL132780.1	0.430767229	9.22E-26	postive
LINC00472	AL132780.1	0.407655585	5.37E-23	postive
LPIN1	AL132780.1	0.438688583	9.26E-27	postive
ATM	AL132780.1	0.475457439	9.44E-32	postive
FBXW7	AL132780.1	0.42502016	4.70E-25	postive
HELLS	PPP3CB-AS1	0.512327819	2.10E-37	postive
ZNF419	PPP3CB-AS1	0.470584148	4.70E-31	postive
TUBE1	PPP3CB-AS1	0.596688239	2.75E-53	postive
ALOX12	PPP3CB-AS1	0.620804608	9.55E-59	postive
GABPB1	PPP3CB-AS1	0.554441163	9.12E-45	postive
MAPK8	PPP3CB-AS1	0.494595514	1.34E-34	postive
ATM	PPP3CB-AS1	0.646552316	3.99E-65	postive
FBXW7	PPP3CB-AS1	0.491206215	4.41E-34	postive
BRD4	AL117209.1	0.451328192	2.08E-28	postive
ZNF419	AL117209.1	0.573945088	1.51E-48	postive
VEGFA	AL117209.1	0.412005262	1.68E-23	postive
TUBE1	AL117209.1	0.495589894	9.41E-35	postive
SETD1B	AL117209.1	0.469022639	7.81E-31	postive
DRD4	AL117209.1	0.415434037	6.65E-24	postive
ALOX12	AL117209.1	0.550756173	4.43E-44	postive
PHKG2	AL117209.1	0.432499385	5.61E-26	postive
TAZ	AL117209.1	0.587795904	2.17E-51	postive
FANCD2	AC008105.2	0.558360508	1.66E-45	postive
HELLS	AC008105.2	0.40961566	3.19E-23	postive
ALOX12	AC008105.2	0.516531665	4.29E-38	postive
PHKG2	AC008105.2	0.522380812	4.55E-39	postive
SAT1	AC008105.2	0.430257491	1.07E-25	postive
IFNG	AC008105.2	0.462480041	6.40E-30	postive
TAZ	AC008105.2	0.570216889	8.35E-48	postive
ISCU	AC005696.1	0.445418382	1.25E-27	postive
ATG4D	AC005696.1	0.562794369	2.35E-46	postive
GABARAPL1	AC005696.1	0.430257763	1.07E-25	postive
BAP1	AC005696.1	0.427180113	2.56E-25	postive
TFAP2C	ZNF687-AS1	0.579730173	1.02E-49	postive
HBA1	ZNF687-AS1	0.522250763	4.78E-39	postive
IL6	AC092376.2	0.506061366	2.15E-36	postive
SLC2A3	AC092376.2	0.454383999	8.12E-29	postive
EIF2AK4	BAALC-AS1	0.464709999	3.14E-30	postive
ALB	AP001107.9	0.401315967	2.83E-22	postive
TFAP2C	AP001107.9	0.512596772	1.90E-37	postive
CDO1	AP001107.9	0.431596534	7.27E-26	postive
PHKG2	AC009097.2	0.544675078	5.76E-43	postive
TAZ	AC009097.2	0.556486142	3.76E-45	postive
KLHL24	AC090236.2	0.540141097	3.78E-42	postive
LINC00472	AC090236.2	0.799227923	7.95E-121	postive
LPIN1	AC090236.2	0.438053463	1.12E-26	postive
HSF1	AL158151.4	0.434305011	3.33E-26	postive
NCOA4	AL158151.4	-0.421945475	1.11E-24	negative

PHKG2	AL158151.4	0.598160062	1.31E-53	postive
TAZ	AL158151.4	0.58156964	4.28E-50	postive
HELLS	AL731563.3	0.563798131	1.50E-46	postive
ZNF419	AL731563.3	0.469183133	7.42E-31	postive
TUBE1	AL731563.3	0.523568423	2.87E-39	postive
ALOX12	AL731563.3	0.645960757	5.68E-65	postive
GABPB1	AL731563.3	0.497257706	5.20E-35	postive
DUOX1	AL731563.3	0.407773674	5.20E-23	postive
LINC00472	AL731563.3	0.435897095	2.10E-26	postive
ATM	AL731563.3	0.556328552	4.03E-45	postive
FBXW7	AL731563.3	0.486522931	2.24E-33	postive
HELLS	AC008781.2	0.540492204	3.27E-42	postive
ALOX12	AC008781.2	0.49440076	1.43E-34	postive
GABPB1	AC008781.2	0.415034364	7.42E-24	postive
ATM	AC008781.2	0.568628452	1.72E-47	postive
FBXW7	AC008781.2	0.425323967	4.32E-25	postive
FANCD2	LINC01357	0.498172894	3.75E-35	postive
HBA1	LINC01357	0.412008649	1.68E-23	postive
IFNG	LINC01357	0.414224672	9.24E-24	postive
HELLS	YEATS2-AS1	0.462863058	5.67E-30	postive
BRD4	YEATS2-AS1	0.446981143	7.82E-28	postive
ZNF419	YEATS2-AS1	0.552218536	2.37E-44	postive
VEGFA	YEATS2-AS1	0.462028813	7.39E-30	postive
TUBE1	YEATS2-AS1	0.529171423	3.17E-40	postive
SETD1B	YEATS2-AS1	0.455840414	5.17E-29	postive
ALOX12	YEATS2-AS1	0.659668857	1.28E-68	postive
GABPB1	YEATS2-AS1	0.402738315	1.95E-22	postive
ATM	YEATS2-AS1	0.481545395	1.23E-32	postive
TAZ	YEATS2-AS1	0.516822003	3.85E-38	postive
FBXW7	YEATS2-AS1	0.453097562	1.21E-28	postive
TAZ	AC004069.1	0.555122409	6.79E-45	postive
ATF4	AL118516.1	0.536520712	1.66E-41	postive
PHKG2	AL118516.1	0.402662133	1.99E-22	postive
TAZ	AL118516.1	0.570331517	7.92E-48	postive
HELLS	PABPC4-AS1	0.451486816	1.98E-28	postive
KLHL24	PABPC4-AS1	0.512416773	2.03E-37	postive
TUBE1	PABPC4-AS1	0.43636453	1.83E-26	postive
IREB2	PABPC4-AS1	0.465014896	2.85E-30	postive
GABPB1	PABPC4-AS1	0.559320076	1.09E-45	postive
PIK3CA	PABPC4-AS1	0.475243463	1.01E-31	postive
KRAS	PABPC4-AS1	0.410803818	2.32E-23	postive
MAPK8	PABPC4-AS1	0.44937432	3.78E-28	postive
LINC00472	PABPC4-AS1	0.906837711	1.21E-203	postive
ATM	PABPC4-AS1	0.663858072	9.01E-70	postive
FBXW7	PABPC4-AS1	0.444708677	1.55E-27	postive
HELLS	DNM3OS	0.433588063	4.09E-26	postive
ZNF419	DNM3OS	0.486750582	2.07E-33	postive
ZFP69B	DNM3OS	0.445589969	1.19E-27	postive
GABPB1	DNM3OS	0.591692229	3.25E-52	postive
ZEB1	DNM3OS	0.509883141	5.23E-37	postive
FBXW7	DNM3OS	0.419139603	2.41E-24	postive
HELLS	PSMD6-AS2	0.540650508	3.06E-42	postive
ZNF419	PSMD6-AS2	0.439716805	6.84E-27	postive
KLHL24	PSMD6-AS2	0.455464153	5.81E-29	postive
TUBE1	PSMD6-AS2	0.518824309	1.79E-38	postive
ALOX12	PSMD6-AS2	0.506892312	1.58E-36	postive
IREB2	PSMD6-AS2	0.461786409	7.98E-30	postive
GABPB1	PSMD6-AS2	0.61718866	6.74E-58	postive

PIK3CA	PSMD6-AS2	0.44688661	8.05E-28	postive
ATG7	PSMD6-AS2	0.450655555	2.56E-28	postive
MAPK8	PSMD6-AS2	0.474001391	1.53E-31	postive
LINC00472	PSMD6-AS2	0.792350306	2.43E-117	postive
ATM	PSMD6-AS2	0.77387828	1.33E-108	postive
FBXW7	PSMD6-AS2	0.540915566	2.74E-42	postive
HELLS	AC114760.2	0.584863442	8.92E-51	postive
TUBE1	AC114760.2	0.429787633	1.22E-25	postive
ALOX12	AC114760.2	0.455050431	6.61E-29	postive
GABPB1	AC114760.2	0.559693017	9.26E-46	postive
ATG7	AC114760.2	0.471185144	3.86E-31	postive
LINC00472	AC114760.2	0.67300698	2.35E-72	postive
ATM	AC114760.2	0.701458407	5.06E-81	postive
FBXW7	AC114760.2	0.488783438	1.03E-33	postive
HELLS	MIRLET7BHG	0.540368628	3.44E-42	postive
ZNF419	MIRLET7BHG	0.459522432	1.63E-29	postive
ALOX12	MIRLET7BHG	0.470019254	5.65E-31	postive
GABPB1	MIRLET7BHG	0.512288531	2.13E-37	postive
LINC00472	MIRLET7BHG	0.517755363	2.69E-38	postive
ATM	MIRLET7BHG	0.543683479	8.72E-43	postive
FBXW7	MIRLET7BHG	0.536394354	1.75E-41	postive
TFAP2C	RN7SL832P	0.566069771	5.45E-47	postive
HBA1	RN7SL832P	0.533674622	5.25E-41	postive
PTGS2	AC008011.2	0.458849491	2.02E-29	postive
IL6	AC008011.2	0.491033476	4.68E-34	postive
SLC2A3	AC008011.2	0.415087313	7.31E-24	postive
VEGFA	AC009163.7	0.424198258	5.92E-25	postive
TUBE1	AC009163.7	0.439773281	6.73E-27	postive
ALOX12	AC009163.7	0.619451681	1.99E-58	postive
SP1	AC009163.7	0.404379271	1.27E-22	postive
TUBE1	AC084876.1	0.54044266	3.34E-42	postive
ALOX12	AC084876.1	0.476119539	7.57E-32	postive
PHKG2	AC084876.1	0.510014502	4.98E-37	postive
TAZ	AC084876.1	0.629575722	7.49E-61	postive
FBXW7	AC084876.1	0.402974757	1.84E-22	postive
ALOX12	AL136368.1	0.443605462	2.16E-27	postive
RB1	CARD8-AS1	0.421293358	1.33E-24	postive
SLC1A4	CARD8-AS1	0.471134882	3.92E-31	postive
IL33	CARD8-AS1	0.453165223	1.18E-28	postive
HMGB1	CARD8-AS1	0.518794178	1.81E-38	postive
GABPB1	CARD8-AS1	0.436375205	1.82E-26	postive
ZEB1	CARD8-AS1	0.59138295	3.79E-52	postive
EPAS1	CARD8-AS1	0.416482711	5.00E-24	postive
TLR4	CARD8-AS1	0.52391054	2.51E-39	postive
TFAP2C	U91319.1	0.409000708	3.76E-23	postive
HRAS	LINC01137	0.500647982	1.54E-35	postive
FANCD2	AC104653.1	0.415301479	6.90E-24	postive
TFAP2C	AC104653.1	0.468716607	8.63E-31	postive
ZNF419	LENG8-AS1	0.529110732	3.25E-40	postive
VEGFA	LENG8-AS1	0.535761725	2.26E-41	postive
TUBE1	LENG8-AS1	0.555268412	6.38E-45	postive
SETD1B	LENG8-AS1	0.415758929	6.09E-24	postive
DRD4	LENG8-AS1	0.444255401	1.78E-27	postive
ALOX12	LENG8-AS1	0.700798931	8.26E-81	postive
PHKG2	LENG8-AS1	0.495222088	1.07E-34	postive
YY1AP1	LENG8-AS1	0.419095681	2.44E-24	postive
TAZ	LENG8-AS1	0.679800144	2.46E-74	postive
GPX4	AC090425.2	0.418936311	2.55E-24	postive

HSPB1	AC090425.2	0.452545216	1.43E-28	postive
HBA1	AC090425.2	0.51786713	2.58E-38	postive
HRAS	AC090425.2	0.68025023	1.81E-74	postive
BECN1	AC090425.2	-0.440402157	5.59E-27	negative
MAP1LC3A	AC090425.2	0.430733222	9.31E-26	postive
EGLN2	AC090425.2	0.774883773	4.68E-109	postive
MUC1	KRT7-AS	0.501703726	1.05E-35	postive
CD44	KRT7-AS	0.430005766	1.15E-25	postive
SOCS1	KRT7-AS	0.467174935	1.42E-30	postive
STMN1	LINC00973	0.736321938	3.70E-93	postive
RRM2	LINC00973	0.529168407	3.18E-40	postive
AURKA	LINC00973	0.547908052	1.48E-43	postive
CDKN2A	LINC00973	0.805354675	4.78E-124	postive
HSPB1	AC009065.4	0.514912732	7.93E-38	postive
SLC2A6	AC009065.4	0.424054068	6.17E-25	postive
NRAS	AC009065.4	-0.40550904	9.46E-23	negative
HRAS	AC009065.4	0.60272622	1.30E-54	postive
NCOA4	AC009065.4	-0.470574492	4.71E-31	negative
PHKG2	AC009065.4	0.710671435	4.71E-84	postive
BECN1	AC009065.4	-0.40565991	9.10E-23	negative
MAPK1	AC009065.4	-0.462668432	6.03E-30	negative
EGLN2	AC009065.4	0.671210007	7.68E-72	postive
TAZ	AC009065.4	0.698066976	6.18E-80	postive
HELLS	COL18A1-AS	0.534388308	3.93E-41	postive
TUBE1	COL18A1-AS	0.405782451	8.81E-23	postive
GABPB1	COL18A1-AS	0.537758126	1.00E-41	postive
ATG7	COL18A1-AS	0.460433185	1.22E-29	postive
LINC00472	COL18A1-AS	0.680880575	1.18E-74	postive
ATM	COL18A1-AS	0.725906279	2.46E-89	postive
FBXW7	COL18A1-AS	0.463407041	4.77E-30	postive
ATG4D	ZNF582-AS1	0.410308444	2.65E-23	postive
GABARAPL2	ZNF582-AS1	0.461531679	8.65E-30	postive
BAP1	ZNF582-AS1	0.441652788	3.86E-27	postive
HELLS	AC012181.1	0.441230048	4.37E-27	postive
ZNF419	AC012181.1	0.442782211	2.76E-27	postive
KLHL24	AC012181.1	0.439932272	6.42E-27	postive
TUBE1	AC012181.1	0.579978695	9.08E-50	postive
ALOX12	AC012181.1	0.498330672	3.54E-35	postive
IREB2	AC012181.1	0.423451975	7.30E-25	postive
GABPB1	AC012181.1	0.545321807	4.40E-43	postive
PIK3CA	AC012181.1	0.414638738	8.26E-24	postive
MAPK8	AC012181.1	0.452112335	1.64E-28	postive
LINC00472	AC012181.1	0.637434345	8.50E-63	postive
ATM	AC012181.1	0.691939836	5.20E-78	postive
FBXW7	AC012181.1	0.515095243	7.41E-38	postive
MUC1	ADCY6-DT	0.487266907	1.73E-33	postive
NFS1	AL118506.1	0.419588399	2.13E-24	postive
SETD1B	AL118506.1	0.436457675	1.78E-26	postive
TXNRD1	LINC01234	0.421566392	1.23E-24	postive
RRM2	LINC01234	0.457893351	2.72E-29	postive
AURKA	LINC01234	0.438628795	9.42E-27	postive
ALOX12	AL031432.3	0.405940033	8.45E-23	postive
TFAP2C	AL031432.3	0.483732506	5.83E-33	postive
HBA1	AL031432.3	0.432082499	6.32E-26	postive
DUOX1	AL031432.3	0.438592889	9.52E-27	postive
HELLS	AC002553.2	0.561199137	4.77E-46	postive
ZNF419	AC002553.2	0.469119384	7.57E-31	postive
TUBE1	AC002553.2	0.592144463	2.61E-52	postive

ALOX12	AC002553.2	0.548122844	1.35E-43	postive
GABPB1	AC002553.2	0.590047944	7.28E-52	postive
LINC00472	AC002553.2	0.708850279	1.91E-83	postive
ATM	AC002553.2	0.708767143	2.04E-83	postive
FBXW7	AC002553.2	0.519640741	1.31E-38	postive
KLHL24	AL512625.1	0.413374646	1.16E-23	postive
LINC00472	AL512625.1	0.453936469	9.33E-29	postive
LPIN1	AL512625.1	0.450561922	2.63E-28	postive
LAMP2	LINC02167	0.535403467	2.61E-41	postive
TFAP2C	AL359397.1	0.472321385	2.66E-31	postive
TUBE1	U91328.3	0.499100163	2.69E-35	postive
ALOX12	U91328.3	0.431762982	6.93E-26	postive
PHKG2	U91328.3	0.516241939	4.79E-38	postive
TAZ	U91328.3	0.620738077	9.90E-59	postive
ZNF419	AC092171.4	0.47248481	2.52E-31	postive
TUBE1	AC092171.4	0.440599105	5.27E-27	postive
ALOX12	AC092171.4	0.459006031	1.92E-29	postive
PHKG2	AC092171.4	0.609197764	4.63E-56	postive
TAZ	AC092171.4	0.701082419	6.69E-81	postive
BRD4	AC022167.2	0.477274874	5.15E-32	postive
ZNF419	AC022167.2	0.483999284	5.32E-33	postive
VEGFA	AC022167.2	0.446249546	9.75E-28	postive
TUBE1	AC022167.2	0.52274115	3.95E-39	postive
SETD1B	AC022167.2	0.498970627	2.82E-35	postive
DRD4	AC022167.2	0.413746509	1.05E-23	postive
ALOX12	AC022167.2	0.633825339	6.75E-62	postive
PHKG2	AC022167.2	0.54181359	1.89E-42	postive
TAZ	AC022167.2	0.664208575	7.20E-70	postive
ZNF419	AC093110.1	0.505359383	2.78E-36	postive
VEGFA	AC093110.1	0.473443295	1.84E-31	postive
TUBE1	AC093110.1	0.553767744	1.22E-44	postive
SETD1B	AC093110.1	0.494933828	1.19E-34	postive
ALOX12	AC093110.1	0.572334935	3.17E-48	postive
GABPB1	AC093110.1	0.492524311	2.78E-34	postive
ZEB1	AC093110.1	0.424322422	5.72E-25	postive
MAPK8	AC093110.1	0.471211515	3.83E-31	postive
LINC00472	AC093110.1	0.574187349	1.35E-48	postive
ATM	AC093110.1	0.477393059	4.95E-32	postive
FBXW7	AC093110.1	0.428064584	1.99E-25	postive
MTOR	AC108693.2	0.464328975	3.55E-30	postive
ACSL3	AC108693.2	0.464518247	3.34E-30	postive
KLHL24	AC108693.2	0.741433111	4.22E-95	postive
MAP3K5	AC108693.2	0.42751115	2.33E-25	postive
EIF2AK4	AC108693.2	0.403602257	1.56E-22	postive
IREB2	AC108693.2	0.542642141	1.34E-42	postive
PIK3CA	AC108693.2	0.607634241	1.04E-55	postive
KRAS	AC108693.2	0.475677967	8.77E-32	postive
SLC38A1	AC108693.2	0.447302339	7.10E-28	postive
ATG7	AC108693.2	0.431220302	8.10E-26	postive
GABARAPL1	AC108693.2	0.458730357	2.09E-29	postive
LINC00472	AC108693.2	0.787008091	1.00E-114	postive
PRKAA1	AC108693.2	0.462908902	5.59E-30	postive
TGFBR1	AC108693.2	0.460487282	1.20E-29	postive
LPIN1	AC108693.2	0.550462232	5.02E-44	postive
ATM	AC108693.2	0.417839945	3.45E-24	postive
TUBE1	LINC02019	0.553455053	1.39E-44	postive
ALOX12	LINC02019	0.449798394	3.33E-28	postive
PHKG2	LINC02019	0.548913711	9.69E-44	postive

TAZ	LINC02019	0.613835258	4.04E-57	postive
SLC3A2	LINC02432	0.409496155	3.29E-23	postive
ISCU	LINC02432	0.521413676	6.61E-39	postive
DDIT3	LINC02432	0.445995845	1.05E-27	postive
KLHL24	LINC02432	0.457976563	2.65E-29	postive
GABARAPL1	LINC02432	0.596004103	3.86E-53	postive
WIPI2	LINC02432	0.408317715	4.51E-23	postive
LPIN1	LINC02432	0.57431086	1.28E-48	postive
ZNF419	AC104532.2	0.554957318	7.29E-45	postive
TUBE1	AC104532.2	0.570442473	7.53E-48	postive
SETD1B	AC104532.2	0.51892148	1.72E-38	postive
ALOX12	AC104532.2	0.658881255	2.10E-68	postive
GABPB1	AC104532.2	0.435535473	2.33E-26	postive
ATM	AC104532.2	0.529760356	2.51E-40	postive
FBXW7	AC104532.2	0.433694459	3.97E-26	postive
PHKG2	AC096677.1	0.424244527	5.85E-25	postive
TUBE1	AC022306.2	0.512158297	2.24E-37	postive
NOX1	AC022306.2	0.484583692	4.36E-33	postive
BECN1	AC022306.2	-0.413152127	1.23E-23	negative
HELLS	TAPT1-AS1	0.475879206	8.20E-32	postive
MTOR	TAPT1-AS1	0.40057185	3.43E-22	postive
ZNF419	TAPT1-AS1	0.463786564	4.22E-30	postive
KLHL24	TAPT1-AS1	0.421140622	1.39E-24	postive
TUBE1	TAPT1-AS1	0.453947052	9.30E-29	postive
ALOX12	TAPT1-AS1	0.44592356	1.08E-27	postive
IREB2	TAPT1-AS1	0.415559528	6.43E-24	postive
GABPB1	TAPT1-AS1	0.532418928	8.69E-41	postive
MAPK8	TAPT1-AS1	0.426441874	3.15E-25	postive
LINC00472	TAPT1-AS1	0.646425137	4.30E-65	postive
LPIN1	TAPT1-AS1	0.476495055	6.68E-32	postive
ATM	TAPT1-AS1	0.60957487	3.80E-56	postive
FBXW7	TAPT1-AS1	0.526268619	9.97E-40	postive
HELLS	AL158163.1	0.524408033	2.07E-39	postive
ZNF419	AL158163.1	0.515191152	7.14E-38	postive
KLHL24	AL158163.1	0.4222106	1.03E-24	postive
TUBE1	AL158163.1	0.56755879	2.79E-47	postive
ALOX12	AL158163.1	0.58186205	3.73E-50	postive
GABPB1	AL158163.1	0.611635159	1.29E-56	postive
MAPK8	AL158163.1	0.456786748	3.85E-29	postive
LINC00472	AL158163.1	0.762018066	2.04E-103	postive
ATM	AL158163.1	0.654246907	3.74E-67	postive
FBXW7	AL158163.1	0.520741997	8.57E-39	postive
CHMP6	AC007114.1	0.423753068	6.71E-25	postive
WIPI1	AC007114.1	0.45457142	7.67E-29	postive
GPX4	SNHG6	0.401285901	2.85E-22	postive
HSPB1	SNHG6	0.41791558	3.38E-24	postive
HSF1	SNHG6	0.617981234	4.40E-58	postive
STAT3	SNHG6	-0.415359496	6.79E-24	negative
MTOR	SNHG6	-0.478581681	3.33E-32	negative
RPL8	SNHG6	0.707521036	5.28E-83	postive
IREB2	SNHG6	-0.433229184	4.54E-26	negative
RIPK1	SNHG6	-0.415446869	6.63E-24	negative
HRAS	SNHG6	0.412240313	1.58E-23	postive
BECN1	SNHG6	-0.427683718	2.22E-25	negative
MAPK1	SNHG6	-0.406979623	6.42E-23	negative
BID	SNHG6	0.470318531	5.12E-31	postive
ALOX12	LINC00671	0.408251744	4.58E-23	postive
MIOX	LINC00671	0.511046459	3.39E-37	postive

MTOR	LINC00863	0.472758515	2.30E-31	postive
SETD1B	LINC00863	0.408894362	3.87E-23	postive
MAPK14	LINC00863	0.521140329	7.35E-39	postive
IREB2	LINC00863	0.474661896	1.23E-31	postive
BECN1	LINC00863	0.411193516	2.09E-23	postive
ULK2	LINC00863	0.476121091	7.57E-32	postive
MAPK8	LINC00863	0.42082716	1.51E-24	postive
ATM	LINC00863	0.415167835	7.15E-24	postive
HRAS	AC114341.1	0.45186992	1.76E-28	postive
EGLN2	AC114341.1	0.560294356	7.10E-46	postive
SLC3A2	PRDM16-DT	0.405772491	8.83E-23	postive
FH	PRDM16-DT	0.407926032	5.00E-23	postive
ISCU	PRDM16-DT	0.584610287	1.01E-50	postive
DDIT3	PRDM16-DT	0.558261295	1.73E-45	postive
SLC2A8	PRDM16-DT	0.445051103	1.40E-27	postive
SLC2A12	PRDM16-DT	0.453653881	1.02E-28	postive
GOT1	PRDM16-DT	0.404301257	1.30E-22	postive
ATG4D	PRDM16-DT	0.566888811	3.77E-47	postive
MAP1LC3A	PRDM16-DT	0.667579318	8.22E-71	postive
GABARAPL1	PRDM16-DT	0.723327539	2.04E-88	postive
WIPI2	PRDM16-DT	0.533462722	5.71E-41	postive
BAP1	PRDM16-DT	0.446058485	1.03E-27	postive
LPIN1	PRDM16-DT	0.567713537	2.60E-47	postive
EGLN2	PRDM16-DT	0.422193261	1.04E-24	postive
HRAS	WDR5-DT	0.409104753	3.65E-23	postive
EGLN2	WDR5-DT	0.406571564	7.15E-23	postive
TAZ	WDR5-DT	0.47934876	2.57E-32	postive
NOX1	AC124016.1	0.587193402	2.91E-51	postive
BECN1	AC124016.1	-0.496936088	5.83E-35	negative
ZNF419	AC005306.1	0.518677302	1.89E-38	postive
VEGFA	AC005306.1	0.489584068	7.76E-34	postive
TUBE1	AC005306.1	0.494748942	1.27E-34	postive
SETD1B	AC005306.1	0.428494804	1.76E-25	postive
DRD4	AC005306.1	0.447891089	5.94E-28	postive
ALOX12	AC005306.1	0.533108662	6.59E-41	postive
PHKG2	AC005306.1	0.559494943	1.01E-45	postive
TAZ	AC005306.1	0.768852592	2.29E-106	postive
HELLS	SCARNA9	0.495791209	8.77E-35	postive
GABPB1	SCARNA9	0.475947586	8.02E-32	postive
ATG7	SCARNA9	0.439012678	8.42E-27	postive
LINC00472	SCARNA9	0.559907665	8.42E-46	postive
ATM	SCARNA9	0.681005965	1.08E-74	postive
FBXW7	SCARNA9	0.490204575	6.25E-34	postive
HELLS	ANKRD10-IT	0.504366028	4.00E-36	postive
ZNF419	ANKRD10-IT	0.55579527	5.07E-45	postive
KLHL24	ANKRD10-IT	0.408457943	4.34E-23	postive
TUBE1	ANKRD10-IT	0.629672587	7.09E-61	postive
SETD1B	ANKRD10-IT	0.431687856	7.08E-26	postive
ALOX12	ANKRD10-IT	0.587584572	2.41E-51	postive
GABPB1	ANKRD10-IT	0.652580586	1.04E-66	postive
MAPK8	ANKRD10-IT	0.472895148	2.20E-31	postive
LINC00472	ANKRD10-IT	0.718935908	7.09E-87	postive
ATM	ANKRD10-IT	0.695538099	3.90E-79	postive
FBXW7	ANKRD10-IT	0.587450279	2.57E-51	postive
PHKG2	AC010719.1	0.498437884	3.41E-35	postive
TAZ	AC010719.1	0.543751453	8.47E-43	postive
SRC	AC002401.4	0.421926918	1.12E-24	postive
ZNF419	ZNF213-AS1	0.536431042	1.72E-41	postive

VEGFA	ZNF213-AS1	0.422986462	8.31E-25	postive
TUBE1	ZNF213-AS1	0.551731565	2.92E-44	postive
DRD4	ZNF213-AS1	0.473672823	1.70E-31	postive
ALOX12	ZNF213-AS1	0.639815627	2.13E-63	postive
PHKG2	ZNF213-AS1	0.636848941	1.19E-62	postive
TAZ	ZNF213-AS1	0.692863278	2.68E-78	postive
GPX4	AP001189.3	-0.406934789	6.50E-23	negative
STAT3	AP001189.3	0.433059162	4.77E-26	postive
SLC1A4	AP001189.3	0.432461722	5.67E-26	postive
IL33	AP001189.3	0.457019918	3.58E-29	postive
HIC1	AP001189.3	0.435950777	2.06E-26	postive
RIPK1	AP001189.3	0.403611371	1.56E-22	postive
ZEB1	AP001189.3	0.657517009	4.93E-68	postive
EPAS1	AP001189.3	0.715942021	7.67E-86	postive
HELLS	AC109454.2	0.515832777	5.60E-38	postive
ALOX12	AC109454.2	0.429926474	1.17E-25	postive
GABPB1	AC109454.2	0.401737289	2.54E-22	postive
DUOX1	AC109454.2	0.407637583	5.40E-23	postive
ATM	AC109454.2	0.48560921	3.07E-33	postive
HELLS	AC253576.2	0.505039462	3.13E-36	postive
ZNF419	AC253576.2	0.461785115	7.98E-30	postive
TUBE1	AC253576.2	0.560522351	6.43E-46	postive
ALOX12	AC253576.2	0.425636964	3.95E-25	postive
GABPB1	AC253576.2	0.588064711	1.91E-51	postive
LINC00472	AC253576.2	0.704116328	6.95E-82	postive
ATM	AC253576.2	0.583705743	1.55E-50	postive
FBXW7	AC253576.2	0.487221023	1.76E-33	postive
HELLS	AC113143.1	0.422027472	1.09E-24	postive
TFAP2C	AC113143.1	0.51278714	1.77E-37	postive
HBA1	AC113143.1	0.534544787	3.69E-41	postive
DUOX1	AC113143.1	0.429856919	1.20E-25	postive
SLC3A2	AL035661.1	0.429833133	1.20E-25	postive
FH	AL035661.1	0.443717001	2.09E-27	postive
ISCU	AL035661.1	0.577982516	2.32E-49	postive
KLHL24	AL035661.1	0.403144033	1.76E-22	postive
SLC2A8	AL035661.1	0.467208997	1.41E-30	postive
GOT1	AL035661.1	0.422816481	8.71E-25	postive
ATG4D	AL035661.1	0.561991961	3.36E-46	postive
MAP1LC3A	AL035661.1	0.421190121	1.37E-24	postive
GABARAPL2	AL035661.1	0.518119391	2.34E-38	postive
GABARAPL1	AL035661.1	0.659793494	1.18E-68	postive
LPIN1	AL035661.1	0.526881982	7.84E-40	postive
PLIN2	AC002401.2	0.45120206	2.17E-28	postive
HNF4A	AC002401.2	0.440004363	6.28E-27	postive
LPCAT3	AC002401.2	0.44524613	1.32E-27	postive
MAPK1	AC002401.2	0.445507807	1.22E-27	postive
VEGFA	BX255925.1	0.521771935	5.75E-39	postive
HIC1	BX255925.1	0.544147982	7.18E-43	postive
MAPK3	BX255925.1	0.407366953	5.80E-23	postive
ZEB1	BX255925.1	0.510777845	3.75E-37	postive
EPAS1	BX255925.1	0.5149808	7.73E-38	postive
SP1	AC103740.1	0.455046368	6.62E-29	postive
ATM	AC103740.1	0.468687821	8.71E-31	postive
HELLS	AC008115.3	0.491979904	3.36E-34	postive
ZNF419	AC008115.3	0.467582059	1.25E-30	postive
VEGFA	AC008115.3	0.406955367	6.46E-23	postive
TUBE1	AC008115.3	0.567537831	2.81E-47	postive
SETD1B	AC008115.3	0.407493707	5.61E-23	postive

ALOX12	AC008115.3	0.60242798	1.52E-54	postive
GABPB1	AC008115.3	0.532458946	8.55E-41	postive
LINC00472	AC008115.3	0.407958346	4.96E-23	postive
ATM	AC008115.3	0.683132647	2.51E-75	postive
FBXW7	AC008115.3	0.593577213	1.29E-52	postive
VEGFA	CEBPB-AS1	0.45154541	1.95E-28	postive
ALOX12	CEBPB-AS1	0.432517074	5.58E-26	postive
SLC2A6	EXOSC10-AS	0.415210924	7.07E-24	postive
SLC2A8	EXOSC10-AS	0.416776105	4.62E-24	postive
VDAC2	NCKAP5-AS2	0.414340133	8.96E-24	postive
ISCU	NCKAP5-AS2	0.676853557	1.80E-73	postive
TMBIM4	NCKAP5-AS2	0.543952091	7.79E-43	postive
CHMP5	NCKAP5-AS2	0.448757578	4.57E-28	postive
DDIT3	NCKAP5-AS2	0.443292783	2.37E-27	postive
GOT1	NCKAP5-AS2	0.500788405	1.47E-35	postive
ATG4D	NCKAP5-AS2	0.483373247	6.59E-33	postive
MAP1LC3A	NCKAP5-AS2	0.448385189	5.11E-28	postive
GABARAPL2	NCKAP5-AS2	0.553487593	1.37E-44	postive
GABARAPL1	NCKAP5-AS2	0.444224725	1.79E-27	postive
TUBE1	AP001767.2	0.488097434	1.30E-33	postive
ALOX12	AP001767.2	0.543975845	7.72E-43	postive
PHKG2	AP001767.2	0.471395373	3.60E-31	postive
TAZ	AP001767.2	0.548804517	1.02E-43	postive
HELLS	ODF2-AS1	0.428017019	2.02E-25	postive
ZNF419	ODF2-AS1	0.442882112	2.68E-27	postive
TUBE1	ODF2-AS1	0.510171646	4.70E-37	postive
ALOX12	ODF2-AS1	0.460476089	1.21E-29	postive
GABPB1	ODF2-AS1	0.513367782	1.42E-37	postive
LINC00472	ODF2-AS1	0.518740534	1.85E-38	postive
ATM	ODF2-AS1	0.510568712	4.05E-37	postive
FBXW7	ODF2-AS1	0.472939818	2.17E-31	postive
TUBE1	ZEB1-AS1	0.438856358	8.81E-27	postive
ALOX12	ZEB1-AS1	0.428535019	1.74E-25	postive
GABPB1	ZEB1-AS1	0.434314049	3.32E-26	postive
MAPK8	ZEB1-AS1	0.406820126	6.70E-23	postive
SLC3A2	LINC02568	0.403928026	1.43E-22	postive
ISCU	LINC02568	0.482638091	8.46E-33	postive
DDIT3	LINC02568	0.43541178	2.41E-26	postive
SLC2A12	LINC02568	0.450151196	2.99E-28	postive
TFAP2C	LINC02568	0.402020284	2.36E-22	postive
ATG4D	LINC02568	0.495466644	9.84E-35	postive
MAP1LC3A	LINC02568	0.427689688	2.21E-25	postive
GABARAPL1	LINC02568	0.617535468	5.59E-58	postive
WIPI2	LINC02568	0.406667968	6.97E-23	postive
LPIN1	LINC02568	0.506977935	1.53E-36	postive
HELLS	AC010680.2	0.553844057	1.18E-44	postive
TUBE1	AC010680.2	0.531982597	1.03E-40	postive
ALOX12	AC010680.2	0.479107128	2.79E-32	postive
GABPB1	AC010680.2	0.574932414	9.58E-49	postive
MAPK8	AC010680.2	0.423900957	6.44E-25	postive
LINC00472	AC010680.2	0.621952188	5.11E-59	postive
ATM	AC010680.2	0.725485017	3.48E-89	postive
FBXW7	AC010680.2	0.469675816	6.32E-31	postive
MAPK14	ANAPC1P2	0.402646587	2.00E-22	postive
IREB2	ANAPC1P2	0.413361135	1.17E-23	postive
SP1	ANAPC1P2	0.429225271	1.43E-25	postive
ZEB1	ANAPC1P2	0.40405854	1.38E-22	postive
MAPK8	ANAPC1P2	0.410445543	2.56E-23	postive

GPX4	AL035420.3	0.472628975	2.40E-31	postive
OTUB1	AL035420.3	0.40404854	1.39E-22	postive
ZNF419	STAG3L5P-P'	0.435018898	2.71E-26	postive
VEGFA	STAG3L5P-P'	0.418656024	2.76E-24	postive
TUBE1	STAG3L5P-P'	0.571160938	5.43E-48	postive
DRD4	STAG3L5P-P'	0.424661265	5.20E-25	postive
ALOX12	STAG3L5P-P'	0.552164381	2.43E-44	postive
PHKG2	STAG3L5P-P'	0.590158794	6.89E-52	postive
TAZ	STAG3L5P-P'	0.728114526	3.93E-90	postive
MAFG	AC010271.1	0.420500958	1.66E-24	postive
ALOX12	AC010271.1	0.404874675	1.12E-22	postive
HELLS	AL133445.2	0.512215983	2.19E-37	postive
TUBE1	AL133445.2	0.419208692	2.37E-24	postive
GABPB1	AL133445.2	0.538449574	7.56E-42	postive
ATG7	AL133445.2	0.457565681	3.02E-29	postive
LINC00472	AL133445.2	0.717482598	2.26E-86	postive
ATM	AL133445.2	0.697411492	9.98E-80	postive
FBXW7	AL133445.2	0.426864752	2.80E-25	postive
HELLS	AC016949.1	0.478460849	3.47E-32	postive
KLHL24	AC016949.1	0.410608501	2.45E-23	postive
TUBE1	AC016949.1	0.427724363	2.19E-25	postive
ALOX12	AC016949.1	0.423100249	8.05E-25	postive
GABPB1	AC016949.1	0.538534519	7.30E-42	postive
LINC00472	AC016949.1	0.812476829	6.14E-128	postive
ATM	AC016949.1	0.568389062	1.91E-47	postive
FBXW7	AC016949.1	0.47273759	2.32E-31	postive
ISCU	AL161729.1	0.535832075	2.19E-41	postive
KLHL24	AL161729.1	0.402486108	2.09E-22	postive
ATG4D	AL161729.1	0.448409994	5.07E-28	postive
MAP1LC3A	AL161729.1	0.428983384	1.53E-25	postive
GABARAPL2	AL161729.1	0.405743211	8.90E-23	postive
GABARAPL1	AL161729.1	0.535369257	2.65E-41	postive
BAP1	AL161729.1	0.492662019	2.65E-34	postive
LPIN1	AL161729.1	0.462126976	7.16E-30	postive
ZNF419	LINC01160	0.427157083	2.58E-25	postive
TUBE1	LINC01160	0.40962742	3.18E-23	postive
ALOX12	LINC01160	0.461187692	9.65E-30	postive
PHKG2	LINC01160	0.60736193	1.20E-55	postive
TAZ	LINC01160	0.675615564	4.14E-73	postive
RB1	AC096921.2	0.403681322	1.53E-22	postive
IL33	AC096921.2	0.410766522	2.35E-23	postive
IREB2	AC096921.2	0.403621211	1.55E-22	postive
HMGB1	AC096921.2	0.538743101	6.70E-42	postive
CAPG	AC096921.2	-0.401798848	2.50E-22	negative
GABPB1	AC096921.2	0.525173361	1.53E-39	postive
PIK3CA	AC096921.2	0.434187557	3.44E-26	postive
KRAS	AC096921.2	0.473792531	1.64E-31	postive
ZEB1	AC096921.2	0.680519559	1.51E-74	postive
MAPK8	AC096921.2	0.575315428	8.02E-49	postive
PRKAA1	AC096921.2	0.499409054	2.41E-35	postive
EPAS1	AC096921.2	0.615942507	1.31E-57	postive
ATM	AC096921.2	0.404228725	1.32E-22	postive
SIRT1	AC096921.2	0.488292219	1.22E-33	postive
TUBE1	AP000553.2	0.484773999	4.08E-33	postive
ALOX12	AP000553.2	0.476075289	7.69E-32	postive
PHKG2	AP000553.2	0.500365858	1.71E-35	postive
TAZ	AP000553.2	0.557098082	2.88E-45	postive
GDF15	AC026369.1	0.415479728	6.57E-24	postive

AGPAT3	AC026369.1	0.468343437	9.74E-31	postive
MIOX	AC026369.1	0.444676267	1.57E-27	postive
HELLS	AL133243.2	0.526962254	7.59E-40	postive
KLHL24	AL133243.2	0.50006564	1.90E-35	postive
TUBE1	AL133243.2	0.438948776	8.58E-27	postive
IREB2	AL133243.2	0.451607846	1.91E-28	postive
GABPB1	AL133243.2	0.592244256	2.48E-52	postive
PIK3CA	AL133243.2	0.475282496	1.00E-31	postive
KRAS	AL133243.2	0.432159499	6.18E-26	postive
MAPK8	AL133243.2	0.446849056	8.14E-28	postive
LINC00472	AL133243.2	0.80166323	4.30E-122	postive
PRKAA1	AL133243.2	0.448439314	5.03E-28	postive
ATM	AL133243.2	0.68391347	1.46E-75	postive
FBXW7	AL133243.2	0.45872359	2.10E-29	postive
BACH1	AL133243.2	0.426955479	2.73E-25	postive
HELLS	AC008667.1	0.404005387	1.40E-22	postive
TFAP2C	AC008667.1	0.458291295	2.40E-29	postive
HBA1	AC008667.1	0.434228812	3.40E-26	postive
DUOX1	AC008667.1	0.415971745	5.75E-24	postive
GPX4	LINC01023	0.412431192	1.50E-23	postive
HSPB1	LINC01023	0.483965723	5.38E-33	postive
RPL8	LINC01023	0.441931646	3.55E-27	postive
HBA1	LINC01023	0.624931963	9.94E-60	postive
HRAS	LINC01023	0.767738477	7.04E-106	postive
MAP1LC3A	LINC01023	0.517430321	3.05E-38	postive
EGLN2	LINC01023	0.856845611	1.27E-156	postive
KLHL24	AC058791.1	0.576017025	5.79E-49	postive
GABPB1	AC058791.1	0.424103811	6.08E-25	postive
PIK3CA	AC058791.1	0.467875464	1.13E-30	postive
KRAS	AC058791.1	0.410436261	2.56E-23	postive
LINC00472	AC058791.1	0.950934329	9.17E-276	postive
HELLS	GMDS-DT	0.539990776	4.02E-42	postive
TUBE1	GMDS-DT	0.527484481	6.18E-40	postive
ALOX12	GMDS-DT	0.480780101	1.59E-32	postive
IREB2	GMDS-DT	0.449137114	4.07E-28	postive
GABPB1	GMDS-DT	0.564881309	9.28E-47	postive
PIK3CA	GMDS-DT	0.41282353	1.35E-23	postive
ATG7	GMDS-DT	0.498480169	3.36E-35	postive
MAPK8	GMDS-DT	0.466992635	1.51E-30	postive
LINC00472	GMDS-DT	0.640882794	1.14E-63	postive
PRKAA2	GMDS-DT	0.421582717	1.23E-24	postive
ATM	GMDS-DT	0.810264666	1.03E-126	postive
FBXW7	GMDS-DT	0.548256272	1.28E-43	postive
HELLS	ALMS1-IT1	0.569484449	1.17E-47	postive
LINC00472	ALMS1-IT1	0.558302353	1.70E-45	postive
ATM	ALMS1-IT1	0.484317185	4.77E-33	postive
HELLS	AC011468.1	0.465549026	2.40E-30	postive
ZNF419	AC011468.1	0.58879059	1.34E-51	postive
TUBE1	AC011468.1	0.481910285	1.08E-32	postive
ALOX12	AC011468.1	0.406601514	7.10E-23	postive
GABPB1	AC011468.1	0.507846595	1.11E-36	postive
PHKG2	AC011468.1	0.457025239	3.57E-29	postive
LINC00472	AC011468.1	0.440204567	5.92E-27	postive
TAZ	AC011468.1	0.509850313	5.30E-37	postive
FBXW7	AC011468.1	0.488269867	1.22E-33	postive
TFAP2C	SNED1-AS1	0.560899155	5.44E-46	postive
HBA1	SNED1-AS1	0.503641603	5.21E-36	postive
DUOX1	SNED1-AS1	0.434334903	3.30E-26	postive

ZNF419	HIF1A-AS3	0.402496671	2.08E-22	postive
VEGFA	HIF1A-AS3	0.505335013	2.80E-36	postive
TUBE1	HIF1A-AS3	0.491087931	4.60E-34	postive
ALOX12	HIF1A-AS3	0.562057088	3.26E-46	postive
IREB2	HIF1A-AS3	0.412517756	1.47E-23	postive
GABPB1	HIF1A-AS3	0.479942382	2.11E-32	postive
ZEB1	HIF1A-AS3	0.419839072	1.99E-24	postive
MAPK8	HIF1A-AS3	0.512920116	1.68E-37	postive
LINC00472	HIF1A-AS3	0.470372184	5.03E-31	postive
ATM	HIF1A-AS3	0.683023197	2.71E-75	postive
FBXW7	HIF1A-AS3	0.421965174	1.10E-24	postive
CD44	TBILA	0.430314397	1.05E-25	postive
SOCS1	TBILA	0.613373784	5.16E-57	postive
DDIT3	BX539320.1	0.428458438	1.78E-25	postive
ATG4D	BX539320.1	0.453568336	1.05E-28	postive
MAP1LC3A	BX539320.1	0.407200318	6.06E-23	postive
HBA1	AL359962.1	0.408905258	3.85E-23	postive
SLC7A11	LINC01270	0.407731946	5.26E-23	postive
TXNRD1	LINC01270	0.470698141	4.53E-31	postive
RRM2	LINC01270	0.411352897	2.00E-23	postive
AURKA	LINC01270	0.446402455	9.31E-28	postive
G6PD	LINC01270	0.410680908	2.40E-23	postive
TFAP2C	AC090114.2	0.419480384	2.20E-24	postive
HBA1	AC090114.2	0.426912452	2.76E-25	postive
FANCD2	AC099850.3	0.660447232	7.85E-69	postive
HELLS	AC099850.3	0.424561597	5.35E-25	postive
STMN1	AC099850.3	0.700500908	1.03E-80	postive
RRM2	AC099850.3	0.802868596	1.00E-122	postive
AURKA	AC099850.3	0.761694786	2.80E-103	postive
CDKN2A	AC099850.3	0.594883114	6.74E-53	postive
PANX1	AC099850.3	0.41457191	8.41E-24	postive
SLC3A2	ST3GAL6-AS	0.424045758	6.18E-25	postive
FH	ST3GAL6-AS	0.424754035	5.07E-25	postive
ISCU	ST3GAL6-AS	0.441445795	4.10E-27	postive
GOT1	ST3GAL6-AS	0.415883515	5.89E-24	postive
ATG4D	ST3GAL6-AS	0.404384249	1.27E-22	postive
GABARAPL1	ST3GAL6-AS	0.602409612	1.53E-54	postive
LPIN1	ST3GAL6-AS	0.43800963	1.13E-26	postive
VEGFA	AC104964.3	0.426310353	3.27E-25	postive
TUBE1	AC104964.3	0.561319256	4.52E-46	postive
ALOX12	AC104964.3	0.481454336	1.26E-32	postive
TAZ	AC104964.3	0.517363252	3.13E-38	postive
HELLS	AL136115.2	0.535360482	2.66E-41	postive
ALOX12	AL136115.2	0.417736297	3.55E-24	postive
GABPB1	AL136115.2	0.51831039	2.18E-38	postive
LINC00472	AL136115.2	0.759133536	3.35E-102	postive
ATM	AL136115.2	0.549754172	6.79E-44	postive
FBXW7	AL136115.2	0.438012731	1.13E-26	postive
TFAP2C	AP000255.1	0.486136981	2.56E-33	postive
HBA1	AP000255.1	0.565980473	5.67E-47	postive
ANO6	AC115618.2	0.41213987	1.62E-23	postive
HELLS	AC116552.1	0.42853928	1.74E-25	postive
ZNF419	AC116552.1	0.519072702	1.63E-38	postive
TUBE1	AC116552.1	0.517866876	2.58E-38	postive
ALOX12	AC116552.1	0.589026193	1.20E-51	postive
GABPB1	AC116552.1	0.480749355	1.60E-32	postive
LINC00472	AC116552.1	0.569029875	1.43E-47	postive
ATM	AC116552.1	0.551108893	3.81E-44	postive

FBXW7	AC116552.1	0.433742629	3.92E-26	postive
VDAC2	AC007405.3	0.402295047	2.19E-22	postive
TMBIM4	AC007405.3	0.455038213	6.63E-29	postive
HELLS	TRAF3IP2-AS	0.526125153	1.05E-39	postive
ZNF419	TRAF3IP2-AS	0.498957918	2.83E-35	postive
TUBE1	TRAF3IP2-AS	0.623129258	2.68E-59	postive
ALOX12	TRAF3IP2-AS	0.586972924	3.23E-51	postive
IREB2	TRAF3IP2-AS	0.460259213	1.29E-29	postive
GABPB1	TRAF3IP2-AS	0.670143373	1.55E-71	postive
ZEB1	TRAF3IP2-AS	0.45662524	4.05E-29	postive
MAPK8	TRAF3IP2-AS	0.559146629	1.18E-45	postive
LINC00472	TRAF3IP2-AS	0.60287192	1.21E-54	postive
ATM	TRAF3IP2-AS	0.730341142	6.09E-91	postive
FBXW7	TRAF3IP2-AS	0.541683234	2.00E-42	postive
JDP2	MEG3	0.627807546	2.01E-60	postive
RGS4	MEG3	0.46425004	3.64E-30	postive
EIF2S1	MEG3	0.407649363	5.38E-23	postive
FANCD2	AL078587.1	0.453114703	1.20E-28	postive
HELLS	AL078587.1	0.472953579	2.16E-31	postive
ZNF419	AL078587.1	0.410656167	2.42E-23	postive
ALOX12	AL078587.1	0.547208286	1.99E-43	postive
DUOX1	AL078587.1	0.405100741	1.05E-22	postive
HELLS	AL157394.1	0.545896181	3.46E-43	postive
GABPB1	AL157394.1	0.404980797	1.09E-22	postive
ATM	AL157394.1	0.525112844	1.57E-39	postive
FBXW7	AL157394.1	0.413685198	1.07E-23	postive
GPX4	AC008608.2	0.483858765	5.58E-33	postive
CHMP6	AC008608.2	0.420155692	1.82E-24	postive
RPL8	AC008608.2	0.409392885	3.38E-23	postive
HRAS	AC008608.2	0.63679299	1.23E-62	postive
BECN1	AC008608.2	-0.432904287	4.99E-26	negative
EGLN2	AC008608.2	0.462287697	6.81E-30	postive
TAZ	AC008608.2	0.40545804	9.59E-23	postive
TFAP2C	MAFTRR	0.490262992	6.13E-34	postive
HBA1	MAFTRR	0.489026262	9.42E-34	postive
PROM2	LINC01702	0.420095674	1.85E-24	postive
ISCU	AP000787.1	0.441643651	3.87E-27	postive
KLHL24	AP000787.1	0.543919413	7.90E-43	postive
GABPB1	AP000787.1	0.437138471	1.46E-26	postive
GABARAPL1	AP000787.1	0.445584147	1.19E-27	postive
LINC00472	AP000787.1	0.68868688	5.23E-77	postive
LPIN1	AP000787.1	0.636850639	1.19E-62	postive
HELLS	DIRC3	0.457998328	2.64E-29	postive
GABPB1	DIRC3	0.442197765	3.28E-27	postive
ATM	DIRC3	0.604753184	4.62E-55	postive
FBXW7	DIRC3	0.46009125	1.36E-29	postive
KLHL24	HOXB-AS2	0.61686648	8.01E-58	postive
IREB2	HOXB-AS2	0.437158347	1.45E-26	postive
PIK3CA	HOXB-AS2	0.505068792	3.09E-36	postive
KRAS	HOXB-AS2	0.432023606	6.43E-26	postive
LINC00472	HOXB-AS2	0.88792824	3.02E-183	postive
LPIN1	HOXB-AS2	0.466298401	1.89E-30	postive
KLHL24	AC010168.1	0.538489165	7.44E-42	postive
IREB2	AC010168.1	0.437682741	1.24E-26	postive
GABPB1	AC010168.1	0.460879355	1.06E-29	postive
PIK3CA	AC010168.1	0.435248978	2.53E-26	postive
LINC00472	AC010168.1	0.834833201	2.53E-141	postive
LPIN1	AC010168.1	0.417725511	3.56E-24	postive

ATM	AC010168.1	0.619112631	2.39E-58	postive
ZNF419	LINC00663	0.533920739	4.75E-41	postive
VEGFA	LINC00663	0.418957366	2.54E-24	postive
TUBE1	LINC00663	0.627394061	2.54E-60	postive
SETD1B	LINC00663	0.429529404	1.31E-25	postive
ALOX12	LINC00663	0.597239517	2.08E-53	postive
IREB2	LINC00663	0.410035184	2.85E-23	postive
SP1	LINC00663	0.411408188	1.97E-23	postive
GABPB1	LINC00663	0.417365047	3.93E-24	postive
ULK2	LINC00663	0.413214764	1.21E-23	postive
MAPK8	LINC00663	0.537042018	1.34E-41	postive
LINC00472	LINC00663	0.423306914	7.60E-25	postive
ATM	LINC00663	0.565841623	6.04E-47	postive
ZNF419	AL031846.2	0.546963606	2.21E-43	postive
VEGFA	AL031846.2	0.553757793	1.22E-44	postive
TUBE1	AL031846.2	0.62216729	4.54E-59	postive
SETD1B	AL031846.2	0.57725847	3.25E-49	postive
ALOX12	AL031846.2	0.777347467	3.53E-110	postive
GABPB1	AL031846.2	0.409195069	3.57E-23	postive
MAPK8	AL031846.2	0.411404005	1.98E-23	postive
ATM	AL031846.2	0.599386528	7.09E-54	postive
YY1AP1	AL031846.2	0.465388734	2.53E-30	postive
FBXW7	AL031846.2	0.436453412	1.78E-26	postive
HELLS	PDC-AS1	0.582666997	2.54E-50	postive
ZNF419	PDC-AS1	0.44290075	2.66E-27	postive
TUBE1	PDC-AS1	0.572143065	3.46E-48	postive
ALOX12	PDC-AS1	0.595298089	5.49E-53	postive
GABPB1	PDC-AS1	0.521507231	6.37E-39	postive
ATM	PDC-AS1	0.685895761	3.70E-76	postive
FBXW7	PDC-AS1	0.56736296	3.04E-47	postive
NRAS	COA6-AS1	-0.41586881	5.91E-24	negative
HRAS	COA6-AS1	0.428692851	1.67E-25	postive
NCOA4	COA6-AS1	-0.469593176	6.49E-31	negative
PHKG2	COA6-AS1	0.598581303	1.06E-53	postive
MAPK1	COA6-AS1	-0.424355205	5.67E-25	negative
ANO6	COA6-AS1	-0.416118239	5.52E-24	negative
TAZ	COA6-AS1	0.623704265	1.96E-59	postive
ATG4D	AC092171.3	0.485443804	3.24E-33	postive
ZNF419	CHASERR	0.43460242	3.05E-26	postive
TUBE1	CHASERR	0.507606024	1.22E-36	postive
GABPB1	CHASERR	0.537443116	1.14E-41	postive
LINC00472	CHASERR	0.452270177	1.56E-28	postive
PML	AL590764.1	0.406506578	7.28E-23	postive
GCH1	AL590764.1	0.456939345	3.67E-29	postive
NCF2	AL590764.1	0.462190288	7.02E-30	postive
SLC2A6	AL590764.1	0.43125528	8.02E-26	postive
CYBB	AL590764.1	0.456715591	3.94E-29	postive
IFNG	AL590764.1	0.622799409	3.21E-59	postive
HSF1	AP001453.2	0.457524845	3.06E-29	postive
G6PD	AP001453.2	0.436233236	1.90E-26	postive
CARS1	AP001453.2	0.411554023	1.90E-23	postive
NCOA4	AP001453.2	-0.411633862	1.86E-23	negative
PHKG2	AP001453.2	0.601226808	2.80E-54	postive
ULK1	AP001453.2	0.421820778	1.15E-24	postive
MAPK1	AP001453.2	-0.419777796	2.02E-24	negative
SOCS1	AP001453.2	0.44913909	4.06E-28	postive
PRKAA2	AP001453.2	-0.40096617	3.10E-22	negative
TAZ	AP001453.2	0.516657073	4.09E-38	postive

HELLS	AC022973.5	0.539768195	4.40E-42	postive
ZNF419	AC022973.5	0.466114937	2.00E-30	postive
TUBE1	AC022973.5	0.523477794	2.97E-39	postive
ALOX12	AC022973.5	0.531124711	1.46E-40	postive
GABPB1	AC022973.5	0.545670144	3.80E-43	postive
LINC00472	AC022973.5	0.576130584	5.49E-49	postive
ATM	AC022973.5	0.627331726	2.63E-60	postive
FBXW7	AC022973.5	0.495991782	8.16E-35	postive
FH	ZNF710-AS1	0.466531231	1.75E-30	postive
ISCU	ZNF710-AS1	0.525838904	1.18E-39	postive
GPT2	ZNF710-AS1	0.432655097	5.36E-26	postive
SLC2A12	ZNF710-AS1	0.506583639	1.77E-36	postive
CS	ZNF710-AS1	0.484367224	4.69E-33	postive
GOT1	ZNF710-AS1	0.444374801	1.71E-27	postive
ATG4D	ZNF710-AS1	0.568147878	2.14E-47	postive
GABARAPL1	ZNF710-AS1	0.57868923	1.66E-49	postive
WIPI2	ZNF710-AS1	0.506717828	1.69E-36	postive
LPIN1	ZNF710-AS1	0.443943851	1.95E-27	postive
KLHL24	AC009237.15	0.455078065	6.55E-29	postive
LINC00472	AC009237.15	0.648345966	1.36E-65	postive
ZNF419	AC009113.1	0.437681345	1.24E-26	postive
VEGFA	AC009113.1	0.439097354	8.21E-27	postive
TUBE1	AC009113.1	0.494729688	1.28E-34	postive
ALOX12	AC009113.1	0.53204447	1.01E-40	postive
TAZ	AC009113.1	0.409366933	3.41E-23	postive
HELLS	AC002563.1	0.405993108	8.33E-23	postive
TUBE1	AC002563.1	0.579787442	9.93E-50	postive
ALOX12	AC002563.1	0.555379837	6.08E-45	postive
ATM	AC002563.1	0.521079464	7.52E-39	postive
BRD4	TMEM147-A:	0.461004799	1.02E-29	postive
ZNF419	TMEM147-A:	0.577803632	2.52E-49	postive
TUBE1	TMEM147-A:	0.466803679	1.60E-30	postive
SETD1B	TMEM147-A:	0.434793234	2.89E-26	postive
ALOX12	TMEM147-A:	0.556612042	3.56E-45	postive
PHKG2	TMEM147-A:	0.606693958	1.70E-55	postive
ULK1	TMEM147-A:	0.420731812	1.56E-24	postive
TAZ	TMEM147-A:	0.715259525	1.31E-85	postive
HELLS	AF117829.1	0.549266766	8.34E-44	postive
ZNF419	AF117829.1	0.40865624	4.12E-23	postive
KLHL24	AF117829.1	0.487247944	1.74E-33	postive
TUBE1	AF117829.1	0.467248244	1.39E-30	postive
ALOX12	AF117829.1	0.402954809	1.85E-22	postive
GABPB1	AF117829.1	0.603830737	7.42E-55	postive
PIK3CA	AF117829.1	0.427571911	2.29E-25	postive
ATG7	AF117829.1	0.444566098	1.62E-27	postive
LINC00472	AF117829.1	0.824477721	6.90E-135	postive
ATM	AF117829.1	0.717783555	1.78E-86	postive
FBXW7	AF117829.1	0.541793157	1.91E-42	postive
ACO1	AC009275.1	0.42539659	4.23E-25	postive
IDH1	AC009275.1	0.513583189	1.31E-37	postive
HELLS	AC025569.1	0.556703864	3.42E-45	postive
ZNF419	AC025569.1	0.51392148	1.15E-37	postive
TUBE1	AC025569.1	0.531216184	1.41E-40	postive
ALOX12	AC025569.1	0.537580783	1.08E-41	postive
GABPB1	AC025569.1	0.6162817	1.10E-57	postive
ZEB1	AC025569.1	0.435477081	2.37E-26	postive
MAPK8	AC025569.1	0.458221246	2.46E-29	postive
LINC00472	AC025569.1	0.586443619	4.17E-51	postive

ATM	AC025569.1	0.704981734	3.62E-82	postive
FBXW7	AC025569.1	0.558187625	1.79E-45	postive
STEAP3	AC018695.6	0.439543325	7.20E-27	postive
PHKG2	AC018695.6	0.442979686	2.60E-27	postive
GOT1	AC116345.3	0.422405601	9.77E-25	postive
ATG4D	AC116345.3	0.467000426	1.50E-30	postive
MIOX	AC116345.3	0.409192516	3.57E-23	postive
GPX4	AL355353.1	0.43333274	4.41E-26	postive
HSPB1	AL355353.1	0.409525118	3.27E-23	postive
HBA1	AL355353.1	0.436725851	1.65E-26	postive
HRAS	AL355353.1	0.684883284	7.48E-76	postive
PHKG2	AL355353.1	0.44082845	4.93E-27	postive
MAP1LC3A	AL355353.1	0.400808412	3.23E-22	postive
PEBP1	AL355353.1	0.41151264	1.92E-23	postive
EGLN2	AL355353.1	0.626776368	3.58E-60	postive
TAZ	AL355353.1	0.465519644	2.42E-30	postive
FANCD2	AC116914.2	0.47743422	4.89E-32	postive
ZNF419	AC116914.2	0.436580311	1.72E-26	postive
ALOX12	AC116914.2	0.590289644	6.47E-52	postive
TAZ	AC116914.2	0.469555771	6.57E-31	postive
ALOX12	AC129507.3	0.487369495	1.67E-33	postive
ZNF419	AL513534.2	0.41850107	2.88E-24	postive
GABPB1	AL513534.2	0.4790504	2.84E-32	postive
ZEB1	AL513534.2	0.45412707	8.80E-29	postive
FBXW7	AL513534.2	0.431709963	7.03E-26	postive
HSPB1	DLG5-AS1	0.425871007	3.70E-25	postive
HRAS	DLG5-AS1	0.437166323	1.45E-26	postive
MAP1LC3A	DLG5-AS1	0.436287579	1.87E-26	postive
MAPK1	DLG5-AS1	-0.424433493	5.55E-25	negative
EGLN2	DLG5-AS1	0.487950903	1.37E-33	postive
NCF2	AC006033.2	0.518414988	2.09E-38	postive
CYBB	AC006033.2	0.576542696	4.54E-49	postive
IFNG	AC006033.2	0.417046877	4.29E-24	postive
HELLS	SP2-AS1	0.467413726	1.32E-30	postive
IREB2	SP2-AS1	0.421572216	1.23E-24	postive
PIK3CA	SP2-AS1	0.411266263	2.05E-23	postive
ATG7	SP2-AS1	0.448408641	5.08E-28	postive
MAPK8	SP2-AS1	0.400458353	3.53E-22	postive
LINC00472	SP2-AS1	0.472672671	2.37E-31	postive
ATM	SP2-AS1	0.633957443	6.26E-62	postive
BACH1	SP2-AS1	0.420097554	1.85E-24	postive
BRD4	AC245052.4	0.410146968	2.77E-23	postive
ZNF419	AC245052.4	0.564146866	1.29E-46	postive
VEGFA	AC245052.4	0.555069787	6.95E-45	postive
TUBE1	AC245052.4	0.568677282	1.68E-47	postive
SETD1B	AC245052.4	0.481458545	1.26E-32	postive
DRD4	AC245052.4	0.431035627	8.54E-26	postive
ALOX12	AC245052.4	0.68204256	5.31E-75	postive
PHKG2	AC245052.4	0.448387605	5.11E-28	postive
TAZ	AC245052.4	0.662024979	2.89E-69	postive
FBXW7	AC245052.4	0.431804766	6.85E-26	postive
TUBE1	AC020634.2	0.42682115	2.83E-25	postive
GABPB1	AC020634.2	0.515840834	5.58E-38	postive
ATM	AC020634.2	0.49622895	7.50E-35	postive
FBXW7	AC020634.2	0.410862466	2.29E-23	postive
HELLS	AC138150.1	0.538216004	8.31E-42	postive
TUBE1	AC138150.1	0.521176972	7.24E-39	postive
ALOX12	AC138150.1	0.507327478	1.35E-36	postive

IREB2	AC138150.1	0.410968327	2.22E-23	postive
GABPB1	AC138150.1	0.571620107	4.40E-48	postive
ATG7	AC138150.1	0.427347359	2.44E-25	postive
MAPK8	AC138150.1	0.422434679	9.69E-25	postive
LINC00472	AC138150.1	0.696849747	1.50E-79	postive
ATM	AC138150.1	0.740348153	1.10E-94	postive
FBXW7	AC138150.1	0.459361736	1.72E-29	postive
ZNF419	AC104463.2	0.439947174	6.39E-27	postive
ALOX12	AC104463.2	0.481111142	1.42E-32	postive
HBA1	AC104463.2	0.435934284	2.07E-26	postive
TAZ	AC104463.2	0.486952463	1.93E-33	postive
GCLC	AL137003.1	0.409600237	3.20E-23	postive
KLHL24	AL137003.1	0.411449467	1.95E-23	postive
TUBE1	AL137003.1	0.5907857	5.07E-52	postive
IREB2	AL137003.1	0.50495227	3.23E-36	postive
SP1	AL137003.1	0.408997185	3.76E-23	postive
GABPB1	AL137003.1	0.449425166	3.73E-28	postive
EMC2	AL137003.1	0.413102659	1.25E-23	postive
PIK3CA	AL137003.1	0.421526629	1.25E-24	postive
KRAS	AL137003.1	0.481376773	1.30E-32	postive
MAPK8	AL137003.1	0.559903833	8.44E-46	postive
LINC00472	AL137003.1	0.487713563	1.48E-33	postive
PRKAA2	AL137003.1	0.480088503	2.00E-32	postive
PRKAA1	AL137003.1	0.510677623	3.89E-37	postive
ATM	AL137003.1	0.554653471	8.32E-45	postive
SIRT1	AL137003.1	0.413434755	1.14E-23	postive
RB1	EBLN3P	0.433706095	3.96E-26	postive
HSPB1	EBLN3P	-0.442557791	2.95E-27	negative
GCLC	EBLN3P	0.49579347	8.76E-35	postive
ACSL3	EBLN3P	0.512688281	1.83E-37	postive
CHMP5	EBLN3P	0.525016778	1.63E-39	postive
OXSR1	EBLN3P	0.469088824	7.65E-31	postive
KLHL24	EBLN3P	0.620108578	1.39E-58	postive
TUBE1	EBLN3P	0.466518335	1.76E-30	postive
EIF2S1	EBLN3P	0.432125023	6.24E-26	postive
MAP3K5	EBLN3P	0.483383705	6.56E-33	postive
IREB2	EBLN3P	0.697374785	1.03E-79	postive
HMGB1	EBLN3P	0.419862719	1.98E-24	postive
GABPB1	EBLN3P	0.440293461	5.77E-27	postive
EMC2	EBLN3P	0.598564236	1.07E-53	postive
PIK3CA	EBLN3P	0.599492308	6.73E-54	postive
KRAS	EBLN3P	0.66703812	1.17E-70	postive
ATG5	EBLN3P	0.415919713	5.83E-24	postive
MAPK1	EBLN3P	0.40369342	1.52E-22	postive
MAPK8	EBLN3P	0.641022532	1.05E-63	postive
LINC00472	EBLN3P	0.463259312	5.00E-30	postive
PRKAA2	EBLN3P	0.558851735	1.34E-45	postive
PRKAA1	EBLN3P	0.682588715	3.65E-75	postive
TLR4	EBLN3P	0.423936174	6.37E-25	postive
ATM	EBLN3P	0.510669293	3.90E-37	postive
SIRT1	EBLN3P	0.556312287	4.05E-45	postive
HELLS	CRTC3-AS1	0.564695651	1.01E-46	postive
ALOX12	CRTC3-AS1	0.413869673	1.02E-23	postive
GABPB1	CRTC3-AS1	0.481958319	1.07E-32	postive
ATM	CRTC3-AS1	0.617322953	6.27E-58	postive
FBXW7	CRTC3-AS1	0.578954611	1.47E-49	postive
ZNF419	AC087741.1	0.463116928	5.23E-30	postive
TUBE1	AC087741.1	0.515144045	7.27E-38	postive

DRD4	AC087741.1	0.417650848	3.63E-24	postive
ALOX12	AC087741.1	0.613016557	6.23E-57	postive
PHKG2	AC087741.1	0.603154694	1.05E-54	postive
TAZ	AC087741.1	0.760716726	7.25E-103	postive
TFAP2C	AC006238.1	0.60685634	1.56E-55	postive
HBA1	AC006238.1	0.495687625	9.09E-35	postive
ALOX12	AL162595.1	0.419229021	2.36E-24	postive
ULK2	AL162595.1	0.446208462	9.87E-28	postive
ATM	AL162595.1	0.528262963	4.54E-40	postive
YY1AP1	AL162595.1	0.443971598	1.93E-27	postive
SLC1A4	AL136084.2	0.484370647	4.69E-33	postive
IL33	AL136084.2	0.400447433	3.54E-22	postive
ZEB1	AL136084.2	0.604224165	6.07E-55	postive
EPAS1	AL136084.2	0.552575079	2.03E-44	postive
CD44	AL390719.2	0.420007818	1.90E-24	postive
SOCS1	AL390719.2	0.66833795	5.02E-71	postive
PLIN2	AC087482.1	0.467517863	1.27E-30	postive
HELLS	WASHC5-AS	0.526749612	8.25E-40	postive
ZNF419	WASHC5-AS	0.425367782	4.27E-25	postive
TUBE1	WASHC5-AS	0.534953792	3.13E-41	postive
ALOX12	WASHC5-AS	0.522285281	4.72E-39	postive
GABPB1	WASHC5-AS	0.588863996	1.29E-51	postive
MAPK8	WASHC5-AS	0.434984844	2.73E-26	postive
LINC00472	WASHC5-AS	0.75955662	2.23E-102	postive
ATM	WASHC5-AS	0.750602763	1.05E-98	postive
FBXW7	WASHC5-AS	0.507869782	1.10E-36	postive
HRAS	AL139246.5	0.544105883	7.31E-43	postive
EGLN2	AL139246.5	0.440333516	5.70E-27	postive
ISCU	AC091151.1	0.41947923	2.20E-24	postive
ACSL3	AC091151.1	0.407958352	4.96E-23	postive
KLHL24	AC091151.1	0.603275584	9.86E-55	postive
PIK3CA	AC091151.1	0.403500287	1.60E-22	postive
GABARAPL1	AC091151.1	0.471079016	4.00E-31	postive
LINC00472	AC091151.1	0.677616803	1.08E-73	postive
LPIN1	AC091151.1	0.539366568	5.19E-42	postive
PHKG2	AC026471.2	0.462692134	5.98E-30	postive
TAZ	AC026471.2	0.535566005	2.44E-41	postive
DRD4	AC040977.1	0.401001377	3.07E-22	postive
ALOX12	AC040977.1	0.573408605	1.94E-48	postive
NOX1	AC040977.1	0.405537386	9.39E-23	postive
ALOX12B	AC040977.1	0.430252659	1.07E-25	postive
PHKG2	AC040977.1	0.405353223	9.86E-23	postive
TAZ	AC040977.1	0.493628119	1.88E-34	postive
MTDH	AC040977.1	-0.40458697	1.21E-22	negative
TFAP2C	AC079414.3	0.534219911	4.21E-41	postive
HBA1	AC079414.3	0.421883672	1.13E-24	postive
BRD4	AC022098.1	0.511547282	2.81E-37	postive
ZNF419	AC022098.1	0.604066264	6.58E-55	postive
VEGFA	AC022098.1	0.553748907	1.23E-44	postive
TUBE1	AC022098.1	0.463212958	5.07E-30	postive
SETD1B	AC022098.1	0.555070401	6.95E-45	postive
ALOX12	AC022098.1	0.597358697	1.96E-53	postive
GABPB1	AC022098.1	0.468326594	9.79E-31	postive
ZEB1	AC022098.1	0.413911098	1.01E-23	postive
YY1AP1	AC022098.1	0.41158862	1.88E-23	postive
TAZ	AC022098.1	0.457251735	3.33E-29	postive
FBXW7	AC022098.1	0.435528746	2.33E-26	postive
TXNIP	LINC00909	0.432639464	5.38E-26	postive

ZNF419	LINC00909	0.429732381	1.24E-25	postive
TUBE1	LINC00909	0.462984395	5.45E-30	postive
ALOX12	LINC00909	0.44254083	2.96E-27	postive
IREB2	LINC00909	0.408951289	3.81E-23	postive
HMGB1	LINC00909	0.445218308	1.33E-27	postive
GABPB1	LINC00909	0.478594916	3.31E-32	postive
ULK2	LINC00909	0.441405461	4.15E-27	postive
MAPK8	LINC00909	0.510812787	3.70E-37	postive
ATM	LINC00909	0.477216006	5.26E-32	postive
YY1AP1	LINC00909	0.414184364	9.34E-24	postive
SIRT1	LINC00909	0.41851256	2.87E-24	postive
TFAP2C	AC079210.1	0.479547786	2.41E-32	postive
EIF2AK4	PANTR1	-0.402454552	2.10E-22	negative
HRAS	PANTR1	0.42743184	2.38E-25	postive
KLHL24	ITFG1-AS1	0.601982258	1.91E-54	postive
IREB2	ITFG1-AS1	0.405551927	9.36E-23	postive
GABPB1	ITFG1-AS1	0.414080272	9.61E-24	postive
PIK3CA	ITFG1-AS1	0.449373236	3.79E-28	postive
KRAS	ITFG1-AS1	0.413123902	1.24E-23	postive
LINC00472	ITFG1-AS1	0.812684174	4.70E-128	postive
PRKAA1	ITFG1-AS1	0.417401396	3.89E-24	postive
LPIN1	ITFG1-AS1	0.496340613	7.21E-35	postive
ATM	ITFG1-AS1	0.442738872	2.79E-27	postive
BRD4	PTOV1-AS2	0.460044052	1.39E-29	postive
ZNF419	PTOV1-AS2	0.553774406	1.22E-44	postive
VEGFA	PTOV1-AS2	0.513109752	1.57E-37	postive
TUBE1	PTOV1-AS2	0.500428569	1.67E-35	postive
SETD1B	PTOV1-AS2	0.525016324	1.63E-39	postive
DRD4	PTOV1-AS2	0.467452931	1.30E-30	postive
ALOX12	PTOV1-AS2	0.582615577	2.61E-50	postive
NCOA4	PTOV1-AS2	-0.418333293	3.01E-24	negative
PHKG2	PTOV1-AS2	0.619522213	1.92E-58	postive
ULK1	PTOV1-AS2	0.405313105	9.96E-23	postive
TAZ	PTOV1-AS2	0.806269209	1.54E-124	postive
ZNF419	AL359504.1	0.490552005	5.54E-34	postive
VEGFA	AL359504.1	0.477762843	4.38E-32	postive
TUBE1	AL359504.1	0.545048626	4.93E-43	postive
ALOX12	AL359504.1	0.562748227	2.40E-46	postive
PHKG2	AL359504.1	0.483969871	5.37E-33	postive
TAZ	AL359504.1	0.716582061	4.62E-86	postive
ZNF419	AL390728.5	0.435075012	2.66E-26	postive
TUBE1	AL390728.5	0.566263729	4.99E-47	postive
NOX1	AL390728.5	0.463217733	5.06E-30	postive
PHKG2	AL390728.5	0.549752763	6.79E-44	postive
TAZ	AL390728.5	0.664067274	7.88E-70	postive
FBXW7	AL390728.5	0.403053811	1.80E-22	postive
HELLS	AC079313.1	0.414301632	9.05E-24	postive
ALOX12	AC079313.1	0.436305245	1.86E-26	postive
TFAP2C	AC079313.1	0.488965146	9.63E-34	postive
HBA1	AC079313.1	0.460117589	1.35E-29	postive
DUOX1	AC079313.1	0.416909972	4.45E-24	postive
ISCU	MAN2A1-DT	0.565814547	6.11E-47	postive
KLHL24	MAN2A1-DT	0.417478005	3.81E-24	postive
MAP1LC3A	MAN2A1-DT	0.45638703	4.36E-29	postive
GABARAPL1	MAN2A1-DT	0.537835628	9.71E-42	postive
LPIN1	MAN2A1-DT	0.444354027	1.72E-27	postive
ZNF419	AC007663.4	0.54329574	1.02E-42	postive
VEGFA	AC007663.4	0.430782388	9.18E-26	postive

TUBE1	AC007663.4	0.561208188	4.75E-46	postive
SETD1B	AC007663.4	0.469372644	6.97E-31	postive
ALOX12	AC007663.4	0.562554684	2.62E-46	postive
GABPB1	AC007663.4	0.541529616	2.13E-42	postive
MAPK8	AC007663.4	0.475580809	9.06E-32	postive
LINC00472	AC007663.4	0.618599484	3.15E-58	postive
ATM	AC007663.4	0.557631848	2.28E-45	postive
YY1AP1	AC007663.4	0.421700061	1.19E-24	postive
FBXW7	AC007663.4	0.41629261	5.27E-24	postive
KLHL24	AC064801.1	0.481904375	1.09E-32	postive
GABPB1	AC064801.1	0.498380169	3.48E-35	postive
LINC00472	AC064801.1	0.860483504	2.13E-159	postive
ATM	AC064801.1	0.523676552	2.75E-39	postive
HELLS	PVT1	0.434080483	3.55E-26	postive
PHKG2	PVT1	0.439395146	7.52E-27	postive
TAZ	PVT1	0.522479739	4.37E-39	postive
ALOX12	FAM160A1-[	0.423677599	6.85E-25	postive
ATM	FAM160A1-[	0.406879221	6.59E-23	postive
ALOX12	AL031722.1	0.403787546	1.49E-22	postive
TFAP2C	AL031722.1	0.472231302	2.74E-31	postive
HBA1	AL031722.1	0.427057219	2.65E-25	postive
HSPB1	HHLA3	0.440093469	6.12E-27	postive
IREB2	HHLA3	-0.488167902	1.27E-33	negative
PIK3CA	HHLA3	-0.458122553	2.53E-29	negative
KRAS	HHLA3	-0.414533377	8.50E-24	negative
NCOA4	HHLA3	-0.409645687	3.16E-23	negative
PHKG2	HHLA3	0.407005612	6.38E-23	postive
PRKAA1	HHLA3	-0.491603997	3.84E-34	negative
GABPB1	AC099811.3	0.428388469	1.82E-25	postive
ZNF419	AP006621.2	0.582178927	3.21E-50	postive
VEGFA	AP006621.2	0.431601462	7.26E-26	postive
TUBE1	AP006621.2	0.558171498	1.80E-45	postive
SETD1B	AP006621.2	0.442637669	2.88E-27	postive
ALOX12	AP006621.2	0.723956775	1.22E-88	postive
ATM	AP006621.2	0.490763723	5.15E-34	postive
TAZ	AP006621.2	0.530883678	1.61E-40	postive
FBXW7	AP006621.2	0.400318198	3.66E-22	postive
ZNF419	AC093752.2	0.423685887	6.84E-25	postive
VEGFA	AC093752.2	0.499363345	2.45E-35	postive
TUBE1	AC093752.2	0.49208459	3.24E-34	postive
SETD1B	AC093752.2	0.535597715	2.41E-41	postive
ALOX12	AC093752.2	0.618135549	4.05E-58	postive
TAZ	AC093752.2	0.49123976	4.36E-34	postive
CXCL2	AC245128.3	0.401734954	2.54E-22	postive
ISCU	LINC01607	0.468150669	1.04E-30	postive
ATG4D	LINC01607	0.434608509	3.05E-26	postive
GABARAPL2	LINC01607	0.564645609	1.03E-46	postive
CDKN2A	AC124067.2	0.416047936	5.63E-24	postive
HELLS	COL18A1-AS	0.488190983	1.26E-33	postive
ALOX12	COL18A1-AS	0.420226591	1.79E-24	postive
GABPB1	COL18A1-AS	0.507967267	1.06E-36	postive
ATG7	COL18A1-AS	0.406959382	6.46E-23	postive
LINC00472	COL18A1-AS	0.642050621	5.76E-64	postive
ATM	COL18A1-AS	0.681783905	6.34E-75	postive
FBXW7	COL18A1-AS	0.408438856	4.36E-23	postive
ZNF419	LINC01004	0.545594569	3.92E-43	postive
TUBE1	LINC01004	0.58598136	5.21E-51	postive
ALOX12	LINC01004	0.457144348	3.44E-29	postive

GABPB1	LINC01004	0.444079719	1.87E-27	postive
PHKG2	LINC01004	0.50588118	2.30E-36	postive
LINC00472	LINC01004	0.587147865	2.97E-51	postive
TAZ	LINC01004	0.562749219	2.40E-46	postive
PHKG2	NALT1	0.602791972	1.26E-54	postive
TAZ	NALT1	0.613334505	5.26E-57	postive
JDP2	AC004816.2	0.597481608	1.85E-53	postive
RGS4	AC004816.2	0.42326121	7.70E-25	postive
EIF2S1	AC004816.2	0.445172995	1.35E-27	postive
TFAP2C	AC027307.3	0.449491444	3.65E-28	postive
STEAP3	AL391095.2	0.467746883	1.18E-30	postive
HELLS	AC004477.2	0.428155257	1.94E-25	postive
TFAP2C	AC004477.2	0.402942001	1.85E-22	postive
ARNTL	SEPTIN7-DT	0.434052967	3.58E-26	postive
VEGFA	SEPTIN7-DT	0.45080908	2.44E-28	postive
TUBE1	SEPTIN7-DT	0.546131806	3.13E-43	postive
ALOX12	SEPTIN7-DT	0.504980282	3.19E-36	postive
IREB2	SEPTIN7-DT	0.532834681	7.35E-41	postive
SP1	SEPTIN7-DT	0.402876681	1.89E-22	postive
GABPB1	SEPTIN7-DT	0.41124634	2.06E-23	postive
MAPK8	SEPTIN7-DT	0.646744706	3.56E-65	postive
PRKAA2	SEPTIN7-DT	0.5123019	2.12E-37	postive
PRKAA1	SEPTIN7-DT	0.457367095	3.21E-29	postive
ATM	SEPTIN7-DT	0.699158487	2.77E-80	postive
SIRT1	SEPTIN7-DT	0.518500825	2.03E-38	postive
TFAP2C	AC009119.1	0.560154756	7.56E-46	postive
HBA1	AC009119.1	0.515280707	6.90E-38	postive
ACSL3	AL078581.2	0.454776543	7.19E-29	postive
KLHL24	AL078581.2	0.521255483	7.03E-39	postive
IREB2	AL078581.2	0.52003278	1.13E-38	postive
PIK3CA	AL078581.2	0.494785295	1.25E-34	postive
KRAS	AL078581.2	0.425352583	4.28E-25	postive
LINC00472	AL078581.2	0.568402747	1.90E-47	postive
LPIN1	AL078581.2	0.430877979	8.93E-26	postive
HELLS	FBXO30-DT	0.468483845	9.31E-31	postive
MTOR	FBXO30-DT	0.417110934	4.21E-24	postive
KLHL24	FBXO30-DT	0.508550065	8.58E-37	postive
TUBE1	FBXO30-DT	0.48783343	1.42E-33	postive
MAP3K5	FBXO30-DT	0.501201904	1.26E-35	postive
IREB2	FBXO30-DT	0.55476703	7.92E-45	postive
GABPB1	FBXO30-DT	0.525986843	1.11E-39	postive
PIK3CA	FBXO30-DT	0.505378122	2.76E-36	postive
ATG5	FBXO30-DT	0.420014422	1.90E-24	postive
ATG7	FBXO30-DT	0.470116131	5.47E-31	postive
MAPK8	FBXO30-DT	0.515803043	5.66E-38	postive
LINC00472	FBXO30-DT	0.644535998	1.33E-64	postive
PRKAA2	FBXO30-DT	0.507208022	1.41E-36	postive
PRKAA1	FBXO30-DT	0.446888457	8.04E-28	postive
TLR4	FBXO30-DT	0.41295047	1.30E-23	postive
ATM	FBXO30-DT	0.73654403	3.05E-93	postive
FBXW7	FBXO30-DT	0.464875943	2.98E-30	postive
BACH1	FBXO30-DT	0.402272289	2.21E-22	postive
HELLS	XPC-AS1	0.497126343	5.45E-35	postive
ZNF419	XPC-AS1	0.574300641	1.28E-48	postive
VEGFA	XPC-AS1	0.410647221	2.42E-23	postive
TUBE1	XPC-AS1	0.663682354	1.01E-69	postive
SETD1B	XPC-AS1	0.490783942	5.11E-34	postive
ALOX12	XPC-AS1	0.714295672	2.80E-85	postive

GABPB1	XPC-AS1	0.582955824	2.22E-50	postive
MAPK8	XPC-AS1	0.43014812	1.10E-25	postive
LINC00472	XPC-AS1	0.621680098	5.93E-59	postive
ATM	XPC-AS1	0.706165203	1.48E-82	postive
FBXW7	XPC-AS1	0.539109696	5.77E-42	postive
FANCD2	CCDC18-AS1	0.403297529	1.69E-22	postive
HELLS	CCDC18-AS1	0.460268929	1.29E-29	postive
ZNF419	CCDC18-AS1	0.549053885	9.13E-44	postive
VEGFA	CCDC18-AS1	0.418455238	2.91E-24	postive
TUBE1	CCDC18-AS1	0.641516079	7.89E-64	postive
SETD1B	CCDC18-AS1	0.419687456	2.08E-24	postive
ALOX12	CCDC18-AS1	0.703391966	1.20E-81	postive
GABPB1	CCDC18-AS1	0.431352717	7.79E-26	postive
PHKG2	CCDC18-AS1	0.462102587	7.22E-30	postive
ATM	CCDC18-AS1	0.506773649	1.65E-36	postive
TAZ	CCDC18-AS1	0.553216244	1.55E-44	postive
FBXW7	CCDC18-AS1	0.476213809	7.34E-32	postive
HELLS	AL359962.2	0.480710289	1.63E-32	postive
ALOX12	AL359962.2	0.425202685	4.47E-25	postive
ATM	AL359962.2	0.513067104	1.59E-37	postive
TAZ	AL033384.2	0.41979429	2.02E-24	postive
SLC3A2	AC009779.2	0.565067889	8.54E-47	postive
NFS1	AC009779.2	0.453028913	1.23E-28	postive
FH	AC009779.2	0.51762978	2.83E-38	postive
ISCU	AC009779.2	0.697026431	1.32E-79	postive
DDIT3	AC009779.2	0.539907399	4.16E-42	postive
HERPUD1	AC009779.2	0.421682259	1.19E-24	postive
SLC2A8	AC009779.2	0.425466343	4.15E-25	postive
SLC2A12	AC009779.2	0.427557504	2.30E-25	postive
CS	AC009779.2	0.45809015	2.56E-29	postive
GOT1	AC009779.2	0.510269199	4.53E-37	postive
ATG4D	AC009779.2	0.636264779	1.67E-62	postive
GABARAPL1	AC009779.2	0.733332412	4.83E-92	postive
WIPI2	AC009779.2	0.54773711	1.59E-43	postive
LPIN1	AC009779.2	0.589570974	9.18E-52	postive
HELLS	AL359880.1	0.537385339	1.17E-41	postive
TUBE1	AL359880.1	0.445279281	1.31E-27	postive
GABPB1	AL359880.1	0.559115537	1.19E-45	postive
LINC00472	AL359880.1	0.745756345	8.83E-97	postive
ATM	AL359880.1	0.661952341	3.03E-69	postive
FBXW7	AL359880.1	0.466962661	1.52E-30	postive
HELLS	AL050343.2	0.520913709	8.02E-39	postive
ZNF419	AL050343.2	0.459920559	1.44E-29	postive
ZFP69B	AL050343.2	0.407336751	5.84E-23	postive
TUBE1	AL050343.2	0.612679875	7.44E-57	postive
ALOX12	AL050343.2	0.582416707	2.87E-50	postive
IREB2	AL050343.2	0.414231156	9.22E-24	postive
GABPB1	AL050343.2	0.592996498	1.71E-52	postive
MAPK8	AL050343.2	0.510043108	4.93E-37	postive
LINC00472	AL050343.2	0.575846964	6.27E-49	postive
ATM	AL050343.2	0.78220997	1.94E-112	postive
FBXW7	AL050343.2	0.537715789	1.02E-41	postive
HELLS	AC011477.1	0.507340617	1.34E-36	postive
ZNF419	AC011477.1	0.590150397	6.92E-52	postive
KLHL24	AC011477.1	0.500242776	1.78E-35	postive
TUBE1	AC011477.1	0.603831702	7.42E-55	postive
ALOX12	AC011477.1	0.534275935	4.12E-41	postive
IREB2	AC011477.1	0.435836838	2.13E-26	postive

GABPB1	AC011477.1	0.649019561	9.06E-66	postive
MAPK8	AC011477.1	0.499585775	2.26E-35	postive
LINC00472	AC011477.1	0.669213441	2.84E-71	postive
ATM	AC011477.1	0.742383779	1.82E-95	postive
FBXW7	AC011477.1	0.5894828	9.58E-52	postive
FANCD2	AC073896.3	0.422401408	9.78E-25	postive
TFAP2C	AC073896.3	0.461087683	9.96E-30	postive
DUOX1	AC073896.3	0.422728566	8.93E-25	postive
FANCD2	LINC01134	0.401392383	2.77E-22	postive
TFAP2C	LINC01134	0.406986925	6.41E-23	postive
HRAS	AC104316.2	0.414003664	9.81E-24	postive
EGLN2	AC104316.2	0.477979725	4.07E-32	postive
HSPB1	SNHG25	0.480135125	1.97E-32	postive
HRAS	SNHG25	0.571454478	4.75E-48	postive
PHKG2	SNHG25	0.488203563	1.25E-33	postive
EGLN2	SNHG25	0.559093389	1.20E-45	postive
TAZ	SNHG25	0.443473957	2.24E-27	postive
HELLS	NRIR	0.449434408	3.72E-28	postive
ZNF419	NRIR	0.438996204	8.46E-27	postive
TUBE1	NRIR	0.575597252	7.04E-49	postive
ALOX12	NRIR	0.593739762	1.19E-52	postive
GABPB1	NRIR	0.461225502	9.53E-30	postive
ATM	NRIR	0.52062949	8.95E-39	postive
FBXW7	NRIR	0.47504708	1.08E-31	postive
HELLS	Z98884.1	0.442178592	3.30E-27	postive
ZNF419	Z98884.1	0.457998847	2.63E-29	postive
TUBE1	Z98884.1	0.52494867	1.67E-39	postive
ALOX12	Z98884.1	0.572474052	2.97E-48	postive
GABPB1	Z98884.1	0.498807462	2.99E-35	postive
MAPK8	Z98884.1	0.407194745	6.07E-23	postive
LINC00472	Z98884.1	0.583953153	1.38E-50	postive
ATM	Z98884.1	0.65170377	1.78E-66	postive
FBXW7	Z98884.1	0.417917873	3.38E-24	postive
HSPB1	BNC2-AS1	0.485330715	3.37E-33	postive
HRAS	BNC2-AS1	0.597011787	2.34E-53	postive
PHKG2	BNC2-AS1	0.459836792	1.48E-29	postive
BECN1	BNC2-AS1	-0.400937895	3.12E-22	negative
EGLN2	BNC2-AS1	0.469937172	5.80E-31	postive
TAZ	BNC2-AS1	0.477994926	4.05E-32	postive
TFAP2C	AC107959.1	0.451615078	1.91E-28	postive
HBA1	AC107959.1	0.43484653	2.84E-26	postive
ZFP69B	AC107021.1	0.415992931	5.72E-24	postive
ATG7	AC107021.1	0.434866145	2.83E-26	postive
TGFBR1	AC107021.1	0.401542521	2.67E-22	postive
ATM	AC107021.1	0.601466921	2.48E-54	postive
MTDH	AC107021.1	0.404453328	1.25E-22	postive
BACH1	AC107021.1	0.594221595	9.36E-53	postive
GDF15	LINC01558	0.436595977	1.71E-26	postive
HELLS	AC127070.2	0.464131309	3.78E-30	postive
TFAP2C	AC127070.2	0.410735107	2.36E-23	postive
ZNF419	AC009107.2	0.510469757	4.21E-37	postive
VEGFA	AC009107.2	0.452666385	1.38E-28	postive
TUBE1	AC009107.2	0.570395137	7.70E-48	postive
SETD1B	AC009107.2	0.443094946	2.51E-27	postive
ALOX12	AC009107.2	0.568380087	1.92E-47	postive
GABPB1	AC009107.2	0.411083015	2.15E-23	postive
ATM	AC009107.2	0.451299384	2.10E-28	postive
TAZ	AC009107.2	0.439089596	8.23E-27	postive

FBXW7	AC009107.2	0.433461219	4.25E-26	postive
FANCD2	POLH-AS1	0.468658209	8.79E-31	postive
HELLS	POLH-AS1	0.561039501	5.12E-46	postive
ALOX12	POLH-AS1	0.480233159	1.91E-32	postive
DUOX1	POLH-AS1	0.418116501	3.20E-24	postive
ZEB1	LINC01738	0.477866206	4.23E-32	postive
EPAS1	LINC01738	0.578126353	2.17E-49	postive
HELLS	LINC01389	0.522868709	3.76E-39	postive
ZNF419	LINC01389	0.504612237	3.65E-36	postive
TUBE1	LINC01389	0.504370043	3.99E-36	postive
ALOX12	LINC01389	0.542437854	1.46E-42	postive
GABPB1	LINC01389	0.499004114	2.78E-35	postive
ATM	LINC01389	0.471511955	3.47E-31	postive
TAZ	LINC01389	0.439192504	7.98E-27	postive
FBXW7	LINC01389	0.48654141	2.22E-33	postive
G6PD	GAPLINC	0.409086961	3.67E-23	postive
ZNF419	AL359715.1	0.51498274	7.73E-38	postive
TUBE1	AL359715.1	0.536230607	1.87E-41	postive
ALOX12	AL359715.1	0.454542207	7.74E-29	postive
LINC00472	AL359715.1	0.437491543	1.32E-26	postive
TAZ	AL359715.1	0.408796992	3.97E-23	postive
PHKG2	LINC01431	0.596028078	3.82E-53	postive
ATG4D	LINC01431	0.418512114	2.87E-24	postive
TAZ	LINC01431	0.582732865	2.47E-50	postive
HELLS	Z98884.2	0.512058123	2.32E-37	postive
ZNF419	Z98884.2	0.607718124	1.00E-55	postive
TUBE1	Z98884.2	0.656033475	1.24E-67	postive
SETD1B	Z98884.2	0.446794505	8.27E-28	postive
ALOX12	Z98884.2	0.672006565	4.55E-72	postive
GABPB1	Z98884.2	0.586280579	4.52E-51	postive
MAPK8	Z98884.2	0.427837678	2.12E-25	postive
LINC00472	Z98884.2	0.519919654	1.18E-38	postive
ATM	Z98884.2	0.617098435	7.08E-58	postive
FBXW7	Z98884.2	0.542009712	1.75E-42	postive
HELLS	AP005131.2	0.517366169	3.13E-38	postive
TUBE1	AP005131.2	0.413893161	1.01E-23	postive
ALOX12	AP005131.2	0.413781575	1.04E-23	postive
GABPB1	AP005131.2	0.526861225	7.90E-40	postive
LINC00472	AP005131.2	0.503887034	4.76E-36	postive
ATM	AP005131.2	0.670322005	1.38E-71	postive
FBXW7	AP005131.2	0.486229411	2.48E-33	postive
HELLS	AC145207.5	0.493710597	1.83E-34	postive
HBA1	AC145207.5	0.418935955	2.55E-24	postive
PCK2	DHRS4-AS1	0.498492876	3.34E-35	postive
AGPAT3	DHRS4-AS1	0.429916079	1.18E-25	postive
HELLS	AC025580.3	0.511563769	2.80E-37	postive
TUBE1	AC025580.3	0.451842007	1.78E-28	postive
ALOX12	AC025580.3	0.52443086	2.05E-39	postive
GABPB1	AC025580.3	0.4392289	7.90E-27	postive
LINC00472	AC025580.3	0.404398274	1.27E-22	postive
ATM	AC025580.3	0.695673829	3.54E-79	postive
FBXW7	AC025580.3	0.42127439	1.34E-24	postive
PCK2	LINC02754	0.420322204	1.74E-24	postive
ALOX12	LINC02754	0.415048605	7.39E-24	postive
PEBP1	LINC02754	0.408450947	4.35E-23	postive
MIOX	LINC02754	0.541868908	1.85E-42	postive
HELLS	AL162274.1	0.486166961	2.53E-33	postive
TUBE1	AL162274.1	0.49633694	7.22E-35	postive

ALOX12	AL162274.1	0.617879362	4.65E-58	postive
GABPB1	AL162274.1	0.445494262	1.22E-27	postive
MAPK8	AL162274.1	0.476707356	6.23E-32	postive
LINC00472	AL162274.1	0.448386645	5.11E-28	postive
ATM	AL162274.1	0.743365369	7.57E-96	postive
FBXW7	AL162274.1	0.47289187	2.20E-31	postive
SRXN1	LINC02709	0.478887969	3.00E-32	postive
FANCD2	NFE2L1-DT	0.436919949	1.55E-26	postive
HELLS	NFE2L1-DT	0.431071072	8.45E-26	postive
ZNF419	NFE2L1-DT	0.509230352	6.67E-37	postive
TUBE1	NFE2L1-DT	0.441055946	4.60E-27	postive
ALOX12	NFE2L1-DT	0.579469167	1.15E-49	postive
PHKG2	NFE2L1-DT	0.532533809	8.30E-41	postive
TAZ	NFE2L1-DT	0.59946396	6.82E-54	postive
PLIN2	AC009060.1	0.411971172	1.70E-23	postive
BNIP3	AC009060.1	0.421018101	1.44E-24	postive
ALB	DCST1-AS1	0.519968775	1.15E-38	postive
GPX2	DCST1-AS1	0.418388854	2.97E-24	postive
PSAT1	DCST1-AS1	0.407239785	6.00E-23	postive
TF	DCST1-AS1	0.49042012	5.80E-34	postive
TFR2	DCST1-AS1	0.491723276	3.68E-34	postive
CDO1	DCST1-AS1	0.493131075	2.24E-34	postive
ATM	AC012464.1	0.511634693	2.72E-37	postive
ZNF419	AL391834.1	0.540714392	2.98E-42	postive
KLHL24	AL391834.1	0.464215776	3.68E-30	postive
TUBE1	AL391834.1	0.64066111	1.30E-63	postive
ALOX12	AL391834.1	0.483355506	6.63E-33	postive
IREB2	AL391834.1	0.403261722	1.71E-22	postive
GABPB1	AL391834.1	0.610866442	1.93E-56	postive
KRAS	AL391834.1	0.428733235	1.65E-25	postive
MAPK8	AL391834.1	0.531257395	1.38E-40	postive
LINC00472	AL391834.1	0.658081984	3.46E-68	postive
PRKAA1	AL391834.1	0.454439705	7.99E-29	postive
ATM	AL391834.1	0.606531579	1.85E-55	postive
FBXW7	AL391834.1	0.460781727	1.10E-29	postive
ALOX12	AC022211.2	0.409537163	3.26E-23	postive
YY1AP1	AC022211.2	0.400913227	3.14E-22	postive
ZNF419	AC025165.4	0.468840973	8.29E-31	postive
ALOX12	AC025165.4	0.469580421	6.52E-31	postive
ATM	AC025165.4	0.441480564	4.06E-27	postive
TAZ	AC025165.4	0.438179437	1.08E-26	postive
TFAP2C	AC090970.1	0.583840329	1.45E-50	postive
HBA1	AC090970.1	0.55459878	8.52E-45	postive
DUOX1	AC090970.1	0.415463297	6.60E-24	postive
TUBE1	AC104938.1	0.455053958	6.60E-29	postive
ALOX12	AC104938.1	0.404097657	1.37E-22	postive
MAPK8	AC104938.1	0.435661077	2.24E-26	postive
HELLS	AC007000.3	0.518783972	1.82E-38	postive
TUBE1	AC007000.3	0.529005382	3.39E-40	postive
ALOX12	AC007000.3	0.525897412	1.15E-39	postive
IREB2	AC007000.3	0.417466064	3.82E-24	postive
GABPB1	AC007000.3	0.566419341	4.66E-47	postive
ATG7	AC007000.3	0.476896633	5.85E-32	postive
MAPK8	AC007000.3	0.421086297	1.41E-24	postive
LINC00472	AC007000.3	0.614391588	3.00E-57	postive
TLR4	AC007000.3	0.408124135	4.74E-23	postive
ATM	AC007000.3	0.786592099	1.59E-114	postive
FBXW7	AC007000.3	0.527828388	5.40E-40	postive

HELLS	AL021707.4	0.484584975	4.35E-33	postive
ZNF419	AL021707.4	0.573489415	1.87E-48	postive
TUBE1	AL021707.4	0.609046231	5.01E-56	postive
SETD1B	AL021707.4	0.455092362	6.52E-29	postive
ALOX12	AL021707.4	0.700104241	1.38E-80	postive
GABPB1	AL021707.4	0.468317937	9.82E-31	postive
ATM	AL021707.4	0.637793895	6.90E-63	postive
FBXW7	AL021707.4	0.48538585	3.31E-33	postive
TFAP2C	LINC02550	0.482802542	8.00E-33	postive
VEGFA	LINCR-0001	0.415352615	6.80E-24	postive
TUBE1	LINCR-0001	0.62860757	1.29E-60	postive
ALOX12	LINCR-0001	0.592228637	2.50E-52	postive
ATM	LINCR-0001	0.518022592	2.43E-38	postive
TAZ	LINCR-0001	0.420789949	1.53E-24	postive
ALOX12	AC004687.1	0.457676678	2.91E-29	postive
PHKG2	AC004687.1	0.495555883	9.53E-35	postive
TAZ	AC004687.1	0.548636399	1.09E-43	postive
HELLS	AC141002.1	0.505230664	2.91E-36	postive
ZNF419	AC141002.1	0.491573236	3.88E-34	postive
TUBE1	AC141002.1	0.493805815	1.77E-34	postive
ALOX12	AC141002.1	0.574289652	1.29E-48	postive
GABPB1	AC141002.1	0.544188725	7.06E-43	postive
MAPK8	AC141002.1	0.441479857	4.06E-27	postive
LINC00472	AC141002.1	0.626475091	4.23E-60	postive
ATM	AC141002.1	0.605715734	2.82E-55	postive
FBXW7	AC141002.1	0.488659569	1.07E-33	postive
ZNF419	CTC-338M12	0.417543136	3.74E-24	postive
TUBE1	CTC-338M12	0.404717833	1.17E-22	postive
ALOX12	CTC-338M12	0.520806505	8.36E-39	postive
TAZ	CTC-338M12	0.456813551	3.82E-29	postive
HELLS	AC131009.3	0.40071883	3.30E-22	postive
ZNF419	AC131009.3	0.409641076	3.17E-23	postive
ALOX12	AC131009.3	0.417678203	3.61E-24	postive
PHKG2	AC131009.3	0.420247281	1.78E-24	postive
EGLN2	AC131009.3	0.5896235	8.95E-52	postive
TAZ	AC131009.3	0.45319837	1.17E-28	postive
HELLS	ZBTB40-IT1	0.489459123	8.11E-34	postive
ZNF419	ZBTB40-IT1	0.461773275	8.01E-30	postive
TUBE1	ZBTB40-IT1	0.579574251	1.10E-49	postive
ALOX12	ZBTB40-IT1	0.601420071	2.54E-54	postive
GABPB1	ZBTB40-IT1	0.54629562	2.92E-43	postive
MAPK8	ZBTB40-IT1	0.442577645	2.93E-27	postive
LINC00472	ZBTB40-IT1	0.517779986	2.67E-38	postive
ATM	ZBTB40-IT1	0.725926021	2.42E-89	postive
FBXW7	ZBTB40-IT1	0.542704178	1.31E-42	postive
FANCD2	DLGAP1-AS2	0.511380198	2.99E-37	postive
HELLS	DLGAP1-AS2	0.466772177	1.62E-30	postive
BRD4	DLGAP1-AS2	0.435486356	2.36E-26	postive
ASNS	DLGAP1-AS2	0.440750832	5.04E-27	postive
G6PD	DLGAP1-AS2	0.421789095	1.16E-24	postive
SCP2	DLGAP1-AS2	-0.406335876	7.61E-23	negative
PHKG2	DLGAP1-AS2	0.568523944	1.80E-47	postive
TAZ	DLGAP1-AS2	0.590007387	7.42E-52	postive
RB1	AC073073.2	0.537204263	1.26E-41	postive
TUBE1	AC073073.2	0.428778929	1.63E-25	postive
IREB2	AC073073.2	0.448588559	4.81E-28	postive
HMGB1	AC073073.2	0.428760295	1.63E-25	postive
SP1	AC073073.2	0.468519085	9.20E-31	postive

GABPB1	AC073073.2	0.426483277	3.12E-25	postive
NRAS	AC073073.2	0.43870284	9.22E-27	postive
KRAS	AC073073.2	0.440596135	5.28E-27	postive
MAPK8	AC073073.2	0.517345592	3.15E-38	postive
PRKAA2	AC073073.2	0.511655325	2.70E-37	postive
PRKAA1	AC073073.2	0.510232486	4.60E-37	postive
ATM	AC073073.2	0.538364469	7.82E-42	postive
SIRT1	AC073073.2	0.504960207	3.22E-36	postive
LINC00472	AL355472.3	0.649372796	7.32E-66	postive
HELLS	AC020978.3	0.532960093	6.99E-41	postive
ZNF419	AC020978.3	0.409923775	2.94E-23	postive
TUBE1	AC020978.3	0.492066866	3.26E-34	postive
ALOX12	AC020978.3	0.498001558	3.99E-35	postive
GABPB1	AC020978.3	0.496144465	7.73E-35	postive
LINC00472	AC020978.3	0.577467909	2.95E-49	postive
ATM	AC020978.3	0.529361518	2.94E-40	postive
FBXW7	AC020978.3	0.503056878	6.44E-36	postive
AGPAT3	SH3RF3-AS1	0.46252947	6.30E-30	postive
NFS1	MIR4435-2H	-0.427354166	2.44E-25	negative
SLC2A6	MIR4435-2H	0.459569483	1.61E-29	postive
HIC1	MIR4435-2H	0.443163562	2.46E-27	postive
CS	MIR4435-2H	-0.402175849	2.26E-22	negative
LPCAT3	MIR4435-2H	-0.471411713	3.58E-31	negative
LINC00472	AC135048.3	0.472119075	2.84E-31	postive
SLC3A2	GTSE1-DT	0.469821482	6.02E-31	postive
ISCU	GTSE1-DT	0.571760691	4.13E-48	postive
DDIT3	GTSE1-DT	0.50558132	2.56E-36	postive
ATG4D	GTSE1-DT	0.558968538	1.27E-45	postive
MAP1LC3A	GTSE1-DT	0.425463318	4.15E-25	postive
GABARAPL1	GTSE1-DT	0.58523488	7.46E-51	postive
WIPI2	GTSE1-DT	0.437729793	1.23E-26	postive
BAP1	GTSE1-DT	0.468130523	1.04E-30	postive
LPIN1	GTSE1-DT	0.478772595	3.12E-32	postive
HELLS	ABALON	0.464770021	3.08E-30	postive
MTOR	ABALON	0.419414178	2.24E-24	postive
HIF1A	ABALON	0.411964901	1.70E-23	postive
ATG7	ABALON	0.597113883	2.22E-53	postive
LINC00472	ABALON	0.440360045	5.66E-27	postive
ATM	ABALON	0.446518792	8.99E-28	postive
HELLS	AC005332.7	0.50051321	1.62E-35	postive
ZNF419	AC005332.7	0.446622721	8.71E-28	postive
TUBE1	AC005332.7	0.600540173	3.96E-54	postive
ALOX12	AC005332.7	0.614517334	2.81E-57	postive
GABPB1	AC005332.7	0.508262131	9.55E-37	postive
ATM	AC005332.7	0.634017272	6.05E-62	postive
FBXW7	AC005332.7	0.512124072	2.27E-37	postive
FANCD2	LINC00426	0.533674274	5.25E-41	postive
HELLS	LINC00426	0.419288563	2.32E-24	postive
PML	LINC00426	0.440825699	4.93E-27	postive
GCH1	LINC00426	0.513055123	1.60E-37	postive
IFNG	LINC00426	0.710473476	5.49E-84	postive
TNFAIP3	LINC00426	0.430644997	9.55E-26	postive
ZNF419	LINC01176	0.454400223	8.08E-29	postive
VEGFA	LINC01176	0.460035914	1.39E-29	postive
TUBE1	LINC01176	0.534443129	3.85E-41	postive
ALOX12	LINC01176	0.536652699	1.57E-41	postive
PHKG2	LINC01176	0.562829693	2.32E-46	postive
TAZ	LINC01176	0.743899084	4.69E-96	postive

MAPK8	LINC01132	0.4056479	9.12E-23	postive
EPAS1	LINC01132	0.468367088	9.67E-31	postive
GCH1	BHLHE40-AS	0.565996997	5.63E-47	postive
IFNG	BHLHE40-AS	0.705444146	2.55E-82	postive
TNFAIP3	BHLHE40-AS	0.408843537	3.92E-23	postive
HRAS	AC015802.5	0.500666563	1.53E-35	postive
PHKG2	AC015802.5	0.622638862	3.51E-59	postive
EGLN2	AC015802.5	0.571596373	4.45E-48	postive
TAZ	AC015802.5	0.667947131	6.47E-71	postive
KLHL24	EP300-AS1	0.496947695	5.81E-35	postive
TUBE1	EP300-AS1	0.446419288	9.27E-28	postive
IREB2	EP300-AS1	0.456735135	3.91E-29	postive
GABPB1	EP300-AS1	0.471192733	3.85E-31	postive
PIK3CA	EP300-AS1	0.428259099	1.88E-25	postive
KRAS	EP300-AS1	0.423877084	6.48E-25	postive
MAPK8	EP300-AS1	0.483898102	5.51E-33	postive
LINC00472	EP300-AS1	0.851275897	1.61E-152	postive
ATM	EP300-AS1	0.575923697	6.05E-49	postive
KLHL24	MALINC1	0.433446571	4.27E-26	postive
TUBE1	MALINC1	0.449535941	3.60E-28	postive
IREB2	MALINC1	0.435523465	2.34E-26	postive
EMC2	MALINC1	0.444062058	1.88E-27	postive
MAPK8	MALINC1	0.410221866	2.71E-23	postive
MAPK9	MALINC1	0.423618507	6.97E-25	postive
PRKAA1	MALINC1	0.469680712	6.31E-31	postive
ATM	MALINC1	0.471807305	3.15E-31	postive
KLHL24	AC025280.1	0.519796657	1.23E-38	postive
GABPB1	AC025280.1	0.480397415	1.81E-32	postive
PIK3CA	AC025280.1	0.42558445	4.01E-25	postive
LINC00472	AC025280.1	0.902446585	1.54E-198	postive
ATM	AC025280.1	0.458827274	2.03E-29	postive
TUBE1	AL135791.1	0.530803025	1.66E-40	postive
ALOX12	AL135791.1	0.598849665	9.30E-54	postive
ATM	AL135791.1	0.52458212	1.93E-39	postive
PHKG2	AC022079.2	0.433645366	4.03E-26	postive
TAZ	AC022079.2	0.469811534	6.04E-31	postive
TUBE1	AL360181.1	0.521090699	7.49E-39	postive
ALOX12	AL360181.1	0.570578678	7.08E-48	postive
ATM	AL360181.1	0.400331349	3.65E-22	postive
TAZ	AL360181.1	0.414064552	9.65E-24	postive
CHMP6	BX537318.1	0.42707726	2.63E-25	postive
PIK3CA	BX537318.1	-0.456440914	4.29E-29	negative
KRAS	BX537318.1	-0.402776131	1.94E-22	negative
PHKG2	BX537318.1	0.431267388	7.99E-26	postive
TGFBR1	BX537318.1	-0.416170773	5.45E-24	negative
TLR4	BX537318.1	-0.426437612	3.16E-25	negative
TAZ	BX537318.1	0.473845642	1.61E-31	postive
ALB	LINC01018	0.862322303	7.86E-161	postive
GPX2	LINC01018	0.6288769	1.11E-60	postive
TF	LINC01018	0.575692097	6.74E-49	postive
TFR2	LINC01018	0.702822747	1.83E-81	postive
CDO1	LINC01018	0.817625738	7.40E-131	postive
KLHL24	LINC02041	0.471237331	3.79E-31	postive
LINC00472	LINC02041	0.446622074	8.72E-28	postive
KLHL24	LINC02006	0.560482711	6.54E-46	postive
PIK3CA	LINC02006	0.41595233	5.78E-24	postive
LINC00472	LINC02006	0.843314337	6.16E-147	postive
ZNF419	AL355297.3	0.435772587	2.17E-26	postive

TUBE1	AL355297.3	0.600114822	4.91E-54	postive
ALOX12	AL355297.3	0.567673897	2.65E-47	postive
GABPB1	AL355297.3	0.484271427	4.85E-33	postive
LINC00472	AL355297.3	0.480175309	1.95E-32	postive
ATM	AL355297.3	0.483202622	6.98E-33	postive
PHKG2	LINC02615	0.431190166	8.17E-26	postive
TAZ	LINC02615	0.499727981	2.15E-35	postive
FBXW7	LINC02615	0.400330896	3.65E-22	postive
ATM	SNHG26	0.432901639	4.99E-26	postive
PHKG2	AP003392.5	0.4575165	3.06E-29	postive
TAZ	AP003392.5	0.544731329	5.63E-43	postive
FANCD2	AC015802.3	0.401581984	2.64E-22	postive
ALOX12	AC015802.3	0.516916006	3.71E-38	postive
TFAP2C	AC015802.3	0.465092303	2.78E-30	postive
HBA1	AC015802.3	0.422869894	8.59E-25	postive
DUOX1	AC015802.3	0.402947497	1.85E-22	postive
FANCD2	TMPO-AS1	0.543601989	9.02E-43	postive
HELLS	TMPO-AS1	0.438098482	1.10E-26	postive
RRM2	TMPO-AS1	0.47635852	6.99E-32	postive
AURKA	TMPO-AS1	0.509121057	6.95E-37	postive
TAZ	TMPO-AS1	0.400346692	3.64E-22	postive
HIC1	SOCAR	0.421389992	1.30E-24	postive
GABPB1	SOCAR	0.459426877	1.68E-29	postive
HELLS	NLGN1-AS1	0.516142699	4.98E-38	postive
GABPB1	NLGN1-AS1	0.477467967	4.83E-32	postive
ATG7	NLGN1-AS1	0.473972618	1.54E-31	postive
LINC00472	NLGN1-AS1	0.451025569	2.29E-28	postive
ATM	NLGN1-AS1	0.695675456	3.53E-79	postive
FBXW7	NLGN1-AS1	0.44457282	1.61E-27	postive
ELAVL1	AL928921.1	0.404266477	1.31E-22	postive
WIPI2	AL928921.1	0.424921129	4.84E-25	postive
IDH1	AL928921.1	0.416189075	5.42E-24	postive
HRAS	AC068338.3	0.475585397	9.05E-32	postive
EGLN2	AC068338.3	0.492518977	2.78E-34	postive
ALOX12	AL451042.1	0.441787955	3.71E-27	postive
ATM	AL451042.1	0.432257352	6.01E-26	postive
FBXW7	AL451042.1	0.405084661	1.06E-22	postive
VEGFA	AC004771.2	0.419365251	2.27E-24	postive
SETD1B	AC004771.2	0.504564452	3.72E-36	postive
ALOX12	AC004771.2	0.527515689	6.10E-40	postive
ULK2	AC004771.2	0.42337083	7.47E-25	postive
YY1AP1	AC004771.2	0.400952317	3.11E-22	postive
FANCD2	AC126118.1	0.465749255	2.25E-30	postive
HELLS	AC126118.1	0.553162536	1.58E-44	postive
ZNF419	AC126118.1	0.57049315	7.36E-48	postive
TUBE1	AC126118.1	0.585270745	7.34E-51	postive
ALOX12	AC126118.1	0.61973425	1.71E-58	postive
GABPB1	AC126118.1	0.485815301	2.86E-33	postive
PHKG2	AC126118.1	0.409375233	3.40E-23	postive
ATM	AC126118.1	0.458376243	2.34E-29	postive
TAZ	AC126118.1	0.479626545	2.34E-32	postive
FBXW7	AC126118.1	0.426870179	2.79E-25	postive
HELLS	AC027514.2	0.502144233	8.97E-36	postive
ZNF419	AC027514.2	0.400083077	3.89E-22	postive
KLHL24	AC027514.2	0.47231459	2.66E-31	postive
TUBE1	AC027514.2	0.492875566	2.46E-34	postive
ALOX12	AC027514.2	0.467255733	1.39E-30	postive
IREB2	AC027514.2	0.458218288	2.46E-29	postive

GABPB1	AC027514.2	0.58699384	3.20E-51	postive
PIK3CA	AC027514.2	0.459688592	1.55E-29	postive
ATG7	AC027514.2	0.422351329	9.92E-25	postive
MAPK8	AC027514.2	0.461837521	7.85E-30	postive
LINC00472	AC027514.2	0.819971066	3.23E-132	postive
ATM	AC027514.2	0.735238291	9.42E-93	postive
FBXW7	AC027514.2	0.504891793	3.30E-36	postive
BRD4	AL135999.1	0.447275752	7.15E-28	postive
ZNF419	AL135999.1	0.606649925	1.74E-55	postive
VEGFA	AL135999.1	0.512443829	2.01E-37	postive
TUBE1	AL135999.1	0.524072785	2.35E-39	postive
SETD1B	AL135999.1	0.545180464	4.67E-43	postive
DRD4	AL135999.1	0.412442691	1.50E-23	postive
ALOX12	AL135999.1	0.666637342	1.51E-70	postive
PHKG2	AL135999.1	0.520788355	8.41E-39	postive
YY1AP1	AL135999.1	0.417653391	3.63E-24	postive
TAZ	AL135999.1	0.731706948	1.92E-91	postive
HSPB1	AC015922.2	-0.402389963	2.14E-22	negative
MAPK14	AC015922.2	0.409386308	3.39E-23	postive
PIK3CA	AC015922.2	0.404027092	1.40E-22	postive
BECN1	AC015922.2	0.480834689	1.56E-32	postive
TAZ	AC015922.2	-0.420390895	1.71E-24	negative
HELLS	MCCC1-AS1	0.405596102	9.25E-23	postive
ZNF419	MCCC1-AS1	0.532031529	1.01E-40	postive
TUBE1	MCCC1-AS1	0.595742275	4.40E-53	postive
SETD1B	MCCC1-AS1	0.415465052	6.60E-24	postive
ALOX12	MCCC1-AS1	0.633318522	9.02E-62	postive
GABPB1	MCCC1-AS1	0.421428805	1.28E-24	postive
PHKG2	MCCC1-AS1	0.437928176	1.16E-26	postive
LINC00472	MCCC1-AS1	0.469661003	6.35E-31	postive
ATM	MCCC1-AS1	0.478300118	3.66E-32	postive
TAZ	MCCC1-AS1	0.503842475	4.84E-36	postive
FBXW7	MCCC1-AS1	0.406113931	8.07E-23	postive
ISCU	AC116351.1	0.43751539	1.31E-26	postive
CS	AC116351.1	0.512584179	1.91E-37	postive
ATG4D	AC116351.1	0.403179118	1.74E-22	postive
GABARAPL1	AC116351.1	0.502401694	8.17E-36	postive
LPIN1	AC116351.1	0.458907912	1.98E-29	postive
HELLS	AC005899.6	0.49113346	4.52E-34	postive
ZNF419	AC005899.6	0.499175103	2.62E-35	postive
TUBE1	AC005899.6	0.542672295	1.33E-42	postive
ALOX12	AC005899.6	0.652474726	1.11E-66	postive
GABPB1	AC005899.6	0.410791258	2.33E-23	postive
ATM	AC005899.6	0.457407552	3.17E-29	postive
TAZ	AC005899.6	0.492635714	2.67E-34	postive
FBXW7	AC005899.6	0.47887029	3.02E-32	postive
HELLS	AP002336.2	0.464983685	2.88E-30	postive
KLHL24	AP002336.2	0.40112424	2.97E-22	postive
GABPB1	AP002336.2	0.474626022	1.24E-31	postive
LINC00472	AP002336.2	0.757402548	1.76E-101	postive
ATM	AP002336.2	0.453601246	1.03E-28	postive
TUBE1	AL023653.1	0.410093536	2.81E-23	postive
ALOX12	AL023653.1	0.422590502	9.28E-25	postive
PHKG2	AL023653.1	0.46938697	6.94E-31	postive
IFNG	AL023653.1	0.415997418	5.71E-24	postive
TAZ	AL023653.1	0.513072487	1.59E-37	postive
FBXW7	AL023653.1	0.466724748	1.64E-30	postive
HELLS	PCBP1-AS1	0.429612974	1.28E-25	postive

TUBE1	PCBP1-AS1	0.564950865	9.00E-47	postive
ALOX12	PCBP1-AS1	0.705084103	3.35E-82	postive
GABPB1	PCBP1-AS1	0.408955377	3.80E-23	postive
ATM	PCBP1-AS1	0.589534477	9.34E-52	postive
HELLS	AL022067.1	0.534634638	3.56E-41	postive
TUBE1	AL022067.1	0.454248842	8.47E-29	postive
GABPB1	AL022067.1	0.559042361	1.23E-45	postive
ATG7	AL022067.1	0.442077866	3.40E-27	postive
LINC00472	AL022067.1	0.656785509	7.77E-68	postive
ATM	AL022067.1	0.693187031	2.13E-78	postive
FBXW7	AL022067.1	0.564361444	1.17E-46	postive
RIPK1	LINC01128	0.426243174	3.33E-25	postive
ZEB1	LINC01128	0.564796289	9.64E-47	postive
MAPK8	LINC01128	0.415425573	6.67E-24	postive
EPAS1	LINC01128	0.55891579	1.30E-45	postive
NF2	ERVH-1	0.401520385	2.68E-22	postive
ZEB1	AC103746.1	0.402306391	2.19E-22	postive
MAPK8	AC103746.1	0.40339949	1.65E-22	postive
HELLS	AC122129.1	0.474634838	1.24E-31	postive
ZNF419	AC122129.1	0.470764083	4.43E-31	postive
TUBE1	AC122129.1	0.491881718	3.48E-34	postive
ALOX12	AC122129.1	0.671597899	5.95E-72	postive
GABPB1	AC122129.1	0.430752264	9.26E-26	postive
DUOX1	AC122129.1	0.411344829	2.01E-23	postive
MAPK8	AC122129.1	0.430968686	8.70E-26	postive
ATM	AC122129.1	0.571040705	5.73E-48	postive
FBXW7	AC122129.1	0.458906532	1.98E-29	postive
ATM	AL137847.1	0.44514827	1.36E-27	postive
HSPB1	Z97653.1	0.406088264	8.13E-23	postive
HRAS	Z97653.1	0.627917886	1.89E-60	postive
NCOA4	Z97653.1	-0.420718481	1.56E-24	negative
PHKG2	Z97653.1	0.477421837	4.91E-32	postive
EGLN2	Z97653.1	0.676533495	2.24E-73	postive
TAZ	Z97653.1	0.540050238	3.92E-42	postive
HELLS	AC005005.3	0.442869226	2.69E-27	postive
ZNF419	AC005005.3	0.476605506	6.44E-32	postive
ALOX12	AC005005.3	0.459288001	1.76E-29	postive
GABPB1	AC005005.3	0.516105206	5.05E-38	postive
LINC00472	AC005005.3	0.58720267	2.89E-51	postive
ATM	AC005005.3	0.509196479	6.75E-37	postive
FBXW7	AC005005.3	0.431614529	7.23E-26	postive
BRD4	AL022328.2	0.429673612	1.26E-25	postive
ZNF419	AL022328.2	0.580018658	8.91E-50	postive
VEGFA	AL022328.2	0.580183023	8.24E-50	postive
TUBE1	AL022328.2	0.55604981	4.54E-45	postive
SETD1B	AL022328.2	0.538379973	7.78E-42	postive
DRD4	AL022328.2	0.482681506	8.33E-33	postive
ALOX12	AL022328.2	0.680150664	1.94E-74	postive
PHKG2	AL022328.2	0.483466687	6.38E-33	postive
TAZ	AL022328.2	0.705879407	1.84E-82	postive
ISCU	AL161630.1	0.473475749	1.82E-31	postive
CS	AL161630.1	0.407463229	5.65E-23	postive
ATG4D	AL161630.1	0.511150647	3.26E-37	postive
GABARAPL1	AL161630.1	0.558343836	1.67E-45	postive
LPIN1	AL161630.1	0.401820029	2.48E-22	postive
HELLS	EHD4-AS1	0.460198035	1.32E-29	postive
TUBE1	EHD4-AS1	0.40848725	4.31E-23	postive
GABPB1	EHD4-AS1	0.512095165	2.29E-37	postive

LINC00472	EHD4-AS1	0.465776636	2.23E-30	postive
ATM	EHD4-AS1	0.678708738	5.16E-74	postive
FBXW7	EHD4-AS1	0.450943476	2.34E-28	postive
HRAS	AC004449.1	0.413401506	1.15E-23	postive
PHKG2	AC004449.1	0.460514652	1.19E-29	postive
TAZ	AC004449.1	0.56244842	2.74E-46	postive
NFS1	LINC01197	-0.414757322	8.00E-24	negative
IL33	LINC01197	0.652556681	1.06E-66	postive
HIC1	LINC01197	0.55050808	4.92E-44	postive
GABPB1	LINC01197	0.423506002	7.19E-25	postive
ZEB1	LINC01197	0.591633439	3.35E-52	postive
EPAS1	LINC01197	0.433278246	4.48E-26	postive
BRD4	AP001107.4	0.457900056	2.72E-29	postive
ZNF419	AP001107.4	0.577328682	3.14E-49	postive
VEGFA	AP001107.4	0.492827164	2.50E-34	postive
TUBE1	AP001107.4	0.55135808	3.43E-44	postive
SETD1B	AP001107.4	0.524716832	1.83E-39	postive
ALOX12	AP001107.4	0.578865396	1.53E-49	postive
TAZ	AP001107.4	0.55373997	1.23E-44	postive
HELLS	AC253536.3	0.458437632	2.30E-29	postive
MTOR	AC253536.3	0.431056529	8.49E-26	postive
KLHL24	AC253536.3	0.47621669	7.33E-32	postive
TUBE1	AC253536.3	0.49379799	1.77E-34	postive
MAP3K5	AC253536.3	0.404416922	1.26E-22	postive
ALOX12	AC253536.3	0.495022125	1.15E-34	postive
IREB2	AC253536.3	0.533533295	5.55E-41	postive
GABPB1	AC253536.3	0.546592249	2.58E-43	postive
PIK3CA	AC253536.3	0.482149312	9.99E-33	postive
MAPK8	AC253536.3	0.533800436	4.99E-41	postive
LINC00472	AC253536.3	0.793250495	8.64E-118	postive
PRKAA2	AC253536.3	0.42147457	1.27E-24	postive
PRKAA1	AC253536.3	0.414252591	9.17E-24	postive
ATM	AC253536.3	0.770804431	3.15E-107	postive
FBXW7	AC253536.3	0.489249057	8.72E-34	postive
TFAP2C	SLC25A30-A	0.511015835	3.43E-37	postive
HBA1	SLC25A30-A	0.453785028	9.78E-29	postive
DUOX1	SLC25A30-A	0.410767861	2.34E-23	postive
FH	BAALC-AS2	0.412523177	1.46E-23	postive
ISCU	BAALC-AS2	0.636132406	1.80E-62	postive
TMBIM4	BAALC-AS2	0.459598088	1.59E-29	postive
CHMP5	BAALC-AS2	0.40179852	2.50E-22	postive
GOT1	BAALC-AS2	0.438854549	8.82E-27	postive
ATG4D	BAALC-AS2	0.600156773	4.81E-54	postive
MAP1LC3A	BAALC-AS2	0.427581308	2.28E-25	postive
GABARAPL2	BAALC-AS2	0.610442162	2.42E-56	postive
GABARAPL1	BAALC-AS2	0.530278303	2.04E-40	postive
JDP2	LINC00920	0.551497279	3.23E-44	postive
ZFP69B	LINC00920	0.424389811	5.61E-25	postive
ZEB1	LINC00920	0.457069319	3.52E-29	postive
SLC2A12	AC100793.4	0.651235266	2.36E-66	postive
PLIN4	AC100793.4	0.432431048	5.72E-26	postive
TFAP2C	GK-IT1	0.578837194	1.55E-49	postive
HBA1	GK-IT1	0.530140799	2.16E-40	postive
DUOX1	GK-IT1	0.412345457	1.53E-23	postive
TAZ	CT66	0.427416174	2.39E-25	postive
HBA1	RPARP-AS1	0.532734177	7.66E-41	postive
HRAS	RPARP-AS1	0.564239301	1.24E-46	postive
EGLN2	RPARP-AS1	0.739455018	2.41E-94	postive

ISCU	AC104984.4	0.535219612	2.81E-41	postive
ACSL3	AC104984.4	0.417621449	3.66E-24	postive
KLHL24	AC104984.4	0.436428213	1.80E-26	postive
ATG4D	AC104984.4	0.467168499	1.42E-30	postive
GABARAPL1	AC104984.4	0.662362499	2.34E-69	postive
LINC00472	AC104984.4	0.417953685	3.34E-24	postive
LPIN1	AC104984.4	0.591661159	3.30E-52	postive
HELLS	RFX3-AS1	0.485432634	3.26E-33	postive
ZNF419	RFX3-AS1	0.423873469	6.49E-25	postive
TUBE1	RFX3-AS1	0.635273422	2.95E-62	postive
ALOX12	RFX3-AS1	0.636806957	1.22E-62	postive
GABPB1	RFX3-AS1	0.48042989	1.79E-32	postive
MAPK8	RFX3-AS1	0.417380128	3.91E-24	postive
ATM	RFX3-AS1	0.697113335	1.24E-79	postive
FBXW7	RFX3-AS1	0.519801275	1.23E-38	postive
FANCD2	AC123768.1	0.426418831	3.17E-25	postive
ALOX12	AC123768.1	0.430703413	9.39E-26	postive
TFAP2C	AC123768.1	0.521775875	5.75E-39	postive
HBA1	AC123768.1	0.517709457	2.74E-38	postive
DUOX1	AC123768.1	0.416489155	4.99E-24	postive
CA9	LINC02298	0.440170687	5.98E-27	postive
HRAS	LINC02298	0.460508572	1.20E-29	postive
TAZ	LINC02298	0.480605703	1.68E-32	postive
ALOX12	PXN-AS1	0.483883602	5.53E-33	postive
PHKG2	PXN-AS1	0.515727036	5.83E-38	postive
TAZ	PXN-AS1	0.628401661	1.45E-60	postive
HELLS	FTX	0.514376328	9.72E-38	postive
KLHL24	FTX	0.464425871	3.44E-30	postive
TUBE1	FTX	0.437193292	1.44E-26	postive
IREB2	FTX	0.439396975	7.52E-27	postive
GABPB1	FTX	0.580654665	6.60E-50	postive
PIK3CA	FTX	0.451283438	2.11E-28	postive
ATG7	FTX	0.458006033	2.63E-29	postive
MAPK8	FTX	0.434898829	2.80E-26	postive
LINC00472	FTX	0.825896867	9.60E-136	postive
ATM	FTX	0.709177167	1.49E-83	postive
FBXW7	FTX	0.470319628	5.12E-31	postive
HELLS	LINC02656	0.481211235	1.37E-32	postive
ZNF419	LINC02656	0.420707596	1.57E-24	postive
VEGFA	LINC02656	0.418121648	3.19E-24	postive
TUBE1	LINC02656	0.556851661	3.21E-45	postive
ALOX12	LINC02656	0.586835149	3.46E-51	postive
GABPB1	LINC02656	0.454294312	8.35E-29	postive
ATM	LINC02656	0.610602492	2.22E-56	postive
FBXW7	LINC02656	0.473890233	1.59E-31	postive
ZFP69B	AC108463.2	0.461707716	8.18E-30	postive
GABPB1	AC108463.2	0.44660314	8.77E-28	postive
ZEB1	AC108463.2	0.639258381	2.95E-63	postive
ATM	AC108463.2	0.447349913	7.00E-28	postive
FBXW7	AC108463.2	0.439345399	7.63E-27	postive
HELLS	DHDDS-AS1	0.589877679	7.90E-52	postive
ZNF419	DHDDS-AS1	0.447971412	5.80E-28	postive
TUBE1	DHDDS-AS1	0.545376922	4.30E-43	postive
ALOX12	DHDDS-AS1	0.595309598	5.46E-53	postive
GABPB1	DHDDS-AS1	0.561652014	3.90E-46	postive
LINC00472	DHDDS-AS1	0.526831747	7.99E-40	postive
ATM	DHDDS-AS1	0.756444988	4.40E-101	postive
FBXW7	DHDDS-AS1	0.528766318	3.72E-40	postive

HELLS	AC004596.1	0.436593835	1.71E-26	postive
MAFG	AC004596.1	0.409548741	3.25E-23	postive
ALOX12	AC004596.1	0.499366895	2.44E-35	postive
ATM	AC004596.1	0.484523531	4.45E-33	postive
TFAP2C	AL024508.1	0.55433634	9.54E-45	postive
HBA1	AL024508.1	0.539922081	4.13E-42	postive
DUOX1	AL024508.1	0.40817884	4.67E-23	postive
HELLS	SDCBP2-AS1	0.46171535	8.16E-30	postive
ZNF419	SDCBP2-AS1	0.40910699	3.65E-23	postive
KLHL24	SDCBP2-AS1	0.501893028	9.83E-36	postive
TUBE1	SDCBP2-AS1	0.506623453	1.75E-36	postive
ALOX12	SDCBP2-AS1	0.455495429	5.76E-29	postive
IREB2	SDCBP2-AS1	0.490728699	5.21E-34	postive
GABPB1	SDCBP2-AS1	0.578992569	1.44E-49	postive
PIK3CA	SDCBP2-AS1	0.480227424	1.91E-32	postive
KRAS	SDCBP2-AS1	0.40828598	4.54E-23	postive
MAPK8	SDCBP2-AS1	0.505274123	2.87E-36	postive
LINC00472	SDCBP2-AS1	0.808751849	6.98E-126	postive
LPIN1	SDCBP2-AS1	0.429942354	1.17E-25	postive
ATM	SDCBP2-AS1	0.688481061	6.04E-77	postive
FBXW7	SDCBP2-AS1	0.461749087	8.08E-30	postive
FH	AL135999.3	0.403073936	1.79E-22	postive
ISCU	AL135999.3	0.490378094	5.89E-34	postive
CS	AL135999.3	0.407812255	5.15E-23	postive
ATG4D	AL135999.3	0.442865726	2.69E-27	postive
GABARAPL1	AL135999.3	0.570495869	7.35E-48	postive
LPIN1	AL135999.3	0.448889514	4.39E-28	postive
NCOA4	SCGB1B2P	-0.424122385	6.05E-25	negative
PHKG2	SCGB1B2P	0.556258504	4.15E-45	postive
TAZ	SCGB1B2P	0.533187315	6.38E-41	postive
HELLS	AP003392.1	0.466864729	1.57E-30	postive
ZNF419	AP003392.1	0.557497546	2.42E-45	postive
VEGFA	AP003392.1	0.436761072	1.63E-26	postive
TUBE1	AP003392.1	0.62071533	1.00E-58	postive
SETD1B	AP003392.1	0.507494925	1.27E-36	postive
ALOX12	AP003392.1	0.739358761	2.63E-94	postive
GABPB1	AP003392.1	0.523767063	2.65E-39	postive
MAPK8	AP003392.1	0.494714102	1.28E-34	postive
LINC00472	AP003392.1	0.432494253	5.61E-26	postive
ATM	AP003392.1	0.66666894	1.48E-70	postive
YY1AP1	AP003392.1	0.426573278	3.04E-25	postive
FBXW7	AP003392.1	0.525645448	1.27E-39	postive
HELLS	FAM13A-AS1	0.454104275	8.86E-29	postive
ZNF419	FAM13A-AS1	0.530927714	1.58E-40	postive
VEGFA	FAM13A-AS1	0.444971546	1.43E-27	postive
TUBE1	FAM13A-AS1	0.655096697	2.21E-67	postive
ALOX12	FAM13A-AS1	0.69611884	2.56E-79	postive
GABPB1	FAM13A-AS1	0.491333766	4.22E-34	postive
LINC00472	FAM13A-AS1	0.439839151	6.60E-27	postive
ATM	FAM13A-AS1	0.609941864	3.14E-56	postive
TAZ	FAM13A-AS1	0.521837011	5.61E-39	postive
FBXW7	FAM13A-AS1	0.526111444	1.06E-39	postive
AKR1C3	AC016924.1	0.482329345	9.39E-33	postive
GPX4	SNHG11	0.432581593	5.47E-26	postive
RB1	SNHG11	-0.418254976	3.08E-24	negative
HSF1	SNHG11	0.484520226	4.45E-33	postive
SRC	SNHG11	0.429724704	1.24E-25	postive
OTUB1	SNHG11	0.514189399	1.04E-37	postive

PIK3CA	SNHG11	-0.416038485	5.65E-24	negative
NRAS	SNHG11	-0.437284778	1.40E-26	negative
NCOA4	SNHG11	-0.415269538	6.96E-24	negative
PHKG2	SNHG11	0.673976007	1.23E-72	postive
ULK1	SNHG11	0.409135445	3.62E-23	postive
ATG4D	SNHG11	0.446777997	8.32E-28	postive
MAPK1	SNHG11	-0.450293571	2.86E-28	negative
EPAS1	SNHG11	-0.406050914	8.21E-23	negative
TAZ	SNHG11	0.571854079	3.95E-48	postive
SIRT1	SNHG11	-0.439793432	6.69E-27	negative
HELLS	AC048344.4	0.577565969	2.81E-49	postive
TUBE1	AC048344.4	0.452081131	1.65E-28	postive
GABPB1	AC048344.4	0.546237129	3.00E-43	postive
LINC00472	AC048344.4	0.558544637	1.53E-45	postive
ATM	AC048344.4	0.602944193	1.17E-54	postive
FBXW7	AC048344.4	0.432905447	4.99E-26	postive
HELLS	LINC00621	0.491608343	3.83E-34	postive
GABPB1	LINC00621	0.413811615	1.03E-23	postive
LINC00472	LINC00621	0.400121219	3.85E-22	postive
ATM	LINC00621	0.596488322	3.03E-53	postive
ALB	LINC01485	0.843566052	4.15E-147	postive
GPX2	LINC01485	0.627888907	1.93E-60	postive
TF	LINC01485	0.567627459	2.70E-47	postive
TFR2	LINC01485	0.693564611	1.62E-78	postive
CDO1	LINC01485	0.78940368	6.88E-116	postive
HELLS	AC013731.1	0.464484221	3.38E-30	postive
ZNF419	AC013731.1	0.507468526	1.28E-36	postive
TUBE1	AC013731.1	0.494359591	1.46E-34	postive
ALOX12	AC013731.1	0.541741001	1.95E-42	postive
PHKG2	AC013731.1	0.574629675	1.10E-48	postive
LINC00472	AC013731.1	0.430437209	1.01E-25	postive
TAZ	AC013731.1	0.621030184	8.45E-59	postive
TFAP2C	AL357874.1	0.405395649	9.75E-23	postive
HBA1	AL357874.1	0.429962527	1.16E-25	postive
ALOX12B	AL357874.1	0.44393626	1.95E-27	postive
HELLS	Z97832.2	0.52934273	2.96E-40	postive
ZNF419	Z97832.2	0.613340275	5.25E-57	postive
VEGFA	Z97832.2	0.448128637	5.53E-28	postive
TUBE1	Z97832.2	0.675005486	6.22E-73	postive
SETD1B	Z97832.2	0.45459128	7.62E-29	postive
ALOX12	Z97832.2	0.756280958	5.14E-101	postive
GABPB1	Z97832.2	0.538117707	8.66E-42	postive
LINC00472	Z97832.2	0.426392067	3.20E-25	postive
ATM	Z97832.2	0.617492824	5.72E-58	postive
TAZ	Z97832.2	0.493315639	2.10E-34	postive
FBXW7	Z97832.2	0.501394362	1.18E-35	postive
ATF4	SNHG30	0.439839984	6.60E-27	postive
OTUB1	SNHG30	0.408741533	4.03E-23	postive
HRAS	SNHG30	0.417370515	3.92E-24	postive
SLC38A1	SNHG30	-0.407975895	4.93E-23	negative
ANO6	SNHG30	-0.428917219	1.56E-25	negative
TAZ	SNHG30	0.461298853	9.31E-30	postive
VEGFA	HPN-AS1	0.460401425	1.24E-29	postive
TUBE1	HPN-AS1	0.523083501	3.46E-39	postive
ALOX12	HPN-AS1	0.645111868	9.42E-65	postive
TAZ	HPN-AS1	0.471478586	3.51E-31	postive
ALOX12	LINC00235	0.479754231	2.24E-32	postive
GABPB1	LINC00235	0.409422639	3.36E-23	postive

ATM	LINC00235	0.507131584	1.45E-36	postive
HELLS	AP003419.2	0.475470415	9.40E-32	postive
TUBE1	AP003419.2	0.450714883	2.51E-28	postive
ALOX12	AP003419.2	0.468555308	9.09E-31	postive
GABPB1	AP003419.2	0.499859411	2.05E-35	postive
LINC00472	AP003419.2	0.64774945	1.95E-65	postive
ATM	AP003419.2	0.679736226	2.57E-74	postive
FBXW7	AP003419.2	0.452587047	1.41E-28	postive
HSPA5	AL157700.1	-0.402119545	2.30E-22	negative
ZNF419	AL157700.1	0.410423167	2.57E-23	postive
TUBE1	AL157700.1	0.492916017	2.42E-34	postive
TAZ	AL157700.1	0.418118591	3.20E-24	postive
FANCD2	SNHG4	0.560461455	6.60E-46	postive
HELLS	SNHG4	0.525242938	1.49E-39	postive
HELLS	AC015987.1	0.436582182	1.72E-26	postive
LINC00472	AC015987.1	0.542681356	1.32E-42	postive
ATM	AC015987.1	0.48216728	9.92E-33	postive
ISCU	AC243964.3	0.580153108	8.36E-50	postive
ATG4D	AC243964.3	0.505904589	2.28E-36	postive
GABARAPL1	AC243964.3	0.5814664	4.50E-50	postive
LPIN1	AC243964.3	0.494469179	1.40E-34	postive
HELLS	AC114763.1	0.540451894	3.32E-42	postive
KLHL24	AC114763.1	0.430406656	1.02E-25	postive
TUBE1	AC114763.1	0.412163566	1.61E-23	postive
ALOX12	AC114763.1	0.411224803	2.07E-23	postive
IREB2	AC114763.1	0.420690522	1.57E-24	postive
GABPB1	AC114763.1	0.547931195	1.47E-43	postive
PIK3CA	AC114763.1	0.418071882	3.24E-24	postive
ATG7	AC114763.1	0.446875653	8.07E-28	postive
LINC00472	AC114763.1	0.774189684	9.64E-109	postive
ATM	AC114763.1	0.710987173	3.69E-84	postive
FBXW7	AC114763.1	0.450469401	2.71E-28	postive
HRAS	LINC02716	0.404779633	1.15E-22	postive
HELLS	AL157813.1	0.423273531	7.67E-25	postive
TUBE1	AL157813.1	0.46825916	1.00E-30	postive
ALOX12	AL157813.1	0.407683064	5.33E-23	postive
GABPB1	AL157813.1	0.4865245	2.24E-33	postive
LINC00472	AL157813.1	0.515163294	7.22E-38	postive
ATM	AL157813.1	0.574669491	1.08E-48	postive
HSPB1	AL122035.1	-0.410071701	2.82E-23	negative
OXSR1	AL122035.1	0.439918115	6.45E-27	postive
EIF2S1	AL122035.1	0.415577613	6.40E-24	postive
IREB2	AL122035.1	0.471732576	3.23E-31	postive
ZEB1	AL122035.1	0.443549399	2.19E-27	postive
MAPK8	AL122035.1	0.512153514	2.24E-37	postive
ATM	AL122035.1	0.463002223	5.42E-30	postive
SIRT1	AL122035.1	0.492012524	3.32E-34	postive
LINC00472	AC008035.1	0.411260527	2.05E-23	postive
FANCD2	AC023825.2	0.410404327	2.58E-23	postive
HELLS	AC023825.2	0.591854515	3.01E-52	postive
TUBE1	AC023825.2	0.434633692	3.03E-26	postive
ALOX12	AC023825.2	0.505264923	2.88E-36	postive
GABPB1	AC023825.2	0.512387449	2.05E-37	postive
LINC00472	AC023825.2	0.52642781	9.37E-40	postive
ATM	AC023825.2	0.591498774	3.58E-52	postive
FBXW7	AC023825.2	0.511813277	2.55E-37	postive
HSF1	AC004812.2	0.400481615	3.51E-22	postive
ZNF419	AC004812.2	0.442478749	3.02E-27	postive

PHKG2	AC004812.2	0.454554702	7.71E-29	postive
ATG4D	AC004812.2	0.404257825	1.31E-22	postive
TAZ	AC004812.2	0.470989325	4.11E-31	postive
ZNF419	AL122035.2	0.516273574	4.74E-38	postive
VEGFA	AL122035.2	0.487144879	1.81E-33	postive
TUBE1	AL122035.2	0.55943074	1.04E-45	postive
ALOX12	AL122035.2	0.597104878	2.23E-53	postive
GABPB1	AL122035.2	0.46220915	6.98E-30	postive
MAPK8	AL122035.2	0.413281339	1.19E-23	postive
ATM	AL122035.2	0.507911131	1.09E-36	postive
TAZ	AL122035.2	0.400423386	3.56E-22	postive
FBXW7	AL122035.2	0.418010958	3.29E-24	postive
ZNF419	AC244197.2	0.415219042	7.06E-24	postive
TUBE1	AC244197.2	0.447357015	6.98E-28	postive
ALOX12	AC244197.2	0.500018358	1.93E-35	postive
PHKG2	AC244197.2	0.449125649	4.08E-28	postive
TAZ	AC244197.2	0.532888415	7.20E-41	postive
ALOX12	AC093249.2	0.444178571	1.82E-27	postive
KLHL24	CBR3-AS1	0.524917409	1.69E-39	postive
GABARAPL1	CBR3-AS1	0.407234452	6.00E-23	postive
LINC00472	CBR3-AS1	0.692014549	4.93E-78	postive
LPIN1	CBR3-AS1	0.554357258	9.45E-45	postive
ZNF419	AL121583.1	0.458683312	2.13E-29	postive
TUBE1	AL121583.1	0.484378355	4.67E-33	postive
GABPB1	AL121583.1	0.422138899	1.05E-24	postive
PHKG2	AL121583.1	0.455866061	5.13E-29	postive
LINC00472	AL121583.1	0.598877589	9.17E-54	postive
TAZ	AL121583.1	0.472612735	2.42E-31	postive
HELLS	AL121839.2	0.400006804	3.97E-22	postive
GABPB1	AL121839.2	0.456008619	4.91E-29	postive
LINC00472	AL121839.2	0.471952348	3.00E-31	postive
ATM	AL121839.2	0.42000453	1.90E-24	postive
HRAS	BTF3-DT	0.480080525	2.01E-32	postive
PHKG2	BTF3-DT	0.449583318	3.55E-28	postive
EGLN2	BTF3-DT	0.40305634	1.80E-22	postive
TAZ	BTF3-DT	0.489085463	9.23E-34	postive
ZFP36	AC011611.3	0.468326201	9.79E-31	postive
KLHL24	L3MBTL4-AS	0.458029364	2.61E-29	postive
GABPB1	L3MBTL4-AS	0.446269144	9.70E-28	postive
PIK3CA	L3MBTL4-AS	0.401904978	2.43E-22	postive
ATG7	L3MBTL4-AS	0.511194501	3.21E-37	postive
LINC00472	L3MBTL4-AS	0.75867441	5.22E-102	postive
TGFBR1	L3MBTL4-AS	0.464039866	3.89E-30	postive
ATM	L3MBTL4-AS	0.540463299	3.31E-42	postive
SLC3A2	AL118558.3	0.466890528	1.56E-30	postive
ATG4D	AL118558.3	0.441255153	4.34E-27	postive
ATM	AC046134.2	0.408826978	3.93E-23	postive
LAMP2	ITGB1-DT	0.467619159	1.23E-30	postive
KLHL24	AC009032.1	0.584625683	9.99E-51	postive
IREB2	AC009032.1	0.404803542	1.14E-22	postive
GABPB1	AC009032.1	0.429673186	1.26E-25	postive
PIK3CA	AC009032.1	0.480672581	1.65E-32	postive
KRAS	AC009032.1	0.420003272	1.90E-24	postive
LINC00472	AC009032.1	0.955181988	4.52E-286	postive
ATM	AC009032.1	0.402582432	2.04E-22	postive
LINC00472	AL021578.1	0.638975526	3.48E-63	postive
HSPB1	HYI-AS1	0.431242196	8.05E-26	postive
HBA1	HYI-AS1	0.664507932	5.95E-70	postive

HRAS	HYI-AS1	0.711440536	2.60E-84	postive
EGLN2	HYI-AS1	0.868821589	4.57E-166	postive
ZNF419	ADIRF-AS1	0.516460691	4.41E-38	postive
TUBE1	ADIRF-AS1	0.414510125	8.55E-24	postive
ALOX12	ADIRF-AS1	0.499767704	2.12E-35	postive
PHKG2	ADIRF-AS1	0.439055444	8.31E-27	postive
TAZ	ADIRF-AS1	0.548045947	1.40E-43	postive
HRAS	LAMA5-AS1	0.513556228	1.32E-37	postive
EGLN2	LAMA5-AS1	0.510538907	4.10E-37	postive
PTGS2	AC132872.3	0.57386057	1.57E-48	postive
VEGFA	AC132872.3	0.485069668	3.69E-33	postive
IL6	AC132872.3	0.55648405	3.76E-45	postive
SLC2A3	AC132872.3	0.525207144	1.51E-39	postive
TAZ	AC132872.3	0.480402838	1.80E-32	postive
HELLS	AL731566.2	0.540193122	3.70E-42	postive
ZNF419	AL731566.2	0.487949899	1.37E-33	postive
TUBE1	AL731566.2	0.583823482	1.47E-50	postive
ALOX12	AL731566.2	0.612894301	6.64E-57	postive
GABPB1	AL731566.2	0.607058494	1.41E-55	postive
MAPK8	AL731566.2	0.493883167	1.72E-34	postive
LINC00472	AL731566.2	0.64969926	6.01E-66	postive
ATM	AL731566.2	0.773525681	1.92E-108	postive
FBXW7	AL731566.2	0.53345339	5.73E-41	postive
HELLS	AP001469.2	0.483720581	5.85E-33	postive
ZNF419	AP001469.2	0.487775068	1.45E-33	postive
TUBE1	AP001469.2	0.563938972	1.41E-46	postive
SETD1B	AP001469.2	0.413740819	1.05E-23	postive
ALOX12	AP001469.2	0.66066454	6.85E-69	postive
GABPB1	AP001469.2	0.502354295	8.31E-36	postive
LINC00472	AP001469.2	0.44653623	8.95E-28	postive
ATM	AP001469.2	0.674444615	9.04E-73	postive
FBXW7	AP001469.2	0.484104819	5.13E-33	postive
TFAP2C	AC010525.1	0.585656071	6.10E-51	postive
HBA1	AC010525.1	0.529859829	2.41E-40	postive
DUOX1	AC010525.1	0.409422583	3.36E-23	postive
VEGFA	FOXO6-AS1	0.429372048	1.37E-25	postive
TAZ	FOXO6-AS1	0.549357022	8.03E-44	postive
HELLS	AC008937.3	0.414193686	9.32E-24	postive
KLHL24	AC008937.3	0.434960744	2.75E-26	postive
TUBE1	AC008937.3	0.448599483	4.79E-28	postive
IREB2	AC008937.3	0.437461808	1.33E-26	postive
GABPB1	AC008937.3	0.536930208	1.40E-41	postive
PIK3CA	AC008937.3	0.443522329	2.21E-27	postive
MAPK8	AC008937.3	0.503802221	4.91E-36	postive
LINC00472	AC008937.3	0.747492681	1.83E-97	postive
PRKAA2	AC008937.3	0.408556809	4.23E-23	postive
PRKAA1	AC008937.3	0.466234796	1.93E-30	postive
ATM	AC008937.3	0.702586512	2.18E-81	postive
FBXW7	AC008937.3	0.465045756	2.82E-30	postive
VEGFA	AC009084.1	0.566663794	4.17E-47	postive
TUBE1	AC009084.1	0.470094669	5.51E-31	postive
ALOX12	AC009084.1	0.561994171	3.35E-46	postive
ATM	AC009084.1	0.40203942	2.34E-22	postive
RGS4	AL009178.2	0.630061291	5.70E-61	postive
SLC3A2	LINC01230	0.563334375	1.85E-46	postive
FH	LINC01230	0.469073701	7.68E-31	postive
ISCU	LINC01230	0.692834257	2.74E-78	postive
DDIT3	LINC01230	0.603426375	9.13E-55	postive

HERPUD1	LINC01230	0.436839098	1.59E-26	postive
SLC2A12	LINC01230	0.430979804	8.67E-26	postive
CS	LINC01230	0.490680622	5.30E-34	postive
GOT1	LINC01230	0.475991444	7.90E-32	postive
ATG4D	LINC01230	0.555794621	5.08E-45	postive
MAP1LC3A	LINC01230	0.43517499	2.59E-26	postive
GABARAPL2	LINC01230	0.430306534	1.05E-25	postive
GABARAPL1	LINC01230	0.813652937	1.35E-128	postive
WIPI2	LINC01230	0.41620051	5.40E-24	postive
LPIN1	LINC01230	0.564768221	9.76E-47	postive
HELLS	AL390208.1	0.559896861	8.46E-46	postive
ZNF419	AL390208.1	0.423131943	7.98E-25	postive
TUBE1	AL390208.1	0.5963787	3.20E-53	postive
ALOX12	AL390208.1	0.539332525	5.26E-42	postive
GABPB1	AL390208.1	0.56715141	3.35E-47	postive
MAPK8	AL390208.1	0.413943525	9.97E-24	postive
LINC00472	AL390208.1	0.468823979	8.33E-31	postive
ATM	AL390208.1	0.709669887	1.02E-83	postive
FBXW7	AL390208.1	0.520470766	9.51E-39	postive
KLHL24	AC036214.2	0.638476252	4.65E-63	postive
IREB2	AC036214.2	0.443757934	2.06E-27	postive
GABPB1	AC036214.2	0.424515159	5.42E-25	postive
PIK3CA	AC036214.2	0.492211655	3.10E-34	postive
KRAS	AC036214.2	0.465371866	2.54E-30	postive
LINC00472	AC036214.2	0.858650585	5.45E-158	postive
PRKAA1	AC036214.2	0.423872577	6.49E-25	postive
TGFBR1	AC036214.2	0.410205588	2.72E-23	postive
LPIN1	AC036214.2	0.440062345	6.18E-27	postive
ATM	AC036214.2	0.526546997	8.94E-40	postive
TFAP2C	AL360157.1	0.587519356	2.48E-51	postive
HBA1	AL360157.1	0.531070663	1.49E-40	postive
ZNF419	AP001094.2	0.451499773	1.98E-28	postive
VEGFA	AP001094.2	0.406910653	6.54E-23	postive
TUBE1	AP001094.2	0.594132084	9.78E-53	postive
SETD1B	AP001094.2	0.405488719	9.51E-23	postive
ALOX12	AP001094.2	0.659149097	1.78E-68	postive
GABPB1	AP001094.2	0.438991688	8.47E-27	postive
ATM	AP001094.2	0.614508828	2.82E-57	postive
FBXW7	AP001094.2	0.44989751	3.23E-28	postive
HELLS	AL139407.1	0.541532625	2.13E-42	postive
ZNF419	AL139407.1	0.410482792	2.53E-23	postive
KLHL24	AL139407.1	0.429530347	1.31E-25	postive
TUBE1	AL139407.1	0.526410648	9.43E-40	postive
ALOX12	AL139407.1	0.492717271	2.60E-34	postive
IREB2	AL139407.1	0.457169578	3.42E-29	postive
GABPB1	AL139407.1	0.598267365	1.25E-53	postive
PIK3CA	AL139407.1	0.442265067	3.22E-27	postive
ATG7	AL139407.1	0.441073726	4.58E-27	postive
MAPK8	AL139407.1	0.479196775	2.71E-32	postive
LINC00472	AL139407.1	0.713177355	6.73E-85	postive
ATM	AL139407.1	0.762002593	2.07E-103	postive
FBXW7	AL139407.1	0.531966038	1.04E-40	postive
HBA1	LINC01983	0.799117279	9.06E-121	postive
HRAS	LINC01983	0.592077483	2.69E-52	postive
MAP1LC3A	LINC01983	0.41373381	1.06E-23	postive
EGLN2	LINC01983	0.8343566	5.11E-141	postive
HSPA5	LINC01089	-0.411272	2.05E-23	negative
BRD4	LINC01089	0.427337744	2.45E-25	postive

ZNF419	LINC01089	0.57087465	6.19E-48	postive
TUBE1	LINC01089	0.461021019	1.02E-29	postive
SETD1B	LINC01089	0.428964423	1.54E-25	postive
DRD4	LINC01089	0.411902053	1.73E-23	postive
ALOX12	LINC01089	0.526788057	8.13E-40	postive
NCOA4	LINC01089	-0.42177471	1.16E-24	negative
PHKG2	LINC01089	0.628098714	1.71E-60	postive
ULK1	LINC01089	0.484814977	4.02E-33	postive
TAZ	LINC01089	0.744157302	3.72E-96	postive
TFAP2C	USP12-AS2	0.573316151	2.02E-48	postive
HBA1	USP12-AS2	0.523117855	3.41E-39	postive
SLC7A5	HOXB-AS4	0.4513646	2.06E-28	postive
NOX1	AL354760.1	0.41240813	1.51E-23	postive
NCOA4	AL354760.1	-0.493544391	1.94E-34	negative
PHKG2	AL354760.1	0.696975687	1.37E-79	postive
BECN1	AL354760.1	-0.452365505	1.51E-28	negative
TAZ	AL354760.1	0.732764201	7.83E-92	postive
HELLS	AC007365.1	0.404149662	1.35E-22	postive
ALOX12	AC007365.1	0.439667959	6.94E-27	postive
IREB2	AC007365.1	0.438221086	1.06E-26	postive
SP1	AC007365.1	0.418142647	3.17E-24	postive
MAPK8	AC007365.1	0.447557648	6.57E-28	postive
PRKAA2	AC007365.1	0.426408991	3.18E-25	postive
ATM	AC007365.1	0.665868168	2.48E-70	postive
SIRT1	AC007365.1	0.400562855	3.44E-22	postive
HELLS	AC004034.1	0.442305041	3.18E-27	postive
BRD4	AC004034.1	0.440344986	5.68E-27	postive
ZNF419	AC004034.1	0.454337174	8.24E-29	postive
TUBE1	AC004034.1	0.510656306	3.92E-37	postive
SETD1B	AC004034.1	0.42929559	1.40E-25	postive
ALOX12	AC004034.1	0.575162647	8.61E-49	postive
PHKG2	AC004034.1	0.516631906	4.13E-38	postive
TAZ	AC004034.1	0.598879577	9.16E-54	postive
MAPK8	AL078644.1	0.409287285	3.48E-23	postive
ATM	AL078644.1	0.415787537	6.04E-24	postive
YY1AP1	AL078644.1	0.416642626	4.79E-24	postive
KLHL24	AC078850.2	0.435657871	2.25E-26	postive
GABARAPL1	AC078850.2	0.430881237	8.92E-26	postive
LPIN1	AC078850.2	0.432153891	6.19E-26	postive
HELLS	AC114956.2	0.46751742	1.27E-30	postive
MTOR	AC114956.2	0.415918561	5.83E-24	postive
ZNF419	AC114956.2	0.441271441	4.32E-27	postive
TUBE1	AC114956.2	0.483734128	5.82E-33	postive
ALOX12	AC114956.2	0.416869518	4.50E-24	postive
IREB2	AC114956.2	0.4013271	2.82E-22	postive
GABPB1	AC114956.2	0.515835551	5.59E-38	postive
ATG7	AC114956.2	0.425279553	4.37E-25	postive
MAPK8	AC114956.2	0.447507761	6.67E-28	postive
ATM	AC114956.2	0.675228295	5.36E-73	postive
FBXW7	AC114956.2	0.509817298	5.36E-37	postive
GPX4	AC098484.1	-0.408143915	4.72E-23	negative
RB1	AC098484.1	0.480895692	1.53E-32	postive
HSPB1	AC098484.1	-0.50101398	1.35E-35	negative
GCLC	AC098484.1	0.422125169	1.06E-24	postive
STAT3	AC098484.1	0.413672681	1.07E-23	postive
MTOR	AC098484.1	0.511603549	2.75E-37	postive
OXSR1	AC098484.1	0.509542274	5.94E-37	postive
MAP3K5	AC098484.1	0.429450327	1.34E-25	postive

MAPK14	AC098484.1	0.522558281	4.24E-39	postive
IREB2	AC098484.1	0.684310356	1.11E-75	postive
SP1	AC098484.1	0.464807017	3.05E-30	postive
PIK3CA	AC098484.1	0.544877701	5.30E-43	postive
NRAS	AC098484.1	0.558941687	1.29E-45	postive
KRAS	AC098484.1	0.458929094	1.97E-29	postive
SLC1A5	AC098484.1	-0.401291069	2.85E-22	negative
NCOA4	AC098484.1	0.558436052	1.61E-45	postive
PHKG2	AC098484.1	-0.44997007	3.16E-28	negative
BECN1	AC098484.1	0.405437177	9.64E-23	postive
ULK2	AC098484.1	0.408763771	4.00E-23	postive
MAPK1	AC098484.1	0.579923073	9.32E-50	postive
ZEB1	AC098484.1	0.469720319	6.23E-31	postive
MAPK8	AC098484.1	0.627751951	2.08E-60	postive
MAPK9	AC098484.1	0.425134374	4.56E-25	postive
PRKAA2	AC098484.1	0.70528057	2.89E-82	postive
PRKAA1	AC098484.1	0.553785679	1.21E-44	postive
ANO6	AC098484.1	0.419321665	2.30E-24	postive
TLR4	AC098484.1	0.542574899	1.38E-42	postive
ATM	AC098484.1	0.588624686	1.45E-51	postive
MTDH	AC098484.1	0.401114776	2.98E-22	postive
SIRT1	AC098484.1	0.590724448	5.23E-52	postive
SLC2A12	RHOXF1-AS1	0.646062826	5.34E-65	postive
PLIN4	RHOXF1-AS1	0.416273089	5.30E-24	postive
ACO1	RHOXF1-AS1	0.441147243	4.48E-27	postive
FANCD2	AL596094.1	0.410296604	2.66E-23	postive
JDP2	AL596094.1	0.501419084	1.17E-35	postive
KLHL24	SIAH2-AS1	0.648227235	1.46E-65	postive
IREB2	SIAH2-AS1	0.41831592	3.03E-24	postive
PIK3CA	SIAH2-AS1	0.485535292	3.14E-33	postive
KRAS	SIAH2-AS1	0.410856003	2.29E-23	postive
LINC00472	SIAH2-AS1	0.804535443	1.31E-123	postive
LPIN1	SIAH2-AS1	0.415031027	7.43E-24	postive
ATM	SIAH2-AS1	0.430635969	9.57E-26	postive
HELLS	AC107068.1	0.427212861	2.54E-25	postive
ZNF419	AC107068.1	0.476956704	5.73E-32	postive
KLHL24	AC107068.1	0.526685682	8.46E-40	postive
TUBE1	AC107068.1	0.477931599	4.14E-32	postive
IREB2	AC107068.1	0.468836611	8.30E-31	postive
GABPB1	AC107068.1	0.610357897	2.53E-56	postive
PIK3CA	AC107068.1	0.450058408	3.07E-28	postive
KRAS	AC107068.1	0.461758203	8.05E-30	postive
MAPK8	AC107068.1	0.536207245	1.88E-41	postive
LINC00472	AC107068.1	0.722935833	2.81E-88	postive
PRKAA2	AC107068.1	0.401130307	2.97E-22	postive
PRKAA1	AC107068.1	0.462730207	5.91E-30	postive
ATM	AC107068.1	0.608577278	6.40E-56	postive
FBXW7	AC107068.1	0.493015056	2.34E-34	postive
FANCD2	COX10-AS1	0.627582975	2.28E-60	postive
HELLS	COX10-AS1	0.624670757	1.15E-59	postive
ANGPTL7	COX10-AS1	0.438126981	1.09E-26	postive
ZNF419	COX10-AS1	0.530438422	1.92E-40	postive
ZFP69B	COX10-AS1	0.456905579	3.71E-29	postive
TUBE1	COX10-AS1	0.434016559	3.62E-26	postive
ALOX12	COX10-AS1	0.55013831	5.76E-44	postive
GABPB1	COX10-AS1	0.50058531	1.58E-35	postive
ULK2	COX10-AS1	0.413456337	1.14E-23	postive
ATM	COX10-AS1	0.482652883	8.42E-33	postive

FBXW7	COX10-AS1	0.469167674	7.45E-31	postive
HELLS	AC080162.1	0.565232078	7.93E-47	postive
ZNF419	AC080162.1	0.417659215	3.63E-24	postive
TUBE1	AC080162.1	0.520921545	7.99E-39	postive
ALOX12	AC080162.1	0.575710844	6.68E-49	postive
GABPB1	AC080162.1	0.562396389	2.81E-46	postive
MAPK8	AC080162.1	0.4595221	1.63E-29	postive
LINC00472	AC080162.1	0.59578501	4.31E-53	postive
ATM	AC080162.1	0.761118679	4.91E-103	postive
FBXW7	AC080162.1	0.515124385	7.32E-38	postive
NCOA4	AC093249.6	-0.436554902	1.73E-26	negative
PHKG2	AC093249.6	0.637089241	1.04E-62	postive
TAZ	AC093249.6	0.641843542	6.51E-64	postive
GABPB1	AL359076.1	0.404468293	1.24E-22	postive
LINC00472	AL359076.1	0.518784443	1.82E-38	postive
HRAS	BRWD1-AS2	0.465334383	2.57E-30	postive
EGLN2	BRWD1-AS2	0.437645939	1.26E-26	postive
HSF1	SNHG3	0.401703682	2.56E-22	postive
FANCD2	SNHG3	0.566807453	3.91E-47	postive
HELLS	SNHG3	0.530389264	1.95E-40	postive
STMN1	SNHG3	0.484529979	4.44E-33	postive
RRM2	SNHG3	0.497458311	4.84E-35	postive
AURKA	SNHG3	0.472219673	2.75E-31	postive
PHKG2	SNHG3	0.44756944	6.55E-28	postive
BID	SNHG3	0.406529727	7.23E-23	postive
CDKN2A	SNHG3	0.568664572	1.69E-47	postive
TAZ	SNHG3	0.518642515	1.92E-38	postive
KLHL24	AC025271.3	0.57890789	1.50E-49	postive
PIK3CA	AC025271.3	0.445633027	1.17E-27	postive
LINC00472	AC025271.3	0.925390201	2.04E-228	postive
NNMT	LINC00160	0.475898266	8.15E-32	postive
FANCD2	AL360091.1	0.409568269	3.23E-23	postive
HELLS	AL360091.1	0.42917413	1.45E-25	postive
ALOX12	AL360091.1	0.410986041	2.21E-23	postive
TFAP2C	AL360091.1	0.536321812	1.80E-41	postive
HBA1	AL360091.1	0.46635726	1.85E-30	postive
DUOX1	AL360091.1	0.437885947	1.17E-26	postive
ZNF419	AL353708.3	0.439371047	7.57E-27	postive
TUBE1	AL353708.3	0.446344108	9.48E-28	postive
GABPB1	AL353708.3	0.423592874	7.02E-25	postive
PHKG2	AL353708.3	0.500286545	1.76E-35	postive
LINC00472	AL353708.3	0.405756053	8.87E-23	postive
TAZ	AL353708.3	0.520259513	1.03E-38	postive
TUBE1	LINC01238	0.446199572	9.90E-28	postive
ALOX12	LINC01238	0.51341662	1.40E-37	postive
TAZ	LINC01238	0.480035392	2.04E-32	postive
FANCD2	C1orf220	0.447586038	6.51E-28	postive
HELLS	C1orf220	0.439523882	7.24E-27	postive
BRD4	C1orf220	0.407816986	5.15E-23	postive
ZNF419	C1orf220	0.586041507	5.07E-51	postive
TUBE1	C1orf220	0.532789075	7.49E-41	postive
SETD1B	C1orf220	0.456510775	4.20E-29	postive
ALOX12	C1orf220	0.654932087	2.45E-67	postive
GABPB1	C1orf220	0.431875751	6.71E-26	postive
LPIN1	C1orf220	0.419078129	2.46E-24	postive
ATM	C1orf220	0.465952253	2.11E-30	postive
TAZ	C1orf220	0.476356068	7.00E-32	postive
FBXW7	C1orf220	0.413689186	1.07E-23	postive

ALOX12	AL161669.1	0.547830263	1.53E-43	postive
KLHL24	AC083843.2	0.590551657	5.69E-52	postive
IREB2	AC083843.2	0.411707602	1.82E-23	postive
GABPB1	AC083843.2	0.429760665	1.23E-25	postive
PIK3CA	AC083843.2	0.44740548	6.88E-28	postive
KRAS	AC083843.2	0.429671626	1.26E-25	postive
LINC00472	AC083843.2	0.921668688	5.77E-223	postive
LPIN1	AC083843.2	0.453524831	1.06E-28	postive
TFAP2C	AL132639.2	0.545590032	3.93E-43	postive
HBA1	AL132639.2	0.453453392	1.08E-28	postive
ZFP36	AC087521.1	0.431480139	7.51E-26	postive
ATF3	AC087521.1	0.409601834	3.20E-23	postive
HIC1	AC087521.1	0.502393123	8.20E-36	postive
CHMP5	RNASEH1-AS	0.413788091	1.04E-23	postive
FANCD2	AC012186.2	0.41394037	9.98E-24	postive
ALOX12	AC012186.2	0.442404974	3.08E-27	postive
TFAP2C	AC012186.2	0.540140094	3.78E-42	postive
HBA1	AC012186.2	0.476243346	7.27E-32	postive
DUOX1	AC012186.2	0.434013867	3.62E-26	postive
TSC22D3	LINC00887	0.419166862	2.40E-24	postive
ALOX12	LINC00887	0.406213147	7.86E-23	postive
HILPDA	LINC00887	0.435217851	2.55E-26	postive
HELLS	AC093535.1	0.436159391	1.94E-26	postive
ZNF419	AC093535.1	0.415748998	6.11E-24	postive
KLHL24	AC093535.1	0.435648047	2.25E-26	postive
TUBE1	AC093535.1	0.474790247	1.18E-31	postive
ALOX12	AC093535.1	0.429279653	1.41E-25	postive
GABPB1	AC093535.1	0.545115884	4.79E-43	postive
LINC00472	AC093535.1	0.825801844	1.10E-135	postive
ATM	AC093535.1	0.618468353	3.39E-58	postive
FBXW7	AC093535.1	0.48335765	6.62E-33	postive
FANCD2	AL354892.3	0.42174396	1.17E-24	postive
HELLS	AL354892.3	0.458206294	2.47E-29	postive
ZNF419	AL354892.3	0.478062414	3.96E-32	postive
TUBE1	AL354892.3	0.498522518	3.31E-35	postive
ALOX12	AL354892.3	0.611118392	1.69E-56	postive
TFAP2C	AL354892.3	0.418851669	2.61E-24	postive
DUOX1	AL354892.3	0.415534658	6.48E-24	postive
FBXW7	AL354892.3	0.415734341	6.13E-24	postive
TUBE1	AC124319.2	0.44704222	7.68E-28	postive
ALOX12	AC124319.2	0.409476394	3.31E-23	postive
BRD4	ZNRD2-AS1	0.406565222	7.17E-23	postive
ZNF419	ZNRD2-AS1	0.512860488	1.72E-37	postive
VEGFA	ZNRD2-AS1	0.530684683	1.74E-40	postive
TUBE1	ZNRD2-AS1	0.506520763	1.81E-36	postive
SETD1B	ZNRD2-AS1	0.483464186	6.38E-33	postive
ALOX12	ZNRD2-AS1	0.656883754	7.31E-68	postive
TAZ	ZNRD2-AS1	0.543777868	8.38E-43	postive
ENPP2	AC009686.2	0.621274743	7.39E-59	postive
HIC1	AL138995.1	0.412951972	1.30E-23	postive
ZEB1	AL138995.1	0.426358346	3.23E-25	postive
BRD4	AC026471.4	0.455372523	5.98E-29	postive
ZNF419	AC026471.4	0.491935839	3.42E-34	postive
TUBE1	AC026471.4	0.411646893	1.85E-23	postive
DRD4	AC026471.4	0.43805128	1.12E-26	postive
ALOX12	AC026471.4	0.568246673	2.04E-47	postive
NCOA4	AC026471.4	-0.411810401	1.77E-23	negative
PHKG2	AC026471.4	0.509555964	5.91E-37	postive

TAZ	AC026471.4	0.564793587	9.65E-47	postive
HELLS	AP001363.1	0.529614044	2.66E-40	postive
TUBE1	AP001363.1	0.449071022	4.15E-28	postive
ALOX12	AP001363.1	0.498328262	3.55E-35	postive
GABPB1	AP001363.1	0.446373792	9.39E-28	postive
LINC00472	AP001363.1	0.415069381	7.35E-24	postive
ATM	AP001363.1	0.665795728	2.60E-70	postive
FBXW7	AP001363.1	0.473400102	1.86E-31	postive
TUBE1	AC090825.1	0.421660644	1.20E-24	postive
ALOX12	AC090825.1	0.480743718	1.61E-32	postive
MAPK8	AC090825.1	0.402636136	2.01E-22	postive
ATM	AC090825.1	0.539208225	5.54E-42	postive
YY1AP1	AC090825.1	0.427462772	2.36E-25	postive
HELLS	AL008723.2	0.521451856	6.51E-39	postive
TUBE1	AL008723.2	0.422878583	8.56E-25	postive
ALOX12	AL008723.2	0.45926618	1.77E-29	postive
GABPB1	AL008723.2	0.501240355	1.24E-35	postive
LINC00472	AL008723.2	0.584052047	1.31E-50	postive
ATM	AL008723.2	0.654749708	2.74E-67	postive
FBXW7	AL008723.2	0.429184915	1.45E-25	postive
HELLS	AL035071.2	0.465882177	2.16E-30	postive
KLHL24	AL035071.2	0.519525595	1.37E-38	postive
TUBE1	AL035071.2	0.420478315	1.67E-24	postive
IREB2	AL035071.2	0.44575983	1.13E-27	postive
GABPB1	AL035071.2	0.573086571	2.25E-48	postive
PIK3CA	AL035071.2	0.476533539	6.60E-32	postive
ATG7	AL035071.2	0.412894383	1.32E-23	postive
MAPK8	AL035071.2	0.436474187	1.77E-26	postive
LINC00472	AL035071.2	0.886797291	3.81E-182	postive
PRKAA1	AL035071.2	0.400947075	3.11E-22	postive
ATM	AL035071.2	0.677560499	1.12E-73	postive
FBXW7	AL035071.2	0.488717518	1.05E-33	postive
RB1	AP001372.2	0.540801155	2.88E-42	postive
HSD17B11	AP001372.2	0.457490434	3.09E-29	postive
IREB2	AP001372.2	0.408870006	3.89E-23	postive
SCP2	AP001372.2	0.535291234	2.73E-41	postive
NRAS	AP001372.2	0.423148987	7.94E-25	postive
ATG5	AP001372.2	0.409503632	3.29E-23	postive
NCOA4	AP001372.2	0.443852597	2.00E-27	postive
SNX4	AP001372.2	0.435150809	2.60E-26	postive
MAPK1	AP001372.2	0.469388578	6.94E-31	postive
MAPK8	AP001372.2	0.462626625	6.11E-30	postive
MAPK9	AP001372.2	0.420825272	1.52E-24	postive
PRKAA2	AP001372.2	0.530730392	1.71E-40	postive
PRKAA1	AP001372.2	0.501383115	1.18E-35	postive
SIRT1	AP001372.2	0.519571526	1.34E-38	postive
STEAP3	AC079209.1	0.514922067	7.91E-38	postive
SETD1B	AC245140.2	0.410694347	2.39E-23	postive
ALOX12	AC245140.2	0.47895854	2.93E-32	postive
TAZ	AC245140.2	0.448015424	5.72E-28	postive
KLHL24	RERG-AS1	0.576137221	5.48E-49	postive
IREB2	RERG-AS1	0.435664908	2.24E-26	postive
PIK3CA	RERG-AS1	0.447578	6.53E-28	postive
KRAS	RERG-AS1	0.401710807	2.55E-22	postive
LINC00472	RERG-AS1	0.85899005	3.00E-158	postive
ATM	RERG-AS1	0.459376136	1.71E-29	postive
MUC1	AC120498.1C	0.448029028	5.70E-28	postive
CD44	AC120498.1C	0.445527858	1.21E-27	postive

SOCS1	AC120498.1C	0.55121862	3.64E-44	postive
HELLS	LINC02649	0.545947467	3.38E-43	postive
ZNF419	LINC02649	0.426324948	3.26E-25	postive
TUBE1	LINC02649	0.515798608	5.67E-38	postive
ALOX12	LINC02649	0.592871014	1.82E-52	postive
GABPB1	LINC02649	0.515420225	6.55E-38	postive
LINC00472	LINC02649	0.431844747	6.77E-26	postive
ATM	LINC02649	0.705012514	3.54E-82	postive
FBXW7	LINC02649	0.491005267	4.73E-34	postive
BRD4	AC008735.2	0.444851906	1.48E-27	postive
ZNF419	AC008735.2	0.501370311	1.19E-35	postive
VEGFA	AC008735.2	0.497190179	5.33E-35	postive
TUBE1	AC008735.2	0.491579562	3.87E-34	postive
SETD1B	AC008735.2	0.519885116	1.19E-38	postive
DRD4	AC008735.2	0.425389551	4.24E-25	postive
ALOX12	AC008735.2	0.545019104	4.99E-43	postive
PHKG2	AC008735.2	0.536135254	1.94E-41	postive
TAZ	AC008735.2	0.676690688	2.01E-73	postive
ZNF419	AC060780.1	0.497614937	4.58E-35	postive
VEGFA	AC060780.1	0.528200104	4.66E-40	postive
TUBE1	AC060780.1	0.562674852	2.48E-46	postive
SETD1B	AC060780.1	0.548586406	1.11E-43	postive
ALOX12	AC060780.1	0.683550378	1.88E-75	postive
GABPB1	AC060780.1	0.402327767	2.18E-22	postive
MAPK8	AC060780.1	0.414998379	7.49E-24	postive
ATM	AC060780.1	0.504328775	4.05E-36	postive
YY1AP1	AC060780.1	0.469913212	5.85E-31	postive
SLC2A8	AC020978.4	0.463851416	4.14E-30	postive
ATG4D	AC020978.4	0.489063981	9.30E-34	postive
GABARAPL1	AC020978.4	0.469998548	5.69E-31	postive
LPIN1	AC020978.4	0.507571599	1.23E-36	postive
BRD4	AC090589.3	0.433150259	4.65E-26	postive
ZNF419	AC090589.3	0.599983671	5.25E-54	postive
VEGFA	AC090589.3	0.548938525	9.59E-44	postive
TUBE1	AC090589.3	0.673533966	1.66E-72	postive
SETD1B	AC090589.3	0.539972703	4.05E-42	postive
ALOX12	AC090589.3	0.737767532	1.05E-93	postive
GABPB1	AC090589.3	0.439150172	8.08E-27	postive
ATM	AC090589.3	0.530690205	1.73E-40	postive
YY1AP1	AC090589.3	0.414152349	9.42E-24	postive
TAZ	AC090589.3	0.555980599	4.68E-45	postive
FBXW7	AC090589.3	0.49008809	6.51E-34	postive
KLHL24	LINC01948	0.406009573	8.30E-23	postive
ATM	LINC01948	0.436434518	1.79E-26	postive
FANCD2	AL512770.1	0.459909555	1.45E-29	postive
BRD4	AL512770.1	0.451893655	1.75E-28	postive
ZNF419	AL512770.1	0.494129671	1.58E-34	postive
TUBE1	AL512770.1	0.46016754	1.33E-29	postive
DRD4	AL512770.1	0.40424425	1.32E-22	postive
ALOX12	AL512770.1	0.639742011	2.23E-63	postive
TAZ	AL512770.1	0.466774208	1.62E-30	postive
BRD4	AC116913.1	0.436161724	1.94E-26	postive
ZNF419	AC116913.1	0.563058483	2.09E-46	postive
TUBE1	AC116913.1	0.51054132	4.10E-37	postive
ALOX12	AC116913.1	0.499546542	2.29E-35	postive
GABPB1	AC116913.1	0.447840098	6.03E-28	postive
PHKG2	AC116913.1	0.524357505	2.11E-39	postive
EGLN2	AC116913.1	0.415442541	6.64E-24	postive

TAZ	AC116913.1	0.591008565	4.55E-52	postive
FBXW7	AC116913.1	0.42453788	5.39E-25	postive
ALOX15B	MMP2-AS1	0.451380447	2.05E-28	postive
MTDH	AP002761.3	-0.426414798	3.18E-25	negative
FANCD2	AL135818.1	0.436875495	1.58E-26	postive
PML	AL135818.1	0.409748696	3.08E-23	postive
GCH1	AL135818.1	0.522600914	4.17E-39	postive
IFNG	AL135818.1	0.662342293	2.37E-69	postive
MAPK8	LINC01060	0.420955603	1.46E-24	postive
ATM	LINC01060	0.453612777	1.03E-28	postive
NCF2	AL133371.2	0.425176531	4.50E-25	postive
CYBB	AL133371.2	0.607341051	1.22E-55	postive
ATG7	AL133371.2	0.477861854	4.23E-32	postive
TLR4	AL133371.2	0.517721877	2.73E-38	postive
ATM	AL133371.2	0.500711332	1.51E-35	postive
DRD4	DM1-AS	0.452250036	1.57E-28	postive
ALOX12	DM1-AS	0.451282523	2.11E-28	postive
NRAS	DM1-AS	-0.412763456	1.37E-23	negative
NCOA4	DM1-AS	-0.477019079	5.61E-32	negative
PHKG2	DM1-AS	0.632376812	1.54E-61	postive
TAZ	DM1-AS	0.658831185	2.17E-68	postive
MTDH	DM1-AS	-0.430216804	1.08E-25	negative
BACH1	DM1-AS	-0.417851349	3.44E-24	negative
ATP6V1G2	AC010997.4	0.441584296	3.94E-27	postive
WIPI1	AC010997.4	0.434385369	3.25E-26	postive
HELLS	AC026202.2	0.418086738	3.22E-24	postive
KLHL24	AC026202.2	0.404103086	1.37E-22	postive
GABPB1	AC026202.2	0.432030391	6.42E-26	postive
LINC00472	AC026202.2	0.779577726	3.30E-111	postive
ATM	AC026202.2	0.533195248	6.36E-41	postive
FBXW7	AC026202.2	0.426010654	3.56E-25	postive
IL33	LINC00924	0.463913557	4.05E-30	postive
HIC1	LINC00924	0.403241628	1.71E-22	postive
GPX4	PCCA-DT	0.429974386	1.16E-25	postive
FH	PCCA-DT	0.432739553	5.23E-26	postive
ISCU	PCCA-DT	0.428999999	1.53E-25	postive
ATP5MC3	PCCA-DT	0.45749571	3.08E-29	postive
GOT1	PCCA-DT	0.410312565	2.65E-23	postive
ATG4D	PCCA-DT	0.447654794	6.38E-28	postive
ISCU	ZNF793-AS1	0.456145067	4.70E-29	postive
ATG4D	ZNF793-AS1	0.501090874	1.31E-35	postive
BAP1	ZNF793-AS1	0.470712912	4.50E-31	postive
AGPAT3	AP000640.1	0.417485141	3.80E-24	postive
ACO1	AP000640.1	0.578690597	1.66E-49	postive
WIPI1	AP000640.1	0.481957045	1.07E-32	postive
HELLS	AC004908.1	0.432150697	6.20E-26	postive
ALOX12	AC004908.1	0.480846749	1.55E-32	postive
TAZ	AC004908.1	0.419680577	2.08E-24	postive
BRD4	AC073575.2	0.41911403	2.43E-24	postive
ZNF419	AC073575.2	0.484112674	5.12E-33	postive
TUBE1	AC073575.2	0.504463108	3.86E-36	postive
SETD1B	AC073575.2	0.513385281	1.41E-37	postive
ALOX12	AC073575.2	0.558011236	1.93E-45	postive
PHKG2	AC073575.2	0.508362548	9.20E-37	postive
TAZ	AC073575.2	0.605168963	3.73E-55	postive
FANCD2	AC114956.1	0.470870704	4.28E-31	postive
HELLS	AC114956.1	0.506966952	1.54E-36	postive
ALOX12	AC114956.1	0.481234014	1.36E-32	postive

TFAP2C	AC114956.1	0.417515282	3.77E-24	postive
ATM	AL592546.2	0.454909501	6.90E-29	postive
PSAT1	AC022509.4	0.430791104	9.16E-26	postive
HELLS	MIATNB	0.469852946	5.96E-31	postive
ZNF419	MIATNB	0.418910092	2.57E-24	postive
HBA1	MIATNB	0.436426293	1.80E-26	postive
GABPB1	MIATNB	0.450073481	3.06E-28	postive
ATG7	MIATNB	0.454263003	8.43E-29	postive
LINC00472	MIATNB	0.4396185	7.04E-27	postive
LPIN1	MIATNB	0.415718015	6.16E-24	postive
ATM	MIATNB	0.480494876	1.75E-32	postive
EGLN2	MIATNB	0.591971676	2.84E-52	postive
FBXW7	MIATNB	0.485367835	3.33E-33	postive
TUBE1	AC009065.8	0.404134633	1.36E-22	postive
NRAS	AC009065.8	-0.427386103	2.41E-25	negative
PHKG2	AC009065.8	0.667938245	6.51E-71	postive
ATG4D	AC009065.8	0.499479731	2.35E-35	postive
TAZ	AC009065.8	0.545094789	4.84E-43	postive
TFAP2C	AC118755.1	0.594725473	7.29E-53	postive
HBA1	AC118755.1	0.527918482	5.21E-40	postive
ALOX12	KDM2B-DT	0.422796147	8.76E-25	postive
HRAS	MIR4458HG	0.431963033	6.54E-26	postive
PHKG2	AC073195.1	0.590621063	5.50E-52	postive
TAZ	AC073195.1	0.625841121	6.01E-60	postive
HSF1	AC063948.1	0.414956734	7.58E-24	postive
NCOA4	AC063948.1	-0.438455634	9.92E-27	negative
PHKG2	AC063948.1	0.692805944	2.80E-78	postive
TAZ	AC063948.1	0.719098042	6.23E-87	postive
HSPB1	AC099518.6	0.443654514	2.13E-27	postive
HBA1	AC099518.6	0.591870386	2.98E-52	postive
HRAS	AC099518.6	0.619065431	2.45E-58	postive
MAP1LC3A	AC099518.6	0.42838874	1.82E-25	postive
EGLN2	AC099518.6	0.705516116	2.42E-82	postive
BRD4	AC022150.2	0.435197793	2.57E-26	postive
ZNF419	AC022150.2	0.56538745	7.40E-47	postive
VEGFA	AC022150.2	0.532207947	9.45E-41	postive
TUBE1	AC022150.2	0.506575642	1.78E-36	postive
SETD1B	AC022150.2	0.608540077	6.53E-56	postive
ALOX12	AC022150.2	0.617536488	5.59E-58	postive
YY1AP1	AC022150.2	0.443946017	1.95E-27	postive
TAZ	AC022150.2	0.561734071	3.76E-46	postive
HELLS	AC008870.2	0.463474266	4.66E-30	postive
BRD4	AC008870.2	0.458380275	2.34E-29	postive
ZNF419	AC008870.2	0.557430675	2.49E-45	postive
TUBE1	AC008870.2	0.591704799	3.23E-52	postive
SETD1B	AC008870.2	0.495219959	1.07E-34	postive
ALOX12	AC008870.2	0.594627436	7.65E-53	postive
GABPB1	AC008870.2	0.478088555	3.93E-32	postive
PHKG2	AC008870.2	0.507691415	1.18E-36	postive
LINC00472	AC008870.2	0.51731366	3.19E-38	postive
ATM	AC008870.2	0.492016064	3.32E-34	postive
TAZ	AC008870.2	0.581036725	5.51E-50	postive
FBXW7	AC008870.2	0.46819448	1.02E-30	postive
ANGPTL7	SEPTIN4-AS1	0.640737737	1.24E-63	postive
ALOX12	CERS3-AS1	0.400494573	3.50E-22	postive
HELLS	LINC00852	0.503089776	6.37E-36	postive
ZNF419	LINC00852	0.456873115	3.75E-29	postive
TUBE1	LINC00852	0.480978316	1.48E-32	postive

ALOX12	LINC00852	0.496171765	7.66E-35	postive
GABPB1	LINC00852	0.559378392	1.06E-45	postive
LINC00472	LINC00852	0.600375104	4.31E-54	postive
ATM	LINC00852	0.557349022	2.58E-45	postive
FBXW7	LINC00852	0.511236451	3.16E-37	postive
PHKG2	AL021392.1	0.404879194	1.12E-22	postive
TAZ	AL021392.1	0.451150198	2.20E-28	postive
HELLS	TMEM161B-/	0.481563283	1.22E-32	postive
ZNF419	TMEM161B-/	0.462006668	7.44E-30	postive
TUBE1	TMEM161B-/	0.538061768	8.86E-42	postive
ALOX12	TMEM161B-/	0.465332699	2.57E-30	postive
GABPB1	TMEM161B-/	0.483076862	7.29E-33	postive
LINC00472	TMEM161B-/	0.639068589	3.30E-63	postive
ATM	TMEM161B-/	0.609455118	4.05E-56	postive
FBXW7	TMEM161B-/	0.449271772	3.90E-28	postive
EIF2S1	AC026367.2	-0.417478571	3.81E-24	negative
PHKG2	AC026367.2	0.406621627	7.06E-23	postive
TAZ	AC026367.2	0.431180011	8.19E-26	postive
FH	PDE11A-AS1	0.408847625	3.91E-23	postive
ISCU	PDE11A-AS1	0.547894167	1.49E-43	postive
DDIT3	PDE11A-AS1	0.471107511	3.96E-31	postive
GOT1	PDE11A-AS1	0.448698584	4.65E-28	postive
ATG4D	PDE11A-AS1	0.502907665	6.80E-36	postive
GABARAPL2	PDE11A-AS1	0.441299482	4.28E-27	postive
GABARAPL1	PDE11A-AS1	0.638618474	4.28E-63	postive
LPIN1	PDE11A-AS1	0.425595098	4.00E-25	postive
KLHL24	AC018552.3	0.525493733	1.35E-39	postive
GABPB1	AC018552.3	0.434985514	2.73E-26	postive
PIK3CA	AC018552.3	0.412855041	1.34E-23	postive
LINC00472	AC018552.3	0.867968167	2.31E-165	postive
LPIN1	AC018552.3	0.434162478	3.47E-26	postive
ATM	AC018552.3	0.416925435	4.43E-24	postive
FANCD2	AP002812.5	0.403823366	1.47E-22	postive
HELLS	AP002812.5	0.501521536	1.12E-35	postive
TUBE1	AP002812.5	0.412428791	1.50E-23	postive
ALOX12	AP002812.5	0.499882995	2.03E-35	postive
TFAP2C	AP002812.5	0.438069294	1.11E-26	postive
GABPB1	AP002812.5	0.430736331	9.30E-26	postive
DUOX1	AP002812.5	0.439709775	6.86E-27	postive
LINC00472	AP002812.5	0.449948627	3.18E-28	postive
ATM	AP002812.5	0.437706406	1.24E-26	postive
FBXW7	AP002812.5	0.435711354	2.21E-26	postive
DRD4	Z69706.1	0.404105648	1.37E-22	postive
HRAS	Z69706.1	0.426485238	3.11E-25	postive
NCOA4	Z69706.1	-0.436448457	1.78E-26	negative
PHKG2	Z69706.1	0.552600267	2.01E-44	postive
EGLN2	Z69706.1	0.466667489	1.67E-30	postive
TAZ	Z69706.1	0.65041335	3.90E-66	postive
HELLS	AC026470.2	0.471423912	3.57E-31	postive
KLHL24	AC026470.2	0.488538885	1.12E-33	postive
IREB2	AC026470.2	0.429205874	1.44E-25	postive
GABPB1	AC026470.2	0.544852596	5.35E-43	postive
PIK3CA	AC026470.2	0.455958043	4.98E-29	postive
ATG7	AC026470.2	0.401377701	2.78E-22	postive
MAPK8	AC026470.2	0.411232033	2.07E-23	postive
LINC00472	AC026470.2	0.889429871	9.98E-185	postive
ATM	AC026470.2	0.636744346	1.27E-62	postive
FBXW7	AC026470.2	0.42393944	6.37E-25	postive

TUBE1	AL139246.3	0.455180297	6.35E-29	postive
ALOX12	AL139246.3	0.477844137	4.26E-32	postive
TAZ	AL139246.3	0.48905729	9.32E-34	postive
GABPB1	AC034198.2	0.414537739	8.49E-24	postive
LINC00472	AC034198.2	0.485432956	3.26E-33	postive
HELLS	ADAMTS9-A	0.484011329	5.30E-33	postive
TUBE1	ADAMTS9-A	0.521615291	6.11E-39	postive
ALOX12	ADAMTS9-A	0.523630372	2.80E-39	postive
IREB2	ADAMTS9-A	0.461541851	8.62E-30	postive
GABPB1	ADAMTS9-A	0.526029665	1.10E-39	postive
MAPK8	ADAMTS9-A	0.51906163	1.63E-38	postive
LINC00472	ADAMTS9-A	0.494149981	1.57E-34	postive
PRKAA2	ADAMTS9-A	0.477388108	4.96E-32	postive
ATM	ADAMTS9-A	0.781427022	4.53E-112	postive
FBXW7	ADAMTS9-A	0.48438437	4.66E-33	postive
ATP6V1G2	LINC02365	0.524358385	2.11E-39	postive
ACO1	LINC02365	0.668307732	5.12E-71	postive
GCLC	AL161782.1	0.447492779	6.70E-28	postive
TUBE1	AL161782.1	0.442207238	3.27E-27	postive
IREB2	AL161782.1	0.546840075	2.33E-43	postive
PIK3CA	AL161782.1	0.434880996	2.82E-26	postive
NCOA4	AL161782.1	0.438305068	1.04E-26	postive
SNX4	AL161782.1	0.441689718	3.82E-27	postive
MAPK1	AL161782.1	0.479510714	2.44E-32	postive
MAPK8	AL161782.1	0.589732988	8.48E-52	postive
PRKAA2	AL161782.1	0.606635775	1.75E-55	postive
PRKAA1	AL161782.1	0.426186251	3.39E-25	postive
TLR4	AL161782.1	0.434274734	3.36E-26	postive
ATM	AL161782.1	0.608155445	7.97E-56	postive
SIRT1	AL161782.1	0.442478768	3.02E-27	postive
HELLS	AL133410.1	0.455570775	5.62E-29	postive
ZNF419	AL133410.1	0.52315701	3.36E-39	postive
TUBE1	AL133410.1	0.552858738	1.80E-44	postive
DRD4	AL133410.1	0.412084414	1.65E-23	postive
ALOX12	AL133410.1	0.53863235	7.01E-42	postive
PHKG2	AL133410.1	0.593913464	1.09E-52	postive
TAZ	AL133410.1	0.636279284	1.66E-62	postive
HELLS	AC027763.2	0.577951426	2.35E-49	postive
ZNF419	AC027763.2	0.424791534	5.02E-25	postive
ALOX12	AC027763.2	0.416818687	4.56E-24	postive
GABPB1	AC027763.2	0.444488393	1.66E-27	postive
DUOX1	AC027763.2	0.403265196	1.70E-22	postive
ATG7	AC027763.2	0.401690112	2.57E-22	postive
LINC00472	AC027763.2	0.439546436	7.19E-27	postive
ATM	AC027763.2	0.509774195	5.45E-37	postive
FBXW7	AC027763.2	0.463466621	4.68E-30	postive
ZNF419	AL645941.1	0.404857946	1.12E-22	postive
TUBE1	AL645941.1	0.526995721	7.49E-40	postive
SETD1B	AL645941.1	0.428078719	1.98E-25	postive
ALOX12	AL645941.1	0.591866038	2.99E-52	postive
GABPB1	AL645941.1	0.413762533	1.05E-23	postive
ATM	AL645941.1	0.591956486	2.86E-52	postive
HELLS	RAB30-DT	0.48579634	2.87E-33	postive
TUBE1	RAB30-DT	0.56276018	2.39E-46	postive
ALOX12	RAB30-DT	0.5293881	2.91E-40	postive
GABPB1	RAB30-DT	0.528474411	4.18E-40	postive
MAPK8	RAB30-DT	0.465970859	2.10E-30	postive
LINC00472	RAB30-DT	0.549877727	6.44E-44	postive

ATM	RAB30-DT	0.675390927	4.81E-73	postive
HELLS	NDUFV2-AS1	0.491442258	4.06E-34	postive
ZNF419	NDUFV2-AS1	0.546891114	2.28E-43	postive
TUBE1	NDUFV2-AS1	0.529516317	2.77E-40	postive
ALOX12	NDUFV2-AS1	0.573970028	1.49E-48	postive
GABPB1	NDUFV2-AS1	0.602408513	1.53E-54	postive
LINC00472	NDUFV2-AS1	0.699599851	2.00E-80	postive
ATM	NDUFV2-AS1	0.535268666	2.76E-41	postive
FBXW7	NDUFV2-AS1	0.491833565	3.54E-34	postive
HSF1	AC084125.4	0.440182622	5.96E-27	postive
PHKG2	AC084125.4	0.445073844	1.39E-27	postive
ATG4D	AC084125.4	0.437929246	1.16E-26	postive
LONP1	AC084125.4	0.418685276	2.74E-24	postive
PHKG2	AP000238.1	0.539092211	5.81E-42	postive
TAZ	AP000238.1	0.56992237	9.55E-48	postive
FBXW7	AP000238.1	0.418212613	3.11E-24	postive
ALB	AC008760.2	0.485525239	3.16E-33	postive
CDO1	AC008760.2	0.42621736	3.36E-25	postive
HSPB1	AC092718.3	0.479189686	2.71E-32	postive
NOX1	AC092718.3	0.480794938	1.58E-32	postive
NCOA4	AC092718.3	-0.45480769	7.13E-29	negative
PHKG2	AC092718.3	0.5296244	2.65E-40	postive
BECN1	AC092718.3	-0.433773804	3.88E-26	negative
SIRT1	AC092718.3	-0.439109486	8.18E-27	negative
HELLS	AP005329.1	0.424453711	5.51E-25	postive
ZNF419	AP005329.1	0.468418834	9.50E-31	postive
TUBE1	AP005329.1	0.49708001	5.54E-35	postive
ALOX12	AP005329.1	0.431449325	7.58E-26	postive
GABPB1	AP005329.1	0.458562327	2.21E-29	postive
PHKG2	AP005329.1	0.494333985	1.47E-34	postive
ATM	AP005329.1	0.431665273	7.13E-26	postive
TAZ	AP005329.1	0.54805221	1.40E-43	postive
FBXW7	AP005329.1	0.460583108	1.17E-29	postive
CHMP5	COMETT	0.400442764	3.55E-22	postive
TAZ	AP000345.2	0.434287535	3.34E-26	postive
HELLS	NR2F2-AS1	0.518779125	1.82E-38	postive
ZNF419	NR2F2-AS1	0.465751646	2.25E-30	postive
TUBE1	NR2F2-AS1	0.591653075	3.32E-52	postive
ALOX12	NR2F2-AS1	0.596589275	2.89E-53	postive
GABPB1	NR2F2-AS1	0.613238481	5.54E-57	postive
ZEB1	NR2F2-AS1	0.421294506	1.33E-24	postive
MAPK8	NR2F2-AS1	0.531643203	1.19E-40	postive
LINC00472	NR2F2-AS1	0.613715402	4.30E-57	postive
ATM	NR2F2-AS1	0.771258326	1.98E-107	postive
FBXW7	NR2F2-AS1	0.575966292	5.93E-49	postive
TUBE1	AC090246.1	0.408384384	4.43E-23	postive
ATM	AC090246.1	0.410718525	2.38E-23	postive
HELLS	AL355916.2	0.466630342	1.69E-30	postive
KLHL24	AL355916.2	0.442603567	2.91E-27	postive
TUBE1	AL355916.2	0.471789856	3.17E-31	postive
ALOX12	AL355916.2	0.452298298	1.55E-28	postive
IREB2	AL355916.2	0.49555592	9.53E-35	postive
GABPB1	AL355916.2	0.608650378	6.16E-56	postive
PIK3CA	AL355916.2	0.470605754	4.66E-31	postive
ATG7	AL355916.2	0.435024271	2.70E-26	postive
MAPK8	AL355916.2	0.499410622	2.41E-35	postive
LINC00472	AL355916.2	0.787633461	5.00E-115	postive
ATM	AL355916.2	0.742070626	2.40E-95	postive

FBXW7	AL355916.2	0.538272888	8.12E-42	postive
LINC00472	AC007014.2	0.583547413	1.67E-50	postive
ACSL4	AC091563.1	0.427682672	2.22E-25	postive
HIC1	AC147067.2	0.437632084	1.26E-26	postive
EPAS1	AC147067.2	0.401944152	2.40E-22	postive
ATM	AL132657.1	0.50140592	1.17E-35	postive
BRD4	AP001160.1	0.441793756	3.70E-27	postive
ZNF419	AP001160.1	0.486186	2.51E-33	postive
TUBE1	AP001160.1	0.467378821	1.33E-30	postive
DRD4	AP001160.1	0.478428278	3.50E-32	postive
ALOX12	AP001160.1	0.527875606	5.30E-40	postive
SCP2	AP001160.1	-0.423161851	7.91E-25	negative
NCOA4	AP001160.1	-0.472755505	2.31E-31	negative
PHKG2	AP001160.1	0.620544392	1.10E-58	postive
EGLN2	AP001160.1	0.44791708	5.89E-28	postive
TAZ	AP001160.1	0.747525141	1.77E-97	postive
ARNTL	CRYZL2P-SE(	0.405210339	1.02E-22	postive
ZNF419	CRYZL2P-SE(	0.436376847	1.82E-26	postive
VEGFA	CRYZL2P-SE(	0.468580272	9.02E-31	postive
TUBE1	CRYZL2P-SE(	0.684428552	1.03E-75	postive
SETD1B	CRYZL2P-SE(	0.416410121	5.10E-24	postive
ALOX12	CRYZL2P-SE(	0.690232136	1.75E-77	postive
SP1	CRYZL2P-SE(	0.404458917	1.25E-22	postive
GABPB1	CRYZL2P-SE(	0.412883799	1.33E-23	postive
MAPK8	CRYZL2P-SE(	0.516729474	3.98E-38	postive
ATM	CRYZL2P-SE(	0.720698081	1.72E-87	postive
YY1AP1	CRYZL2P-SE(	0.431145688	8.27E-26	postive
FBXW7	CRYZL2P-SE(	0.424126665	6.04E-25	postive
ACVR1B	C8orf31	0.411782004	1.79E-23	postive
VEGFA	PHKA2-AS1	0.490401026	5.84E-34	postive
ALOX12	PHKA2-AS1	0.429812051	1.21E-25	postive
TAZ	PHKA2-AS1	0.525679217	1.26E-39	postive
HELLS	GAS8-AS1	0.462601914	6.16E-30	postive
ZNF419	GAS8-AS1	0.504223702	4.21E-36	postive
TUBE1	GAS8-AS1	0.542576488	1.38E-42	postive
ALOX12	GAS8-AS1	0.692857334	2.70E-78	postive
GABPB1	GAS8-AS1	0.413363289	1.17E-23	postive
ATM	GAS8-AS1	0.509695787	5.61E-37	postive
FBXW7	GAS8-AS1	0.43770195	1.24E-26	postive
NOX1	AC100814.1	0.637776328	6.98E-63	postive
BECN1	AC100814.1	-0.469427616	6.85E-31	negative
VEGFA	AC024909.1	0.412598796	1.43E-23	postive
ZEB1	AC024909.1	0.450470981	2.71E-28	postive
HELLS	MORF4L2-AS	0.444496977	1.65E-27	postive
ZNF419	MORF4L2-AS	0.561356592	4.45E-46	postive
TUBE1	MORF4L2-AS	0.538541955	7.28E-42	postive
ALOX12	MORF4L2-AS	0.690027194	2.03E-77	postive
GABPB1	MORF4L2-AS	0.45076126	2.48E-28	postive
DUOX1	MORF4L2-AS	0.406955521	6.46E-23	postive
LINC00472	MORF4L2-AS	0.41547741	6.58E-24	postive
ATM	MORF4L2-AS	0.459754674	1.52E-29	postive
HELLS	AC019080.5	0.534679585	3.50E-41	postive
TUBE1	AC019080.5	0.473680318	1.70E-31	postive
GABPB1	AC019080.5	0.442195216	3.28E-27	postive
LINC00472	AC019080.5	0.501971244	9.55E-36	postive
ATM	AC019080.5	0.637546336	7.97E-63	postive
HELLS	ITPRIP-AS1	0.512650556	1.86E-37	postive
TUBE1	ITPRIP-AS1	0.440088693	6.13E-27	postive

ALOX12	ITPRIP-AS1	0.407681887	5.33E-23	postive
IREB2	ITPRIP-AS1	0.401623102	2.61E-22	postive
GABPB1	ITPRIP-AS1	0.567546749	2.80E-47	postive
PIK3CA	ITPRIP-AS1	0.414220415	9.25E-24	postive
ATG7	ITPRIP-AS1	0.433942247	3.70E-26	postive
MAPK8	ITPRIP-AS1	0.444549811	1.63E-27	postive
LINC00472	ITPRIP-AS1	0.752484716	1.83E-99	postive
ATM	ITPRIP-AS1	0.719132617	6.06E-87	postive
FBXW7	ITPRIP-AS1	0.487971468	1.36E-33	postive
ANGPTL7	MAP3K4-AS1	0.410781331	2.34E-23	postive
CDKN2A	MAP3K4-AS1	0.48446017	4.54E-33	postive
IL6	AC015819.1	0.404310205	1.30E-22	postive
TFAP2C	AL391832.2	0.536036096	2.02E-41	postive
HBA1	AL391832.2	0.540810512	2.87E-42	postive
HELLS	AC068790.7	0.54030015	3.54E-42	postive
TUBE1	AC068790.7	0.480691886	1.64E-32	postive
ALOX12	AC068790.7	0.472856391	2.23E-31	postive
GABPB1	AC068790.7	0.560583699	6.25E-46	postive
ATG7	AC068790.7	0.401479198	2.71E-22	postive
MAPK8	AC068790.7	0.413476068	1.13E-23	postive
LINC00472	AC068790.7	0.667529402	8.49E-71	postive
ATM	AC068790.7	0.747676628	1.54E-97	postive
FBXW7	AC068790.7	0.495869463	8.53E-35	postive
HSPB1	GS1-124K5.4	0.485889833	2.78E-33	postive
PHKG2	GS1-124K5.4	0.468535203	9.15E-31	postive
TAZ	GS1-124K5.4	0.417677803	3.61E-24	postive
SETD1B	ADAMTSL4-A	0.42606053	3.51E-25	postive
ALOX12	ADAMTSL4-A	0.480657963	1.65E-32	postive
KLHL24	FAM198B-AS	0.578144921	2.15E-49	postive
IREB2	FAM198B-AS	0.431253408	8.02E-26	postive
PIK3CA	FAM198B-AS	0.484643883	4.27E-33	postive
KRAS	FAM198B-AS	0.452556532	1.43E-28	postive
LINC00472	FAM198B-AS	0.808250486	1.31E-125	postive
PRKAA1	FAM198B-AS	0.409806127	3.03E-23	postive
LPIN1	FAM198B-AS	0.50790022	1.09E-36	postive
ZNF419	AL122125.1	0.531722897	1.15E-40	postive
TUBE1	AL122125.1	0.423222346	7.78E-25	postive
ALOX12	AL122125.1	0.492929322	2.41E-34	postive
TAZ	AL122125.1	0.592708596	1.98E-52	postive
FH	Z99572.1	0.407665286	5.36E-23	postive
ISCU	Z99572.1	0.519179675	1.56E-38	postive
HERPUD1	Z99572.1	0.438194735	1.07E-26	postive
KLHL24	Z99572.1	0.425845902	3.73E-25	postive
SLC2A12	Z99572.1	0.493203199	2.19E-34	postive
CS	Z99572.1	0.500640258	1.55E-35	postive
GOT1	Z99572.1	0.452453623	1.47E-28	postive
GABARAPL1	Z99572.1	0.713895729	3.84E-85	postive
WIPI2	Z99572.1	0.454629141	7.53E-29	postive
BAP1	Z99572.1	0.489688449	7.49E-34	postive
LPIN1	Z99572.1	0.524353087	2.11E-39	postive
VEGFA	ERVK9-11	0.429936524	1.17E-25	postive
TUBE1	ERVK9-11	0.465505411	2.43E-30	postive
ALOX12	ERVK9-11	0.458097448	2.55E-29	postive
TAZ	ERVK9-11	0.417431716	3.86E-24	postive
HBA1	AC011472.2	0.496064611	7.95E-35	postive
HELLS	PSPC1-AS2	0.532385128	8.81E-41	postive
ZNF419	PSPC1-AS2	0.508668625	8.21E-37	postive
TUBE1	PSPC1-AS2	0.637348274	8.93E-63	postive

ALOX12	PSPC1-AS2	0.55177615	2.87E-44	postive
GABPB1	PSPC1-AS2	0.606032447	2.39E-55	postive
LINC00472	PSPC1-AS2	0.589441236	9.78E-52	postive
ATM	PSPC1-AS2	0.650999111	2.73E-66	postive
TAZ	PSPC1-AS2	0.412228768	1.58E-23	postive
FBXW7	PSPC1-AS2	0.56919744	1.33E-47	postive
FANCD2	C10orf55	0.45167956	1.87E-28	postive
AURKA	C10orf55	0.417258251	4.05E-24	postive
GPX4	AP001505.1	0.404051924	1.39E-22	postive
HSPB1	AP001505.1	0.566327564	4.85E-47	postive
IREB2	AP001505.1	-0.449871237	3.25E-28	negative
HBA1	AP001505.1	0.410785388	2.33E-23	postive
HRAS	AP001505.1	0.720374049	2.23E-87	postive
NCOA4	AP001505.1	-0.457669036	2.92E-29	negative
PHKG2	AP001505.1	0.471835327	3.12E-31	postive
BECN1	AP001505.1	-0.425580733	4.02E-25	negative
MAP1LC3A	AP001505.1	0.410149032	2.77E-23	postive
PRKAA1	AP001505.1	-0.405218079	1.02E-22	negative
EGLN2	AP001505.1	0.68750293	1.20E-76	postive
ISCU	AC016705.2	0.403646216	1.54E-22	postive
CS	AC016705.2	0.446654932	8.63E-28	postive
GABARAPL1	AC016705.2	0.499212606	2.58E-35	postive
LPIN1	AC016705.2	0.402651215	2.00E-22	postive
HELLS	AL008721.2	0.59838224	1.18E-53	postive
ZNF419	AL008721.2	0.44742061	6.85E-28	postive
TUBE1	AL008721.2	0.468587073	9.00E-31	postive
ALOX12	AL008721.2	0.514332148	9.88E-38	postive
GABPB1	AL008721.2	0.512800073	1.76E-37	postive
LINC00472	AL008721.2	0.518915662	1.73E-38	postive
ATM	AL008721.2	0.61839309	3.53E-58	postive
FBXW7	AL008721.2	0.499660299	2.20E-35	postive
SOCS1	BX470102.1	0.440358752	5.66E-27	postive
ISCU	AC103563.2	0.434375142	3.26E-26	postive
KLHL24	AC103563.2	0.566892144	3.76E-47	postive
SLC2A12	AC103563.2	0.409267741	3.50E-23	postive
ATG4D	AC103563.2	0.407773459	5.21E-23	postive
GABARAPL1	AC103563.2	0.613152305	5.80E-57	postive
WIPI2	AC103563.2	0.45960356	1.59E-29	postive
LINC00472	AC103563.2	0.609013749	5.10E-56	postive
LPIN1	AC103563.2	0.602794885	1.26E-54	postive
SETD1B	AL596244.1	0.425914669	3.66E-25	postive
PHKG2	AC092809.4	0.579156081	1.34E-49	postive
TAZ	AC092809.4	0.601211085	2.82E-54	postive
PML	U62317.1	0.400882296	3.17E-22	postive
DRD4	U62317.1	0.427331669	2.45E-25	postive
NCOA4	U62317.1	-0.431682162	7.09E-26	negative
PHKG2	U62317.1	0.657842571	4.02E-68	postive
TAZ	U62317.1	0.712779137	9.18E-85	postive
KLHL24	AC010333.1	0.42538557	4.24E-25	postive
GABPB1	AC010333.1	0.409449588	3.33E-23	postive
LINC00472	AC010333.1	0.624015624	1.65E-59	postive
LPIN1	AC010333.1	0.435604451	2.28E-26	postive
ATM	AC010333.1	0.581371427	4.70E-50	postive
ISCU	UBA6-AS1	0.4610584	1.01E-29	postive
ATG4D	UBA6-AS1	0.453646212	1.02E-28	postive
HELLS	AL442125.2	0.523204926	3.30E-39	postive
ZNF419	AL442125.2	0.424265862	5.81E-25	postive
TUBE1	AL442125.2	0.498276554	3.61E-35	postive

ALOX12	AL442125.2	0.47457283	1.27E-31	postive
IREB2	AL442125.2	0.41539759	6.72E-24	postive
GABPB1	AL442125.2	0.56310383	2.05E-46	postive
ATG7	AL442125.2	0.400789757	3.24E-22	postive
MAPK8	AL442125.2	0.434418358	3.22E-26	postive
LINC00472	AL442125.2	0.64359555	2.32E-64	postive
ATM	AL442125.2	0.687610735	1.11E-76	postive
FBXW7	AL442125.2	0.484884105	3.93E-33	postive
BRD4	AC020907.4	0.483189309	7.01E-33	postive
ZNF419	AC020907.4	0.536873785	1.44E-41	postive
VEGFA	AC020907.4	0.511936567	2.43E-37	postive
TUBE1	AC020907.4	0.487181003	1.78E-33	postive
SETD1B	AC020907.4	0.478502088	3.42E-32	postive
DRD4	AC020907.4	0.434252408	3.38E-26	postive
ALOX12	AC020907.4	0.57733276	3.14E-49	postive
PHKG2	AC020907.4	0.518025074	2.43E-38	postive
TAZ	AC020907.4	0.676620673	2.11E-73	postive
TUBE1	AC092747.4	0.516299055	4.69E-38	postive
HMGB1	AC092747.4	0.444522765	1.64E-27	postive
GABPB1	AC092747.4	0.441420818	4.13E-27	postive
NOX1	AC092747.4	0.470939997	4.18E-31	postive
MAPK8	AC092747.4	0.427804774	2.14E-25	postive
HELLS	AC027801.1	0.475623576	8.93E-32	postive
ZNF419	AC027801.1	0.46037449	1.25E-29	postive
TUBE1	AC027801.1	0.502377331	8.25E-36	postive
ALOX12	AC027801.1	0.526470029	9.21E-40	postive
GABPB1	AC027801.1	0.458707813	2.11E-29	postive
LINC00472	AC027801.1	0.477734343	4.42E-32	postive
ATM	AC027801.1	0.517123687	3.43E-38	postive
TAZ	AC027801.1	0.434951371	2.76E-26	postive
FBXW7	AC027801.1	0.472336379	2.65E-31	postive
RB1	AC008669.1	0.435888231	2.10E-26	postive
TMBIM4	AC008669.1	0.474398632	1.34E-31	postive
CHMP5	AC008669.1	0.441110719	4.53E-27	postive
KLHL24	AC008669.1	0.474292524	1.39E-31	postive
IREB2	AC008669.1	0.462326608	6.72E-30	postive
EMC2	AC008669.1	0.492155997	3.16E-34	postive
PIK3CA	AC008669.1	0.444272856	1.77E-27	postive
SCP2	AC008669.1	0.41694944	4.40E-24	postive
KRAS	AC008669.1	0.529743945	2.53E-40	postive
GABARAPL2	AC008669.1	0.447906474	5.91E-28	postive
MAPK8	AC008669.1	0.497982755	4.01E-35	postive
MAPK9	AC008669.1	0.436768564	1.63E-26	postive
LINC00472	AC008669.1	0.471669549	3.29E-31	postive
PRKAA2	AC008669.1	0.571085768	5.62E-48	postive
PRKAA1	AC008669.1	0.565890708	5.90E-47	postive
ZNF419	KLF3-AS1	0.534734034	3.42E-41	postive
VEGFA	KLF3-AS1	0.561142207	4.89E-46	postive
TUBE1	KLF3-AS1	0.633052279	1.05E-61	postive
SETD1B	KLF3-AS1	0.468412646	9.52E-31	postive
ALOX12	KLF3-AS1	0.620363931	1.21E-58	postive
GABPB1	KLF3-AS1	0.405507924	9.47E-23	postive
MAPK8	KLF3-AS1	0.418152338	3.17E-24	postive
ATM	KLF3-AS1	0.481696161	1.16E-32	postive
IFNG	LINC01232	0.426937317	2.74E-25	postive
IDH1	LINC01232	0.439224226	7.91E-27	postive
TFAP2C	LINC02356	0.561674105	3.86E-46	postive
HBA1	LINC02356	0.57027417	8.13E-48	postive

HELLS	LINC02328	0.407125952	6.18E-23	postive
ATM	LINC02328	0.407703617	5.30E-23	postive
ALOX12	AC136475.1	0.438663474	9.33E-27	postive
TAZ	AC136475.1	0.447717283	6.26E-28	postive
VEGFA	LINC00294	0.409487252	3.30E-23	postive
ALOX12	LINC00294	0.414509071	8.56E-24	postive
HELLS	AC005104.1	0.419549173	2.16E-24	postive
BRD4	AC005104.1	0.422686913	9.03E-25	postive
ZNF419	AC005104.1	0.524216556	2.23E-39	postive
VEGFA	AC005104.1	0.505167753	2.98E-36	postive
TUBE1	AC005104.1	0.648137885	1.54E-65	postive
SETD1B	AC005104.1	0.514283104	1.01E-37	postive
ALOX12	AC005104.1	0.674762953	7.31E-73	postive
GABPB1	AC005104.1	0.481295752	1.33E-32	postive
ATM	AC005104.1	0.537757609	1.00E-41	postive
YY1AP1	AC005104.1	0.443846086	2.01E-27	postive
TAZ	AC005104.1	0.500296878	1.75E-35	postive
FBXW7	AC005104.1	0.475493167	9.33E-32	postive
SLC3A2	CEROX1	0.43853082	9.70E-27	postive
ISCU	CEROX1	0.485844822	2.83E-33	postive
CHAC1	CEROX1	0.436869114	1.58E-26	postive
DDIT3	CEROX1	0.480051194	2.03E-32	postive
SLC2A12	CEROX1	0.431161732	8.23E-26	postive
PLIN4	CEROX1	0.404605938	1.20E-22	postive
GOT1	CEROX1	0.425269254	4.39E-25	postive
WIPI2	CEROX1	0.510829545	3.68E-37	postive
ALOX12	AC104825.1	0.418648405	2.76E-24	postive
VDAC2	ZNF503-AS2	0.435552192	2.32E-26	postive
ISCU	ZNF503-AS2	0.621251527	7.49E-59	postive
OTUB1	ZNF503-AS2	0.41755734	3.73E-24	postive
TMBIM4	ZNF503-AS2	0.442394636	3.09E-27	postive
CHMP5	ZNF503-AS2	0.427044685	2.66E-25	postive
DDIT3	ZNF503-AS2	0.432216565	6.08E-26	postive
ATP5MC3	ZNF503-AS2	0.451464098	2.00E-28	postive
GOT1	ZNF503-AS2	0.506423584	1.88E-36	postive
ATG4D	ZNF503-AS2	0.512510408	1.96E-37	postive
MAP1LC3A	ZNF503-AS2	0.419002815	2.51E-24	postive
GABARAPL2	ZNF503-AS2	0.584402123	1.11E-50	postive
BAP1	ZNF503-AS2	0.400031034	3.95E-22	postive
HELLS	AC004471.1	0.400638226	3.37E-22	postive
RPL8	PIK3CD-AS2	0.417259783	4.04E-24	postive
HRAS	PIK3CD-AS2	0.434758256	2.92E-26	postive
ZNF419	AC105345.1	0.541751479	1.94E-42	postive
VEGFA	AC105345.1	0.560329947	6.99E-46	postive
TUBE1	AC105345.1	0.446939382	7.92E-28	postive
SETD1B	AC105345.1	0.466147258	1.98E-30	postive
ALOX12	AC105345.1	0.5394635	4.99E-42	postive
ULK1	AC105345.1	0.408850394	3.91E-23	postive
TAZ	AC105345.1	0.490757073	5.16E-34	postive
RB1	WDFY3-AS2	0.539979777	4.03E-42	postive
GCLC	WDFY3-AS2	0.407072795	6.27E-23	postive
CISD2	WDFY3-AS2	0.542057289	1.71E-42	postive
CHMP5	WDFY3-AS2	0.459550314	1.62E-29	postive
BNIP3	WDFY3-AS2	0.402487105	2.09E-22	postive
HSD17B11	WDFY3-AS2	0.498199415	3.72E-35	postive
IREB2	WDFY3-AS2	0.430982109	8.67E-26	postive
EMC2	WDFY3-AS2	0.429707992	1.25E-25	postive
SCP2	WDFY3-AS2	0.566467194	4.56E-47	postive

NRAS	WDFY3-AS2	0.502513548	7.85E-36	postive
KRAS	WDFY3-AS2	0.407781219	5.19E-23	postive
CARS1	WDFY3-AS2	-0.411650331	1.85E-23	negative
NCOA4	WDFY3-AS2	0.491067634	4.63E-34	postive
SNX4	WDFY3-AS2	0.616450423	1.00E-57	postive
MAPK1	WDFY3-AS2	0.586807602	3.50E-51	postive
MAPK8	WDFY3-AS2	0.5075038	1.26E-36	postive
PRKAA2	WDFY3-AS2	0.610210675	2.73E-56	postive
PRKAA1	WDFY3-AS2	0.413709002	1.06E-23	postive
SIRT1	WDFY3-AS2	0.496838133	6.04E-35	postive
HELLS	NPTN-IT1	0.408150799	4.71E-23	postive
ZNF419	NPTN-IT1	0.455829042	5.19E-29	postive
KLHL24	NPTN-IT1	0.56682905	3.87E-47	postive
TUBE1	NPTN-IT1	0.456116749	4.74E-29	postive
ALOX12	NPTN-IT1	0.445202009	1.34E-27	postive
IREB2	NPTN-IT1	0.528972319	3.43E-40	postive
GABPB1	NPTN-IT1	0.573547371	1.82E-48	postive
PIK3CA	NPTN-IT1	0.523866202	2.55E-39	postive
KRAS	NPTN-IT1	0.433746293	3.91E-26	postive
MAPK8	NPTN-IT1	0.513314215	1.45E-37	postive
LINC00472	NPTN-IT1	0.897866609	1.82E-193	postive
PRKAA1	NPTN-IT1	0.420665572	1.58E-24	postive
LPIN1	NPTN-IT1	0.426719007	2.91E-25	postive
ATM	NPTN-IT1	0.632472258	1.46E-61	postive
FBXW7	NPTN-IT1	0.467974654	1.10E-30	postive
TUBE1	AL035701.1	0.451475401	1.99E-28	postive
TUBE1	AC010538.1	0.42215033	1.05E-24	postive
ALOX12	AC010538.1	0.583549388	1.67E-50	postive
PHKG2	AC010538.1	0.449515	3.63E-28	postive
TAZ	AC010538.1	0.426622557	3.00E-25	postive
TFAP2C	AC024941.2	0.45674362	3.90E-29	postive
ZNF419	AL050341.2	0.467334071	1.35E-30	postive
TUBE1	AL050341.2	0.513577672	1.31E-37	postive
GABPB1	AL050341.2	0.462400275	6.57E-30	postive
TAZ	AL050341.2	0.452841524	1.31E-28	postive
HELLS	NARF-IT1	0.49071172	5.24E-34	postive
BRD4	NARF-IT1	0.446672224	8.59E-28	postive
ZNF419	NARF-IT1	0.536284642	1.83E-41	postive
VEGFA	NARF-IT1	0.483548338	6.20E-33	postive
TUBE1	NARF-IT1	0.603464431	8.95E-55	postive
SETD1B	NARF-IT1	0.504677045	3.57E-36	postive
ALOX12	NARF-IT1	0.709710177	9.88E-84	postive
GABPB1	NARF-IT1	0.510129931	4.77E-37	postive
ATM	NARF-IT1	0.60842199	6.94E-56	postive
YY1AP1	NARF-IT1	0.423510316	7.18E-25	postive
TAZ	NARF-IT1	0.42897093	1.54E-25	postive
FBXW7	NARF-IT1	0.519132014	1.59E-38	postive
ZNF419	AL133215.1	0.465632747	2.34E-30	postive
VEGFA	AL133215.1	0.404653011	1.19E-22	postive
TUBE1	AL133215.1	0.500559431	1.59E-35	postive
SETD1B	AL133215.1	0.503701068	5.10E-36	postive
ALOX12	AL133215.1	0.606521018	1.86E-55	postive
TAZ	AL133215.1	0.483546788	6.21E-33	postive
OTUB1	AC025181.2	0.416184003	5.43E-24	postive
SLC2A8	AC025181.2	0.484673136	4.22E-33	postive
VEGFA	AL391845.2	0.415548115	6.45E-24	postive
HIC1	AL391845.2	0.437526446	1.30E-26	postive
ZEB1	AL391845.2	0.61008351	2.92E-56	postive

EPAS1	AL391845.2	0.508491427	8.77E-37	postive
HSPB1	AC046143.2	0.532015397	1.02E-40	postive
SLC2A6	AC046143.2	0.402604935	2.02E-22	postive
HBA1	AC046143.2	0.415042081	7.40E-24	postive
NOX1	AC046143.2	0.405600707	9.24E-23	postive
HRAS	AC046143.2	0.543577921	9.11E-43	postive
NCOA4	AC046143.2	-0.471297199	3.72E-31	negative
PHKG2	AC046143.2	0.514158843	1.05E-37	postive
MAPK1	AC046143.2	-0.425922851	3.65E-25	negative
EGLN2	AC046143.2	0.527706509	5.66E-40	postive
TAZ	AC046143.2	0.432919665	4.97E-26	postive
NCOA4	U47924.2	-0.417350144	3.95E-24	negative
PHKG2	U47924.2	0.481802442	1.12E-32	postive
TAZ	U47924.2	0.468999752	7.87E-31	postive
SLC3A2	SLC25A5-AS	0.462868807	5.66E-30	postive
FH	SLC25A5-AS	0.509590284	5.83E-37	postive
ISCU	SLC25A5-AS	0.703664509	9.75E-82	postive
ACSL3	SLC25A5-AS	0.428677902	1.67E-25	postive
DDIT3	SLC25A5-AS	0.429691243	1.25E-25	postive
KLHL24	SLC25A5-AS	0.465371667	2.54E-30	postive
SLC2A12	SLC25A5-AS	0.431667098	7.12E-26	postive
CS	SLC25A5-AS	0.409700269	3.12E-23	postive
GOT1	SLC25A5-AS	0.541195343	2.45E-42	postive
ATG4D	SLC25A5-AS	0.616843629	8.11E-58	postive
GABARAPL2	SLC25A5-AS	0.485367721	3.33E-33	postive
GABARAPL1	SLC25A5-AS	0.676225462	2.75E-73	postive
WIPI2	SLC25A5-AS	0.575089401	8.91E-49	postive
LINC00472	SLC25A5-AS	0.434721451	2.95E-26	postive
BAP1	SLC25A5-AS	0.426397548	3.19E-25	postive
LPIN1	SLC25A5-AS	0.59823496	1.27E-53	postive
SLC1A4	AL158206.1	0.424266884	5.81E-25	postive
BLOC1S5-TXNDC5	AL158206.1	0.419109839	2.43E-24	postive
HMGB1	AL158206.1	0.448604308	4.78E-28	postive
ZEB1	AL158206.1	0.460268778	1.29E-29	postive
MAPK8	AL158206.1	0.410713954	2.38E-23	postive
SRC	AC018653.3	0.424080301	6.12E-25	postive
SETD1B	AC018653.3	0.482191025	9.84E-33	postive
ALOX12	AC018653.3	0.600249502	4.59E-54	postive
PHKG2	AC018653.3	0.527562	5.99E-40	postive
TAZ	AC018653.3	0.549310491	8.19E-44	postive
HELLS	AP001429.1	0.401495643	2.70E-22	postive
KLHL24	AP001429.1	0.533413267	5.83E-41	postive
IREB2	AP001429.1	0.446622134	8.72E-28	postive
GABPB1	AP001429.1	0.521971435	5.33E-39	postive
PIK3CA	AP001429.1	0.480585063	1.70E-32	postive
KRAS	AP001429.1	0.401024719	3.05E-22	postive
MAPK8	AP001429.1	0.40537408	9.81E-23	postive
LINC00472	AP001429.1	0.931111908	2.23E-237	postive
ATM	AP001429.1	0.597472597	1.86E-53	postive
HELLS	LINC01772	0.425723859	3.86E-25	postive
ZNF419	LINC01772	0.582955933	2.22E-50	postive
VEGFA	LINC01772	0.501030265	1.34E-35	postive
TUBE1	LINC01772	0.656710349	8.15E-68	postive
SETD1B	LINC01772	0.52280242	3.86E-39	postive
ALOX12	LINC01772	0.722466618	4.11E-88	postive
GABPB1	LINC01772	0.504625866	3.64E-36	postive
LINC00472	LINC01772	0.409579537	3.22E-23	postive
ATM	LINC01772	0.629785844	6.65E-61	postive

TAZ	LINC01772	0.467199745	1.41E-30	postive
FBXW7	LINC01772	0.519954912	1.16E-38	postive
ZNF419	AL022322.1	0.490884302	4.93E-34	postive
VEGFA	AL022322.1	0.495502972	9.71E-35	postive
TUBE1	AL022322.1	0.566827393	3.87E-47	postive
SETD1B	AL022322.1	0.482454085	9.00E-33	postive
DRD4	AL022322.1	0.426854975	2.81E-25	postive
ALOX12	AL022322.1	0.699164728	2.76E-80	postive
TAZ	AL022322.1	0.565821424	6.09E-47	postive
HELLS	AC015849.3	0.549186378	8.63E-44	postive
ZNF419	AC015849.3	0.540188745	3.70E-42	postive
TUBE1	AC015849.3	0.579810929	9.82E-50	postive
SETD1B	AC015849.3	0.44372527	2.08E-27	postive
ALOX12	AC015849.3	0.704148894	6.78E-82	postive
GABPB1	AC015849.3	0.552862745	1.80E-44	postive
MAPK8	AC015849.3	0.431087936	8.41E-26	postive
LINC00472	AC015849.3	0.549933302	6.29E-44	postive
ATM	AC015849.3	0.665669767	2.82E-70	postive
FBXW7	AC015849.3	0.554016225	1.09E-44	postive
ALOX12	OGFRP1	0.420802926	1.52E-24	postive
ATM	OGFRP1	0.433565518	4.12E-26	postive
HELLS	AL162727.2	0.473957218	1.55E-31	postive
ZNF419	AL162727.2	0.40956927	3.23E-23	postive
TUBE1	AL162727.2	0.464440098	3.43E-30	postive
ALOX12	AL162727.2	0.500518855	1.61E-35	postive
GABPB1	AL162727.2	0.56086514	5.52E-46	postive
LINC00472	AL162727.2	0.539574506	4.77E-42	postive
ATM	AL162727.2	0.685288013	5.65E-76	postive
FBXW7	AL162727.2	0.53513818	2.91E-41	postive
HELLS	AC131391.1	0.479392938	2.53E-32	postive
KLHL24	AC131391.1	0.401479451	2.71E-22	postive
GABPB1	AC131391.1	0.485890712	2.78E-33	postive
ATG7	AC131391.1	0.463398424	4.78E-30	postive
LINC00472	AC131391.1	0.674641565	7.92E-73	postive
ATM	AC131391.1	0.670365425	1.34E-71	postive
FBXW7	AC131391.1	0.422194345	1.04E-24	postive
TFAP2C	AC124947.1	0.575435091	7.59E-49	postive
HBA1	AC124947.1	0.520668615	8.81E-39	postive
IL33	LINC00702	0.513487558	1.36E-37	postive
ZEB1	LINC00702	0.431297694	7.92E-26	postive
HELLS	AC018926.1	0.503346735	5.80E-36	postive
TUBE1	AC018926.1	0.517812196	2.64E-38	postive
ALOX12	AC018926.1	0.468811444	8.37E-31	postive
GABPB1	AC018926.1	0.555867212	4.92E-45	postive
LINC00472	AC018926.1	0.693295371	1.97E-78	postive
ATM	AC018926.1	0.687423705	1.27E-76	postive
FBXW7	AC018926.1	0.497664094	4.50E-35	postive
TAZ	AC010487.1	0.408364947	4.45E-23	postive
FANCD2	AC092112.1	0.48600319	2.68E-33	postive
TFAP2C	AC092112.1	0.502953237	6.69E-36	postive
HSPA5	AC015726.1	-0.40316962	1.75E-22	negative
LAMP2	AC015726.1	-0.424254935	5.83E-25	negative
PHKG2	AC015726.1	0.479328862	2.59E-32	postive
TAZ	AC015726.1	0.566430257	4.63E-47	postive
MTDH	AC015726.1	-0.436472464	1.77E-26	negative
HELLS	A2M-AS1	0.417250542	4.05E-24	postive
GABPB1	A2M-AS1	0.465487166	2.45E-30	postive
ATM	A2M-AS1	0.528418674	4.27E-40	postive

KLHL24	AC008649.2	0.510622987	3.97E-37	postive
IREB2	AC008649.2	0.401552027	2.66E-22	postive
GABPB1	AC008649.2	0.481728375	1.15E-32	postive
PIK3CA	AC008649.2	0.440782079	4.99E-27	postive
LINC00472	AC008649.2	0.919463353	7.35E-220	postive
ATM	AC008649.2	0.538714072	6.78E-42	postive
FANCD2	AC073842.2	0.455172146	6.36E-29	postive
HELLS	AC073842.2	0.464180458	3.72E-30	postive
ZNF419	AC073842.2	0.506473198	1.85E-36	postive
TUBE1	AC073842.2	0.555416195	5.98E-45	postive
SETD1B	AC073842.2	0.450246469	2.90E-28	postive
ALOX12	AC073842.2	0.681166606	9.68E-75	postive
TAZ	AC073842.2	0.50008091	1.89E-35	postive
NOX4	AP003400.1	0.502519464	7.83E-36	postive
TUBE1	AC092953.2	0.405169134	1.03E-22	postive
HELLS	AC010201.1	0.410330865	2.63E-23	postive
ZNF419	AC010201.1	0.540470299	3.30E-42	postive
TUBE1	AC010201.1	0.610063583	2.95E-56	postive
ALOX12	AC010201.1	0.612041233	1.04E-56	postive
GABPB1	AC010201.1	0.452256544	1.57E-28	postive
PHKG2	AC010201.1	0.45744779	3.13E-29	postive
LINC00472	AC010201.1	0.422419949	9.73E-25	postive
ATM	AC010201.1	0.483626103	6.04E-33	postive
TAZ	AC010201.1	0.587137582	2.99E-51	postive
KLHL24	AC023421.1	0.525513883	1.34E-39	postive
LINC00472	AC023421.1	0.754331871	3.25E-100	postive
LPIN1	AC023421.1	0.425804932	3.77E-25	postive
KLHL24	AC084048.1	0.562844801	2.30E-46	postive
PIK3CA	AC084048.1	0.41828713	3.05E-24	postive
LINC00472	AC084048.1	0.840462845	5.17E-145	postive
HELLS	AL157400.4	0.443353864	2.32E-27	postive
ZNF419	AL157400.4	0.434475922	3.17E-26	postive
TUBE1	AL157400.4	0.58897	1.23E-51	postive
ALOX12	AL157400.4	0.590687195	5.32E-52	postive
GABPB1	AL157400.4	0.454533354	7.76E-29	postive
MAPK8	AL157400.4	0.46008913	1.37E-29	postive
ATM	AL157400.4	0.683125128	2.52E-75	postive
FBXW7	AL157400.4	0.443150001	2.47E-27	postive
GPX4	AC040169.1	0.451398731	2.04E-28	postive
RB1	AC040169.1	-0.408661193	4.11E-23	negative
HSPB1	AC040169.1	0.550508021	4.92E-44	postive
OTUB1	AC040169.1	0.407648937	5.38E-23	postive
IREB2	AC040169.1	-0.403829768	1.47E-22	negative
HRAS	AC040169.1	0.63439085	4.89E-62	postive
NCOA4	AC040169.1	-0.403295243	1.69E-22	negative
PHKG2	AC040169.1	0.497707252	4.43E-35	postive
PRKAA1	AC040169.1	-0.421260174	1.34E-24	negative
ANO6	AC040169.1	-0.407386347	5.77E-23	negative
EGLN2	AC040169.1	0.511326931	3.05E-37	postive
SIRT1	AC040169.1	-0.410390904	2.59E-23	negative
FANCD2	LINC01410	0.510026617	4.96E-37	postive
HELLS	LINC01410	0.460476962	1.21E-29	postive
HELLS	SSBP3-AS1	0.402930969	1.86E-22	postive
ZNF419	SSBP3-AS1	0.526357317	9.63E-40	postive
VEGFA	SSBP3-AS1	0.474641092	1.24E-31	postive
TUBE1	SSBP3-AS1	0.563674896	1.59E-46	postive
SETD1B	SSBP3-AS1	0.460150875	1.34E-29	postive
ALOX12	SSBP3-AS1	0.596744705	2.67E-53	postive

GABPB1	SSBP3-AS1	0.472499602	2.51E-31	postive
LINC00472	SSBP3-AS1	0.458339386	2.37E-29	postive
ATM	SSBP3-AS1	0.613665702	4.42E-57	postive
FBXW7	SSBP3-AS1	0.513954273	1.14E-37	postive
BRD4	AL136295.7	0.419472558	2.20E-24	postive
ZNF419	AL136295.7	0.563767955	1.53E-46	postive
VEGFA	AL136295.7	0.460830284	1.08E-29	postive
TUBE1	AL136295.7	0.512905813	1.69E-37	postive
SETD1B	AL136295.7	0.418407255	2.95E-24	postive
DRD4	AL136295.7	0.438541288	9.67E-27	postive
ALOX12	AL136295.7	0.595300746	5.48E-53	postive
NCOA4	AL136295.7	-0.411051634	2.17E-23	negative
PHKG2	AL136295.7	0.530878315	1.61E-40	postive
TAZ	AL136295.7	0.746658077	3.90E-97	postive
HELLS	HCG18	0.550207521	5.60E-44	postive
ARNTL	HCG18	0.400277866	3.70E-22	postive
ZNF419	HCG18	0.426515226	3.09E-25	postive
ZFP69B	HCG18	0.404910205	1.11E-22	postive
TUBE1	HCG18	0.549532168	7.46E-44	postive
ALOX12	HCG18	0.528921562	3.50E-40	postive
IREB2	HCG18	0.442417304	3.07E-27	postive
GABPB1	HCG18	0.63076407	3.84E-61	postive
PIK3CA	HCG18	0.401042247	3.04E-22	postive
ATG7	HCG18	0.422630781	9.18E-25	postive
ZEB1	HCG18	0.409190717	3.57E-23	postive
MAPK8	HCG18	0.528506717	4.13E-40	postive
LINC00472	HCG18	0.596563319	2.92E-53	postive
ATM	HCG18	0.779083514	5.59E-111	postive
FBXW7	HCG18	0.549256256	8.38E-44	postive
KLHL24	AC121493.1	0.471648274	3.32E-31	postive
GABPB1	AC121493.1	0.426688847	2.94E-25	postive
LINC00472	AC121493.1	0.723948889	1.23E-88	postive
DRD4	AC087289.5	0.421106578	1.40E-24	postive
ALOX12	AC087289.5	0.497101295	5.50E-35	postive
PHKG2	AC087289.5	0.555053209	7.00E-45	postive
TAZ	AC087289.5	0.622208874	4.44E-59	postive
ZNF419	AL354733.3	0.58435296	1.14E-50	postive
VEGFA	AL354733.3	0.544926467	5.19E-43	postive
TUBE1	AL354733.3	0.663635577	1.04E-69	postive
SETD1B	AL354733.3	0.506683117	1.71E-36	postive
ALOX12	AL354733.3	0.683913137	1.46E-75	postive
GABPB1	AL354733.3	0.417623561	3.66E-24	postive
ATM	AL354733.3	0.502449811	8.03E-36	postive
YY1AP1	AL354733.3	0.427056922	2.65E-25	postive
TAZ	AL354733.3	0.430692031	9.42E-26	postive
FBXW7	AL354733.3	0.414788878	7.93E-24	postive
BRD4	LINC00174	0.411227274	2.07E-23	postive
ZNF419	LINC00174	0.508234801	9.64E-37	postive
VEGFA	LINC00174	0.414939495	7.61E-24	postive
TUBE1	LINC00174	0.574861051	9.90E-49	postive
SETD1B	LINC00174	0.43182381	6.81E-26	postive
DRD4	LINC00174	0.503693145	5.11E-36	postive
ALOX12	LINC00174	0.632380966	1.54E-61	postive
PHKG2	LINC00174	0.612146606	9.86E-57	postive
TAZ	LINC00174	0.73637735	3.53E-93	postive
FANCD2	AC025171.3	0.443028908	2.56E-27	postive
HELLS	AC025171.3	0.464056775	3.87E-30	postive
ALOX12	AC025171.3	0.414397646	8.82E-24	postive

TFAP2C	AC025171.3	0.403772793	1.49E-22	postive
FBXW7	AC025171.3	0.414647331	8.24E-24	postive
RPL8	CRIM1-DT	0.419006225	2.50E-24	postive
HRAS	CRIM1-DT	0.504015092	4.54E-36	postive
EGLN2	CRIM1-DT	0.401177907	2.93E-22	postive
ZNF419	AC012615.1	0.537993293	9.11E-42	postive
VEGFA	AC012615.1	0.405692024	9.02E-23	postive
TUBE1	AC012615.1	0.499178328	2.62E-35	postive
ALOX12	AC012615.1	0.488209014	1.25E-33	postive
NCOA4	AC012615.1	-0.409258561	3.51E-23	negative
PHKG2	AC012615.1	0.599678328	6.12E-54	postive
TAZ	AC012615.1	0.769131228	1.73E-106	postive
HELLS	LINC01473	0.597432899	1.89E-53	postive
ZNF419	LINC01473	0.400692956	3.32E-22	postive
TUBE1	LINC01473	0.5503673	5.23E-44	postive
ALOX12	LINC01473	0.477941049	4.12E-32	postive
GABPB1	LINC01473	0.604834077	4.44E-55	postive
ATG7	LINC01473	0.419671126	2.08E-24	postive
MAPK8	LINC01473	0.423184014	7.87E-25	postive
LINC00472	LINC01473	0.644530365	1.33E-64	postive
ATM	LINC01473	0.791028431	1.10E-116	postive
FBXW7	LINC01473	0.598015937	1.41E-53	postive
BNIP3	MSC-AS1	0.446443207	9.20E-28	postive
HSD17B11	MSC-AS1	0.460039103	1.39E-29	postive
NRAS	MSC-AS1	0.496373121	7.13E-35	postive
ANO6	MSC-AS1	0.420464293	1.67E-24	postive
MTDH	MSC-AS1	0.482209333	9.78E-33	postive
BACH1	MSC-AS1	0.436117742	1.97E-26	postive
ASNS	MIR193BHG	0.455694074	5.41E-29	postive
TRIB3	MIR193BHG	0.412540624	1.46E-23	postive
AURKA	MIR193BHG	0.400961951	3.10E-22	postive
CARS1	MIR193BHG	0.429746563	1.23E-25	postive
TUBE1	AC244035.1	0.439406396	7.50E-27	postive
FANCD2	AC010973.1	0.408431724	4.37E-23	postive
HELLS	AC010973.1	0.435160786	2.60E-26	postive
ZNF419	AC010973.1	0.490042396	6.62E-34	postive
TUBE1	AC010973.1	0.524364381	2.10E-39	postive
ALOX12	AC010973.1	0.613486938	4.86E-57	postive
GABPB1	AC010973.1	0.40309156	1.78E-22	postive
ATM	AC010973.1	0.49492851	1.19E-34	postive
FBXW7	AC010973.1	0.445838767	1.10E-27	postive
RPL8	HOXC-AS1	0.459114618	1.86E-29	postive
TAZ	AC040160.1	0.410704811	2.38E-23	postive
WIPI1	LINC02038	0.40077033	3.26E-22	postive
ZNF419	AC010809.1	0.435044263	2.69E-26	postive
HELLS	AC073912.1	0.415239609	7.02E-24	postive
ZNF419	AC073912.1	0.424731823	5.10E-25	postive
TUBE1	AC073912.1	0.512009349	2.37E-37	postive
ALOX12	AC073912.1	0.593416337	1.39E-52	postive
GABPB1	AC073912.1	0.470325567	5.11E-31	postive
ATM	AC073912.1	0.613105536	5.94E-57	postive
FBXW7	AC073912.1	0.405003073	1.08E-22	postive
KLHL24	AC068580.1	0.460504443	1.20E-29	postive
LINC00472	AC068580.1	0.461164855	9.72E-30	postive
LPIN1	AC068580.1	0.519984253	1.15E-38	postive
TUBE1	PSORS1C3	0.50607991	2.13E-36	postive
ALOX12	PSORS1C3	0.546986262	2.19E-43	postive
ATM	PSORS1C3	0.49296982	2.38E-34	postive

FANCD2	AC092802.2	0.427201533	2.54E-25	postive
HELLS	AC092802.2	0.496754387	6.22E-35	postive
ALOX12	AC092802.2	0.556212871	4.23E-45	postive
TFAP2C	AC092802.2	0.470979259	4.13E-31	postive
HBA1	AC092802.2	0.40554344	9.38E-23	postive
DUOX1	AC092802.2	0.469291836	7.16E-31	postive
ATM	AC092802.2	0.425402782	4.22E-25	postive
ZFP36	NR4A1AS	0.41342847	1.15E-23	postive
ATF3	NR4A1AS	0.419682741	2.08E-24	postive
VEGFA	NR4A1AS	0.42612406	3.45E-25	postive
PHKG2	LINC02804	0.499082409	2.71E-35	postive
TAZ	LINC02804	0.62641807	4.37E-60	postive
ISCU	AL049870.3	0.563629214	1.62E-46	postive
KLHL24	AL049870.3	0.4056759	9.06E-23	postive
ATG4D	AL049870.3	0.404539422	1.22E-22	postive
GABARAPL1	AL049870.3	0.567402042	2.99E-47	postive
LINC00472	AL049870.3	0.420996	1.45E-24	postive
LPIN1	AL049870.3	0.522873907	3.75E-39	postive
FANCD2	ZNF433-AS1	0.465203039	2.68E-30	postive
HELLS	ZNF433-AS1	0.640414979	1.50E-63	postive
ZNF419	ZNF433-AS1	0.486938255	1.94E-33	postive
TUBE1	ZNF433-AS1	0.502493819	7.90E-36	postive
ALOX12	ZNF433-AS1	0.446663433	8.61E-28	postive
GABPB1	ZNF433-AS1	0.597560058	1.78E-53	postive
ATG7	ZNF433-AS1	0.416840234	4.54E-24	postive
LINC00472	ZNF433-AS1	0.685447114	5.05E-76	postive
ATM	ZNF433-AS1	0.605069394	3.93E-55	postive
FBXW7	ZNF433-AS1	0.503543486	5.40E-36	postive
ALOX12	RPL37A-DT	0.50614584	2.08E-36	postive
ZFP69B	AC092614.1	0.405011086	1.08E-22	postive
GABPB1	AC092614.1	0.554807479	7.78E-45	postive
ZEB1	AC092614.1	0.626393994	4.42E-60	postive
MAPK8	AC092614.1	0.497571441	4.65E-35	postive
LINC00472	AC092614.1	0.478946354	2.94E-32	postive
ATM	AC092614.1	0.584954555	8.54E-51	postive
FBXW7	AC092614.1	0.466855774	1.58E-30	postive
GPX4	AC084036.1	0.459687388	1.55E-29	postive
HSPB1	AC084036.1	0.47707368	5.51E-32	postive
EIF2S1	AC084036.1	-0.45088764	2.38E-28	negative
IREB2	AC084036.1	-0.41911349	2.43E-24	negative
NOX1	AC084036.1	0.46490167	2.95E-30	postive
HRAS	AC084036.1	0.437518561	1.31E-26	postive
NCOA4	AC084036.1	-0.47179294	3.16E-31	negative
PHKG2	AC084036.1	0.577000916	3.66E-49	postive
BECN1	AC084036.1	-0.535061194	3.00E-41	negative
BID	AC084036.1	0.463171622	5.14E-30	postive
SIRT1	AC084036.1	-0.425723279	3.86E-25	negative
HRAS	AC103724.4	0.499201618	2.59E-35	postive
EGLN2	AC103724.4	0.566569769	4.35E-47	postive
HELLS	AL033397.2	0.543298736	1.02E-42	postive
ZNF419	AL033397.2	0.441671254	3.84E-27	postive
TUBE1	AL033397.2	0.538958422	6.14E-42	postive
ALOX12	AL033397.2	0.544332447	6.65E-43	postive
GABPB1	AL033397.2	0.524723352	1.83E-39	postive
MAPK8	AL033397.2	0.421587854	1.23E-24	postive
LINC00472	AL033397.2	0.596369252	3.22E-53	postive
ATM	AL033397.2	0.694970561	5.88E-79	postive
FBXW7	AL033397.2	0.520948395	7.91E-39	postive

HRAS	AC010654.1	0.45517159	6.36E-29	postive
PHKG2	AC010654.1	0.405770031	8.84E-23	postive
EGLN2	AC010654.1	0.544314065	6.70E-43	postive
TAZ	AC010654.1	0.401852684	2.46E-22	postive
GPX4	AC010503.4	0.420881673	1.49E-24	postive
ZFP69B	AC010503.4	-0.452458	1.47E-28	negative
ACSL4	AC010503.4	-0.401322891	2.82E-22	negative
ATG7	AC010503.4	-0.462410567	6.55E-30	negative
PEBP1	AC010503.4	0.640445129	1.48E-63	postive
MIOX	AC010503.4	0.402965532	1.84E-22	postive
MTDH	AC010503.4	-0.42981532	1.21E-25	negative
PANX1	AC010503.4	-0.481882479	1.09E-32	negative
BACH1	AC010503.4	-0.466344148	1.86E-30	negative
EIF2S1	LYRM4-AS1	0.46405128	3.88E-30	postive
MAPK14	LYRM4-AS1	0.43071376	9.36E-26	postive
ZNF419	AC245060.2	0.422497404	9.52E-25	postive
SETD1B	AC245060.2	0.514104632	1.08E-37	postive
ALOX12	AC245060.2	0.495352563	1.02E-34	postive
ZNF419	MRPL20-DT	0.522440918	4.44E-39	postive
TUBE1	MRPL20-DT	0.474057552	1.50E-31	postive
SETD1B	MRPL20-DT	0.479234445	2.67E-32	postive
ALOX12	MRPL20-DT	0.550012654	6.08E-44	postive
PHKG2	MRPL20-DT	0.593865335	1.12E-52	postive
TAZ	MRPL20-DT	0.693523331	1.67E-78	postive
HSF1	PCAT6	0.403147582	1.76E-22	postive
NCOA4	PCAT6	-0.442303981	3.18E-27	negative
PHKG2	PCAT6	0.525715362	1.24E-39	postive
LONP1	PCAT6	0.405773904	8.83E-23	postive
PHKG2	TFAP2A-AS1	0.48282974	7.92E-33	postive
HELLS	AP000919.3	0.495357189	1.02E-34	postive
TUBE1	AP000919.3	0.429168548	1.46E-25	postive
ALOX12	AP000919.3	0.572585229	2.83E-48	postive
GABPB1	AP000919.3	0.409216126	3.55E-23	postive
ATM	AP000919.3	0.569936538	9.49E-48	postive
FBXW7	AP000919.3	0.455595579	5.58E-29	postive
GCH1	AL365361.1	0.487509604	1.59E-33	postive
CYBB	AL365361.1	0.428981943	1.53E-25	postive
IFNG	AL365361.1	0.447415179	6.86E-28	postive
TNFAIP3	AL365361.1	0.413901037	1.01E-23	postive
FBXW7	AL365361.1	0.483998015	5.32E-33	postive
KLHL24	LINC-PINT	0.616486789	9.82E-58	postive
GABPB1	LINC-PINT	0.474201405	1.43E-31	postive
PIK3CA	LINC-PINT	0.455249162	6.21E-29	postive
LINC00472	LINC-PINT	0.925026685	7.15E-228	postive
LPIN1	LINC-PINT	0.481209966	1.37E-32	postive
ATM	LINC-PINT	0.418588858	2.81E-24	postive
ZNF419	AC103691.1	0.417129841	4.19E-24	postive
NCOA4	AC103691.1	-0.406147704	8.00E-23	negative
PHKG2	AC103691.1	0.523440832	3.01E-39	postive
EGLN2	AC103691.1	0.417003287	4.34E-24	postive
TAZ	AC103691.1	0.603734193	7.80E-55	postive
HELLS	AC093227.1	0.417141586	4.18E-24	postive
ZNF419	AC093227.1	0.531670328	1.17E-40	postive
TUBE1	AC093227.1	0.666899029	1.28E-70	postive
ALOX12	AC093227.1	0.46256911	6.22E-30	postive
HMGB1	AC093227.1	0.429253991	1.42E-25	postive
GABPB1	AC093227.1	0.504834737	3.37E-36	postive
MAPK8	AC093227.1	0.437956115	1.15E-26	postive

PRKAA1	AC093227.1	0.406130528	8.04E-23	postive
ATM	AC093227.1	0.509349406	6.38E-37	postive
FBXW7	AC093227.1	0.408705784	4.06E-23	postive
EPAS1	ZNF503-AS1	0.449134308	4.07E-28	postive
VEGFA	AL109615.3	0.457633413	2.95E-29	postive
HELLS	AC138207.4	0.5033394	5.81E-36	postive
ZNF419	AC138207.4	0.487923186	1.38E-33	postive
TUBE1	AC138207.4	0.560064043	7.86E-46	postive
SETD1B	AC138207.4	0.443911279	1.97E-27	postive
ALOX12	AC138207.4	0.652658334	9.92E-67	postive
GABPB1	AC138207.4	0.57044993	7.51E-48	postive
LINC00472	AC138207.4	0.53208194	9.94E-41	postive
ATM	AC138207.4	0.677666988	1.04E-73	postive
FBXW7	AC138207.4	0.549259452	8.37E-44	postive
HELLS	AC131971.1	0.426486985	3.11E-25	postive
KLHL24	AC131971.1	0.537173562	1.27E-41	postive
IREB2	AC131971.1	0.445676695	1.16E-27	postive
GABPB1	AC131971.1	0.538700533	6.82E-42	postive
PIK3CA	AC131971.1	0.483898247	5.51E-33	postive
KRAS	AC131971.1	0.401855046	2.46E-22	postive
ATG7	AC131971.1	0.408150067	4.71E-23	postive
MAPK8	AC131971.1	0.410662351	2.41E-23	postive
LINC00472	AC131971.1	0.927836202	3.71E-232	postive
TGFBR1	AC131971.1	0.405497298	9.49E-23	postive
ATM	AC131971.1	0.625697827	6.51E-60	postive
FBXW7	AC131971.1	0.412843126	1.34E-23	postive
ZNF419	AL513218.1	0.485362232	3.34E-33	postive
TUBE1	AL513218.1	0.463221288	5.06E-30	postive
ALOX12	AL513218.1	0.504879374	3.31E-36	postive
NCOA4	AL513218.1	-0.410265214	2.68E-23	negative
PHKG2	AL513218.1	0.617749455	4.99E-58	postive
TAZ	AL513218.1	0.732625739	8.81E-92	postive
VEGFA	AC025627.1	0.406081908	8.14E-23	postive
TUBE1	AC025627.1	0.505174564	2.97E-36	postive
ALOX12	AC025627.1	0.672874187	2.56E-72	postive
ATM	AC025627.1	0.518596139	1.95E-38	postive
KLHL24	ARRDC3-AS1	0.642286216	5.01E-64	postive
IREB2	ARRDC3-AS1	0.455998512	4.92E-29	postive
GABPB1	ARRDC3-AS1	0.495654033	9.20E-35	postive
PIK3CA	ARRDC3-AS1	0.522331616	4.63E-39	postive
KRAS	ARRDC3-AS1	0.432456161	5.68E-26	postive
ATG7	ARRDC3-AS1	0.427373344	2.42E-25	postive
LINC00472	ARRDC3-AS1	0.899084194	8.61E-195	postive
PRKAA1	ARRDC3-AS1	0.464591799	3.26E-30	postive
TGFBR1	ARRDC3-AS1	0.424355102	5.67E-25	postive
LPIN1	ARRDC3-AS1	0.483711502	5.87E-33	postive
ATM	ARRDC3-AS1	0.561879377	3.53E-46	postive
FBXW7	ARRDC3-AS1	0.438802888	8.95E-27	postive
HNF4A	AC141273.1	0.403668771	1.53E-22	postive
PHKG2	AC027020.2	0.459455787	1.67E-29	postive
TAZ	AC027020.2	0.512523726	1.95E-37	postive
KLHL24	C1QTNF7-AS	0.525171367	1.53E-39	postive
TUBE1	C1QTNF7-AS	0.43384194	3.81E-26	postive
IREB2	C1QTNF7-AS	0.453263841	1.15E-28	postive
GABPB1	C1QTNF7-AS	0.517547403	2.92E-38	postive
PIK3CA	C1QTNF7-AS	0.473008097	2.12E-31	postive
KRAS	C1QTNF7-AS	0.444196208	1.81E-27	postive
MAPK8	C1QTNF7-AS	0.449303558	3.87E-28	postive

LINC00472	C1QTNF7-AS	0.926184243	1.29E-229	postive
PRKAA1	C1QTNF7-AS	0.402895283	1.88E-22	postive
ATM	C1QTNF7-AS	0.598082495	1.37E-53	postive
ZNF419	AC018648.1	0.452446377	1.48E-28	postive
PHKG2	AC018648.1	0.4694581	6.78E-31	postive
TAZ	AC018648.1	0.496776773	6.17E-35	postive
PHKG2	AC020658.5	0.560518533	6.44E-46	postive
TAZ	AC020658.5	0.5582835	1.72E-45	postive
HELLS	LINC02157	0.533733448	5.12E-41	postive
TUBE1	LINC02157	0.475958811	7.99E-32	postive
ALOX12	LINC02157	0.502530862	7.80E-36	postive
GABPB1	LINC02157	0.54522356	4.58E-43	postive
MAPK8	LINC02157	0.443464152	2.25E-27	postive
LINC00472	LINC02157	0.571610353	4.42E-48	postive
ATM	LINC02157	0.757457912	1.67E-101	postive
FBXW7	LINC02157	0.506875094	1.59E-36	postive
HIC1	SENCR	0.486519952	2.24E-33	postive
CDKN2A	SENCR	0.484956204	3.83E-33	postive
SCP2	AC009878.1	0.433859043	3.79E-26	postive
SLC2A6	DBH-AS1	0.412437887	1.50E-23	postive
PHKG2	DBH-AS1	0.430477659	1.00E-25	postive
TAZ	DBH-AS1	0.471823632	3.13E-31	postive
KLHL24	LINC02035	0.443249108	2.40E-27	postive
LINC00472	LINC02035	0.445417071	1.25E-27	postive
TGFBR1	LINC02035	0.427071289	2.64E-25	postive
TFAP2C	AC008514.1	0.523068429	3.48E-39	postive
GCLC	NORAD	0.426273989	3.30E-25	postive
ACSL3	NORAD	0.527093575	7.21E-40	postive
OXSR1	NORAD	0.452174568	1.61E-28	postive
KLHL24	NORAD	0.649734995	5.88E-66	postive
MAP3K5	NORAD	0.42835174	1.84E-25	postive
EIF2AK4	NORAD	0.453288198	1.14E-28	postive
IREB2	NORAD	0.611398362	1.46E-56	postive
PIK3CA	NORAD	0.668294133	5.17E-71	postive
ACSL4	NORAD	0.433242008	4.53E-26	postive
KRAS	NORAD	0.547090397	2.09E-43	postive
SLC38A1	NORAD	0.449523971	3.62E-28	postive
MAPK8	NORAD	0.48903302	9.40E-34	postive
LINC00472	NORAD	0.412572996	1.44E-23	postive
PRKAA2	NORAD	0.499013839	2.78E-35	postive
PRKAA1	NORAD	0.592935821	1.77E-52	postive
MTDH	NORAD	0.414297876	9.06E-24	postive
SIRT1	NORAD	0.431265176	7.99E-26	postive
ZNF419	AC090772.3	0.481798214	1.12E-32	postive
TUBE1	AC090772.3	0.52226601	4.75E-39	postive
ALOX12	AC090772.3	0.436229311	1.90E-26	postive
GABPB1	AC090772.3	0.412857582	1.34E-23	postive
PHKG2	AC090772.3	0.407484656	5.62E-23	postive
LINC00472	AC090772.3	0.489111915	9.15E-34	postive
TAZ	AC090772.3	0.531867807	1.08E-40	postive
HELLS	AC090948.3	0.510395529	4.32E-37	postive
ZNF419	AC090948.3	0.407238874	6.00E-23	postive
TUBE1	AC090948.3	0.493689321	1.84E-34	postive
ALOX12	AC090948.3	0.476480896	6.72E-32	postive
GABPB1	AC090948.3	0.550336066	5.30E-44	postive
LINC00472	AC090948.3	0.541423735	2.23E-42	postive
ATM	AC090948.3	0.628936401	1.07E-60	postive
FBXW7	AC090948.3	0.525842775	1.18E-39	postive

ALOX12	AC080023.1	0.443482939	2.24E-27	postive
HELLS	PSMA3-AS1	0.418152324	3.17E-24	postive
BRD4	PSMA3-AS1	0.428466524	1.78E-25	postive
ZNF419	PSMA3-AS1	0.661644653	3.68E-69	postive
VEGFA	PSMA3-AS1	0.493279156	2.13E-34	postive
TUBE1	PSMA3-AS1	0.699324645	2.45E-80	postive
SETD1B	PSMA3-AS1	0.505617736	2.53E-36	postive
ALOX12	PSMA3-AS1	0.663932852	8.59E-70	postive
GABPB1	PSMA3-AS1	0.604364232	5.65E-55	postive
PRDX1	PSMA3-AS1	-0.408464956	4.33E-23	negative
MAPK8	PSMA3-AS1	0.473466187	1.82E-31	postive
LINC00472	PSMA3-AS1	0.527668496	5.75E-40	postive
ATM	PSMA3-AS1	0.582006915	3.48E-50	postive
TAZ	PSMA3-AS1	0.512263416	2.15E-37	postive
FBXW7	PSMA3-AS1	0.545229666	4.57E-43	postive
BLOC1S5-TXNDC5	AC083964.1	0.413857712	1.02E-23	postive
HMGB1	AC083964.1	0.433916866	3.72E-26	postive
ZEB1	AC083964.1	0.496249015	7.45E-35	postive
EPAS1	AC083964.1	0.526683595	8.47E-40	postive
HELLS	AC068790.4	0.492459174	2.84E-34	postive
TUBE1	AC068790.4	0.460040647	1.39E-29	postive
ALOX12	AC068790.4	0.454698519	7.37E-29	postive
GABPB1	AC068790.4	0.552123024	2.47E-44	postive
MAPK8	AC068790.4	0.429594067	1.29E-25	postive
LINC00472	AC068790.4	0.759476537	2.41E-102	postive
ATM	AC068790.4	0.707071135	7.44E-83	postive
FBXW7	AC068790.4	0.482497778	8.87E-33	postive
TFAP2C	AC124017.1	0.593507452	1.33E-52	postive
HBA1	AC124017.1	0.47964629	2.33E-32	postive
MAPK1	LINC02747	0.475529687	9.21E-32	postive
ALOX12	AC024560.3	0.418929308	2.56E-24	postive
FANCD2	AC010319.4	0.458997498	1.93E-29	postive
HELLS	AC010319.4	0.422300408	1.01E-24	postive
BRD4	AC010319.4	0.439964602	6.36E-27	postive
ZNF419	AC010319.4	0.501393045	1.18E-35	postive
TUBE1	AC010319.4	0.480600876	1.69E-32	postive
ALOX12	AC010319.4	0.647444578	2.34E-65	postive
TAZ	AC010319.4	0.520611119	9.01E-39	postive
ISCU	AL049555.1	0.509922324	5.16E-37	postive
KLHL24	AL049555.1	0.533077332	6.67E-41	postive
GABARAPL1	AL049555.1	0.64236722	4.78E-64	postive
LINC00472	AL049555.1	0.55694028	3.09E-45	postive
LPIN1	AL049555.1	0.616817651	8.23E-58	postive
BRD4	AC087623.1	0.441501027	4.04E-27	postive
TUBE1	AC087623.1	0.411049324	2.17E-23	postive
ALOX12	AC087623.1	0.428875749	1.58E-25	postive
TAZ	AC087623.1	0.472400029	2.59E-31	postive
FANCD2	SLC16A1-AS	0.442449377	3.04E-27	postive
HELLS	SLC16A1-AS	0.546145663	3.11E-43	postive
TUBE1	SLC16A1-AS	0.401458181	2.73E-22	postive
ALOX12	SLC16A1-AS	0.567777513	2.52E-47	postive
ATM	SLC16A1-AS	0.415874465	5.90E-24	postive
FBXW7	SLC16A1-AS	0.439371931	7.57E-27	postive
TFAP2C	AL109741.1	0.410401869	2.59E-23	postive
HBA1	AL109741.1	0.409402767	3.38E-23	postive
EPAS1	AL109741.1	0.408043994	4.84E-23	postive
ZNF419	AC004918.1	0.45972536	1.53E-29	postive
VEGFA	AC004918.1	0.479910671	2.13E-32	postive

TUBE1	AC004918.1	0.501366973	1.19E-35	postive
ALOX12	AC004918.1	0.435222001	2.55E-26	postive
PHKG2	AC004918.1	0.453292103	1.14E-28	postive
TAZ	AC004918.1	0.648127751	1.55E-65	postive
MTOR	AC005670.3	0.530249305	2.07E-40	postive
OXSR1	AC005670.3	0.418872147	2.60E-24	postive
MAFG	AC005670.3	0.415574373	6.41E-24	postive
MAPK14	AC005670.3	0.475780279	8.48E-32	postive
ALOX12	AC005670.3	0.444904717	1.46E-27	postive
IREB2	AC005670.3	0.588317162	1.69E-51	postive
SP1	AC005670.3	0.42201096	1.09E-24	postive
PIK3CA	AC005670.3	0.444351045	1.73E-27	postive
BECN1	AC005670.3	0.441299008	4.28E-27	postive
ULK2	AC005670.3	0.492265537	3.04E-34	postive
MAPK8	AC005670.3	0.482612217	8.53E-33	postive
PRKAA2	AC005670.3	0.468524927	9.18E-31	postive
ATM	AC005670.3	0.546560918	2.62E-43	postive
TFAP2C	AC011477.3	0.540865699	2.80E-42	postive
HBA1	AC011477.3	0.469894705	5.88E-31	postive
OXSR1	EMX2OS	0.43684709	1.59E-26	postive
FTL	EMX2OS	-0.407249217	5.98E-23	negative
MAPK1	EMX2OS	0.41928561	2.32E-24	postive
BID	EMX2OS	-0.488434065	1.16E-33	negative
PEBP1	EMX2OS	0.406401155	7.48E-23	postive
MAPK8	EMX2OS	0.493539177	1.94E-34	postive
PRKAA2	EMX2OS	0.439482445	7.33E-27	postive
SIRT1	EMX2OS	0.436833088	1.59E-26	postive
ALOX12	HOXC-AS2	0.422691616	9.02E-25	postive
TAZ	HOXC-AS2	0.436003455	2.03E-26	postive
HAMP	LINC01111	0.415676962	6.23E-24	postive
KLHL24	AC097504.2	0.515075694	7.46E-38	postive
LINC00472	AC097504.2	0.770926121	2.78E-107	postive
LPIN1	AC097504.2	0.444600345	1.60E-27	postive
HELLS	AC092794.1	0.470225005	5.28E-31	postive
ZNF419	AC092794.1	0.445571055	1.20E-27	postive
KLHL24	AC092794.1	0.412730439	1.38E-23	postive
TUBE1	AC092794.1	0.582414996	2.87E-50	postive
ALOX12	AC092794.1	0.430735867	9.30E-26	postive
GABPB1	AC092794.1	0.565154951	8.21E-47	postive
MAPK8	AC092794.1	0.410602123	2.45E-23	postive
LINC00472	AC092794.1	0.724952788	5.39E-89	postive
ATM	AC092794.1	0.634848484	3.76E-62	postive
FBXW7	AC092794.1	0.455783336	5.26E-29	postive
TFAP2C	LINC02427	0.464869995	2.98E-30	postive
IREB2	AC244517.7	0.412698701	1.40E-23	postive
PIK3CA	AC244517.7	0.405168808	1.03E-22	postive
ATM	AC244517.7	0.414201928	9.30E-24	postive
BACH1	AC244517.7	0.410456849	2.55E-23	postive
PHKG2	AP000781.1	0.45178586	1.81E-28	postive
TAZ	AP000781.1	0.423624886	6.95E-25	postive
TFAP2C	AC092809.2	0.561223214	4.72E-46	postive
HBA1	AC092809.2	0.524326326	2.13E-39	postive
TFAP2C	AC011498.1	0.581396517	4.65E-50	postive
HBA1	AC011498.1	0.521298253	6.91E-39	postive
ZNF419	AC135050.5	0.437392927	1.35E-26	postive
KLHL24	AC135050.5	0.445309881	1.29E-27	postive
TUBE1	AC135050.5	0.473240704	1.96E-31	postive
SETD1B	AC135050.5	0.417478306	3.81E-24	postive

ALOX12	AC135050.5	0.544355116	6.59E-43	postive
IREB2	AC135050.5	0.430310639	1.05E-25	postive
MAPK8	AC135050.5	0.416023777	5.67E-24	postive
LINC00472	AC135050.5	0.642646806	4.05E-64	postive
LPIN1	AC135050.5	0.455870399	5.12E-29	postive
ATM	AC135050.5	0.499386646	2.43E-35	postive
PHKG2	AL596223.2	0.534946603	3.14E-41	postive
TAZ	AL596223.2	0.529274695	3.04E-40	postive
PHKG2	TONSL-AS1	0.453179937	1.18E-28	postive
FANCD2	MCM3AP-AS	0.404273413	1.31E-22	postive
HELLS	MCM3AP-AS	0.564228434	1.24E-46	postive
ZNF419	MCM3AP-AS	0.598444232	1.14E-53	postive
TUBE1	MCM3AP-AS	0.614106289	3.50E-57	postive
SETD1B	MCM3AP-AS	0.436601113	1.71E-26	postive
ALOX12	MCM3AP-AS	0.707317027	6.17E-83	postive
GABPB1	MCM3AP-AS	0.588167341	1.82E-51	postive
MAPK8	MCM3AP-AS	0.459974484	1.42E-29	postive
LINC00472	MCM3AP-AS	0.612029738	1.05E-56	postive
ATM	MCM3AP-AS	0.658276402	3.07E-68	postive
FBXW7	MCM3AP-AS	0.554304515	9.67E-45	postive
HELLS	AL109614.1	0.403623175	1.55E-22	postive
ZNF419	AL109614.1	0.414467231	8.65E-24	postive
KLHL24	AL109614.1	0.562587531	2.58E-46	postive
TUBE1	AL109614.1	0.43493398	2.77E-26	postive
IREB2	AL109614.1	0.486111723	2.58E-33	postive
GABPB1	AL109614.1	0.548231744	1.29E-43	postive
PIK3CA	AL109614.1	0.493863464	1.73E-34	postive
KRAS	AL109614.1	0.43686285	1.58E-26	postive
ATG7	AL109614.1	0.406071902	8.16E-23	postive
MAPK8	AL109614.1	0.454660412	7.46E-29	postive
LINC00472	AL109614.1	0.904946481	2.06E-201	postive
LPIN1	AL109614.1	0.438276109	1.05E-26	postive
ATM	AL109614.1	0.649834272	5.53E-66	postive
FBXW7	AL109614.1	0.459620684	1.58E-29	postive
ALOX12	AC027237.3	0.43264006	5.38E-26	postive
HELLS	AL162274.2	0.416813196	4.57E-24	postive
ZNF419	AL162274.2	0.541184699	2.46E-42	postive
VEGFA	AL162274.2	0.487508903	1.59E-33	postive
TUBE1	AL162274.2	0.554965105	7.27E-45	postive
SETD1B	AL162274.2	0.420452089	1.68E-24	postive
ALOX12	AL162274.2	0.709036364	1.66E-83	postive
ATM	AL162274.2	0.478473132	3.45E-32	postive
TAZ	AL162274.2	0.50960519	5.80E-37	postive
FBXW7	AL162274.2	0.438365571	1.02E-26	postive
TFAP2C	AC011462.3	0.588750231	1.37E-51	postive
HBA1	AC011462.3	0.529915663	2.36E-40	postive
HELLS	GABPB1-AS1	0.515732854	5.82E-38	postive
ZNF419	GABPB1-AS1	0.566884003	3.78E-47	postive
TUBE1	GABPB1-AS1	0.543524366	9.31E-43	postive
ALOX12	GABPB1-AS1	0.658139965	3.34E-68	postive
GABPB1	GABPB1-AS1	0.608618428	6.26E-56	postive
LINC00472	GABPB1-AS1	0.524596631	1.92E-39	postive
ATM	GABPB1-AS1	0.614263552	3.22E-57	postive
FBXW7	GABPB1-AS1	0.487334361	1.69E-33	postive
ZNF419	AL359220.1	0.417024982	4.31E-24	postive
KLHL24	AL359220.1	0.555861202	4.93E-45	postive
IREB2	AL359220.1	0.469429225	6.85E-31	postive
GABPB1	AL359220.1	0.559766739	8.96E-46	postive

PIK3CA	AL359220.1	0.487774681	1.45E-33	postive
KRAS	AL359220.1	0.418134878	3.18E-24	postive
MAPK8	AL359220.1	0.440517795	5.40E-27	postive
LINC00472	AL359220.1	0.857804844	2.39E-157	postive
PRKAA1	AL359220.1	0.416942171	4.41E-24	postive
LPIN1	AL359220.1	0.483317101	6.71E-33	postive
ATM	AL359220.1	0.48844551	1.15E-33	postive
FBXW7	AL359220.1	0.441261841	4.33E-27	postive
HELLS	OPA1-AS1	0.429099707	1.48E-25	postive
KLHL24	OPA1-AS1	0.560096878	7.75E-46	postive
IREB2	OPA1-AS1	0.45224916	1.57E-28	postive
GABPB1	OPA1-AS1	0.540723682	2.97E-42	postive
PIK3CA	OPA1-AS1	0.492436713	2.86E-34	postive
KRAS	OPA1-AS1	0.400136914	3.84E-22	postive
MAPK8	OPA1-AS1	0.418667928	2.75E-24	postive
LINC00472	OPA1-AS1	0.92529283	2.85E-228	postive
ATM	OPA1-AS1	0.582478876	2.78E-50	postive
FBXW7	OPA1-AS1	0.428364305	1.83E-25	postive
HELLS	AC012181.2	0.41239837	1.51E-23	postive
ZNF419	AC012181.2	0.487286403	1.72E-33	postive
KLHL24	AC012181.2	0.475183434	1.03E-31	postive
TUBE1	AC012181.2	0.567798211	2.50E-47	postive
ALOX12	AC012181.2	0.499567665	2.27E-35	postive
IREB2	AC012181.2	0.460464299	1.21E-29	postive
GABPB1	AC012181.2	0.557628052	2.29E-45	postive
PIK3CA	AC012181.2	0.456109923	4.75E-29	postive
ATG7	AC012181.2	0.421894984	1.13E-24	postive
MAPK8	AC012181.2	0.4781854	3.80E-32	postive
LINC00472	AC012181.2	0.631226689	2.95E-61	postive
ATM	AC012181.2	0.716817124	3.84E-86	postive
FBXW7	AC012181.2	0.575827604	6.33E-49	postive
TFAP2C	LINC01220	0.486875158	1.98E-33	postive
FANCD2	AC091057.1	0.765067449	1.01E-104	postive
HELLS	AC091057.1	0.707279945	6.35E-83	postive
PML	AC091057.1	0.408376249	4.44E-23	postive
BRD4	AC091057.1	0.410928488	2.25E-23	postive
ZNF419	AC091057.1	0.431398889	7.69E-26	postive
ZFP69B	AC091057.1	0.468647513	8.83E-31	postive
ALOX12	AC091057.1	0.512011689	2.36E-37	postive
RRM2	AC091057.1	0.519628592	1.31E-38	postive
GABPB1	AC091057.1	0.422044567	1.08E-24	postive
AURKA	AC091057.1	0.454299728	8.34E-29	postive
IFNG	AC091057.1	0.418126733	3.19E-24	postive
ATM	AC091057.1	0.458976491	1.94E-29	postive
FBXW7	AC091057.1	0.402544	2.06E-22	postive
ISCU	COLCA1	0.432066526	6.35E-26	postive
KLHL24	COLCA1	0.582954574	2.22E-50	postive
GABARAPL1	COLCA1	0.547294243	1.92E-43	postive
LINC00472	COLCA1	0.646639487	3.79E-65	postive
LPIN1	COLCA1	0.506412299	1.89E-36	postive
HSPB1	LIPE-AS1	0.488569009	1.10E-33	postive
RPL8	LIPE-AS1	0.408414223	4.39E-23	postive
HBA1	LIPE-AS1	0.765958358	4.18E-105	postive
HRAS	LIPE-AS1	0.712130414	1.52E-84	postive
MAP1LC3A	LIPE-AS1	0.489351299	8.42E-34	postive
EGLN2	LIPE-AS1	0.84002684	1.01E-144	postive
HELLS	AL049552.1	0.55512094	6.80E-45	postive
ZNF419	AL049552.1	0.423950194	6.35E-25	postive

TUBE1	AL049552.1	0.597140286	2.19E-53	postive
ALOX12	AL049552.1	0.550945582	4.09E-44	postive
GABPB1	AL049552.1	0.547940157	1.46E-43	postive
MAPK8	AL049552.1	0.418789856	2.66E-24	postive
LINC00472	AL049552.1	0.607256289	1.27E-55	postive
ATM	AL049552.1	0.751033362	7.06E-99	postive
FBXW7	AL049552.1	0.53691935	1.41E-41	postive
ARRDC3	AC123595.1	0.490006324	6.70E-34	postive
ATM	AC123595.1	0.446265468	9.71E-28	postive
MT3	AC009549.1	0.405128637	1.05E-22	postive
SLC2A6	AC009549.1	0.431011933	8.59E-26	postive
HSPB1	MUC12-AS1	0.42574337	3.84E-25	postive
PHKG2	MUC12-AS1	0.45994377	1.43E-29	postive
ZNF419	PAN3-AS1	0.556741725	3.36E-45	postive
TUBE1	PAN3-AS1	0.560664175	6.04E-46	postive
SETD1B	PAN3-AS1	0.451217017	2.16E-28	postive
ALOX12	PAN3-AS1	0.629790633	6.64E-61	postive
GABPB1	PAN3-AS1	0.540599175	3.13E-42	postive
ATM	PAN3-AS1	0.406694222	6.93E-23	postive
TAZ	PAN3-AS1	0.530655743	1.76E-40	postive
GPX4	LINC02166	0.467033677	1.49E-30	postive
HSPB1	LINC02166	0.409820129	3.02E-23	postive
EIF2S1	LINC02166	-0.474074384	1.49E-31	negative
EIF2AK4	LINC02166	-0.417401754	3.89E-24	negative
PIK3CA	LINC02166	-0.434417967	3.22E-26	negative
PHKG2	LINC02166	0.556979027	3.03E-45	postive
PRKAA1	LINC02166	-0.418392552	2.96E-24	negative
TAZ	LINC02166	0.404877039	1.12E-22	postive
MUC1	LHX1-DT	0.431412476	7.66E-26	postive
ZNF419	AC048341.1	0.511539796	2.82E-37	postive
TUBE1	AC048341.1	0.47614994	7.50E-32	postive
SETD1B	AC048341.1	0.425793271	3.78E-25	postive
ALOX12	AC048341.1	0.516784818	3.90E-38	postive
PHKG2	AC048341.1	0.476016393	7.84E-32	postive
TAZ	AC048341.1	0.563275087	1.90E-46	postive
FBXW7	AC048341.1	0.428379545	1.82E-25	postive
SLC3A2	LINC00885	0.435195208	2.57E-26	postive
ISCU	LINC00885	0.405582784	9.28E-23	postive
CHAC1	LINC00885	0.641237127	9.29E-64	postive
DDIT3	LINC00885	0.534830042	3.29E-41	postive
SLC2A8	LINC00885	0.448919472	4.35E-28	postive
ATG4D	LINC00885	0.505290893	2.85E-36	postive
MAP1LC3A	LINC00885	0.594917964	6.63E-53	postive
GABARAPL1	LINC00885	0.55492717	7.39E-45	postive
WIPI2	LINC00885	0.425154035	4.53E-25	postive
LPIN1	LINC00885	0.41435591	8.92E-24	postive
HELLS	AC004908.2	0.455502794	5.74E-29	postive
ZNF419	AC004908.2	0.506911467	1.57E-36	postive
KLHL24	AC004908.2	0.453480414	1.07E-28	postive
TUBE1	AC004908.2	0.601092776	2.99E-54	postive
ALOX12	AC004908.2	0.540497592	3.26E-42	postive
GABPB1	AC004908.2	0.558455293	1.59E-45	postive
MAPK8	AC004908.2	0.426271939	3.31E-25	postive
LINC00472	AC004908.2	0.750291737	1.40E-98	postive
ATM	AC004908.2	0.589037808	1.19E-51	postive
FBXW7	AC004908.2	0.436631872	1.69E-26	postive
HSF1	AC103706.1	0.505584832	2.56E-36	postive
FANCD2	AC103706.1	0.404256837	1.31E-22	postive

CHAC1	AC103706.1	0.479971582	2.09E-32 postive
CARS1	AC103706.1	0.405025175	1.07E-22 postive
NCOA4	AC103706.1	-0.415481363	6.57E-24 negative
PHKG2	AC103706.1	0.618441228	3.44E-58 postive
TAZ	AC103706.1	0.554120822	1.05E-44 postive
ALB	LINC02637	0.445668777	1.16E-27 postive
TFR2	LINC02637	0.41432565	8.99E-24 postive
RPL8	TRIM52-AS1	0.464357021	3.52E-30 postive
HELLS	AL157871.5	0.512800547	1.76E-37 postive
ZNF419	AL157871.5	0.450343957	2.82E-28 postive
TUBE1	AL157871.5	0.441822018	3.67E-27 postive
ALOX12	AL157871.5	0.589162654	1.12E-51 postive
GABPB1	AL157871.5	0.467124718	1.44E-30 postive
DUOX1	AL157871.5	0.408507399	4.28E-23 postive
LINC00472	AL157871.5	0.492879262	2.45E-34 postive
ATM	AL157871.5	0.550289703	5.40E-44 postive
FBXW7	AL157871.5	0.484179751	5.00E-33 postive
HELLS	AL031670.1	0.491245624	4.35E-34 postive
BRD4	AL031670.1	0.422709438	8.98E-25 postive
ZNF419	AL031670.1	0.602341362	1.59E-54 postive
VEGFA	AL031670.1	0.445018992	1.41E-27 postive
TUBE1	AL031670.1	0.598115134	1.34E-53 postive
SETD1B	AL031670.1	0.516728821	3.98E-38 postive
ALOX12	AL031670.1	0.630474579	4.52E-61 postive
GABPB1	AL031670.1	0.565705641	6.41E-47 postive
MAPK8	AL031670.1	0.41529309	6.91E-24 postive
LINC00472	AL031670.1	0.511295455	3.09E-37 postive
ATM	AL031670.1	0.609275042	4.45E-56 postive
YY1AP1	AL031670.1	0.418435484	2.93E-24 postive
FBXW7	AL031670.1	0.507243299	1.39E-36 postive
BRD4	AC114730.3	0.437320633	1.38E-26 postive
ZNF419	AC114730.3	0.515869832	5.52E-38 postive
VEGFA	AC114730.3	0.565340411	7.56E-47 postive
TUBE1	AC114730.3	0.534027295	4.55E-41 postive
SETD1B	AC114730.3	0.462616442	6.13E-30 postive
DRD4	AC114730.3	0.47759693	4.63E-32 postive
ALOX12	AC114730.3	0.628379766	1.46E-60 postive
PHKG2	AC114730.3	0.528766954	3.72E-40 postive
TAZ	AC114730.3	0.739557602	2.21E-94 postive
HELLS	AC090510.2	0.499373775	2.44E-35 postive
ZNF419	AC090510.2	0.593653519	1.24E-52 postive
TUBE1	AC090510.2	0.587223753	2.87E-51 postive
ALOX12	AC090510.2	0.646235694	4.82E-65 postive
GABPB1	AC090510.2	0.55921459	1.14E-45 postive
LINC00472	AC090510.2	0.524057266	2.37E-39 postive
ATM	AC090510.2	0.563941949	1.41E-46 postive
TAZ	AC090510.2	0.403988175	1.41E-22 postive
FBXW7	AC090510.2	0.502196333	8.81E-36 postive
CHMP5	AL162171.1	0.43054033	9.84E-26 postive
KLHL24	AL162171.1	0.496157717	7.70E-35 postive
IREB2	AL162171.1	0.445872237	1.09E-27 postive
GABARAPL2	AL162171.1	0.406058597	8.19E-23 postive
GABARAPL1	AL162171.1	0.462381166	6.61E-30 postive
LPIN1	AL162171.1	0.43458181	3.07E-26 postive
ISCU	AC007342.4	0.55274275	1.89E-44 postive
ACSL3	AC007342.4	0.406299451	7.69E-23 postive
KLHL24	AC007342.4	0.533282055	6.14E-41 postive
CS	AC007342.4	0.413089342	1.26E-23 postive

ATG4D	AC007342.4	0.42394321	6.36E-25	postive
GABARAPL1	AC007342.4	0.617863874	4.69E-58	postive
LINC00472	AC007342.4	0.491906888	3.45E-34	postive
LPIN1	AC007342.4	0.576782856	4.06E-49	postive
HELLS	AC107884.1	0.416398994	5.12E-24	postive
ZNF419	AC107884.1	0.401312166	2.83E-22	postive
TUBE1	AC107884.1	0.550887027	4.19E-44	postive
ALOX12	AC107884.1	0.623563677	2.11E-59	postive
IREB2	AC107884.1	0.411481292	1.94E-23	postive
GABPB1	AC107884.1	0.43804755	1.12E-26	postive
MAPK8	AC107884.1	0.422881487	8.56E-25	postive
LINC00472	AC107884.1	0.52500721	1.63E-39	postive
ATM	AC107884.1	0.705484648	2.48E-82	postive
FBXW7	AC107884.1	0.417700901	3.58E-24	postive
TFAP2C	AC010422.2	0.523760319	2.66E-39	postive
HBA1	AC010422.2	0.474361711	1.36E-31	postive
HELLS	ZNF337-AS1	0.484200351	4.97E-33	postive
TUBE1	ZNF337-AS1	0.45894037	1.96E-29	postive
ALOX12	ZNF337-AS1	0.554545119	8.72E-45	postive
IREB2	ZNF337-AS1	0.404712174	1.17E-22	postive
ULK2	ZNF337-AS1	0.408502163	4.29E-23	postive
MAPK8	ZNF337-AS1	0.420317785	1.74E-24	postive
LINC00472	ZNF337-AS1	0.425166704	4.51E-25	postive
ATM	ZNF337-AS1	0.656939982	7.06E-68	postive
FANCD2	AC243960.1	0.449682192	3.45E-28	postive
PML	AC243960.1	0.401299889	2.84E-22	postive
SLC2A6	AC243960.1	0.424766102	5.05E-25	postive
IFNG	AC243960.1	0.589880251	7.90E-52	postive
FBXW7	AC243960.1	0.418681286	2.74E-24	postive
HELLS	LINC01767	0.480962891	1.49E-32	postive
TUBE1	LINC01767	0.407465396	5.65E-23	postive
GABPB1	LINC01767	0.476133406	7.54E-32	postive
LINC00472	LINC01767	0.515178107	7.18E-38	postive
ATM	LINC01767	0.650439513	3.83E-66	postive
FBXW7	LINC01767	0.475014074	1.09E-31	postive
VEGFA	AC006449.2	0.444060306	1.88E-27	postive
TUBE1	AC006449.2	0.459234905	1.79E-29	postive
ALOX12	AC006449.2	0.456312792	4.46E-29	postive
GABPB1	AC006449.2	0.449137533	4.07E-28	postive
ZEB1	AC006449.2	0.419051898	2.47E-24	postive
MAPK8	AC006449.2	0.403245045	1.71E-22	postive
ATM	AC006449.2	0.468828686	8.32E-31	postive
ANGPTL7	LINC00205	0.419856417	1.98E-24	postive
ZNF419	LINC00205	0.534802743	3.33E-41	postive
TFAP2C	LINC02544	0.481527733	1.23E-32	postive
HBA1	LINC02544	0.416373596	5.15E-24	postive
FANCD2	AP4B1-AS1	0.491321471	4.23E-34	postive
HELLS	AP4B1-AS1	0.566556488	4.38E-47	postive
BRD4	AP4B1-AS1	0.403411907	1.64E-22	postive
ZNF419	AP4B1-AS1	0.585560415	6.38E-51	postive
TUBE1	AP4B1-AS1	0.581006204	5.59E-50	postive
SETD1B	AP4B1-AS1	0.41334091	1.17E-23	postive
ALOX12	AP4B1-AS1	0.691047341	9.82E-78	postive
GABPB1	AP4B1-AS1	0.546406749	2.79E-43	postive
LINC00472	AP4B1-AS1	0.418500954	2.88E-24	postive
ATM	AP4B1-AS1	0.590375286	6.20E-52	postive
FBXW7	AP4B1-AS1	0.56244464	2.75E-46	postive
HELLS	AC078846.1	0.485539926	3.14E-33	postive

ZNF419	AC078846.1	0.435465249	2.38E-26 postive
TUBE1	AC078846.1	0.559737778	9.08E-46 postive
ALOX12	AC078846.1	0.611076237	1.73E-56 postive
GABPB1	AC078846.1	0.51742341	3.06E-38 postive
LINC00472	AC078846.1	0.541499445	2.16E-42 postive
ATM	AC078846.1	0.644601253	1.28E-64 postive
FBXW7	AC078846.1	0.447754583	6.19E-28 postive
FANCD2	ATP2A1-AS1	0.52205935	5.15E-39 postive
ASNS	ATP2A1-AS1	0.401620338	2.61E-22 postive
PSAT1	ATP2A1-AS1	0.425904271	3.67E-25 postive
SLC2A6	ATP2A1-AS1	0.428006943	2.02E-25 postive
STMN1	ATP2A1-AS1	0.502103518	9.11E-36 postive
RRM2	ATP2A1-AS1	0.567467056	2.90E-47 postive
AURKA	ATP2A1-AS1	0.632363135	1.55E-61 postive
SLC1A5	ATP2A1-AS1	0.442830947	2.72E-27 postive
CDKN2A	ATP2A1-AS1	0.506015829	2.19E-36 postive
TFAP2C	AP001372.1	0.528348947	4.39E-40 postive
HBA1	AP001372.1	0.456556071	4.14E-29 postive
EGLN2	AC018529.1	0.493170673	2.21E-34 postive
TFAP2C	AC131097.3	0.546155554	3.10E-43 postive
HBA1	AC131097.3	0.51674184	3.96E-38 postive
HELLS	AL359697.1	0.464554036	3.30E-30 postive
TFAP2C	AL359697.1	0.470000518	5.68E-31 postive
HBA1	AL359697.1	0.436321497	1.85E-26 postive
DUOX1	AL359697.1	0.428075071	1.99E-25 postive
ZNF419	AP001021.2	0.417243418	4.06E-24 postive
KLHL24	AP001021.2	0.492447496	2.85E-34 postive
TUBE1	AP001021.2	0.425059048	4.65E-25 postive
GABPB1	AP001021.2	0.419273774	2.33E-24 postive
LINC00472	AP001021.2	0.747800591	1.38E-97 postive
ATM	AP001021.2	0.50415993	4.31E-36 postive
BRD4	AL022238.2	0.4755919	9.03E-32 postive
ZNF419	AL022238.2	0.488202471	1.25E-33 postive
VEGFA	AL022238.2	0.441228273	4.38E-27 postive
TUBE1	AL022238.2	0.443936168	1.95E-27 postive
SETD1B	AL022238.2	0.419097105	2.44E-24 postive
ALOX12	AL022238.2	0.581151624	5.22E-50 postive
GABPB1	AL022238.2	0.403907596	1.44E-22 postive
ATM	AL022238.2	0.436285812	1.87E-26 postive
TAZ	AL022238.2	0.40365726	1.54E-22 postive
FBXW7	AL022238.2	0.436800618	1.61E-26 postive
FANCD2	AP000763.3	0.425168988	4.51E-25 postive
HELLS	AP000763.3	0.465252957	2.64E-30 postive
ALOX12	AP000763.3	0.472810504	2.26E-31 postive
TFAP2C	AP000763.3	0.431474264	7.53E-26 postive
DUOX1	AP000763.3	0.421533579	1.25E-24 postive
LINC00472	AP000763.3	0.413047348	1.27E-23 postive
FBXW7	AP000763.3	0.426869907	2.79E-25 postive
IL33	MBNL1-AS1	0.510110469	4.81E-37 postive
GABPB1	MBNL1-AS1	0.440397434	5.60E-27 postive
ZEB1	MBNL1-AS1	0.441234697	4.37E-27 postive
FANCD2	AC022126.1	0.451515259	1.97E-28 postive
GCH1	AC022126.1	0.432187671	6.13E-26 postive
STMN1	AC022126.1	0.401193265	2.92E-22 postive
RRM2	AC022126.1	0.526328277	9.74E-40 postive
AURKA	AC022126.1	0.45885414	2.01E-29 postive
IFNG	AC022126.1	0.554817708	7.75E-45 postive
HELLS	AL591848.3	0.417087169	4.24E-24 postive

GABPB1	AL591848.3	0.488487935	1.14E-33	postive
MAPK8	AL591848.3	0.437996611	1.13E-26	postive
LINC00472	AL591848.3	0.486703139	2.10E-33	postive
ATM	AL591848.3	0.595520765	4.91E-53	postive
BRD4	AC145285.6	0.405167269	1.04E-22	postive
ZNF419	AC145285.6	0.457933494	2.69E-29	postive
TUBE1	AC145285.6	0.406019656	8.27E-23	postive
DRD4	AC145285.6	0.421885097	1.13E-24	postive
ALOX12	AC145285.6	0.518341238	2.15E-38	postive
PHKG2	AC145285.6	0.603757492	7.71E-55	postive
EGLN2	AC145285.6	0.432929635	4.95E-26	postive
TAZ	AC145285.6	0.688923785	4.42E-77	postive
ISCU	LINC01187	0.653108888	7.52E-67	postive
ACSL3	LINC01187	0.426356388	3.23E-25	postive
DDIT3	LINC01187	0.445680694	1.16E-27	postive
KLHL24	LINC01187	0.506679659	1.71E-36	postive
ATG4D	LINC01187	0.512831063	1.74E-37	postive
GABARAPL1	LINC01187	0.727044048	9.58E-90	postive
LINC00472	LINC01187	0.402825705	1.91E-22	postive
BAP1	LINC01187	0.410197088	2.73E-23	postive
LPIN1	LINC01187	0.58811811	1.86E-51	postive
FANCD2	BET1-AS1	0.404931704	1.10E-22	postive
HELLS	BET1-AS1	0.547992647	1.43E-43	postive
TUBE1	BET1-AS1	0.453271266	1.15E-28	postive
ALOX12	BET1-AS1	0.494283059	1.50E-34	postive
GABPB1	BET1-AS1	0.425272896	4.38E-25	postive
ATM	BET1-AS1	0.50980963	5.38E-37	postive
FBXW7	BET1-AS1	0.462312016	6.75E-30	postive
HELLS	DLGAP4-AS1	0.57338474	1.96E-48	postive
ZNF419	DLGAP4-AS1	0.498573693	3.25E-35	postive
TUBE1	DLGAP4-AS1	0.555552907	5.64E-45	postive
ALOX12	DLGAP4-AS1	0.581480014	4.47E-50	postive
GABPB1	DLGAP4-AS1	0.554755695	7.96E-45	postive
LINC00472	DLGAP4-AS1	0.572276233	3.26E-48	postive
ATM	DLGAP4-AS1	0.649233101	7.96E-66	postive
FBXW7	DLGAP4-AS1	0.539270372	5.40E-42	postive
KLHL24	AL161804.1	0.455204432	6.30E-29	postive
HELLS	AC012557.1	0.4844967	4.49E-33	postive
KLHL24	AC012557.1	0.508804218	7.81E-37	postive
TUBE1	AC012557.1	0.439970069	6.35E-27	postive
IREB2	AC012557.1	0.448233084	5.35E-28	postive
GABPB1	AC012557.1	0.586626598	3.82E-51	postive
PIK3CA	AC012557.1	0.465260794	2.63E-30	postive
ATG7	AC012557.1	0.436478244	1.77E-26	postive
MAPK8	AC012557.1	0.430012469	1.14E-25	postive
LINC00472	AC012557.1	0.861062314	7.58E-160	postive
TGFBR1	AC012557.1	0.400918315	3.14E-22	postive
ATM	AC012557.1	0.673048908	2.28E-72	postive
FBXW7	AC012557.1	0.446197855	9.91E-28	postive
KLHL24	PTPRJ-AS1	0.521525358	6.33E-39	postive
GABPB1	PTPRJ-AS1	0.43406868	3.56E-26	postive
PIK3CA	PTPRJ-AS1	0.423687647	6.83E-25	postive
LINC00472	PTPRJ-AS1	0.874666254	5.08E-171	postive
HELLS	AC040169.3	0.460814957	1.09E-29	postive
KLHL24	AC040169.3	0.436107161	1.97E-26	postive
GABPB1	AC040169.3	0.458409304	2.32E-29	postive
LINC00472	AC040169.3	0.792441773	2.18E-117	postive
LPIN1	AC040169.3	0.469391482	6.93E-31	postive

ATM	AC040169.3	0.422490138	9.54E-25 postive
FBXW7	AC040169.3	0.410038702	2.85E-23 postive
FANCD2	LINC01943	0.404384551	1.27E-22 postive
PML	LINC01943	0.459831442	1.48E-29 postive
SLC2A6	LINC01943	0.546556978	2.62E-43 postive
IFNG	LINC01943	0.49817107	3.75E-35 postive
ZNF419	AL117379.1	0.551321275	3.48E-44 postive
TUBE1	AL117379.1	0.492622767	2.68E-34 postive
ALOX12	AL117379.1	0.503437415	5.61E-36 postive
GABPB1	AL117379.1	0.423832235	6.56E-25 postive
NCOA4	AL117379.1	-0.403803429	1.48E-22 negative
PHKG2	AL117379.1	0.597742386	1.62E-53 postive
TAZ	AL117379.1	0.70116058	6.31E-81 postive
ENPP2	LINC01235	0.56707533	3.47E-47 postive
BLOC1S5-TXNDC5	LINC01235	0.431731556	6.99E-26 postive
PHKG2	AC091153.3	0.406697653	6.92E-23 postive
TAZ	AC091153.3	0.417867015	3.42E-24 postive
ALOX12	AC011005.4	0.520686705	8.75E-39 postive
TAZ	AC011005.4	0.449658547	3.47E-28 postive
RB1	AC104083.1	0.457195735	3.39E-29 postive
IL33	AC104083.1	0.520437305	9.63E-39 postive
HMGB1	AC104083.1	0.521940262	5.39E-39 postive
ZEB1	AC104083.1	0.606951298	1.49E-55 postive
PRKAA1	AC104083.1	0.402589197	2.03E-22 postive
EPAS1	AC104083.1	0.497180721	5.35E-35 postive
SIRT1	AC104083.1	0.443158742	2.46E-27 postive
RB1	CASC2	0.52589825	1.15E-39 postive
HELLS	CASC2	0.400038849	3.94E-22 postive
SP1	CASC2	0.431703959	7.05E-26 postive
PRKAA2	CASC2	0.529763722	2.51E-40 postive
ATM	CASC2	0.547566529	1.71E-43 postive
HELLS	AC063965.2	0.600768663	3.53E-54 postive
TUBE1	AC063965.2	0.41363987	1.08E-23 postive
ALOX12	AC063965.2	0.48967157	7.53E-34 postive
GABPB1	AC063965.2	0.50133362	1.20E-35 postive
DUOX1	AC063965.2	0.410535619	2.49E-23 postive
LINC00472	AC063965.2	0.528906734	3.52E-40 postive
ATM	AC063965.2	0.609912677	3.19E-56 postive
FBXW7	AC063965.2	0.465650661	2.32E-30 postive
ANGPTL7	LINC01546	0.598180941	1.30E-53 postive
HELLS	AC005332.3	0.50318914	6.14E-36 postive
TUBE1	AC005332.3	0.602528823	1.44E-54 postive
ALOX12	AC005332.3	0.511979205	2.39E-37 postive
GABPB1	AC005332.3	0.50751516	1.26E-36 postive
LINC00472	AC005332.3	0.433899578	3.74E-26 postive
ATM	AC005332.3	0.575349353	7.90E-49 postive
FBXW7	AC005332.3	0.481742486	1.15E-32 postive
ZNF419	AC005840.2	0.455043298	6.62E-29 postive
TUBE1	AC005840.2	0.438217474	1.06E-26 postive
ALOX12	AC005840.2	0.471412541	3.58E-31 postive
PHKG2	AC005840.2	0.463732968	4.29E-30 postive
TAZ	AC005840.2	0.630104521	5.56E-61 postive
HELLS	AC090912.2	0.522940824	3.66E-39 postive
GABPB1	AC090912.2	0.453031115	1.23E-28 postive
LINC00472	AC090912.2	0.624404117	1.33E-59 postive
ATM	AC090912.2	0.589578145	9.15E-52 postive
FBXW7	AC090912.2	0.443429021	2.27E-27 postive
HELLS	AL450384.1	0.400956649	3.11E-22 postive

KLHL24	AL450384.1	0.517944592	2.51E-38	postive
TUBE1	AL450384.1	0.41782671	3.46E-24	postive
GABPB1	AL450384.1	0.524214963	2.23E-39	postive
PIK3CA	AL450384.1	0.414825092	7.85E-24	postive
LINC00472	AL450384.1	0.88365903	3.78E-179	postive
ATM	AL450384.1	0.557763621	2.15E-45	postive
FBXW7	AL450384.1	0.410067749	2.83E-23	postive
TFAP2C	AC105020.5	0.547234425	1.97E-43	postive
HBA1	AC105020.5	0.495827743	8.65E-35	postive
DUOX1	AC105020.5	0.404662229	1.18E-22	postive
ZNF419	AL158212.2	0.476778843	6.08E-32	postive
TUBE1	AL158212.2	0.412481642	1.48E-23	postive
ALOX12	AL158212.2	0.471309522	3.71E-31	postive
PHKG2	AL158212.2	0.569759802	1.03E-47	postive
TAZ	AL158212.2	0.695184925	5.04E-79	postive
HELLS	AC010261.2	0.439760768	6.75E-27	postive
KLHL24	AC010261.2	0.519541573	1.36E-38	postive
TUBE1	AC010261.2	0.408084735	4.79E-23	postive
IREB2	AC010261.2	0.442903906	2.66E-27	postive
GABPB1	AC010261.2	0.54411089	7.30E-43	postive
PIK3CA	AC010261.2	0.465349585	2.56E-30	postive
MAPK8	AC010261.2	0.423204292	7.82E-25	postive
LINC00472	AC010261.2	0.913370868	1.00E-211	postive
ATM	AC010261.2	0.651766993	1.71E-66	postive
FBXW7	AC010261.2	0.417661275	3.62E-24	postive
TUBE1	AC135803.1	0.456879371	3.74E-29	postive
NOX1	AC135803.1	0.52684607	7.95E-40	postive
BECN1	AC135803.1	-0.400977553	3.09E-22	negative
TAZ	AC135803.1	0.443766482	2.06E-27	postive
CXCL2	AC104695.3	0.402272209	2.21E-22	postive
LINC00472	AC104695.3	0.552251751	2.34E-44	postive
EGLN2	AC026740.1	0.468405599	9.55E-31	postive
TAZ	AC026740.1	0.400836476	3.20E-22	postive
ATG4D	AL359513.1	0.498868027	2.92E-35	postive
TUBE1	AC067945.2	0.452849976	1.30E-28	postive
ALOX12	AC067945.2	0.487387163	1.66E-33	postive
GABPB1	AC067945.2	0.423792189	6.64E-25	postive
ATM	AC067945.2	0.456078967	4.80E-29	postive
TAZ	AC067945.2	0.418244562	3.09E-24	postive
FBXW7	AC067945.2	0.472812146	2.26E-31	postive
ALOX12	AC010359.2	0.438340292	1.03E-26	postive
PHKG2	AC010359.2	0.506293954	1.97E-36	postive
TAZ	AC010359.2	0.524033565	2.39E-39	postive
LINC00472	AC012467.2	0.487374739	1.67E-33	postive
HELLS	AC114980.1	0.53997535	4.04E-42	postive
TUBE1	AC114980.1	0.404271074	1.31E-22	postive
ALOX12	AC114980.1	0.479480933	2.46E-32	postive
TFAP2C	AC114980.1	0.423380213	7.45E-25	postive
HBA1	AC114980.1	0.421880189	1.13E-24	postive
GABPB1	AC114980.1	0.484981403	3.80E-33	postive
DUOX1	AC114980.1	0.436014916	2.02E-26	postive
LINC00472	AC114980.1	0.458558102	2.21E-29	postive
ATM	AC114980.1	0.480604929	1.68E-32	postive
FBXW7	AC114980.1	0.45224658	1.57E-28	postive
MTOR	AL158212.3	0.481172256	1.39E-32	postive
IREB2	AL158212.3	0.460789007	1.09E-29	postive
GABPB1	AL158212.3	0.418077809	3.23E-24	postive
PIK3CA	AL158212.3	0.406305809	7.67E-23	postive

MAPK8	AL158212.3	0.469523587	6.64E-31	postive
LINC00472	AL158212.3	0.431663623	7.13E-26	postive
ATM	AL158212.3	0.540000388	4.00E-42	postive
HELLS	AC144548.1	0.440092704	6.12E-27	postive
BRD4	AC144548.1	0.409342919	3.43E-23	postive
ZNF419	AC144548.1	0.520555582	9.20E-39	postive
TUBE1	AC144548.1	0.600497794	4.05E-54	postive
SETD1B	AC144548.1	0.537122902	1.30E-41	postive
ALOX12	AC144548.1	0.593250739	1.51E-52	postive
GABPB1	AC144548.1	0.450325203	2.83E-28	postive
LINC00472	AC144548.1	0.505666192	2.48E-36	postive
ATM	AC144548.1	0.520467803	9.52E-39	postive
TAZ	AC144548.1	0.407201496	6.06E-23	postive
FBXW7	AC144548.1	0.435061673	2.67E-26	postive
KLHL24	AC068580.3	0.43667832	1.67E-26	postive
GABARAPL1	AC068580.3	0.469035622	7.78E-31	postive
LPIN1	AC068580.3	0.548011238	1.42E-43	postive
BLOC1S5-TXNDC5	AC025175.1	0.430387611	1.03E-25	postive
IL33	AC025175.1	0.504870575	3.32E-36	postive
ZEB1	AC025175.1	0.482185756	9.86E-33	postive
HELLS	AP000941.1	0.508263349	9.54E-37	postive
ZNF419	AP000941.1	0.460855112	1.07E-29	postive
ZFP69B	AP000941.1	0.415763056	6.08E-24	postive
TUBE1	AP000941.1	0.549710334	6.91E-44	postive
SETD1B	AP000941.1	0.419376229	2.26E-24	postive
ALOX12	AP000941.1	0.569744445	1.04E-47	postive
GABPB1	AP000941.1	0.560484246	6.53E-46	postive
ZEB1	AP000941.1	0.413690037	1.07E-23	postive
MAPK8	AP000941.1	0.458148563	2.51E-29	postive
LINC00472	AP000941.1	0.513221859	1.50E-37	postive
ATM	AP000941.1	0.687562561	1.15E-76	postive
FBXW7	AP000941.1	0.524189691	2.25E-39	postive
ATF4	TMEM44-AS	0.411620448	1.87E-23	postive
ALOX12	TMEM44-AS	0.460435095	1.22E-29	postive
PHKG2	TMEM44-AS	0.429055516	1.50E-25	postive
TAZ	TMEM44-AS	0.566426658	4.64E-47	postive
HELLS	AP001432.1	0.563726867	1.55E-46	postive
ZNF419	AP001432.1	0.46448562	3.38E-30	postive
ZFP69B	AP001432.1	0.456909289	3.70E-29	postive
TUBE1	AP001432.1	0.48958866	7.75E-34	postive
ALOX12	AP001432.1	0.553941755	1.13E-44	postive
GABPB1	AP001432.1	0.51733558	3.16E-38	postive
ATG7	AP001432.1	0.428465985	1.78E-25	postive
MAPK8	AP001432.1	0.408808184	3.95E-23	postive
ATM	AP001432.1	0.779360761	4.16E-111	postive
FBXW7	AP001432.1	0.544438642	6.36E-43	postive
TUBE1	LINC00622	0.413977391	9.88E-24	postive
HELLS	AL049840.2	0.483864415	5.57E-33	postive
ZNF419	AL049840.2	0.539249401	5.45E-42	postive
KLHL24	AL049840.2	0.46425992	3.63E-30	postive
TUBE1	AL049840.2	0.549993409	6.13E-44	postive
SETD1B	AL049840.2	0.406425535	7.43E-23	postive
ALOX12	AL049840.2	0.55911422	1.19E-45	postive
IREB2	AL049840.2	0.45647567	4.24E-29	postive
GABPB1	AL049840.2	0.609313918	4.36E-56	postive
PIK3CA	AL049840.2	0.442288865	3.19E-27	postive
MAPK8	AL049840.2	0.511735196	2.62E-37	postive
LINC00472	AL049840.2	0.789790122	4.45E-116	postive

LPIN1	AL049840.2	0.406485755	7.32E-23	postive
ATM	AL049840.2	0.705867961	1.85E-82	postive
FBXW7	AL049840.2	0.547388095	1.85E-43	postive
TFAP2C	AC002550.2	0.563257426	1.91E-46	postive
HBA1	AC002550.2	0.509419889	6.22E-37	postive
DUOX1	AC002550.2	0.434267655	3.36E-26	postive
ZNF419	AP006621.4	0.462380168	6.61E-30	postive
VEGFA	AP006621.4	0.466589464	1.72E-30	postive
TUBE1	AP006621.4	0.522356819	4.59E-39	postive
SETD1B	AP006621.4	0.444537299	1.63E-27	postive
ALOX12	AP006621.4	0.638595339	4.34E-63	postive
TAZ	AP006621.4	0.463186457	5.11E-30	postive
BECN1	SNHG16	0.409845343	3.00E-23	postive
MTDH	SNHG16	0.404046927	1.39E-22	postive
BACH1	SNHG16	0.407808767	5.16E-23	postive
HELLS	EPS15-AS1	0.430631273	9.58E-26	postive
TUBE1	EPS15-AS1	0.444217323	1.80E-27	postive
ALOX12	EPS15-AS1	0.526398813	9.47E-40	postive
GABPB1	EPS15-AS1	0.424084401	6.11E-25	postive
LINC00472	EPS15-AS1	0.437341153	1.37E-26	postive
ATM	EPS15-AS1	0.571783254	4.08E-48	postive
FBXW7	EPS15-AS1	0.408988765	3.77E-23	postive
LURAP1L	NR2F1-AS1	0.44926603	3.91E-28	postive
IL33	NR2F1-AS1	0.426379388	3.21E-25	postive
GABPB1	NR2F1-AS1	0.452928727	1.27E-28	postive
ZEB1	NR2F1-AS1	0.565580681	6.78E-47	postive
EPAS1	NR2F1-AS1	0.534856521	3.26E-41	postive
ALOX12	SLC6A1-AS1	0.470182853	5.35E-31	postive
GABPB1	SLC6A1-AS1	0.466427045	1.81E-30	postive
ZEB1	SLC6A1-AS1	0.521384569	6.68E-39	postive
MAPK8	SLC6A1-AS1	0.424930037	4.82E-25	postive
LINC00472	SLC6A1-AS1	0.404239737	1.32E-22	postive
ATM	SLC6A1-AS1	0.594906576	6.67E-53	postive
FBXW7	SLC6A1-AS1	0.440981106	4.71E-27	postive
ACSL3	AL606834.1	0.43433933	3.29E-26	postive
DDIT3	AL606834.1	0.406380381	7.52E-23	postive
KLHL24	AL606834.1	0.550707043	4.52E-44	postive
KRAS	AL606834.1	0.429507814	1.32E-25	postive
GABARAPL1	AL606834.1	0.594295835	9.02E-53	postive
LINC00472	AL606834.1	0.613672808	4.40E-57	postive
LPIN1	AL606834.1	0.597508282	1.82E-53	postive
TUBE1	AC093297.2	0.445988903	1.05E-27	postive
ALOX12	AC093297.2	0.41853088	2.85E-24	postive
ATM	AC093297.2	0.431868993	6.72E-26	postive
HELLS	AC026368.1	0.484564903	4.38E-33	postive
TUBE1	AC026368.1	0.401878112	2.45E-22	postive
GABPB1	AC026368.1	0.531910153	1.07E-40	postive
LINC00472	AC026368.1	0.695257451	4.78E-79	postive
ATM	AC026368.1	0.614152037	3.41E-57	postive
FBXW7	AC026368.1	0.449323368	3.84E-28	postive
HSPB1	MINCR	0.401090043	3.00E-22	postive
HSF1	MINCR	0.569036389	1.43E-47	postive
HRAS	MINCR	0.423690184	6.83E-25	postive
NCOA4	MINCR	-0.442483701	3.01E-27	negative
PHKG2	MINCR	0.651357067	2.19E-66	postive
MAPK1	MINCR	-0.417345205	3.95E-24	negative
TAZ	MINCR	0.607496443	1.12E-55	postive
ALOX12	TMCC1-AS1	0.411107769	2.14E-23	postive

HELLS	AC084871.1	0.517890056	2.56E-38	postive
KLHL24	AC084871.1	0.400205979	3.77E-22	postive
TUBE1	AC084871.1	0.403214709	1.73E-22	postive
IREB2	AC084871.1	0.407263849	5.96E-23	postive
GABPB1	AC084871.1	0.529537188	2.74E-40	postive
ATG7	AC084871.1	0.470074699	5.55E-31	postive
LINC00472	AC084871.1	0.72041048	2.17E-87	postive
ATM	AC084871.1	0.714853645	1.81E-85	postive
FBXW7	AC084871.1	0.468185609	1.03E-30	postive
ZNF419	AL160006.1	0.481295052	1.33E-32	postive
VEGFA	AL160006.1	0.573317568	2.02E-48	postive
TUBE1	AL160006.1	0.441494226	4.04E-27	postive
SETD1B	AL160006.1	0.593880789	1.11E-52	postive
ALOX12	AL160006.1	0.44034853	5.68E-27	postive
YY1AP1	AL160006.1	0.420736075	1.55E-24	postive
ALOX12	AC016396.1	0.406539498	7.21E-23	postive
ALOX12	AC118754.1	0.496491379	6.83E-35	postive
MIOX	AC118754.1	0.412958004	1.30E-23	postive
TAZ	AC118754.1	0.458858636	2.01E-29	postive
ALOX12	LINC01550	0.429227513	1.43E-25	postive
ALOX5	AL731567.1	0.536193576	1.90E-41	postive
ALOX12	AL731567.1	0.400799523	3.23E-22	postive
PHKG2	AL731567.1	0.541399712	2.25E-42	postive
TAZ	AL731567.1	0.451646013	1.89E-28	postive
SLC3A2	AC148477.4	0.494862737	1.22E-34	postive
FH	AC148477.4	0.440457703	5.50E-27	postive
ISCU	AC148477.4	0.631761238	2.18E-61	postive
DDIT3	AC148477.4	0.502423438	8.11E-36	postive
SLC2A12	AC148477.4	0.438068338	1.11E-26	postive
CS	AC148477.4	0.502753325	7.19E-36	postive
GOT1	AC148477.4	0.42826901	1.88E-25	postive
ATG4D	AC148477.4	0.595131678	5.96E-53	postive
MAP1LC3A	AC148477.4	0.458844379	2.02E-29	postive
GABARAPL1	AC148477.4	0.740768229	7.60E-95	postive
WIPI2	AC148477.4	0.445043966	1.40E-27	postive
LPIN1	AC148477.4	0.62200283	4.97E-59	postive
PLIN2	DOCK8-AS1	0.478691107	3.21E-32	postive
PCK2	DOCK8-AS1	0.416945208	4.41E-24	postive
PEBP1	DOCK8-AS1	0.403204272	1.73E-22	postive
MIOX	DOCK8-AS1	0.459239153	1.78E-29	postive
HELLS	AC068790.5	0.545260953	4.51E-43	postive
ZNF419	AC068790.5	0.436527924	1.74E-26	postive
TUBE1	AC068790.5	0.520547326	9.23E-39	postive
ALOX12	AC068790.5	0.512441079	2.01E-37	postive
GABPB1	AC068790.5	0.562392932	2.81E-46	postive
LINC00472	AC068790.5	0.647505393	2.25E-65	postive
ATM	AC068790.5	0.708078328	3.45E-83	postive
FBXW7	AC068790.5	0.553333509	1.47E-44	postive
ZNF419	AL121655.1	0.473611889	1.74E-31	postive
TUBE1	AL121655.1	0.488843686	1.00E-33	postive
GABPB1	AL121655.1	0.46297038	5.48E-30	postive
LINC00472	AL121655.1	0.603299158	9.74E-55	postive
TAZ	AL121655.1	0.42733476	2.45E-25	postive
PROM2	AL008726.1	0.438603447	9.49E-27	postive
TFAP2C	AL008726.1	0.44319175	2.44E-27	postive
HELLS	AC090739.1	0.511441317	2.93E-37	postive
ZNF419	AC090739.1	0.405145481	1.04E-22	postive
KLHL24	AC090739.1	0.466255685	1.91E-30	postive

TUBE1	AC090739.1	0.466162723	1.97E-30	postive
ALOX12	AC090739.1	0.435394734	2.43E-26	postive
IREB2	AC090739.1	0.421169904	1.38E-24	postive
GABPB1	AC090739.1	0.585946463	5.30E-51	postive
PIK3CA	AC090739.1	0.423662886	6.88E-25	postive
MAPK8	AC090739.1	0.442742371	2.79E-27	postive
LINC00472	AC090739.1	0.87796465	6.30E-174	postive
ATM	AC090739.1	0.650849473	2.99E-66	postive
FBXW7	AC090739.1	0.471371132	3.63E-31	postive
SLC40A1	BACE1-AS	-0.406198718	7.89E-23	negative
LAMP2	BACE1-AS	-0.436716575	1.65E-26	negative
ZNF419	BACE1-AS	0.418771696	2.67E-24	postive
DRD4	BACE1-AS	0.431153193	8.25E-26	postive
ALOX12	BACE1-AS	0.431671988	7.11E-26	postive
NRAS	BACE1-AS	-0.439390326	7.53E-27	negative
NCOA4	BACE1-AS	-0.53988812	4.19E-42	negative
PHKG2	BACE1-AS	0.511275627	3.11E-37	postive
BECN1	BACE1-AS	-0.427065981	2.64E-25	negative
ANO6	BACE1-AS	-0.430454437	1.01E-25	negative
TAZ	BACE1-AS	0.625527753	7.15E-60	postive
MTDH	BACE1-AS	-0.49452176	1.37E-34	negative
HRAS	ODC1-DT	0.423729932	6.75E-25	postive
PHKG2	ODC1-DT	0.484790636	4.06E-33	postive
TAZ	ODC1-DT	0.453806421	9.71E-29	postive
ZNF419	DICER1-AS1	0.507715355	1.17E-36	postive
TUBE1	DICER1-AS1	0.460857755	1.07E-29	postive
DRD4	DICER1-AS1	0.402127825	2.29E-22	postive
ALOX12	DICER1-AS1	0.580757772	6.29E-50	postive
PHKG2	DICER1-AS1	0.517203436	3.33E-38	postive
TAZ	DICER1-AS1	0.621679948	5.93E-59	postive
ZNF419	AL139349.1	0.410330095	2.64E-23	postive
PHKG2	AL139349.1	0.424461307	5.50E-25	postive
ULK1	AL139349.1	0.461903369	7.69E-30	postive
TAZ	AL139349.1	0.431892281	6.68E-26	postive
GCLC	CYTOR	-0.437982895	1.14E-26	negative
SLC2A6	CYTOR	0.510066995	4.89E-37	postive
IREB2	CYTOR	-0.41164856	1.85E-23	negative
LPCAT3	CYTOR	-0.406436923	7.41E-23	negative
HRAS	CYTOR	0.507288529	1.37E-36	postive
NCOA4	CYTOR	-0.437966639	1.14E-26	negative
MAPK1	CYTOR	-0.407957132	4.96E-23	negative
PRKAA2	CYTOR	-0.423361406	7.49E-25	negative
EGLN2	CYTOR	0.499993871	1.95E-35	postive
HELLS	AP000907.2	0.463973737	3.98E-30	postive
ZNF419	AP000907.2	0.433381706	4.35E-26	postive
TUBE1	AP000907.2	0.471210932	3.83E-31	postive
ALOX12	AP000907.2	0.663954075	8.47E-70	postive
GABPB1	AP000907.2	0.435806959	2.15E-26	postive
ATM	AP000907.2	0.582103538	3.32E-50	postive
FBXW7	AP000907.2	0.418996964	2.51E-24	postive
TUBE1	AL356361.2	0.414348721	8.94E-24	postive
ATM	AL356361.2	0.527173509	6.99E-40	postive
FANCD2	LINC02528	0.415181595	7.13E-24	postive
GCH1	LINC02528	0.629047394	1.01E-60	postive
NCF2	LINC02528	0.41526651	6.96E-24	postive
CYBB	LINC02528	0.629095492	9.80E-61	postive
IFNG	LINC02528	0.730434923	5.63E-91	postive
TLR4	LINC02528	0.402751407	1.95E-22	postive

TUBE1	AC068051.1	0.460988039	1.03E-29	postive
ALOX12	AC068051.1	0.447352596	6.99E-28	postive
MAPK8	AC068051.1	0.493304593	2.11E-34	postive
PRKAA2	AC068051.1	0.468821194	8.34E-31	postive
ATM	AC068051.1	0.611078267	1.73E-56	postive
HELLS	AC005740.3	0.454129424	8.79E-29	postive
KLHL24	AC005740.3	0.509300473	6.50E-37	postive
TUBE1	AC005740.3	0.426472948	3.12E-25	postive
IREB2	AC005740.3	0.405101994	1.05E-22	postive
GABPB1	AC005740.3	0.557413875	2.51E-45	postive
PIK3CA	AC005740.3	0.432188708	6.13E-26	postive
MAPK8	AC005740.3	0.401836068	2.47E-22	postive
LINC00472	AC005740.3	0.885633566	5.04E-181	postive
ATM	AC005740.3	0.658345778	2.94E-68	postive
FBXW7	AC005740.3	0.469666056	6.34E-31	postive
FANCD2	AP001527.2	0.400405839	3.58E-22	postive
PHKG2	AP001527.2	0.431962438	6.54E-26	postive
PLIN2	LINC01843	0.413539881	1.11E-23	postive
BNIP3	LINC01843	0.40540065	9.74E-23	postive
ISCU	AC023794.2	0.498978092	2.81E-35	postive
DDIT3	AC023794.2	0.448663319	4.70E-28	postive
ATG4D	AC023794.2	0.474327155	1.37E-31	postive
MAP1LC3A	AC023794.2	0.531255589	1.38E-40	postive
GABARAPL2	AC023794.2	0.401433709	2.74E-22	postive
GABARAPL1	AC023794.2	0.450928229	2.35E-28	postive
LPIN1	AC023794.2	0.409971847	2.90E-23	postive
HELLS	ZKSCAN2-D	0.41023538	2.70E-23	postive
BRD4	ZKSCAN2-D	0.404483197	1.24E-22	postive
ZNF419	ZKSCAN2-D	0.602838991	1.23E-54	postive
VEGFA	ZKSCAN2-D	0.408741536	4.03E-23	postive
TUBE1	ZKSCAN2-D	0.619223952	2.25E-58	postive
SETD1B	ZKSCAN2-D	0.446312944	9.57E-28	postive
DRD4	ZKSCAN2-D	0.417340141	3.96E-24	postive
ALOX12	ZKSCAN2-D	0.678295884	6.82E-74	postive
GABPB1	ZKSCAN2-D	0.467171853	1.42E-30	postive
PHKG2	ZKSCAN2-D	0.549311309	8.19E-44	postive
LINC00472	ZKSCAN2-D	0.446637825	8.68E-28	postive
ATM	ZKSCAN2-D	0.484954827	3.84E-33	postive
TAZ	ZKSCAN2-D	0.650436461	3.84E-66	postive
FBXW7	ZKSCAN2-D	0.433696732	3.97E-26	postive
HELLS	DGUOK-AS1	0.570150926	8.60E-48	postive
ZNF419	DGUOK-AS1	0.518013613	2.44E-38	postive
TUBE1	DGUOK-AS1	0.576866177	3.90E-49	postive
ALOX12	DGUOK-AS1	0.543724896	8.57E-43	postive
GABPB1	DGUOK-AS1	0.56363707	1.62E-46	postive
LINC00472	DGUOK-AS1	0.519518795	1.37E-38	postive
ATM	DGUOK-AS1	0.608009668	8.60E-56	postive
TAZ	DGUOK-AS1	0.433715245	3.95E-26	postive
FBXW7	DGUOK-AS1	0.505249867	2.89E-36	postive
HELLS	AC125437.1	0.493366908	2.07E-34	postive
CHAC1	AC139100.2	0.49338681	2.05E-34	postive
PHKG2	AC139100.2	0.507247543	1.39E-36	postive
ATG4D	AC139100.2	0.41726177	4.04E-24	postive
MAP1LC3A	AC139100.2	0.413220651	1.21E-23	postive
TAZ	AC139100.2	0.516969954	3.63E-38	postive
HELLS	AC069120.1	0.419439889	2.22E-24	postive
HELLS	AC090948.1	0.432062377	6.36E-26	postive
KLHL24	AC090948.1	0.48336375	6.61E-33	postive

GABPB1	AC090948.1	0.549039936	9.19E-44	postive
PIK3CA	AC090948.1	0.419539727	2.16E-24	postive
ATG7	AC090948.1	0.401807739	2.49E-22	postive
LINC00472	AC090948.1	0.892209747	1.58E-187	postive
ATM	AC090948.1	0.604662791	4.84E-55	postive
FBXW7	AC090948.1	0.426454363	3.14E-25	postive
ZNF419	AP001010.1	0.407909545	5.02E-23	postive
ALOX12	AP001010.1	0.490900804	4.91E-34	postive
TAZ	AP001010.1	0.454292114	8.36E-29	postive
HSF1	FOXD2-AS1	0.432060843	6.36E-26	postive
ATF4	FOXD2-AS1	0.407893663	5.04E-23	postive
PML	FOXD2-AS1	0.417353929	3.94E-24	postive
BRD4	FOXD2-AS1	0.462641113	6.08E-30	postive
NCOA4	FOXD2-AS1	-0.4404328	5.54E-27	negative
TAZ	FOXD2-AS1	0.51216621	2.23E-37	postive
TFAP2C	AC010336.2	0.443674205	2.11E-27	postive
HBA1	AC010336.2	0.408425236	4.38E-23	postive
ATM	AC246817.1	0.488922053	9.77E-34	postive
VDAC2	NRSN2-AS1	0.41758528	3.70E-24	postive
SRXN1	NRSN2-AS1	0.423849552	6.53E-25	postive
IREB2	AP001528.1	0.458285584	2.41E-29	postive
ZEB1	AP001528.1	0.41470058	8.12E-24	postive
MAPK8	AP001528.1	0.456584057	4.10E-29	postive
PRKAA2	AP001528.1	0.413981779	9.87E-24	postive
PRKAA1	AP001528.1	0.405446201	9.62E-23	postive
ATM	AP001528.1	0.48214707	9.99E-33	postive
SIRT1	AP001528.1	0.429017766	1.52E-25	postive
HELLS	AC090198.1	0.449754081	3.37E-28	postive
ZNF419	AC090198.1	0.459918838	1.44E-29	postive
KLHL24	AC090198.1	0.422722262	8.95E-25	postive
TUBE1	AC090198.1	0.524411016	2.06E-39	postive
SETD1B	AC090198.1	0.411943841	1.71E-23	postive
ALOX12	AC090198.1	0.498590518	3.23E-35	postive
IREB2	AC090198.1	0.521423456	6.58E-39	postive
SP1	AC090198.1	0.493177808	2.21E-34	postive
GABPB1	AC090198.1	0.564496127	1.10E-46	postive
PIK3CA	AC090198.1	0.474178937	1.44E-31	postive
KRAS	AC090198.1	0.428099084	1.97E-25	postive
ULK2	AC090198.1	0.431282606	7.95E-26	postive
ZEB1	AC090198.1	0.42342477	7.35E-25	postive
MAPK8	AC090198.1	0.571823612	4.01E-48	postive
LINC00472	AC090198.1	0.551565066	3.14E-44	postive
PRKAA2	AC090198.1	0.463948161	4.01E-30	postive
PRKAA1	AC090198.1	0.465541797	2.41E-30	postive
ATM	AC090198.1	0.684382076	1.06E-75	postive
FBXW7	AC090198.1	0.517328763	3.17E-38	postive
TFAP2C	AC013553.3	0.543082424	1.12E-42	postive
HBA1	AC013553.3	0.531028037	1.52E-40	postive
ALOX12	MIS18A-AS1	0.41177622	1.79E-23	postive
TFAP2C	MIS18A-AS1	0.550481522	4.98E-44	postive
HBA1	MIS18A-AS1	0.478417306	3.52E-32	postive
DUOX1	MIS18A-AS1	0.447556652	6.57E-28	postive
HELLS	AL020997.3	0.484027651	5.27E-33	postive
TUBE1	AL020997.3	0.526993578	7.50E-40	postive
ALOX12	AL020997.3	0.526439507	9.32E-40	postive
GABPB1	AL020997.3	0.49055593	5.53E-34	postive
LINC00472	AL020997.3	0.46672369	1.64E-30	postive
ATM	AL020997.3	0.675517953	4.41E-73	postive

FBXW7	AL020997.3	0.461805509	7.93E-30	postive
ZNF419	AL590617.2	0.419889773	1.96E-24	postive
SLC2A6	AL590617.2	0.401371817	2.79E-22	postive
HRAS	AL590617.2	0.402440378	2.11E-22	postive
PHKG2	AL590617.2	0.408825617	3.94E-23	postive
EGLN2	AL590617.2	0.505209078	2.94E-36	postive
TAZ	AL590617.2	0.520932587	7.96E-39	postive
ALOX12	AC116447.1	0.423792592	6.64E-25	postive
HELLS	C21orf62-AS	0.437821675	1.19E-26	postive
TUBE1	C21orf62-AS	0.477741199	4.41E-32	postive
ALOX12	C21orf62-AS	0.500308424	1.74E-35	postive
MAPK8	C21orf62-AS	0.417441786	3.85E-24	postive
ATM	C21orf62-AS	0.596541972	2.95E-53	postive
TFAP2C	USP2-AS1	0.496501426	6.81E-35	postive
HBA1	USP2-AS1	0.425685183	3.90E-25	postive
FANCD2	KDM4A-AS1	0.488945415	9.69E-34	postive
HELLS	KDM4A-AS1	0.612219919	9.49E-57	postive
ZNF419	KDM4A-AS1	0.553271549	1.51E-44	postive
TUBE1	KDM4A-AS1	0.558148406	1.82E-45	postive
ALOX12	KDM4A-AS1	0.659750517	1.22E-68	postive
GABPB1	KDM4A-AS1	0.476346647	7.02E-32	postive
ATM	KDM4A-AS1	0.510486612	4.18E-37	postive
TAZ	KDM4A-AS1	0.4682025	1.02E-30	postive
FBXW7	KDM4A-AS1	0.495609777	9.35E-35	postive
BRD4	COL4A2-AS1	0.433944738	3.69E-26	postive
ZNF419	COL4A2-AS1	0.47592128	8.09E-32	postive
VEGFA	COL4A2-AS1	0.486551043	2.22E-33	postive
TUBE1	COL4A2-AS1	0.459937272	1.43E-29	postive
SETD1B	COL4A2-AS1	0.436073738	1.99E-26	postive
ALOX12	COL4A2-AS1	0.524934448	1.68E-39	postive
GABPB1	COL4A2-AS1	0.423229237	7.77E-25	postive
ZEB1	COL4A2-AS1	0.453505979	1.07E-28	postive
FBXW7	COL4A2-AS1	0.453721392	9.97E-29	postive
KLHL24	MECOM-AS1	0.600675905	3.70E-54	postive
IREB2	MECOM-AS1	0.403743611	1.50E-22	postive
GABPB1	MECOM-AS1	0.408673948	4.10E-23	postive
PIK3CA	MECOM-AS1	0.488717529	1.05E-33	postive
KRAS	MECOM-AS1	0.426625582	2.99E-25	postive
LINC00472	MECOM-AS1	0.944940985	1.11E-262	postive
HELLS	SEMA6A-AS1	0.527577251	5.96E-40	postive
TUBE1	SEMA6A-AS1	0.576714247	4.19E-49	postive
ALOX12	SEMA6A-AS1	0.558951279	1.28E-45	postive
GABPB1	SEMA6A-AS1	0.534416027	3.89E-41	postive
MAPK8	SEMA6A-AS1	0.450401125	2.77E-28	postive
LINC00472	SEMA6A-AS1	0.559370523	1.07E-45	postive
ATM	SEMA6A-AS1	0.742482294	1.66E-95	postive
FBXW7	SEMA6A-AS1	0.467362157	1.34E-30	postive
ATP6V1G2	AC023906.5	0.404229354	1.32E-22	postive
ACO1	AC023906.5	0.631763726	2.18E-61	postive
HSPB1	AL035461.2	0.407469334	5.64E-23	postive
FANCD2	AL035461.2	0.431068114	8.46E-26	postive
TFAP2C	AL035461.2	0.495351912	1.02E-34	postive
HBA1	AL035461.2	0.542550991	1.40E-42	postive
PHKG2	AL035461.2	0.417418878	3.87E-24	postive
EGLN2	AL035461.2	0.409385673	3.39E-23	postive
FANCD2	AC131009.1	0.438542935	9.66E-27	postive
TFAP2C	AC131009.1	0.552969319	1.72E-44	postive
TFAP2C	SUCLA2-AS1	0.40608991	8.12E-23	postive

HBA1	SUCLA2-AS1	0.644822853	1.12E-64	postive
HRAS	SUCLA2-AS1	0.455723435	5.36E-29	postive
EGLN2	SUCLA2-AS1	0.547216898	1.98E-43	postive
ZNF419	AL731571.1	0.509505256	6.02E-37	postive
VEGFA	AL731571.1	0.589686742	8.68E-52	postive
TUBE1	AL731571.1	0.586924979	3.31E-51	postive
SETD1B	AL731571.1	0.460617778	1.16E-29	postive
ALOX12	AL731571.1	0.693687219	1.49E-78	postive
GABPB1	AL731571.1	0.425034892	4.68E-25	postive
ATM	AL731571.1	0.50244571	8.04E-36	postive
YY1AP1	AL731571.1	0.423147298	7.95E-25	postive
FBXW7	AL731571.1	0.447848492	6.02E-28	postive
HELLS	AGAP1-IT1	0.493930684	1.69E-34	postive
ALOX12	AGAP1-IT1	0.404660586	1.18E-22	postive
LINC00472	AGAP1-IT1	0.41343759	1.14E-23	postive
ATM	AGAP1-IT1	0.541600487	2.07E-42	postive
HELLS	AL603756.1	0.524064968	2.36E-39	postive
ALOX12	AL603756.1	0.445030499	1.41E-27	postive
GABPB1	AL603756.1	0.466022511	2.06E-30	postive
ATG7	AL603756.1	0.442208541	3.27E-27	postive
LINC00472	AL603756.1	0.479760416	2.24E-32	postive
ATM	AL603756.1	0.685281298	5.67E-76	postive
FBXW7	AL603756.1	0.459243629	1.78E-29	postive
TFAP2C	AC067817.2	0.581861744	3.73E-50	postive
HBA1	AC067817.2	0.541185699	2.46E-42	postive
DUOX1	AC067817.2	0.409547375	3.25E-23	postive
GPX4	AC005034.5	-0.409226308	3.54E-23	negative
RB1	AC005034.5	0.576896191	3.85E-49	postive
OTUB1	AC005034.5	-0.498644868	3.17E-35	negative
OXSR1	AC005034.5	0.433932582	3.71E-26	postive
KLHL24	AC005034.5	0.495174262	1.09E-34	postive
MAP3K5	AC005034.5	0.476816926	6.00E-32	postive
MAPK14	AC005034.5	0.448195749	5.41E-28	postive
IREB2	AC005034.5	0.66494151	4.50E-70	postive
SP1	AC005034.5	0.527058662	7.31E-40	postive
GABPB1	AC005034.5	0.469422115	6.86E-31	postive
PIK3CA	AC005034.5	0.642380667	4.74E-64	postive
NRAS	AC005034.5	0.456016852	4.89E-29	postive
KRAS	AC005034.5	0.608216701	7.72E-56	postive
SLC38A1	AC005034.5	0.418231856	3.10E-24	postive
MAPK1	AC005034.5	0.49485508	1.22E-34	postive
ZEB1	AC005034.5	0.447445881	6.80E-28	postive
MAPK8	AC005034.5	0.601454139	2.49E-54	postive
MAPK9	AC005034.5	0.525405059	1.40E-39	postive
LINC00472	AC005034.5	0.587378859	2.66E-51	postive
PRKAA2	AC005034.5	0.632855271	1.17E-61	postive
PRKAA1	AC005034.5	0.631435354	2.63E-61	postive
TGFBR1	AC005034.5	0.435852381	2.12E-26	postive
ANO6	AC005034.5	0.406306203	7.67E-23	postive
TLR4	AC005034.5	0.474408999	1.34E-31	postive
ATM	AC005034.5	0.612193918	9.62E-57	postive
MTDH	AC005034.5	0.415060632	7.37E-24	postive
SIRT1	AC005034.5	0.527504685	6.13E-40	postive
BACH1	AC005034.5	0.472285389	2.69E-31	postive
HELLS	AC006504.5	0.455821842	5.20E-29	postive
LINC00472	AC069234.5	0.483042122	7.37E-33	postive
ATM	AC069234.5	0.420724906	1.56E-24	postive
ZNF419	AC002059.1	0.453911096	9.40E-29	postive

ALOX12	AC002059.1	0.50780678	1.13E-36	postive
TAZ	AC002059.1	0.458442694	2.29E-29	postive
FANCD2	AL163051.1	0.401237493	2.89E-22	postive
TFAP2C	AL163051.1	0.582443231	2.83E-50	postive
HBA1	AL163051.1	0.587988408	1.98E-51	postive
PHKG2	HDAC4-AS1	0.525144555	1.55E-39	postive
TAZ	HDAC4-AS1	0.566580712	4.33E-47	postive
PTGS2	MIAT	0.559132635	1.18E-45	postive
IL6	MIAT	0.638340583	5.03E-63	postive
SLC2A3	MIAT	0.554169786	1.02E-44	postive
EGLN2	Z97192.2	0.477902798	4.18E-32	postive
HELLS	AC021078.1	0.487115843	1.83E-33	postive
ZNF419	AC021078.1	0.51501606	7.63E-38	postive
TUBE1	AC021078.1	0.635247398	2.99E-62	postive
ALOX12	AC021078.1	0.570530445	7.24E-48	postive
GABPB1	AC021078.1	0.556894821	3.15E-45	postive
MAPK8	AC021078.1	0.424389404	5.61E-25	postive
LINC00472	AC021078.1	0.634438528	4.76E-62	postive
ATM	AC021078.1	0.661936267	3.06E-69	postive
FBXW7	AC021078.1	0.542310242	1.54E-42	postive
PHKG2	AC127070.1	0.439808819	6.66E-27	postive
HELLS	AC012404.1	0.501306098	1.22E-35	postive
KLHL24	AC012404.1	0.428872756	1.58E-25	postive
IREB2	AC012404.1	0.411489037	1.93E-23	postive
GABPB1	AC012404.1	0.547527486	1.74E-43	postive
PIK3CA	AC012404.1	0.421735505	1.18E-24	postive
ATG7	AC012404.1	0.438399176	1.01E-26	postive
MAPK8	AC012404.1	0.405347047	9.88E-23	postive
LINC00472	AC012404.1	0.813545017	1.55E-128	postive
ATM	AC012404.1	0.681113809	1.00E-74	postive
FBXW7	AC012404.1	0.431216824	8.10E-26	postive
TFAP2C	AC124242.1	0.419664298	2.09E-24	postive
HBA1	AC124242.1	0.42905758	1.50E-25	postive
TUBE1	AL645940.1	0.412316622	1.55E-23	postive
PHKG2	AL645940.1	0.617621139	5.34E-58	postive
TAZ	AL645940.1	0.708304705	2.90E-83	postive
ZNF419	OBSCN-AS1	0.46844446	9.43E-31	postive
TUBE1	OBSCN-AS1	0.407207744	6.05E-23	postive
ALOX12	OBSCN-AS1	0.530157447	2.14E-40	postive
TAZ	OBSCN-AS1	0.448391322	5.10E-28	postive
TFAP2C	AC010463.2	0.572006264	3.69E-48	postive
HBA1	AC010463.2	0.519461105	1.40E-38	postive
FANCD2	TENM3-AS1	0.415564012	6.42E-24	postive
ANGPTL7	TENM3-AS1	0.460679704	1.13E-29	postive
BRD4	AC005387.1	0.404846874	1.13E-22	postive
TUBE1	AC005387.1	0.4070414	6.32E-23	postive
DRD4	AC005387.1	0.408825723	3.94E-23	postive
ALOX12	AC005387.1	0.412593218	1.44E-23	postive
NCOA4	AC005387.1	-0.460389988	1.24E-29	negative
PHKG2	AC005387.1	0.653781223	4.98E-67	postive
TAZ	AC005387.1	0.767025192	1.44E-105	postive
ALOX12	AL353803.2	0.431295517	7.92E-26	postive
AKR1C1	AL391427.1	0.544405436	6.45E-43	postive
AKR1C2	AL391427.1	0.517900643	2.55E-38	postive
NQO1	AL391427.1	0.430303653	1.05E-25	postive
LINC00472	AL391427.1	0.460837592	1.08E-29	postive
ZNF419	LINC00899	0.445286905	1.30E-27	postive
ALOX12	LINC00899	0.483787306	5.72E-33	postive

HELLS	AC010834.3	0.517782168	2.67E-38	postive
MTOR	AC010834.3	0.419901504	1.96E-24	postive
ZNF419	AC010834.3	0.469263902	7.22E-31	postive
KLHL24	AC010834.3	0.560279722	7.15E-46	postive
TUBE1	AC010834.3	0.523778593	2.64E-39	postive
MAP3K5	AC010834.3	0.400085672	3.89E-22	postive
ALOX12	AC010834.3	0.5268752	7.86E-40	postive
IREB2	AC010834.3	0.57111181	5.55E-48	postive
SP1	AC010834.3	0.416814561	4.57E-24	postive
GABPB1	AC010834.3	0.611238282	1.59E-56	postive
PIK3CA	AC010834.3	0.538777073	6.61E-42	postive
KRAS	AC010834.3	0.406552303	7.19E-23	postive
ATG7	AC010834.3	0.42179162	1.16E-24	postive
MAPK8	AC010834.3	0.52206422	5.14E-39	postive
LINC00472	AC010834.3	0.766328059	2.89E-105	postive
PRKAA2	AC010834.3	0.455131907	6.44E-29	postive
PRKAA1	AC010834.3	0.437854114	1.18E-26	postive
ATM	AC010834.3	0.781314284	5.12E-112	postive
FBXW7	AC010834.3	0.513142384	1.55E-37	postive
PML	ITGB2-AS1	0.423212195	7.80E-25	postive
PHKG2	ITGB2-AS1	0.547993524	1.43E-43	postive
TAZ	ITGB2-AS1	0.544712528	5.68E-43	postive
TFAP2C	TMEM167B-I	0.500993805	1.36E-35	postive
HBA1	TMEM167B-I	0.492349493	2.95E-34	postive
ALOX12B	TMEM167B-I	0.406047923	8.21E-23	postive
LAMP2	AC003092.1	0.513534061	1.33E-37	postive
HELLS	AC092338.1	0.552736166	1.90E-44	postive
ALOX12	AC092338.1	0.499721243	2.15E-35	postive
TFAP2C	AC092338.1	0.403018369	1.82E-22	postive
GABPB1	AC092338.1	0.448426651	5.05E-28	postive
DUOX1	AC092338.1	0.428290802	1.87E-25	postive
LINC00472	AC092338.1	0.504664143	3.59E-36	postive
ATM	AC092338.1	0.511290711	3.10E-37	postive
FBXW7	AC092338.1	0.417686379	3.60E-24	postive
VEGFA	AC105020.1	0.440860655	4.88E-27	postive
TUBE1	AC105020.1	0.41405356	9.68E-24	postive
ALOX12	AC105020.1	0.494066454	1.61E-34	postive
TAZ	AC105020.1	0.472359181	2.63E-31	postive
HELLS	AC073487.1	0.534353765	3.99E-41	postive
ZNF419	AC073487.1	0.506121796	2.10E-36	postive
TUBE1	AC073487.1	0.597180622	2.15E-53	postive
ALOX12	AC073487.1	0.598650425	1.03E-53	postive
GABPB1	AC073487.1	0.582983593	2.19E-50	postive
MAPK8	AC073487.1	0.402702875	1.97E-22	postive
LINC00472	AC073487.1	0.727312473	7.67E-90	postive
ATM	AC073487.1	0.663168673	1.40E-69	postive
FBXW7	AC073487.1	0.52151505	6.36E-39	postive
TFAP2C	AL135790.1	0.581538375	4.35E-50	postive
HBA1	AL135790.1	0.523861438	2.56E-39	postive
FANCD2	AC020915.1	0.405652464	9.11E-23	postive
HELLS	AC020915.1	0.531721268	1.15E-40	postive
ZNF419	AC020915.1	0.542362213	1.51E-42	postive
TUBE1	AC020915.1	0.475225788	1.02E-31	postive
ALOX12	AC020915.1	0.551261175	3.57E-44	postive
GABPB1	AC020915.1	0.493232432	2.17E-34	postive
LINC00472	AC020915.1	0.544621903	5.89E-43	postive
ATM	AC020915.1	0.503089939	6.37E-36	postive
FBXW7	AC020915.1	0.481377142	1.30E-32	postive

ZNF419	AC127502.2	0.404952624	1.10E-22	postive
TUBE1	AC127502.2	0.431541243	7.38E-26	postive
ALOX12	AC127502.2	0.418833769	2.63E-24	postive
PHKG2	AC127502.2	0.553871784	1.17E-44	postive
TAZ	AC127502.2	0.666699335	1.45E-70	postive
MAPK1	AC124854.1	0.452114084	1.64E-28	postive
MAPK8	AC124854.1	0.483601095	6.09E-33	postive
PRKAA2	AC124854.1	0.50757467	1.23E-36	postive
SIRT1	AC124854.1	0.458517177	2.24E-29	postive
ZNF419	AL358472.2	0.603374284	9.37E-55	postive
VEGFA	AL358472.2	0.452087238	1.65E-28	postive
TUBE1	AL358472.2	0.532701702	7.76E-41	postive
ALOX12	AL358472.2	0.526024765	1.10E-39	postive
GABPB1	AL358472.2	0.439059903	8.30E-27	postive
YY1AP1	AL358472.2	0.403567182	1.57E-22	postive
TAZ	AL358472.2	0.56428788	1.21E-46	postive
TUBE1	LINC01138	0.41548853	6.56E-24	postive
ALOX12	LINC01138	0.458912411	1.98E-29	postive
PHKG2	LINC01138	0.525406679	1.40E-39	postive
TAZ	LINC01138	0.513367852	1.42E-37	postive
HELLS	AC108449.2	0.439139765	8.11E-27	postive
MTOR	AC108449.2	0.523480343	2.97E-39	postive
OTUB1	AC108449.2	-0.409728025	3.10E-23	negative
OXSR1	AC108449.2	0.402244686	2.22E-22	postive
ZNF419	AC108449.2	0.413004537	1.29E-23	postive
KLHL24	AC108449.2	0.540971718	2.68E-42	postive
TUBE1	AC108449.2	0.460666495	1.14E-29	postive
MAP3K5	AC108449.2	0.450987222	2.31E-28	postive
ALOX12	AC108449.2	0.480383108	1.82E-32	postive
IREB2	AC108449.2	0.682764292	3.24E-75	postive
SP1	AC108449.2	0.430843802	9.02E-26	postive
GABPB1	AC108449.2	0.569509268	1.15E-47	postive
PIK3CA	AC108449.2	0.625465827	7.40E-60	postive
KRAS	AC108449.2	0.48773332	1.47E-33	postive
ATG7	AC108449.2	0.417428495	3.86E-24	postive
ZEB1	AC108449.2	0.517586093	2.87E-38	postive
MAPK8	AC108449.2	0.637708864	7.25E-63	postive
LINC00472	AC108449.2	0.719972695	3.09E-87	postive
PRKAA2	AC108449.2	0.533555557	5.50E-41	postive
PRKAA1	AC108449.2	0.503426611	5.63E-36	postive
TLR4	AC108449.2	0.454054785	9.00E-29	postive
ATM	AC108449.2	0.767135284	1.29E-105	postive
FBXW7	AC108449.2	0.492977259	2.37E-34	postive
FH	PART1	0.509196206	6.75E-37	postive
ISCU	PART1	0.579318334	1.24E-49	postive
KLHL24	PART1	0.513053331	1.60E-37	postive
SLC2A8	PART1	0.423513285	7.17E-25	postive
SLC2A12	PART1	0.455083852	6.54E-29	postive
GOT1	PART1	0.511293986	3.09E-37	postive
ATG4D	PART1	0.520320027	1.01E-38	postive
GABARAPL2	PART1	0.531200157	1.41E-40	postive
GABARAPL1	PART1	0.648207996	1.48E-65	postive
WIPI2	PART1	0.528115652	4.82E-40	postive
LINC00472	PART1	0.416066805	5.60E-24	postive
LPIN1	PART1	0.532251957	9.29E-41	postive
FANCD2	AC022211.3	0.585776157	5.76E-51	postive
HELLS	AC022211.3	0.53086429	1.62E-40	postive
ALOX12	AC022211.3	0.534697989	3.47E-41	postive

TFAP2C	AC022211.3	0.401787918	2.50E-22	postive
TFAP2C	AL109936.2	0.454285417	8.38E-29	postive
HBA1	AL109936.2	0.491423595	4.09E-34	postive
NCF2	PELATON	0.668912884	3.45E-71	postive
FTL	PELATON	0.587371398	2.67E-51	postive
CAPG	PELATON	0.409341199	3.43E-23	postive
PGD	PELATON	0.550855194	4.25E-44	postive
ALOX15B	PELATON	0.420704296	1.57E-24	postive
KLHL24	AP003059.2	0.41827922	3.06E-24	postive
TUBE1	AP003059.2	0.444338295	1.73E-27	postive
ALOX12	AP003059.2	0.413432354	1.14E-23	postive
GABPB1	AP003059.2	0.407890802	5.05E-23	postive
LINC00472	AP003059.2	0.695752533	3.34E-79	postive
LPIN1	AP003059.2	0.416654391	4.77E-24	postive
ATM	AP003059.2	0.545118619	4.79E-43	postive
HELLS	AC103739.1	0.500621153	1.56E-35	postive
KLHL24	AC103739.1	0.400462055	3.53E-22	postive
TUBE1	AC103739.1	0.431711614	7.03E-26	postive
ALOX12	AC103739.1	0.426656745	2.97E-25	postive
GABPB1	AC103739.1	0.552962581	1.72E-44	postive
ATG7	AC103739.1	0.409781463	3.05E-23	postive
MAPK8	AC103739.1	0.419449371	2.22E-24	postive
LINC00472	AC103739.1	0.740673256	8.26E-95	postive
ATM	AC103739.1	0.709510965	1.15E-83	postive
FBXW7	AC103739.1	0.500052184	1.91E-35	postive
HELLS	AC004908.3	0.447023843	7.72E-28	postive
ZNF419	AC004908.3	0.419563733	2.15E-24	postive
TUBE1	AC004908.3	0.502460711	8.00E-36	postive
GABPB1	AC004908.3	0.41641939	5.09E-24	postive
LINC00472	AC004908.3	0.52946141	2.83E-40	postive
TAZ	AC004908.3	0.410833906	2.30E-23	postive
HELLS	RUFY1-AS1	0.448693543	4.65E-28	postive
ZNF419	RUFY1-AS1	0.52030028	1.02E-38	postive
VEGFA	RUFY1-AS1	0.477279968	5.14E-32	postive
TUBE1	RUFY1-AS1	0.635598422	2.45E-62	postive
SETD1B	RUFY1-AS1	0.471097195	3.97E-31	postive
ALOX12	RUFY1-AS1	0.703298926	1.28E-81	postive
GABPB1	RUFY1-AS1	0.484174471	5.01E-33	postive
ATM	RUFY1-AS1	0.664292322	6.83E-70	postive
TAZ	RUFY1-AS1	0.402328623	2.17E-22	postive
FBXW7	RUFY1-AS1	0.50243295	8.08E-36	postive
ENPP2	AL359853.1	0.440113083	6.09E-27	postive
SLC1A4	AL359853.1	0.406860295	6.63E-23	postive
HSPA5	AC012409.3	-0.405682878	9.04E-23	negative
ZNF419	AC012409.3	0.463847876	4.14E-30	postive
VEGFA	AC012409.3	0.526773182	8.18E-40	postive
TUBE1	AC012409.3	0.648052335	1.62E-65	postive
ALOX12	AC012409.3	0.591349233	3.85E-52	postive
GABPB1	AC012409.3	0.426386107	3.20E-25	postive
ATM	AC012409.3	0.495491214	9.75E-35	postive
TAZ	AC012409.3	0.515588264	6.14E-38	postive
FBXW7	AC012409.3	0.424213073	5.90E-25	postive
HELLS	AC006270.1	0.507438325	1.29E-36	postive
TFAP2C	AC006270.1	0.426584722	3.03E-25	postive
HBA1	AC006270.1	0.438617384	9.46E-27	postive
DUOX1	AC006270.1	0.431341523	7.82E-26	postive
LINC00472	AC006270.1	0.401609302	2.62E-22	postive
ATM	AC006270.1	0.421953504	1.11E-24	postive

ZNF419	SLC25A25-A:	0.49161806	3.82E-34	postive
PHKG2	SLC25A25-A:	0.41616127	5.46E-24	postive
ATG4D	SLC25A25-A:	0.509413597	6.23E-37	postive
GABARAPL1	SLC25A25-A:	0.419254364	2.34E-24	postive
LPIN1	SLC25A25-A:	0.546904633	2.26E-43	postive
TAZ	SLC25A25-A:	0.424460222	5.50E-25	postive
ALOX12	AC012184.3	0.403110696	1.77E-22	postive
ISCU	LINC01543	0.580797737	6.17E-50	postive
ALB	LINC01543	0.401014398	3.06E-22	postive
CS	LINC01543	0.418135474	3.18E-24	postive
GABARAPL1	LINC01543	0.513526866	1.34E-37	postive
CDO1	LINC01543	0.402100384	2.31E-22	postive
LPIN1	LINC01543	0.411435942	1.96E-23	postive
HELLS	MIR17HG	0.447214185	7.29E-28	postive
TUBE1	MIR17HG	0.434995596	2.72E-26	postive
GABPB1	MIR17HG	0.515755451	5.77E-38	postive
MAPK8	MIR17HG	0.440490536	5.44E-27	postive
LINC00472	MIR17HG	0.54148595	2.17E-42	postive
ATM	MIR17HG	0.642286784	5.01E-64	postive
FBXW7	MIR17HG	0.489680655	7.51E-34	postive
HELLS	H3-3A-DT	0.512268593	2.15E-37	postive
ZNF419	H3-3A-DT	0.427756259	2.17E-25	postive
TUBE1	H3-3A-DT	0.555708949	5.27E-45	postive
ALOX12	H3-3A-DT	0.560185029	7.46E-46	postive
GABPB1	H3-3A-DT	0.563142957	2.01E-46	postive
LINC00472	H3-3A-DT	0.570314637	7.99E-48	postive
ATM	H3-3A-DT	0.724040528	1.14E-88	postive
FBXW7	H3-3A-DT	0.441777544	3.72E-27	postive
PHKG2	AC016027.1	0.539394521	5.13E-42	postive
EPAS1	AC016027.1	-0.500174966	1.83E-35	negative
LINC00472	AP003110.1	0.520072431	1.11E-38	postive
HELLS	ZNF197-AS1	0.405564502	9.33E-23	postive
KLHL24	ZNF197-AS1	0.481110782	1.42E-32	postive
IREB2	ZNF197-AS1	0.401928005	2.41E-22	postive
GABPB1	ZNF197-AS1	0.554502599	8.88E-45	postive
PIK3CA	ZNF197-AS1	0.414810087	7.88E-24	postive
ATG7	ZNF197-AS1	0.459233501	1.79E-29	postive
LINC00472	ZNF197-AS1	0.721090688	1.25E-87	postive
ATM	ZNF197-AS1	0.563091567	2.06E-46	postive
HELLS	L3MBTL2-AS	0.423622726	6.96E-25	postive
ZNF419	L3MBTL2-AS	0.438800321	8.96E-27	postive
ALOX12	L3MBTL2-AS	0.490761455	5.15E-34	postive
GABPB1	L3MBTL2-AS	0.472390183	2.60E-31	postive
ATM	L3MBTL2-AS	0.427071488	2.64E-25	postive
FBXW7	L3MBTL2-AS	0.468121029	1.05E-30	postive
TFAP2C	AC005014.2	0.517795658	2.65E-38	postive
HBA1	AC005014.2	0.425153405	4.53E-25	postive
ALOX12	AC005280.1	0.434167573	3.46E-26	postive
ZEB1	LINC02685	0.586330159	4.41E-51	postive
EPAS1	LINC02685	0.490869486	4.96E-34	postive
PHKG2	AC127537.1	0.449421066	3.73E-28	postive
HELLS	AL513327.2	0.515658748	5.98E-38	postive
ZNF419	AL513327.2	0.444907564	1.46E-27	postive
TUBE1	AL513327.2	0.451564348	1.94E-28	postive
ALOX12	AL513327.2	0.518960801	1.70E-38	postive
GABPB1	AL513327.2	0.559175298	1.16E-45	postive
MAPK8	AL513327.2	0.423844137	6.54E-25	postive
LINC00472	AL513327.2	0.676805822	1.86E-73	postive

ATM	AL513327.2	0.695427953	4.23E-79	postive
FBXW7	AL513327.2	0.482859688	7.84E-33	postive
ZNF419	SUGT1-DT	0.495155667	1.10E-34	postive
TUBE1	SUGT1-DT	0.5197994	1.23E-38	postive
ALOX12	SUGT1-DT	0.483159798	7.08E-33	postive
PHKG2	SUGT1-DT	0.61317502	5.73E-57	postive
TAZ	SUGT1-DT	0.676627239	2.10E-73	postive
HELLS	AL731568.1	0.491492304	3.99E-34	postive
KLHL24	AL731568.1	0.464101789	3.82E-30	postive
IREB2	AL731568.1	0.41345522	1.14E-23	postive
GABPB1	AL731568.1	0.554094188	1.06E-44	postive
PIK3CA	AL731568.1	0.436808362	1.61E-26	postive
ATG7	AL731568.1	0.462772464	5.83E-30	postive
MAPK8	AL731568.1	0.400789429	3.24E-22	postive
LINC00472	AL731568.1	0.806442381	1.25E-124	postive
ATM	AL731568.1	0.692610677	3.22E-78	postive
FBXW7	AL731568.1	0.456053485	4.84E-29	postive
ZNF419	AC006042.1	0.550833798	4.29E-44	postive
TUBE1	AC006042.1	0.424540364	5.38E-25	postive
GABPB1	AC006042.1	0.430735533	9.30E-26	postive
LINC00472	AC006042.1	0.582200797	3.17E-50	postive
LPIN1	AC006042.1	0.520934326	7.95E-39	postive
FANCD2	LINC00539	0.483371501	6.59E-33	postive
HELLS	LINC00539	0.505216376	2.93E-36	postive
TUBE1	LINC00539	0.467562476	1.25E-30	postive
ALOX12	LINC00539	0.563662207	1.60E-46	postive
GABPB1	LINC00539	0.477889524	4.20E-32	postive
IFNG	LINC00539	0.560697839	5.95E-46	postive
ATM	LINC00539	0.57196883	3.75E-48	postive
FBXW7	LINC00539	0.481626015	1.19E-32	postive
MUC1	LINC00511	0.561110137	4.96E-46	postive
SLC7A5	LINC00511	0.554242803	9.93E-45	postive
SLC2A1	LINC00511	0.512985822	1.64E-37	postive
GABPB1	AC009041.3	0.476320675	7.08E-32	postive
LINC00472	AC009041.3	0.447687937	6.32E-28	postive
ATM	AC009041.3	0.433557428	4.13E-26	postive
TUBE1	AC116025.1	0.4185848	2.81E-24	postive
ALOX12	AC116025.1	0.54196184	1.78E-42	postive
ATM	AC116025.1	0.547836146	1.53E-43	postive
TFAP2C	AL136131.2	0.561466319	4.24E-46	postive
HBA1	AL136131.2	0.52710787	7.17E-40	postive
DUOX1	AL136131.2	0.4222321	1.03E-24	postive
ALOX12	LINC02068	0.40082525	3.21E-22	postive
ATM	LINC02068	0.454701802	7.36E-29	postive
ZNF419	AL109659.2	0.490716205	5.23E-34	postive
VEGFA	AL109659.2	0.462392665	6.58E-30	postive
TUBE1	AL109659.2	0.532846583	7.32E-41	postive
SETD1B	AL109659.2	0.438383233	1.01E-26	postive
ALOX12	AL109659.2	0.601513525	2.42E-54	postive
ATM	AL109659.2	0.479438437	2.50E-32	postive
TAZ	AL109659.2	0.501714365	1.05E-35	postive
FBXW7	AL109659.2	0.459893129	1.45E-29	postive
HSPB1	SNHG19	0.462788317	5.80E-30	postive
RPL8	SNHG19	0.495648828	9.22E-35	postive
HBA1	SNHG19	0.631387551	2.70E-61	postive
HRAS	SNHG19	0.769039969	1.89E-106	postive
MAP1LC3A	SNHG19	0.451594572	1.92E-28	postive
EGLN2	SNHG19	0.8614664	3.67E-160	postive

HELLS	AC009268.2	0.498552995	3.27E-35	postive
GABPB1	AC009268.2	0.465653784	2.32E-30	postive
LINC00472	AC009268.2	0.659397728	1.52E-68	postive
ATM	AC009268.2	0.453856603	9.56E-29	postive
HELLS	LINC01545	0.40305208	1.80E-22	postive
TFAP2C	LINC01545	0.408619427	4.16E-23	postive
HSPA5	AC024075.2	-0.455061222	6.59E-29	negative
ZNF419	AC024075.2	0.543020898	1.15E-42	postive
TUBE1	AC024075.2	0.498471626	3.37E-35	postive
ALOX12	AC024075.2	0.47048598	4.85E-31	postive
NCOA4	AC024075.2	-0.422481916	9.57E-25	negative
PHKG2	AC024075.2	0.491896055	3.46E-34	postive
TAZ	AC024075.2	0.562598535	2.57E-46	postive
ZNF419	CSGALNACT:	0.440385374	5.62E-27	postive
ALOX12	CSGALNACT:	0.468834686	8.30E-31	postive
PHKG2	CSGALNACT:	0.613445513	4.96E-57	postive
TAZ	CSGALNACT:	0.688518152	5.89E-77	postive
ZNF419	AC011498.6	0.453494603	1.07E-28	postive
TUBE1	AC011498.6	0.452217024	1.59E-28	postive
SETD1B	AC011498.6	0.411176586	2.10E-23	postive
ALOX12	AC011498.6	0.549111768	8.91E-44	postive
TAZ	AC011498.6	0.487872123	1.41E-33	postive
PHKG2	AC098479.1	0.413606174	1.09E-23	postive
VDAC2	ZFAS1	0.414847445	7.80E-24	postive
RPL8	ZFAS1	0.532727824	7.68E-41	postive
FANCD2	RNF139-AS1	0.41762907	3.66E-24	postive
HELLS	RNF139-AS1	0.530483028	1.88E-40	postive
ZNF419	RNF139-AS1	0.592929245	1.77E-52	postive
TUBE1	RNF139-AS1	0.605228361	3.62E-55	postive
SETD1B	RNF139-AS1	0.437701507	1.24E-26	postive
ALOX12	RNF139-AS1	0.646299714	4.64E-65	postive
GABPB1	RNF139-AS1	0.473725466	1.67E-31	postive
PHKG2	RNF139-AS1	0.520012368	1.13E-38	postive
ATM	RNF139-AS1	0.501991312	9.48E-36	postive
TAZ	RNF139-AS1	0.591126919	4.29E-52	postive
FBXW7	RNF139-AS1	0.455003358	6.71E-29	postive
PROM2	U62317.3	0.638917227	3.60E-63	postive
EGLN2	U62317.3	0.411279393	2.04E-23	postive
VEGFA	AL645608.1	0.561838906	3.59E-46	postive
TUBE1	AL645608.1	0.412030715	1.67E-23	postive
SETD1B	AL645608.1	0.460329835	1.27E-29	postive
ALOX12	AL645608.1	0.468758812	8.51E-31	postive
TAZ	AL645608.1	0.481990248	1.05E-32	postive
NOX1	AL358472.3	0.451293868	2.10E-28	postive
NCOA4	AL358472.3	-0.438149601	1.08E-26	negative
PHKG2	AL358472.3	0.535215306	2.82E-41	postive
TAZ	AL358472.3	0.502232698	8.69E-36	postive
TFAP2C	LINC01985	0.579476014	1.15E-49	postive
HBA1	LINC01985	0.526006364	1.11E-39	postive
TUBE1	ELOA-AS1	0.41078448	2.33E-23	postive
ALOX12	ELOA-AS1	0.531790925	1.12E-40	postive
TAZ	ELOA-AS1	0.415984384	5.73E-24	postive
IL6	AC148477.2	0.42436276	5.66E-25	postive
HSPA5	AC016957.2	-0.425427435	4.19E-25	negative
VDAC2	AC016957.2	-0.440034067	6.23E-27	negative
LAMP2	AC016957.2	-0.405896298	8.55E-23	negative
SRXN1	AC016957.2	-0.410938188	2.24E-23	negative
ZNF419	AC016957.2	0.459201767	1.81E-29	postive

TUBE1	AC016957.2	0.446065263	1.03E-27	postive
ALOX12	AC016957.2	0.403312138	1.68E-22	postive
GABPB1	AC016957.2	0.405640833	9.14E-23	postive
NOX1	AC016957.2	0.401876762	2.45E-22	postive
NCOA4	AC016957.2	-0.425385466	4.24E-25	negative
PHKG2	AC016957.2	0.548227484	1.30E-43	postive
BECN1	AC016957.2	-0.466543573	1.74E-30	negative
TAZ	AC016957.2	0.602986879	1.14E-54	postive
FBXW7	AC016957.2	0.473324552	1.91E-31	postive
LINC00472	ZBED3-AS1	0.483231498	6.91E-33	postive
ZNF419	AC139795.2	0.422054283	1.08E-24	postive
SETD1B	AC139795.2	0.440385714	5.61E-27	postive
ALOX12	AC139795.2	0.451490964	1.98E-28	postive
LINC00472	AC139795.2	0.404726581	1.16E-22	postive
LPIN1	AC139795.2	0.46076677	1.10E-29	postive
ATM	AC139795.2	0.427939294	2.06E-25	postive
HELLS	AC067747.1	0.523889855	2.53E-39	postive
TUBE1	AC067747.1	0.407149803	6.14E-23	postive
ALOX12	AC067747.1	0.527148541	7.05E-40	postive
GABPB1	AC067747.1	0.445919611	1.08E-27	postive
DUOX1	AC067747.1	0.443004495	2.58E-27	postive
ATM	AC067747.1	0.459636942	1.57E-29	postive
FBXW7	AC067747.1	0.4274096	2.40E-25	postive
LINC00472	ITGA6-AS1	0.710551584	5.17E-84	postive
HIC1	AL590226.1	0.402618677	2.02E-22	postive
MAPK3	AL590226.1	0.467069384	1.47E-30	postive
ZEB1	AL590226.1	0.442742057	2.79E-27	postive
EPAS1	AL590226.1	0.524222905	2.22E-39	postive
HELLS	AL354696.1	0.510176761	4.69E-37	postive
ZNF419	AL354696.1	0.408886145	3.87E-23	postive
TUBE1	AL354696.1	0.521848302	5.59E-39	postive
ALOX12	AL354696.1	0.492384306	2.92E-34	postive
GABPB1	AL354696.1	0.4381	1.10E-26	postive
ATM	AL354696.1	0.508036826	1.04E-36	postive
FANCD2	LINC00528	0.434755844	2.92E-26	postive
PML	LINC00528	0.504645696	3.61E-36	postive
ZNF419	LINC00528	0.414806195	7.89E-24	postive
SLC2A6	LINC00528	0.471954897	3.00E-31	postive
ALOX12	LINC00528	0.448947778	4.31E-28	postive
PHKG2	LINC00528	0.429492747	1.33E-25	postive
IFNG	LINC00528	0.451368926	2.06E-28	postive
TAZ	LINC00528	0.526029683	1.10E-39	postive
HELLS	AL133227.1	0.496605322	6.56E-35	postive
KLHL24	AL133227.1	0.404484947	1.24E-22	postive
TUBE1	AL133227.1	0.423547462	7.11E-25	postive
ALOX12	AL133227.1	0.505063174	3.10E-36	postive
IREB2	AL133227.1	0.481530105	1.23E-32	postive
GABPB1	AL133227.1	0.503978979	4.60E-36	postive
PIK3CA	AL133227.1	0.42035662	1.73E-24	postive
MAPK8	AL133227.1	0.470768794	4.42E-31	postive
LINC00472	AL133227.1	0.668621212	4.18E-71	postive
ATM	AL133227.1	0.637802925	6.87E-63	postive
FBXW7	AL133227.1	0.459877295	1.46E-29	postive
ALOX12	AC084125.2	0.405532697	9.41E-23	postive
PHKG2	AC084125.2	0.468781885	8.45E-31	postive
TAZ	AC084125.2	0.56174985	3.74E-46	postive
HELLS	AC004943.1	0.511992836	2.38E-37	postive
KLHL24	AC004943.1	0.484871646	3.95E-33	postive

TUBE1	AC004943.1	0.468203648	1.02E-30	postive
ALOX12	AC004943.1	0.413208126	1.22E-23	postive
IREB2	AC004943.1	0.449061602	4.16E-28	postive
GABPB1	AC004943.1	0.570866882	6.21E-48	postive
PIK3CA	AC004943.1	0.451743307	1.83E-28	postive
ATG7	AC004943.1	0.427805428	2.14E-25	postive
MAPK8	AC004943.1	0.445461562	1.24E-27	postive
LINC00472	AC004943.1	0.834058309	7.94E-141	postive
ATM	AC004943.1	0.714162087	3.11E-85	postive
FBXW7	AC004943.1	0.466379796	1.84E-30	postive
ACSL3	AL450263.1	0.422779219	8.81E-25	postive
KLHL24	AL450263.1	0.655831551	1.41E-67	postive
IREB2	AL450263.1	0.404668498	1.18E-22	postive
PIK3CA	AL450263.1	0.453096089	1.21E-28	postive
KRAS	AL450263.1	0.423710189	6.79E-25	postive
GABARAPL1	AL450263.1	0.532214731	9.43E-41	postive
LINC00472	AL450263.1	0.822027002	2.00E-133	postive
LPIN1	AL450263.1	0.645482841	7.55E-65	postive
HELLS	SMC5-AS1	0.437328236	1.38E-26	postive
ZNF419	SMC5-AS1	0.451539889	1.95E-28	postive
KLHL24	SMC5-AS1	0.526940431	7.66E-40	postive
TUBE1	SMC5-AS1	0.453041732	1.23E-28	postive
IREB2	SMC5-AS1	0.450020609	3.11E-28	postive
GABPB1	SMC5-AS1	0.505979401	2.21E-36	postive
PIK3CA	SMC5-AS1	0.415600234	6.36E-24	postive
ATG7	SMC5-AS1	0.428009942	2.02E-25	postive
MAPK8	SMC5-AS1	0.404102764	1.37E-22	postive
LINC00472	SMC5-AS1	0.687419785	1.27E-76	postive
LPIN1	SMC5-AS1	0.464218923	3.68E-30	postive
ATM	SMC5-AS1	0.617681906	5.17E-58	postive
FBXW7	SMC5-AS1	0.41821304	3.11E-24	postive
FANCD2	LASTR	0.497441517	4.87E-35	postive
JDP2	LASTR	0.510367238	4.37E-37	postive
RGS4	LASTR	0.422376542	9.85E-25	postive
EIF2S1	LASTR	0.447550868	6.58E-28	postive
STMN1	LASTR	0.46699599	1.51E-30	postive
RRM2	LASTR	0.500933241	1.39E-35	postive
AURKA	LASTR	0.49727104	5.18E-35	postive
SLC1A5	LASTR	0.432251125	6.02E-26	postive
CDKN2A	LASTR	0.451473626	1.99E-28	postive
MTOR	AC234775.2	0.500569628	1.59E-35	postive
ACSL3	AC234775.2	0.455817318	5.21E-29	postive
OTUB1	AC234775.2	-0.410148471	2.77E-23	negative
OXSR1	AC234775.2	0.412855746	1.34E-23	postive
KLHL24	AC234775.2	0.701608327	4.53E-81	postive
MAP3K5	AC234775.2	0.521082478	7.51E-39	postive
EIF2AK4	AC234775.2	0.466042656	2.05E-30	postive
IREB2	AC234775.2	0.725104397	4.75E-89	postive
GABPB1	AC234775.2	0.530780959	1.67E-40	postive
EMC2	AC234775.2	0.433764727	3.89E-26	postive
PIK3CA	AC234775.2	0.719420296	4.81E-87	postive
KRAS	AC234775.2	0.569755446	1.03E-47	postive
SLC38A1	AC234775.2	0.444779441	1.52E-27	postive
MAPK8	AC234775.2	0.608028919	8.51E-56	postive
MAPK9	AC234775.2	0.416736259	4.67E-24	postive
LINC00472	AC234775.2	0.795520066	6.24E-119	postive
PRKAA2	AC234775.2	0.571519748	4.61E-48	postive
PRKAA1	AC234775.2	0.637336476	9.00E-63	postive

TGFBR1	AC234775.2	0.483927242	5.45E-33	postive
LPIN1	AC234775.2	0.423095439	8.06E-25	postive
TLR4	AC234775.2	0.425982131	3.59E-25	postive
ATM	AC234775.2	0.707293889	6.28E-83	postive
FBXW7	AC234775.2	0.449040709	4.19E-28	postive
BACH1	AC234775.2	0.45456269	7.69E-29	postive
FANCD2	AC079907.1	0.425265746	4.39E-25	postive
TFAP2C	AC079907.1	0.54023968	3.63E-42	postive
HBA1	AC079907.1	0.458261965	2.43E-29	postive
TFAP2C	AL022313.2	0.419665161	2.09E-24	postive
ALOX12	ARHGAP42-/	0.411010974	2.20E-23	postive
SRC	SBNO1-AS1	0.422224224	1.03E-24	postive
NCOA4	SBNO1-AS1	-0.411281495	2.04E-23	negative
PHKG2	SBNO1-AS1	0.662568227	2.05E-69	postive
TAZ	SBNO1-AS1	0.620299523	1.26E-58	postive
ZNF419	DPP9-AS1	0.435396131	2.42E-26	postive
TUBE1	DPP9-AS1	0.465854667	2.18E-30	postive
DRD4	DPP9-AS1	0.436838644	1.59E-26	postive
ALOX12	DPP9-AS1	0.613120852	5.89E-57	postive
PHKG2	DPP9-AS1	0.486731485	2.08E-33	postive
TAZ	DPP9-AS1	0.551416219	3.34E-44	postive
FBXW7	DPP9-AS1	0.425520515	4.09E-25	postive
HELLS	AC009095.1	0.556854897	3.20E-45	postive
ZNF419	AC009095.1	0.439441884	7.42E-27	postive
TUBE1	AC009095.1	0.484778291	4.08E-33	postive
ALOX12	AC009095.1	0.586958053	3.26E-51	postive
GABPB1	AC009095.1	0.495759829	8.86E-35	postive
DUOX1	AC009095.1	0.406038035	8.23E-23	postive
LINC00472	AC009095.1	0.511691005	2.67E-37	postive
ATM	AC009095.1	0.556684574	3.45E-45	postive
FBXW7	AC009095.1	0.51941231	1.43E-38	postive
ZNF419	AL353803.5	0.450158315	2.98E-28	postive
TUBE1	AL353803.5	0.435288007	2.50E-26	postive
ALOX12	AL353803.5	0.561524758	4.13E-46	postive
GABPB1	AL353803.5	0.421849802	1.14E-24	postive
ATM	AL353803.5	0.435097873	2.64E-26	postive
ZNF419	AL118558.4	0.431069251	8.45E-26	postive
ALOX12	AL118558.4	0.428734767	1.65E-25	postive
PHKG2	AL118558.4	0.430318651	1.05E-25	postive
ATG4D	AL118558.4	0.422560623	9.36E-25	postive
ANO6	AL118558.4	-0.417751652	3.53E-24	negative
TAZ	AL118558.4	0.504379015	3.98E-36	postive
ALOX12	AC104596.1	0.426169484	3.40E-25	postive
IREB2	AC104596.1	0.488568078	1.10E-33	postive
MAPK8	AC104596.1	0.420468814	1.67E-24	postive
PRKAA2	AC104596.1	0.411264061	2.05E-23	postive
ATM	AC104596.1	0.599597307	6.38E-54	postive
SIRT1	AC104596.1	0.403655393	1.54E-22	postive
HELLS	AL137847.2	0.520286866	1.02E-38	postive
TUBE1	AL137847.2	0.467093349	1.46E-30	postive
ALOX12	AL137847.2	0.485931439	2.74E-33	postive
GABPB1	AL137847.2	0.492550765	2.75E-34	postive
LINC00472	AL137847.2	0.464848137	3.01E-30	postive
ATM	AL137847.2	0.68429062	1.13E-75	postive
FBXW7	AL137847.2	0.432649096	5.37E-26	postive
FANCD2	AL731569.1	0.436884566	1.57E-26	postive
HELLS	AL731569.1	0.433457214	4.25E-26	postive
ZNF419	AL731569.1	0.495123303	1.11E-34	postive

TUBE1	AL731569.1	0.454753299	7.25E-29	postive
ALOX12	AL731569.1	0.621965481	5.07E-59	postive
PHKG2	AL731569.1	0.408992361	3.77E-23	postive
TAZ	AL731569.1	0.487988026	1.35E-33	postive
FANCD2	AL354989.1	0.441497191	4.04E-27	postive
HELLS	AL354989.1	0.485357403	3.34E-33	postive
ZNF419	AL354989.1	0.447673767	6.34E-28	postive
TUBE1	AL354989.1	0.459275802	1.76E-29	postive
ALOX12	AL354989.1	0.525788867	1.20E-39	postive
GABPB1	AL354989.1	0.490293152	6.06E-34	postive
LINC00472	AL354989.1	0.57012592	8.70E-48	postive
ATM	AL354989.1	0.474774705	1.18E-31	postive
FBXW7	AL354989.1	0.473856676	1.60E-31	postive
IL6	LINC01503	0.424813479	4.98E-25	postive
EPAS1	AC002070.1	0.437664036	1.25E-26	postive
HELLS	AC006441.1	0.576783318	4.06E-49	postive
TUBE1	AC006441.1	0.465773357	2.23E-30	postive
ALOX12	AC006441.1	0.534455771	3.83E-41	postive
GABPB1	AC006441.1	0.519839799	1.21E-38	postive
ATG7	AC006441.1	0.428049391	2.00E-25	postive
LINC00472	AC006441.1	0.56189288	3.51E-46	postive
ATM	AC006441.1	0.695581134	3.78E-79	postive
FBXW7	AC006441.1	0.488229103	1.24E-33	postive
VEGFA	AC092118.2	0.488077903	1.31E-33	postive
TUBE1	AC092118.2	0.457025572	3.57E-29	postive
DRD4	AC092118.2	0.407689217	5.32E-23	postive
ALOX12	AC092118.2	0.499568168	2.27E-35	postive
PHKG2	AC092118.2	0.56680021	3.92E-47	postive
TAZ	AC092118.2	0.690948474	1.05E-77	postive
ENPP2	AC104211.1	0.406307149	7.67E-23	postive
SLC1A4	AC104211.1	0.486690371	2.11E-33	postive
MAPK3	AC104211.1	0.414051296	9.68E-24	postive
ZEB1	AC104211.1	0.649310287	7.60E-66	postive
MAPK8	AC104211.1	0.480320508	1.85E-32	postive
EPAS1	AC104211.1	0.681923023	5.77E-75	postive
MTOR	AP000866.1	0.432048212	6.38E-26	postive
ZNF419	AP000866.1	0.471394123	3.60E-31	postive
KLHL24	AP000866.1	0.461927184	7.63E-30	postive
TUBE1	AP000866.1	0.440264774	5.82E-27	postive
ALOX12	AP000866.1	0.487188949	1.78E-33	postive
IREB2	AP000866.1	0.527896226	5.25E-40	postive
GABPB1	AP000866.1	0.449597249	3.54E-28	postive
PIK3CA	AP000866.1	0.430298139	1.05E-25	postive
ULK2	AP000866.1	0.440812242	4.95E-27	postive
MAPK8	AP000866.1	0.487549125	1.57E-33	postive
LINC00472	AP000866.1	0.426249908	3.33E-25	postive
PRKAA1	AP000866.1	0.405663376	9.09E-23	postive
LPIN1	AP000866.1	0.447113653	7.51E-28	postive
ATM	AP000866.1	0.548895247	9.77E-44	postive
HELLS	AL606534.1	0.559507972	1.00E-45	postive
ZNF419	AL606534.1	0.453337721	1.12E-28	postive
TUBE1	AL606534.1	0.589754309	8.39E-52	postive
ALOX12	AL606534.1	0.543903711	7.95E-43	postive
GABPB1	AL606534.1	0.566901815	3.75E-47	postive
ATM	AL606534.1	0.653895834	4.64E-67	postive
FBXW7	AL606534.1	0.547085566	2.10E-43	postive
HELLS	AC007038.1	0.611459778	1.42E-56	postive
ZNF419	AC007038.1	0.521330405	6.83E-39	postive

TUBE1	AC007038.1	0.599381148	7.11E-54	postive
ALOX12	AC007038.1	0.635181418	3.11E-62	postive
GABPB1	AC007038.1	0.560409142	6.75E-46	postive
LINC00472	AC007038.1	0.506600376	1.76E-36	postive
ATM	AC007038.1	0.591218647	4.11E-52	postive
TAZ	AC007038.1	0.450829756	2.43E-28	postive
FBXW7	AC007038.1	0.550701183	4.53E-44	postive
HELLS	AC006064.2	0.528179767	4.70E-40	postive
KLHL24	AC006064.2	0.435622384	2.27E-26	postive
TUBE1	AC006064.2	0.44431581	1.74E-27	postive
IREB2	AC006064.2	0.426184991	3.39E-25	postive
GABPB1	AC006064.2	0.572475844	2.97E-48	postive
PIK3CA	AC006064.2	0.418796349	2.65E-24	postive
KRAS	AC006064.2	0.402553327	2.05E-22	postive
MAPK8	AC006064.2	0.429759125	1.23E-25	postive
LINC00472	AC006064.2	0.817155539	1.38E-130	postive
ATM	AC006064.2	0.643730605	2.14E-64	postive
FBXW7	AC006064.2	0.42907428	1.49E-25	postive
PEBP1	SMIM2-AS1	0.418608686	2.79E-24	postive
MTOR	AC024075.1	0.510372111	4.36E-37	postive
ZNF419	AC024075.1	0.492109778	3.21E-34	postive
KLHL24	AC024075.1	0.528759019	3.73E-40	postive
TUBE1	AC024075.1	0.450509914	2.68E-28	postive
SETD1B	AC024075.1	0.503821287	4.88E-36	postive
MAP3K5	AC024075.1	0.429534047	1.31E-25	postive
ALOX12	AC024075.1	0.480495944	1.75E-32	postive
IREB2	AC024075.1	0.582862217	2.32E-50	postive
GABPB1	AC024075.1	0.453167368	1.18E-28	postive
PRDX1	AC024075.1	-0.407212836	6.04E-23	negative
PIK3CA	AC024075.1	0.46829722	9.89E-31	postive
MAPK8	AC024075.1	0.48616599	2.53E-33	postive
LINC00472	AC024075.1	0.57553158	7.26E-49	postive
PRKAA1	AC024075.1	0.411500959	1.93E-23	postive
LPIN1	AC024075.1	0.474943891	1.12E-31	postive
ATM	AC024075.1	0.627186352	2.85E-60	postive
FBXW7	AC024075.1	0.419525211	2.17E-24	postive
HELLS	AL445248.1	0.519636078	1.31E-38	postive
TUBE1	AL445248.1	0.414915229	7.66E-24	postive
ALOX12	AL445248.1	0.438187976	1.07E-26	postive
GABPB1	AL445248.1	0.543294714	1.02E-42	postive
LINC00472	AL445248.1	0.682161277	4.90E-75	postive
ATM	AL445248.1	0.651965613	1.51E-66	postive
FBXW7	AL445248.1	0.495821494	8.67E-35	postive
JDP2	AL161421.1	0.45000087	3.13E-28	postive
HELLS	SYNE1-AS1	0.451053049	2.27E-28	postive
ATM	SYNE1-AS1	0.517222914	3.30E-38	postive
FBXW7	SYNE1-AS1	0.431355277	7.79E-26	postive
ALOX12	CYP4A22-AS	0.412404526	1.51E-23	postive
JDP2	LINC02154	0.583354164	1.83E-50	postive
RGS4	LINC02154	0.468561793	9.07E-31	postive
EIF2S1	LINC02154	0.47297607	2.14E-31	postive
SLC1A5	LINC02154	0.422207826	1.03E-24	postive
VEGFA	STPG3-AS1	0.441717021	3.78E-27	postive
HELLS	AC008770.3	0.512195432	2.21E-37	postive
MTOR	AC008770.3	0.418800219	2.65E-24	postive
ZNF419	AC008770.3	0.508709422	8.09E-37	postive
KLHL24	AC008770.3	0.528243567	4.58E-40	postive
TUBE1	AC008770.3	0.552097688	2.50E-44	postive

ALOX12	AC008770.3	0.546929698	2.24E-43	postive
IREB2	AC008770.3	0.525567254	1.31E-39	postive
GABPB1	AC008770.3	0.610494668	2.35E-56	postive
PIK3CA	AC008770.3	0.482747649	8.15E-33	postive
ATG7	AC008770.3	0.428706431	1.66E-25	postive
MAPK8	AC008770.3	0.530697145	1.73E-40	postive
LINC00472	AC008770.3	0.792265147	2.67E-117	postive
PRKAA1	AC008770.3	0.403912678	1.44E-22	postive
LPIN1	AC008770.3	0.425447298	4.17E-25	postive
ATM	AC008770.3	0.754961039	1.79E-100	postive
FBXW7	AC008770.3	0.545517209	4.05E-43	postive
TUBE1	AL645933.2	0.443360301	2.32E-27	postive
ALOX12	AL645933.2	0.481961949	1.06E-32	postive
HELLS	AC018809.1	0.417875128	3.42E-24	postive
ZNF419	AC018809.1	0.555482684	5.81E-45	postive
TUBE1	AC018809.1	0.551580722	3.12E-44	postive
ALOX12	AC018809.1	0.51774094	2.71E-38	postive
GABPB1	AC018809.1	0.440355587	5.66E-27	postive
PHKG2	AC018809.1	0.496163145	7.68E-35	postive
LINC00472	AC018809.1	0.487465844	1.62E-33	postive
ATM	AC018809.1	0.441389891	4.17E-27	postive
TAZ	AC018809.1	0.584474193	1.07E-50	postive
FBXW7	AC018809.1	0.443424415	2.28E-27	postive
TUBE1	TNS1-AS1	0.422694645	9.02E-25	postive
ATM	AC079766.1	0.412015936	1.68E-23	postive
TFAP2C	AC068888.2	0.54382326	8.22E-43	postive
HBA1	AC068888.2	0.504570629	3.71E-36	postive
DUOX1	AC068888.2	0.445371517	1.27E-27	postive
CHMP6	AC073508.3	0.428606047	1.71E-25	postive
KLHL24	ATP1A1-AS1	0.411348687	2.01E-23	postive
TUBE1	ATP1A1-AS1	0.4753196	9.88E-32	postive
ALOX12	ATP1A1-AS1	0.515938886	5.38E-38	postive
IREB2	ATP1A1-AS1	0.485722226	2.95E-33	postive
GABPB1	ATP1A1-AS1	0.445140742	1.36E-27	postive
MAPK8	ATP1A1-AS1	0.551195377	3.67E-44	postive
LINC00472	ATP1A1-AS1	0.583163712	2.01E-50	postive
PRKAA2	ATP1A1-AS1	0.478549702	3.36E-32	postive
ATM	ATP1A1-AS1	0.649035828	8.97E-66	postive
TFAP2C	AC112491.1	0.541366054	2.28E-42	postive
HBA1	AC112491.1	0.562754321	2.39E-46	postive
HELLS	AC139887.2	0.483631722	6.03E-33	postive
MTOR	AC139887.2	0.403102177	1.78E-22	postive
ZNF419	AC139887.2	0.520012503	1.13E-38	postive
TUBE1	AC139887.2	0.553676103	1.27E-44	postive
SETD1B	AC139887.2	0.452017051	1.69E-28	postive
ALOX12	AC139887.2	0.674732056	7.46E-73	postive
GABPB1	AC139887.2	0.540385392	3.41E-42	postive
MAPK8	AC139887.2	0.429070841	1.50E-25	postive
LINC00472	AC139887.2	0.524576342	1.93E-39	postive
ATM	AC139887.2	0.705756326	2.02E-82	postive
FBXW7	AC139887.2	0.507368214	1.33E-36	postive
HELLS	AP000873.1	0.522435211	4.45E-39	postive
ZNF419	AP000873.1	0.51820271	2.27E-38	postive
TUBE1	AP000873.1	0.651230668	2.37E-66	postive
ALOX12	AP000873.1	0.67288861	2.54E-72	postive
GABPB1	AP000873.1	0.583625543	1.61E-50	postive
MAPK8	AP000873.1	0.430777584	9.19E-26	postive
LINC00472	AP000873.1	0.649049194	8.90E-66	postive

ATM	AP000873.1	0.775727093	1.94E-109	postive
FBXW7	AP000873.1	0.508126366	1.00E-36	postive
MAPK1	AC073346.1	0.42722359	2.53E-25	postive
HELLS	AL355075.2	0.471618005	3.35E-31	postive
ZNF419	AL355075.2	0.577511635	2.89E-49	postive
VEGFA	AL355075.2	0.446231634	9.81E-28	postive
TUBE1	AL355075.2	0.626196646	4.94E-60	postive
SETD1B	AL355075.2	0.48353106	6.24E-33	postive
ALOX12	AL355075.2	0.698112748	5.97E-80	postive
GABPB1	AL355075.2	0.592836372	1.85E-52	postive
MAPK8	AL355075.2	0.470545698	4.76E-31	postive
LINC00472	AL355075.2	0.610493556	2.35E-56	postive
ATM	AL355075.2	0.703359916	1.23E-81	postive
FBXW7	AL355075.2	0.501164814	1.28E-35	postive
HELLS	AC073912.2	0.468296849	9.89E-31	postive
TUBE1	AC073912.2	0.494669597	1.30E-34	postive
ALOX12	AC073912.2	0.586551228	3.96E-51	postive
GABPB1	AC073912.2	0.497342852	5.05E-35	postive
LINC00472	AC073912.2	0.400979731	3.09E-22	postive
ATM	AC073912.2	0.686664325	2.16E-76	postive
FBXW7	AC073912.2	0.461634576	8.37E-30	postive
ATG4D	MIR200CHG	0.416722051	4.68E-24	postive
GABARAPL2	MIR200CHG	0.570973808	5.91E-48	postive
HIF1A	LINC02693	0.466608152	1.71E-30	postive
ANGPTL7	LINC02693	0.411642296	1.85E-23	postive
AURKA	LINC02693	0.410917833	2.25E-23	postive
BRD4	MMP25-AS1	0.426390999	3.20E-25	postive
ZNF419	MMP25-AS1	0.400037196	3.94E-22	postive
VEGFA	MMP25-AS1	0.427563061	2.30E-25	postive
TUBE1	MMP25-AS1	0.449865758	3.26E-28	postive
SETD1B	MMP25-AS1	0.485741138	2.93E-33	postive
DRD4	MMP25-AS1	0.468537451	9.15E-31	postive
ALOX12	MMP25-AS1	0.596105057	3.67E-53	postive
PHKG2	MMP25-AS1	0.543082867	1.12E-42	postive
YY1AP1	MMP25-AS1	0.417101502	4.22E-24	postive
TAZ	MMP25-AS1	0.67235903	3.60E-72	postive
FANCD2	SNHG10	0.4616125	8.43E-30	postive
ZNF419	SNHG10	0.566129834	5.30E-47	postive
TUBE1	SNHG10	0.427184436	2.56E-25	postive
ALOX12	SNHG10	0.41498097	7.53E-24	postive
GABPB1	SNHG10	0.403755169	1.50E-22	postive
PHKG2	SNHG10	0.477364895	5.00E-32	postive
TAZ	SNHG10	0.656354173	1.02E-67	postive
TUBE1	AC093915.1	0.464838648	3.01E-30	postive
ALOX12	AC093915.1	0.519907436	1.18E-38	postive
TAZ	AC093915.1	0.498094893	3.86E-35	postive
SLC7A5	AC061992.2	0.403178041	1.74E-22	postive
TRIB3	AC061992.2	0.400079213	3.90E-22	postive
SLC2A6	AC061992.2	0.400216265	3.76E-22	postive
G6PD	AC061992.2	0.471505014	3.48E-31	postive
KLHL24	Z82243.1	0.550396102	5.16E-44	postive
IREB2	Z82243.1	0.422425784	9.72E-25	postive
GABPB1	Z82243.1	0.516038365	5.18E-38	postive
PIK3CA	Z82243.1	0.448574715	4.83E-28	postive
KRAS	Z82243.1	0.412672075	1.41E-23	postive
LINC00472	Z82243.1	0.939935493	7.82E-253	postive
LPIN1	Z82243.1	0.438452862	9.92E-27	postive
ATM	Z82243.1	0.532099208	9.88E-41	postive

VEGFA	AC018730.1	0.558381928	1.64E-45	postive
ALOX12	AC018730.1	0.450009796	3.12E-28	postive
SP1	AC018730.1	0.401511253	2.69E-22	postive
YY1AP1	AC018730.1	0.469539066	6.60E-31	postive
KLHL24	ARAP1-AS2	0.610051599	2.96E-56	postive
IREB2	ARAP1-AS2	0.469378262	6.96E-31	postive
GABPB1	ARAP1-AS2	0.518006772	2.45E-38	postive
PIK3CA	ARAP1-AS2	0.514691055	8.63E-38	postive
KRAS	ARAP1-AS2	0.419512181	2.18E-24	postive
MAPK8	ARAP1-AS2	0.402955598	1.85E-22	postive
LINC00472	ARAP1-AS2	0.943165554	4.37E-259	postive
LPIN1	ARAP1-AS2	0.439916447	6.45E-27	postive
ATM	ARAP1-AS2	0.603356567	9.46E-55	postive
FBXW7	ARAP1-AS2	0.417193083	4.12E-24	postive
HELLS	LINC02614	0.554252081	9.89E-45	postive
ZNF419	LINC02614	0.463657908	4.40E-30	postive
TUBE1	LINC02614	0.601251601	2.76E-54	postive
ALOX12	LINC02614	0.629034696	1.01E-60	postive
GABPB1	LINC02614	0.535190692	2.85E-41	postive
LINC00472	LINC02614	0.536949644	1.39E-41	postive
ATM	LINC02614	0.66137099	4.38E-69	postive
FBXW7	LINC02614	0.468996985	7.88E-31	postive
BRD4	AC027796.4	0.464570367	3.29E-30	postive
ZNF419	AC027796.4	0.43835553	1.02E-26	postive
DRD4	AC027796.4	0.407300541	5.90E-23	postive
ALOX12	AC027796.4	0.480057398	2.03E-32	postive
PHKG2	AC027796.4	0.657883712	3.92E-68	postive
TAZ	AC027796.4	0.704689974	4.51E-82	postive
HELLS	AC087683.2	0.443099223	2.51E-27	postive
KLHL24	AC087683.2	0.545945662	3.39E-43	postive
IREB2	AC087683.2	0.41475652	8.00E-24	postive
GABPB1	AC087683.2	0.552616886	2.00E-44	postive
PIK3CA	AC087683.2	0.459659588	1.56E-29	postive
MAPK8	AC087683.2	0.402566183	2.04E-22	postive
LINC00472	AC087683.2	0.906336739	4.78E-203	postive
ATM	AC087683.2	0.581407729	4.62E-50	postive
FBXW7	AC087683.2	0.425712474	3.87E-25	postive
HELLS	AL807757.2	0.481585856	1.21E-32	postive
ALOX12	AL807757.2	0.421934924	1.11E-24	postive
TFAP2C	AL807757.2	0.422063221	1.07E-24	postive
GABPB1	AL807757.2	0.432814131	5.12E-26	postive
DUOX1	AL807757.2	0.4114285	1.96E-23	postive
LINC00472	AL807757.2	0.530747105	1.70E-40	postive
TUBE1	BDNF-AS	0.464641328	3.21E-30	postive
MAPK8	BDNF-AS	0.402630421	2.01E-22	postive
HELLS	TPT1-AS1	0.475660666	8.82E-32	postive
BRD4	TPT1-AS1	0.416217755	5.38E-24	postive
ZNF419	TPT1-AS1	0.534615953	3.59E-41	postive
VEGFA	TPT1-AS1	0.421641235	1.21E-24	postive
TUBE1	TPT1-AS1	0.587650046	2.33E-51	postive
ALOX12	TPT1-AS1	0.70271275	1.99E-81	postive
GABPB1	TPT1-AS1	0.455907865	5.06E-29	postive
ATM	TPT1-AS1	0.447757988	6.18E-28	postive
TAZ	TPT1-AS1	0.471365933	3.64E-31	postive
FBXW7	TPT1-AS1	0.420187009	1.81E-24	postive
ZNF419	AC079807.1	0.483571257	6.16E-33	postive
TUBE1	AC079807.1	0.456316906	4.46E-29	postive
SETD1B	AC079807.1	0.474407943	1.34E-31	postive

ALOX12	AC079807.1	0.595394989	5.23E-53	postive
TAZ	AC079807.1	0.432626231	5.40E-26	postive
HELLS	AC003101.2	0.477371924	4.99E-32	postive
ALOX12	AC003101.2	0.440636309	5.21E-27	postive
TAZ	AC003101.2	0.449149516	4.05E-28	postive
FBXW7	AC003101.2	0.463659568	4.40E-30	postive
HELLS	AP002490.1	0.472634411	2.40E-31	postive
ZNF419	AP002490.1	0.545113753	4.80E-43	postive
VEGFA	AP002490.1	0.405806072	8.75E-23	postive
TUBE1	AP002490.1	0.651708372	1.77E-66	postive
SETD1B	AP002490.1	0.435861437	2.12E-26	postive
ALOX12	AP002490.1	0.682055021	5.27E-75	postive
GABPB1	AP002490.1	0.513738986	1.24E-37	postive
LINC00472	AP002490.1	0.563709458	1.57E-46	postive
ATM	AP002490.1	0.573942024	1.51E-48	postive
TAZ	AP002490.1	0.426845013	2.81E-25	postive
FBXW7	AP002490.1	0.515109551	7.37E-38	postive
OTUB1	AP002748.4	0.453089853	1.21E-28	postive
TFAP2C	AC231981.1	0.407396715	5.75E-23	postive
HELLS	AC008771.1	0.43674371	1.64E-26	postive
STMN1	LINC02195	0.482101381	1.01E-32	postive
RRM2	LINC02195	0.458695659	2.12E-29	postive
AURKA	LINC02195	0.418481646	2.89E-24	postive
CDKN2A	LINC02195	0.426271001	3.31E-25	postive
IFNG	LINC02195	0.426788294	2.86E-25	postive
PML	NNT-AS1	-0.458377323	2.34E-29	negative
FH	NNT-AS1	0.418669113	2.75E-24	postive
ISCU	NNT-AS1	0.529007907	3.38E-40	postive
ACSL3	NNT-AS1	0.476704333	6.23E-32	postive
KLHL24	NNT-AS1	0.636032085	1.91E-62	postive
SLC2A12	NNT-AS1	0.427797658	2.15E-25	postive
IREB2	NNT-AS1	0.480125383	1.98E-32	postive
CS	NNT-AS1	0.408421266	4.38E-23	postive
PIK3CA	NNT-AS1	0.409360088	3.41E-23	postive
KRAS	NNT-AS1	0.404857633	1.12E-22	postive
GOT1	NNT-AS1	0.496656867	6.44E-35	postive
GABARAPL2	NNT-AS1	0.427893273	2.09E-25	postive
GABARAPL1	NNT-AS1	0.594130203	9.79E-53	postive
WIPI2	NNT-AS1	0.422922516	8.46E-25	postive
LINC00472	NNT-AS1	0.416493321	4.99E-24	postive
PRKAA2	NNT-AS1	0.400809449	3.23E-22	postive
PRKAA1	NNT-AS1	0.456478979	4.24E-29	postive
LPIN1	NNT-AS1	0.545101125	4.82E-43	postive
TAZ	AC112484.3	0.425676155	3.91E-25	postive
HELLS	LINC00216	0.465847374	2.18E-30	postive
KLHL24	LINC00216	0.505413878	2.72E-36	postive
TUBE1	LINC00216	0.413309755	1.18E-23	postive
IREB2	LINC00216	0.446452548	9.17E-28	postive
GABPB1	LINC00216	0.560907189	5.42E-46	postive
PIK3CA	LINC00216	0.468238928	1.01E-30	postive
ATG7	LINC00216	0.404700312	1.17E-22	postive
MAPK8	LINC00216	0.432573836	5.49E-26	postive
LINC00472	LINC00216	0.891818038	3.97E-187	postive
ATM	LINC00216	0.667323777	9.70E-71	postive
FBXW7	LINC00216	0.44342001	2.28E-27	postive
ZNF419	AL158835.2	0.492347757	2.96E-34	postive
VEGFA	AL158835.2	0.42024602	1.78E-24	postive
TUBE1	AL158835.2	0.517264441	3.25E-38	postive

SETD1B	AL158835.2	0.505897525	2.28E-36	postive
ALOX12	AL158835.2	0.640685752	1.28E-63	postive
GABPB1	AL158835.2	0.449452836	3.69E-28	postive
ATM	AL158835.2	0.562196524	3.07E-46	postive
FBXW7	AL158835.2	0.410709235	2.38E-23	postive
FANCD2	LINC01711	0.47633762	7.04E-32	postive
STMN1	LINC01711	0.577014739	3.64E-49	postive
RRM2	LINC01711	0.553846351	1.18E-44	postive
AURKA	LINC01711	0.569450876	1.18E-47	postive
SLC1A5	LINC01711	0.400288226	3.69E-22	postive
CDKN2A	LINC01711	0.693444205	1.77E-78	postive
ZNF419	AL161452.1	0.464811889	3.04E-30	postive
TUBE1	AL161452.1	0.411894088	1.73E-23	postive
DRD4	AL161452.1	0.417811729	3.48E-24	postive
ALOX12	AL161452.1	0.432742187	5.23E-26	postive
NCOA4	AL161452.1	-0.417771941	3.51E-24	negative
PHKG2	AL161452.1	0.539832086	4.29E-42	postive
TAZ	AL161452.1	0.677931404	8.72E-74	postive
MTOR	AP000759.1	0.439969159	6.35E-27	postive
HELLS	NCBP2-AS1	0.516489555	4.36E-38	postive
ZNF419	NCBP2-AS1	0.528467762	4.19E-40	postive
TUBE1	NCBP2-AS1	0.508608649	8.40E-37	postive
SETD1B	NCBP2-AS1	0.401950296	2.40E-22	postive
ALOX12	NCBP2-AS1	0.610399147	2.47E-56	postive
GABPB1	NCBP2-AS1	0.444042426	1.89E-27	postive
ATM	NCBP2-AS1	0.578632318	1.71E-49	postive
FBXW7	NCBP2-AS1	0.461657379	8.31E-30	postive

Table S5 Univariate and Multivariate COX analysis

Univariate COX analysis

**LncRNA**	HR	HR.95L	HR.95H	pvalue
PARD3-AS1	1.156956574	1.063441358	1.258695183	0.000698004
OGFR-AS1	2.415704743	1.67304976	3.488019033	2.53E-06
AC010809.2	2.368570158	1.71047858	3.279856677	2.08E-07
AL031714.1	1.482713979	1.210272869	1.816483538	0.000143346
AC110285.2	1.108080459	1.0470459	1.172672853	0.000384735
AL031600.1	1.345829865	1.156102738	1.566692966	0.00012767
AC017104.1	2.06657381	1.555284591	2.745945878	5.57E-07
AC010883.1	1.226150901	1.121249613	1.340866489	7.90E-06
AC093458.1	1.994361041	1.42530778	2.790608469	5.64E-05
SNHG17	1.148672693	1.100512498	1.198940455	2.26E-10
LINC00941	1.412990083	1.271104997	1.570712868	1.52E-10
AC138150.2	1.578821056	1.266431178	1.968267972	4.91E-05
RRN3P2	2.242709163	1.640591319	3.065811902	4.11E-07
AC107081.1	1.421554461	1.17576052	1.718731877	0.000281603
AC087239.1	1.56677586	1.302629408	1.88448578	1.87E-06
AL121782.1	3.038852329	2.129196236	4.337140617	9.14E-10
LINC01929	1.131305621	1.05606255	1.211909662	0.000442513
AL442125.1	4.891377394	2.701614413	8.85602797	1.59E-07
AL513320.1	1.274759117	1.135785864	1.430736952	3.76E-05
OCIAD1-AS1	1.68650032	1.400533729	2.030856716	3.52E-08
AC068722.2	1.992814017	1.475863044	2.690837556	6.78E-06
AL021707.8	1.225599089	1.108384081	1.355209942	7.30E-05
AL031705.1	3.288222284	1.844600613	5.861651411	5.44E-05
ARHGAP27P1-BPTFP1-KPNA2P3	1.277056728	1.135757898	1.435934444	4.36E-05
AL008582.1	1.260571594	1.10897226	1.432894943	0.000396899
AC025171.5	2.150782225	1.629919243	2.838094095	6.20E-08
AC232271.1	1.437979157	1.248345356	1.656419873	4.80E-07
LINC00894	1.922977853	1.556325904	2.376008659	1.38E-09
AL451050.2	4.2150234	2.722786866	6.525087396	1.10E-10
AC006017.1	1.457901005	1.167259103	1.820911344	0.00088957
APP-DT	0.216842711	0.10735836	0.437979506	2.03E-05
AC116407.2	1.30193312	1.171928061	1.446359982	8.84E-07
HM13-IT1	1.32233038	1.18552129	1.474927232	5.33E-07
AC012615.6	1.477457582	1.226045333	1.780424302	4.11E-05
AL096865.1	1.741478126	1.431304994	2.118867799	2.97E-08
AC108134.3	1.251636633	1.1247838	1.392795898	3.84E-05
LINC00115	1.612913155	1.330621913	1.955092443	1.12E-06
AC011462.4	1.205178398	1.12659907	1.289238567	5.79E-08
AC025766.1	1.492889517	1.177182437	1.893265683	0.000947625
AC093281.2	1.439140751	1.17282026	1.765936496	0.000488868
AC147067.1	1.285095108	1.137941554	1.451277907	5.29E-05
AC008750.1	1.893700001	1.412024143	2.539687238	2.01E-05
Z98200.1	4.738416932	2.846472832	7.88786556	2.19E-09
AC108673.2	1.744397808	1.339400458	2.271855063	3.66E-05
AC012645.4	2.042529248	1.540373959	2.708385004	7.02E-07
AC016737.1	2.3974779	1.761583966	3.262915871	2.69E-08
AC006272.1	2.904129837	1.845915022	4.568991536	4.00E-06
RUSC1-AS1	1.301342547	1.155579711	1.465491655	1.39E-05
CEP250-AS1	2.262699712	1.531781266	3.34238974	4.09E-05
AL445222.1	1.793616429	1.435950034	2.24037036	2.62E-07
RHOQ-AS1	2.5861956	1.69826982	3.938365745	9.51E-06
PIK3IP1-DT	1.287858302	1.119256004	1.481858484	0.000409822
H1-10-AS1	1.963934015	1.448187092	2.663355334	1.41E-05
AC008610.1	1.139238643	1.082024884	1.199477668	7.10E-07

AC078906.1	1.622045799	1.228785484	2.141165084	0.000639543
AC084824.3	2.106790239	1.465681668	3.028328189	5.69E-05
AC097641.2	2.633286061	1.883615496	3.681322167	1.48E-08
AC083967.1	1.054081171	1.032344329	1.076275699	7.27E-07
AC004816.1	1.105272456	1.041335226	1.173135387	0.00099404
DTX2P1-UPK3BP1-PMS2P11	2.978394722	1.688285663	5.254344874	0.000164456
AC018638.7	1.422333197	1.186901237	1.704465088	0.000135702
HOTAIR	1.393325833	1.221701568	1.589059822	7.59E-07
AC100803.4	1.433108012	1.248316557	1.645254612	3.24E-07
TMEM92-AS1	1.537634001	1.323072314	1.786991003	2.01E-08
AC026401.3	1.225137804	1.168178135	1.284874793	6.30E-17
MRPS9-AS1	2.533800466	1.69899598	3.778787518	5.14E-06
AC008875.1	1.740443002	1.44509457	2.096154747	5.21E-09
AC245884.8	1.178067842	1.094944753	1.267501247	1.14E-05
AC015961.2	1.576684742	1.269845176	1.957667613	3.73E-05
AP003352.1	1.325393576	1.194848447	1.470201628	1.01E-07
AC006480.2	1.285676901	1.107206221	1.492915288	0.000982167
AP001001.1	2.459722472	1.44831188	4.177439073	0.000866527
SNHG15	1.07369191	1.042821396	1.105476281	1.78E-06
AC005332.5	1.363972273	1.199022018	1.551614844	2.36E-06
AC110015.1	1.92618013	1.310377472	2.831374907	0.000852104
HOXB-AS1	1.109197609	1.051212905	1.170380738	0.00015487
AC048382.2	1.527609833	1.224791053	1.905297885	0.000170736
AL021707.6	1.13116146	1.072778981	1.192721213	5.16E-06
AL096701.3	2.976046932	2.005104166	4.417154726	6.20E-08
AC131953.1	2.001539959	1.32427534	3.025173153	0.000992324
AL158196.1	4.301302898	2.388316641	7.746546793	1.17E-06
AC005261.3	1.226504909	1.157831747	1.299251204	3.79E-12
AC099343.2	1.630343798	1.369728901	1.94054524	3.79E-08
KLHDC7B-DT	1.082032754	1.039287883	1.126535679	0.000126148
AC005253.1	1.464495177	1.174464043	1.826148817	0.000703811
LINC02027	0.701884139	0.604232591	0.815317399	3.63E-06
AC015912.3	1.389577615	1.249311585	1.545591966	1.36E-09
AC000123.1	1.31441063	1.150103218	1.502191523	6.00E-05
AC010201.2	1.280717079	1.112262469	1.474684513	0.000584583
LINC02604	1.151767819	1.095000081	1.211478549	4.27E-08
MATN1-AS1	1.613623477	1.247144789	2.087793451	0.000272428
AC104564.3	1.359201927	1.169548436	1.579609551	6.27E-05
AC135050.3	1.143089645	1.078693362	1.211330284	6.17E-06
ACBD3-AS1	2.002601249	1.410194708	2.843870949	0.000104089
AL928654.2	1.135986589	1.06175773	1.215404883	0.000217263
AC026333.4	3.088547574	2.073571753	4.600335679	2.90E-08
LINC01474	1.410425101	1.181126086	1.684239294	0.000145331
AP000525.1	1.466127988	1.279346981	1.68017849	3.73E-08
AC002553.1	1.315698252	1.159708976	1.492669217	2.03E-05
LINC01355	1.422053961	1.273341294	1.588134679	4.17E-10
AL355488.1	1.265730252	1.169864145	1.369452237	4.52E-09
AC003070.1	1.238874308	1.100156226	1.395083275	0.00040718
CR936218.1	1.295063059	1.113579338	1.506123784	0.000789301
AP002907.1	1.82042794	1.405802803	2.357341925	5.55E-06
AC005785.1	1.621108505	1.38417427	1.898599651	2.06E-09
AC008906.1	1.374944566	1.154398946	1.637624988	0.000357583
AC145098.1	1.365487342	1.15138319	1.619404987	0.00034366
AC025171.4	1.18326355	1.100893044	1.271797144	4.86E-06
MYG1-AS1	1.211720307	1.135855245	1.292652483	5.83E-09
LINC02449	1.737618198	1.386113178	2.178261524	1.66E-06
PCED1B-AS1	1.112958896	1.057784541	1.171011163	3.70E-05
AL512652.1	3.825238681	2.44359683	5.988079041	4.42E-09

Z84485.1	1.445311649	1.255917471	1.663266744	2.75E-07
FSIP2-AS1	2.792968542	1.907576869	4.089310057	1.29E-07
AC026356.2	1.455472056	1.280940027	1.653784613	8.46E-09
AC084824.5	1.361516738	1.234304493	1.501839973	7.00E-10
AL078604.2	1.421144763	1.230310804	1.641579046	1.78E-06
AL158834.2	3.927543032	2.420950394	6.371710179	3.00E-08
LINC01615	1.084437853	1.052826341	1.116998512	7.85E-08
AP000240.1	1.418459985	1.262366601	1.593854532	4.18E-09
AC048341.2	1.095579377	1.060200926	1.132138392	5.02E-08
AC010999.1	4.018098112	2.204536914	7.323584529	5.60E-06
AL590560.3	1.116457146	1.06944839	1.165532221	5.19E-07
AC104758.1	2.044503665	1.532213022	2.728077088	1.18E-06
AC093788.1	1.674856318	1.395256353	2.010486229	3.13E-08
LINC02100	1.314661895	1.198713333	1.441825873	6.34E-09
N4BP2L2-IT2	1.379798501	1.185556631	1.605865004	3.20E-05
AL080317.1	1.400873652	1.16320907	1.687097393	0.000379732
AC130650.2	3.159166574	2.0158721	4.95087632	5.21E-07
AL159169.2	1.90498761	1.451014689	2.500993147	3.48E-06
ASMTL-AS1	1.053376319	1.032095394	1.075096038	5.92E-07
AC009690.2	2.252720494	1.571745011	3.22873595	9.77E-06
ARHGEF2-AS2	3.124559372	2.313705844	4.219581887	1.07E-13
SLBP-DT	1.712272477	1.493457819	1.963146866	1.26E-14
AC092301.1	3.865882231	2.105773122	7.097177405	1.29E-05
AC009120.2	1.269572062	1.154422909	1.396206891	8.65E-07
PKD1P6-NPIPP1	3.224564261	2.017430864	5.153988104	9.93E-07
AC069549.1	1.282978684	1.116433327	1.474368656	0.000443964
AC012645.3	1.540816694	1.194441273	1.987637349	0.000876015
AC005899.7	1.877418831	1.446957334	2.435940152	2.13E-06
AC108463.3	1.56461913	1.208472134	2.025725669	0.000681608
AC019257.1	1.066096436	1.03582114	1.097256626	1.33E-05
AC117383.1	1.343943295	1.136080396	1.589837819	0.000564325
AC107021.2	1.097956903	1.058600702	1.138776271	5.23E-07
AC007497.1	1.98560913	1.425540591	2.765718242	4.97E-05
AC103591.3	1.223685612	1.106162065	1.35369538	8.91E-05
AL354836.1	1.050219079	1.029682184	1.071165581	1.16E-06
AL133406.2	2.73352584	1.625533922	4.596744132	0.000149427
ZMIZ1-AS1	2.160432035	1.674482156	2.787408968	3.12E-09
AC124319.1	1.406302561	1.203005946	1.643954379	1.87E-05
SNHG1	1.046239774	1.026240968	1.066628306	4.42E-06
AC004253.1	1.519083073	1.270001306	1.817016543	4.74E-06
AC093484.4	1.448808392	1.185649894	1.770375697	0.000288921
AP003390.1	1.597707415	1.224864299	2.084042276	0.000548382
AC010326.3	1.149250148	1.097633274	1.203294338	2.97E-09
AL359881.1	1.209165132	1.098455749	1.331032514	0.000105897
NFE4	1.018929036	1.008439978	1.029527193	0.00038246
AC079848.1	0.252936501	0.120932355	0.529030246	0.000261083
AC080013.2	1.75827315	1.449177147	2.133296453	1.06E-08
AC004264.1	1.111666369	1.061908087	1.163756196	5.87E-06
AC034213.1	1.529271819	1.311780484	1.782822907	5.72E-08
LINC00271	0.095870771	0.031052918	0.295985224	4.57E-05
LINC00926	1.63900376	1.374534466	1.954358651	3.73E-08
AC068620.2	2.210021791	1.749486077	2.791789189	2.91E-11
AC055822.1	1.170549534	1.067907963	1.283056461	0.00077059
PRKAR1B-AS1	1.133065065	1.089567343	1.178299303	3.98E-10
AC005387.2	1.880548339	1.450879503	2.437460898	1.82E-06
AC132192.2	1.474463901	1.289929978	1.685396752	1.26E-08
VPS9D1-AS1	1.50427298	1.328560423	1.703224904	1.17E-10
AC027601.1	3.700455634	2.310875525	5.925620723	5.13E-08

AC069281.2	1.967797194	1.596706137	2.425133658	2.17E-10
AP002807.1	1.479215576	1.314284074	1.664844583	8.53E-11
DLEU2	1.626520215	1.25187649	2.113281967	0.000270779
AC138932.4	1.917116073	1.486325983	2.472764442	5.39E-07
AC092535.5	1.030179637	1.013722209	1.046904245	0.000296127
AL133243.3	3.117719952	2.039438773	4.766104197	1.51E-07
LINC02609	1.519002635	1.322477399	1.744732278	3.34E-09
AC027271.1	2.779079011	2.117032978	3.648162419	1.81E-13
AC084018.1	1.128722629	1.067239157	1.193748154	2.26E-05
AC060766.3	1.133945562	1.052241106	1.221994208	0.000985594
AL360181.2	1.159554125	1.083914109	1.240472614	1.70E-05
AC027607.1	2.126093429	1.488606117	3.036581145	3.36E-05
LMNTD2-AS1	1.051227812	1.029700074	1.073205626	2.22E-06
MIR155HG	1.072420036	1.040915005	1.104878619	4.31E-06
LINC01126	1.60401234	1.231083155	2.089912104	0.000465583
AL139123.1	2.846621193	2.085951093	3.884679867	4.26E-11
AC055855.1	1.685502968	1.255590549	2.262616787	0.00051095
TFAP2E-AS1	2.016589895	1.448223877	2.808015299	3.29E-05
AC233728.1	1.819916308	1.409794762	2.349345775	4.30E-06
AL662797.1	3.662027128	2.454428568	5.463773876	2.04E-10
AC010168.2	1.392502264	1.169689488	1.657758385	0.000197805
AC129510.1	1.444260802	1.259344909	1.656328818	1.45E-07
AC105105.3	1.669433418	1.333830262	2.089477211	7.62E-06
ZEB2-AS1	2.334980783	1.501043088	3.632231015	0.000168802
AC017083.1	2.80879795	1.850232659	4.263975065	1.24E-06
AL162377.1	0.219401227	0.111888622	0.430221566	1.01E-05
AC133644.1	1.031658933	1.014802252	1.048795617	0.000208828
U47924.3	1.886858554	1.545136159	2.304156291	4.72E-10
AC009133.1	1.151465146	1.061070679	1.249560474	0.000722082
AC020558.2	1.81942752	1.443313435	2.293553444	4.07E-07
AC008555.1	0.600285729	0.470577896	0.765745606	3.98E-05
AC108673.3	1.131720693	1.082650259	1.183015213	4.47E-08
LAMC1-AS1	1.65622475	1.258460126	2.17971183	0.000317617
SCAT2	1.352270506	1.233009185	1.483067234	1.49E-10
MED8-AS1	2.00339004	1.614705876	2.485636369	2.72E-10
AL161935.1	2.417714174	1.536781175	3.803626646	0.000134249
AL442128.2	4.59782458	2.50797313	8.429113781	8.09E-07
AL683807.1	1.319846051	1.163566637	1.497115458	1.59E-05
AL359881.2	1.825194213	1.333296618	2.498569239	0.000173134
LINC01705	1.06453704	1.02962928	1.100628285	0.000236531
LINC01871	1.1633795	1.093625483	1.237582593	1.61E-06
AL031186.1	1.58787364	1.273270838	1.980209256	4.05E-05
AC112220.2	0.468740313	0.32200974	0.682331786	7.64E-05
LINC00460	1.115490976	1.081626355	1.150415864	3.69E-12
AC021851.1	3.382482732	1.82819195	6.258199218	0.000103667
NUP153-AS1	1.612031348	1.350798528	1.923784351	1.20E-07
AC040162.3	2.447097099	1.732758044	3.455926366	3.75E-07
AL645939.4	1.447559118	1.232625145	1.699971324	6.47E-06
ZNF32-AS2	1.463770893	1.199560218	1.786175631	0.000175778
LINC00893	1.418824986	1.215022365	1.656812582	9.79E-06
KIF1C-AS1	2.09613758	1.652629964	2.658667003	1.05E-09
AL021707.1	2.395229676	1.6070103	3.570061252	1.79E-05
AC011374.2	1.299498556	1.156031026	1.460770912	1.14E-05
AC007220.1	2.237723216	1.579769248	3.169706714	5.79E-06
AGAP2-AS1	1.058117011	1.036451361	1.080235554	8.71E-08
IGBP1-AS1	2.77712943	1.885008495	4.091465842	2.39E-07
AC127024.5	1.325876198	1.165305756	1.508572049	1.85E-05
AL162586.1	1.27238348	1.156396472	1.40000403	7.83E-07

AP000892.3	1.992464947	1.32388722	2.998681841	0.000949263
AC015660.3	1.872162135	1.402072074	2.499865109	2.13E-05
AC073655.2	1.61409679	1.323755748	1.968118705	2.22E-06
AC010245.2	1.876481177	1.46183806	2.408735758	7.80E-07
RASGRP3-AS1	1.703613668	1.443084571	2.011177714	3.15E-10
AC008760.1	1.396266493	1.180274109	1.651785889	9.90E-05
ZNNT1	1.189262894	1.112730633	1.27105895	3.27E-07
LINC02446	1.125191244	1.072282931	1.180710146	1.59E-06
IBA57-DT	1.666055966	1.238722837	2.240809971	0.000736305
AC092119.2	1.781690478	1.474618447	2.152706665	2.17E-09
AC004585.1	1.228589894	1.096009604	1.377207939	0.000410127
LINC01801	0.474685423	0.325821059	0.691564419	0.000104097
RAP2C-AS1	0.275917637	0.129441123	0.588148038	0.000854735
APCDD1L-DT	1.112057867	1.076788976	1.148481946	1.05E-10
AC007743.1	0.588647964	0.449673277	0.770573754	0.000114926
AC018638.6	1.289788308	1.121446927	1.483399562	0.000362145
UST-AS2	1.651811712	1.42748944	1.91138502	1.59E-11
AL021154.1	1.524509172	1.196177151	1.942963227	0.00065559
AC015813.1	1.199078069	1.110027645	1.29527244	4.00E-06
AC068792.1	2.45614534	1.782719857	3.383958453	3.88E-08
AL592211.1	2.762278704	1.808233643	4.219689014	2.60E-06
AC010973.2	1.44946102	1.27427267	1.648734448	1.63E-08
MELTF-AS1	1.164763394	1.121080832	1.210148033	5.27E-15
GARS1-DT	1.286304124	1.131663959	1.462075634	0.000116834
AC010761.1	1.323554328	1.175900293	1.489748807	3.40E-06
AL157392.4	1.520066936	1.230641415	1.877560322	0.000102015
LINC01311	2.058778751	1.580792843	2.681293734	8.45E-08
PDXDC2P-NPIPB14P	1.649910151	1.372235465	1.983772885	1.01E-07
AC106782.5	1.412240133	1.166779732	1.709339081	0.000395043
INE1	1.37725074	1.210822883	1.566554141	1.11E-06
AL137244.1	3.048868493	1.642510364	5.659385347	0.0004119
AC004148.1	1.248605212	1.146266183	1.360081104	3.61E-07
AL049780.1	1.323268901	1.125895482	1.555242571	0.000676822
AP000866.6	1.523958258	1.241456535	1.870745134	5.64E-05
ITPR1-DT	1.369487796	1.159177187	1.617955257	0.000218691
AC087289.2	4.210794598	2.50796725	7.069785757	5.39E-08
AC008764.8	2.063167081	1.575847532	2.701186706	1.38E-07
AC009053.3	0.900926184	0.848287052	0.956831756	0.000682405
MYOSLID	1.113071691	1.071072151	1.156718142	4.80E-08
LINC00342	1.160718206	1.103387713	1.221027513	8.08E-09
AL359921.1	1.798352452	1.304260045	2.479621724	0.000342677
AC156455.1	1.113836185	1.068350103	1.161258882	4.02E-07
MIR223HG	1.181343644	1.092317067	1.27762611	3.06E-05
ZNF436-AS1	1.361876376	1.196353985	1.550299731	2.99E-06
AC006435.2	1.358855865	1.187738396	1.554626227	7.99E-06
UBE2Q1-AS1	2.322127593	1.625471112	3.317362282	3.67E-06
AC005393.1	1.820714923	1.454395298	2.279299744	1.71E-07
AC127024.6	2.741532273	1.889292141	3.978209107	1.10E-07
IL10RB-DT	2.566778105	1.687158878	3.904996693	1.07E-05
AC008105.2	1.348604664	1.213551546	1.498687506	2.77E-08
AL158151.4	1.823916485	1.515245764	2.19546652	2.11E-10
YEATS2-AS1	2.266322081	1.69164164	3.036231583	4.18E-08
AC084876.1	1.645000544	1.42461521	1.89947908	1.18E-11
LENG8-AS1	1.124179618	1.067552359	1.183810614	9.05E-06
LINC01234	1.133429505	1.053612006	1.219293663	0.000774731
AC002553.2	1.431706117	1.226670358	1.671013235	5.35E-06
U91328.3	1.670366472	1.354526662	2.059851778	1.61E-06
AC092171.4	1.365993982	1.187730661	1.571012369	1.23E-05

AC022167.2	1.937150779	1.401390897	2.677734777	6.26E-05
LINC02019	2.569381282	1.739010091	3.796251792	2.16E-06
AC005306.1	2.109002612	1.538322349	2.891391405	3.56E-06
ANKRD10-IT1	1.04761294	1.020216423	1.075745153	0.000581051
AC010719.1	1.315354671	1.166850904	1.482758342	7.31E-06
AC253576.2	1.505081199	1.186330923	1.909475148	0.00075943
AP001767.2	1.67980807	1.312302753	2.150231833	3.83E-05
STAG3L5P-PVRIG2P-PILRB	1.212423335	1.0820374	1.358520827	0.000905967
LINC01160	3.760758858	2.208698679	6.403457078	1.07E-06
AP000553.2	1.507746331	1.278513399	1.778079919	1.06E-06
AC011468.1	1.218255499	1.129940097	1.313473576	2.72E-07
LINC01270	3.68327184	2.382733147	5.69366799	4.44E-09
AC099850.3	1.115228896	1.073900456	1.158147837	1.51E-08
AL157394.1	1.59687627	1.283682555	1.986483195	2.65E-05
AC022973.5	3.944112495	2.147701564	7.243102876	9.65E-06
AF117829.1	1.586502177	1.31602514	1.912569205	1.30E-06
AC245052.4	2.595978961	1.499591324	4.493962226	0.000656498
AC087741.1	1.317648459	1.186572628	1.463203702	2.47E-07
PTOV1-AS2	1.131620147	1.076762305	1.189272833	1.08E-06
AL359504.1	1.615516778	1.304359838	2.00090066	1.11E-05
AL390728.5	1.063817669	1.036597622	1.091752489	2.90E-06
PVT1	1.156411522	1.093091082	1.223399981	4.24E-07
AP006621.2	1.127179886	1.0562307	1.202894875	0.000307096
AC245128.3	1.54570267	1.23394238	1.936230396	0.000151267
AC124067.2	1.344769392	1.20749029	1.497655703	6.97E-08
COL18A1-AS1	0.041235466	0.006777608	0.250879609	0.000538371
LINC01004	1.249642066	1.15728393	1.349370931	1.28E-08
NALT1	1.498837416	1.31184335	1.712486175	2.64E-09
AL359880.1	1.735295893	1.294175442	2.326772506	0.000230352
NRIR	1.806580816	1.307870141	2.495457417	0.000332671
LINC01389	1.871177075	1.482340601	2.362010219	1.35E-07
AC004687.1	1.190043824	1.073573646	1.319149653	0.000929933
AC131009.3	1.243009782	1.130404314	1.366832468	7.13E-06
LINC00426	1.655899265	1.237642776	2.215503884	0.000685503
AC126118.1	1.654865925	1.282395138	2.135520596	0.000108033
AL135999.1	1.429370847	1.226560224	1.665716022	4.75E-06
AC005899.6	1.162138618	1.064290063	1.26898316	0.000812699
AL023653.1	1.847388829	1.466510355	2.327188127	1.89E-07
AL022067.1	1.659116102	1.300395941	2.116790858	4.64E-05
SCGB1B2P	1.232731551	1.137121217	1.336380902	3.78E-07
FAM13A-AS1	1.303451967	1.142777393	1.486717397	7.87E-05
SNHG4	1.895464681	1.522324703	2.360065727	1.08E-08
AC244197.2	1.351164847	1.200954281	1.520163152	5.58E-07
ITGB1-DT	1.11280941	1.045195756	1.184796988	0.000831407
AC132872.3	1.043909435	1.018232552	1.070233814	0.000719778
FOXO6-AS1	1.821299205	1.329663151	2.494715139	0.000187776
AL354760.1	2.748155759	2.057459979	3.670720282	7.64E-12
AC004034.1	2.860799545	2.010536273	4.070642319	5.19E-09
SNHG3	1.131645462	1.093063058	1.171589729	2.79E-12
AC093535.1	1.507100468	1.183489745	1.919198564	0.000881126
AC026471.4	1.059697799	1.031491204	1.088675716	2.53E-05
AC008735.2	1.085986203	1.044392347	1.129236572	3.48E-05
AC090589.3	1.338930418	1.165939655	1.537587865	3.55E-05
AC004908.1	1.234022107	1.154778689	1.318703381	5.31E-10
AC073575.2	2.516878519	1.669219085	3.794994638	1.06E-05
KDM2B-DT	2.448371441	1.501766932	3.991646495	0.000329967
AC008870.2	2.234304358	1.747557696	2.856624406	1.43E-10
AL133410.1	1.948544665	1.540243971	2.465081105	2.69E-08

AC007014.2	1.327101439	1.131504118	1.556510668	0.000503996
AP001160.1	1.581547087	1.353074086	1.84859884	8.49E-09
AC015819.1	1.463024324	1.204756198	1.776658361	0.000123189
ADAMTSL4-AS2	1.255588166	1.124822762	1.401555601	4.99E-05
AL122125.1	1.515628518	1.185471216	1.937735622	0.000909115
PSPC1-AS2	1.699792275	1.391263644	2.076740662	2.09E-07
C10orf55	3.382895098	2.053081278	5.574050753	1.73E-06
AC092809.4	2.190976258	1.612611733	2.976771695	5.28E-07
U62317.1	1.063455307	1.043005838	1.084305714	5.29E-10
AC020907.4	1.362676563	1.226950719	1.513416461	7.44E-09
LINC02328	2.080732943	1.598885622	2.707791928	4.98E-08
AC005104.1	1.348312808	1.186093096	1.532719002	4.89E-06
WDFY3-AS2	0.737416991	0.640187098	0.849413898	2.42E-05
NARF-IT1	2.179104875	1.589297161	2.987797482	1.32E-06
AL022322.1	1.180470747	1.080445392	1.289756238	0.000239965
AC010201.1	1.61412887	1.338239803	1.946894721	5.54E-07
AC087289.5	2.339629932	1.573219448	3.479405384	2.70E-05
LINC00174	1.219058826	1.120360288	1.326452246	4.26E-06
AC040160.1	2.059697357	1.405442887	3.018516969	0.000211141
LINC02804	1.596759145	1.210319689	2.10658373	0.000932439
AC084036.1	1.081846028	1.044969718	1.120023681	8.75E-06
MRPL20-DT	1.608183749	1.367880385	1.890702578	8.73E-09
AP000919.3	2.960241875	1.561254857	5.612813258	0.000885078
AC138207.4	1.303815499	1.125348085	1.510585816	0.000411909
AL513218.1	1.690523968	1.425535392	2.004770491	1.58E-09
AC018648.1	2.170785056	1.738143295	2.711115806	8.22E-12
AC020658.5	2.108812823	1.591519534	2.7942425	2.03E-07
AC090772.3	1.822531341	1.288291498	2.578314375	0.000695999
AC090948.3	1.465270713	1.20017318	1.788923713	0.000175491
LINC02747	0.963484939	0.948958254	0.978233999	1.59E-06
AC087623.1	1.28015546	1.124016633	1.457983764	0.000197993
AL596223.2	1.612190682	1.235868913	2.103102333	0.000429216
AC091057.1	3.986387496	2.369529886	6.706513964	1.88E-07
MUC12-AS1	1.155447511	1.100847294	1.21275581	4.91E-09
AC103706.1	1.519713915	1.370620359	1.685025593	1.96E-15
AL031670.1	1.67721444	1.334672617	2.107669135	9.13E-06
AC114730.3	1.746292154	1.332224698	2.289055511	5.41E-05
AL162171.1	0.523942169	0.361128968	0.760158895	0.000663518
AC243960.1	1.260944233	1.132197731	1.404330987	2.45E-05
AL022238.2	1.847298705	1.370261461	2.490409752	5.66E-05
AC022126.1	3.186250948	1.974528779	5.141578695	2.07E-06
LINC01943	1.623404742	1.382139432	1.906785159	3.58E-09
AL117379.1	1.286201931	1.181072216	1.400689464	7.24E-09
AC011005.4	1.501081668	1.254121458	1.796673009	9.47E-06
AC005840.2	1.362675527	1.191761987	1.558100201	6.02E-06
AL158212.2	1.838213184	1.466001602	2.304927707	1.34E-07
AC067945.2	2.579658552	1.673459967	3.976574508	1.77E-05
AL731567.1	1.339014845	1.205091014	1.487821861	5.64E-08
AC068790.5	1.404873947	1.152770709	1.712110475	0.000754874
AL139349.1	1.095944845	1.054823197	1.138669595	2.66E-06
CYTOR	1.06109772	1.034379045	1.088506554	5.17E-06
AC068051.1	0.336091799	0.186623764	0.605269635	0.000280457
LINC01843	0.801926876	0.730966028	0.879776474	3.02E-06
ZKSCAN2-DT	1.601221516	1.353625316	1.894106377	3.96E-08
AC139100.2	1.142806936	1.068280885	1.222532117	0.000104609
FOXD2-AS1	1.54700378	1.37471392	1.740886349	4.40E-13
KDM4A-AS1	1.989671057	1.523927001	2.597756266	4.28E-07
AC021078.1	1.12715666	1.052902345	1.206647646	0.000576188

AC127070.1	3.067480164	1.766974305	5.325167735	6.81E-05
AL645940.1	2.117523008	1.615491859	2.775565636	5.51E-08
AC005387.1	1.899498277	1.490219124	2.421183333	2.20E-07
ITGB2-AS1	1.122181442	1.063019945	1.184635524	3.03E-05
AC105020.1	1.158693995	1.065704999	1.259796825	0.000558765
AC073487.1	1.699290915	1.335676066	2.161893657	1.59E-05
AC127502.2	1.206004225	1.130112754	1.286992105	1.62E-08
AC124854.1	0.906086089	0.872121518	0.941373401	4.21E-07
LINC01138	1.563991023	1.353323253	1.807452812	1.37E-09
AC004908.3	1.569941497	1.352988125	1.821683618	2.78E-09
AC011498.6	1.513564181	1.224097556	1.871481988	0.000129692
RNF139-AS1	5.627805285	3.283817362	9.64493114	3.26E-10
AC016957.2	1.754293011	1.388699494	2.216133861	2.43E-06
LINC00528	2.1988449	1.524055906	3.172402583	2.52E-05
LASTR	1.175673733	1.112363214	1.242587592	1.00E-08
DPP9-AS1	1.739821028	1.289139601	2.348060059	0.000294257
AC002070.1	0.667569538	0.560340949	0.795317724	6.08E-06
AC092118.2	1.562854215	1.2758992	1.914346601	1.60E-05
AL606534.1	4.426643394	2.247007323	8.72056425	1.71E-05
AC007038.1	1.176293019	1.090557495	1.268768747	2.61E-05
LINC02154	1.062266137	1.035921684	1.089280554	2.43E-06
MMP25-AS1	1.177793921	1.081250121	1.282958025	0.000176711
AC027796.4	1.297206226	1.183694032	1.421603849	2.56E-08
LINC02195	1.628666741	1.354363214	1.958525843	2.18E-07
AL161452.1	2.625598482	1.625665232	4.24058241	7.93E-05
NCBP2-AS1	2.949397405	1.736871279	5.00839939	6.24E-05

Multivariate Cox analysis

**LncRNA**	coef	HR	HR.95L	HR.95H
AC026401.3	0.062124799	1.064095135	0.990621474	1.143018283
LINC01615	0.057177391	1.058843623	1.015436862	1.104105888
`PRKAR1B-AS1`	0.049535837	1.050783249	0.994718885	1.110007513
LINC02609	0.221623791	1.248101742	1.05376734	1.478275042
LINC00460	0.039951118	1.040759899	0.9953125	1.088282491
AC084876.1	0.251078409	1.285410868	1.042947972	1.584241156
AC008870.2	0.298935357	1.348422455	0.95027485	1.91338655
LINC02747	-0.018735587	0.981438833	0.966483507	0.996625578
AC103706.1	0.172116641	1.187816374	0.980619097	1.438792843

Table S6 FRLRS set enrichment analyses and immunity gene expression

NAME	SIZE	ES	NES	NOM p-val	FDR q-val	FWER p-val	RANK AT MAX	LEADING EDGE
KEGG_VASOPRESSIN_REGULATED_WATER_REABSORPTION	44	-0.68	-2.33	0	0.014312	0.011	6520	tags=61%, list=12%, signal=70%
KEGG_PROSTATE_CANCER	89	-0.62	-2.25	0	0.021235	0.024	5067	tags=51%, list=9%, signal=56%
KEGG_PROPANOATE_METABOLISM	33	-0.8	-2.23	0	0.01703	0.03	5342	tags=64%, list=10%, signal=70%
KEGG_PEROXISOME	78	-0.7	-2.21	0.0040241	0.01439	0.033	6032	tags=59%, list=11%, signal=66%
KEGG_ENDOMETRIAL_CANCER	52	-0.67	-2.21	0	0.011896	0.033	5658	tags=60%, list=10%, signal=66%
KEGG_VALINE_LEUCINE_AND_ISOLEUCINE_DEGRADATION	44	-0.81	-2.19	0.001992	0.012489	0.038	6507	tags=80%, list=12%, signal=90%
KEGG_PROXIMAL_TUBULE_BICARBONATE_RECLAMATION	23	-0.76	-2.19	0	0.010705	0.038	8201	tags=74%, list=15%, signal=87%
KEGG_INSULIN_SIGNALING_PATHWAY	137	-0.52	-2.19	0	0.009498	0.039	4332	tags=38%, list=8%, signal=41%
KEGG_ADHERENS_JUNCTION	73	-0.64	-2.19	0.002004	0.008879	0.04	5067	tags=55%, list=9%, signal=60%
KEGG_ERBB_SIGNALING_PATHWAY	87	-0.57	-2.18	0	0.008383	0.041	5658	tags=49%, list=10%, signal=55%
KEGG_TIGHT_JUNCTION	132	-0.55	-2.17	0	0.008241	0.044	5885	tags=45%, list=11%, signal=50%
KEGG_ADIPOCYTOKINE_SIGNALING_PATHWAY	67	-0.56	-2.15	0	0.009375	0.055	8033	tags=55%, list=15%, signal=65%
KEGG_ENDOCYTOSIS	181	-0.53	-2.14	0.0020325	0.00945	0.058	6437	tags=49%, list=12%, signal=55%
KEGG_RENAL_CELL_CARCINOMA	70	-0.61	-2.11	0.0020121	0.010519	0.064	4785	tags=49%, list=9%, signal=53%
KEGG_SPHINGOLIPID_METABOLISM	39	-0.61	-2.11	0.0040241	0.010811	0.071	6930	tags=56%, list=13%, signal=64%
KEGG_FATTY_ACID_METABOLISM	42	-0.75	-2.08	0.0040816	0.012479	0.082	8308	tags=81%, list=15%, signal=95%
KEGG_TRYPTOPHAN_METABOLISM	40	-0.63	-2.08	0.0020367	0.013074	0.086	7871	tags=60%, list=14%, signal=70%
KEGG_MELANOMA	71	-0.51	-2.06	0	0.014382	0.097	6040	tags=39%, list=11%, signal=44%
KEGG_NEUROTROPHIN_SIGNALING_PATHWAY	126	-0.55	-2.06	0	0.013955	0.099	5658	tags=48%, list=10%, signal=53%
KEGG_CITRATE_CYCLE_TCA_CYCLE	31	-0.83	-2.04	0.0019802	0.015152	0.108	5155	tags=77%, list=9%, signal=85%
KEGG_RENIN_ANGIOTENSIN_SYSTEM	17	-0.67	-2.02	0	0.017782	0.125	7982	tags=71%, list=14%, signal=82%
KEGG_BUTANOATE_METABOLISM	34	-0.69	-2.02	0.0060852	0.017541	0.126	8273	tags=74%, list=15%, signal=86%
KEGG_THYROID_CANCER	29	-0.61	-2	0	0.02016	0.139	5783	tags=55%, list=10%, signal=62%
KEGG_PYRUVATE_METABOLISM	40	-0.65	-2	0.0102459	0.019462	0.141	8681	tags=63%, list=16%, signal=74%
KEGG_WNT_SIGNALING_PATHWAY	151	-0.5	-2	0.0060484	0.019221	0.145	5120	tags=36%, list=9%, signal=39%
KEGG_TGF_BETA_SIGNALING_PATHWAY	86	-0.57	-1.99	0.0080808	0.019344	0.149	4741	tags=43%, list=9%, signal=47%
KEGG_LYSINE_DEGRADATION	44	-0.61	-1.99	0.0098619	0.019246	0.154	3146	tags=43%, list=6%, signal=46%
KEGG_COLORECTAL_CANCER	62	-0.6	-1.99	0.0039761	0.019525	0.162	6310	tags=56%, list=11%, signal=64%
KEGG_GLYCOLYSIS_GLUCONEOGENESIS	62	-0.61	-1.98	0.012	0.019671	0.167	8308	tags=60%, list=15%, signal=70%
KEGG_LONG_TERM_POTENTIATION	70	-0.51	-1.97	0	0.02048	0.174	6134	tags=43%, list=11%, signal=48%
KEGG_INOSITOL_PHOSPHATE_METABOLISM	54	-0.59	-1.95	0.0039761	0.023925	0.196	3439	tags=39%, list=6%, signal=41%
KEGG_NON_SMALL_CELL_LUNG_CANCER	54	-0.56	-1.94	0.0020367	0.024883	0.206	5783	tags=50%, list=10%, signal=56%
KEGG_GLIOMA	65	-0.52	-1.94	0	0.024187	0.207	5658	tags=45%, list=10%, signal=50%
KEGG_TERPENOID_BACKBONE_BIOSYNTHESIS	15	-0.7	-1.93	0.0076923	0.024739	0.211	5725	tags=60%, list=10%, signal=67%
KEGG_PANCREATIC_CANCER	70	-0.57	-1.93	0.01	0.025569	0.217	6249	tags=50%, list=11%, signal=56%
KEGG_ALDOSTERONE_REGULATED_SODIUM_REABSORPTION	42	-0.55	-1.93	0.0102459	0.025128	0.219	8033	tags=55%, list=15%, signal=64%
KEGG_PPAR_SIGNALING_PATHWAY	69	-0.58	-1.91	0.0100402	0.0267	0.229	5806	tags=45%, list=10%, signal=50%
KEGG_TYPE_II_DIABETES_MELLITUS	47	-0.51	-1.9	0.0100604	0.028042	0.246	5450	tags=38%, list=10%, signal=42%
KEGG_EPITHELIAL_CELL_SIGNALING_IN_HELICOBACTER_PYLORI_INFECTION	68	-0.53	-1.9	0.017341	0.028941	0.259	6968	tags=50%, list=13%, signal=57%
KEGG_CHRONIC_MYELOID_LEUKEMIA	73	-0.57	-1.89	0.0080483	0.030243	0.273	3437	tags=44%, list=6%, signal=47%
KEGG_GLYCOSYLPHOSPHATIDYLINOSITOL_GPI_ANCHOR_BIOSYNTHESIS	25	-0.62	-1.88	0.0060484	0.03111	0.278	6545	tags=56%, list=12%, signal=63%
KEGG_UBIQUITIN_MEDIATED_PROTEOLYSIS	135	-0.51	-1.87	0.0140562	0.033344	0.292	5695	tags=42%, list=10%, signal=47%
KEGG_PATHWAYS_IN_CANCER	325	-0.44	-1.86	0.0118343	0.033683	0.295	6310	tags=38%, list=11%, signal=43%
KEGG_AXON_GUIDANCE	129	-0.47	-1.86	0.0178926	0.033311	0.301	5844	tags=41%, list=11%, signal=46%
KEGG_DORSO_VENTRAL_AXIS_FORMATION	24	-0.59	-1.85	0.0060729	0.034527	0.309	5211	tags=54%, list=9%, signal=60%

KEGG_REGULATION_OF_ACTIN_CYTOSKELETON	213	-0.45	-1.83	0.0252918	0.039178	0.339	5067 tags=34%, list=9%, signal=38%
KEGG_MELANOGENESIS	101	-0.44	-1.82	0.0099404	0.041623	0.356	5120 tags=35%, list=9%, signal=38%
KEGG_GLYCINE_SERINE_AND_THREONINE_METABOLISM	31	-0.61	-1.81	0.0243408	0.043032	0.368	8330 tags=55%, list=15%, signal=65%
KEGG_FOCAL_ADHESION	199	-0.48	-1.81	0.0355731	0.042544	0.369	4357 tags=33%, list=8%, signal=36%
KEGG_BIOSYNTHESIS_OF_UNSATURATED_FATTY_ACIDS	22	-0.59	-1.8	0.0186722	0.042475	0.371	7836 tags=59%, list=14%, signal=69%
KEGG_MTOR_SIGNALING_PATHWAY	52	-0.49	-1.8	0.0162272	0.043561	0.383	4785 tags=37%, list=9%, signal=40%
KEGG_GAP_JUNCTION	90	-0.48	-1.79	0.0140562	0.043163	0.385	7642 tags=43%, list=14%, signal=50%
KEGG_BETA_ALANINE_METABOLISM	22	-0.65	-1.78	0.036017	0.04677	0.41	8170 tags=73%, list=15%, signal=85%
KEGG_VASCULAR_SMOOTH_MUSCLE_CONTRACTION	115	-0.44	-1.77	0.0216963	0.047012	0.416	7038 tags=41%, list=13%, signal=47%
KEGG_OOCYTE_MEIOSIS	113	-0.45	-1.77	0.0238095	0.046262	0.417	4808 tags=41%, list=9%, signal=44%
KEGG_LONG_TERM_DEPRESSION	70	-0.42	-1.76	0.0105042	0.048915	0.429	4990 tags=31%, list=9%, signal=35%
KEGG_PHOSPHATIDYLINOSITOL_SIGNALING_SYSTEM	76	-0.49	-1.76	0.0178926	0.048974	0.436	6610 tags=43%, list=12%, signal=49%
KEGG_GNRH_SIGNALING_PATHWAY	101	-0.4	-1.76	0.0097087	0.048926	0.439	6520 tags=39%, list=12%, signal=44%
KEGG_HISTIDINE_METABOLISM	29	-0.55	-1.75	0.0470348	0.048281	0.439	5823 tags=41%, list=11%, signal=46%
KEGG_REGULATION_OF_AUTOPHAGY	35	-0.48	-1.75	0.0097656	0.049714	0.447	5154 tags=34%, list=9%, signal=38%
KEGG_MAPK_SIGNALING_PATHWAY	267	-0.39	-1.73	0.0183299	0.053882	0.467	6481 tags=36%, list=12%, signal=40%
KEGG_ARGININE_AND_PROLINE_METABOLISM	54	-0.5	-1.73	0.0323194	0.054683	0.474	6605 tags=43%, list=12%, signal=48%
KEGG_ONE_CARBON_POOL_BY_FOLATE	17	-0.61	-1.72	0.0281125	0.055022	0.478	6071 tags=53%, list=11%, signal=59%
KEGG_O_GLYCAN_BIOSYNTHESIS	30	-0.49	-1.71	0.0229167	0.056674	0.486	10141 tags=53%, list=18%, signal=65%
KEGG_ACUTE_MYELOID_LEUKEMIA	57	-0.5	-1.7	0.0426829	0.058942	0.504	5658 tags=47%, list=10%, signal=53%
KEGG_LEUKOCYTE_TRANSENDOTHELIAL_MIGRATION	116	-0.45	-1.69	0.040856	0.064502	0.531	5247 tags=39%, list=9%, signal=43%
KEGG_SELENOAMINO_ACID_METABOLISM	26	-0.51	-1.68	0.0390625	0.064397	0.535	6120 tags=42%, list=11%, signal=48%
KEGG_BLADDER_CANCER	42	-0.47	-1.66	0.0217391	0.072262	0.564	5067 tags=40%, list=9%, signal=45%
KEGG_PROGESTERONE_MEDIATED_OOCYTE_MATURATION	85	-0.44	-1.64	0.0412574	0.078612	0.593	5915 tags=45%, list=11%, signal=50%
KEGG_SMALL_CELL_LUNG_CANCER	84	-0.47	-1.63	0.0569745	0.081388	0.609	7170 tags=40%, list=13%, signal=46%
KEGG_N_GLYCAN_BIOSYNTHESIS	46	-0.5	-1.62	0.0818363	0.08618	0.628	5277 tags=37%, list=10%, signal=41%
KEGG_GLYCEROLIPID_METABOLISM	49	-0.41	-1.62	0.0264766	0.085257	0.628	6940 tags=37%, list=13%, signal=42%
KEGG_CALCIUM_SIGNALING_PATHWAY	178	-0.34	-1.58	0.0257426	0.098504	0.668	7990 tags=37%, list=14%, signal=43%
KEGG_FC_EPSILON_RI_SIGNALING_PATHWAY	79	-0.39	-1.58	0.0560928	0.097698	0.67	5658 tags=35%, list=10%, signal=39%
KEGG_STEROID_BIOSYNTHESIS	17	-0.58	-1.57	0.0789474	0.10067	0.681	8130 tags=59%, list=15%, signal=69%
KEGG_VEGF_SIGNALING_PATHWAY	76	-0.37	-1.57	0.0336634	0.100951	0.689	5658 tags=36%, list=10%, signal=40%
KEGG_CYSTEINE_AND_METHIONINE_METABOLISM	34	-0.47	-1.56	0.039666	0.105857	0.71	6120 tags=41%, list=11%, signal=46%
KEGG_ETHER_LIPID_METABOLISM	33	-0.4	-1.55	0.0458167	0.109629	0.724	4001 tags=30%, list=7%, signal=33%
KEGG_APOPTOSIS	87	-0.42	-1.52	0.1024096	0.123792	0.761	7702 tags=41%, list=14%, signal=48%
KEGG_AMINOACYL_TRNA_BIOSYNTHESIS	41	-0.5	-1.51	0.118	0.128455	0.767	6180 tags=51%, list=11%, signal=58%
KEGG_ARRHYTHMOGENIC_RIGHT_VENTRICULAR_CARDIOMYOPATHY_ARVC	74	-0.41	-1.51	0.0856574	0.127001	0.767	5000 tags=28%, list=9%, signal=31%
KEGG_ALANINE_ASPARTATE_AND_GLUTAMATE_METABOLISM	32	-0.51	-1.5	0.0890269	0.129037	0.77	6605 tags=44%, list=12%, signal=50%
KEGG_PANTOTHENATE_AND_COA_BIOSYNTHESIS	16	-0.5	-1.5	0.0815109	0.129066	0.772	5887 tags=31%, list=11%, signal=35%
KEGG_PROTEIN_EXPORT	24	-0.53	-1.49	0.1199187	0.131054	0.779	7217 tags=67%, list=13%, signal=77%
KEGG_NITROGEN_METABOLISM	23	-0.48	-1.47	0.0582329	0.144087	0.806	6605 tags=30%, list=12%, signal=35%
KEGG_B_CELL_RECEPTOR_SIGNALING_PATHWAY	75	-0.42	-1.46	0.1395349	0.1479	0.82	5658 tags=39%, list=10%, signal=43%
KEGG_GLYCOSPHINGOLIPID_BIOSYNTHESIS_LACTO_AND_NEOLACTO_SERIES	26	-0.41	-1.45	0.0682261	0.14848	0.824	6301 tags=42%, list=11%, signal=48%
KEGG_ASCORBATE_AND_ALDARATE_METABOLISM	25	-0.57	-1.45	0.1012146	0.152506	0.838	8325 tags=56%, list=15%, signal=66%
KEGG_VIBRIO_CHOLERAE_INFECTION	54	-0.44	-1.44	0.1342685	0.151019	0.839	6968 tags=46%, list=13%, signal=53%
KEGG_HUNTINGTONS_DISEASE	182	-0.39	-1.42	0.1745731	0.163625	0.858	5848 tags=31%, list=11%, signal=35%
KEGG_GLYOXYLATE_AND_DICARBOXYLATE_METABOLISM	16	-0.54	-1.41	0.1568228	0.166421	0.864	4893 tags=44%, list=9%, signal=48%
KEGG_LYSOSOME	121	-0.4	-1.38	0.197318	0.186466	0.891	7780 tags=52%, list=14%, signal=60%

KEGG_ALZHEIMERS_DISEASE	166	-0.37	-1.38	0.1928166	0.189002	0.899	7867	tags=39%, list=14%, signal=46%
KEGG_DRUG_METABOLISM_CYTOCHROME_P450	71	-0.48	-1.34	0.1208333	0.209734	0.914	9275	tags=51%, list=17%, signal=61%
KEGG_PURINE_METABOLISM	159	-0.31	-1.34	0.122288	0.209311	0.917	7803	tags=41%, list=14%, signal=47%
KEGG_RETINOL_METABOLISM	64	-0.49	-1.33	0.1239669	0.218018	0.924	9275	tags=55%, list=17%, signal=66%
KEGG_RNA_DEGRADATION	59	-0.38	-1.33	0.1854839	0.216344	0.924	7439	tags=44%, list=13%, signal=51%
KEGG_SNARE_INTERACTIONS_IN_VESICULAR_TRANSPORT	38	-0.38	-1.32	0.170297	0.21521	0.925	3314	tags=29%, list=6%, signal=31%
KEGG_JAK_STAT_SIGNALING_PATHWAY	155	-0.31	-1.31	0.1719368	0.22471	0.931	3755	tags=23%, list=7%, signal=24%
KEGG_STARCH_AND_SUCROSE_METABOLISM	52	-0.41	-1.31	0.154334	0.224528	0.932	8492	tags=42%, list=15%, signal=50%
KEGG_NICOTINATE_AND_NICOTINAMIDE_METABOLISM	24	-0.39	-1.3	0.1739131	0.228906	0.937	7466	tags=50%, list=13%, signal=58%
KEGG_RIG_I_LIKE_RECEPTOR_SIGNALING_PATHWAY	71	-0.34	-1.29	0.1839081	0.233292	0.941	6906	tags=34%, list=12%, signal=39%
KEGG_FC_GAMMA_R_MEDIATED_PHAGOCYTOSIS	96	-0.36	-1.29	0.2023576	0.233387	0.942	5766	tags=35%, list=10%, signal=39%
KEGG_T_CELL_RECEPTOR_SIGNALING_PATHWAY	108	-0.37	-1.29	0.2417154	0.232782	0.942	5658	tags=36%, list=10%, signal=40%
KEGG_NOTCH_SIGNALING_PATHWAY	47	-0.39	-1.28	0.204	0.232557	0.945	6206	tags=40%, list=11%, signal=45%
KEGG_METABOLISM_OF_XENOBIOTICS_BY_CYTOCHROME_P450	69	-0.44	-1.28	0.1869919	0.232755	0.948	9275	tags=48%, list=17%, signal=57%
KEGG_TYROSINE_METABOLISM	42	-0.39	-1.27	0.1925466	0.237008	0.949	8565	tags=43%, list=15%, signal=51%
KEGG_FRUCTOSE_AND_MANNOSE_METABOLISM	34	-0.4	-1.27	0.2465753	0.236697	0.951	6592	tags=38%, list=12%, signal=43%
KEGG_PRIMARY_BILE_ACID_BIOSYNTHESIS	16	-0.57	-1.27	0.2186235	0.236554	0.952	9576	tags=75%, list=17%, signal=91%
KEGG_GALACTOSE_METABOLISM	26	-0.39	-1.25	0.2151395	0.246437	0.959	6460	tags=38%, list=12%, signal=44%
KEGG_SPLICEOSOME	127	-0.37	-1.24	0.2893204	0.251827	0.965	5917	tags=38%, list=11%, signal=42%
KEGG_PENTOSE_AND_GLUCURONATE_INTERCONVERSIONS	28	-0.46	-1.23	0.2270833	0.260109	0.971	8325	tags=46%, list=15%, signal=55%
KEGG_PENTOSE_PHOSPHATE_PATHWAY	27	-0.39	-1.23	0.2165992	0.258327	0.971	7664	tags=41%, list=14%, signal=47%
KEGG_CHEMOKINE_SIGNALING_PATHWAY	188	-0.33	-1.23	0.2604374	0.256842	0.971	3364	tags=22%, list=6%, signal=24%
KEGG_TOLL_LIKE_RECEPTOR_SIGNALING_PATHWAY	102	-0.33	-1.22	0.272381	0.256379	0.971	3193	tags=22%, list=6%, signal=23%
KEGG_HEDGEHOG_SIGNALING_PATHWAY	56	-0.33	-1.22	0.2269939	0.257477	0.973	6299	tags=30%, list=11%, signal=34%
KEGG_PATHOGENIC_ESCHERICHIA_COLI_INFECTION	56	-0.37	-1.2	0.2862903	0.269637	0.979	4543	tags=30%, list=8%, signal=33%
KEGG_GLUTATHIONE_METABOLISM	49	-0.35	-1.19	0.2648221	0.277484	0.979	7105	tags=37%, list=13%, signal=42%
KEGG_DILATED_CARDIOMYOPATHY	90	-0.3	-1.18	0.2757936	0.282301	0.983	7799	tags=32%, list=14%, signal=37%
KEGG_PHENYLALANINE_METABOLISM	18	-0.39	-1.16	0.2651822	0.300186	0.987	8279	tags=39%, list=15%, signal=46%
KEGG_PORPHYRIN_AND_CHLOROPHYLL_METABOLISM	41	-0.39	-1.15	0.2929293	0.302215	0.987	8325	tags=41%, list=15%, signal=49%
KEGG_HYPERTROPHIC_CARDIOMYOPATHY_HCM	83	-0.3	-1.15	0.2837022	0.301032	0.987	7930	tags=31%, list=14%, signal=37%
KEGG_ECM_RECEPTOR_INTERACTION	84	-0.34	-1.14	0.3171717	0.30657	0.988	7995	tags=36%, list=14%, signal=42%
KEGG_BASAL_TRANSCRIPTION_FACTORS	35	-0.35	-1.12	0.3339882	0.326106	0.994	8802	tags=51%, list=16%, signal=61%
KEGG_AMINO_SUGAR_AND_NUCLEOTIDE_SUGAR_METABOLISM	44	-0.35	-1.11	0.3346303	0.329005	0.994	7957	tags=41%, list=14%, signal=48%
KEGG_BASAL_CELL_CARCINOMA	55	-0.3	-1.11	0.3536585	0.332638	0.994	5120	tags=25%, list=9%, signal=28%
KEGG_AMYOTROPHIC_LATERAL_SCLEROSIS_ALS	53	-0.28	-1.1	0.3202358	0.333824	0.994	6352	tags=26%, list=11%, signal=30%
KEGG_CELL_ADHESION_MOLECULES_CAMS	131	-0.3	-1.1	0.3444882	0.333563	0.994	5199	tags=24%, list=9%, signal=26%
KEGG_CELL_CYCLE	125	-0.32	-1.08	0.3847695	0.348574	0.996	4790	tags=30%, list=9%, signal=33%
KEGG_GLYCOSAMINOGLYCAN_BIOSYNTHESIS_HEPARAN_SULFATE	26	-0.32	-1.07	0.3514056	0.360097	0.996	3951	tags=23%, list=7%, signal=25%
KEGG_ABC_TRANSPORTERS	44	-0.28	-1.06	0.375502	0.370552	0.996	8483	tags=34%, list=15%, signal=40%
KEGG_NOD_LIKE_RECEPTOR_SIGNALING_PATHWAY	62	-0.3	-1.04	0.4177694	0.382693	0.998	4965	tags=29%, list=9%, signal=32%
KEGG_NEUROACTIVE_LIGAND_RECEPTOR_INTERACTION	270	-0.21	-1.01	0.4132232	0.407654	0.999	6205	tags=18%, list=11%, signal=20%
KEGG_MATURITY_ONSET_DIABETES_OF_THE_YOUNG	25	-0.31	-1.01	0.4391218	0.412286	0.999	10857	tags=48%, list=20%, signal=60%
KEGG_PARKINSONS_DISEASE	130	-0.34	-0.99	0.4705882	0.432061	0.999	9368	tags=46%, list=17%, signal=55%
KEGG_PRION_DISEASES	35	-0.32	-0.98	0.4702381	0.429514	0.999	8123	tags=40%, list=15%, signal=47%
KEGG_OXIDATIVE_PHOSPHORYLATION	132	-0.36	-0.95	0.5009901	0.461702	1	9368	tags=47%, list=17%, signal=56%
KEGG_GLYCOSPHINGOLIPID_BIOSYNTHESIS_GANGLIO_SERIES	15	-0.33	-0.94	0.4928425	0.467185	1	9721	tags=53%, list=18%, signal=65%
KEGG_STEROID_HORMONE_BIOSYNTHESIS	55	-0.28	-0.83	0.7246377	0.602937	1	9275	tags=42%, list=17%, signal=50%

KEGG_DRUG_METABOLISM_OTHER_ENZYMES	51	-0.26	-0.78	0.7556468	0.659239	1	8433	tags=41%, list=15%, signal=49%
KEGG_GLYCOSAMINOGLYCAN_BIOSYNTHESIS_KERATAN_SULFATE	15	-0.26	-0.7	0.8253968	0.749804	1	1774	tags=13%, list=3%, signal=14%
