# Supplementary figures and images for: A Novel Prognostic Ferroptosis-Related Long Noncoding RNA Signature in Clear Cell Renal Cell Carcinoma
Source: J Oncol. 2022 Feb 22;2022:6304824. doi: 10.1155/2022/6304824 (PMC8888116; doi:10.1155/2022/6304824)

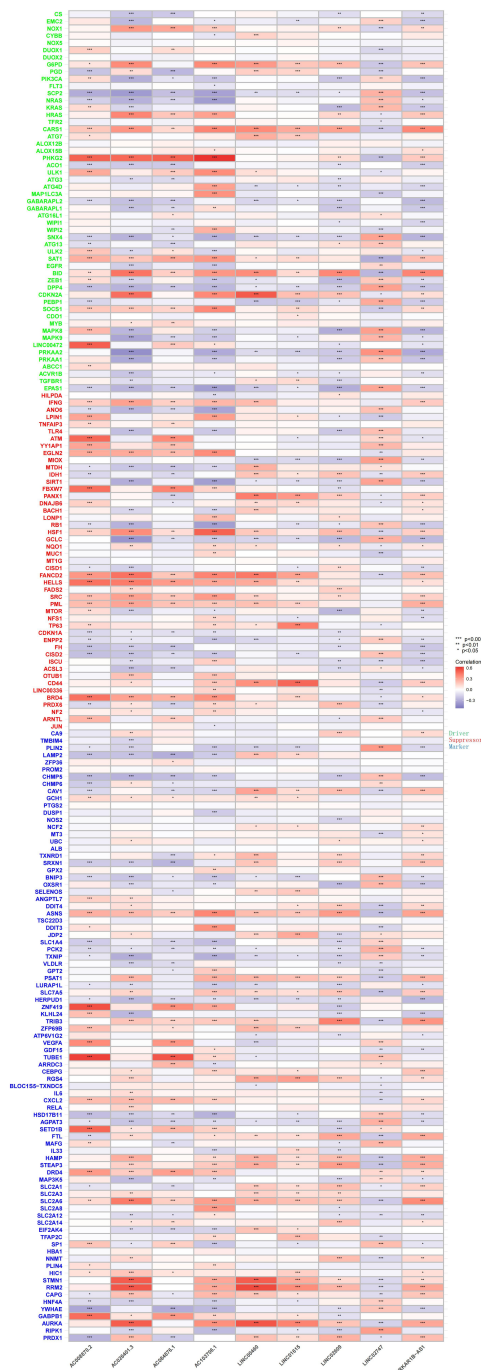

Supplement: Supplementary Materials — Table S1: 259 ferroptosis-related genes in FerrDb. Table S2: 76 ferroptosis-related genes in TCGA-KIRC. Table S3: GO and KEGG analysis in DEGs. Table S4: 1502 FRLRS in TCGA-KIRC cohort. Table S5: univariate and multivariate Cox analyses. Table S6: FRLRS set enrichment analyses and immunity gene expression. Figure S1. The heatmap of the correlations between ferroptosis-related lncRNA and genes. [file 6304824.f1.zip › 6304824.f1/Figure s1.pdf]
